# On‐Target Photoassembly of Pyronin Dyes for Super‐Resolution Microscopy

**DOI:** 10.1002/anie.202506894

**Published:** 2025-09-09

**Authors:** Gergely Knorr, Mariano L. Bossi, Stefan W. Hell

**Affiliations:** ^1^ Department of Optical Nanoscopy Max Planck Institute for Medical Research Jahnstrasse 29 69120 Heidelberg Germany; ^2^ Department of NanoBiophotonics Max Planck Institute for Multidisciplinary Sciences Am Fassberg 11 37077 Göttingen Germany

**Keywords:** Dyes, Fluorescence, Photoactivation, Pyronin, Super‐resolution microscopy

## Abstract

Controlled photoactivation is an auspicious and emerging approach in super‐resolution microscopy, offering virtually zero background signal from the marker prior to activation. Pyronins are well‐established fluorophores, but due to their inherent intercalating tendency towards nucleic acids, their use has been mostly avoided in super‐resolution microscopy. Here, we describe a new class of diaryl ether and diaryl silane molecules that upon photoactivation close into fluorescent (silicon−)pyronins and term them Pyronin Upon Light Irradiation (PULI). This concept exploits the outstanding photophysical properties of pyronins (bright, photostable, and optimal spectral features for standard microscopes), while overcoming their major drawback (intrinsic affinity of accumulating in the nucleus and around RNA) for the design of fluorescent markers for imaging applications. Furthermore, we also demonstrate that this approach is applicable to their Si‐bridged analogues, extending this family of photoactivatable molecules to the far‐red regime. The versatility of our approach was also highlighted by tagging diverse biological targets in cells and visualizing them using advanced super‐resolution microscopy techniques, such as PALM, STED, and MINFLUX.

## Graphical abstract

We report a new class of diaryl ether and diaryl silane compounds bearing hydrazone substituents that, upon irradiation, cyclize to a fluorescent (silicon−)pyronin dye. After optimizing their photophysical properties, we introduced reactive tags into their structure for biological labeling, and demonstrated their utility in STED, PALM, and MINFLUX super‐resolution microscopy.

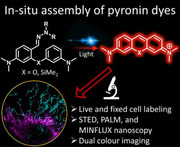

## Introduction

**Scheme 1:**
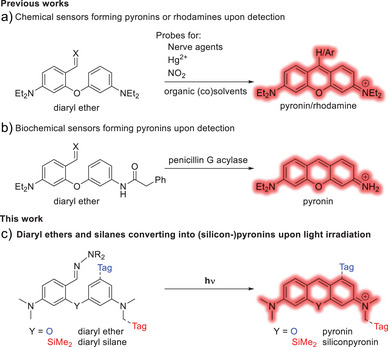
Previously described a) chemical and b) biochemical fluorescent sensors based on the assembly of a pyronin chromophore to different analytes, and c) light probes created in this paper. Our compounds also include a handle (Tag) to enable labelling and (super‐resolution) imaging of biological samples.

Super‐resolution microscopy has become an invaluable tool in cellular and molecular biology, providing insights into complex biological processes, in particular the dynamic organization of cellular (sub‐)structures, with a spatial resolution surpassing the diffraction limit. A key feature shared by all established super‐resolution methods is the controlled switching of fluorescent markers between dark (off) and bright (on) molecular states.^[^ ^1^ ^]^ Single‐molecule‐based methods (e.g., MINFLUX,^[^ ^2^ ^]^ PALM,^[^ ^3^ ^]^ STORM,^[^ ^4^ ^]^ PAINT^[^ ^5^, ^6^ ^]^) require a tight control over these states to achieve a sparse subset of markers in the bright one. Common strategies to achieve such control rely on the photoinduced blinking of fluorophores (cyanines,^[^ ^7^, ^8^, ^9^ ^]^ oxazines,^[^ ^10^, ^11^, ^12^ ^]^ rhodamines^[^ ^13^, ^14^ ^]^) in dedicated buffers, spontaneously blinking dyes,^[^ ^15^, ^16^, ^17^ ^]^ or binding of diffusing probes (PAINT),^[^ ^18^, ^19^, ^20^ ^]^ and have been recently reviewed in the literature.^[^ ^21^, ^22^, ^23^, ^24^, ^25^ ^]^ Redox and oxygen scavenging buffers and DNA‐PAINT approaches are challenging for live‐cell labelling and imaging, and self‐blinking processes usually depend on the environment (e.g., the pH), however notable efforts have been made to overcome these challenges.^[^ ^15^, ^26^, ^27^ ^]^ Photoactivatable dyes, a class of fluorophores that can be selectively and irreversibly converted from a dark state to a bright and fluorescent one by the effect of light, have recently emerged as a powerful alternative.^[^ ^28^ ^]^ They provide high spatio‐temporal control of the off–on process and eliminate the need for specific buffers and high irradiation intensities of light. Beyond imaging applications in fluorescence nanoscopy, photoactivatable dyes are valuable molecular tools in live cell tracking,^[^ ^29^, ^30^, ^31^ ^]^ spectral multiplexing,^[^ ^32^, ^33^ ^]^ and as quantification tools in opto‐pharmacology,^[^ ^34^ ^]^ photopatterning,^[^ ^35^ ^]^ and photoactivatable chemical sensors.^[^ ^36^, ^37^ ^]^ In general, photoactivatable dyes rely on a photocleavable or photoremovable group,^[^ ^38^ ^]^ also referred to as a caging group, whose purpose is to quench or disrupt the fluorescence of the fluorophore. By elimination of this group using light as the trigger or stimulus, the fluorescence is reinstated. Such strategies have been applied to several common dye families, such as coumarin,^[^ ^39^, ^40^ ^]^ BODIPY,^[^ ^41^, ^42^, ^43^ ^]^ cyanine,^[^ ^44^, ^45^, ^46^ ^]^ and various xanthene^[^ ^33^, ^47^, ^48^ ^]^ scaffolds. Pyronin Y (Py‐Y) is a bright and photostable fluorophore, with excellent water solubility, and is smaller in size compared to other xanthenes (e.g. fluoresceins, rhodamines), with similar spectral properties. However, its cationic nature and compact planar shape imparts a high affinity for nucleic acids, in particular towards RNA. Therefore, its *in cellulo* use has been mostly limited to applications for DNA and RNA staining, and thus has been mostly disregarded for further developments, with a few exceptions. The conjugation system of the fluorescent core of (silicon−)pyronin analogues can be disrupted by the addition of a thiol^[^ ^16^ ^]^ or an exo double bond formation with a carbon^[^ ^49^ ^]^ or an oxygen,^[^ ^50^ ^]^ on the *meso* position. The photoinduced elimination of the thiol, protonation, or reaction of the excited ketone with an appropriate radical trap, respectively, leads to recovery of the fluorescence. However, these methods depend on a pre‐formed pyronin core. The covalent assembly of diaryl ethers into pyronin dyes has been exploited for the design of fluorogenic sensors (Scheme 1). Specifically, the Yang group (Scheme 1a) has developed a series of diaryl ether molecules forming a pyronin or rhodamine molecule, in the presence of Sarin, Hg^2+^ or radicals.^[^ ^51^, ^52^, ^53^ ^]^ A similar covalent assembly was also described for protease activity sensing by the Romieu group (Scheme 1b).^[^ ^54^, ^55^, ^56^, ^57^, ^58^ ^]^

Inspired by these works, we sought to utilize the condensation mechanism of diaryl ethers and silanes to pyronins by using light as the sole trigger. The aim was to induce precise spatiotemporal control into the photoreaction, while avoiding the off‐targeting pitfalls of traditional pyronin dyes. Here, we report the design of a new class of turn‐on fluorescence probes, based on the photoinduced assembly of diaryl ethers and diaryl silanes into bright and stable (silicon−)pyronin fluorophores (Scheme 1c). With sensitivity down to the single molecule level, termed **PULI** (**P**yronin **U**pon **L**ight **I**rradiation), these photoactivatable dyes can be prepared from readily available starting materials via a simple and straightforward synthetic route. We also incorporated reactive groups to convert these probes into effective labels for imaging in fixed (silanes) and living cells (ethers), and demonstrated their use in confocal, stimulated emission depletion (STED), photoactivated localization microscopy (PALM), and minimal fluorescence photon fluxes (MINFLUX) techniques.

## Results and Discussion

### Design, Synthesis and Characterization

An optimal photoactivatable compound should undergo a complete and clean photoinduced conversion to a bright and photostable fluorophore, as incomplete reactions or side products can compromise image quality and make quantitative analysis difficult. If a competing thermal reaction exists, its rate should be minimized under analysis conditions to prevent premature off‐target activation. Additionally, the compound should have a moderately high photoactivation quantum yield to avoid the need for extreme irradiation doses during activation and to minimize unwanted activation during sample preparation and handling. We sought to prepare two sets of molecules, which activate to either a pyronin or a silicon–pyronin dye (Scheme 1c). Both pyronin Y (tetramethyl pyronin) and its silicon analogue^[^ ^59^ ^]^ have absorption maxima (546 and 634 nm) close to the light sources commonly used in fluorescence microscopy, enabling efficient excitation and signal detection. In an ideal setup, both diaryl precursors (O‐ and Si‐) for (silicon−)pyronins could be activated simultaneously with the same activation wavelength, with subsequent formation and detection of the (silicon−)pyronins using different excitation lasers and channels, thus enabling multicolour imaging.

**Scheme 2:**
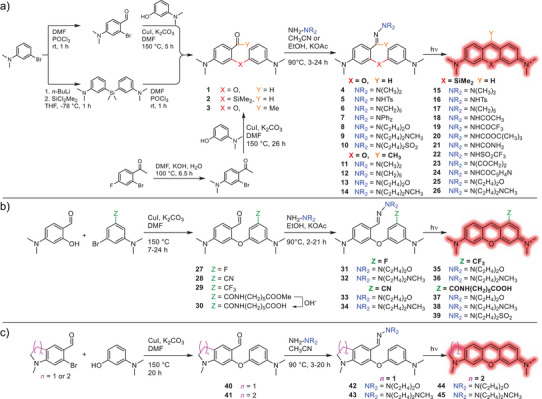
a) Synthesis of model compounds **1**–**26**. b) Synthesis of electron deficient hydrazones. c) Synthesis of structurally rigidized hydrazones.

As a starting point, we synthesized the diaryl ether aldehyde **1** (Scheme 2a), first reported by Yang and Romieu (a precursor for their chemical or enzymatic sensors) by means of an Ullmann coupling. Upon exposure to acidic aqueous media, we observed that this molecule already formed the Py‐Y dye by an intramolecular self‐condensation, a phenomenon that to the best of our knowledge had not yet been described. Moreover, this self‐condensation also occurs at neutral pH (Figure S1), albeit at a much slower rate.

Unexpectedly, UV light irradiation at 365 nm increased the reaction rate of the self‐condensation of **1** by approximately 30%, suggesting the involvement of an alternative pathway via an excited‐state mechanism. This observation prompted us to investigate alternative functional groups as substitutes for the aldehyde, which would facilitate the photoreaction, while at the same time slow or even suppress the thermal closing reaction in aqueous media. Imines are generally not suitable, as they are prone to hydrolysis in aqueous conditions. Oximes^[^ ^60^ ^]^ are usually more stable, but irradiation of oxime **SI‐2** yielded an imino‐xanthone as the main product (Table S1), with only a small amount of Py‐Y as the by‐product. Our attention came to hydrazones as their use as photocaged diazo compounds for the synthesis of tertiary alkyl organoboronates and diaryl alkanes^[^ ^61^, ^62^ ^]^ in organic solvents had been recently demonstrated. The simplest hydrazone (‐NMe_2_, compound **4**) successfully produced the expected Py‐Y product (Figure S2), with a considerably increased rate compared to aldehyde **1**. Therefore, after thorough optimization, a library of diaryl ether hydrazones (**4–14, 31–45**) and diaryl silane hydrazones (**15**–**26**) was prepared to explore the effects of the hydrazone substituent (Scheme 2a), the effect of electron withdrawing groups (Scheme 2b), and structure rigidification (Scheme 2c) on the reaction rates and on the fluorescence properties of the products. The general synthesis of these compounds was carried out by heating the corresponding *o*‐carbonyl diaryl ether or silane with an excess of the substituted hydrazine (NH_2_‐NR_2_), in acetonitrile or in mildly basic ethanol (see the Supporting Information for detailed information).

Following this, we carried out a detailed study of the photoconversion under irradiation with 365 nm light, as well as measuring the rate of the thermal conversion, if present, in neutral aqueous solutions (pH = 7). All compounds were found stable for months in dry DMSO stock solutions, the thermal reaction occurs only in aqueous media.

**Figure 1:**
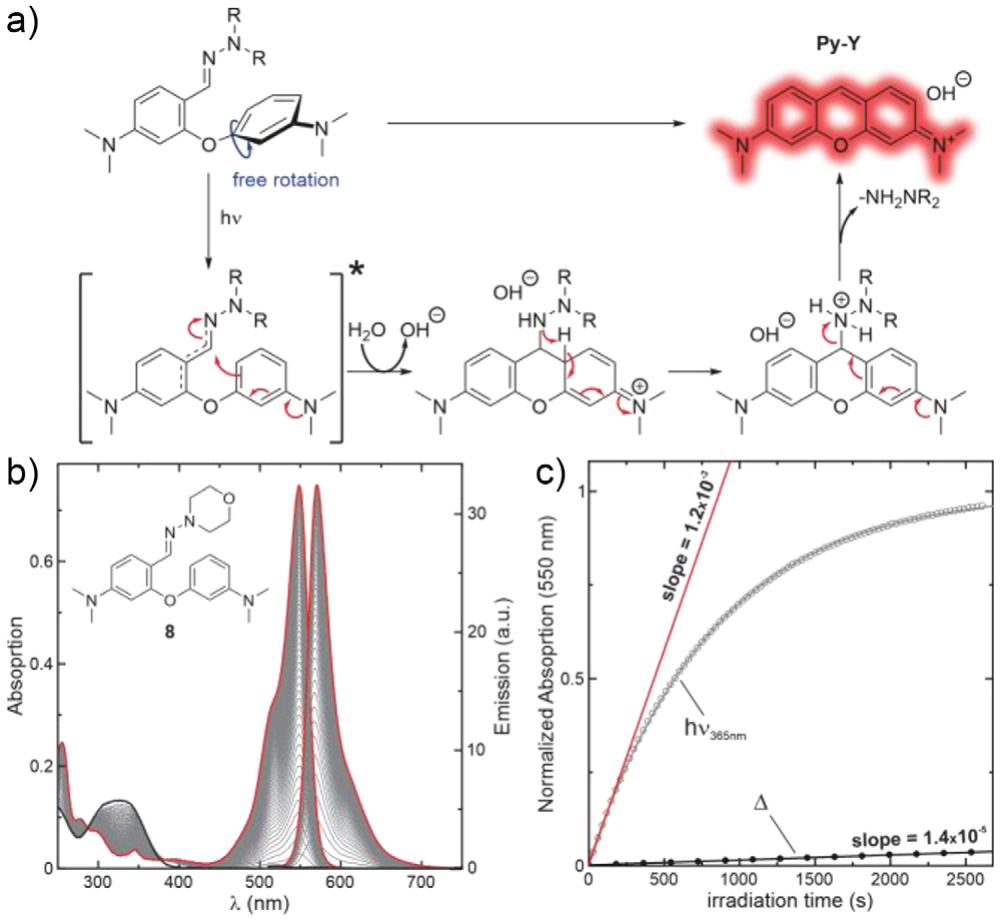
a) Proposed photoreaction mechanism of pyronin Y formation. b) Absorption and emission changes for the photoinduced conversion of compound **8** into pyronin Y, in an aqueous solution at pH = 7.0 (black lines, initial state; red lines, final state; gray lines, intermediate irradiation times). c) Comparison of the initial rates of the photoinduced reaction (hollow circles, red line) and the thermal reaction (filled circles, black line).

According to our proposed reaction mechanism (Figure 1a), excitation enhances the electron‐withdrawing properties of the hydrazone, thereby increasing the electrophilicity of the aldiminyl carbon. This facilitates an intramolecular attack by the electron‐rich aromatic carbon in the *para* position of the adjacent dimethyl aniline ring, leading to a ring‐closing reaction. After protonation from solvent and a bond rearrangement, the hydrazine departs and the newly formed pyronin core undergoes aromatization. We propose a similar mechanism for the thermal reaction, but with the protonation of the hydrazone from the solvent being the initiating step, which increases the electrophilicity of the aldiminyl carbon, priming it for cyclization. This is supported by the fact that pyronin formation is accelerated in acidic solution. However, further extensive studies such as ultrafast transient absorption spectroscopy,^[^ ^63^, ^64^ ^]^ would be required to confirm the mechanism proposed in Figure 1a.

Next, we tested diaryl ether hydrazones **5**–**10** (Scheme 2a). Compounds **5**–**7** had low solubility in aqueous solutions and failed to produce the expected Py‐Y product. In contrast, compounds **8**–**10** converted quantitatively to the desired fluorescent Py‐Y product (see Figure 1b,c for photoconversion of **8**). Moreover, the thermal (dark) ring‐closing reaction to Py‐Y of compounds **8**–**10** was slowed down by a factor of 2–4 in comparison to aldehyde **1**, while the photoinduced reactions were accelerated by over an order of magnitude (Table 1; compare also Figures 1C and S1). This resulted in an overall difference between the thermal and photoreactions of almost two orders of magnitude for the irradiation intensity used. Despite this significant difference, the thermal reaction of hydrazones **8**–**10** was still too rapid, so we anticipated a significant self‐condensation during live cell labelling at 37 °C, thus we sought to further reduce the thermal reaction rate. For this, we formally replaced the aldehyde of compound **1** with a methyl‐ketone (**3**), which gave rise to hydrazones **11**–**14**. This approach slightly improved the photoactivation rates with respect to compounds **8–10**, but unfortunately also resulted in an acceleration of the thermal reaction (See Table S1).

**Table 1:** Summary of the photophysical properties measured in aqueous buffer (phosphate 100 mM, pH = 7.0) of the best performing compounds. The pyronin product is derived from photoactivation (*λ* ^ACT^ = 365 nm) of the corresponding diaryl ether or silane.

	Diaryl ether/silane			Pyronin product		
Comp.	$\lambda_{Abs}^{max}$ (nm)	*k*_Δ_ × 10^−5^ (s^−1^)	*Φ*_PA_ × 10^−4^	$\lambda_{Abs}^{max}$/$\lambda_{Em}^{max}$ (nm)	*Φ*_Fluo_	*τ*_Fluo_ (s)
**1**	353	4.3	0.13	546/565	0.42	1.83
**4**	317	1.6	7.5			
**8**	326	1.7	8.4			
**9**	327	1.5	8.0			
**10**	322	0.55	10.7			
**37**	326	0.44	1.20	557/574	0.33	1.46
**38**	328	0.27	0.62			
**39**	327	0.12	0.56			
**42**	327	1.7	4.8	544/564	0.75	3.15
**44**	345	1.5	4.3	550/568	0.76	3.06
**2**	361	n.r.	n.d.	635/650	0.55	2.60
**15**	326	n.r.	n.d.			
**25**	322	n.r.	0.81			
**26**	330	n.r.	0.46			

Note: $\lambda_{Abs}^{max}$: absorption maximum, $\lambda_{Em}^{max}$: emission maximum, *k*_Δ_: first order thermal reaction rate, *Φ*_PA_: photoactivation quantum yield, *Φ*_Fluo_: fluorescence quantum yield, *τ*_Fluo_: fluorescence lifetime, n.r.: not reacting, n.d.: not determined.

To hinder the thermal activation rate, we next aimed to reduce the electron density on the nucleophilic attacking ring by placing electron withdrawing groups (F, CN, CF_3_, CONH(CH_2_)_5_COOH) *ortho* to the reactive carbon (Scheme 2b). We synthesized the diaryl ether aldehydes (**27**–**30**) by Ullmann coupling of 4‐(diethylamino)salicylaldehyde with the appropriate bromo‐dimethylaniline derivative. From these aldehydes, hydrazones **31**–**39** were synthesized via a slightly modified approach. This strategy effectively slowed down the thermal reaction, albeit with a reduction in the quantum yield of the photoinduced reaction. The slowest thermal and fastest photoactivation rates were measured for diaryl ether hydrazones **37–39**, bearing an amide group and an alkyl carboxylic acid linker (Table 1) (For compounds **31**–**36** see Table S1). The addition of electron withdrawing groups caused a slight bathochromic shift in the absorption maximum of the resulting pyronin products, from 546 to 552–557 nm with respect to the parent Py‐Y. This shift made them more suitable candidates for imaging applications, due to the improvement in the excitation and detection efficiency of excitation sources (i.e., a 561 nm laser) commonly available in commercial microscopes.

It is well known that the fluorescence quantum yield of dimethylamino‐xanthene dyes is hampered by twisted intramolecular charge transfer (TICT).^[^ ^65^ ^]^ This mechanism can be blocked through structural rigidification or by hindering the free rotation of the amino group.^[^ ^66^, ^67^ ^]^ To improve the fluorescence quantum yield of the pyronin dyes, we chose to rigidify the structure by incorporating the amino group into a fused ring system (Scheme 2c). To this end, we synthesized diaryl ether aldehydes **40**–**41** and converted them into hydrazones **42**–**45**. We observed an almost two‐fold enhancement in the fluorescent quantum yield of the resulting pyronins derived from hydrazones **42** (0.75) and **44** (0.76), compared to Py‐Y (0.42) in neutral aqueous solutions (Table 1). A small but noticeable bathochromic shift was observed for **44** (Figure S3), however the photoactivation quantum yield was halved, compared to **8**.

For imaging applications, in particular super‐resolution techniques, photostability is just as crucial as brightness. Thus, we assessed the fatigue resistance of the best pyronins derived from hydrazones **37**, **42,** and **44** with irradiation of 550 nm light. Compared to Py‐Y, pyronins with rigidized cores (dyes formed from **42** and **44**) displayed between 3‐ to 10‐fold poorer photostability. In contrast, the pyronin derived from the electron deficient amide substituted hydrazone **37** presented an over 3‐fold improvement (Figure S4A), again highlighting **37** as an ideal candidate for cellular labelling. The latter also shows superior photostability compared to established commercial dyes (e.g., Cy3B, Atto 565, JF 549), and similar to TMR (Figure S4B).

After the careful optimization of diaryl ethers, we turned to the evaluation of silane‐hydrazones **15**–**26**. Upon irradiation we observed a rapid decline in the absorption at their absorption maxima (∼320 nm) for most silanes (**16**–**24**) without significant ring closure to the expected silicon–pyronin (Si–Py). We measured a moderate photoactivation rate with dimethylamino hydrazone **15**, and slightly higher conversion for the morpholine and *N*‐methyl aminopiperazine bearing compounds **25** and **26**, respectively, indicating that these hydrazones may have general applicability as suitable fluorescent turn‐on probes and labels. Compared to their O‐bridged analogues **8–10**, their photoactivation rates were lower by about an order of magnitude, which can be explained by the increased distance between the two rings and the lower nucleophilicity of the dimethylsilanyl‐*N,N*‐dimethylaniline moiety due to the weaker electron donating ability of the Si‐bridge. As a result of this hindered reactivity, we could detect no thermal closing reaction for Si‐bridged compounds. As compounds **25** and **26** exhibited no thermal reactivity, but photoconverted to fluorescent Si–Py products with a moderate but acceptable rate, further optimization of these model compounds was unnecessary.

The quantum yield for the photoactivation (*Φ*_PA_) to pyronins of the selected compounds (Table 1) were in the order of 10^−3^–10^−5^, ranging at the lower end of the values presented by other photoactivatable compounds, such as siliconrhodamines^[^ ^49^ ^]^ (9 × 10^−4^), rhodamine NNs^[^ ^68^ ^]^ (1–10 × 10^−3^), thioketones^[^ ^69^ ^]^ (1 × 10^−2^) and *o*‐Nitroveratryloxycarbonyl‐rhodamines^[^ ^48^ ^]^ (3–6 × 10^−3^). For a more detailed comparison with other benchmarked compounds, please see Table S2.

In summary, diaryl ether hydrazones **37**–**39** and diaryl silane hydrazones **25**–**26** were identified as ideal model compounds. These compounds displayed a fast and efficient photoconversion to the corresponding pyronin fluorophore while either limiting or completely averting the undesired thermal reaction. Thus, they were selected for further development as photoactivatable markers for cell labelling and imaging experiments.

### Reactive Adducts for Fixed and Live Cell Labelling

To demonstrate the biological availability of our compounds, we sought to further modify them with reactive tags. We first started with diaryl ethers **37–39**, which have a major associated advantage that a carboxylic acid for the introduction of reactive groups is already available on them, rendering further modifications unnecessary. Biological labelling and imaging effectiveness can be influenced by many factors, such as permeability and microenvironment on the target, which are hard to consider in advance. As we found minimal differences between their photophysical properties, we decided to test the three compounds. Transformation of the free acids **37**–**39** gave rise to their respective NHS esters (**46**–**48**) and HaloTag ligands (**49**–**51**).

**Figure 2:**
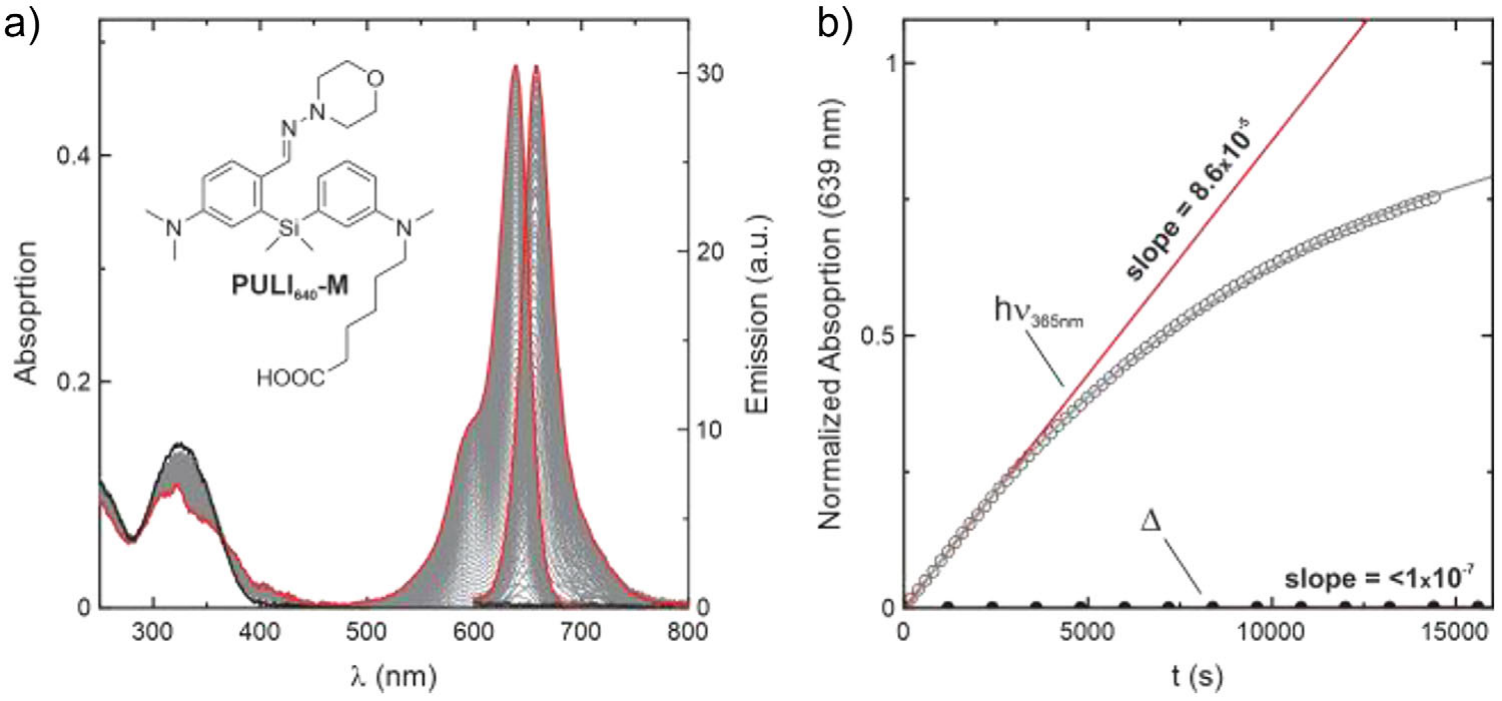
a) Absorption and emission changes for the photoinduced conversion of **52** into the corresponding silicon–pyronin, in aqueous solution at pH=7.0. b) Comparison of the initial rates of the photoinduced reaction (hollow circles, red line) and almost imperceptible thermal reaction (filled circles, black line).

As silane model compounds **25** and **26** did not have any free reactive substituent, their core required further modifications for the incorporation of a suitable functionality for labelling. Based on previous reports on silicon–pyronin markers,^[^ ^16^ ^]^ we decided to incorporate an alkyl‐carboxylic acid linker attached to the amino group. In this way, we prepared a new set of silanes with morpholine, N‐Me piperazine and thiomorpholine 1,1‐dioxide hydrazone substituents (**52**–**54**) (Scheme S2) (see Supporting Information). Subsequent reaction of the resulting free carbocyclic acids gave NHS esters (**55**–**57**) and HaloTag ligands (**58**–**60**). In comparison to **25**, compound **52** showed a significantly faster and more complete conversion to the corresponding Si–Py under UV irradiation, with a barely observable thermal reaction (Figure 2).

**Scheme 3:**
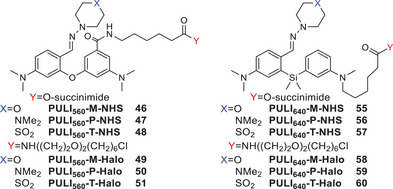
PULI dyes for biological labelling experiments.

Based on their intrinsic property of displaying a photoinduced conversion into (silicon−)pyronin dyes, we named our new family of photoactivatable molecules **PULI**. We also introduced a new naming system for a better description of the molecules for cell labelling and imaging. We named the compounds derived from aldehydes **30** and **SI‐35 PULI_560_** and **PULI_640_**, respectively, where the 560 and 640 correspond to the excitation wavelength of the nearest commercially available laser as this is their last common precursor molecule. Hydrazones with morpholine, piperazine, or thiomorpholine 1,1‐dioxide groups are indicated by **M, P,** and **T,** respectively. Finally, the reactive tag is indicated with an ‐**NHS** or ‐**Halo** extension (Scheme 3). Regrettably, **PULI_560_‐M/P/T‐NHS** compounds, though successfully labeled antibodies, thermally activated within a day after labelling, rendering them unusable for further photoactivation experiments and therefore have been excluded from further experiments.

### Cellular Imaging with PULI Labels

**Figure 3:**
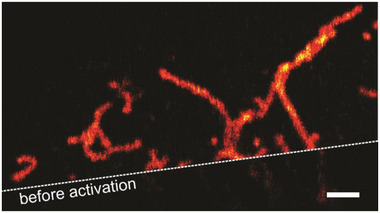
Confocal images of live U2OS cells stably expressing a Tomm20‐HaloTag7 construct. Cells were labelled live with **PULI_560_‐P‐Halo** for 90 min, washed (30 min) and imaged in cell medium before (bottom) and after (top) photoactivation. Scale‐bar: 2 µm.

To evaluate the compatibility of the PULI compounds with live cell labelling and imaging, we first tested **PULI_560_‐P‐Halo** on a stable cell line expressing Halotag7 on Tomm20, a protein of the translocase of the outer membrane complex located on the outer mitochondria membrane. Cells were labeled and imaged in cell media without further additives (Figure 3). Confocal images before photoactivation (Figure 3, bottom) reveal a small subset of thermally (pre‐)activated dyes, which is likely resulting from the handling of the dye during labelling (Figure S5). Short exposure (ca.5s) to UV light using the lamp of the microscope in combination with a DAPI filter (350–402 nm), successfully induced complete photoactivation of the markers resulting in the bright and fluorescent pyronin product (Figure 3, top). Similar results were obtained with **PULI_560_‐M‐Halo** and **PULI_560_‐T‐Halo** (Figure S6). Their labelling specificity was further confirmed through colocalization experiments with a standard mitochondrial marker (Figure S7). These results confirmed that our markers can be used for live cell labelling and that the in situ photoassembly of pyronin can be performed in the complex live cell environment, and in particular it is compatible with a self‐labelling enzyme.

**Figure 4:**
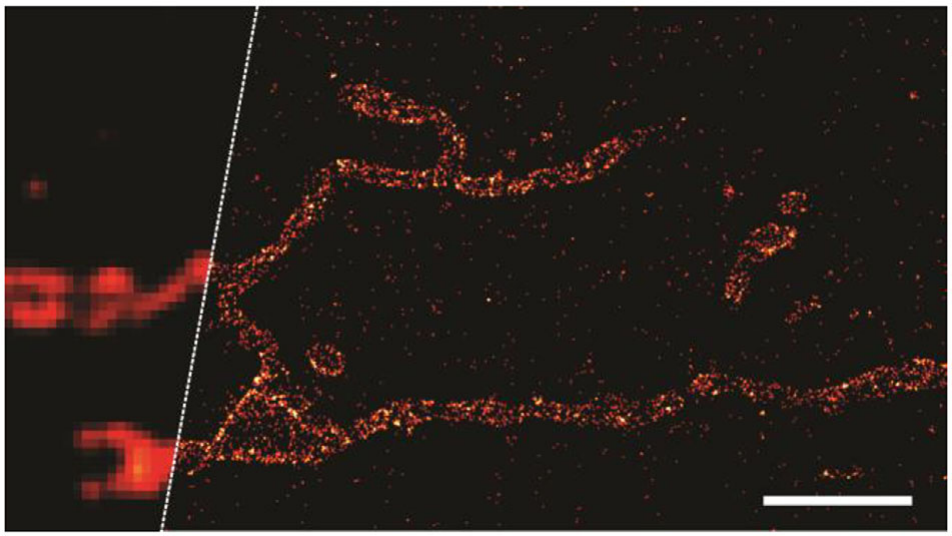
PALM image of U2OS cells stably expressing a Tomm20‐HaloTag7 construct. Cells were labelled live with **PULI_560_‐T‐Halo**, fixed and imaged in PBS at pH 8.5 in a widefield microscope. The widefield image (left) is the first frame recorded, seen is a small proportion of thermally activated dye. This fraction is rapidly bleached allowing the system to reach a single molecule regime (the first 50 frames were discarded for rendering of the super‐resolution image) for the acquisition of the PALM image. For relevant quantitative metrics see Figure S10 and Table S3. Scale‐bar: 2 µm.

Next, PULI dyes were used for super‐resolution PALM imaging. Utilizing the same live labelling strategy, samples were fixed prior to imaging to avoid artefacts from the movement of the imaged structures during the measurement (Figure S8) that may impair image quality and resolution. To reduce long‐term thermal activation of the samples after fixation, samples were mounted and imaged in PBS buffer with pH adjusted to 8.5. In this way, the samples were stable for several hours. As our commercial microscope is equipped with a 405 nm laser source for photoactivation, we first evaluated if the PULI photoassembly can be also performed at this wavelength. **PULI_560_‐M** (**37**) was irradiated in similar conditions with UV (365 nm) and violet (405 nm) light, and the product obtained was analysed by LCMS (Figure S9). We confirmed that irradiation under both conditions produced the same pyronin product. Naturally, irradiation at 405 nm proceeded with slower kinetics due to a substantial lower absorption of **PULI_560_‐M** at this wavelength, compared with 365 nm. We also observed a lower photoconversion that can be attributed to photobleaching of the product induced by 405 nm light, as the pyronin product has a larger absorption at this wavelength than the starting compound. However, for imaging methods based on single molecule detection like PALM, molecules are bleached by the excitation light after their position are assessed (i.e., only sparse subsets of molecules are activated), therefore this bleaching is not problematic. Indeed, PALM images acquired in a commercial microscope with 561 nm excitation and 405 nm activation (detection at 576–620 nm), show a remarkable quality (Figures 4, and S10–S12) comparable to other established fluorescent markers (Figure S6), with a mean localization uncertainty of 12 nm and 1600 photons per localization on average. The results also show a good response of the PULI activation to the 405 nm intensity step‐raising events (Figure S6D), and a good match of the on‐times (1.4 frames) to the select frame time of 20 ms (Figure S6B). Importantly, imaging was successfully carried out in aqueous buffer, without the addition of any blinking auxiliary agent or additive, nor any photostabilization coadjuvant.

**Figure 5:**
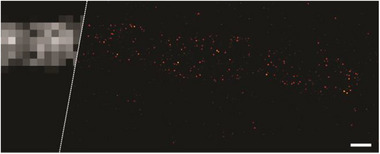
Confocal (left) and MINFLUX image (right) of U2OS cells stably expressing a Tomm20‐HaloTag construct. Cells were labelled live with **PULI_560_‐T‐Halo**, fixed and imaged in PBS at pH 8.5. The confocal image was recorded with the small fraction of thermally activated dye, which was bleached prior to the acquisition of the MINFLUX image. For relevant quantitative metrics see Figure S13, and Table S3. Scale‐bar: 200 nm.

Widefield super‐resolution methods, such as PALM, can routinely achieve on commercial microscopes a single molecule localization precision of 10–20 nm. A more recent and advanced technique, MINFLUX nanoscopy, can improve that value around an order of magnitude on common and commercial microscopes, reaching single‐digit nanometre localization uncertainty.^[^ ^70^ ^]^ By using a doughnut‐shaped excitation beam, where the position of the central node is known, MINFLUX can establish the position of the fluorophores with typically two orders of magnitude less photons than camera‐based methods (for the same precision). This can be used to reduce the burden of photons produced by the fluorophore, or if a similar number of photons is produced, to increase the resolution. Thus, we tested our PULI compounds in a commercial MINFLUX microscope, with 560 nm excitation and 405 nm activation, in the detection range of 580 to 630 nm (a window typically used for Cy3 emitters). For a fairer comparison, we chose to use the Tomm20‐HaloTag7 construct in U2OS cells again, labelling with **PULI_560_‐T‐Halo** with the same conditions as used in PALM. After a quick bleaching of the small pool of self‐activated dyes that allowed for recording a confocal image of the structure, a MINFLUX image (Figure 5) was acquired, showing a much crisper distribution of Tomm20 clusters on the mitochondrial membrane. Fluorophores were localized on average 30 times, producing 168 photons per localization on average, and yielding a mean localization uncertainty (standard deviation of a Gaussian fit) of 2.9 nm (Figure S13). Combining localizations of a single activation event can be eventually used to further improve the localization uncertainty.^[^ ^70^, ^71^ ^]^ The super‐resolution images can clearly discern Tomm20 clusters in the mitochondrial membrane, otherwise undiscernible in conventional images. This clustering behaviour of Tomm20 (an important receptor of the translocase of the mitochondrial outer membrane TOMM complex), has been validated by other super‐resolution techniques.^[^ ^72^ ^]^ In summary, we successfully demonstrated that the PULI_560_ compounds are compatible with MINFLUX, matching closely with the performance of other high‐quality photoactivatable dyes. Their reliable behaviour in this advanced imaging technique highlight their potential as valuable tools for super‐resolution microscopy.^[^ ^48^, ^50^, ^73^ ^]^

**Figure 6:**
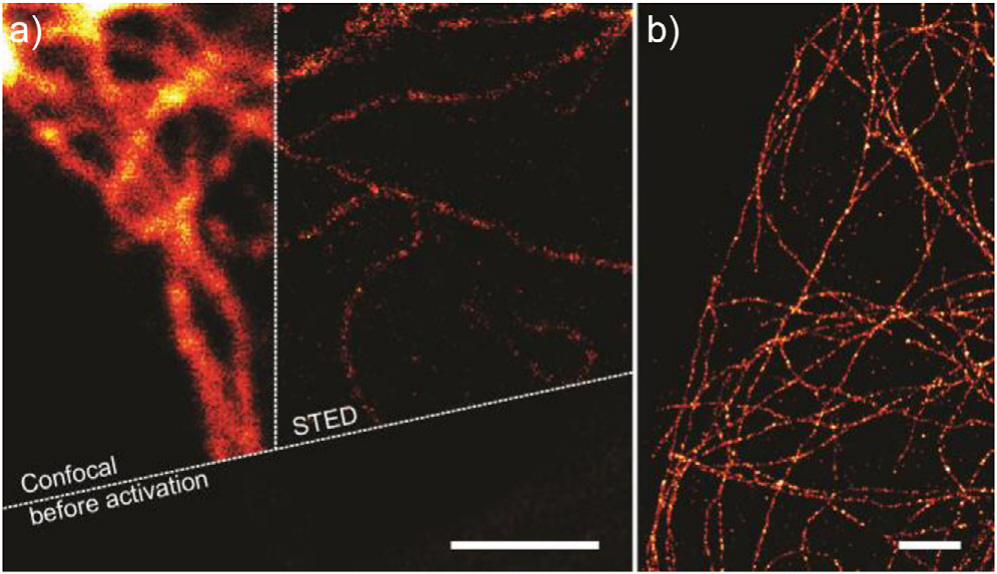
Conventional and super‐resolution imaging in fixed cells immunostained with a primary antibody against tubulin, and a secondary antibody labelled with **PULI_640_‐M‐NHS** a) Confocal image before (bottom) and after (left) photoactivation, and super‐resolution STED image (right). b) super‐resolution PALM image obtained on a different region. For relevant quantitative metrics for the PALM image see Figure S14, and Table S3. Scale‐bars: 2 µm.

Next, after evaluating the strength and wavelength limitations of PULI_560_ compounds, we investigated the silicon–pyronin forming PULI_640_ molecules as a next‐generation alternative, aiming for labelling versatility and far‐red detectability. To this end, we labelled secondary antibodies with **PULI_640_‐M‐NHS**. The adduct proved to be thermally stable for over a year (stored in the fridge in PBS pH = 7.4) with no observable colouration. We first imaged immunostained cells, in a confocal microscope, mounted in PBS buffer (Figure 6). From the image before activation, barely a molecule in its fluorescent form could be observed and no discernible structure could be detected (Figure 6a, bottom). Short exposure (ca.30s) to UV light using the lamp of the microscope (with DAPI filter), photoactivated the markers resulting in the bright image and revealing the fine tubulin filamentous structure (Figure 6a, top left). Super‐resolution STED imaging of the formed silicon–pyronin was possible, accounting for the high photostability of the ensembled fluorophore (Figure 6a, right).

Attempts to obtain PALM images with PULI_640_ compounds using 405 nm activation in a commercial microscope were unsuccessful, rendering very few single molecule activation events, even when the maximum available power for the activation laser was used. We hypothesised that the combination of the lower activation quantum yield and absorption coefficient at the activation wavelength results in a much lower activation rate than oxygen‐bridged PULI_560_ compounds. Thus, measurements were performed in a custom‐made microscope, with a 375 nm activation laser (for a description for the microscope, see the Supporting Information). This small (∼30 nm) blue‐shift in the activation wavelength resulted in a remarkable improvement on the number of activation events, successfully rendering a high‐quality STED image (Figure 6b). Unfortunately, MINFLUX imaging with PULI_640_ compounds could not be attempted due to the lack of an activation laser source below 405 nm. Nevertheless, these results demonstrate the potential extension of the PULI concept for imaging in the far‐red, and paves the way for future improvements of the activation rates of PULI_640_ family, either by increasing the activation quantum yield or by red‐shifting the absorption of the molecules, to increase the cross section at the excitation wavelength. Attempts to visualise HaloTag labelled targets were unsuccessful, likely due to either the lower cell permeability of the **PULI_640_‐M/P/T‐Halo** compounds, or that rotation to a planar position for ring closing was blocked after binding to the protein.

**Figure 7:**
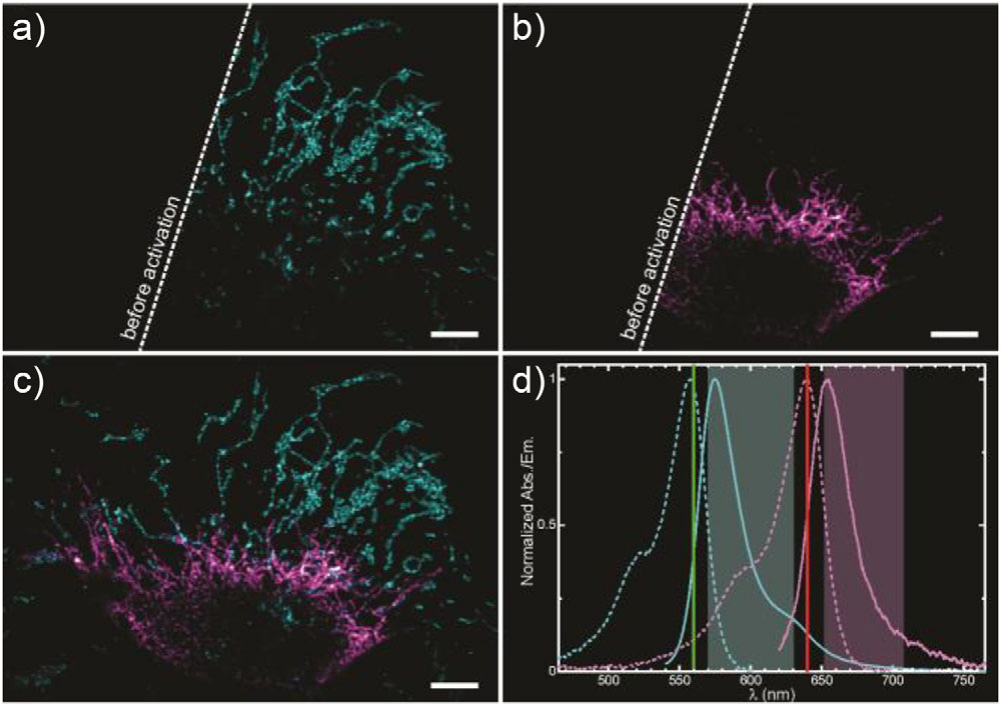
Two‐colour confocal images of Tomm20‐Halo cells labelled (live) with **PULI_560_‐P‐Halo**, fixed and then immunostained with a primary antibody against vimentin, and a secondary antibody labelled with **PULI_640_‐M‐NHS**. Images were recorded in an a) orange‐red and b) a far‐red channel, before (left) and after photoactivation (right). The combined image is displayed in c). Images were acquired simultaneously (line‐by‐line) with the channel configuration (spectra of photoactivated dyes, excitation lasers and the detection windows) shown in d). Scale‐bars: 5 µm.

Finally, we demonstrate the compatibility of our compounds for multicolour imaging. Labelling was performed combining the two strategies described above. We undertook live labelling of the TOMM20‐Halo proteins with **PULI_560_‐P‐Halo**. Then, the samples were fixed and immunostained with a secondary antibody labelled with **PULI_640_‐M‐NHS** targeting vimentin. This dye combination proved to be ideal for a standard dual colour acquisition, after simultaneous photoactivation with UV light, with negligible channel cross‐talk (Figure 7).

## Conclusion

In this paper, we introduced a family of photoactivatable compounds, which convert to Pyronin Upon Light Irradiation (PULI) via an intramolecular ring closing reaction. We created two sets of compounds, the diaryl ether PULI_560_ that converts to pyronins, and the diaryl silane PULI_640_ that converts to silicon–pyronins upon photoactivation. These two classes align perfectly for use in two of the most common imaging channels (i.e., 560 and 640 nm excitation) available for commercial microscopes. After careful optimization for the right substitution pattern, we determined that the highest photoactivation and lowest thermal self‐activation rate can be reached with morpholine, N‐Me piperazine and thiomorpholine 1,1‐dioxide hydrazones. We showed their wide biological applicability by labelling Tomm20 proteins on mitochondria, vimentin and microtubules in U2OS cells, using confocal, and the super‐resolution techniques STED, PALM, and MINFLUX. Without the need of special or dedicated buffers, we reached a spatial resolution of 12 nm in a PALM microscope, and down to 3 nm in a commercial MINFLUX microscope, which is comparable with commercial and other fluorophores developed for nanoscopy. Finally, to demonstrate the high versatility of their labelling capabilities, we successfully employed a combination of PULIs for two colour imaging. In summary, we introduce a new covalent assembly approach towards pyronin fluorophores by photoactivating intrinsically nonfluorescent precursor molecules, which enables the general use of (silicon−)pyronins for *in‐cellulo* imaging, what was previously not possible due to their off‐targeting effects.

## Conflict of Interests

The authors declare no conflict of interest.

## Supporting information



Supporting Information

## Data Availability

The data that support the findings of this study are available in the Supporting Information of this article.
