# Supplementary material for: On‐Target Photoassembly of Pyronin Dyes for Super‐Resolution Microscopy
Source: Angew Chem Int Ed Engl. 2025 Sep 9;64(44):e202506894. doi: 10.1002/anie.202506894 (PMC12559456; doi:10.1002/anie.202506894)

Supplementary Information

On-target Photoassembly of Pyronine Dyes for Super-resolution Microscopy

Gergely Knorr[a], Mariano L. Bossi[a*], Stefan W. Hell[a,b*]

[a] Department of Optical Nanoscopy, MaxPlanck Institute for Medical Research, 69120 Heidelberg, Germany

[b] Department of NanoBiophotonics, Max Planck Institute for Multidisciplinary Sciences, 37077 Göttingen, Germany

*Corresponding Authors

Table of Contents

Table of Contents 1

Supplementary Methods 2

Supplementary Figures 6

Supplementary Tables 18

General synthesis of O-bridged molecules 21

Synthesis and characterization of O-bridged compounds 22

Description for the Synthesis of Si-bridged compounds 59

Synthesis and characterization of Si-bridged compounds 61

NMR data 85

Supplementary Methods

General experimental information and synthesis

The reaction mixtures were heated using stirred silicone oil baths (Fisher Scientific, Cat. No. 11470673), pre-set to the specified temperature. Reactions conducted in a closed cap vials were heated in a 4ml Screw Vial (Art.-Nr.: 130400) from BGB Analytik Vertrieb GmbH, heated on a Heidolf magnetic stirrer plate.

Thin layer chromatography

Analytical TLC (normal phase) was carried out using Merck Millipore pre-coated aluminum sheets with silica gel 60 (F254) (Cat. No. 1.05554.0001). Compounds were visualized by exposing the TLC plates to UV light (254 or 366 nm) or by heating with 1 N HCl.

Preparative flash column chromatography

Automated separations on normal phase (silica gel) were conducted using an Isolera Spektra One system (Biotage AG, Sweden) with commercially available cartridges of appropriate size (BGB Scorpius 40-60 µm series from BGB Analytik, and Puriflash Silica HP 30 µm series from Interchim), following the specified solvent gradient.

High-Performance Liquid Chromatography (HPLC) and Mass Spectrometry (MS)

Analytical liquid chromatography-mass spectrometry (LC-MS) was performed using a Shimadzu system comprising 2x LC-20AD HPLC pumps, a DGU-20A3R solvent degassing unit, a SIL-20ACHT autosampler, a CTO-20AC column oven, an SPD-M30A diode array detector, and a CBM-20A communication bus module. The system was integrated with a CAMAG TLC-MS interface 2, an FCV-20AH2 diverter valve, and an LCMS-2020 spectrometer with electrospray ionization (ESI, 100–1500 m/z). The analytical column used was a Hypersil GOLD (50×2.1 mm, 1.9 µm), with standard conditions of a 1-2 µL sample volume, a solvent flow rate of 0.45 mL/min, and a column temperature of 30°C. General method: isocratic 90:10 A:B over 2 min, then gradient 90:10 to 1:99 A:B over 5 min, then isocratic 1:99 A:B over 2 min; solvent A – water + 0.1% (v/v) HCOOH, solvent B – acetonitrile + 0.1% (v/v) HCOOH.

High-resolution mass spectra (HRMS) were acquired using a Bruker maXis II ETD with electrospray ionization (ESI) and a quadrupole time-of-flight (QTOF) mass analyser at the Max-Planck Institute for Medical Research Mass Spectrometry Core facility (Heidelberg, Germany).

Preparative high-performance liquid chromatography (HPLC) was conducted using a Büchi Reveleris Prep system with preparative columns and specific conditions tailored for individual samples. Method scouting was carried out using either a Shimadzu HPLC system, which included 2x LC-20AD HPLC pumps, a DGU-20A3R solvent degassing unit, a CTO-20AC column oven, a manual injector with a 20 µL sample loop, an SPD-M20A diode array detector, an RF-20A fluorescence detector, and a CBM-20A communication bus module; or a Dionex Ultimate 3000 UPLC system equipped with an LPG-3400SD pump, a WPS-3000SL autosampler, a TCC-3000SD column compartment with 2× 7-port 6-position valves, and a DAD-3000RS diode array detector. Analytical columns with matching phases were used for test runs: HPLC columns included Interchim 250×4.6 mm 10 µm C18HQ and Interchim 250×4.6 mm 5 µm PhC4, with a solvent flow rate of 1.2 mL/min; UPLC columns included Interchim C18HQ or PhC4 75×2.1 mm 2.2 µm, and ThermoFisher Hypersil GOLD 100×2.1 mm 1.9 µm, with a solvent flow rate of 0.5 mL/min.

NMR spectra

NMR spectra were recorded at 25 °C using a Bruker Ascend 400 spectrometer at 400.15 MHz for ^{1}H, 376.52 MHz for ^{19}F, and 100.62 MHz for ^{13}C, with chemical shifts reported in ppm. All ^{1}H spectra were referenced to tetramethylsilane (TMS) as the internal standard (δ = 0.00 ppm). For ^{13}C spectra, tetramethylsilane (δ = 0 ppm) was used as a reference, with the solvent signals from $CDCl_3$ (77.16 ppm), CD_3CN (1.32 ppm), or DMSO-d_6 (39.52 ppm). Signal multiplicities are indicated as follows: s = singlet, d = doublet, t = triplet, q = quartet, m = multiplet or overlapping resonances, and bs = broad signal. Coupling constants *J* are given in Hz.

Optical spectroscopy

Absorption spectra were measured using a Varian Cary 4000 UV-Vis double-beam spectrophotometer (Agilent Technologies, USA), emission spectra were obtained using a Varian Cary Eclipse fluorescence spectrophotometer (Agilent). Both types of spectra were recorded in quartz cuvettes with a 10 mm light path (Hellma Analytics, Cat. # 119-10-40). All spectra were acquired at 25 °C in air-saturated solvents and were background corrected.

Fluorescence quantum yields (absolute method determinations) were measured using a Quantaurus-QY absolute PL quantum yield spectrometer (model C11347-11, Hamamatsu) following the manufacturer's instructions. Fluorescence lifetimes were recorded with a FluoTime 300 fluorescence lifetime spectrometer (PicoQuant) controlled by EasyTau1.4 software. All measurements were carried out in air-saturated solvents at ambient temperature.

Photolysis experiments

Solutions in phosphate buffer (100 mM, pH = 7.0; 10 µM) were irradiated in a previously described home-built setup[74-75] with a 365 nm (M365L2, Thorlabs Inc.) and 405 nm LED source (M405L3, Thorlabs Inc.). During the irradiation, samples were maintained at 20 °C and continuously stirred with a Peltier-based temperature-controlled cuvette holder (Luma 40, Quantum Northwest, Inc.). The absorption and emission of irradiated solutions were monitored at desired irradiation intervals with a fiber-based spectrometer (Flame-S-UV-Vis-ES, Ocean Insight). For absorption measurements, a deuterium and tungsten halogen source was used for illumination (DH-2000-BAL, Ocean Insight), and fluorescence excitation was performed in a 90° configuration with an LED source (MINTL5 or M625L3, Thorlabs Inc.) in combination with an appropriate bandpass filter (FBH560-10 or FL632.8-1, Thorlabs Inc.). Data collection and analysis was performed with custom-made routines in Matlab. Samples for LCMS or ESI-MS analysis were taken before and after the photolysis. Thermal reactions were measured in the same way, but without irradiation with the 365 nm or 405 nm LED.

Antibody conjugation

Secondary antibodies were coupled with NHS-esters of the indicated dye using a standard protocol. In brief, the pH of 420 µL of a 2.4 mg/ml antibody solution (Goat anti-rabbit IgG, 111-005-003, Jackson Immunoresearch), containing 1 mg of antibody, was adjusted with 40 µL of carbonate buffer (1M $NaHCO_3$ at pH = 8.4), and immediately mixed with 30 µg of the dye (7-8 equivalents) dissolved in 8 µl of DMSO. The mixture was stirred in the dark at room temperature for 1 h, and the protein was separated from the unreacted dye via size-exclusion chromatography with a Sephadex G-25 column (PD Minitrap, 28918007, Cytiva), using PBS (pH = 7.4) as the elution buffer (1 ml).

The degree of labelling (DOL) of obtained antibodies were estimated by UV-Vis absorption measurements of the conjugates in a small-volume spectrometer (DS-11+, DeNovix), assuming the absorption of the conjugated dye is identical to the one of the corresponding free carboxylate dye, and

the absorption coefficient of the protein at 280 nm is 210000 $M^{-1}cm^{-1}$ (typical for an IgG). The DOL's obtained ranged between 4-6 dye molecules/protein.

Cell culture

U2OS and U2OS FlpIN TREx Tomm20-Halo[76] cells were cultured in Dulbecco's Modified Eagle Medium (DMEM) with high glucose, GlutaMAX Supplement, and pyruvate (ThermoFisher, Cat. # 10569010), supplemented with 10% (v/v) fetal bovine serum (FBS, ThermoFisher, Cat. # 10500064) and 1% penicillin-streptomycin (ThermoFisher, Cat. # 15140122). Cells were maintained in a humidified incubator at 37 °C with 5% CO_2. They were split when they reached 80-90% confluency and routinely tested for mycoplasma contamination. The cells were grown on glass coverslips for 48 hours before labelling. Tomm20-Halo cells were induced with doxycycline (100 ng/ml) 36–48 hours before labelling.

Live-cell labelling and imaging

Cells were incubated in the dark for 60–90 min with the respective fluorescent HaloTag-ligands diluted from concentrated DMSO stock solutions (1 µM) in culture medium (without phenol red) to a final concentration of 2 µM, and then washed with cell culture medium for ca. 15-30 minutes. For live-cell imaging, the samples were mounted in a magnetic imaging chamber (CM-B18-1, Live Cell Instrument Co.) with Fluorobrite (A1896701, Gibco) supplemented with 10% (v/v) FBS (10500064, ThermoFisher), 2% (v/v) GlutaMAX (35050061,Gibco) and 1% (v/v) penicillin/streptomycin. For fixed-cell imaging (e.g. PALM, MINFLUX), fixation was performed with a 4% paraformaldehyde solution in PBS (pH = 8.5) at room temperature for 15 min, then incubated with quenching solution (0.1 M NH_4Cl and 0.1 M glycine in PBS) for 5 min at room temperature, and finally rinsed and mounted in PBS (pH = 8.5) in a magnetic imaging chamber (CM-B18-1, Live Cell Instrument Co.). For MINFLUX measurements, the samples were incubated 5 min with a 150 nm gold beads solution (BBI Solutions, EM.GC150), used by the microscope for sample and beam stabilization, and then rinsed three times with PBS (pH = 8.5).

Fixed-cell labelling and imaging

Samples were covered with cold (-20 °C) MeOH and left at room temperature for 5 minutes, washed with PBS (3×5 min), and blocked for 30 min at room temperature in blocking buffer (2% bovine serum albumin in PBS). The samples were then incubated for 1 h at room temperature with a primary antibody (monoclonal rabbit anti-alpha tubulin, ab18251, Abcam; or monoclonal rabbit anti-Vimentin, ab92547, Abcam), diluted (1:100) in blocking buffer. After washing with blocking buffer (3×5 min), the cells were incubated with the conjugated secondary antibody (dilution 1:100 in blocking buffer) for 1 h at room temperature, and finally washed with blocking buffer (2×5 min) and PBS (2×5 min). The sample was mounted with PBS in a magnetic imaging chamber (CM-B18-1, Live Cell Instrument Co.).

Confocal, PALM and MINFLUX microscopy

Confocal and STED (stimulated emission depletion) imaging

Confocal and STED imaging was performed on an Abberior Expert Line (Abberior Instruments GmbH, Göttingen, Germany) built on a motorized inverted microscope IX83 (Olympus, Tokyo, Japan). The microscope is equipped with a pulsed STED laser at 775 nm shaped by a Spatial Light Modulator (SLM), and with 561 nm and 640 nm excitation lasers. Spectral detection was performed with avalanche photodiodes (APD) in the indicated spectral windows. Images were acquired with a 100x/1.40 UPlanSApo Oil immersion objective lens (Olympus). Pixel size was 70 nm for confocal images, and 30 nm for STED images. Laser powers and dwell times, as well as line accumulations, were optimized for each sample. Imaging and image processing were performed with ImSpector software (v. 16.3.13367; Abberior Instruments GmbH, Göttingen, Germany). Images are displayed as raw data.

Photoactivated localization microscopy (PALM) imaging

PALM imaging was performed using two different setups. First, a commercial ONI Nanoimager V3 (Oxford Nanoimaging, Oxford, UK) was used. The microscope is equipped with a 100X NA 1.45 objective lens, and a Hamamatsu Orca Flash4.0 v3 Digital CMOS camera. Excitation was performed with a 561 nm laser, activation with a 405 nm, and detection in the 575-620 nm range. Exposure time was set to 20 ms, the illumination angle used was 52.5° (HILO), and the sample's temperature stabilized to 28°C. The laser powers measured before the objective lens were 180 mW for the excitation laser, and the activation was increased up to 8 mW (at the end of the measurement) depending the density of localizations observed for each sample. The second custom-built widefield setup used was previously described.[77] In brief, the microscope is equipped with an inverted IX83 microscope body (Olympus), an 100x/1.4 objective lens (Olympus, UPLSAPO100XO), with a z-drift compensator unit (Olympus, IX3-ZDC), and an EMCCD camera (Oxford Instruments, Andor iXonEM+ DU-897D-CSO-BV). The setup has a 640 nm excitation laser (HÜBNER Photonics, 05-01 Cobolt Bolero), and a 375 nm activation laser (Coherent, CUBE 375-16C). The beam angle is controlled via a motorized stage enabling total internal reflection fluorescence (TIRF) and highly inclined and laminated optical sheet (HILO) illumination. Detection was selected using a set of two quad-band filters (AHF analysentechnik, F72-832 and F73-832S) to a range of 660-750 nm. A custom-written LabVIEW program (National Instruments, version 20.0.1f1) controls all components of the setup.

The data was analysed with ThunderSTORM plugin2 on ImageJ (version 1.52p). In brief, sub-pixel localization of the molecules was performed with a maximum likelihood fitting method (PSF integrated method, fitting radius of 3 pixels, initial sigma 1.6). Then the data was drift-corrected based on the cross-correlation method of the plugin, merged within the size of 50 nm with 1 off-frames allowed, and sigma values were filtered to converge to a normal Gaussian distribution function (typically 75 nm < sigma < 175 nm). No further filters were applied. Final images were renderd using normalized Gaussian rendered with a fixed sigma of approximately the mean localization uncertainty.

MINFLUX Imaging

MINFLUX data were recorded on a commercial Abberior Instruments MINFLUX setup (Abberior Instruments GmbH), similar to the one reported by Schmidt et al..[78] The system was equipped with a 561 nm continuous-wave (CW) laser for excitation and a 405 nm CW laser for activation. Detection was performed with a 580-630 nm window. A neutral density filter (ND2) was installed in front of the fiber to attenuate the 405 nm laser intensity. Data acquisition was performed with a standard imaging sequence provided by the microscope vendor. Measurements were started without activation (405 nm) for 30-60 sec before the activation power was gradually increased during data acquisition, based on the frequency of localizations observed. Gold nanoparticles were used to monitor the beam and actively stabilize the samples. The Abberior Instruments Imspector software with MINFLUX drivers was used to operate the system.

Data analysis and image rendering was performed using dedicated Matlab routines as previously described.[75, 79] A first filter was applied to the list of localizations using the Matlab implementation of the density-based clustering algorithm dbscan (epsilon = 6 nm, minPts = 4). A second filter was applied to include only molecules which provided at least 3 localizations, and to discard localizations outside a radius of 6 nm around their mean position. The final image was produced using an amplitude-normalized Gaussian rendering method from the list of filtered localizations, with a fixed sigma value of 3 nm (corresponding approximately to their average localization uncertainty of 2.9 nm) and a pixel-size of 1 nm for the rendered image. For ease of visualization, the image in Figure 7 is displayed with a colormap with a nonlinear (gamma correction with A = 1 and γ = 0.5) brightness progression.

Supplementary Figures

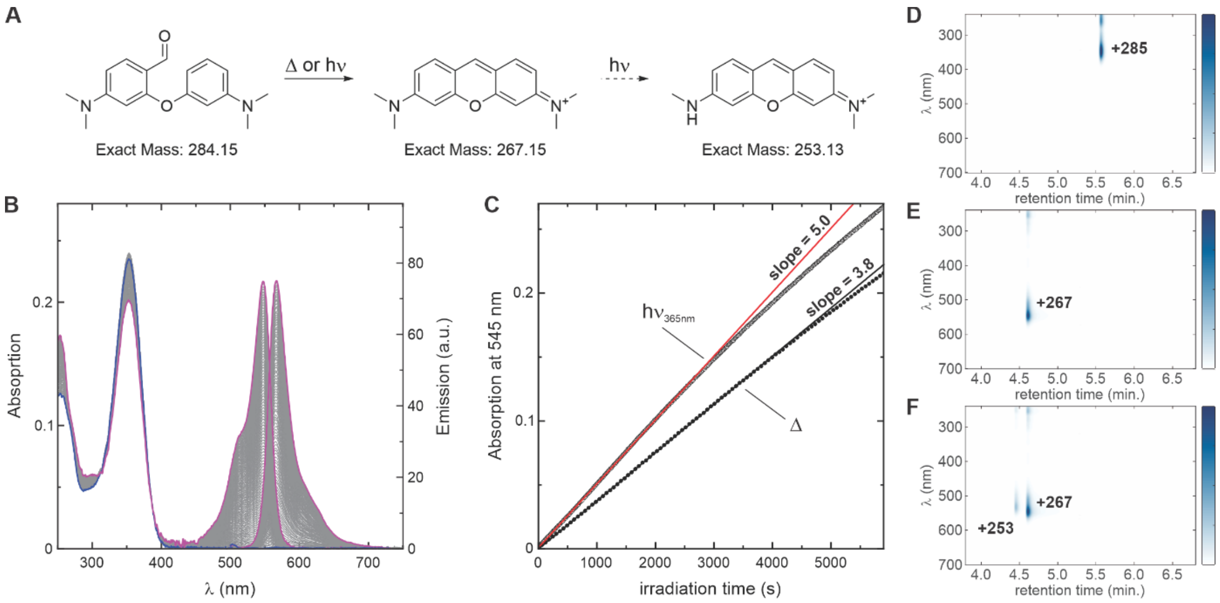

Figure S1. (A) Thermal and photoinduced conversion of compound **1** into pyronin Y in an aqueous solution buffered at pH = 7.0. (B) Absorption and emission changes up to 5800 s. (C) Initial rates for the thermal and photoinduced reaction. (D-F) 2D plots of the LCMS analysis of the starting solution (D), after complete thermal (E) and photoinduced (F) reactions. The m/z of the main peak for each reactant is presented. A small amount of de-methylated by-product is observed[80] in (F), produced by photodecomposition of the main product (pyronin Y) by 365 nm irradiation light, as indicated in (A).

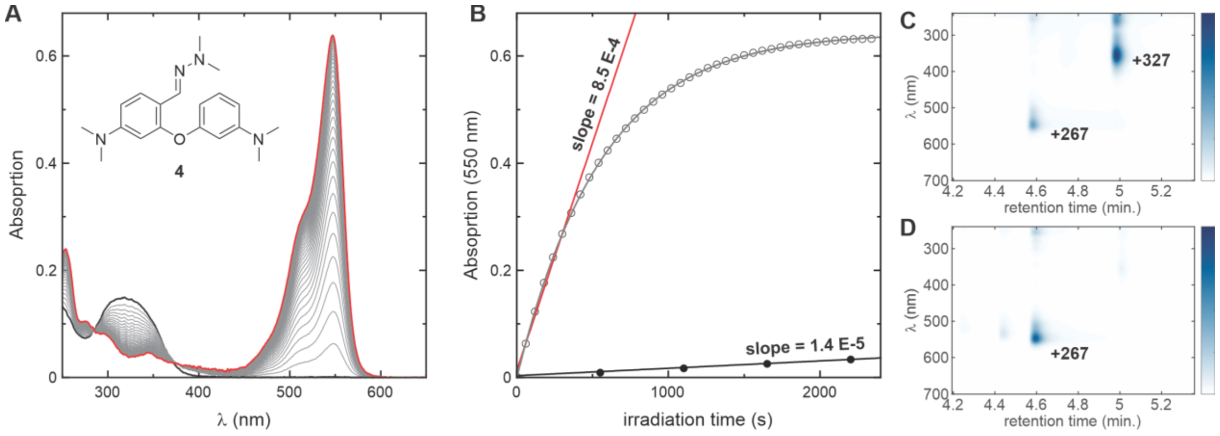

Figure S2. Thermal and photoinduced conversion of compound **4** into pyronin Y in an aqueous solution buffered at pH = 7.0. (A) Absorption changes up to 2500 s. (B) Initial rates for the thermal (black) and photoinduced (red) reaction. (C) 2D plots of the LCMS analysis of the starting solution (D) after photoinduced reaction. The m/z of the main peak for each reactant is presented. A small amount of de-methylated by-product is observed[80] in (D), produced by photodecomposition of the main product (pyronin Y) by 365 nm irradiation light.

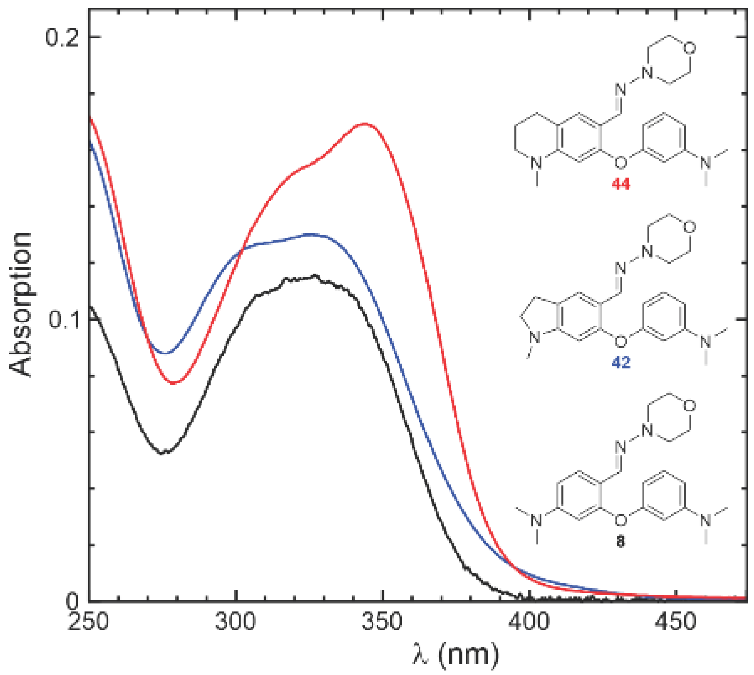

Figure S3. Comparative absorption of compounds **8** (black line), **42** (blue line) and **44** (red line) in an aqueous solution at pH = 7.0.

Photostability measurement

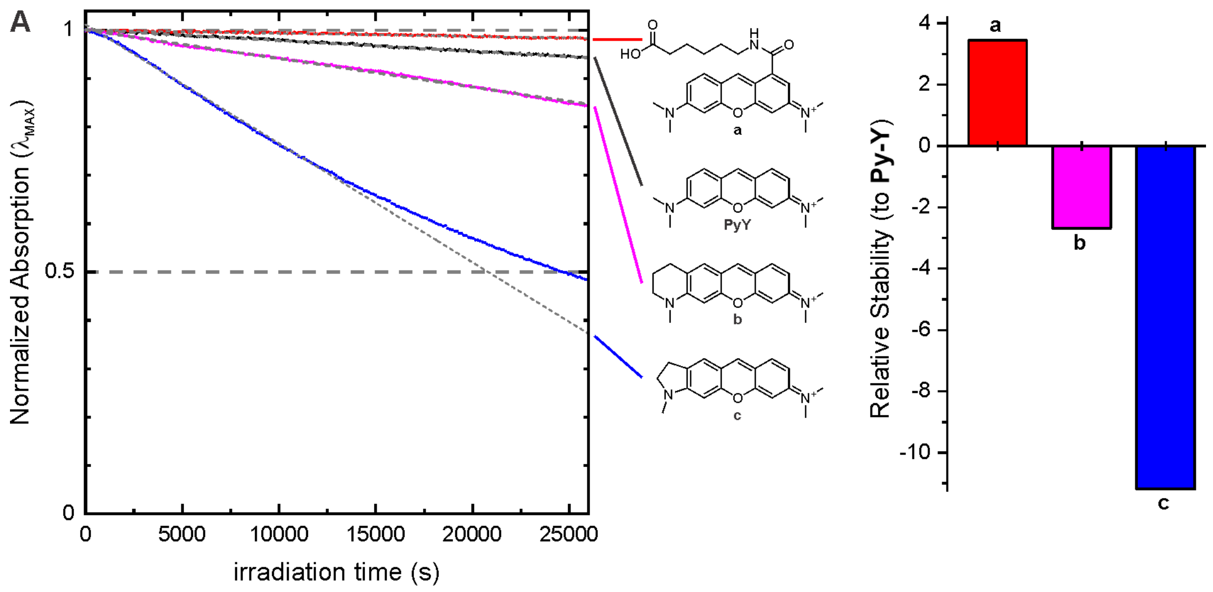

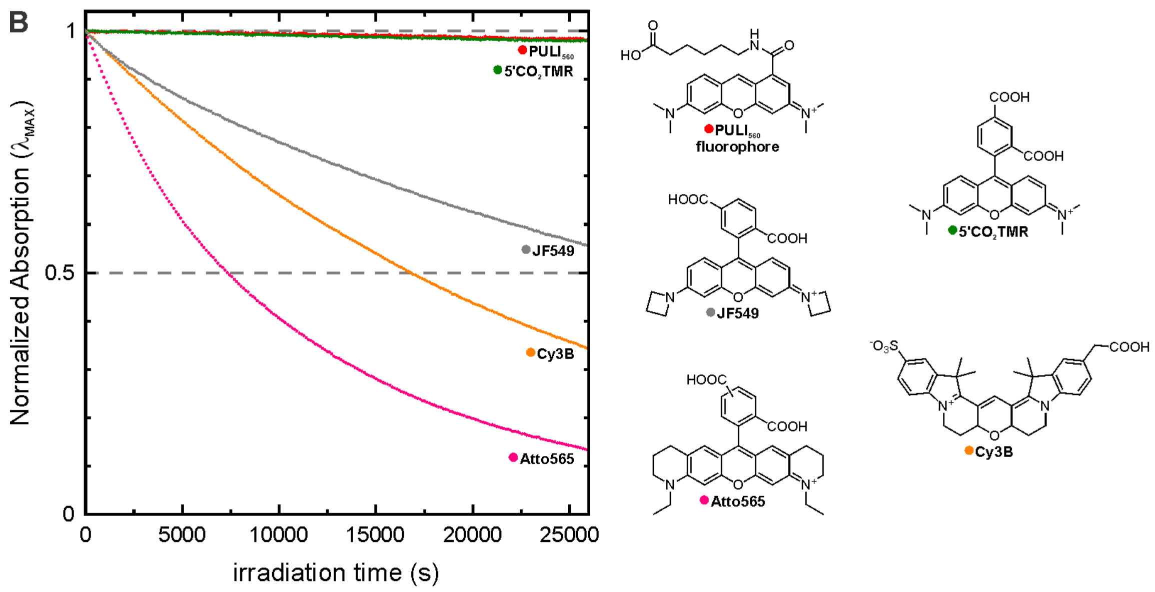

Figure S4. (A) Photochemical stability of the corresponding pyronin dyes obtained from compounds **37** (**A**), **44** (**B**) and **42** (**C**) in neutral buffered aqueous solutions. Samples were irradiated with light of 550 ± 20 nm. The bargraph shows the relative photostability introduced by the respective chemical modification to the frame of Py-Y (obtained from compound **8**), estimated as the decrease (positive values) or the increase (negative values) of the bleaching rates. All bleaching rates are calculated from the initial decomposition rates (linear fits shown in the figure), and the slopes are indicated under each compound structure. Dyes were prepared by diluting the DMSO stock solution of the corresponding compound in aqueous solutions and waiting (ca. 4 days) until the thermal closing reaction was complete. We avoided photoactivation of the compounds to eliminate the possibility of any side reactions or partial photobleaching of the product dyes. The purity of the dyes **A, B, C** was checked with LCMS prior to measurements. (B) Photochemical stability of our best pyronin dye (obtained from compounds **37**, the fluorescent core used in **PULI$_{560}$** labels) compared with commercial and established dyes with similar spectral properties. The chemical structure provided by the vendor is presented. All fluorophores were irradiated in similar conditions.

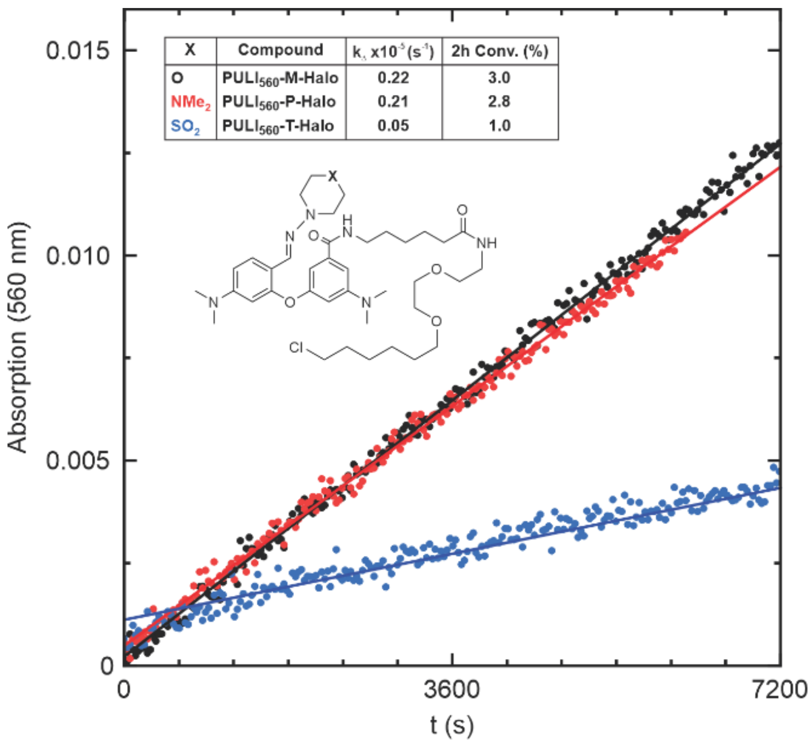

Figure S5. Thermal activation of **PULI$_{560}$ M/P/T-Halo** compounds. Absorption changes measured in PBS buffer (pH = 7.4) at 37°C. The initial rates (k_{Δ}), calculated as those reported in Table 1 and Table S1, and the conversion after 2 hours is tabulated.

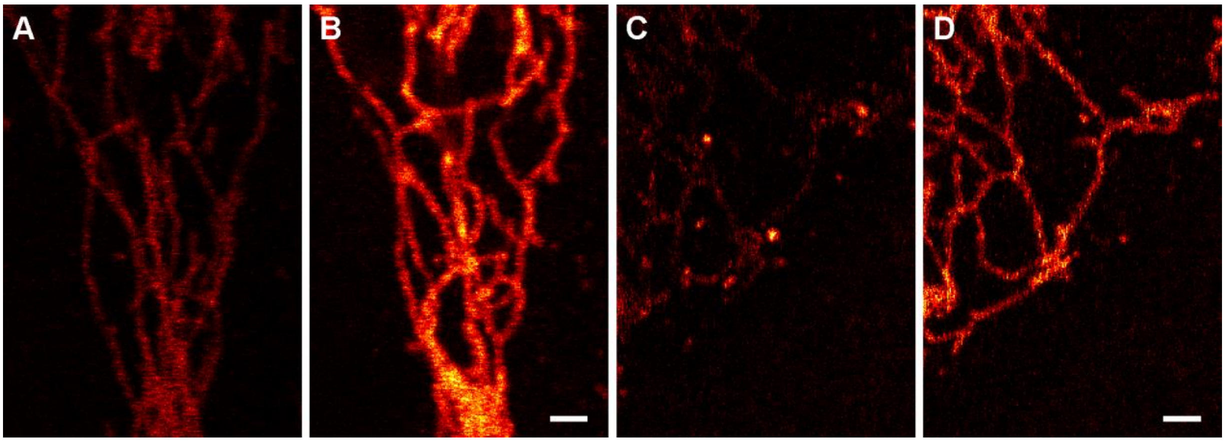

Figure S6. Confocal images of live U2OS cells stably expressing a Tomm20-HaloTag construct. Cells were labelled live with **PULI$_{560}$-M-Halo** (A-B) or with **PULI$_{560}$-T-Halo** (C-D) for 90 minutes, washed (30 min) and imaged in cell medium before (A, C) and after (B, D) photoactivation. Images are displayed with the same intensity scale (before/after activation). Scalebars: 2 µm.

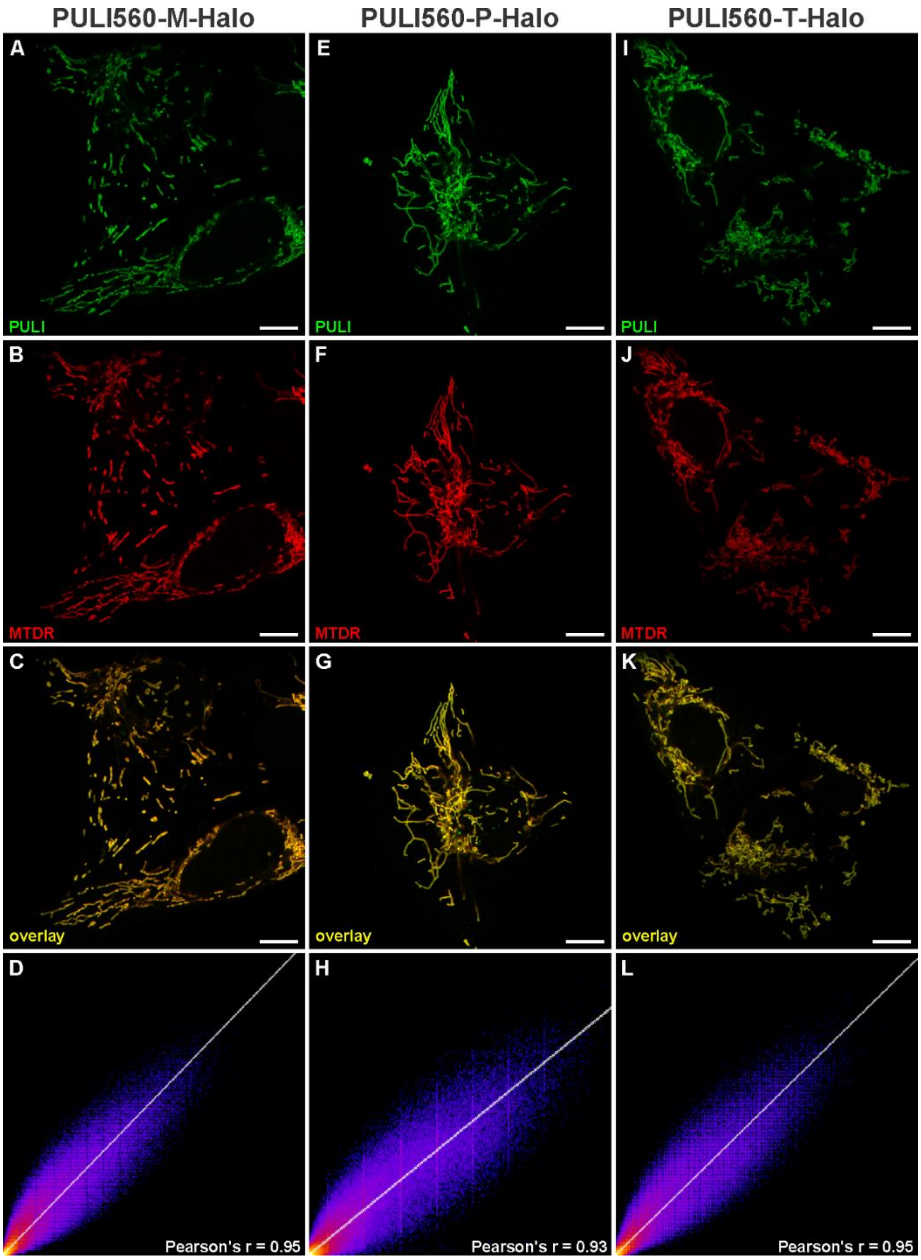

Figure S7. Confocal images of live U2OS cells stably expressing a Tomm20-HaloTag construct, stained with **PULI$_{560}$-M-Halo** (A-D), **PULI$_{560}$-P-Halo** (E-H), or **PULI$_{560}$-T-Halo** (I-L) for 60 minutes, washed (30 min) and co-stained with mitochondria marker MTDR (MitoTracker Deep Red, ThermoFischer). Samples were imaged in cell medium after photoactivation of the PULI compound. From top to bottom, the PULI channel (A, E, I), the MTDR channel (B, F, J), the overlay (C, G, K), and the 2D pixel Intensity Histogram (D, H, L) are displayed. The Pearson's correlation coefficient obtained (calculated with Coloc 2 plugin on ImageJ) in each case is also indicated. Scalebars: 10 µm.

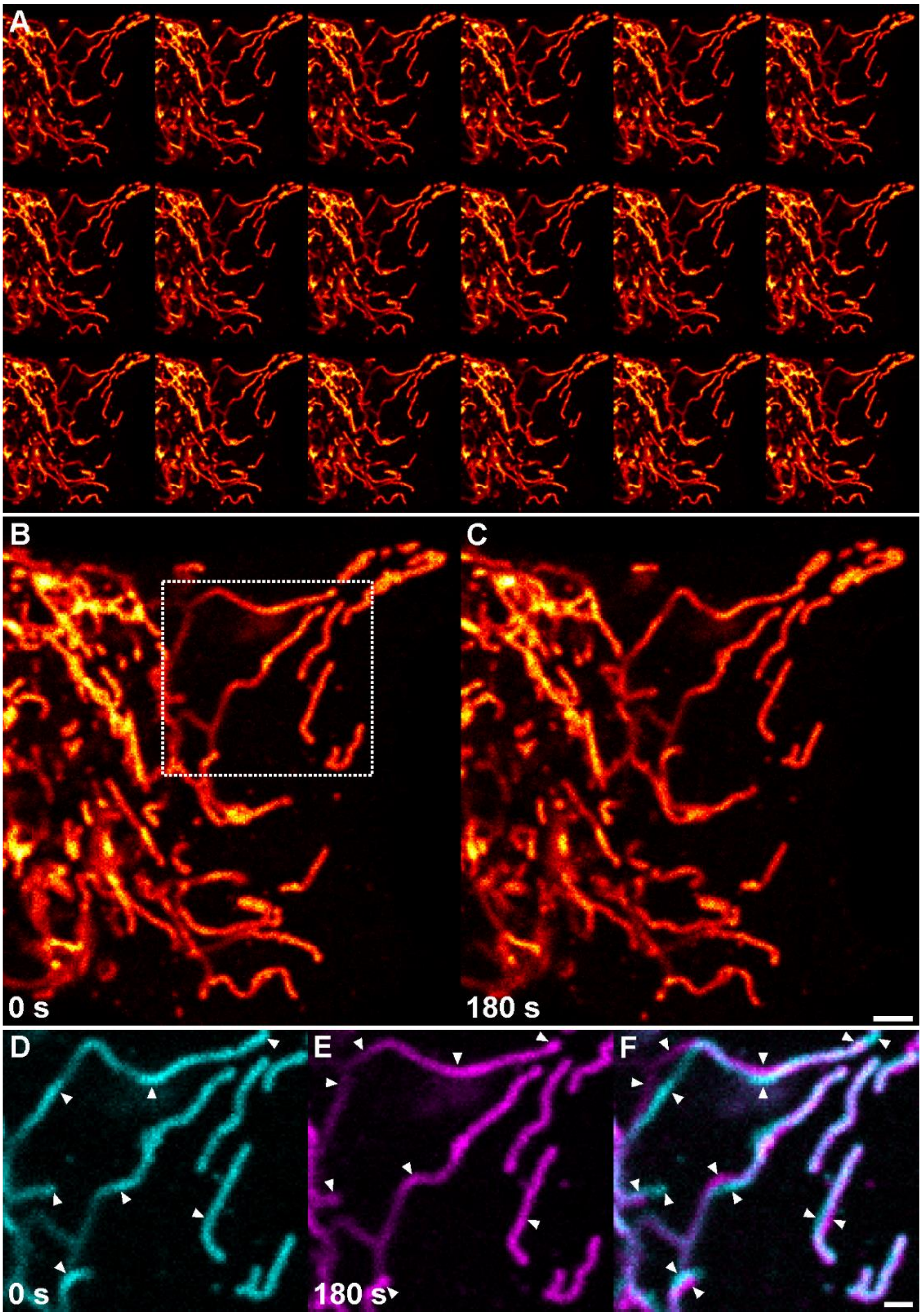

Figure S8. Confocal images of live U2OS cells stably expressing a Tomm20-HaloTag construct, stained with **PULI$_{560}$-T-Halo**. Images were acquired after activation. (A) A 3 minutes time-series of frames recorded every 10 s. The first and the last frames are presented in (B-C), respectively. (D-E) A zoomed ROI color-coded by time, and their overlap (F). A set of arrows, manually selected, show structures with large displacement. Scalebars: 2 µm (B-C) and 1 µm (D-F).

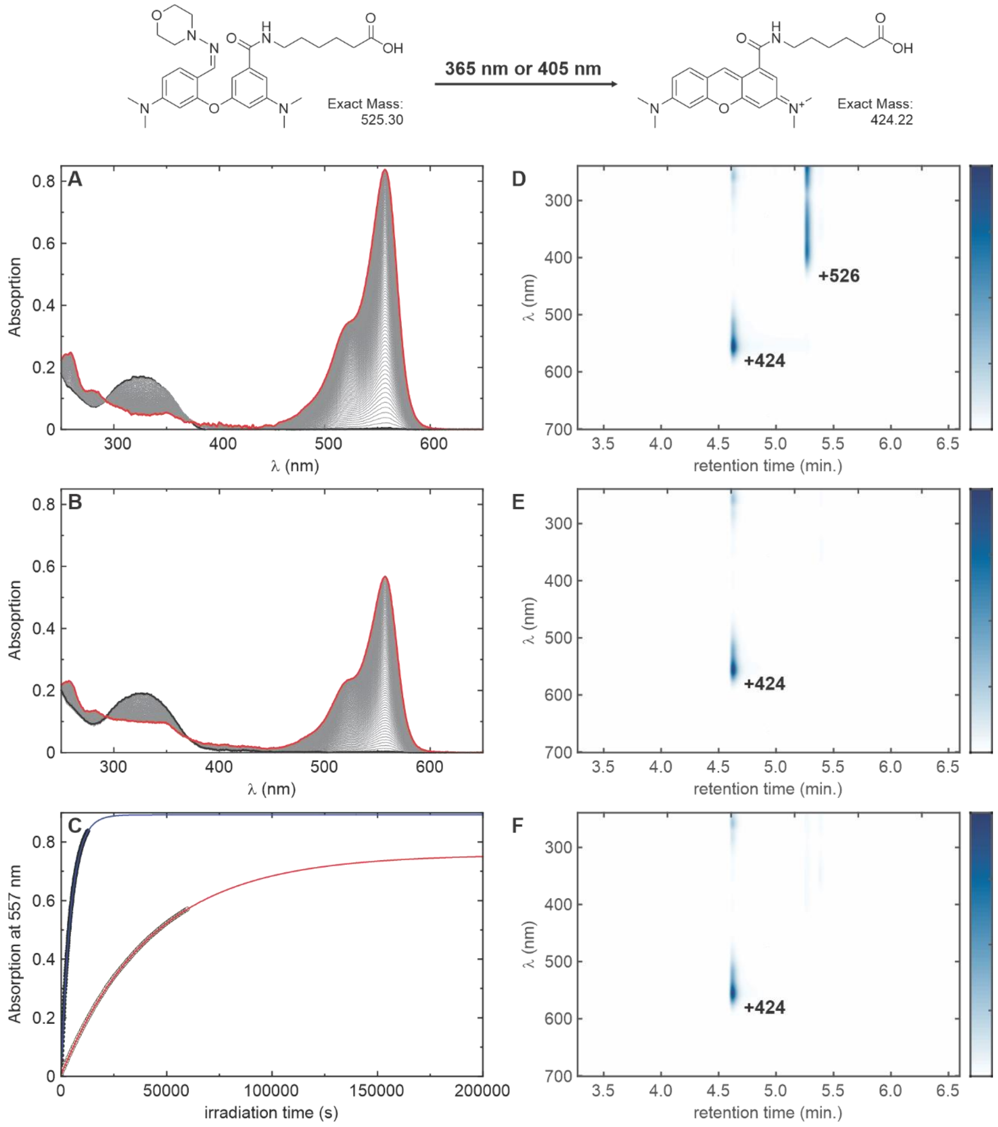

Figure S9. Photoactivation of **PULI$_{560}$-M (37)** in buffer under irradiation with 365 nm (A) or 405 nm (B), and compared transients at 577 nm, the absorption maximum of the pyronin product (C). LCMS (2D absorption maps) of the starting compound (D) and the irradiated mixtures at 365 nm and 405 nm (E and F, respectively).

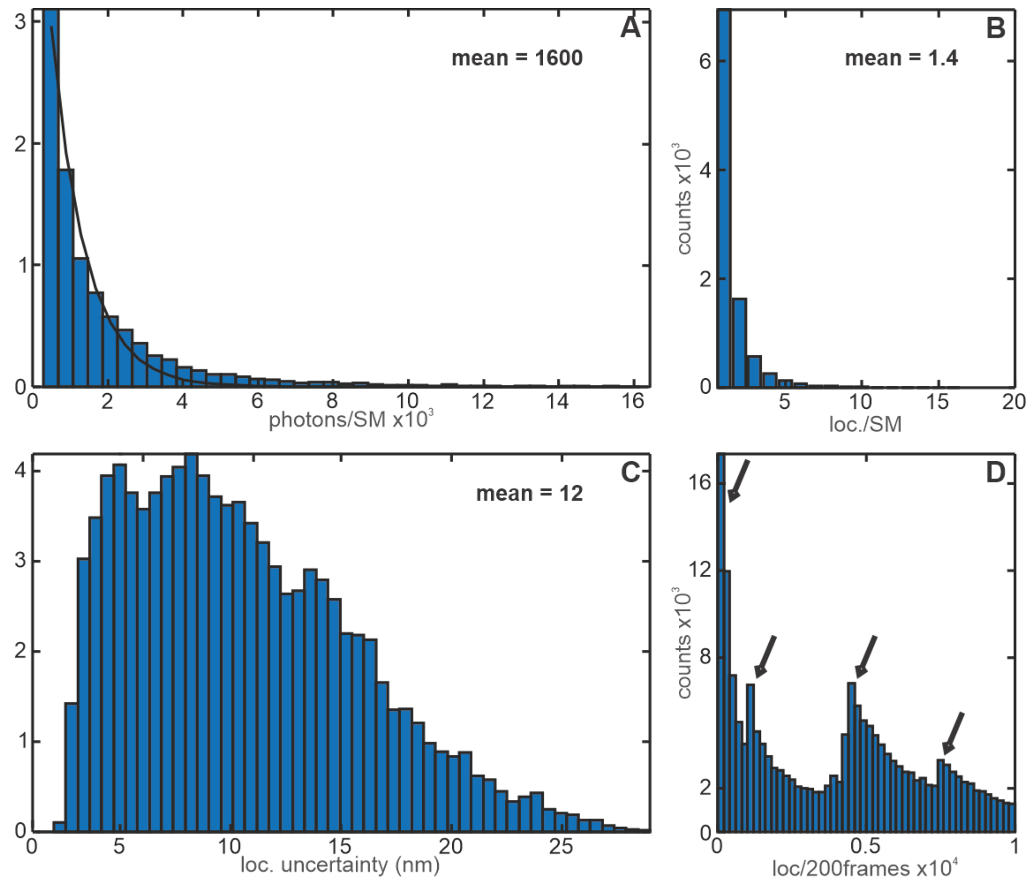

Figure S10. Histograms of the photons per SM (A), localizations per molecule after merging the data (B), localization uncertainty (C) and SM localizations over time (D) from the PALM image in Figure 4 right. Peaks in (D) correspond to an increase of the activation (405 nm) laser.

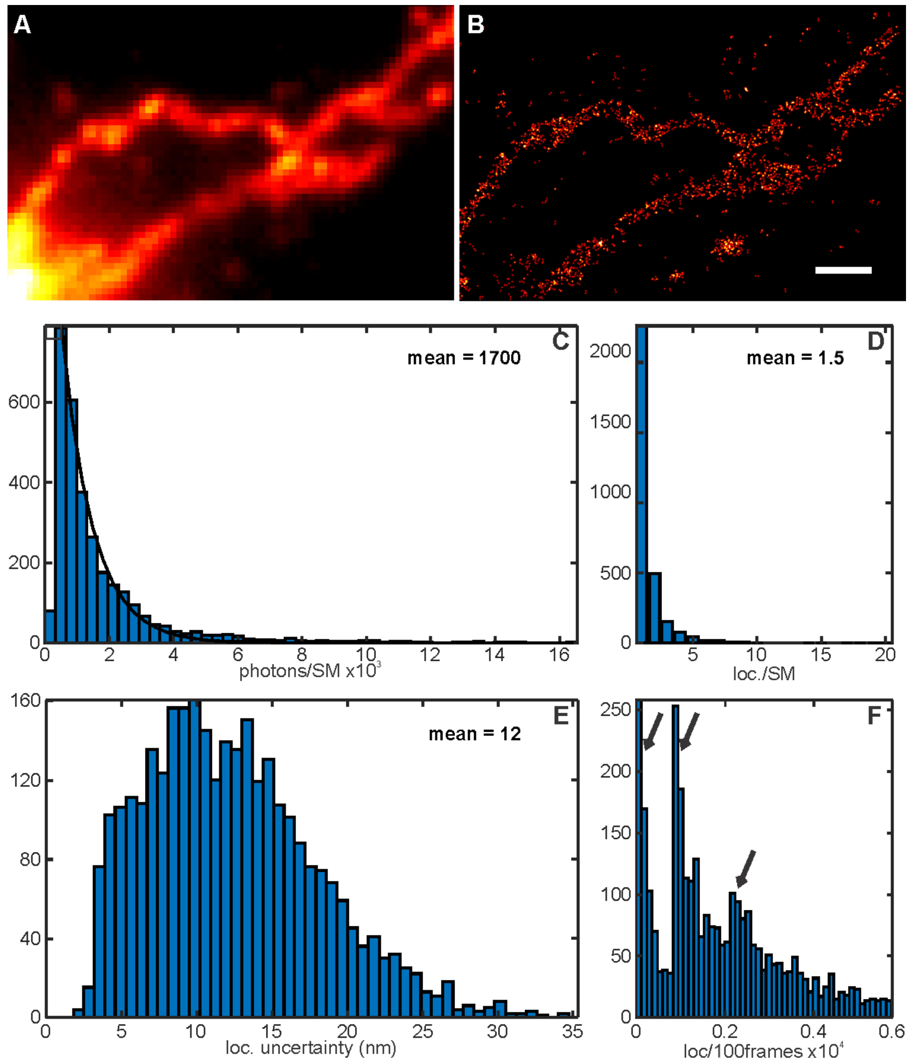

Figure S11. (A) Wide-field and (B) super-resolution PALM image of U2OS cells stably expressing a Tomm20-HaloTag construct. Cells were labelled live with **PULI$_{560}$-M-Halo**, fixed and imaged in PBS at pH 8.5 in a WF microscope. Images were acquired in a similar way as the ones displayed in Figure 4. Histograms of the photons per SM (C), localizations per molecule after merging the data (D), localization uncertainty (E) and SM localizations over time (F). Peaks in (F) correspond to an increase of the activation (405 nm) laser. Scalebar: 1 µm

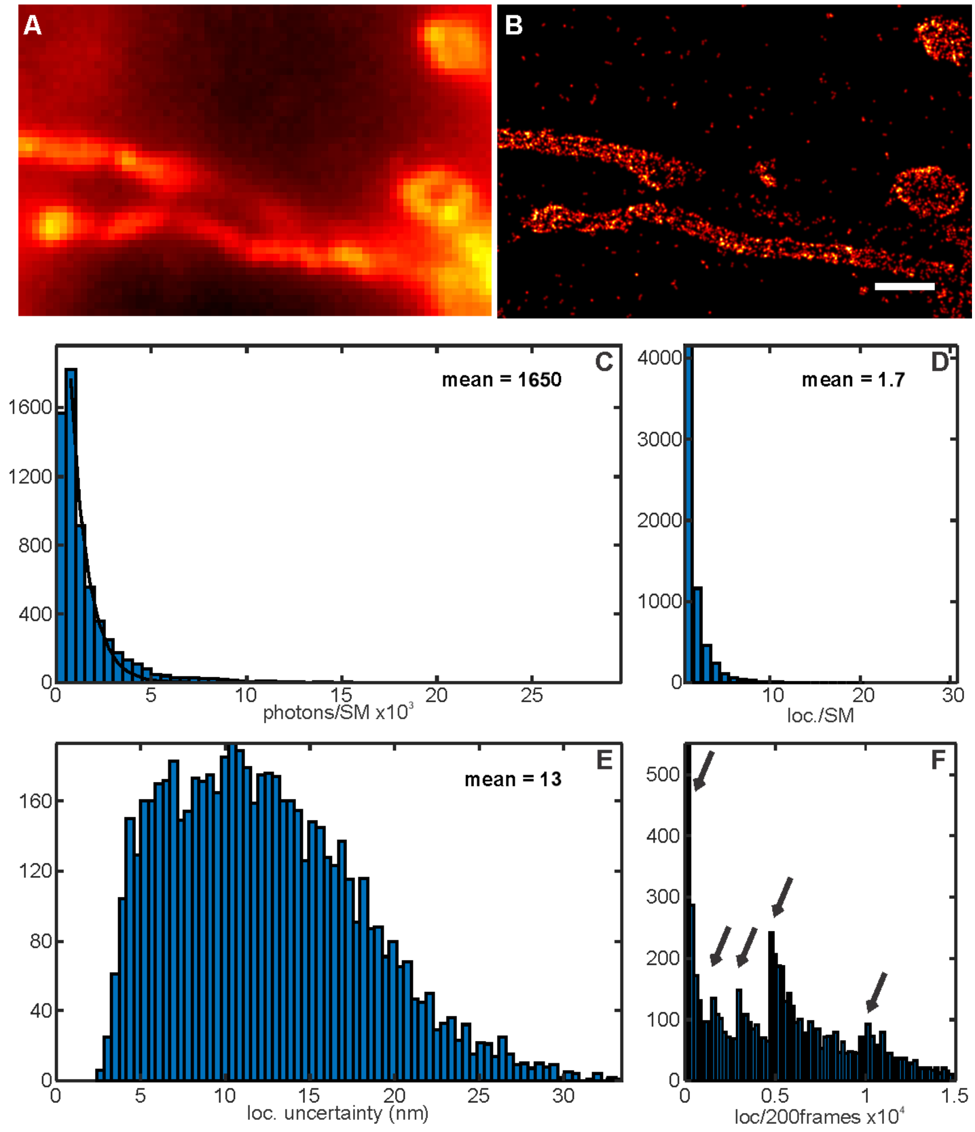

Figure S12. (A) Wide-field and (B) super-resolution PALM image of U2OS cells stably expressing a Tomm20-HaloTag construct. Cells were labelled live with **PULI$_{560}$-P-Halo**, fixed and imaged in PBS at pH 8.5 in a WF microscope. Images were acquired in a similar way as the ones displayed in Figure 4. Histograms of the photons per SM (C), localizations per molecule after merging the data (D), localization uncertainty (E) and SM localizations over time (F). Peaks in (F) correspond to an increase of the activation (405 nm) laser. Scalebar: 1 µm

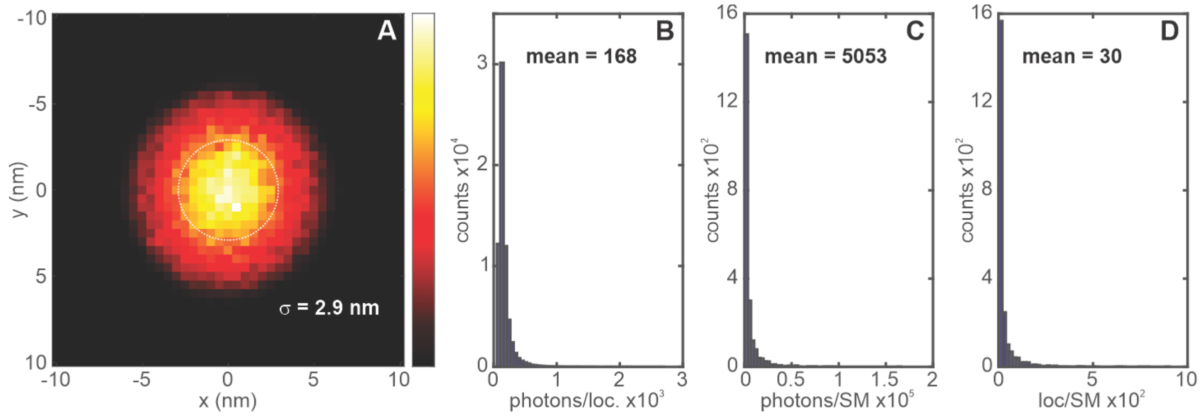

Figure S13. (A) 2D dispersion of localizations around the mean position, fitted to a 2D gaussian function (the obtained sigma value is indicated), and histogram of the photons per localizations (B), photons per single molecule event (C), and the number of localizations per single molecule (D), from the MINFLUX image in Figure 5 right. The mean values of the histograms are indicated on each case.

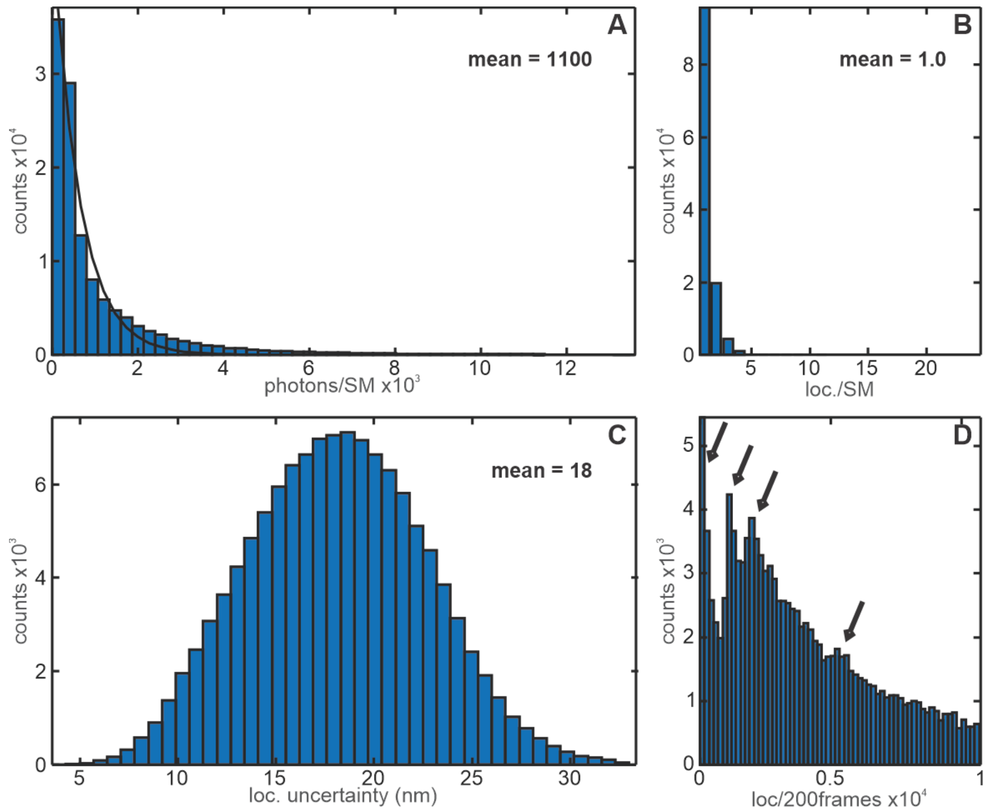

Figure S14. Histograms of the photons per SM (A), localizations per molecule after merging the data (B), localization uncertainty (C) and SM localizations over time (D) from the PALM image in Figure 6B. Peaks in (D) correspond to an increase of the activation (375 nm) laser.

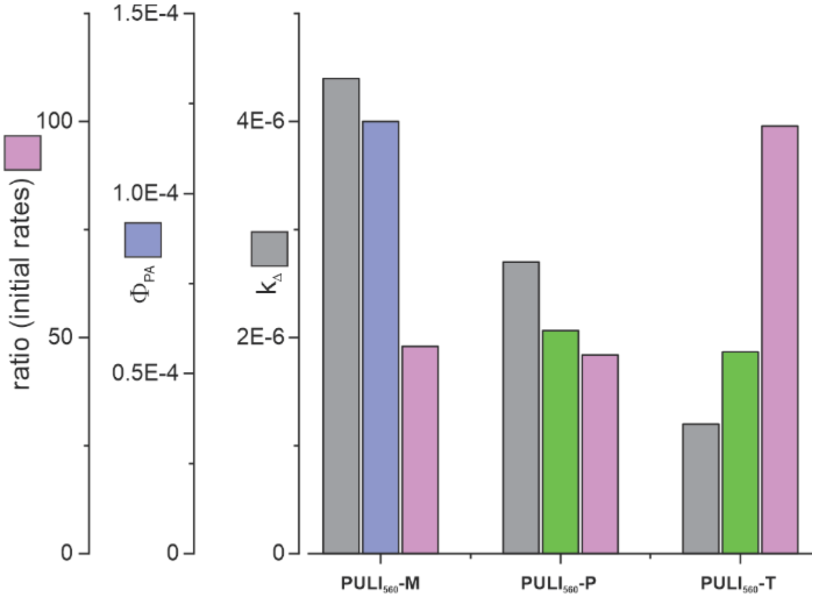

Figure S15. Initial rate constants for the thermal reaction k_Δ (gray bars), photoactivation quantum yields Φ_{PA} (blue bars) and the ratio of the initial rates of photoinduced and thermal reactions (purple bars) for the three $PULI_{560}$ dyes. The initial rate (slope of the linear fits) of the photoinduced reaction is proportional to the photoactivation quantum yield (Φ_{PA}), the absorption coefficient of the dye at the irradiation wavelength ($\varepsilon_{\lambda IRR}$), the intensity of the photolyzing light, and the photokinetic factor ($1\text{-}10^{-A}$), where A is the absorption of the solution at the irradiation wavelength.

Supplementary Tables

Table S1. extended table of the photophysical properties of the tested compounds. Samples were irradiated with 365 nm light source, or thermally equilibrated after a small amount of stock solution in DMSO was added to a buffered aqueous solution (10 µM compound in 100 mM phosphate, pH = 7).

<table>
<tr><th>Compound</th><th>k_{Δ} (s^{-1})</th><th>λ_{Abs}^{max} (nm)</th><th>λ_{Abs}^{max} Pyr</th><th>λ_{Em}^{max} Pyr</th><th>Φ_{PA}</th><th>Φ_{Fluo} Pyronin</th><th>τ_{Fluo} (s) Pyronin</th><th>Notes on irradiation</th></tr>
<tr><td>1</td><td>4.3E-5</td><td>353</td><td>546</td><td>565</td><td>1.3E-5</td><td>0.42</td><td>1.83</td><td></td></tr>
<tr><td>2</td><td>-</td><td>361</td><td>635</td><td>650</td><td>n.d.</td><td>0.55</td><td>2.60</td><td>Very slow photoconversion</td></tr>
<tr><td>3</td><td>5.6E-6</td><td>341</td><td>538</td><td>557</td><td>5.8E-6</td><td>n.d.</td><td>n.d.</td><td>Product photobleaching</td></tr>
<tr><td>4</td><td>1.6E-5</td><td>317</td><td rowspan="7">546</td><td rowspan="7">565</td><td>7.5E-4</td><td rowspan="7">0.42</td><td rowspan="7">1.83</td><td></td></tr>
<tr><td>5</td><td>-</td><td>n.d.</td><td>-</td><td>drop of absorption, no conversion</td></tr>
<tr><td>6</td><td>1.5E-5</td><td>324</td><td>3.5E-4</td><td>drop of absorption, no conversion</td></tr>
<tr><td>7</td><td>-</td><td>n.d.</td><td>-</td><td>Insoluble, drop of absorption, no conversion</td></tr>
<tr><td>8</td><td>1.7E-5</td><td>326</td><td>8.4E-4</td><td></td></tr>
<tr><td>9</td><td>1.5E-5</td><td>327</td><td>8.0E-4</td><td></td></tr>
<tr><td>10</td><td>0.6E-5</td><td>322</td><td>1.1E-3</td><td></td></tr>
<tr><td>11</td><td>1.8E-4</td><td>292</td><td rowspan="4">538</td><td rowspan="4">557</td><td>n.d.</td><td rowspan="4">n.d.</td><td rowspan="4">n.d.</td><td>Non monoexponential behaviour</td></tr>
<tr><td>12</td><td>1.7E-4</td><td>293</td><td>n.d.</td><td>Non monoexponential behaviour</td></tr>
<tr><td>13</td><td>2.2E-4</td><td>292</td><td>n.d.</td><td>Non monoexponential behaviour</td></tr>
<tr><td>14</td><td>1.4E-4</td><td>293</td><td>n.d.</td><td>Non monoexponential behaviour</td></tr>
<tr><td>15</td><td>-</td><td>326</td><td rowspan="12">635</td><td rowspan="12">650</td><td>n.d.</td><td rowspan="12">0.55</td><td rowspan="12">2.60</td><td>Fast bleaching, low photoconversion</td></tr>
<tr><td>16</td><td>-</td><td>~350</td><td>-</td><td>Insoluble, drop of absorption, no photoconversion</td></tr>
<tr><td>17</td><td>-</td><td>~320</td><td>-</td><td>Insoluble, drop of absorption, no photoconversion</td></tr>
<tr><td>18</td><td>-</td><td>~350</td><td>-</td><td>Rapid drop of absorption, then slow thermal conv.</td></tr>
<tr><td>19</td><td>-</td><td>~370</td><td>-</td><td>drop of absorption, no conversion, decompose</td></tr>
<tr><td>20</td><td>-</td><td>~350</td><td>-</td><td>drop of absorption, no conversion, decompose</td></tr>
<tr><td>21</td><td>-</td><td>~330</td><td>-</td><td>drop of absorption, no conversion, decompose</td></tr>
<tr><td>22</td><td>-</td><td>~320</td><td>-</td><td>drop of absorption, no conversion, decompose</td></tr>
<tr><td>23</td><td>-</td><td>~380</td><td>-</td><td>Rapid drop of absorption, no conversion</td></tr>
<tr><td>24</td><td>-</td><td>~390</td><td>-</td><td>drop of absorption, no conversion, decompose</td></tr>
<tr><td>25</td><td>-</td><td>322</td><td>8.1E-5</td><td>Low conversion</td></tr>
<tr><td>26</td><td>-</td><td>330</td><td>4.6E-5</td><td>Low conversion</td></tr>
<tr><td>27</td><td>n.d.</td><td>n.d.</td><td>552</td><td>568</td><td>n.d.</td><td>0.33</td><td>1.57</td><td></td></tr>
<tr><td>28</td><td>n.d.</td><td>355</td><td>n.d.</td><td>n.d.</td><td>n.d.</td><td></td><td></td><td>Biexp. thermal decay/Product photobleaching</td></tr>
<tr><td>29</td><td>n.d.</td><td>353</td><td>n.d.</td><td>n.d.</td><td>n.d.</td><td></td><td></td><td>Biexp. thermal decay/bleaching</td></tr>
<tr><td>30</td><td>6.8E-6</td><td>353</td><td>557</td><td>574</td><td></td><td>0.33</td><td>1.46</td><td></td></tr>
<tr><td>31</td><td>9.5E-6</td><td>324</td><td rowspan="3">552</td><td rowspan="3">568</td><td>5.7E-4</td><td rowspan="3">0.33</td><td rowspan="3">1.57</td><td></td></tr>
<tr><td>32</td><td>8.1E-6</td><td>325</td><td>4.2E-4</td><td></td></tr>
<tr><td>SI-6</td><td>7.9E-6</td><td>~310</td><td></td><td></td></tr>
<tr><td>33</td><td>n.d.</td><td>328</td><td rowspan="2">n.d.</td><td rowspan="2">n.d.</td><td>n.d.</td><td rowspan="2">0.16</td><td rowspan="2">0.82</td><td>Thermal reaction shows an upward curvature, fast photobleaching of product</td></tr>
<tr><td>34</td><td>n.d.</td><td>332</td><td>n.d.</td><td>Thermal reaction shows an upward curvature, fast photobleaching of product</td></tr>
<tr><td>35</td><td>n.d.</td><td>322</td><td rowspan="2">n.d.</td><td rowspan="2">n.d.</td><td></td><td rowspan="2">0.20*</td><td rowspan="2">1.02</td><td>Not monoexponential conversion, *CF3 converts to COOH in photoproduct</td></tr>
<tr><td>36</td><td>n.d.</td><td>321</td><td></td><td>Not monoexponential conversion, *CF3 converts to COOH in photoproduct</td></tr>
<tr><td>37</td><td>4.4E-6</td><td>326</td><td rowspan="3">557</td><td rowspan="3">574</td><td>1.2E-4</td><td rowspan="3">0.33</td><td rowspan="3">1.46</td><td></td></tr>
<tr><td>38</td><td>2.7E-6</td><td>328</td><td>6.2E-5</td><td></td></tr>
<tr><td>39</td><td>1.2E-6</td><td>327</td><td>5.6E-5</td><td></td></tr>
<tr><td>40</td><td>n.d.</td><td>n.d.</td><td>n.d.</td><td>n.d.</td><td>n.d.</td><td>0.75</td><td>3.06</td><td></td></tr>
<tr><td>41</td><td>2.3E-5</td><td>365</td><td>550</td><td>568</td><td>1.9E-6</td><td>0.76</td><td>3.06</td><td></td></tr>
<tr><td>42</td><td>1.7E-5</td><td>327</td><td rowspan="2">544</td><td rowspan="2">564</td><td>3.2E-4</td><td rowspan="2">0.75</td><td rowspan="2">3.15</td><td></td></tr>
<tr><td>43</td><td>1.5E-5</td><td>330</td><td>3.4E-4</td><td></td></tr>
<tr><td>44</td><td>1.5E-5</td><td>345</td><td rowspan="2">550</td><td rowspan="2">568</td><td>4.3E-4</td><td rowspan="2">0.76</td><td rowspan="2">3.06</td><td></td></tr>
<tr><td>45</td><td>1.4E-5</td><td>345</td><td>3.4E-4</td><td></td></tr>
<tr><td>52</td><td><2E-7</td><td>326</td><td rowspan="2">639</td><td rowspan="2">654</td><td>4.8E-5</td><td rowspan="2">n.d.</td><td rowspan="2">n.d.</td><td>Higher conversion than 25 or 26</td></tr>
<tr><td>53</td><td>2.5E-7</td><td>328</td><td>3.5E-5</td><td>Higher conversion than 25 or 26</td></tr>
<tr><td>SI-2</td><td>n.d.</td><td>290</td><td>n.d.</td><td>n.d.</td><td>n.d.</td><td>n.d.</td><td>n.d.</td><td>The main product is the imine</td></tr>
<tr><td>SI-4</td><td>n.d.</td><td>287</td><td>n.d.</td><td>n.d.</td><td>n.d.</td><td>n.d.</td><td>n.d.</td><td>Complex photoreaction, no clean product</td></tr>
<tr><td>SI-20</td><td>-</td><td>322</td><td>n.d.</td><td>n.d.</td><td>n.d.</td><td>n.d.</td><td>n.d.</td><td>No activation</td></tr>
<tr><td>SI-21</td><td>-</td><td>326</td><td>n.d.</td><td>n.d.</td><td>n.d.</td><td>n.d.</td><td>n.d.</td><td>No activation</td></tr>
<tr><td>SI-27</td><td>-</td><td>~340</td><td>n.d.</td><td>n.d.</td><td>n.d.</td><td>n.d.</td><td>n.d.</td><td>drop of absorption, decompose</td></tr>
<tr><td>SI-28</td><td>-</td><td>362</td><td>n.d.</td><td>n.d.</td><td>n.d.</td><td>n.d.</td><td>n.d.</td><td>low conversion, high decomposition</td></tr>
</table>

Table S2. Photoactivation quantum yields of dyes used in super-resolution microscopy.

	Compound	Φ_{PA}	λ^{irrad}	Solvent	Reference
PA-SiR	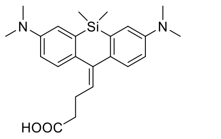	9.0×10^{-4}	405 nm	PBS	Frei et al., **2019**[81]
Rh-NN[a]	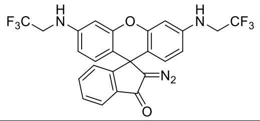	9.7×10^{-3}	405 nm	MeOH	Belov et al., **2014**[82]
	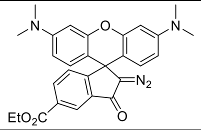	8.5×10^{-3}	405 nm	MeOH	Belov et al., **2014**[82]
	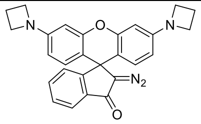	2.3×10^{-3}	365 nm	HEPES pH = 7.3	Grim et al., **2016**[83]
	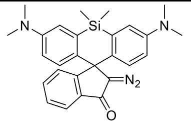	5.1×10^{-3}	405 nm	MeOH	Eördögh et al., **2020**[84]
Thioketones	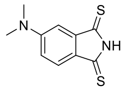	3.0×10^{-2}	365 nm	DMSO	Tang et al., **2019**[85]
	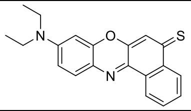	2.6×10^{-2}	470 nm	DMSO	Tang et al., **2019**[85]
Azido Push–Pull	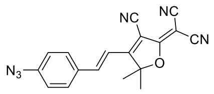	5.9×10^{-3}	407 nm	EtOH	Lord et al., **2010**[86]
o-NV[b]	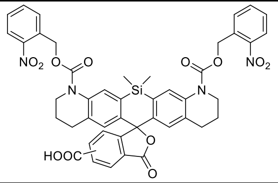	5.2×10^{-2} 3.3×10^{-2}	365 nm	MeCN/ Buffer (1:1)	Aktalay et al., **2023**[87]
	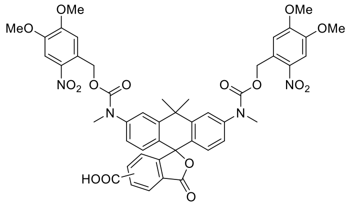	9.5×10^{-3} 7.7×10^{-3}	365 nm	MeCN/ Buffer (1:1)	Aktalay et al., **2023**[87]
	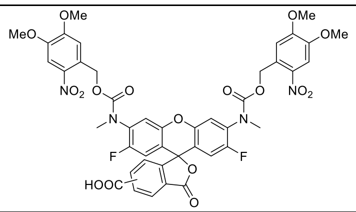	4.9×10^{-3} 4.6×10^{-3}	365 nm	MeCN/ Buffer (1:1)	Aktalay et al., **2023**[87]

[a] Φ_{PA} for the production of the fluorescent product; [b] two Φ_{PA} values informed for the consecutive reactions.

Table S3. Summary of relevant parameters calculated for superresolution images acquired with PULI dyes. N_{ph}: mean photons detected per single-molecule event after data merging; Δxy: mean localization uncertainty; loc./SM: mean localizations (in frames) per single-molecule event after merging, or mean number of MINFLUX localizations per single-molecule event.

Figure	Compound	N_{ph} (photons/SM)	Dxy (nm)	loc./SM (frames)
Fig. 4	**PULI$_{560}$-T-Halo**	1600	12	1.4
Fig. S11B	**PULI$_{560}$-M-Halo**	1700	12	1.5
Fig. S12B	**PULI$_{560}$-P-Halo**	1650	13	1.7
Fig. 6B	**PULI$_{640}$-M-NHS**	1100	18	1.0
Fig. 5	**PULI$_{560}$-T-Halo**	5053	2.9	30 (MINFLUX locs.)

General synthesis of O-bridged molecules

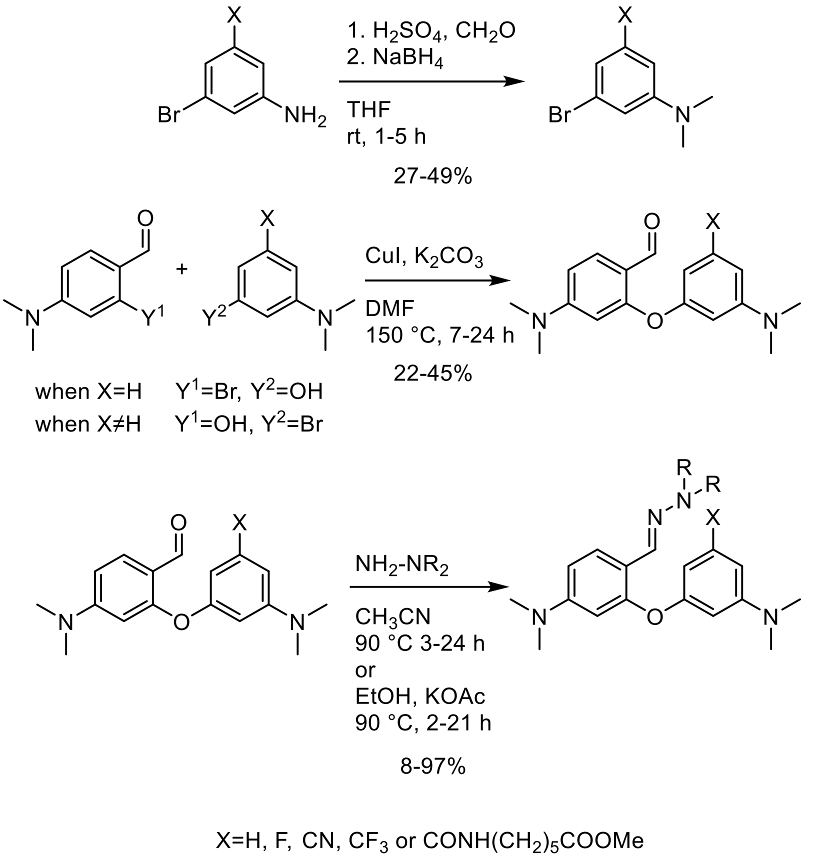

Scheme S1: General synthesis of compounds **4-10** and **27-39**

Synthesis and characterization of O-bridged compounds

Compound **SI-1**

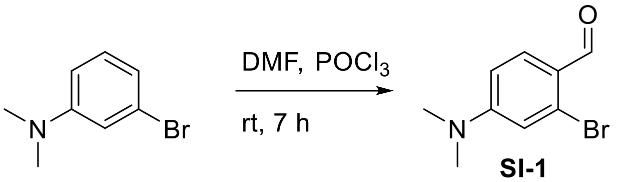

Dimethylformamide (8 ml) and $POCl_3$ (4 mL) was mixed slowly at room temperature, while the mixture warmed up by itself. The mixture was let to cool back to room temperature (15 minutes), then 3-Bromoaniline (1 g, 0,704 mL) was added dropwise. The solution was stirred for 7 hours, then poured on water and waited until a white precipitate formed. The precipitate was filtered, washed with water and dried to yield 0.77 g (68 %) of clean product.

^{1}H NMR (400 MHz, $CDCl_3$) δ 10.09 (s, 1H), 7.80 (d, *J* = 8.8 Hz, 1H), 6.80 (d, *J* = 2.5 Hz, 1H), 6.64 (dd, *J* = 8.9, 2.5 Hz, 1H), 3.08 (s, 6H).

^{13}C NMR (101 MHz, $CDCl_3$) δ 190.44, 154.68, 131.24, 129.89, 122.23, 114.99, 110.73, 40.24.

Compound **1**

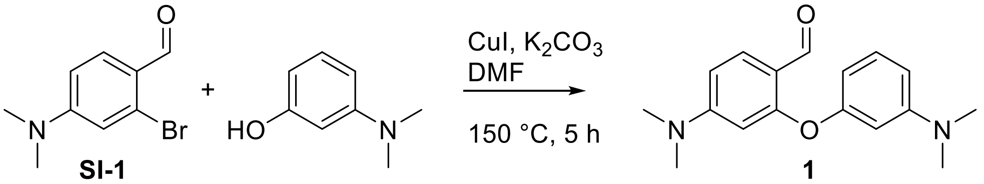

Compound **SI-1** (0.5 g, 1 eq), 3-Dimethylaminophenol (0.6 g, 2 eq), CuI (0.209 g, 0.5 eq) and K_2CO_3 (0.605 g, 2 eq) was placed in a flame dried flask filled with Argon. The components were dissolved in 10 mL of dry Dimethylformamide and stirred under Argon for 3 hours at 150 °C. After 3 hours, another 0.209 g of CuI (0.5 eq) was added to the mixture and it was stirred again at 150 °C for another 2 hours. After altogether 5 hours, according to LC-MS, most starting materials were consumed, so the mixture was poured on water, filtered through Cellite, washed with water and ethyl acetate to clear it from copper salts. The supernatant was diluted with ethyl acetate (to 30 mL) and washed with water (3*30 mL), the organic phase dried over Na_2SO_4 and evaporated. The product was purified with flash column chromatography (hexane:ethyl acetate 0 to 20% in 10 CV) to give 190 mg (31 %) of yellow oil, which later solidified and became slightly red in the freezer.

^{1}H NMR (400 MHz, CD_3CN) δ 10.08 (d, *J* = 0.8 Hz, 1H), 7.70 (d, *J* = 8.9 Hz, 1H), 7.17 (t, *J* = 8.2 Hz, 1H), 6.54 (ddd, *J* = 4.3, 2.5, 0.8 Hz, 1H), 6.52 (ddd, *J* = 3.5, 2.5, 0.7 Hz, 1H), 6.45 (t, *J* = 2.4 Hz, 1H), 6.29 (ddd, *J* = 8.0, 2.3, 0.8 Hz, 1H), 6.12 (d, *J* = 2.4 Hz, 1H), 2.94 (s, 6H), 2.90 (s, 6H).

^{13}C NMR (101 MHz, CD_3CN) δ 187.16, 162.52, 159.28, 156.87, 153.41, 131.02, 130.41, 117.09, 108.88, 108.32, 106.95, 103.71, 101.33, 40.62, 40.25.

HRMS (ESI-QTOF) m/z: $[M + H]^+$ Calcd for $C_{17}H_{20}N_2O_2$: 285.1598; Found 285.1598

Compound **4**

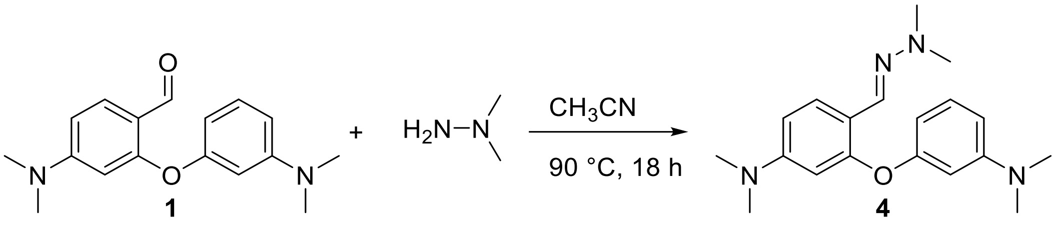

Compound **1** (20 mg, 1 eq) and N, N-dimethylhydrazine (80 mg, 0.1 mL, 19 eq) was dissolved in 1 mL of acetonitrile in a sealed screw cap vial and the solution was heated at 90 °C overnight. Next day the solution was poured on water, and after 1 hour the formed off white crystals were filtered, dissolved in ethyl acetate and passed through a small plug of silica. The solvent was evaporated to give 20 mg (87 %) of reddish oil as product.

^{1}H NMR (400 MHz, CD_3CN) δ 7.69 (d, *J* = 8.8 Hz, 1H), 7.38 – 7.36 (m, 1H), 7.09 (t, *J* = 8.2 Hz, 1H), 6.57 (ddd, *J* = 8.9, 2.6, 0.6 Hz, 1H), 6.43 (ddd, *J* = 8.4, 2.5, 0.8 Hz, 1H), 6.36 (t, *J* = 2.4 Hz, 1H), 6.27 (d, *J* = 2.6 Hz, 1H), 6.15 (ddd, *J* = 8.1, 2.3, 0.8 Hz, 1H), 2.87 (s, 6H), 2.87 (s, 6H), 2.74 (s, 6H).

^{13}C NMR (101 MHz, CD_3CN) δ 160.55, 155.41, 153.32, 152.41, 130.79, 130.03, 126.78, 117.91, 109.99, 107.86, 105.72, 104.65, 102.49, 43.23, 40.65, 40.51.

HRMS (ESI-QTOF) *m/z*: $[M + H]^+$ Calcd for $C_{19}H_{26}N_4O$: 327.2179; Found 327.2171

Compound **SI-2**

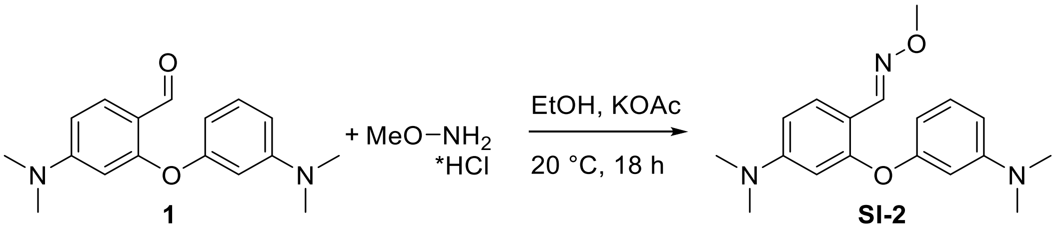

Compound **1** (20 mg, 1 eq), Methoxyamine hydrochloride (50 mg, 8.5 eq) and potassium acetate (100 mg, 14.5 eq) was dissolved in 1 mL of ethanol in a sealed screw cap vial and stirred overnight. Next day the solution was poured on water, and after 1 hour the formed pale pink powder was filtered, dissolved in ethyl acetate and passed through a small plug of silica. The solvent was evaporated to give 21 mg (95 %) of brownish oil as product.

^{1}H NMR (400 MHz, CD_3CN) δ 8.11 (s, 1H), 7.66 (d, *J* = 8.9 Hz, 1H), 7.11 (t, *J* = 8.2 Hz, 1H), 6.57 (ddd, *J* = 8.8, 2.6, 0.6 Hz, 1H), 6.46 (ddd, *J* = 8.3, 2.5, 0.8 Hz, 1H), 6.35 (t, *J* = 2.4 Hz, 1H), 6.25 (d, *J* = 2.6 Hz, 1H), 6.13 (ddd, *J* = 8.0, 2.3, 0.8 Hz, 1H), 3.81 (s, 3H), 2.90 (s, 6H), 2.88 (s, 6H).

^{13}C NMR (101 MHz, CD_3CN) δ 160.11, 156.75, 154.04, 153.35, 144.85, 130.90, 127.85, 112.11, 109.57, 108.17, 105.66, 103.85, 102.52, 61.93, 40.61, 40.31.

HRMS (ESI-QTOF) m/z: $[M + H]^+$ Calcd for $C_{18}H_{23}N_3O_2$: 314.1863; Found 314.1861

Compound **5**

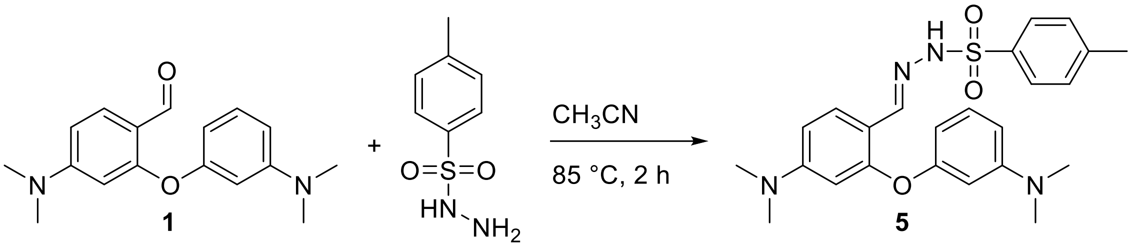

Compound **1** (20 mg, 1 eq) and p-Toluenesulfonyl hydrazide (131 mg, 10 eq) was dissolved in 1 mL of acetonitrile in a sealed screw cap vial and stirred at 85°C for 2 hours. Then the solution was diluted with ethyl acetate (10 mL) and washed with water (3*10 mL), the organic layer dried over Na_2SO_4 and evaporated. The product was purified with flash column chromatography (hexane:ethyl acetate 0 to 30% in 10 CV) to give 20 mg (63 %) of product.

^{1}H NMR (400 MHz, CD_3CN) δ 8.71 (s, 1H), 7.98 (s, 1H), 7.81 – 7.72 (m, 2H), 7.62 (d, *J* = 8.9 Hz, 1H), 7.41 – 7.33 (m, 2H), 7.10 (t, *J* = 8.2 Hz, 1H), 6.53 (dd, *J* = 8.9, 2.5 Hz, 1H), 6.46 (ddd, *J* = 8.4, 2.5, 0.8 Hz, 1H), 6.31 (t, *J* = 2.4 Hz, 1H), 6.16 (d, *J* = 2.6 Hz, 1H), 6.12 (ddd, *J* = 8.1, 2.3, 0.8 Hz, 1H), 2.88 (s, 6H), 2.86 (s, 6H), 2.40 (s, 3H).

^{13}C NMR (101 MHz, CD_3CN) δ 159.84, 157.56, 154.34, 153.31, 145.33, 145.13, 136.86, 130.91, 130.50, 128.63, 127.72, 113.53, 109.46, 108.31, 105.98, 103.18, 102.73, 40.58, 40.25, 21.51.

HRMS (ESI-QTOF) m/z: $[M + H]^+$ Calcd for $C_{24}H_{28}N_4O_3S$: 453.1952; Found 453.1955

Compound **6**

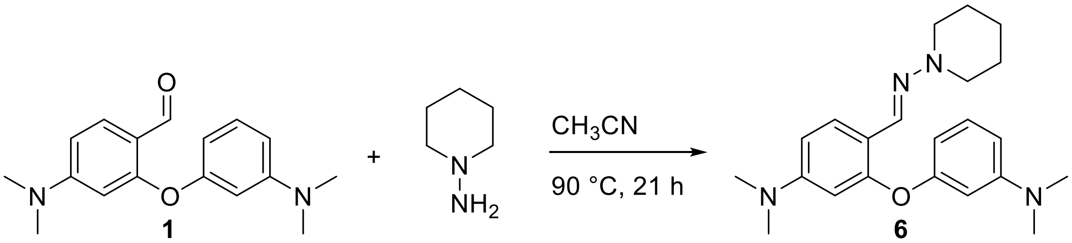

Compound **1** (20 mg, 1 eq) and N-Aminopiperidine (70 mg, 75 µL, 10 eq) was dissolved in 1 mL of acetonitrile in a sealed screw cap vial and stirred at 90°C for 21 hours. Then the solution was diluted with ethyl acetate (10 mL) and washed with water (3*10 mL), the organic layer dried over Na_2SO_4 and evaporated. The product was purified with flash column chromatography (hexane:ethyl acetate 0 to 20% in 10 CV) to give 19 mg (73 %) of product.

^{1}H NMR (400 MHz, CD_3CN) δ 7.71 (d, *J* = 8.9 Hz, 1H), 7.64 (s, 1H), 7.09 (t, *J* = 8.2 Hz, 1H), 6.58 (dd, *J* = 9.0, 2.5 Hz, 1H), 6.44 (ddd, *J* = 8.3, 2.5, 0.9 Hz, 1H), 6.35 (t, *J* = 2.4 Hz, 1H), 6.27 (d, *J* = 2.6 Hz, 1H), 6.14 (ddd, *J* = 8.1, 2.3, 0.8 Hz, 1H), 2.93 (t, *J* = 5.6 Hz, 4H), 2.88 (s, 6H), 2.87 (s, 6H), 1.62 (p, *J* = 5.8 Hz, 4H), 1.50 – 1.41 (m, 2H).

^{13}C NMR (101 MHz, CD_3CN) δ 160.56, 155.72, 153.33, 152.69, 131.37, 130.80, 126.96, 117.71, 109.94, 107.88, 105.66, 104.58, 102.45, 53.05, 40.65, 40.48, 25.90, 24.87.

HRMS (ESI-QTOF) m/z: $[M + H]^+$ Calcd for $C_{22}H_{30}N_4O$: 367.2492; Found 367.2486

Compound **7**

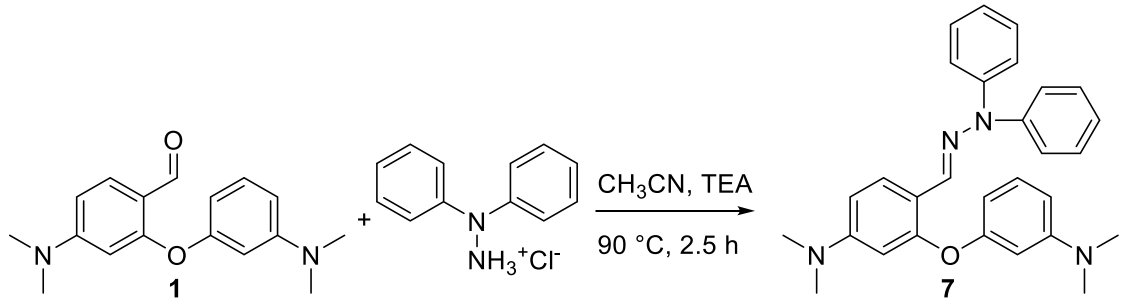

Compound **1** (20 mg, 1 eq), 1,1-Diphenylhydrazine HCl (155 mg, 10 eq) and triethylamine (77 mg, 107 µL, 10 eq) was dissolved in 1 mL of acetonitrile in a sealed screw cap vial and stirred at 90°C for 2.5 hours. Then the solution was diluted with ethyl acetate (10 mL) and washed with water (3*10 mL), the organic layer dried over Na_2SO_4 and evaporated. The product was purified with flash column chromatography (hexane:ethyl acetate 0 to 10% in 10 CV) to give 13 mg (41 %) of product.

^{1}H NMR (400 MHz, CD_3CN) δ 7.96 (d, *J* = 8.9 Hz, 1H), 7.36 – 7.29 (m, 4H), 7.24 (s, 1H), 7.13 – 7.08 (m, 2H), 7.04 – 6.98 (m, 5H), 6.64 (ddd, *J* = 8.9, 2.6, 0.7 Hz, 1H), 6.39 (ddd, *J* = 8.4, 2.4, 0.8 Hz, 1H), 6.26 (d, *J* = 2.6 Hz, 1H), 6.07 (t, *J* = 2.4 Hz, 1H), 5.88 (ddd, *J* = 8.1, 2.3, 0.8 Hz, 1H), 2.91 (s, 6H), 2.86 (s, 6H).

^{13}C NMR (101 MHz, CD_3CN) δ 160.55, 155.91, 153.20, 153.04, 144.86, 133.34, 130.68, 127.48, 124.95, 123.00, 120.25, 116.56, 110.06, 107.71, 104.91, 104.82, 101.80, 40.60, 40.43.

HRMS (ESI-QTOF) m/z: $[M + H]^+$ Calcd for $C_{29}H_{30}N_4O$: 451.2492; Found 451.2499

Compound **8**

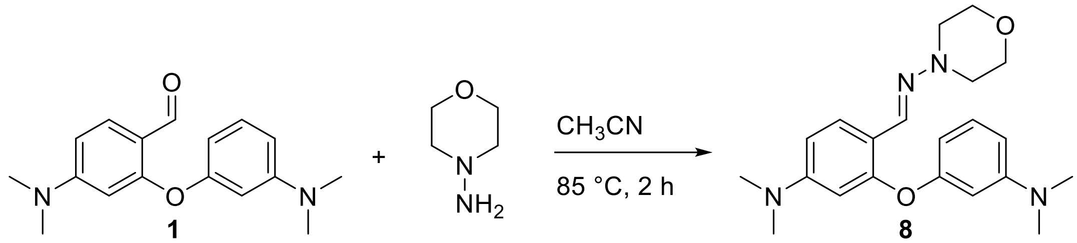

Compound **1** (20 mg, 1 eq) and 4-Aminomorpholine (72 mg, 68 µL, 10 eq) was dissolved in 1 mL of acetonitrile in a sealed screw cap vial and stirred at 85°C for 2 hours. Then the solution was diluted with ethyl acetate (10 mL) and washed with water (3*10 mL), the organic layer dried over Na_2SO_4 and evaporated. The product was purified with flash column chromatography (hexane:ethyl acetate 0 to 20% in 10 CV) to give 15 mg (58 %) of product.

^{1}H NMR (400 MHz, CD_3CN) δ 7.74 (s, 1H), 7.72 (d, *J* = 8.9 Hz, 1H), 7.10 (t, *J* = 8.2 Hz, 1H), 6.58 (dd, *J* = 8.9, 2.6 Hz, 1H), 6.45 (ddd, *J* = 8.3, 2.5, 0.8 Hz, 1H), 6.36 (t, *J* = 2.4 Hz, 1H), 6.26 (d, *J* = 2.6 Hz, 1H), 6.15 (ddd, *J* = 8.1, 2.3, 0.8 Hz, 1H), 3.79 – 3.66 (m, 4H), 2.97 – 2.90 (m, 4H), 2.88 (s, 6H), 2.88 (s, 6H).

^{13}C NMR (101 MHz, CD_3CN) δ 160.40, 156.17, 153.34, 153.00, 133.20, 130.83, 127.15, 116.85, 109.79, 108.00, 105.85, 104.24, 102.62, 66.97, 53.07, 40.64, 40.43.

HRMS (ESI-QTOF) m/z: $[M + H]^+$ Calcd for $C_{21}H_{28}N_4O_2$: 369.2285; Found 369.2287

Compound **9**

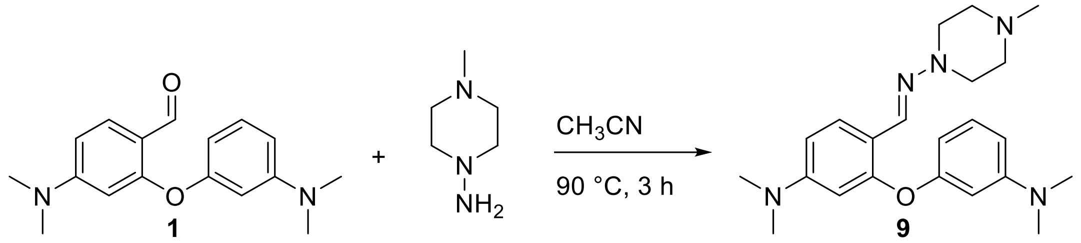

Compound **1** (20 mg, 1 eq) and 1-Amino-4-methylpiperazin (81 mg, 85 µL, 10 eq) was dissolved in 1 mL of acetonitrile in a sealed screw cap vial and stirred at 90°C for 3 hours. Then the solution was diluted with ethyl acetate (10 mL) and washed with water (3*10 mL), the organic layer dried over Na_2SO_4 and evaporated to give 26 mg (97 %) of product.

^{1}H NMR (400 MHz, CD_3CN) δ 7.71 (d, *J* = 8.8 Hz, 1H), 7.66 (s, 1H), 7.10 (t, *J* = 8.2 Hz, 1H), 6.57 (dd, *J* = 8.8, 2.4 Hz, 1H), 6.44 (ddd, *J* = 8.4, 2.5, 0.8 Hz, 1H), 6.35 (t, *J* = 2.4 Hz, 1H), 6.26 (d, *J* = 2.6 Hz, 1H), 6.15 (ddd, *J* = 8.1, 2.3, 0.8 Hz, 1H), 3.01 – 2.91 (m, 4H), 2.88 (s, 6H), 2.87 (s, 6H), 2.49 – 2.39 (m, 4H), 2.21 (s, 3H).

^{13}C NMR (101 MHz, CD_3CN) δ 160.46, 155.97, 153.33, 152.85, 132.58, 130.82, 127.05, 117.22, 109.85, 107.95, 105.79, 104.37, 102.56, 55.16, 52.12, 46.04, 40.65, 40.45.

HRMS (ESI-QTOF) m/z: $[M + H]^+$ Calcd for $C_{22}H_{31}N_5O$: 382.2601; Found 382.2605

Compound **10**

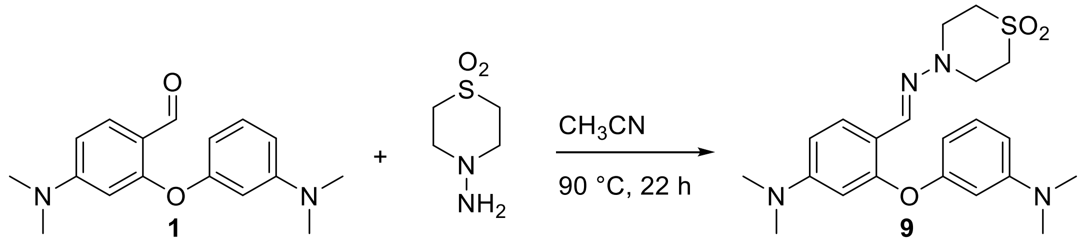

Compound **1** (20 mg, 1 eq) and 4-Aminothiomorpholine 1,1-Dioxide (10 mg, 5 eq) was dissolved in 1 mL of acetonitrile in a sealed screw cap vial and stirred at 90°C for 22 hours. Then the solution was diluted with ethyl acetate (10 mL) and washed with water (3*10 mL), the organic layer dried over Na_2SO_4 and evaporated. The product was purified with flash column chromatography (hexane:ethyl acetate 0 to 30% in 10 CV) to give 9.4 mg (64 %) of product.

^{1}H NMR (400 MHz, CD_3CN) δ 7.74 (s, 1H), 7.71 (d, *J* = 8.8 Hz, 2H), 7.10 (t, *J* = 8.2 Hz, 1H), 6.64 – 6.56 (m, 1H), 6.43 (ddd, *J* = 8.4, 2.5, 0.8 Hz, 1H), 6.33 (t, *J* = 2.4 Hz, 2H), 6.31 (d, *J* = 2.6 Hz, 2H), 6.14 (ddd, *J* = 8.0, 2.3, 0.8 Hz, 1H), 3.73 – 3.60 (m, 4H), 2.91 (s, 6H), 2.87 (s, 6H), 2.86 – 2.78 (m, 4H).

^{13}C NMR (101 MHz, CD_3CN) δ 160.66, 156.17, 153.41, 137.74, 130.94, 127.74, 116.33, 109.84, 108.03, 105.57, 104.54, 102.26, 49.82, 48.74, 40.68, 40.46.

HRMS (ESI-QTOF) m/z: $[M + H]^+$ Calcd for $C_{21}H_{28}N_4O_3S$: 417.1955; Found 417.1947

Compound **SI-3**

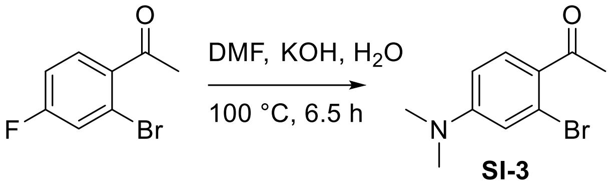

SI-3

Compound **SI-3** was synthetized based on literature.[88] A mixture of 10 mL of DMF and 1 mL of 10 M KOH was heated to 100 °C and then 2'-Bromo-4'-fluoroacetophenone (1 g) was added to the mixture. To the mixture further portions of 1 mL KOH solution was added each subsequent hour (5 mL altogether). The mixture was heated at 100 °C for 6.5 hours, then let to cool to room temperature and stirred overnight. Next day the mixture was diluted with water (50 mL) and washed with ethyl acetate (3*30 mL). The combined organic layers were dried over Na_2SO_4 and evaporated to give 930 mg (83 %) of yellow solid.

^{1}H NMR (400 MHz, $CDCl_3$) δ 7.65 (d, *J* = 8.9 Hz, 1H), 6.87 (d, *J* = 2.6 Hz, 1H), 6.58 (dd, *J* = 8.9, 2.6 Hz, 1H), 3.03 (s, 6H), 2.60 (s, 3H).

^{13}C NMR (101 MHz, $CDCl_3$) δ 197.66, 152.82, 132.78, 126.28, 123.01, 117.02, 109.87, 40.13, 29.79.

Compound **3**

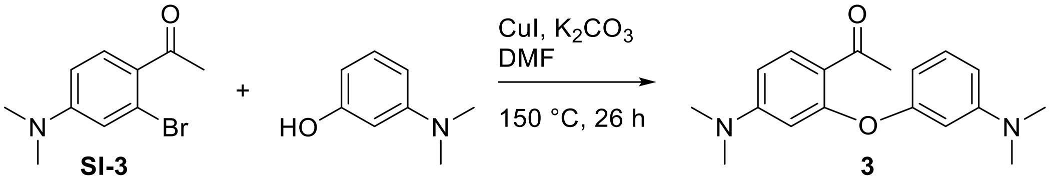

SI-3 **3**

Compound **SI-3** (0.5 g, 1 eq), 3-Dimethylaminophenol (0.283 g, 2 eq), CuI (0.393 g, 1 eq) and K_2CO_3 (0.285 g, 1 eq) was placed in a flame dried flask filled with Argon. The components were dissolved in 5 mL of dry Dimethylformamide and stirred under Argon for 26 hours at 150 °C. Next day the mixture was poured on water, filtered through Cellite, washed with water and ethyl acetate to clear it from copper salts. The supernatant was diluted with ethyl acetate (to 30 mL) and washed with water (3*30 mL), the organic phase dried over Na_2SO_4 and evaporated. The product was purified with flash column chromatography (hexane:ethyl acetate 0 to 20% in 10 CV) and preparative HPLC (CH_3CN:H_2O 10-80% in 30 minutes) to give 170 mg (28 %) of yellow oil, which later solidified and became slightly red in freezer.

^{1}H NMR (400 MHz, CD_3CN) δ 7.78 (d, *J* = 9.0 Hz, 1H), 7.15 (t, *J* = 8.2 Hz, 1H), 6.52 (dd, *J* = 9.0, 2.6 Hz, 1H), 6.51 – 6.46 (m, 1H), 6.40 (t, *J* = 2.4 Hz, 1H), 6.23 (ddd, *J* = 8.0, 2.3, 0.8 Hz, 1H), 6.15 (d, *J* = 2.5 Hz, 1H), 2.91 (s, 6H), 2.89 (s, 6H), 2.45 (s, 3H).

^{13}C NMR (101 MHz, CD_3CN) δ 195.96, 159.43, 159.09, 155.73, 153.38, 132.84, 130.98, 118.86, 108.46, 108.24, 106.44, 103.31, 102.80, 40.62, 40.17, 31.30.

HRMS (ESI-QTOF) m/z: $[M + H]^+$ Calcd for $C_{18}H_{22}N_2O_2$: 299.1754; Found 299.1755

Compound **11**

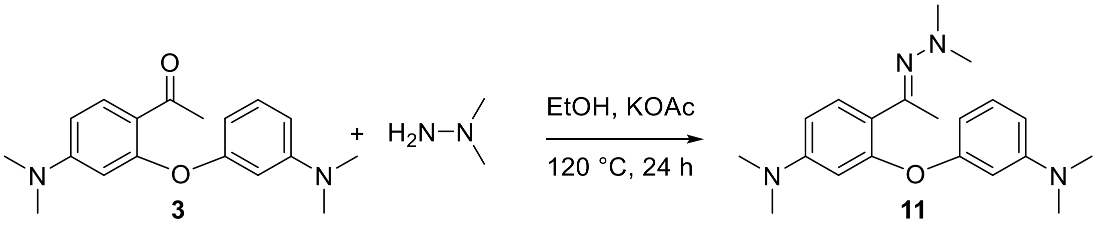

Compound **3** (20 mg), N, N-dimethylhydrazine (80 mg, 0.1 mL, 20 eq) and potassium acetate (66 mg, 10 eq) was dissolved in 1 mL of ethanol in a sealed screw cap vial and the solution was heated at 120 °C overnight. Next day the solution was cooled, diluted with ethyl acetate (10 mL) and washed with water (3*10 mL), the organic layer dried over Na_2SO_4 and evaporated. The product was purified with flash column chromatography (hexane:ethyl acetate 0 to 40% in 10 CV), then again dissolved in ethyl acetate and passed through a small plug of silica to give 4 mg (18 %) of product.

^{1}H NMR (400 MHz, CD_3CN) δ 7.34 (d, J = 8.7 Hz, 1H), 7.09 (t, J = 8.2 Hz, 1H), 6.54 (dd, J = 8.7, 2.6 Hz, 1H), 6.42 (ddd, J = 8.4, 2.5, 0.8 Hz, 1H), 6.30 (t, J = 2.4 Hz, 1H), 6.28 (d, J = 2.6 Hz, 1H), 6.13 (ddd, J = 8.1, 2.3, 0.8 Hz, 1H), 2.88 (s, 6H), 2.86 (s, 6H), 2.36 (s, 6H), 2.16 (s, 3H).

^{13}C NMR (101 MHz, CD_3CN) δ 163.53, 159.88, 155.61, 153.33, 153.23, 131.26, 130.69, 121.76, 108.95, 107.76, 105.74, 104.83, 102.48, 47.19, 40.64, 40.44, 18.71.

HRMS (ESI-QTOF) m/z: $[M + H]^+$ Calcd for $C_{20}H_{28}N_4O$: 341.2336; Found 341.2338

Compound **12**

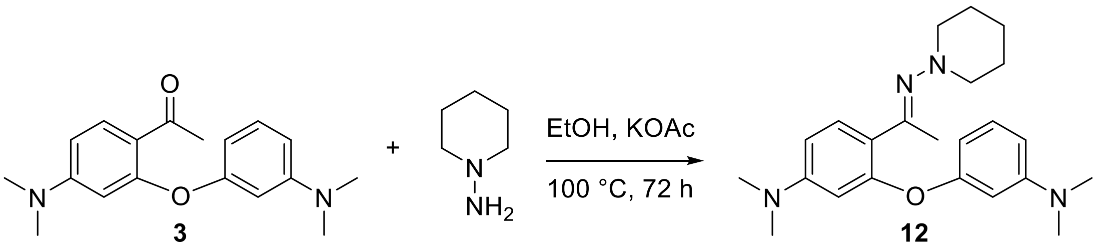

Compound **3** (20 mg), 1-Aminopiperidine (134 mg, 0.143 mL, 20 eq) and potassium acetate (26 mg, 4 eq) was dissolved in 1 mL of ethanol in a sealed screw cap vial and the solution was heated at 100 °C for three days. Then the solution was cooled, diluted with ethyl acetate (10 mL) and washed with water (3*10 mL), the organic layer dried over Na_2SO_4 and evaporated. The product was purified with flash column chromatography (hexane:ethyl acetate 0 to 30% in 10 CV) to give 10 mg (39 %) of product.

^{1}H NMR (400 MHz, CD_3CN) δ 7.34 (d, J = 8.7 Hz, 1H), 7.08 (t, J = 8.2 Hz, 1H), 6.54 (dd, J = 8.7, 2.5 Hz, 1H), 6.42 (ddd, J = 8.4, 2.5, 0.8 Hz, 1H), 6.29 (t, J = 2.4 Hz, 1H), 6.27 (d, J = 2.6 Hz, 1H), 6.12 (ddd, J = 8.1, 2.3, 0.8 Hz, 1H), 2.88 (s, 6H), 2.86 (s, 6H), 2.59 – 2.52 (m, 4H), 2.17 (s, 3H), 1.68 – 1.57 (m, 4H), 1.47 – 1.38 (m, 2H).

^{13}C NMR (101 MHz, CD_3CN) δ 163.66, 159.92, 155.62, 153.32, 153.24, 131.32, 130.69, 121.87, 108.98, 107.75, 105.70, 104.89, 102.46, 56.67, 40.66, 40.45, 26.09, 24.73, 18.69.

HRMS (ESI-QTOF) m/z: $[M + H]^+$ Calcd for $C_{23}H_{32}N_4O$: 381.2649; Found 381.2650

Compound **13**

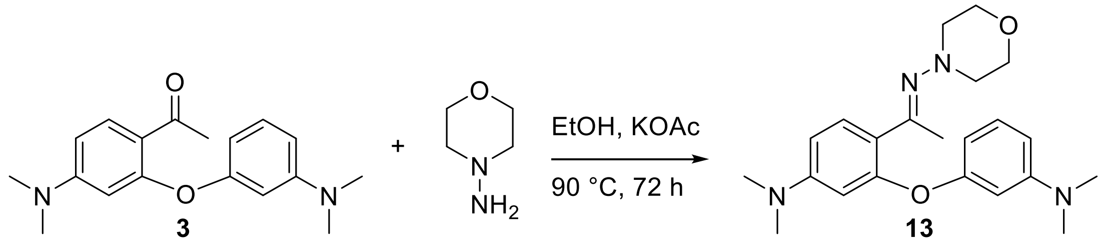

Compound **3** (20 mg), 4-Aminomorpholine (137 mg, 0.129 mL, 20 eq) and potassium acetate (13 mg, 2 eq) was dissolved in 1 mL of ethanol in a sealed screw cap vial and the solution was heated at 90 °C for three days. Then the solution was cooled, diluted with ethyl acetate (10 mL) and washed with water (3*10 mL), the organic layer dried over Na_2SO_4 and evaporated. The product was purified with flash column chromatography (hexane:ethyl acetate 0 to 50% in 10 CV), then again dissolved in ethyl acetate and passed through a small plug of silica to give 11 mg (43 %) of product.

^{1}H NMR (400 MHz, CD_3CN) δ 7.36 (d, *J* = 8.7 Hz, 1H), 7.08 (t, *J* = 8.2 Hz, 1H), 6.54 (dd, *J* = 8.7, 2.6 Hz, 1H), 6.42 (ddd, *J* = 8.4, 2.5, 0.8 Hz, 1H), 6.29 (t, *J* = 2.4 Hz, 1H), 6.28 (d, *J* = 2.5 Hz, 1H), 6.13 (ddd, *J* = 8.1, 2.3, 0.8 Hz, 1H), 3.72 – 3.64 (m, 4H), 2.88 (s, 6H), 2.86 (s, 6H), 2.64 – 2.56 (m, 4H), 2.20 (s, 3H).

^{13}C NMR (101 MHz, CD_3CN) δ 164.70, 159.85, 155.70, 153.43, 153.23, 131.30, 130.72, 121.46, 108.93, 107.80, 105.74, 104.80, 102.48, 66.78, 55.80, 40.65, 40.43, 18.83.

HRMS (ESI-QTOF) m/z: $[M + H]^+$ Calcd for $C_{22}H_{30}N_4O_2$: 383.2442; Found 383.2446

Compound **14**

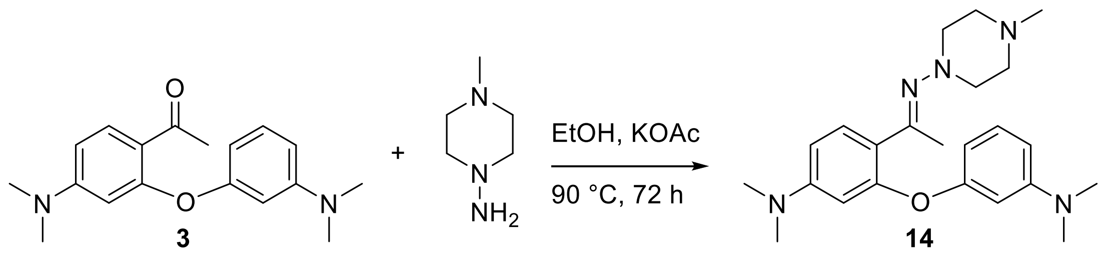

Compound **3** (20 mg), 1-Amino-4-methylpiperazin (154 mg, 0.161 mL, 20 eq) and potassium acetate (13 mg, 2 eq) was dissolved in 1 mL of ethanol in a sealed screw cap vial and the solution was heated at 90 °C for three days. Then the solution was cooled, diluted with ethyl acetate (10 mL) and washed with water (3*10 mL), the organic layer dried over Na_2SO_4 and evaporated. The product was purified with flash column chromatography (dicholormethane:methanol 0 to 10% in 10 CV), then again dissolved in ethyl acetate and passed through a small plug of silica to give 6 mg (23 %) of product.

^{1}H NMR (400 MHz, CD_3CN) δ 7.35 (d, *J* = 8.7 Hz, 1H), 7.08 (t, *J* = 8.2 Hz, 1H), 6.54 (dd, *J* = 8.7, 2.6 Hz, 1H), 6.42 (ddd, *J* = 8.4, 2.5, 0.8 Hz, 1H), 6.30 – 6.27 (m, 2H), 6.13 (ddd, *J* = 8.1, 2.3, 0.8 Hz, 1H), 2.88 (s, 6H), 2.86 (s, 6H), 2.63 (t, *J* = 4.9 Hz, 4H), 2.51 – 2.38 (m, 4H), 2.20 (s, 3H), 2.17 (s, 3H).

^{13}C NMR (101 MHz, CD_3CN) δ 164.34, 159.89, 155.63, 153.38, 153.23, 131.29, 130.71, 121.66, 108.96, 107.77, 105.72, 104.85, 102.45, 55.10, 55.06, 45.97, 40.66, 40.44, 18.79.

HRMS (ESI-QTOF) m/z: $[M + 2H]^+$ Calcd for $C_{23}H_{33}N_5O$: 198.6415; Found 198.6411

Compound **SI-4**

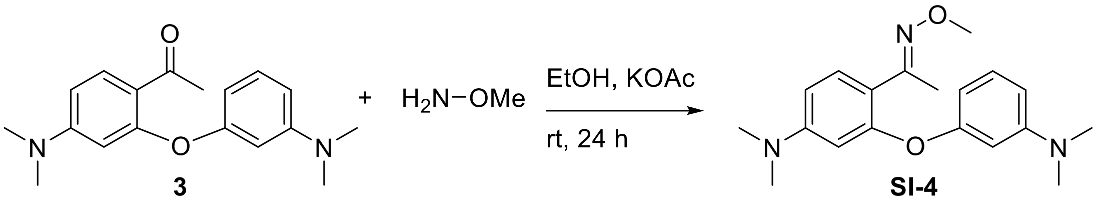

Compound 3 (10 mg), Methoxyamine Hydrochloride (28 mg, 10 eq) and potassium acetate (13 mg, 4 eq) was dissolved in 1 mL of ethanol in a sealed screw cap vial and the solution was stirred at room temperature overnight. Then the solution was diluted with ethyl acetate (10 mL) and washed with water (3*10 mL), the organic layer dried over Na_2SO_4 and evaporated to give 10 mg (91 %) of product

^{1}H NMR (400 MHz, CD_3CN) δ 7.28 (d, *J* = 8.7 Hz, 1H), 7.10 (t, *J* = 8.2 Hz, 1H), 6.53 (dd, *J* = 8.7, 2.6 Hz, 1H), 6.45 (ddd, *J* = 8.4, 2.5, 0.8 Hz, 1H), 6.32 (t, *J* = 2.4 Hz, 1H), 6.28 (d, *J* = 2.6 Hz, 1H), 6.15 (ddd, *J* = 8.1, 2.3, 0.8 Hz, 1H), 3.82 (s, 3H), 2.87 (s, 6H), 2.87 (s, 6H), 2.05 (s, 3H).

^{13}C NMR (101 MHz, CD_3CN) δ 159.68, 155.98, 155.94, 153.43, 153.27, 131.20, 130.78, 118.41, 108.77, 108.00, 105.84, 104.70, 102.65, 61.76, 40.62, 40.42, 15.93.

HRMS (ESI-QTOF) m/z: $[M + H]^+$ Calcd for $C_{19}H_{25}N_3O_2$: 328.2020; Found 328.2018

Compound **SI-5**

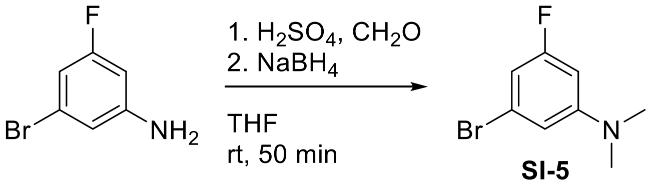

The compound **SI-5** was synthetized based on literature.[89] Aqueous formaldehyde (37%, 1 mL) was added to a mixture of THF (10 mL) and 3 M H_2SO_4 (1 mL) and stirred at room temperature for 10 minutes, then 3-Bromo-5-Fluoroaniline (1 g, 0.59 mL) was added and the mixture was stirred for 50 minutes. After 50 minutes 1 g of $NaBH_4$ was slowly added to the flask while cooling it in an ice bath. The reaction mixture was diluted with ethyl acetate (50 mL) and washed with saturated $NaHCO_3$ solution (3*50 mL), the organic layer dried over Na_2SO_4 and evaporated. The product was purified with flash column chromatography (hexane:ethyl acetate 0 to 20% in 10 CV) to give 306 mg (27 %) of product.

^{1}H NMR (400 MHz, $CDCl_3$) δ 6.60 – 6.57 (m, 1H), 6.56 (dt, *J* = 8.0, 2.0 Hz, 1H), 6.29 (dt, *J* = 12.4, 2.3 Hz, 1H), 2.94 (s, 6H).

^{13}C NMR (101 MHz, $CDCl_3$) δ 163.73 (d, *J* = 245.4 Hz), 152.38 (d, *J* = 11.6 Hz), 123.05 (d, *J* = 13.3 Hz), 110.84 (d, *J* = 2.5 Hz), 106.35 (d, *J* = 25.4 Hz), 97.93 (d, *J* = 25.9 Hz), 40.26.

HRMS (ESI-QTOF) m/z: $[M + H]^+$ Calcd for C_8H_9BrFN: 229.9975; Found 229.9975

Compound **27**

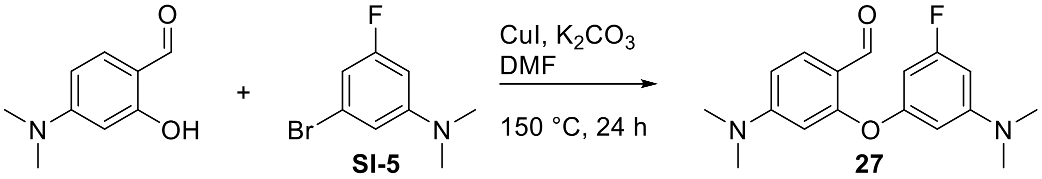

Compound **SI-5** (300 mg, 1 eq), 4-(Dimethylamino)salicylaldehyde (227 mg, 1 eq), CuI (262 mg, 0.5 eq) and K_2CO_3 (380 mg, 2 eq) was placed in a flame dried flask filled with Argon. The components were dissolved in 10 mL of dry Dimethylformamide and stirred under Argon for 24 hours at 150 °C. Next day the mixture was poured on water, filtered through Cellite, washed with water and ethyl acetate to clear it from copper salts. The supernatant was diluted with ethyl acetate (to 30 mL) and washed with water (3*30 mL), the organic phase dried over Na_2SO_4 and evaporated. The product was purified with flash column chromatography (hexane:ethyl acetate 0 to 30% in 10 CV) to give 90 mg of regenerated **SI-5** and 90 mg (22 %) of yellow oil as product.

^{1}H NMR (400 MHz, $CDCl_3$) δ 10.12 (d, *J* = 0.8 Hz, 1H), 7.82 (d, *J* = 8.9 Hz, 1H), 6.50 (ddd, *J* = 9.0, 2.4, 0.8 Hz, 1H), 6.19 (td, *J* = 2.3, 0.7 Hz, 1H), 6.18 – 6.12 (m, 2H), 6.03 (dt, *J* = 9.8, 2.2 Hz, 1H), 3.01 (s, 6H), 2.94 (s, 6H).

^{13}C NMR (101 MHz, $CDCl_3$) δ 187.31, 164.63 (d, *J* = 241.5 Hz), 161.08, 159.38 (d, *J* = 14.2 Hz), 155.81, 152.65 (d, *J* = 13.1 Hz), 130.12, 116.86, 107.90, 101.13, 98.20 (d, *J* = 2.5 Hz), 94.67 (d, *J* = 26.3 Hz), 93.85 (d, *J* = 26.0 Hz), 40.48, 40.26.

HRMS (ESI-QTOF) m/z: $[M + H]^+$ Calcd for $C_{17}H_{19}FN_2O_2$: 303.1503; Found 303.1514

Compound **SI-6**

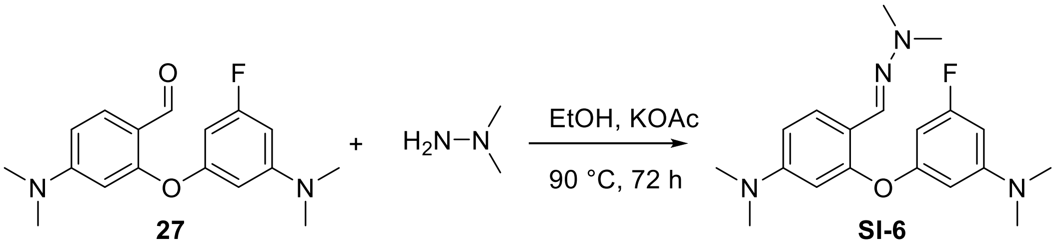

Compound **27** (10 mg, 1 eq), N, N-dimethylhydrazine (20 mg, 25 µL, 10 eq) and potassium acetate (16 mg, 5 eq) was dissolved in 1 mL of ethanol in a sealed screw cap vial and the solution was heated at 90 °C for 3 days. Then the solution was diluted with ethyl acetate (10 mL) and washed with water (3*10 mL), the organic layer dried over Na_2SO_4 and evaporated to give 9 mg (79 %) of yellow oil.

^{1}H NMR (400 MHz, $CDCl_3$) δ 7.81 (d, *J* = 8.8 Hz, 1H), 7.44 (s, 1H), 6.56 (dd, *J* = 8.9, 2.6 Hz, 1H), 6.26 (d, *J* = 2.6 Hz, 1H), 6.13 (td, *J* = 2.2, 0.8 Hz, 1H), 6.08 (dt, *J* = 12.2, 2.3 Hz, 1H), 5.96 (dt, *J* = 10.2, 2.2 Hz, 1H), 2.92 (s, 7H), 2.91 (s, 7H), 2.83 (s, 6H).

^{13}C NMR (101 MHz, $CDCl_3$) δ 164.68 (d, *J* = 240.8 Hz), 160.53 (d, *J* = 14.3 Hz), 154.10, 152.51 (d, *J* = 13.1 Hz), 151.52, 130.12, 126.49, 116.85, 109.66, 104.05, 97.12 (d, *J* = 2.0 Hz), 93.67 (d, *J* = 26.5 Hz), 92.95 (d, *J* = 26.1 Hz), 43.45, 40.54, 40.51.

HRMS (ESI-QTOF) m/z: $[M + H]^+$ Calcd for $C_{19}H_{25}FN_4O$: 345.2085; Found 345.2090

Compound **31**

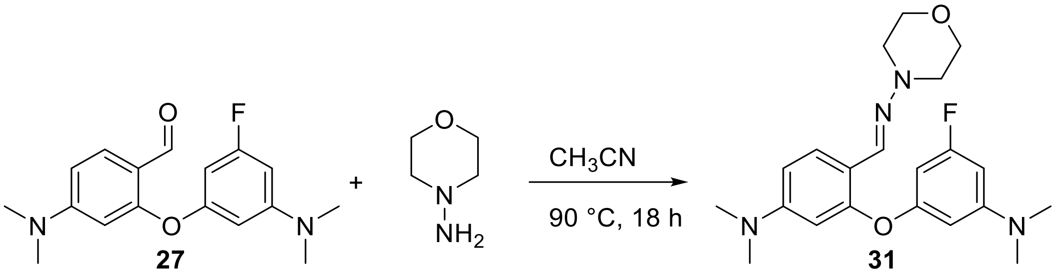

Compound **27** (10 mg, 1 eq) and 4-Aminomorpholine (34 mg, 32 µL, 10 eq) was dissolved in 1 mL of acetonitrile in a sealed screw cap vial and the solution was heated at 90 °C overnight. Next day the solution was diluted with ethyl acetate (10 mL) and washed with water (3*10 mL), the organic layer dried over Na_2SO_4 and evaporated to give 11 mg (86 %) of product.

^{1}H NMR (400 MHz, $CDCl_3$) δ 7.84 (d, *J* = 8.8 Hz, 1H), 7.78 (s, 1H), 6.55 (dd, *J* = 8.8, 2.6 Hz, 1H), 6.25 (d, *J* = 2.5 Hz, 1H), 6.15 – 6.04 (m, 2H), 5.95 (dt, *J* = 10.1, 2.1 Hz, 1H), 3.88 – 3.76 (m, 4H), 3.08 – 3.02 (m, 4H), 2.93 (s, 6H), 2.91 (s, 6H).

^{13}C NMR (101 MHz, $CDCl_3$) δ 164.68 (d, *J* = 241.0 Hz), 160.39 (d, *J* = 14.0 Hz), 154.75, 152.52 (d, *J* = 13.0 Hz), 152.07, 133.64, 126.78, 116.25, 109.48, 103.67, 97.12 (d, *J* = 2.2 Hz), 93.79 (d, *J* = 26.5 Hz), 92.99 (d, *J* = 26.2 Hz), 66.68, 52.48, 40.50, 40.45.

HRMS (ESI-QTOF) m/z: $[M + H]^+$ Calcd for $C_{21}H_{27}FN_4O_2$: 387.2191; Found 387.2190

Compound **32**

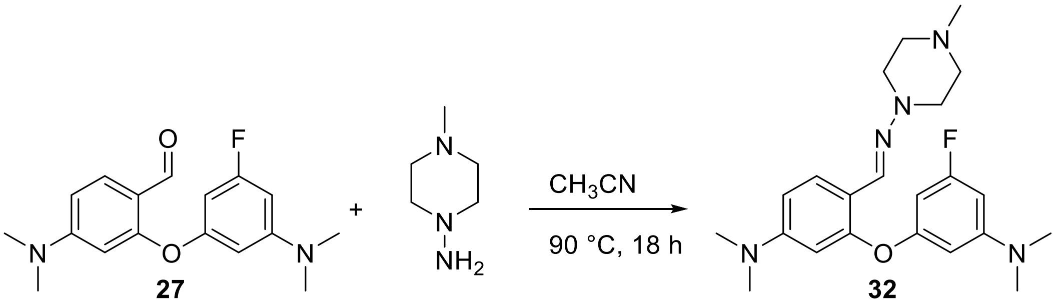

Compound **27** (10 mg, 1 eq) and 1-Amino-4-methylpiperazin (38 mg, 40 µL, 10 eq) was dissolved in 1 mL of acetonitrile in a sealed screw cap vial and the solution was heated at 90 °C overnight. Next day the solution was diluted with ethyl acetate (10 mL) and washed with water (3*10 mL), the organic layer dried over Na_2SO_4 and evaporated to give 12 mg (91 %) of product.

^{1}H NMR (400 MHz, $CDCl_3$) δ 7.84 (d, *J* = 8.8 Hz, 1H), 7.72 (s, 1H), 6.55 (dd, *J* = 8.9, 2.6 Hz, 1H), 6.24 (d, *J* = 2.5 Hz, 1H), 6.11 (t, *J* = 2.3 Hz, 1H), 6.08 (dt, *J* = 12.1, 2.3 Hz, 1H), 5.95 (dt, *J* = 10.1, 2.2 Hz, 1H), 3.09 (t, *J* = 5.0 Hz, 4H), 2.92 (s, 6H), 2.90 (s, 6H), 2.56 (t, *J* = 5.1 Hz, 4H), 2.31 (s, 3H).

^{13}C NMR (101 MHz, $CDCl_3$) δ 164.67 (d, *J* = 240.8 Hz), 160.44 (d, *J* = 14.0 Hz), 154.59, 152.49 (d, *J* = 13.1 Hz), 151.92, 133.18, 126.71, 116.64, 109.52, 103.77, 97.10 (d, *J* = 2.4 Hz), 93.74 (d, *J* = 26.4 Hz), 93.00 (d, *J* = 26.1 Hz), 54.71, 51.55, 46.07, 40.49, 40.47.

HRMS (ESI-QTOF) m/z: $[M + H]^+$ Calcd for $C_{22}H_{30}FN_5O$: 400.2507; Found 400.2509

Compound **SI-7**

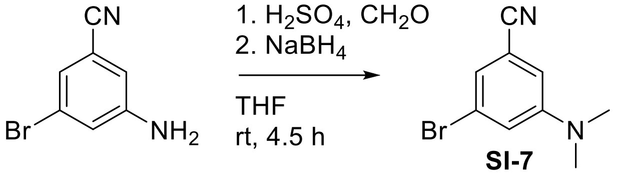

Compound **SI-7** was synthetized based on literature.[89] Aqueous formaldehyde (37%, 2 mL) was added to a mixture of THF (20 mL) and 3 M H_2SO_4 (2 mL) and stirred at room temperature for 10 minutes, then 5-Amino-3-bromobenzonitrile (1 g) was added and the mixture was stirred for 4.5 hours. After 4.5 hours 1 g of $NaBH_4$ was slowly added to the flask while cooling it in an ice bath. The reaction mixture was diluted with ethyl acetate (50 mL) and washed with saturated $NaHCO_3$ solution (3*50 mL), the organic layer dried over Na_2SO_4 and evaporated. The product was purified with flash column chromatography (hexane:ethyl acetate 0 to 20% in 10 CV) to give 436 mg (38 %) of product and carried straight on to the next reaction.

^{1}H NMR (400 MHz, CD_3CN) δ 7.09 (d, *J* = 1.9 Hz, 2H), 6.97 (t, *J* = 1.9 Hz, 1H), 2.95 (s, 6H).

^{13}C NMR (101 MHz, CD_3CN) δ 152.46, 124.01, 121.51, 119.47, 119.17, 114.97, 114.88, 40.37.

HRMS (ESI-QTOF) m/z: $[M + H]^+$ Calcd for $C_9H_9BrN_2$: 225.0022; Found 225.0023

Compound **28**

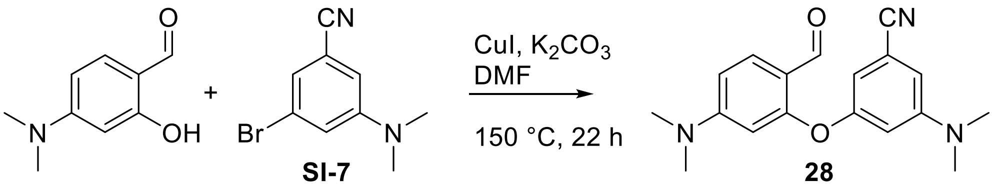

4-(Dimethylamino)salicylaldehyde (319 mg, 1 eq), compound **SI-7** (435 mg, 1 eq), CuI (184 mg, 0.5 eq) and K_2CO_3 (533 mg, 2 eq) was placed in a flame dried flask filled with Argon. The components were dissolved in 10 mL of dry Dimethylformamide and stirred under Argon for 22 hours at 150 °C. After 22 hours, according to LC-MS, most starting materials were consumed, so the mixture was poured on water, filtered through Cellite, washed with water and ethyl acetate to clear it from copper salts. The supernatant was diluted with ethyl acetate (to 30 mL) and washed with water (3*30 mL), the organic phase dried over Na_2SO_4 and evaporated. The product was purified with flash column chromatography (hexane:ethyl acetate 0 to 30% in 10 CV) to give 266 mg (45 %) of yellow solid.

^{1}H NMR (400 MHz, $CDCl_3$) δ 10.05 (s, 1H), 7.83 (d, *J* = 8.9 Hz, 1H), 6.65 (dd, *J* = 2.5, 1.3 Hz, 1H), 6.58 (t, *J* = 2.3 Hz, 1H), 6.53 (dd, *J* = 8.9, 2.4 Hz, 1H), 6.49 (t, *J* = 1.7 Hz, 1H), 6.08 (d, *J* = 2.4 Hz, 1H), 3.03 (s, 6H), 2.98 (s, 6H).

^{13}C NMR (101 MHz, $CDCl_3$) δ 186.93, 160.29, 159.17, 155.83, 151.96, 130.62, 119.30, 116.83, 113.71, 110.62, 108.37, 108.26, 106.08, 101.41, 40.34, 40.27.

HRMS (ESI-QTOF) m/z: $[M + H]^+$ Calcd for $C_{18}H_{19}N_3O_2$: 310.1550; Found 310.1555

Compound **33**

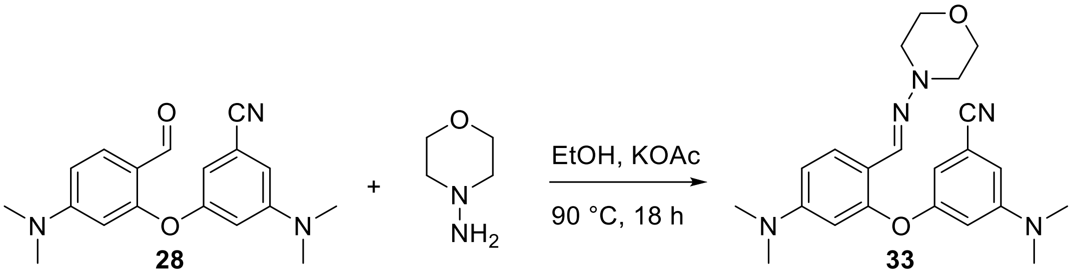

Compound **28** (25 mg, 1 eq), 4-Aminomorpholine (165 mg, 156 µL, 20 eq) and potassium acetate (32 mg, 4 eq) was dissolved in 1 mL of ethanol in a sealed screw cap vial and stirred at 90°C for 18 hours. Then the solution was diluted with ethyl acetate (10 mL) and washed with water (3*10 mL), the organic layer dried over Na_2SO_4 and evaporated. The compound was purified with preparative HPLC (CH_3CN:H_2O 10-80% in 30 minutes) to give 16 mg (50 %) of product.

^{1}H NMR (400 MHz, $CDCl_3$) δ 7.84 (d, *J* = 8.9 Hz, 1H), 7.71 (s, 1H), 6.61 – 6.54 (m, 2H), 6.49 (t, *J* = 2.3 Hz, 1H), 6.47 – 6.40 (m, 1H), 6.18 (d, *J* = 2.6 Hz, 1H), 3.88 – 3.77 (m, 4H), 3.08 – 3.00 (m, 4H), 2.94 (s, 6H), 2.94 (s, 6H).

^{13}C NMR (101 MHz, $CDCl_3$) δ 159.88, 154.14, 152.08, 151.83, 133.06, 127.09, 119.48, 116.16, 113.54, 109.84, 109.81, 107.75, 105.13, 103.50, 66.62, 52.40, 40.40, 40.34.

HRMS (ESI-QTOF) m/z: $[M + H]^+$ Calcd for $C_{22}H_{27}N_5O_2$: 394.2238; Found 394.2240

Compound **34**

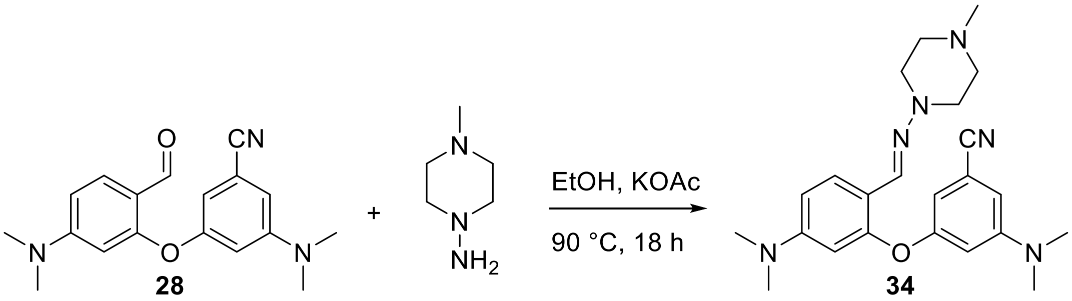

Compound **28** (25 mg, 1 eq), 1-Amino-4-methylpiperazin (186 mg, 194 µL, 20 eq) and potassium acetate (32 mg, 4 eq) was dissolved in 1 mL of ethanol in a sealed screw cap vial and stirred at 90°C for 18 hours. Then the solution was diluted with ethyl acetate (10 mL) and washed with water (3*10 mL), the organic layer dried over Na_2SO_4 and evaporated. The compound was purified with preparative HPLC (CH_3CN:H_2O 10-80% in 30 minutes) to give 19 mg (58 %) of product.

^{1}H NMR (400 MHz, $CDCl_3$) δ 7.84 (d, *J* = 8.9 Hz, 1H), 7.65 (s, 1H), 6.62 – 6.52 (m, 2H), 6.48 (s, 1H), 6.43 (s, 1H), 6.19 (s, 1H), 3.08 (t, *J* = 5.1 Hz, 4H), 2.97 – 2.89 (m, 12H), 2.56 (t, *J* = 5.1 Hz, 4H), 2.31 (s, 3H).

^{13}C NMR (101 MHz, $CDCl_3$) δ 159.92, 153.97, 151.94, 151.81, 132.68, 127.02, 119.48, 116.50, 113.49, 109.87, 109.74, 107.76, 105.08, 103.60, 54.54, 51.38, 45.93, 40.41, 40.32.

HRMS (ESI-QTOF) m/z: $[M + H]^+$ Calcd for $C_{23}H_{30}N_6O$: 407.2554; Found 407.2542

Compound **SI-8**

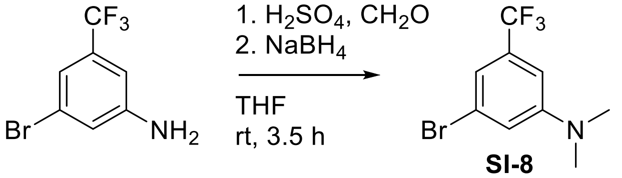

Compound **SI-8** was synthetized based on literature.[89] Aqueous formaldehyde (37%, 2 mL) was added to a mixture of THF (20 mL) and 3 M H_2SO_4 (2 mL) and stirred at room temperature for 10 minutes, then 3-Bromo-5-(trifluoromethyl)aniline (1 g, 0.589 mL) was added and the mixture was stirred for 3.5 hours. After 3.5 hours 1 g of $NaBH_4$ was slowly added to the flask while cooling it in an ice bath. The reaction mixture was diluted with ethyl acetate (50 mL) and washed with saturated $NaHCO_3$ solution (3*50 mL), the organic layer dried over Na_2SO_4 and evaporated. The product was purified with flash column chromatography (hexane:ethyl acetate 0 to 10% in 10 CV) to give 552 mg (49 %) of transparent liquid as product, which slowly crystalized later in the freezer.

^{1}H NMR (400 MHz, $CDCl_3$) δ 7.04 (t, *J* = 1.7 Hz, 1H), 6.93 (t, *J* = 2.1 Hz, 1H), 6.78 (t, *J* = 2.0 Hz, 1H), 2.99 (s, 6H).

^{13}C NMR (101 MHz, $CDCl_3$) δ 151.40, 132.78 (q, *J* = 32.2 Hz), 123.75 (q, *J* = 273.0 Hz), 123.58, 117.64, 115.42 (q, *J* = 3.9 Hz), 107.31 (q, *J* = 3.9 Hz), 40.36.

HRMS (ESI-QTOF) m/z: $[M + H]^+$ Calcd for $C_9H_9BrF_3N$: 267.9943; Found 267.9949

Compound **29**

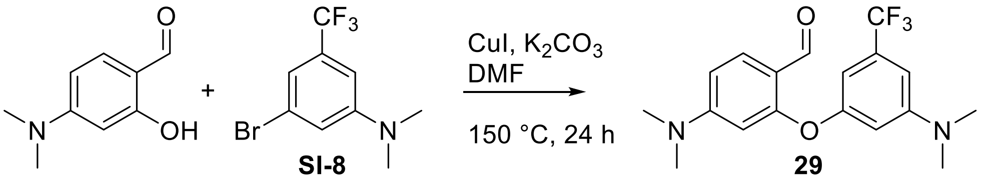

4-(Dimethylamino)salicylaldehyde (0.27 g, 0.8 eq), compound **SI-7** (0.54 g, 1 eq), CuI (0.192 g, 0.5 eq) and K_2CO_3 (0.556 g, 2 eq) was placed in a flame dried flask filled with Argon. The components were dissolved in 10 mL of dry Dimethylformamide and stirred under Argon for 19 hours at 150 °C. After 19 hours, another 0.192 g of CuI (0.5 eq) was added to the mixture and it was stirred again at 150 °C for another 5 hours. After altogether 24 hours, according to LC-MS, most starting materials were consumed, so the mixture was poured on water, filtered through Cellite, washed with water and ethyl acetate to clear it from copper salts. The supernatant was diluted with ethyl acetate (to 30 mL) and washed with water (3*30 mL), the organic phase dried over Na_2SO_4 and evaporated. The product was purified with flash column chromatography (hexane:ethyl acetate 0 to 30% in 10 CV) to give 305 mg (43 %) of yellow solid.

^{1}H NMR (400 MHz, $CDCl_3$) δ 10.13 (d, *J* = 0.8 Hz, 1H), 7.83 (d, *J* = 8.9 Hz, 1H), 6.65 (t, *J* = 1.9 Hz, 1H), 6.55 (t, *J* = 1.8 Hz, 1H), 6.53 (t, *J* = 2.3 Hz, 1H), 6.50 (ddd, *J* = 8.9, 2.5, 0.8 Hz, 1H), 6.08 (d, *J* = 2.4 Hz, 1H), 3.00 (s, 6H), 2.98 (s, 6H).

^{13}C NMR (101 MHz, $CDCl_3$) δ 187.14, 160.99, 158.65, 155.80, 152.06, 132.62 (q, *J* = 32.0 Hz), 130.29, 124.18 (q, *J* = 272.7 Hz), 116.66, 107.90, 105.12, 104.08 (q, *J* = 4.1 Hz), 102.70 (q, *J* = 3.8 Hz), 100.72, 40.45, 40.22.

HRMS (ESI-QTOF) m/z: $[M + H]^+$ Calcd for $C_{18}H_{19}F_3N_2O_2$: 353.1471; Found 353.1463

Compound **35**

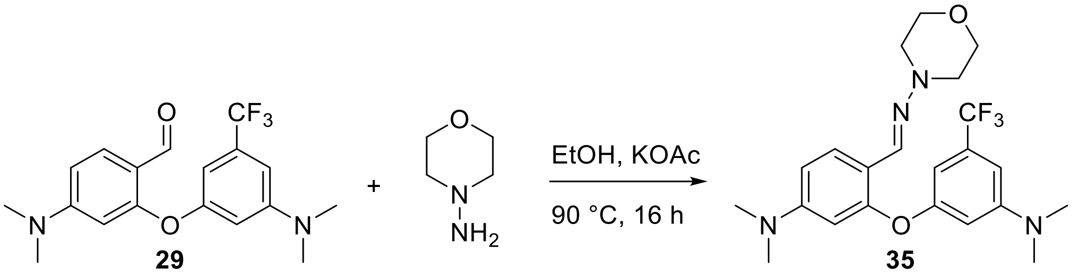

Compound **29** (15 mg, 1 eq), 4-Aminomorpholine (87 mg, 82 µL, 20 eq) and potassium acetate (32 mg, 10 eq) was dissolved in 1 mL of ethanol in a sealed screw cap vial and stirred at 90°C for 16 hours. Then the solution was diluted with ethyl acetate (10 mL) and washed with water (3*10 mL), the organic layer dried over Na_2SO_4 and evaporated to give 18 mg (97 %) of product.

^{1}H NMR (400 MHz, $CDCl_3$) δ 7.84 (d, *J* = 8.9 Hz, 1H), 7.79 (s, 1H), 6.59 (t, *J* = 2.0 Hz, 1H), 6.55 (dd, *J* = 8.9, 2.6 Hz, 1H), 6.50 (t, *J* = 1.8 Hz, 1H), 6.43 (t, *J* = 2.3 Hz, 1H), 6.21 (d, *J* = 2.5 Hz, 1H), 3.85 – 3.78 (m, 4H), 3.08 – 3.01 (m, 4H), 2.95 (s, 6H), 2.92 (s, 6H).

^{13}C NMR (101 MHz, $CDCl_3$) δ 159.49, 154.60, 151.94, 151.82, 133.43, 132.35 (q, *J* = 31.9 Hz), 126.79, 124.15 (q, *J* = 272.8 Hz), 115.92, 109.32, 103.97, 103.10, 103.06 (q, *J* = 4.3 Hz), 101.95 (q, *J* = 3.8 Hz), 66.53, 52.35, 40.37, 40.30.

HRMS (ESI-QTOF) m/z: $[M + H]^+$ Calcd for $C_{22}H_{27}F_3N_4O_2$: 437.2159; Found 437.2166

Compound **36**

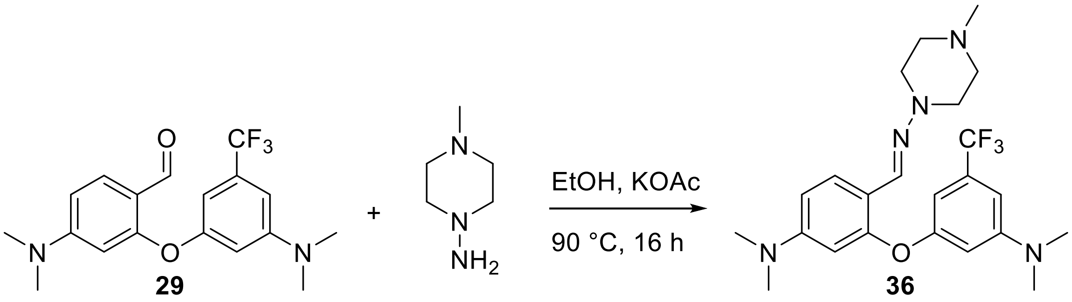

Compound **29** (15 mg, 1 eq), 1-Amino-4-methylpiperazin (98 mg, 102 µL, 20 eq) and potassium acetate (32 mg, 10 eq) was dissolved in 1 mL of ethanol in a sealed screw cap vial and stirred at 90°C for 16 hours. Then the solution was diluted with ethyl acetate (10 mL) and washed with water (3*10 mL), the organic layer dried over Na_2SO_4 and evaporated to give 15 mg (78 %) of product.

^{1}H NMR (400 MHz, $CDCl_3$) δ 7.85 (d, *J* = 8.8 Hz, 1H), 7.73 (s, 1H), 6.58 (t, *J* = 2.0 Hz, 1H), 6.55 (dd, *J* = 8.9, 2.6 Hz, 1H), 6.50 (t, *J* = 1.9 Hz, 1H), 6.42 (t, *J* = 2.3 Hz, 1H), 6.21 (d, *J* = 2.6 Hz, 1H), 3.09 (t, *J* = 5.1 Hz, 4H), 2.94 (s, 6H), 2.92 (s, 6H), 2.55 (t, *J* = 5.1 Hz, 4H), 2.31 (s, 3H).

^{13}C NMR (101 MHz, $CDCl_3$) δ 159.70, 154.57, 151.94, 133.10, 132.45 (q, *J* = 31.8 Hz), 126.86, 124.30 (q, *J* = 272.6 Hz), 116.46, 109.52, 104.08, 103.37, 103.14 (q, *J* = 4.0 Hz), 102.11 (q, *J* = 3.8 Hz), 54.68, 51.55, 46.06, 40.50, 40.46.

HRMS (ESI-QTOF) m/z: $[M + H]^+$ Calcd for $C_{23}H_{30}F_3N_5O$: 450.2475; Found 450.2477

Compound **SI-9**

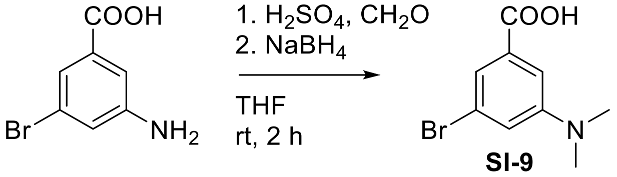

Compound **SI-9** was synthetized based on literature.[89] Aqueous formaldehyde (37%, 10 mL) was added to a mixture of THF (20 mL) and 3 M H_2SO_4 (10 mL) and stirred at room temperature for 10 minutes, then 5-Amino-3-bromobenzoic acid (5 g) was added and the mixture was stirred for 2 hours. After 2 hours 1.5 g of $NaBH_4$ was slowly added to the flask while cooling it in an ice bath. The reaction mixture was diluted with ethyl acetate (50 mL) and washed with saturated $NaHCO_3$ solution (3*50 mL), the organic layer dried over Na_2SO_4 and evaporated to give 5.6 g (99 %) of product.

^{1}H NMR (400 MHz, DMSO) δ 7.26 (t, *J* = 1.5 Hz, 1H), 7.19 (dd, *J* = 2.6, 1.3 Hz, 1H), 7.04 (dd, *J* = 2.6, 1.8 Hz, 1H), 6.52 (s, 1H), 2.95 (s, 6H).

^{13}C NMR (101 MHz, DMSO) δ 166.59, 151.35, 133.12, 122.52, 118.47, 117.83, 111.42, 39.88.

HRMS (ESI-QTOF) m/z: $[M + H]^+$ Calcd for $C_9H_{10}BrNO_2$: 243.9968; Found 243.9967

Compound **SI-10**

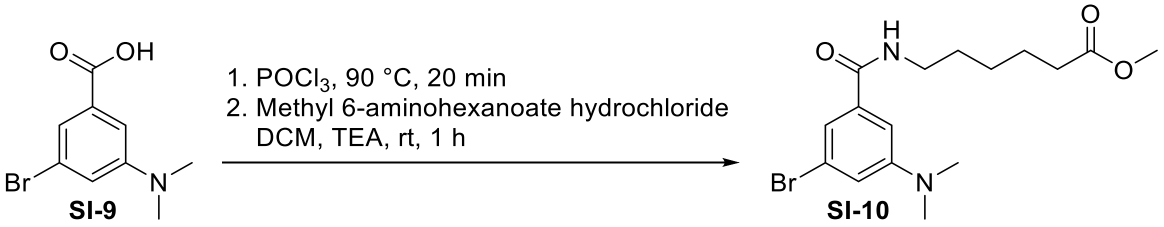

Compound **SI-9** (300 mg, 1 eq) was dissolved in 2 mL of $POCl_3$ and the solution was stirred at 90 °C for 20 minutes, during which the solution turned deep red. The solution was then cooled to room temperature, diluted with 20 mL of hexane, and the hexane layer decanted, to give a red oil. This red oil was then dissolved in 10 mL of DCM, to which Methyl 6-aminohexanoate hydrochloride (290 mg, 1.3 eq) and 1 mL of triethylamine was added and the reaction mixture stirred at room temperature for 1 hour, then the solution was washed with water (3*30 mL), the organic layer dried over Na_2SO_4 and evaporated. The product was purified with flash column chromatography (hexane:ethyl acetate 20 to 80% in 10 CV) to give 180 mg (39 %) of oil as product.

^{1}H NMR (400 MHz, $CDCl_3$) δ 7.15 (s, 1H), 7.11 (s, 1H), 6.96 (s, 1H), 6.17 (s, 1H), 3.67 (s, 3H), 3.44 (q, *J* = 6.9 Hz, 2H), 3.00 (s, 6H), 2.34 (t, *J* = 7.3 Hz, 2H), 1.71 – 1.60 (m, 4H), 1.48 – 1.35 (m, 2H).

^{13}C NMR (101 MHz, $CDCl_3$) δ 174.26, 166.93, 151.23, 137.37, 123.43, 117.89, 117.36, 110.68, 51.73, 40.82, 39.88, 33.93, 29.28, 26.44, 24.43.

HRMS (ESI-QTOF) m/z: $[M + H]^+$ Calcd for $C_{16}H_{23}BrN_2O_3$: 371.0965; Found 371.0962

Compound **SI-11**

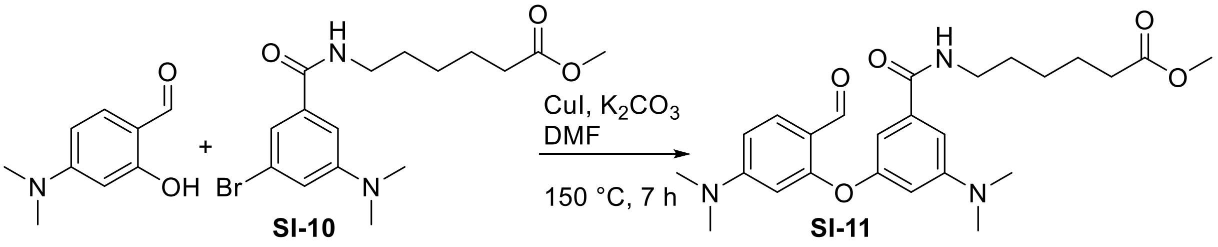

4-(Dimethylamino)salicylaldehyde (382 mg, 1 eq), compound **SI-10** (860 mg, 1 eq), CuI (221 mg, 0.5 eq) and K_2CO_3 (640 mg, 2 eq) was placed in a flame dried flask filled with Argon. The components were dissolved in 20 mL of dry Dimethylformamide and stirred under Argon for 7 hours at 150 °C. After 7 hours, according to LC-MS, most starting materials were consumed, so the mixture was poured on water, filtered through Cellite, washed with water and ethyl acetate to clear it from copper salts. The supernatant was diluted with ethyl acetate (to 30 mL) and washed with water (3*30 mL), the organic phase dried over Na_2SO_4 and evaporated. The product was purified with flash column chromatography (hexane:ethyl acetate 20 to 80% in 10 CV) to give 325 mg (45 %) of light brown oil.

^{1}H NMR (400 MHz, $CDCl_3$) δ 10.15 (s, 1H), 7.81 (d, *J* = 8.9 Hz, 1H), 6.94 (dd, *J* = 2.4, 1.4 Hz, 1H), 6.62 – 6.56 (m, 1H), 6.51 (t, *J* = 2.3 Hz, 1H), 6.49 – 6.43 (m, 1H), 6.16 (t, *J* = 5.9 Hz, 1H), 6.03 (d, *J* = 2.4 Hz, 1H), 3.64 (s, 3H), 3.41 (td, *J* = 7.2, 5.9 Hz, 2H), 2.98 (s, 12H), 2.31 (t, *J* = 7.4 Hz, 2H), 1.66 – 1.56 (m, 4H), 1.43 – 1.34 (m, 2H).

^{13}C NMR (101 MHz, $CDCl_3$) δ 187.32, 174.21, 167.86, 161.67, 158.16, 155.84, 152.17, 137.33, 130.22, 116.50, 107.53, 107.04, 105.53, 104.34, 100.30, 51.65, 40.60, 40.26, 39.90, 33.96, 29.34, 26.51, 24.53.

Compound **SI-12**

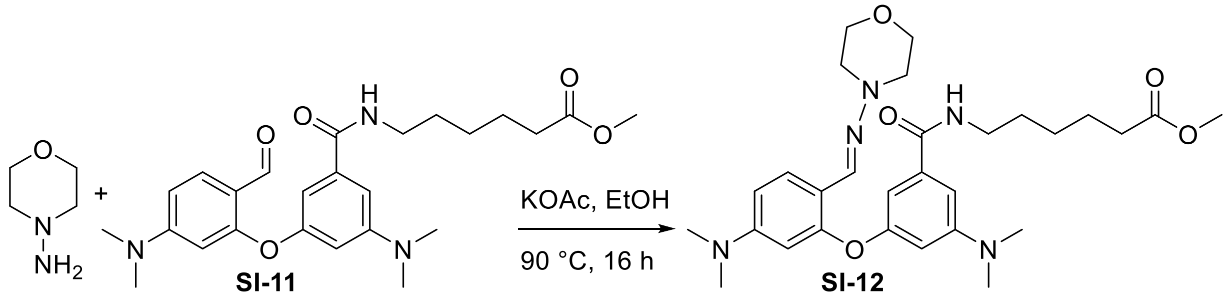

Compound **SI-11** (20 mg, 1 eq), 4-Aminomorpholine (45 mg, 43 µL, 10 eq) and potassium acetate (34 mg, 8 eq) was dissolved in 1 mL of ethanol in a sealed screw cap vial and stirred at 90°C for 16 hours. Then the solution was diluted with ethyl acetate (10 mL) and washed with water (3*10 mL), the organic layer dried over Na_2SO_4 and evaporated. The compound was purified with preparative HPLC (CH_3CN:H_2O 10-80% in 30 minutes) to give 6 mg (25 %) of product.

^{1}H NMR (400 MHz, CD_3CN) δ 7.73 (d, *J* = 8.9 Hz, 1H), 7.70 (s, 1H), 6.96 (t, *J* = 6.0 Hz, 1H), 6.79 (dd, *J* = 2.4, 1.5 Hz, 1H), 6.60 (dd, *J* = 8.9, 2.6 Hz, 1H), 6.52 – 6.44 (m, 2H), 6.27 (d, *J* = 2.6 Hz, 1H), 3.75 – 3.68 (m, 4H), 3.59 (s, 3H), 3.26 (td, *J* = 7.0, 5.9 Hz, 2H), 2.95 – 2.88 (m, 16H), 2.28 (t, *J* = 7.5 Hz, 2H), 1.65 – 1.54 (m, 2H), 1.57 – 1.47 (m, 2H), 1.39 – 1.26 (m, 2H).

^{13}C NMR (101 MHz, CD_3CN) δ 174.79, 167.89, 160.63, 155.82, 153.10, 153.07, 138.12, 133.10, 127.36, 116.98, 110.15, 106.32, 104.67, 104.53, 104.22, 67.01, 53.10, 51.89, 40.74, 40.49, 40.17, 34.41, 29.88, 27.09, 25.36.

HRMS (ESI-QTOF) m/z: $[M + H]^+$ Calcd for $C_{29}H_{41}N_5O_5$: 540.3180; Found 540.3183

Compound **SI-13**

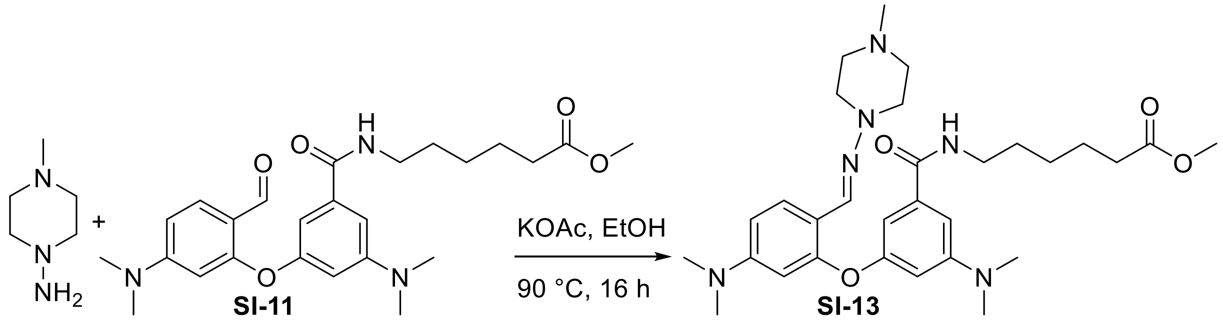

Compound **SI-11** (20 mg, 1 eq), 1-Amino-4-methylpiperazin (51 mg, 53 µL, 10 eq) and potassium acetate (34 mg, 8 eq) was dissolved in 1 mL of ethanol in a sealed screw cap vial and stirred at 90°C for 16 hours. Then the solution was diluted with ethyl acetate (10 mL) and washed with water (3*10 mL), the organic layer dried over Na_2SO_4 and evaporated. The compound was purified with preparative HPLC (CH_3CN:H_2O 10-80% in 30 minutes) to give 2 mg (8 %) of product.

^{1}H NMR (400 MHz, CD_3CN) δ 7.71 (d, *J* = 8.9 Hz, 1H), 7.62 (s, 1H), 6.94 (s, 1H), 6.79 (t, *J* = 1.9 Hz, 1H), 6.60 (dd, *J* = 8.8, 2.6 Hz, 1H), 6.50 – 6.44 (m, 2H), 6.28 (d, *J* = 2.6 Hz, 1H), 3.59 (s, 3H), 3.26 (q, *J* = 6.7 Hz, 2H), 2.98 – 2.88 (m, 16H), 2.43 (t, *J* = 5.1 Hz, 4H), 2.28 (t, *J* = 7.5 Hz, 2H), 2.21 (s, 3H), 1.65 – 1.56 (m, 2H), 1.52 (dd, *J* = 14.8, 7.2 Hz, 2H), 1.39 – 1.30 (m, 2H).

^{13}C NMR (101 MHz, CD_3CN) δ 174.77, 167.85, 160.70, 155.62, 153.07, 152.96, 138.13, 132.50, 127.26, 117.34, 110.20, 106.27, 104.67, 104.60, 104.16, 55.17, 52.14, 51.88, 46.07, 40.74, 40.51, 40.15, 34.41, 29.89, 27.09, 25.36.

HRMS (ESI-QTOF) m/z: $[M + H]^+$ Calcd for $C_{30}H_{44}N_6O_4$: 553.3497; Found 553.3512

Compound **30 (PULI$_{560}$)**

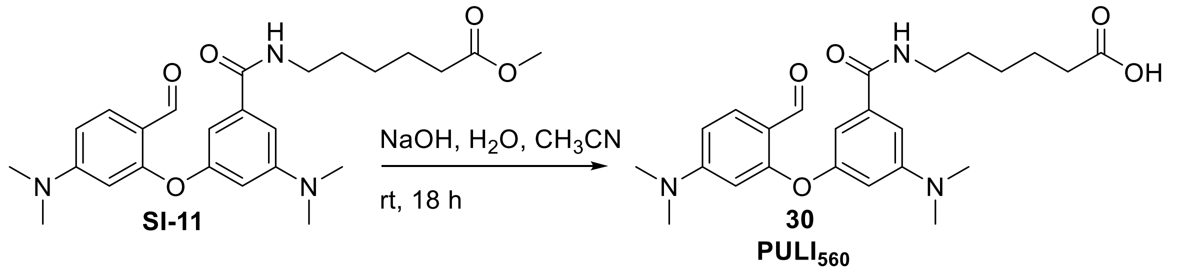

Compound **SI-11** (290 mg) was dissolved in a mixture of 3 mL of acetonitrile and 1 mL of 1 M NaOH aqueous solution and stirred at room temperature for 18 hours. The solution was neutralized by slowly adding 1 mL of 1 M HCl, then the solution was diluted with dichloromethane (10 mL) and washed with water (3*10 mL), the organic layer dried over Na_2SO_4 and evaporated to give 267 mg (95%) of pink solid.

^{1}H NMR (400 MHz, CD_3CN) δ 10.05 (s, 1H), 8.91 (bs, 1H), 7.72 (d, *J* = 9.0 Hz, 1H), 6.98 (t, *J* = 6.4 Hz, 1H), 6.88 (dd, *J* = 2.4, 1.4 Hz, 1H), 6.62 (t, *J* = 1.8 Hz, 1H), 6.60 – 6.54 (m, 2H), 6.16 (d, *J* = 2.4 Hz, 1H), 3.28 (q, *J* = 6.7 Hz, 2H), 2.97 (s, 6H), 2.95 (s, 6H), 2.26 (t, *J* = 7.4 Hz, 2H), 1.63 – 1.50 (m, 4H), 1.39 – 1.29 (m, 2H).

^{13}C NMR (101 MHz, CD_3CN) δ 186.83, 174.62, 167.29, 161.67, 159.25, 156.58, 152.73, 137.90, 130.26, 116.86, 108.33, 106.79, 105.23, 104.73, 101.57, 40.29, 39.93, 39.77, 33.65, 29.44, 26.66, 24.82.

HRMS (ESI-QTOF) m/z: $[M + H]^+$ Calcd for $C_{24}H_{31}N_3O_5$: 442.2336; Found 442.2335

Compound **37 (PULI$_{560}$-M)**

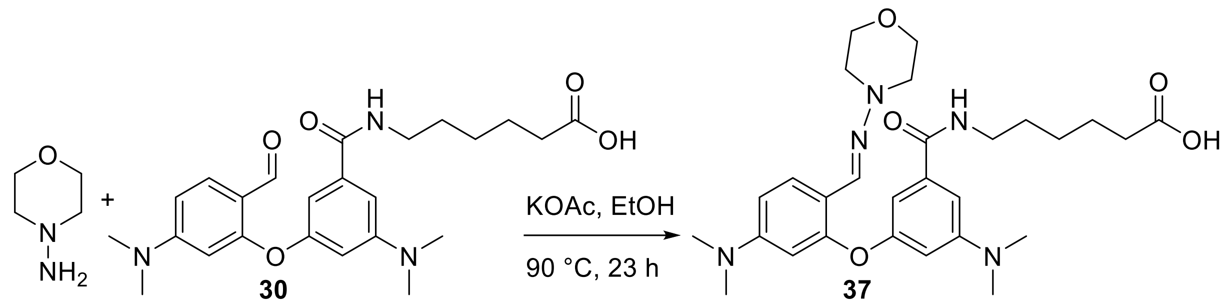

30
PULI$_{560}$

37
PULI$_{560}$-M

Compound **PULI$_{560}$** (100 mg, 1 eq), 4-Aminomorpholine (116 mg, 109 µL, 5 eq) and potassium acetate (88 mg, 4 eq) was dissolved in 2 mL of ethanol in a sealed screw cap vial and stirred at 90°C for 23 hours. Then the solution was diluted with ethyl acetate (10 mL) and washed with water (3*10 mL), the organic layer dried over Na_2SO_4 and evaporated. The compound was purified with preparative HPLC (CH_3CN:H_2O 10-80% in 30 minutes) and then with flash column chromatography (dichloromethane: methanol 0 to 5% in 10 CV) to give 47 mg (39 %) of reddish product.

^{1}H NMR (400 MHz, CD_3CN) δ 7.73 (d, *J* = 8.9 Hz, 1H), 7.71 (s, 1H), 6.94 (t, *J* = 6.3 Hz, 1H), 6.80 (t, *J* = 1.9 Hz, 1H), 6.61 (dd, *J* = 8.8, 2.6 Hz, 1H), 6.50 – 6.44 (m, 2H), 6.28 (d, *J* = 2.6 Hz, 1H), 3.78 – 3.66 (m, 4H), 3.27 (q, *J* = 6.7 Hz, 2H), 2.95 – 2.88 (m, 16H), 2.26 (t, *J* = 7.4 Hz, 2H), 1.61 – 1.47 (m, 4H), 1.38 – 1.29 (m, 2H).

^{13}C NMR (101 MHz, CD_3CN) δ 175.05, 167.86, 160.67, 155.82, 153.14, 153.07, 138.14, 133.32, 127.38, 117.02, 110.20, 106.32, 104.64, 104.13, 66.99, 53.11, 40.74, 40.50, 40.12, 34.07, 29.86, 27.05, 25.22.

HRMS (ESI-QTOF) m/z: $[M + H]^+$ Calcd for $C_{28}H_{39}N_5O_5$: 526.3024; Found 526.3025

Compound **38 (PULI$_{560}$-P)**

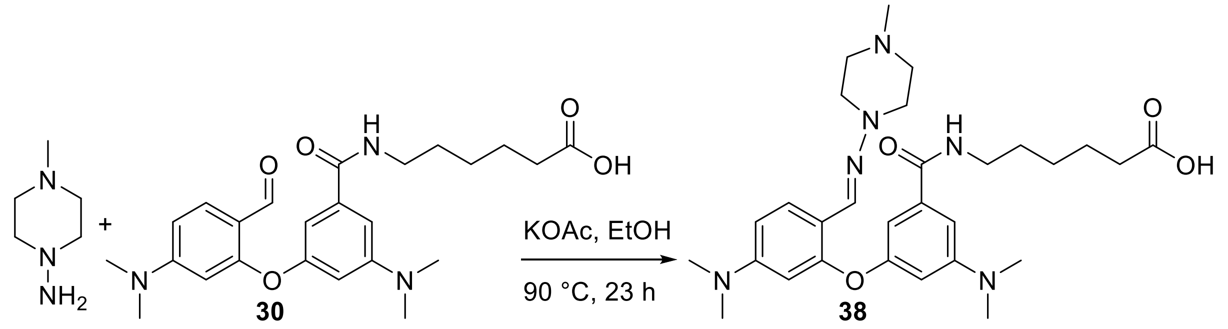

30
PULI$_{560}$

38
PULI$_{560}$-P

Compound **PULI$_{560}$** (100 mg, 1 eq), 1-Amino-4-methylpiperazin (130 mg, 136 µL, 5 eq) and potassium acetate (88 mg, 4 eq) was dissolved in 2 mL of ethanol in a sealed screw cap vial and stirred at 90°C for 23 hours. Then the solution was diluted with ethyl acetate (10 mL) and washed with water (3*10 mL), the organic layer dried over Na_2SO_4 and evaporated. The compound was purified with flash column chromatography (dichloromethane: methanol 0 to 20% in 10 CV) to give 122 mg (99 %) of yellow product.

^{1}H NMR (400 MHz, CD_3CN) δ 7.73 (d, *J* = 8.9 Hz, 1H), 7.54 (s, 1H), 6.95 (t, *J* = 6.0 Hz, 1H), 6.78 (dd, *J* = 2.4, 1.4 Hz, 1H), 6.64 (dd, *J* = 8.9, 2.6 Hz, 1H), 6.55 (t, *J* = 2.3 Hz, 1H), 6.34 (d, *J* = 2.6 Hz, 1H), 6.27 (t, *J* = 1.8 Hz, 1H), 3.28 (q, *J* = 6.1 Hz, 2H), 2.96 (s, 6H), 2.93 (s, 6H), 2.90 – 2.86 (m, 4H), 2.62 – 2.59 (m, 4H), 2.26 (s, 3H), 2.21 – 2.17 (m, 2H), 1.57 – 1.45 (m, 4H), 1.33 – 1.21 (m, 2H).

^{13}C NMR (101 MHz, CD_3CN) δ 176.53, 167.66, 161.29, 155.29, 153.26, 153.08, 137.87, 134.08, 127.34, 117.41, 110.57, 106.00, 105.40, 104.27, 102.82, 54.33, 51.42, 44.90, 40.77, 40.48, 40.13, 34.99, 30.02, 27.29, 25.78.

HRMS (ESI-QTOF) m/z: $[M + 2H]^{2+}$ Calcd for $C_{29}H_{42}N_6O_4$: 270.1707; Found 270.1701

Compound **39 (PULI$_{560}$-T)**

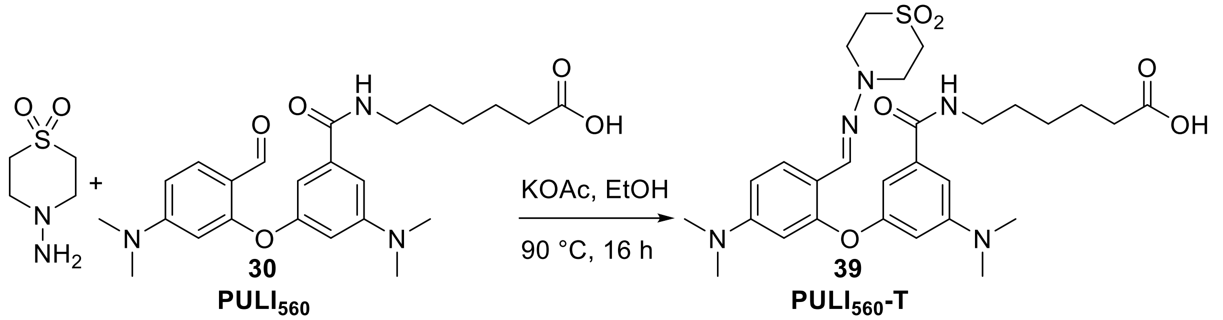

Compound **PULI$_{560}$** (20 mg, 1 eq), 4-Aminothiomorpholine 1,1-Dioxide (68 mg, 10 eq) and potassium acetate (44 mg, 10 eq) was dissolved in 2 mL of ethanol in a sealed screw cap vial and stirred at 90°C for 16 hours. Then the solution was diluted with ethyl acetate (10 mL) and washed with water (3*10 mL), the organic layer dried over Na_2SO_4 and evaporated to give 20 mg (77 %) of reddish product.

^{1}H NMR (400 MHz, CD_3CN) δ 7.75 – 7.66 (m, 2H), 6.95 (t, *J* = 5.9 Hz, 1H), 6.75 (t, *J* = 1.9 Hz, 1H), 6.63 (dd, *J* = 8.9, 2.6 Hz, 1H), 6.47 – 6.40 (m, 2H), 6.35 (d, *J* = 2.6 Hz, 1H), 3.70 – 3.62 (m, 4H), 3.32 – 3.21 (m, 2H), 2.93 (s, 6H), 2.93 (s, 6H), 2.80 – 2.73 (m, 4H), 2.26 (t, *J* = 7.4 Hz, 2H), 1.61 – 1.50 (m, 4H), 1.40 – 1.30 (m, 2H).

^{13}C NMR (101 MHz, CD_3CN) δ 175.07, 168.01, 160.88, 155.68, 153.48, 153.10, 138.34, 137.65, 128.07, 116.46, 110.16, 106.24, 104.93, 104.10, 103.81, 49.71, 48.61, 40.74, 40.48, 40.18, 34.08, 29.88, 27.09, 25.25.

HRMS (ESI-QTOF) m/z: $[M + H]^+$ Calcd for $C_{28}H_{39}N_5O_6S$: 574.2694; Found 574.2703

Compound **46 (PULI$_{560}$-M-NHS)**

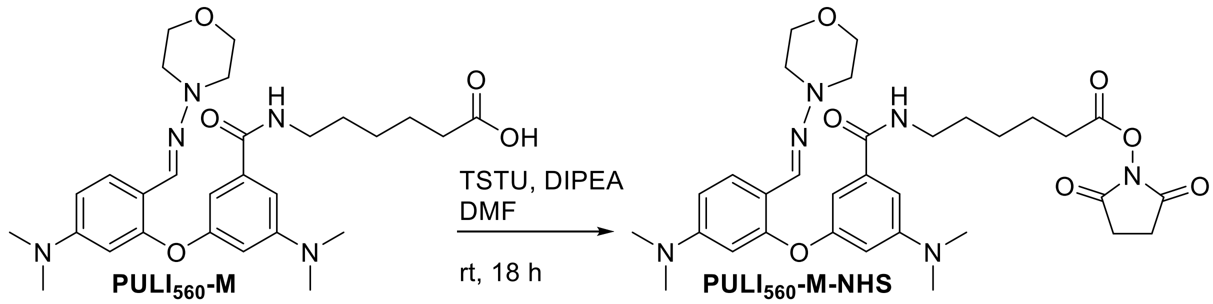

PULI$_{560}$-M (10 mg, 1 eq), TSTU (N,N,N′,N′-Tetramethyl-O-(N-succinimidyl)uroniumtetrafluoroborat, 8.6 mg, 1.5 eq), and DIPEA (N,N-Diisopropylethylamine, 3.7 mg, 5 µL, 1.5 eq) was dissolved in 1 mL of dry DMF in a closed cap vial and stirred at room temperature for 18 hours. Then the compound was diluted with acetonitrile and purified with preparative HPLC (CH_3CN:H_2O 10-80% in 30 minutes) to give 6.6 mg (56 %) of product.

^{1}H NMR (400 MHz, CD_3CN) δ 7.73 (d, *J* = 8.9 Hz, 1H), 7.71 (s, 1H), 6.97 (t, *J* = 6.0 Hz, 1H), 6.80 (dd, *J* = 2.4, 1.4 Hz, 1H), 6.61 (dd, *J* = 8.9, 2.6 Hz, 1H), 6.49 (dt, *J* = 11.1, 2.1 Hz, 2H), 6.27 (d, *J* = 2.6 Hz, 1H), 3.77 – 3.64 (m, 4H), 3.28 (q, *J* = 6.7 Hz, 2H), 2.96 – 2.85 (m, 16H), 2.75 (s, 4H), 2.61 (t, *J* = 7.4 Hz, 2H), 1.71 (p, *J* = 7.4 Hz, 2H), 1.64 – 1.50 (m, 2H), 1.50 – 1.35 (m, 2H).

^{13}C NMR (101 MHz, CD_3CN) δ 171.16, 170.13, 167.85, 160.61, 155.82, 153.09, 153.07, 138.12, 133.05, 127.35, 116.98, 110.14, 106.34, 104.67, 104.51, 104.26, 67.01, 53.09, 40.74, 40.49, 40.03, 31.37, 29.67, 26.69, 26.38, 25.10.

HRMS (ESI-QTOF) m/z: $[M + H]^+$ Calcd for $C_{33}H_{43}N_5O_7$: 623.3188; Found 623.3204

Compound **47 (PULI$_{560}$-P-NHS)**

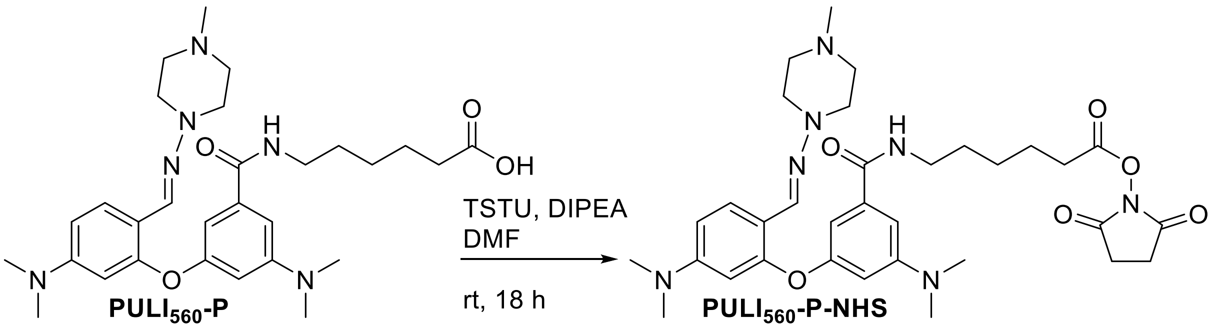

PULI$_{560}$-P (10 mg, 1 eq), TSTU (N,N,N′,N′-Tetramethyl-O-(N-succinimidyl)uroniumtetrafluoroborat, 8.4 mg, 1.5 eq), and DIPEA (N,N-Diisopropylethylamine, 3.6 mg, 4.8 µL, 1.5 eq) was dissolved in 1 mL of dry DMF in a closed cap vial and stirred at room temperature for 18 hours. Then the compound was diluted with acetonitrile and purified with preparative HPLC (CH_3CN:H_2O 10-80% in 30 minutes) to give 4 mg (34 %) of product.

^{1}H NMR (400 MHz, CD_3CN) δ 7.72 (d, *J* = 8.8 Hz, 1H), 7.63 (s, 1H), 6.97 (t, *J* = 6.0 Hz, 1H), 6.79 (dd, *J* = 2.4, 1.4 Hz, 1H), 6.60 (dd, *J* = 8.9, 2.6 Hz, 1H), 6.53 – 6.43 (m, 2H), 6.28 (d, *J* = 2.6 Hz, 1H), 3.28 (q, *J* = 6.6 Hz, 2H), 2.96 – 2.88 (m, 16H), 2.75 (s, 4H), 2.61 (t, *J* = 7.4 Hz, 2H), 2.44 (t, *J* = 5.1 Hz, 4H), 2.21 (s, 3H), 1.61 – 1.50 (m, 4H), 1.47 – 1.41 (m, 2H).

^{13}C NMR (101 MHz, CD_3CN) δ 171.16, 170.13, 167.87, 160.67, 155.64, 153.07, 152.96, 138.11, 132.56, 127.26, 117.30, 110.19, 106.30, 104.64, 104.61, 104.21, 55.12, 52.09, 46.02, 40.75, 40.51, 40.03, 31.37, 29.67, 26.69, 26.38, 25.35.

HRMS (ESI-QTOF) m/z: $[M + H]^+$ Calcd for $C_{34}H_{46}N_6O_6$: 636.3504; Found 636.3493

Compound **48 (PULI$_{560}$-T-NHS)**

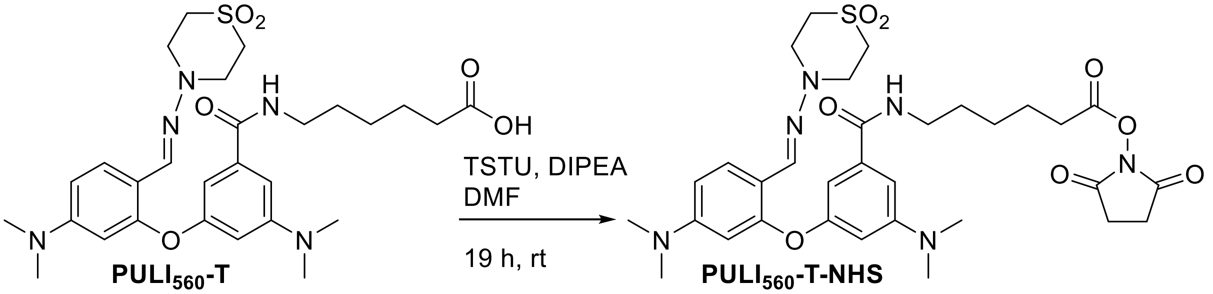

PULI$_{560}$-T (6.5 mg, 1 eq), TSTU (N,N,N′,N′-Tetramethyl-O-(N-succinimidyl)uroniumtetrafluoroborat, 5.1 mg, 1.5 eq), and DIPEA (N,N-Diisopropylethylamine, 2.2 mg, 3.0 µL, 1.5 eq) was dissolved in 1 mL of dry DMF in a closed cap vial and stirred at room temperature for 19 hours. Then the compound was diluted with acetonitrile and purified with preparative HPLC (CH_3CN:H_2O 10-80% in 30 minutes) to give 6 mg (79 %) of product.

^{1}H NMR (400 MHz, CD_3CN) δ 7.75 – 7.63 (m, 2H), 6.95 (t, *J* = 6.3 Hz, 1H), 6.75 (t, *J* = 1.9 Hz, 1H), 6.63 (dd, *J* = 8.9, 2.6 Hz, 1H), 6.51 – 6.39 (m, 2H), 6.35 (d, *J* = 2.6 Hz, 1H), 3.74 – 3.55 (m, 4H), 3.33 – 3.22 (m, 2H), 2.95 (s, 6H), 2.81 – 2.73 (m, 8H), 2.61 (t, *J* = 7.3 Hz, 2H), 1.72 (p, *J* = 7.4 Hz, 2H), 1.56 (p, *J* = 7.0 Hz, 2H), 1.48 – 1.39 (m, 2H).

^{13}C NMR (101 MHz, CD_3CN) δ 170.74, 169.71, 167.56, 160.42, 155.26, 153.04, 152.67, 137.91, 137.13, 127.63, 116.01, 109.70, 105.82, 104.43, 103.69, 103.45, 49.29, 48.19, 40.32, 40.05, 39.62, 30.95, 29.24, 26.27, 25.96, 24.67.

HRMS (ESI-QTOF) m/z: $[M + H]^+$ Calcd for $C_{32}H_{42}N_6O_8S$: 671.2858; Found 671.2840

Compound **49 (PULI$_{560}$-M-Halo)**

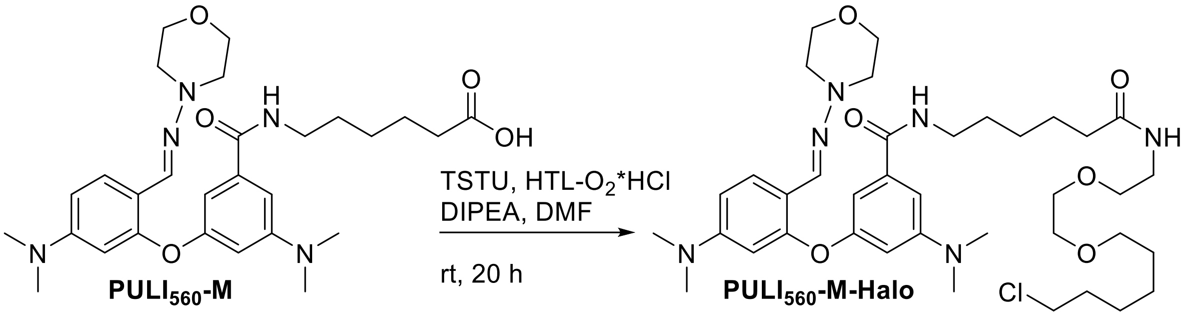

PULI$_{560}$-M (10 mg, 1 eq), TSTU (N,N,N′,N′-Tetramethyl-O-(N-succinimidyl)uroniumtetrafluoroborat, 8.6 mg, 1.5 eq), DIPEA (N,N-Diisopropylethylamine, 7.4 mg, 10 µL, 3 eq) and HaloTag ligand (2-(2-((6-Chlorohexyl)oxy)ethoxy)ethan-1-amine hydrochloride) (7.4 mg, 1.5 eq) was dissolved in 1 mL of dry DMF in a closed cap vial and stirred at room temperature for 20 hours. Then the compound was diluted with acetonitrile and purified with preparative HPLC (CH_3CN:H_2O 10-80% in 30 minutes). The final product was then passed through a short silica plug with acetone to remove red contaminants to give 9 mg (65 %) of colourless product.

^{1}H NMR (400 MHz, CD_3CN) δ 7.73 (d, *J* = 8.8 Hz, 1H), 7.71 (s, 1H), 7.01 (t, *J* = 5.9 Hz, 1H), 6.81 (t, *J* = 1.9 Hz, 1H), 6.60 (dd, *J* = 8.8, 2.6 Hz, 1H), 6.51 (t, *J* = 1.8 Hz, 1H), 6.47 (t, *J* = 2.3 Hz, 1H), 6.41 (s, 1H), 6.27 (d, *J* = 2.6 Hz, 1H), 3.77 – 3.65 (m, 4H), 3.56 (t, *J* = 6.7 Hz, 2H), 3.53 – 3.45 (m, 4H), 3.45 – 3.37 (m, 4H), 3.26 (p, *J* = 6.2, 5.7 Hz, 4H), 2.98 – 2.91 (m, 10H), 2.90 (s, 6H), 2.10 (t, *J* = 7.4 Hz, 2H), 1.78 – 1.69 (m, 2H), 1.59 – 1.49 (m, 6H), 1.45 – 1.36 (m, 2H), 1.36 – 1.28 (m, 4H).

^{13}C NMR (101 MHz, CD_3CN) δ 173.63, 167.81, 160.59, 155.84, 153.08, 153.06, 138.13, 133.04, 127.35, 116.98, 110.13, 106.35, 104.68, 104.48, 104.30, 71.63, 70.93, 70.76, 70.31, 67.01, 53.10, 46.22, 40.75, 40.50, 40.21, 39.77, 36.69, 33.34, 30.33, 29.91, 27.40, 27.18, 26.19, 26.06.

HRMS (ESI-QTOF) m/z: $[M + H]^+$ Calcd for $C_{38}H_{59}ClN_6O_6$: 731.4257; Found 731.4256

Compound **50 (PULI$_{560}$-P-Halo)**

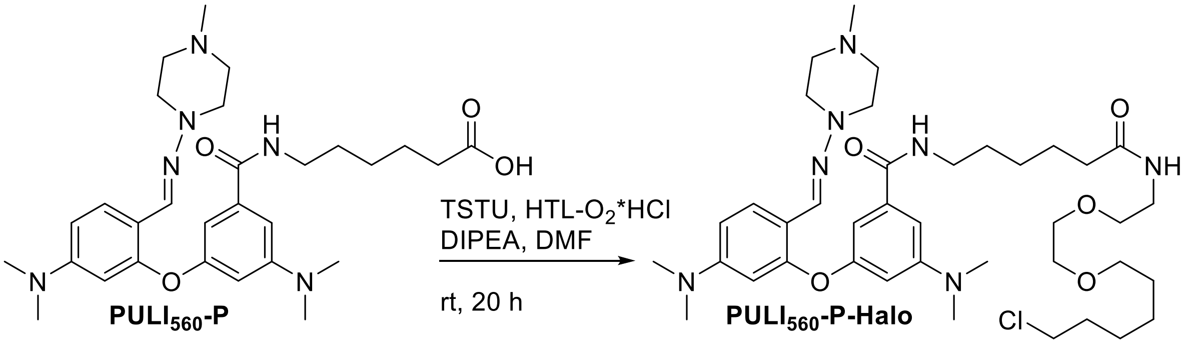

PULI$_{560}$-P (10 mg, 1 eq), TSTU (N,N,N′,N′-Tetramethyl-O-(N-succinimidyl)uroniumtetrafluoroborat, 8.4 mg, 1.5 eq), DIPEA (N,N-Diisopropylethylamine, 7.2 mg, 9.7 µL, 3 eq) and HaloTag ligand (2-(2-((6-Chlorohexyl)oxy)ethoxy)ethan-1-amine hydrochloride) (7.2 mg, 1.5 eq) was dissolved in 1 mL of dry DMF in a closed cap vial and stirred at room temperature for 20 hours. Then the compound was diluted with acetonitrile and purified with preparative HPLC (CH_3CN:H_2O 10-80% in 30 minutes) to give 5 mg (36 %) of colourless product.

^{1}H NMR (400 MHz, CD_3CN) δ 7.72 (d, *J* = 8.9 Hz, 1H), 7.63 (s, 1H), 7.00 (t, *J* = 5.9 Hz, 1H), 6.80 (t, *J* = 1.9 Hz, 1H), 6.60 (dd, *J* = 8.9, 2.6 Hz, 1H), 6.50 (t, *J* = 1.8 Hz, 1H), 6.47 (t, *J* = 2.3 Hz, 1H), 6.41 (s, 1H), 6.28 (d, *J* = 2.6 Hz, 1H), 3.56 (t, *J* = 6.7 Hz, 2H), 3.53 – 3.45 (m, 4H), 3.45 – 3.36 (m, 4H), 3.32 – 3.22 (m, 4H), 2.96 (t, *J* = 5.1 Hz, 4H), 2.93

(s, 6H), 2.90 (s, 6H), 2.47 (t, *J* = 5.1 Hz, 4H), 2.23 (s, 3H), 2.10 (t, *J* = 7.4 Hz, 2H), 1.74 (p, *J* = 7.0 Hz, 2H), 1.60 – 1.50 (m, 6H), 1.43 – 1.36 (m, 2H), 1.36 – 1.30 (m, 4H).

^{13}C NMR (101 MHz, CD_3CN) δ 173.64, 167.83, 160.67, 155.67, 153.06, 152.98, 138.13, 132.75, 127.28, 117.26, 110.18, 106.31, 104.62, 104.23, 71.63, 70.94, 70.76, 70.30, 55.03, 51.98, 46.22, 45.92, 40.75, 40.52, 40.20, 39.78, 36.69, 33.34, 30.33, 29.90, 27.40, 27.17, 26.19, 26.05.

HRMS (ESI-QTOF) m/z: $[M + H]^+$ Calcd for $C_{39}H_{62}ClN_7O_5$: 744.4574; Found 744.4595

Compound **51 (PULI$_{560}$-T-Halo)**

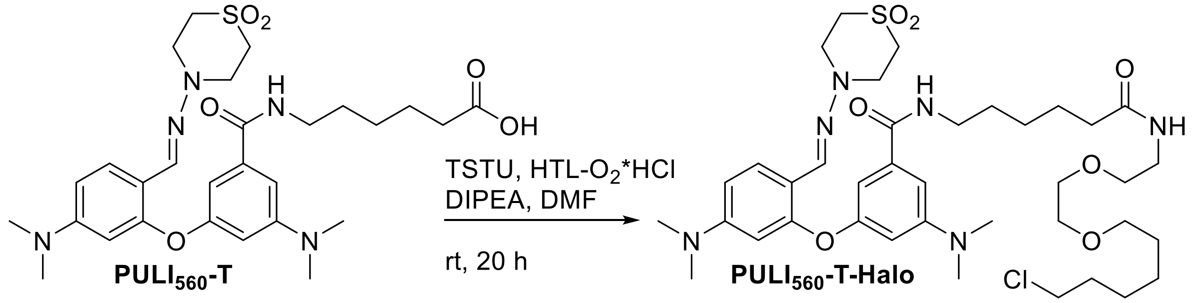

PULI$_{560}$-T (6.5 mg, 1 eq), TSTU (N,N,N′,N′-Tetramethyl-O-(N-succinimidyl)uroniumtetrafluoroborat, 5.1 mg, 1.5 eq), DIPEA (N,N-Diisopropylethylamine, 2.2 mg, 3.0 µL, 3 eq) and HaloTag ligand (2-(2-((6-Chlorohexyl)oxy)ethoxy)ethan-1-amine hydrochloride) (4.4 mg, 1.5 eq) was dissolved in 1 mL of dry DMF in a closed cap vial and stirred at room temperature for 20 hours. Then the compound was diluted with acetonitrile and purified with preparative HPLC (CH_3CN:H_2O 10-80% in 30 minutes) to give 4 mg (45 %) of colourless product.

^{1}H NMR (400 MHz, CD_3CN) δ 7.74 – 7.65 (m, 2H), 6.98 (t, *J* = 5.8 Hz, 1H), 6.76 (t, *J* = 1.9 Hz, 1H), 6.63 (dd, *J* = 8.9, 2.6 Hz, 1H), 6.51 – 6.41 (m, 2H), 6.39 (s, 1H), 6.34 (d, *J* = 2.6 Hz, 1H), 3.66 (t, *J* = 5.4 Hz, 4H), 3.57 (t, *J* = 6.7 Hz, 2H), 3.55 – 3.44 (m, 5H), 3.47 – 3.35 (m, 4H), 3.31 – 3.22 (m, 4H), 2.76 (t, *J* = 5.2 Hz, 5H), 2.10 (t, *J* = 7.4 Hz, 4H), 1.80 – 1.68 (m, 2H), 1.61 – 1.49 (m, 6H), 1.45 – 1.38 (m, 2H), 1.36 – 1.29 (m, 4H).

^{13}C NMR (101 MHz, CD_3CN) δ 173.64, 167.95, 160.84, 155.71, 153.46, 153.09, 138.36, 137.56, 128.05, 116.43, 110.12, 106.27, 104.84, 104.12, 103.89, 71.63, 70.94, 70.76, 70.30, 49.72, 48.63, 46.23, 40.75, 40.48, 40.23, 39.77, 36.70, 33.34, 30.33, 29.90, 27.39, 27.19, 26.18, 26.07.

HRMS (ESI-QTOF) m/z: $[M + H]^+$ Calcd for $C_{38}H_{59}ClN_6O_7S$: 801.3747; Found 801.3732

Compound **PULI$_{560}$-M-Snap**

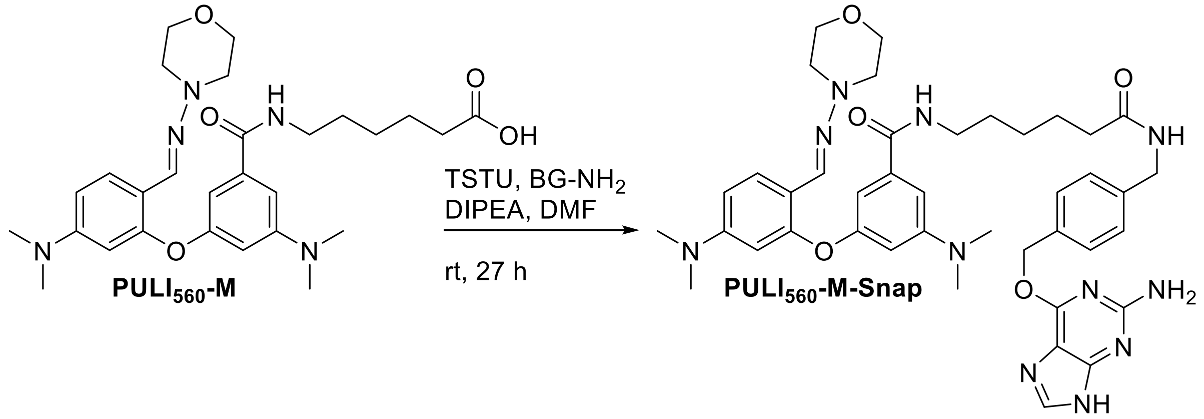

PULI$_{560}$-M (10 mg, 1 eq), TSTU (N,N,N′,N′-Tetramethyl-O-(N-succinimidyl)uroniumtetrafluoroborat, 8.6 mg, 1.5 eq), DIPEA (N,N-Diisopropylethylamine, 7.4 mg, 10 µL, 3 eq) and BG-NH_2 (O6-(4-Aminomethyl-benzyl)guanine) (7.7 mg, 1.5 eq) was dissolved in 1 mL of dry DMF in a closed cap vial

and stirred at room temperature for 22 hours. Next day another 7.7 mg of BG-NH2 was added and the reaction mixture was stirred for another 5 hours, when full conversion was verified by LC-MS. Then the compound was diluted with acetonitrile and purified with preparative HPLC (CH_3CN:H_2O 10-80% in 30 minutes) to give 12 mg (81 %) of colourless product.

^{1}H NMR (400 MHz, $CDCl_3$) δ 7.84 – 7.73 (m, 2H), 7.34 (d, *J* = 7.7 Hz, 2H), 7.25 (d, *J* = 7.5 Hz, 2H), 6.93 – 6.82 (m, 1H), 6.65 – 6.52 (m, 2H), 6.49 (dd, *J* = 9.0, 2.6 Hz, 1H), 6.42 (t, *J* = 2.3 Hz, 1H), 6.16 (d, *J* = 2.6 Hz, 1H), 5.30 (s, 2H), 5.04 (s, 2H), 4.41 (d, *J* = 5.2 Hz, 2H), 3.85 – 3.69 (m, 4H), 3.31 (q, *J* = 6.6 Hz, 2H), 3.05 – 2.98 (m, 4H), 2.90 (s, 6H), 2.86 (s, 6H), 2.32 (t, *J* = 7.3 Hz, 2H), 1.67 (p, *J* = 7.4 Hz, 2H), 1.53 (p, *J* = 7.3 Hz, 2H), 1.35 (p, *J* = 7.9 Hz, 2H).

^{13}C NMR (101 MHz, $CDCl_3$) δ 168.19, 159.48, 159.33, 155.26, 152.09, 152.03, 139.52, 137.04, 135.10, 133.91, 129.93, 126.81, 115.87, 109.12, 106.18, 104.41, 103.53, 103.04, 68.71, 66.62, 52.50, 43.80, 40.58, 40.41, 39.89, 36.22, 31.07, 29.83, 29.41, 29.17, 26.51, 25.30.

HRMS (ESI-QTOF) m/z: $[M + H]^+$ Calcd for $C_{41}H_{51}ClN_{11}O_5$: 778.4148; Found 778.4138

Compound **PULI$_{560}$-P-Snap**

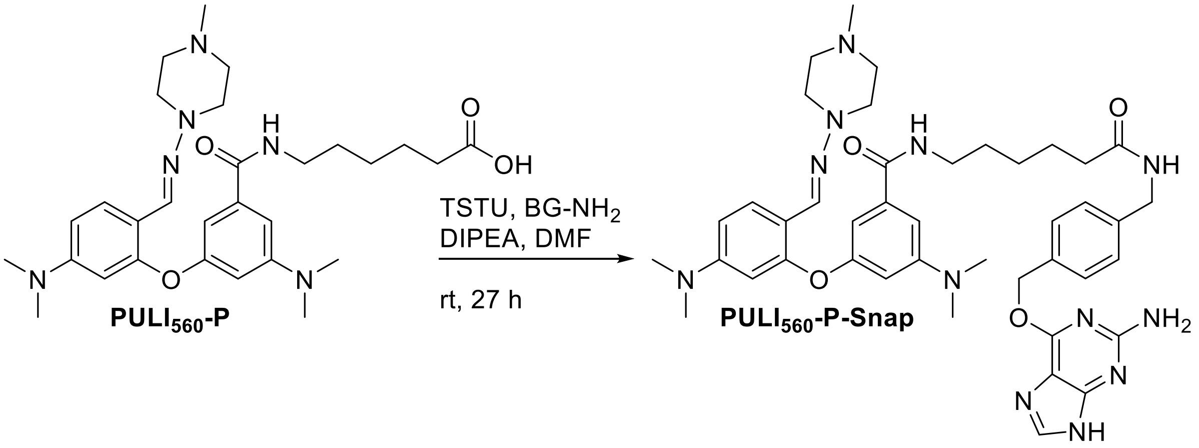

PULI$_{560}$-P (10 mg, 1 eq), TSTU (N,N,N′,N′-Tetramethyl-O-(N-succinimidyl)uroniumtetrafluoroborat, 8.4 mg, 1.5 eq), DIPEA (N,N-Diisopropylethylamine, 7.2 mg, 9.7 µL, 3 eq) and BG-NH_2 (O6-(4-Aminomethyl-benzyl)guanine) (7.5 mg, 1.5 eq) was dissolved in 1 mL of dry DMF in a closed cap vial and stirred at room temperature for 22 hours. Next day another 7.5 mg of BG-NH2 was added and the reaction mixture was stirred for another 5 hours, when full conversion was verified by LC-MS. Then the compound was diluted with acetonitrile and purified with preparative HPLC (CH_3CN:H_2O 10-80% in 30 minutes) to give 7 mg (48 %) of colourless product.

^{1}H NMR (400 MHz, $CDCl_3$) δ 7.80 (d, *J* = 8.8 Hz, 1H), 7.76 (s, 1H), 7.42 (s, 1H), 7.35 (d, *J* = 7.7 Hz, 2H), 7.26 – 7.23 (m, 2H), 6.88 (t, *J* = 1.9 Hz, 1H), 6.69 (s, 1H), 6.60 – 6.51 (m, 2H), 6.49 (dd, *J* = 8.9, 2.6 Hz, 1H), 6.42 (t, *J* = 2.2 Hz, 1H), 6.16 (d, *J* = 2.5 Hz, 1H), 5.31 (s, 2H), 4.99 (s, 2H), 4.42 (d, *J* = 5.2 Hz, 2H), 3.30 (q, *J* = 6.6 Hz, 2H), 3.07 (t, *J* = 5.1 Hz, 4H), 2.90 (s, 6H), 2.86 (s, 6H), 2.55 (t, *J* = 5.1 Hz, 4H), 2.38 – 2.26 (m, 5H), 1.67 (p, *J* = 7.5 Hz, 2H), 1.53 (p, *J* = 7.3 Hz, 2H), 1.41 – 1.29 (m, 2H).

^{13}C NMR (101 MHz, $CDCl_3$) δ 174.02, 168.22, 159.46, 159.37, 155.14, 152.02, 151.99, 139.49, 137.04, 135.14, 133.69, 129.90, 128.31, 126.77, 116.15, 109.16, 106.18, 104.40, 103.48, 103.15, 68.64, 54.53, 51.44, 45.86, 43.76, 40.59, 40.42, 39.85, 36.23, 31.07, 29.15, 26.48, 25.29.

HRMS (ESI-QTOF) m/z: $[M + H]^+$ Calcd for $C_{42}H_{54}N_{12}O_4$: 791.4464; Found 791.4488

Compound **PULI$_{560}$-M-maleimide**

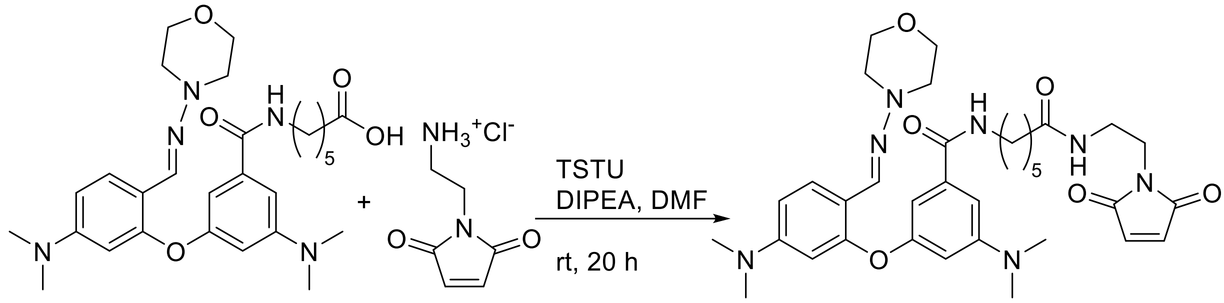

PULI$_{560}$-M **PULI$_{560}$-M-maleimide**

PULI$_{560}$-M (10 mg, 1 eq), TSTU (N,N,N′,N′-Tetramethyl-O-(N-succinimidyl)uroniumtetrafluoroborat, 8.6 mg, 1.5 eq), DIPEA (N,N-Diisopropylethylamine, 7.4 mg, 10 µL, 3 eq) and 2-Maleimidoethylamine hydrochloride (5.0 mg, 1.5 eq) was dissolved in 1 mL of dry DMF in a closed cap vial and stirred at room temperature for 20 hours. Then the compound was diluted with acetonitrile and purified with preparative HPLC (CH_3CN:H_2O 10-80% in 30 minutes). The final product was then passed through a short silica plug with acetone to remove red contaminants to give 7 mg (57 %) of pale yellow product.

^{1}H NMR (400 MHz, CD_3CN) δ 7.72 (d, *J* = 8.9 Hz, 1H), 7.70 (s, 1H), 7.01 (t, *J* = 5.9 Hz, 1H), 6.81 (t, *J* = 1.8 Hz, 1H), 6.72 (s, 2H), 6.60 (dd, *J* = 8.9, 2.6 Hz, 1H), 6.51 (t, *J* = 1.8 Hz, 1H), 6.47 (t, *J* = 2.3 Hz, 1H), 6.41 (s, 1H), 6.27 (d, *J* = 2.6 Hz, 1H), 3.76 – 3.64 (m, 4H), 3.48 (dd, *J* = 6.6, 5.0 Hz, 2H), 3.30 – 3.22 (m, 4H), 2.95 – 2.91 (m, 10H), 2.90 (s, 6H), 2.02 (t, *J* = 7.3 Hz, 2H), 1.51 (p, *J* = 7.3 Hz, 4H), 1.30 – 1.26 (m, 2H).

^{13}C NMR (101 MHz, CD_3CN) δ 173.94, 172.11, 167.82, 160.60, 155.83, 153.07, 153.05, 138.11, 135.23, 133.04, 127.34, 116.97, 110.12, 106.36, 104.68, 104.50, 104.25, 67.00, 53.09, 40.74, 40.49, 40.23, 38.36, 38.15, 36.63, 29.91, 27.17, 25.90.

HRMS (ESI-QTOF) m/z: [M + H]$^+$ Calcd for $C_{34}H_{45}N_7O_6$: 648.3504; Found 648.3508

Compound **PULI$_{560}$-P-maleimide**

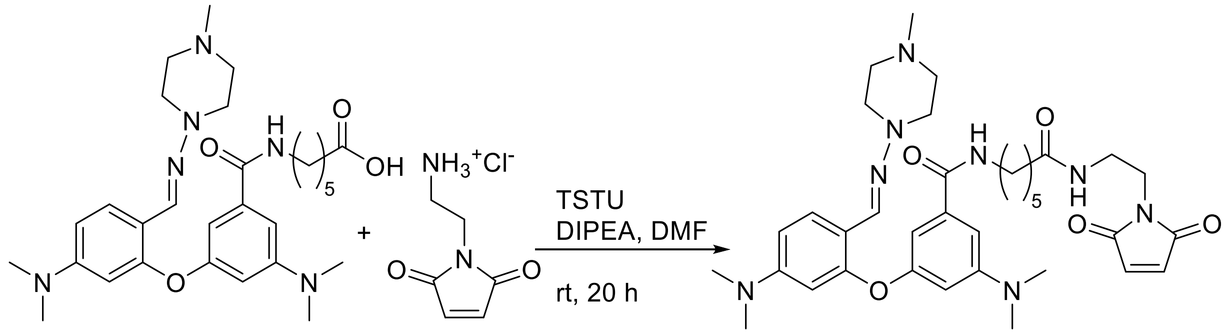

PULI$_{560}$-P **PULI$_{560}$-P-maleimide**

PULI$_{560}$-P (10 mg, 1 eq), TSTU (N,N,N′,N′-Tetramethyl-O-(N-succinimidyl)uroniumtetrafluoroborat, 8.4 mg, 1.5 eq), DIPEA (N,N-Diisopropylethylamine, 7.2 mg, 9.7 µL, 3 eq) and 2-Maleimidoethylamine hydrochloride (4.9 mg, 1.5 eq) was dissolved in 1 mL of dry DMF in a closed cap vial and stirred at room temperature for 20 hours. Then the compound was diluted with acetonitrile and purified with preparative HPLC (CH_3CN:H_2O 10-80% in 30 minutes). The final product was then passed through a short silica plug with acetone to remove red contaminants to give 5 mg (41 %) of pale yellow product.

^{1}H NMR (400 MHz, CD_3CN) δ 7.71 (d, *J* = 8.8 Hz, 1H), 7.62 (s, 1H), 7.01 (t, *J* = 5.9 Hz, 1H), 6.80 (t, *J* = 1.9 Hz, 1H), 6.72 (s, 2H), 6.60 (dd, *J* = 8.9, 2.6 Hz, 1H), 6.50 (t, *J* = 1.8 Hz, 1H), 6.46 (t, *J* = 2.3 Hz, 1H), 6.43 (s, 1H), 6.27 (d, *J* = 2.6 Hz, 1H), 3.48 (dd, *J* = 6.5, 5.0 Hz, 2H), 3.30 – 3.22 (m, 4H), 2.95 (t, *J* = 5.0 Hz, 4H), 2.92 (s, 6H), 2.89 (s, 6H), 2.45 (t, *J* = 5.1 Hz, 4H), 2.22 (s, 3H), 2.02 (t, *J* = 7.3 Hz, 2H), 1.51 (p, *J* = 7.3 Hz, 4H), 1.31 – 1.24 (m, 2H).

^{13}C NMR (101 MHz, CD_3CN) δ 173.94, 172.11, 167.83, 160.67, 155.65, 153.05, 152.95, 138.10, 135.24, 132.62, 127.26, 117.29, 110.18, 106.32, 104.63, 104.61, 104.19, 55.09, 52.06, 46.00, 40.74, 40.51, 40.23, 38.36, 38.15, 36.63, 29.91, 27.17, 25.90.

HRMS (ESI-QTOF) m/z: $[M + H]^+$ Calcd for $C_{35}H_{48}N_8O_5$: 661.3820; Found 661.3822

Compound **PULI$_{560}$-T-maleimide**

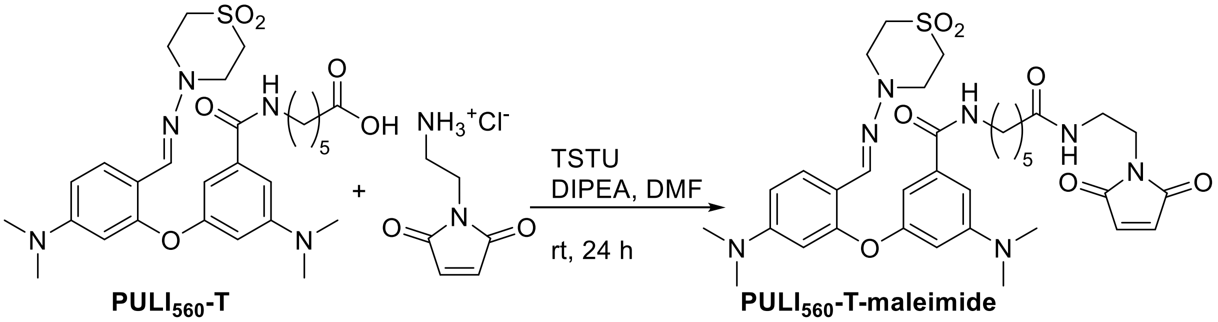

PULI$_{560}$-T **PULI$_{560}$-T-maleimide**

PULI$_{560}$-T (5 mg, 1 eq), TSTU (N,N,N′,N′-Tetramethyl-O-(N-succinimidyl)uroniumtetrafluoroborat, 3.9 mg, 1.5 eq), DIPEA (N,N-Diisopropylethylamine, 3.4 mg, 4.6 µL, 3 eq) and 2-Maleimidoethylamine hydrochloride (2.3 mg, 1.5 eq) was dissolved in 1 mL of dry DMF in a closed cap vial and stirred at room temperature for 24 hours. Then the compound was diluted with acetonitrile and purified with preparative HPLC (CH_3CN:H_2O 10-80% in 30 minutes). The final product was then passed through a short silica plug with ethyl acetate to remove pink contaminants to give 2.7 mg (45 %) of pale yellow product.

^1H NMR (400 MHz, CD_3CN) δ 7.72 – 7.65 (m, 2H), 6.99 (t, J = 5.2 Hz, 1H), 6.76 (dd, J = 2.4, 1.4 Hz, 1H), 6.72 (s, 2H), 6.62 (dd, J = 8.9, 2.6 Hz, 1H), 6.48 – 6.38 (m, 3H), 6.34 (d, J = 2.6 Hz, 1H), 3.71 – 3.59 (m, 4H), 3.52 – 3.44 (m, 2H), 3.32 – 3.20 (m, 4H), 2.93 (s, 6H), 2.92 (s, 6H), 2.80 – 2.73 (m, 4H), 2.02 (t, J = 7.4 Hz, 2H), 1.57 – 1.44 (m, 4H), 1.32 – 1.27 (m, 2H).

^{13}C NMR (101 MHz, CD_3CN) δ 173.98, 172.13, 167.98, 160.84, 155.70, 153.46, 153.08, 138.32, 137.57, 135.25, 128.04, 116.43, 110.12, 106.28, 104.85, 104.13, 103.86, 49.71, 48.62, 40.74, 40.48, 40.24, 38.35, 38.16, 36.63, 29.89, 27.17, 25.90.

HRMS (ESI-QTOF) m/z: $[M + H]^+$ Calcd for $C_{34}H_{45}N_7O_7S$: 696.3174; Found 696.3173

Compound **PULI$_{560}$-T-cabazitaxel**

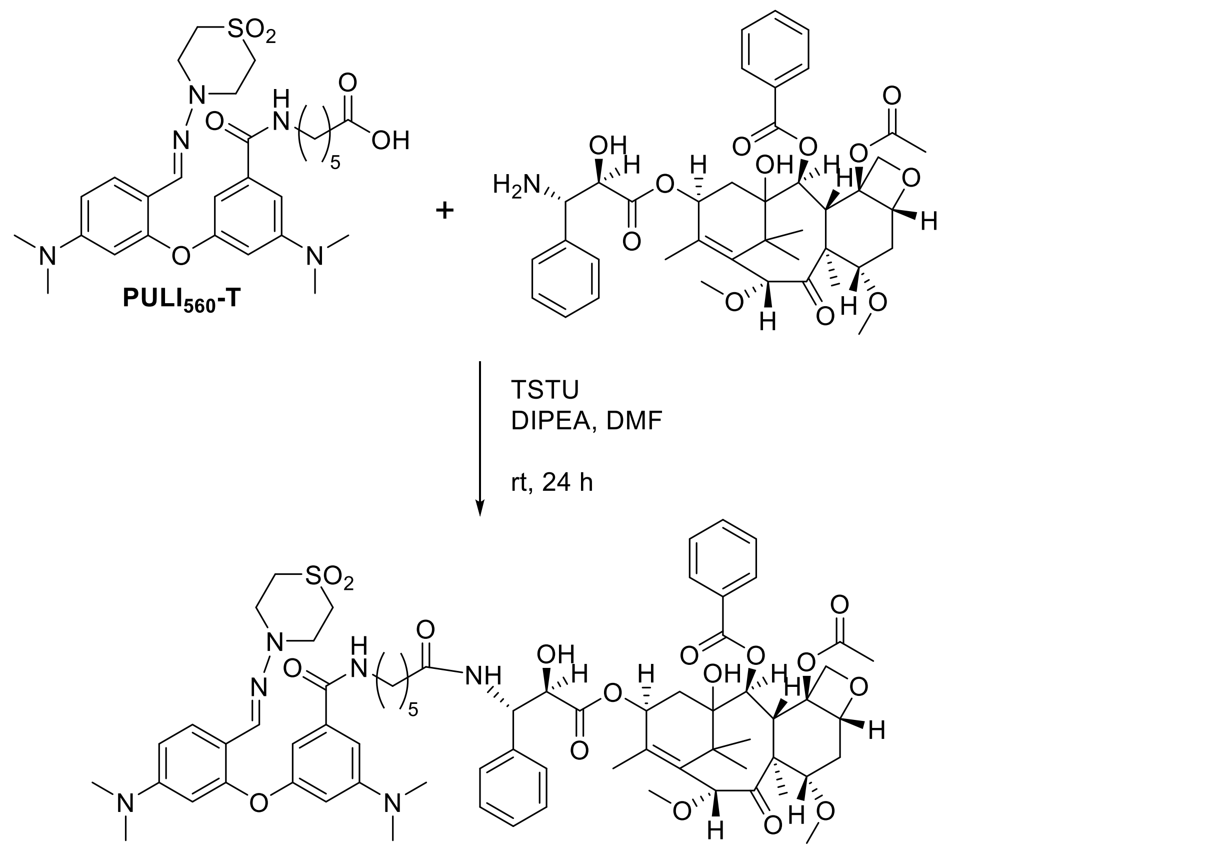

PULI$_{560}$-T (5 mg, 1 eq), TSTU (N,N,N′,N′-Tetramethyl-O-(N-succinimidyl)uroniumtetrafluoroborat, 3.9 mg, 1.5 eq), DIPEA (N,N-Diisopropylethylamine, 3.4 mg, 4.6 µL, 3 eq) and Boc-deprotected Cabazitaxel (deprotection was carried out following literature procedure[90])(9.6 mg, 1.5 eq) was dissolved in 1 mL of dry DMF in a closed cap vial and stirred at room temperature for 24 hours. Then the compound was diluted with acetonitrile and purified with preparative HPLC (CH_3CN:H_2O 10-80% in 30 minutes). The final product was then passed through a short silica plug with ethyl acetate to remove pink contaminants to give 2.7 mg (24 %) of pale yellow product.

^{1}H NMR (400 MHz, CD_3CN) δ 8.15 – 8.05 (m, 2H), 7.72 – 7.66 (m, 2H), 7.66 – 7.61 (m, 1H), 7.58 – 7.51 (m, 2H), 7.38 (d, *J* = 4.4 Hz, 4H), 7.33 – 7.23 (m, 1H), 7.07 (d, *J* = 9.0 Hz, 1H), 7.00 – 6.90 (m, 1H), 6.74 – 6.69 (m, 1H), 6.62 (dd, *J* = 8.8, 2.6 Hz, 1H), 6.46 – 6.39 (m, 2H), 6.33 (d, *J* = 2.6 Hz, 1H), 6.19 – 6.10 (m, 1H), 5.53 (d, *J* = 7.2 Hz, 1H), 5.48 (dd, *J* = 9.1, 3.5 Hz, 1H), 4.96 (dd, *J* = 9.8, 2.1 Hz, 1H), 4.79 (s, 1H), 4.62 (dd, *J* = 6.0, 3.5 Hz, 1H), 4.13 (s, 2H), 4.06 (d, *J* = 5.9 Hz, 1H), 3.86 (dd, *J* = 10.7, 6.5 Hz, 1H), 3.77 (d, *J* = 7.2 Hz, 1H), 3.68 – 3.58 (m, 4H), 3.36 (s, 3H), 3.25 (s, 3H), 3.20 (s, 1H), 3.17 – 3.06 (m, 1H), 2.92 (s, 6H), 2.91 (s, 6H), 2.79 – 2.73 (m, 4H), 2.72 – 2.63 (m, 1H), 2.36 (s, 3H), 2.23 – 2.19 (m, 3H), 1.96 (s, 4H), 1.61 (s, 4H), 1.58 – 1.51 (m, 2H), 1.49 – 1.36 (m, 2H), 1.12 (s, 3H), 1.05 (s, 3H).

^{13}C NMR (101 MHz, CD_3CN) δ 173.77, 173.71, 171.50, 168.19, 166.87, 160.85, 155.67, 153.46, 153.08, 140.33, 140.05, 138.13, 137.64, 136.19, 134.35, 131.17, 130.99, 129.68, 129.46, 128.50, 128.08, 116.38, 110.12, 106.27, 104.85, 104.17, 103.86, 84.77, 83.43, 82.03, 81.71, 79.01, 76.97, 75.67, 74.66, 72.42, 57.58, 57.44, 57.39, 55.87, 49.70, 48.59, 48.10, 44.28, 40.74, 40.48, 40.00, 36.81, 36.56, 32.88, 29.89, 27.44, 27.00, 26.07, 23.23, 22.02, 14.89, 11.04.

HRMS (ESI-QTOF) m/z: $[M + H]^+$ Calcd for $C_{68}H_{86}N_6O_{17}S$: 1291.5843; Found 1291.5846

Compound **SI-14**

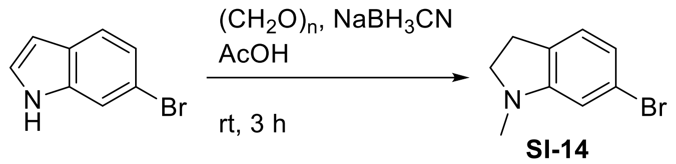

The reaction was made based on literature procedure.[91] 6-Bromoindole (1.013 g, 1 eq), paraformaldehyde (1.55 g, 10 eq) and $NaBH_3CN$ (1.628 g, 5 eq) were dissolved in 10 mL of acetic acid and the mixture was stirred for 3 hours. The reaction mixture was quenched by adding 1 M NaOH solution, then washed with dichloromethane (3*20 mL), the combined organic layers dried over Na_2SO_4 and evaporated. The product was purified with flash column chromatography (hexane:ethyl acetate 0 to 10% in 10 CV) to recover 180 mg of 6-bromoindole and give 303 mg (28%) of product.

^{1}H NMR (400 MHz, $CDCl_3$) δ 6.90 (dt, *J* = 7.6, 1.2 Hz, 1H), 6.76 (dd, *J* = 7.6, 1.8 Hz, 1H), 6.56 (d, *J* = 1.8 Hz, 1H), 3.34 (t, *J* = 8.2 Hz, 2H), 2.89 (td, *J* = 8.2, 1.2 Hz, 2H), 2.74 (s, 3H).

^{13}C NMR (101 MHz, $CDCl_3$) δ 154.72, 129.48, 125.42, 121.21, 120.41, 110.34, 56.27, 35.91, 28.36.

HRMS (ESI-QTOF) m/z: $[M + H]^+$ Calcd for $C_9H_{10}BrN$: 212.0069; Found 212.0070

Compound **SI-15**

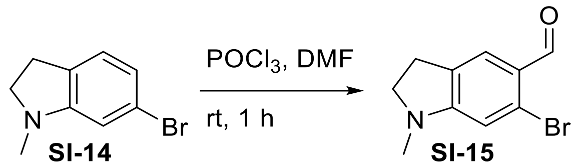

Dimethylformamide (2 ml) and $POCl_3$ (1 mL) was mixed slowly at room temperature and stirred for 10 minutes, then compound **SI-14** (145 mg) was added to the solution. The solution was stirred for 1 hour, poured on water and waited until a precipitate formed. The precipitate was filtered, washed with water and dried to yield 155 mg (94 %) of purple powder as product.

^{1}H NMR (400 MHz, $CDCl_3$) δ 10.04 (s, 1H), 7.57 (d, *J* = 1.5 Hz, 1H), 6.46 (s, 1H), 3.58 (t, *J* = 8.5 Hz, 2H), 2.98 (td, *J* = 8.6, 1.4 Hz, 2H), 2.87 (s, 3H).

^{13}C NMR (101 MHz, $CDCl_3$) δ 190.31, 158.16, 130.48, 129.96, 124.89, 123.08, 108.86, 54.98, 33.85, 27.08.

HRMS (ESI-QTOF) m/z: $[M + H]^+$ Calcd for $C_{10}H_{10}BrNO$: 240.0019; Found 240.0023

Compound **40**

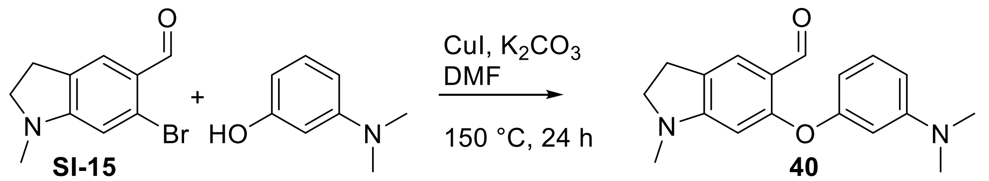

Compound **SI-15** (130 mg, 1 eq), 3-Dimethylaminophenol (82 mg, 1.1 eq), CuI (52 mg, 0.5 eq) and K_2CO_3 (150 mg, 2 eq) was placed in a flame dried flask filled with Argon. The components were dissolved in 5 mL of dry Dimethylformamide and stirred under Argon for 24 hours at 150 °C. After 24 hours, according to LC-MS, most starting materials were consumed, so the mixture was poured on water, filtered through Cellite, washed with water and ethyl acetate to clear it from copper salts. The supernatant was diluted with ethyl acetate (to 30 mL) and washed with water (3*30 mL), the organic phase dried over Na_2SO_4 and evaporated. The product was purified with flash column chromatography (hexane:ethyl acetate 0 to 20% in 10 CV) to give 24 mg (15 %) of yellow oil.

^{1}H NMR (400 MHz, CD_3CN) δ 10.02 (s, 1H), 7.44 (t, *J* = 1.5 Hz, 1H), 7.16 (t, *J* = 8.2 Hz, 1H), 6.52 (dd, *J* = 8.4, 2.5 Hz, 1H), 6.44 (t, *J* = 2.4 Hz, 1H), 6.28 (dd, *J* = 8.0, 2.3 Hz, 1H), 5.82 (s, 1H), 3.52 (t, *J* = 8.4 Hz, 2H), 2.95 (td, *J* = 8.4, 1.4 Hz, 2H), 2.90 (s, 6H), 2.72 (s, 3H).

^{13}C NMR (101 MHz, CD_3CN) δ 186.76, 163.92, 160.14, 159.44, 153.43, 131.06, 127.11, 123.23, 117.60, 108.91, 107.01, 103.76, 95.69, 55.64, 40.65, 33.97, 27.36.

HRMS (ESI-QTOF) m/z: $[M + H]^+$ Calcd for $C_{18}H_{20}N_2O_2$: 297.1598; Found 297.1600

Compound **42**

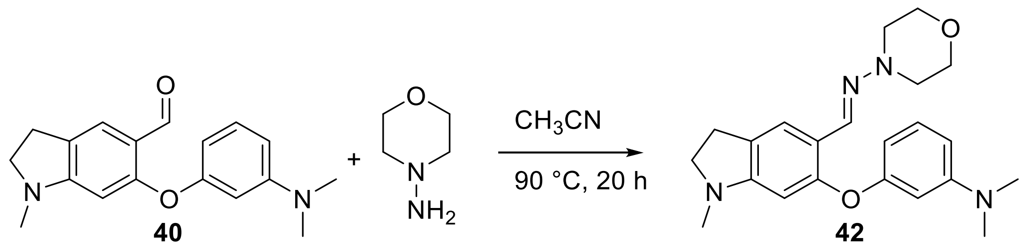

Compound **40** (11 mg, 1 eq) and 4-Aminomorpholine (38 mg, 36 µL, 10 eq) was dissolved in 1 mL of acetonitrile in a sealed screw cap vial and the solution was heated at 90 °C for 20 hours. Next day the solution was diluted with ethyl acetate (10 mL) and washed with water (3*10 mL), the organic layer dried over Na_2SO_4 and evaporated to give 13 mg (92 %) of product.

^{1}H NMR (400 MHz, CD_3CN) δ 7.74 (s, 1H), 7.55 (t, *J* = 1.4 Hz, 1H), 7.10 (t, *J* = 8.2 Hz, 1H), 6.44 (dd, *J* = 8.3, 2.4 Hz, 1H), 6.35 (t, *J* = 2.4 Hz, 1H), 6.14 (dd, *J* = 8.1, 1.9 Hz, 1H), 6.01 (s, 1H), 3.73 – 3.68 (m, 4H), 3.35 (t, *J* = 8.2 Hz, 2H), 2.94 – 2.89 (m, 6H), 2.87 (s, 6H), 2.66 (s, 3H).

^{13}C NMR (101 MHz, CD_3CN) δ 160.67, 156.07, 155.69, 153.36, 133.85, 130.86, 128.07, 121.49, 117.67, 107.96, 105.74, 102.53, 99.44, 67.02, 56.58, 53.20, 40.69, 35.51, 28.45.

HRMS (ESI-QTOF) m/z: $[M + H]^+$ Calcd for $C_{22}H_{28}N_4O_2$: 281.2285; Found 281.2286

Compound **43**

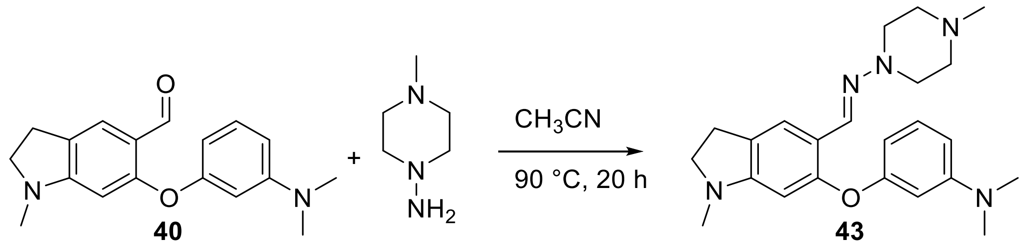

Compound **40** (11 mg, 1 eq) and 1-Amino-4-methylpiperazin (43 mg, 45 µL, 10 eq) was dissolved in 1 mL of acetonitrile in a sealed screw cap vial and the solution was heated at 90 °C for 20 hours. Next day the solution was diluted with ethyl acetate (10 mL) and washed with water (3*10 mL), the organic layer dried over Na_2SO_4 and evaporated to give 12 mg (82 %) of product.

^{1}H NMR (400 MHz, CD_3CN) δ 7.39 (s, 1H), 7.28 (t, *J* = 1.4 Hz, 1H), 6.82 (t, *J* = 8.2 Hz, 1H), 6.16 (ddd, *J* = 8.4, 2.5, 0.7 Hz, 1H), 6.08 (t, *J* = 2.4 Hz, 1H), 5.87 (ddd, *J* = 8.1, 2.4, 0.8 Hz, 1H), 5.74 (s, 1H), 3.08 (t, *J* = 8.2 Hz, 2H), 2.70 – 2.62 (m, 6H), 2.61 (s, 6H), 2.39 (s, 3H), 2.21 – 2.13 (m, 4H), 1.94 (s, 3H).

^{13}C NMR (101 MHz, CD_3CN) δ 160.73, 155.90, 155.47, 153.36, 133.27, 130.85, 128.08, 121.43, 118.03, 107.91, 105.69, 102.48, 99.57, 56.62, 55.22, 52.25, 46.09, 40.69, 35.57, 28.48.

HRMS (ESI-QTOF) m/z: $[M + H]^+$ Calcd for $C_{23}H_{31}N_5O$: 394.2601; Found 394.2598

Compound **SI-16**

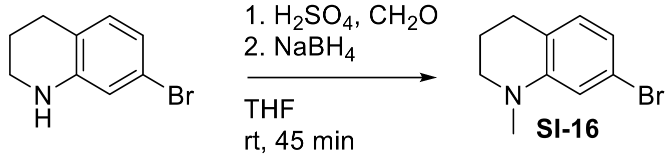

Compound **SI-16** was synthetized based on literature.[89] Aqueous formaldehyde (37%, 1 mL) was added to a mixture of THF (10 mL) and 3 M H_2SO_4 (1 mL) and stirred at 0°C for 10 minutes, then 7-Bromo-1,2,3,4-tetrahydroquinoline (0.5 g) was added and the mixture was stirred for 45 minutes at 0°C. After 45 minutes 250 mg of $NaBH_4$ was slowly added to the flask while still cooling it in an ice bath. The reaction mixture was diluted with ethyl acetate (50 mL) and washed with saturated $NaHCO_3$ solution (3*50 mL), the organic layer dried over Na_2SO_4 and evaporated to give 501 mg (94 %) of yellow oil as product.

^{1}H NMR (400 MHz, $CDCl_3$) δ 6.80 – 6.75 (m, 1H), 6.71 – 6.63 (m, 2H), 3.26 – 3.20 (m, 2H), 2.87 (s, 3H), 2.68 (t, *J* = 6.4 Hz, 2H), 1.99 – 1.89 (m, 2H).

^{13}C NMR (101 MHz, $CDCl_3$) δ 147.83, 129.96, 121.65, 120.75, 118.65, 113.41, 51.02, 39.07, 27.50, 22.21.

HRMS (ESI-QTOF) m/z: $[M + H]^+$ Calcd for $C_{10}H_{12}BrN$: 226.0226; Found 226.0224

Compound **SI-17**

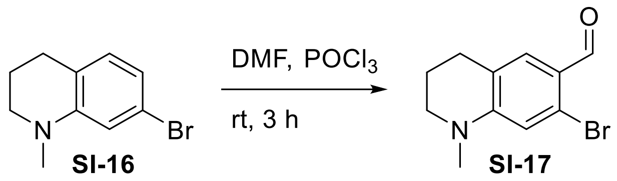

Dimethylformamide (4 ml) and $POCl_3$ (2 mL) was mixed slowly at 0 °C and stirred for 25 minutes, then compound **SI-16** (0.5 g) was added to the solution. The solution was stirred for 3 hours, poured on water and waited until a white precipitate formed. The precipitate was filtered, washed with water and dried to yield 0.35 g (62 %) of pale green powder as product.

^{1}H NMR (400 MHz, $CDCl_3$) δ 10.03 (s, 1H), 7.51 (s, 1H), 6.64 (s, 1H), 3.37 (t, *J* = 5.8 Hz, 2H), 3.00 (s, 3H), 2.71 (t, *J* = 6.3 Hz, 2H), 2.01 – 1.85 (m, 2H).

^{13}C NMR (101 MHz, $CDCl_3$) δ 190.43, 151.66, 129.48, 128.13, 121.74, 121.70, 113.26, 51.15, 38.98, 27.29, 21.49.

HRMS (ESI-QTOF) m/z: $[M + H]^+$ Calcd for $C_{11}H_{12}BrNO$: 254.0175; Found 254.0174

Compound **41**

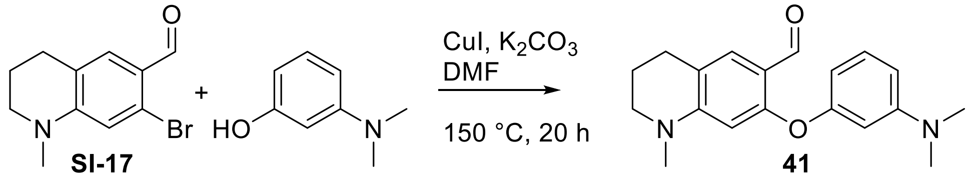

3-Dimethylaminophenol (54 mg, 1 eq), compound **SI-17** (100 mg, 1 eq), CuI (38 mg, 0.5 eq) and K_2CO_3 (109 mg, 2 eq) was placed in a flame dried flask filled with Argon. The components were dissolved in 5 mL of dry Dimethylformamide and stirred under Argon for 20 hours at 150 °C. After 20 hours, according to LC-MS, most starting materials were consumed, so the mixture was poured on water, filtered through Cellite, washed with water and ethyl acetate to clear it from copper salts. The supernatant

was diluted with ethyl acetate (to 30 mL) and washed with water (3*30 mL), the organic phase dried over Na_2SO_4 and evaporated. The product was purified with flash column chromatography (hexane:ethyl acetate 0 to 20% in 10 CV) to give 46 mg (38 %) of yellow oil.

^{1}H NMR (400 MHz, $CDCl_3$) δ 10.12 (s, 1H), 7.51 (s, 1H), 7.15 (t, *J* = 8.1 Hz, 1H), 6.50 – 6.42 (m, 2H), 6.36 – 6.29 (m, 1H), 5.99 (s, 1H), 3.32 (t, *J* = 5.8 Hz, 2H), 2.94 (s, 6H), 2.83 (s, 3H), 2.72 (t, *J* = 6.3 Hz, 2H), 1.94 (p, *J* = 6.1 Hz, 2H).

^{13}C NMR (101 MHz, $CDCl_3$) δ 187.25, 160.79, 158.67, 152.43, 152.19, 130.03, 127.73, 118.08, 116.14, 107.74, 106.21, 102.91, 99.36, 51.07, 40.58, 38.93, 27.09, 21.81.

HRMS (ESI-QTOF) m/z: [M + H]$^+$ Calcd for $C_{19}H_{22}N_2O_2$: 311.1754; Found 311.1755

Compound **44**

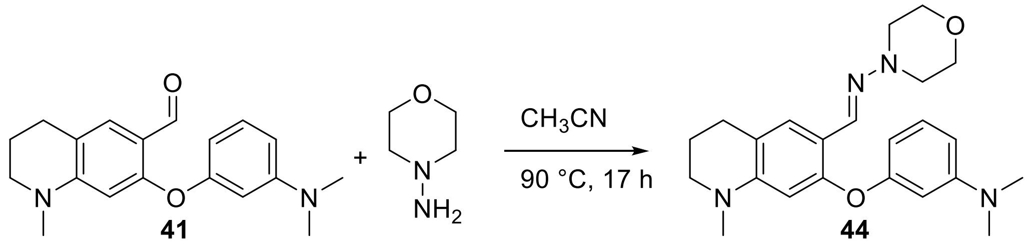

Compound **41** (10 mg, 1 eq) and 4-Aminomorpholine (33 mg, 31 µL, 10 eq) was dissolved in 1 mL of acetonitrile in a sealed screw cap vial and the solution was heated at 90 °C for 17 hours. Next day the solution was diluted with ethyl acetate (10 mL) and washed with water (3*10 mL), the organic layer dried over Na_2SO_4 and evaporated. The product was further purified by passing it through a short plug of silica in hexane-ethyl acetate 7:3 to give 6 mg (47 %) of yellow oil.

^{1}H NMR (400 MHz, CD_3CN) δ 7.69 (s, 1H), 7.43 (s, 1H), 7.09 (t, *J* = 8.2 Hz, 1H), 6.43 (dd, *J* = 8.3, 2.5 Hz, 1H), 6.35 (t, *J* = 2.4 Hz, 1H), 6.15 – 6.08 (m, 2H), 3.74 – 3.68 (m, 4H), 3.24 (t, *J* = 5.7 Hz, 2H), 2.91 – 2.88 (m, 4H), 2.87 (s, 6H), 2.76 (s, 3H), 2.73 (t, *J* = 6.4 Hz, 2H), 1.93 – 1.87 (m, 2H).

^{13}C NMR (101 MHz, CD_3CN) δ 160.83, 154.63, 153.37, 149.09, 133.63, 130.83, 126.07, 120.48, 116.27, 107.84, 105.56, 103.03, 102.39, 67.02, 53.20, 51.43, 40.71, 39.16, 27.97, 23.02.

HRMS (ESI-QTOF) m/z: [M + H]$^+$ Calcd for $C_{23}H_{30}N_4O_2$: 395.2442; Found 395.2445

Compound **45**

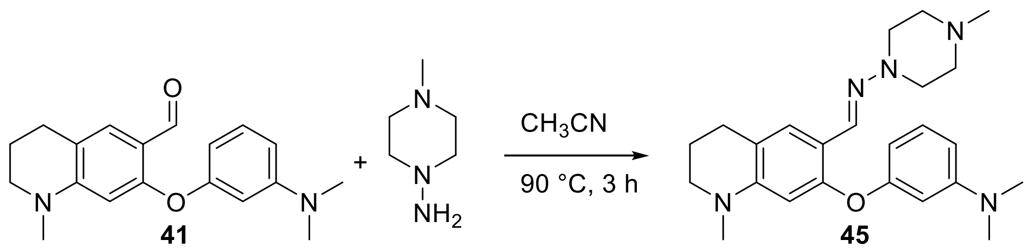

Compound **41** (12 mg, 1 eq) and 1-Amino-4-methylpiperazin (45 mg, 47 µL, 10 eq) was dissolved in 1 mL of acetonitrile in a sealed screw cap vial and the solution was heated at 90 °C for 3 hours. After 3 hours the solution was diluted with ethyl acetate (10 mL) and washed with water (3*10 mL), the organic layer dried over Na_2SO_4 and evaporated to give 11 mg (70 %) of yellow oil.

^{1}H NMR (400 MHz, CD_3CN) δ 7.62 (s, 1H), 7.42 (s, 1H), 7.08 (t, *J* = 8.2 Hz, 1H), 6.42 (dd, *J* = 8.3, 2.5 Hz, 1H), 6.35 (t, *J* = 2.4 Hz, 1H), 6.16 – 6.04 (m, 2H), 3.23 (t, *J* = 5.7 Hz, 2H), 2.92 (t, *J* = 5.1 Hz, 4H), 2.87 (s, 6H), 2.73 (t, *J* = 6.4 Hz, 4H), 2.43 (t, *J* = 5.1 Hz, 4H), 2.21 (s, 3H), 1.94 – 1.86 (m, 2H).

^{13}C NMR (101 MHz, CD_3CN) δ 160.88, 154.44, 153.36, 148.94, 133.07, 130.82, 126.01, 120.50, 116.62, 107.79, 105.51, 103.14, 102.34, 55.19, 52.23, 51.44, 46.06, 40.71, 39.18, 27.97, 23.05.

HRMS (ESI-QTOF) m/z: [M + H]$^+$ Calcd for $C_{24}H_{33}N_5O$: 408.2758; Found 408.2755

Compound **SI-18**

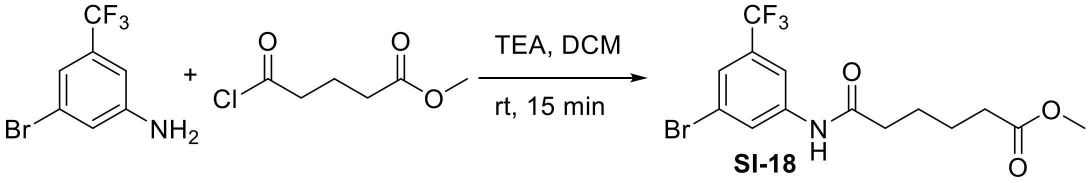

3-Bromo-5-(trifluoromethyl)aniline (500 mg, 295 µL, 1 eq), Methyl adipoyl chloride (372 mg, 324 µL, 1.5 eq) and Triethylamine (421 mg, 580 µL, 2 eq) was dissolved in 10 mL of dichloromethane at room temperature and the mixture was stirred for 15 minutes, then it was washed with water (3*10 mL), the organic phase dried over Na_2SO_4 and evaporated to give 770 mg (97%) of product.

^{1}H NMR (400 MHz, $CDCl_3$) δ 8.13 – 7.98 (m, 2H), 7.77 (s, 1H), 7.46 (s, 1H), 3.70 (s, 3H), 2.46 – 2.35 (m, 4H), 1.76 – 1.66 (m, 4H).

^{13}C NMR (101 MHz, $CDCl_3$) δ 174.60, 171.38, 139.96, 132.80 (q, *J* = 33.1 Hz), 125.71, 123.78 (q, *J* = 3.8 Hz), 123.10 (q, *J* = 273.0 Hz), 123.08, 115.19 (q, *J* = 4.1 Hz), 51.93, 37.09, 33.57, 24.69, 24.09.

HRMS (ESI-QTOF) m/z: $[M + H]^+$ Calcd for $C_{14}H_{15}BrF_3NO_3$: 404.0080; Found 404.0090

Compound **SI-19**

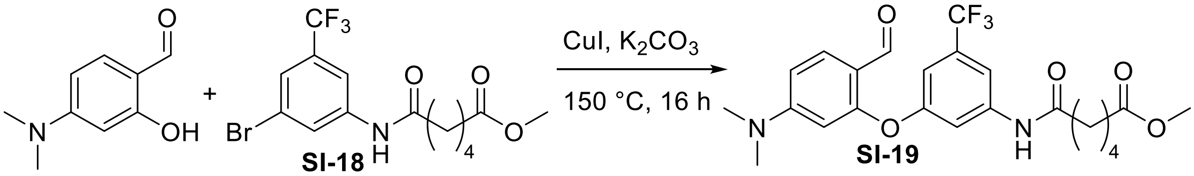

4-(Dimethylamino)salicylaldehyde (144 mg, 1 eq), compound **SI-18** (333 mg, 1 eq), CuI (83 mg, 0.5 eq) and K_2CO_3 (241 mg, 2 eq) was placed in a flame dried flask filled with Argon. The components were dissolved in 5 mL of dry Dimethylformamide and stirred under Argon for 16 hours at 150 °C. After 16 hours, according to LC-MS, most starting materials were consumed, so the mixture was poured on water, filtered through Cellite, washed with water and ethyl acetate to clear it from copper salts. The supernatant was diluted with ethyl acetate (to 30 mL) and washed with water (3*30 mL), the organic phase dried over Na_2SO_4 and evaporated. The product was purified with flash column chromatography (hexane:ethyl acetate 0 to 40% in 10 CV) to give 88 mg (22 %) of yellow oil.

^{1}H NMR (400 MHz, $CDCl_3$) δ 10.06 (s, 1H), 7.83 (dd, *J* = 8.9, 0.9 Hz, 1H), 7.77 (s, 1H), 7.61 (s, 1H), 7.50 (s, 1H), 7.00 (s, 1H), 6.53 (dd, *J* = 8.9, 2.5 Hz, 1H), 6.10 (d, *J* = 2.4 Hz, 1H), 5.30 (d, *J* = 0.9 Hz, 1H), 3.68 (d, *J* = 0.9 Hz, 3H), 3.02 (s, 6H), 2.44 – 2.33 (m, 4H), 1.83 – 1.65 (m, 4H).

^{13}C NMR (101 MHz, $CDCl_3$) δ 186.91, 174.42, 171.15, 160.17, 158.58, 155.90, 140.27, 132.79 (q, *J* = 33.1 Hz), 130.72, 123.52 (d, *J* = 274.1 Hz), 116.73, 112.47, 111.39 (q, *J* = 4.4 Hz), 110.50 (q, *J* = 4.5 Hz), 108.48, 101.32, 51.88, 40.29, 37.20, 33.58, 24.75, 24.14.

HRMS (ESI-QTOF) m/z: $[M + H]^+$ Calcd for $C_{23}H_{35}F_3N_2O_5$: 467.1788; Found 467.1791

Compound **SI-20**

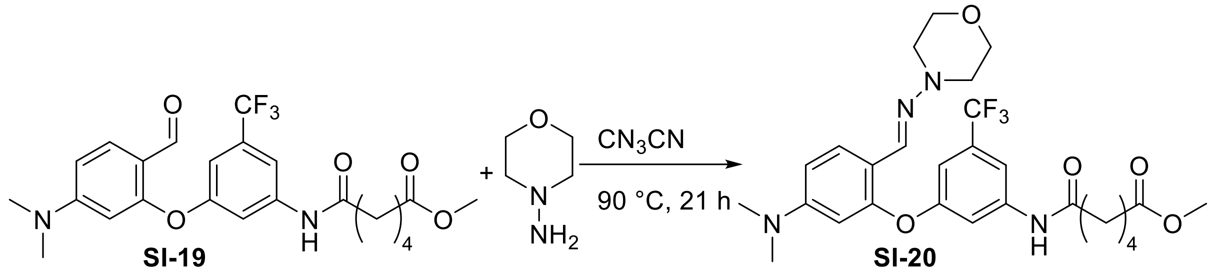

Compound **SI-19** (10 mg, 1 eq) and 4-Aminomorpholine (22 mg, 21 µL, 10 eq) was dissolved in 1 mL of acetonitrile in a sealed screw cap vial and the solution was heated at 90 °C for 21 hours. Next day the solution was diluted with ethyl acetate (10 mL) and washed with water (3*10 mL), the organic layer dried over Na_2SO_4 and evaporated to give 10 mg (85 %) of yellow oil as product.

^1H NMR (400 MHz, CD_3CN) δ 8.46 (s, 1H), 7.74 (d, *J* = 8.9 Hz, 1H), 7.66 – 7.54 (m, 2H), 7.32 (t, *J* = 2.2 Hz, 1H), 6.92 (t, *J* = 2.1 Hz, 1H), 6.67 (dd, *J* = 8.9, 2.6 Hz, 1H), 6.34 (d, *J* = 2.6 Hz, 1H), 3.73 – 3.68 (m, 4H), 3.60 (s, 3H), 2.96 – 2.87 (m, 10H), 2.34 – 2.25 (m, 4H), 1.71 – 1.51 (m, 4H).

^{13}C NMR (101 MHz, CD_3CN) δ 174.59, 172.74, 160.68, 154.56, 153.13, 142.30, 132.48 (q, *J* = 32.2 Hz), 132.34, 127.82, 124.93 (q, *J* = 271.7 Hz), 117.12, 111.05, 110.91, 110.12 (q, *J* = 3.6 Hz), 109.27 (q, *J* = 4.0 Hz), 105.00, 66.96, 52.97, 51.93, 40.46, 37.27, 34.18, 25.37, 25.13.

HRMS (ESI-QTOF) m/z: $[M + H]^+$ Calcd for $C_{27}H_{33}F_3N_4O_5$: 551.2476; Found 551.2483

Compound **SI-21**

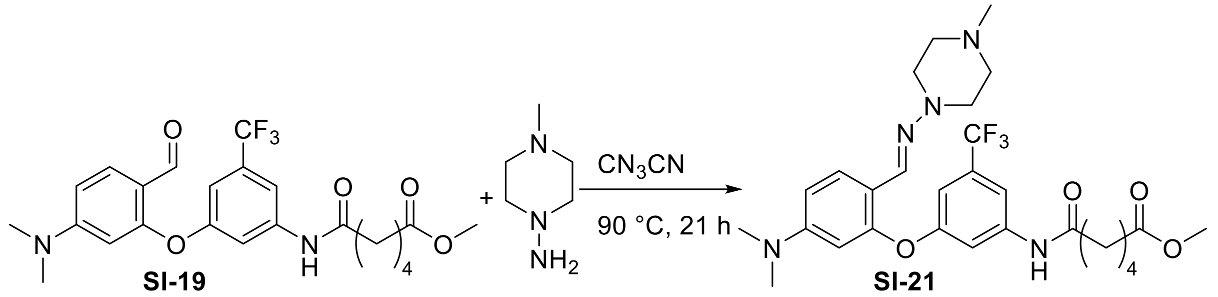

Compound **SI-19** (10 mg, 1 eq) and 1-Amino-4-methylpiperazin (25 mg, 26 µL, 10 eq) was dissolved in 1 mL of acetonitrile in a sealed screw cap vial and the solution was heated at 90 °C for 21 hours. Next day the solution was diluted with ethyl acetate (10 mL) and washed with water (3*10 mL), the organic layer dried over Na_2SO_4 and evaporated to give 11 mg (91 %) of yellow oil as product.

^1H NMR (400 MHz, CD_3CN) δ 8.47 (s, 1H), 7.73 (d, *J* = 8.8 Hz, 1H), 7.60 (s, 1H), 7.52 (s, 1H), 7.31 (t, *J* = 2.2 Hz, 1H), 6.92 (t, *J* = 2.1 Hz, 1H), 6.67 (dd, *J* = 8.9, 2.7 Hz, 1H), 6.34 (d, *J* = 2.6 Hz, 1H), 3.60 (s, 3H), 2.96 – 2.88 (m, 10H), 2.42 (t, *J* = 5.1 Hz, 4H), 2.35 – 2.26 (m, 4H), 2.20 (s, 3H), 1.69 – 1.55 (m, 4H)

^{13}C NMR (101 MHz, CD_3CN) δ 174.59, 172.73, 160.73, 154.39, 152.99, 142.31, 131.80, 127.72, 117.44, 110.95, 110.06 (q, *J* = 4.6 Hz), 109.23 (q, *J* = 4.1 Hz), 105.10, 55.08, 52.02, 51.94, 46.06, 40.48, 37.27, 34.18, 25.38, 25.13.

HRMS (ESI-QTOF) m/z: $[M + 2H]^+$ Calcd for $C_{28}H_{36}F_3N_5O_4$: 282.6432; Found 282.6432

Compound **SI-22**

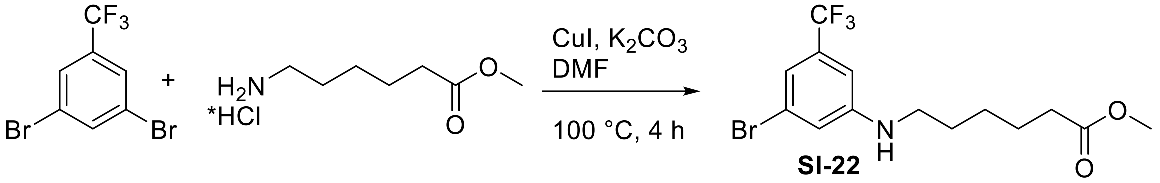

3,5-Dibromobenzotrifluoride (1 g, 0.513 mL, 1 eq), Methyl 6-aminohexanoate hydrochloride (0.597 g, 1 eq), CuI (0.627 g, 1 eq) and K_2CO_3 (0.908 g, 2 eq) was dissolved in 10 mL of dry DMF and the mixture was heated at 100 °C for 4 hours. After 4 hours, the mixture was poured on water, filtered through Cellite, washed with water and ethyl acetate to clear it from copper salts. The supernatant was diluted with ethyl acetate (to 30 mL) and washed with water (3*30 mL), the organic phase dried over Na_2SO_4 and evaporated. The product was purified with flash column chromatography (hexane:ethyl acetate 0 to 30% in 10 CV) to give 180 mg (31 %) of white solid product.

^{1}H NMR (400 MHz, $CDCl_3$) δ 7.02 (s, 1H), 6.84 (s, 1H), 6.68 (s, 1H), 3.93 (s, 1H), 3.68 (s, 3H), 3.12 (t, *J* = 6.9 Hz, 2H), 2.34 (t, *J* = 7.3 Hz, 2H), 1.73 – 1.61 (m, 4H), 1.48 – 1.39 (m, 2H).

^{13}C NMR (101 MHz, $CDCl_3$) δ 174.13, 149.70, 133.03 (q, *J* = 32.5 Hz), 124.91 (q, *J* = 274.0 Hz), 123.54, 117.95, 116.40 (q, *J* = 3.9 Hz), 108.01 (q, *J* = 3.8 Hz), 51.72, 43.46, 33.98, 28.89, 26.61, 24.65.

HRMS (ESI-QTOF) m/z: $[M + H]^+$ Calcd for $C_{14}H_{17}BrF_3NO_2$: 368.0468; Found 368.0465

Compound **SI-23**

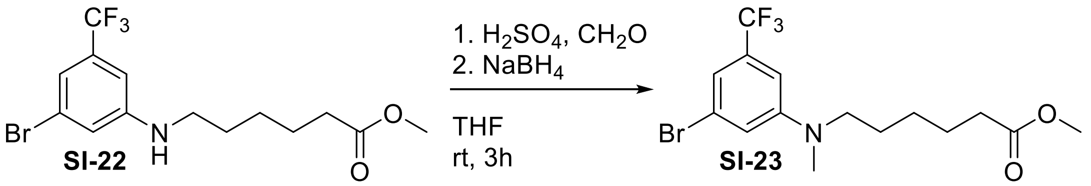

Compound **SI-23** was synthetized based on literature.[89] Aqueous formaldehyde (37%, 1 mL) was added to a mixture of THF (5 mL) and 3 M H_2SO_4 (1 mL) and stirred at room temperature for 10 minutes, then compound **SI-22** (180 mg) was added and the mixture was stirred for 3 hours. After 3 hours 1 g of $NaBH_4$ was slowly added to the flask while cooling it in an ice bath. The reaction mixture was diluted with ethyl acetate (50 mL) and washed with saturated $NaHCO_3$ solution (3*50 mL), the organic layer dried over Na_2SO_4 and evaporated to give 184 mg (98 %) of product.

^{1}H NMR (400 MHz, $CDCl_3$) δ 7.03 – 6.97 (m, 1H), 6.89 (t, *J* = 2.1 Hz, 1H), 6.74 (t, *J* = 2.0 Hz, 1H), 3.67 (s, 3H), 3.35 – 3.28 (m, 2H), 2.95 (s, 3H), 2.33 (t, *J* = 7.4 Hz, 2H), 1.72 – 1.63 (m, 2H), 1.63 – 1.58 (m, 2H), 1.40 – 1.32 (m, 2H).

^{13}C NMR (101 MHz, $CDCl_3$) δ 132.74 (q, *J* = 32.4 Hz), 124.81 (q, *J* = 272.9 Hz), 173.95, 150.16, 123.56, 117.19, 114.90 (q, *J* = 3.9 Hz), 106.88 (q, *J* = 3.8 Hz), 52.29, 51.55, 38.44, 33.89, 26.51, 26.36, 24.70.

HRMS (ESI-QTOF) m/z: $[M + H]^+$ Calcd for $C_{15}H_{19}BrF_3NO_2$: 382.0624; Found 382.0638

Compound **SI-24**

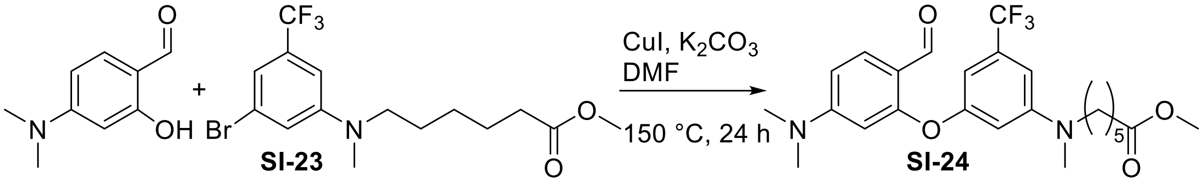

4-(Dimethylamino)salicylaldehyde (78 mg, 1 eq), compound **SI-23** (180 mg, 1 eq), CuI (90 mg, 1 eq) and K_2CO_3 (130 mg, 2 eq) was placed in a flame dried flask filled with Argon. The components were dissolved in 5 mL of dry Dimethylformamide and stirred under Argon for 24 hours at 150 °C. After 24 hours the mixture was poured on water, filtered through Cellite, washed with water and ethyl acetate to clear it from copper salts. The supernatant was diluted with ethyl acetate (to 30 mL) and washed with water (3*30 mL), the organic phase dried over Na_2SO_4 and evaporated. The product was purified with flash column chromatography (hexane:ethyl acetate 0 to 20% in 10 CV) to give 42 mg (19 %) of yellow oil.

^{1}H NMR (400 MHz, CD_3CN) δ 10.01 (s, 1H), 7.72 (d, *J* = 8.9 Hz, 1H), 6.67 (s, 1H), 6.60 (dd, *J* = 8.9, 2.5 Hz, 1H), 6.51 (s, 2H), 6.49 (s, 2H), 6.19 (d, *J* = 2.5 Hz, 1H), 3.59 (s, 3H), 3.30 (t, *J* = 7.5 Hz, 2H), 2.98 (s, 6H), 2.92 (s, 3H), 2.26 (t, *J* = 7.4 Hz, 2H), 1.59 – 1.48 (m, 4H), 1.32 – 1.22 (m, 2H).

^{13}C NMR (101 MHz, CD_3CN) δ 187.05, 174.67, 161.31, 160.30, 156.97, 152.09, 132.94 (q, *J* = 31.6 Hz), 130.94, 125.38 (q, *J* = 271.8 Hz), 117.33, 109.09, 105.30, 104.17 (q, *J* = 4.0 Hz), 102.33, 102.12 (q, *J* = 3.9 Hz), 52.77, 51.88, 40.36, 38.79, 34.41, 27.02, 26.78, 25.47.

HRMS (ESI-QTOF) m/z: $[M + H]^+$ Calcd for $C_{24}H_{29}F_3N_2O_4$: 467.2152; Found 467.2141

Compound **SI-25**

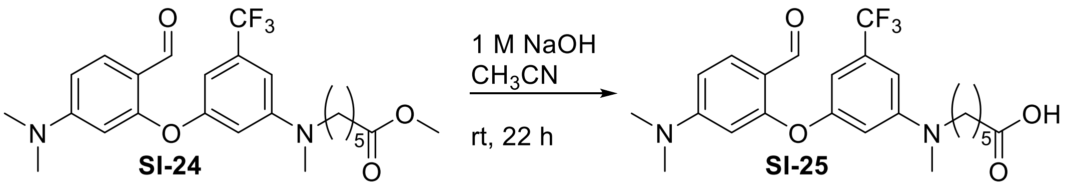

Compound **SI-24** (42 mg, 1 eq) was dissolved in 3 mL of acetonitrile and 1 mL of 1 M NaOH was added to the solution, which was then stirred at room temperature for 22 hours. After 22 hours 1 mL of 1 M HCl was added to the solution to neutralize the base, the solution was diluted with water (30 mL) and washed with ethyl acetate (3*30 mL), the combined organic phases dried over Na_2SO_4 and evaporated to give 40 mg (97%) yellow solid.

^{1}H NMR (400 MHz, $CDCl_3$) δ 10.09 (s, 1H), 7.83 (d, *J* = 9.0 Hz, 1H), 6.60 (s, 1H), 6.55 – 6.49 (m, 2H), 6.48 – 6.44 (m, 1H), 6.11 (d, *J* = 2.4 Hz, 1H), 3.31 (t, *J* = 7.4 Hz, 2H), 3.01 (s, 6H), 2.95 (s, 3H), 2.36 (t, *J* = 7.3 Hz, 2H), 1.66 (p, *J* = 7.3 Hz, 2H), 1.61 – 1.53 (m, 2H), 1.41 – 1.29 (m, 2H).

^{13}C NMR (101 MHz, $CDCl_3$) δ 187.40, 176.92, 160.91, 158.99, 155.89, 150.93, 130.49, 116.72, 108.03, 104.65, 103.75 (q, *J* = 4.0 Hz), 102.14 (q, *J* = 3.7 Hz), 101.08, 52.49, 40.27, 38.63, 33.55, 26.55, 26.52, 24.61.

HRMS (ESI-QTOF) m/z: $[M + H]^+$ Calcd for $C_{23}H_{27}F_3N_2O_4$: 453.1996; Found 453.1990

Compound **SI-25-T**

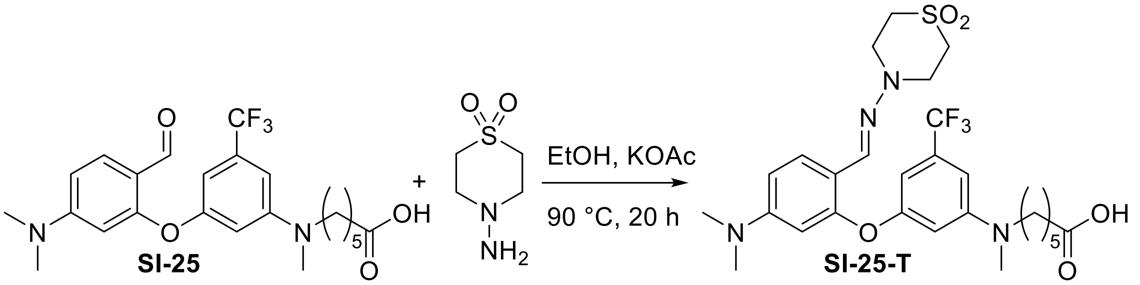

Compound **SI-25** (10 mg, 1 eq), 4-Aminothiomorpholine 1,1-Dioxide (33 mg, 10 eq) and potassium acetate (16 mg, 5 eq) was dissolved in 1 mL of ethanol in a sealed screw cap vial and the solution was heated at 90 °C for 20 hours. Then the solution was diluted with ethyl acetate (10 mL) and washed with water (3*10 mL), the organic layer dried over Na_2SO_4 and evaporated to give 12 mg (93 %) of yellow oil.

^{1}H NMR (400 MHz, CD_3CN) δ 7.71 (d, *J* = 8.9 Hz, 1H), 7.65 (s, 1H), 6.65 (dd, *J* = 8.8, 2.6 Hz, 1H), 6.58 (t, *J* = 2.1 Hz, 1H), 6.39 – 6.36 (m, 2H), 6.34 (t, *J* = 2.3 Hz, 1H), 3.72 – 3.61 (m, 4H), 3.28 – 3.20 (m, 2H), 2.93 (s, 6H), 2.90 (s, 6H), 2.77 (dd, *J* = 6.6, 3.9 Hz, 4H), 2.24 (t, *J* = 7.4 Hz, 2H), 1.59 – 1.44 (m, 4H), 1.26 – 1.18 (m, 2H).

^{13}C NMR (101 MHz, CD_3CN) δ 175.05, 161.35, 155.08, 153.48, 151.94, 137.31, 132.68 (q, *J* = 31.5 Hz), 128.22, 125.43 (q, *J* = 271.8 Hz), 116.43, 110.49, 105.04, 103.72, 103.26 (q, *J* = 4.2 Hz), 100.82 (q, *J* = 3.9 Hz), 52.85, 49.72, 48.64, 40.48, 38.77, 34.12, 27.04, 26.76, 25.39.

HRMS (ESI-QTOF) m/z: $[M + H]^+$ Calcd for $C_{27}H_{35}F_3N_4O_5S$: 585.2353; Found 585.2335

Compound **SI-25-T-NHS**

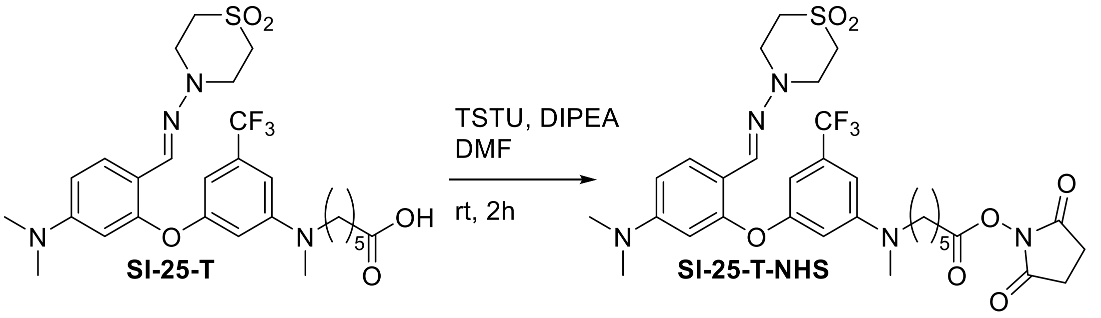

SI-25-T (5 mg, 1 eq), TSTU (N,N,N′,N′-Tetramethyl-O-(N-succinimidyl)uroniumtetrafluoroborat, 3.9 mg, 1.5 eq), and DIPEA (N,N-Diisopropylethylamine, 1.7 mg, 2.2 µL, 1.5 eq) was dissolved in 1 mL of dry DMF in a closed cap vial and stirred at room temperature for 2 hours. Then the compound was diluted with acetonitrile and purified with preparative HPLC (CH_3CN:H_2O 10-80% in 30 minutes) to give 2.9 mg (50 %) of product.

^{1}H NMR (400 MHz, CD_3CN) δ 7.71 (d, *J* = 8.9 Hz, 1H), 7.65 (s, 1H), 6.66 (dd, *J* = 8.8, 2.6 Hz, 1H), 6.58 (t, *J* = 2.1 Hz, 1H), 6.39 – 6.30 (m, 3H), 3.74 – 3.55 (m, 4H), 3.32 – 3.18 (m, 2H), 2.94 (s, 6H), 2.91 (s, 3H), 2.79 – 2.71 (m, 8H), 2.59 (t, *J* = 7.3 Hz, 2H), 1.67 (p, *J* = 7.4 Hz, 2H), 1.58 – 1.44 (m, 2H), 1.39 – 1.29 (m, 2H).

^{13}C NMR (101 MHz, CD_3CN) δ 171.15, 170.08, 161.34, 155.07, 153.48, 151.90, 137.18, 132.86 (q, *J* = 31.5 Hz), 128.22, 125.43 (q, *J* = 272.0 Hz), 116.42, 110.48, 105.02, 103.75, 103.30 (q, *J* = 4.2 Hz), 100.86 (q, *J* = 4.0 Hz), 52.79, 49.69, 48.64, 40.47, 38.79, 31.37, 26.63, 26.51, 26.39, 25.24.

HRMS (ESI-QTOF) m/z: $[M + H]^+$ Calcd for $C_{31}H_{38}F_3N_5O_7S$: 682.2517; Found 682.2498

Compound **SI-25-T-Halo**

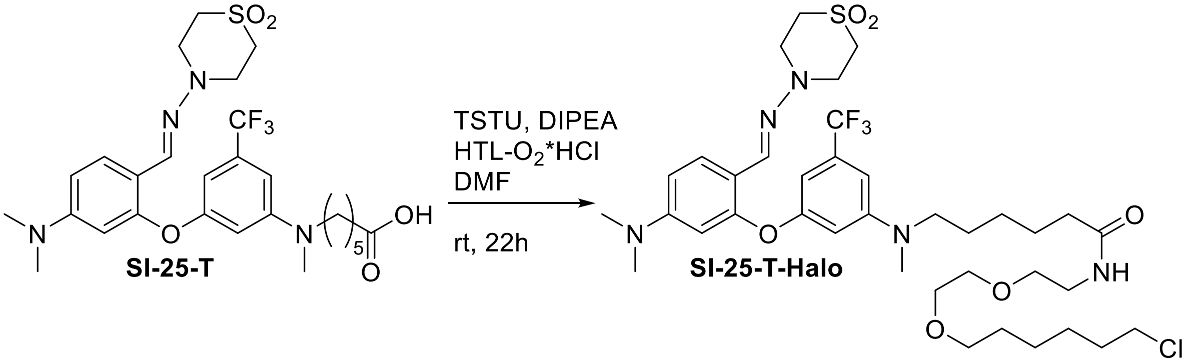

SI-25-T (5 mg, 1 eq), TSTU (N,N,N',N'-Tetramethyl-O-(N-succinimidyl)uroniumtetrafluoroborat, 3.9 mg, 1.5 eq), DIPEA (N,N-Diisopropylethylamine, 1.7 mg, 2.2 µL, 3 eq) and HaloTag ligand (2-(2-((6-Chlorohexyl)oxy)ethoxy)ethan-1-amine hydrochloride) (3.3 mg, 1.5 eq) was dissolved in 1 mL of dry DMF in a closed cap vial and stirred at room temperature for 20 hours. Then the compound was diluted with acetonitrile and purified with preparative HPLC (CH_3CN:H_2O 10-80% in 30 minutes) to give 3.4 mg (50 %) of product.

^{1}H NMR (400 MHz, None) δ 7.71 (d, *J* = 8.8 Hz, 1H), 7.65 (s, 1H), 6.65 (dd, *J* = 8.8, 2.6 Hz, 1H), 6.58 (t, *J* = 2.0 Hz, 1H), 6.39 (s, 1H), 6.37 – 6.32 (m, 3H), 3.72 – 3.61 (m, 4H), 3.57 (t, *J* = 6.7 Hz, 2H), 3.54 – 3.47 (m, 4H), 3.45 (t, *J* = 5.6 Hz, 2H), 3.40 (t, *J* = 6.6 Hz, 2H), 3.31 – 3.20 (m, 4H), 2.94 (s, 6H), 2.90 (s, 3H), 2.77 (dd, *J* = 6.9, 3.7 Hz, 4H), 2.08 (t, *J* = 7.4 Hz, 2H), 1.78 – 1.67 (m, 2H), 1.58 – 1.39 (m, 8H), 1.39 – 1.29 (m, 2H), 1.27 – 1.16 (m, 2H).

^{13}C NMR (101 MHz, None) δ 173.51, 161.36, 155.08, 153.47, 151.93, 137.20, 128.23, 116.43, 110.49, 105.03, 103.73, 103.30 (q, *J* = 4.2 Hz), 100.81 (q, *J* = 3.9 Hz), 71.63, 70.96, 70.77, 70.34, 52.91, 49.70, 48.65, 46.22, 40.49, 39.79, 38.81, 36.74, 33.34, 30.33, 27.39, 27.17, 26.80, 26.26, 26.18.

HRMS (ESI-QTOF) m/z: $[M + H]^+$ Calcd for $C_{37}H_{55}ClF_3N_5O_6S$: 790.3586; Found 790.3594

Description for the Synthesis of Si-bridged compounds

The absence of thermal activation meant that no further modifications to the core were necessary to fine-tune its electron density. After identifying the most promising hydrazones as model compounds **25** and **26,** we needed to modify them to include a linker for biological labelling. To accomplish this, we decided to formally replace one of the methyl groups in the dimethylamino groups with hexanoic acid methyl ester, followed by hydrolysis of the ester (Scheme S2) for subsequent modifications. To achieve this, we initiated a new synthetic route starting from 2-bromo-4-(dimethylamino)benzaldehyde (compound **SI-1**). We protected the aldehyde using pinacol (as protection with ethylene glycol was unsuccessful) to get **SI-31**. Next, we mixed compound **SI-31** with an excess of 1,3-dibromobenzene and performed a lithium-bromide exchange using butyllithium, followed by the addition of dichlorodimethylsilane (**SI-32**). Afterwards, Buchwald–Hartwig coupling with 6-aminohexanoic acid methyl ester yielded compound **SI-33**, which featured a long alkyl chain suitable for a linker. Finally, we methylated the amino group (compound **SI-34**) and conducted acidic hydrolysis on both the pinacolate and the methyl ester, resulting in an aldehyde with a free carboxylic acid (compound **SI-35**). Next step we created hydrazones by reacting the aldehyde with 4-Aminomorpholine or 1-Amino-4-methylpiperazine or 4-Aminothiomorpholine 1,1-Dioxide, yielding compounds **PULI$_{640}$-M, PULI$_{640}$-P** and **PULI$_{640}$-T**. Reacting their free carboxylic acid with TSTU created an NHS ester of the hydrazones, and further reacting it with Halotag ligand or BG-NH_2 created compounds with versatile labelling options.

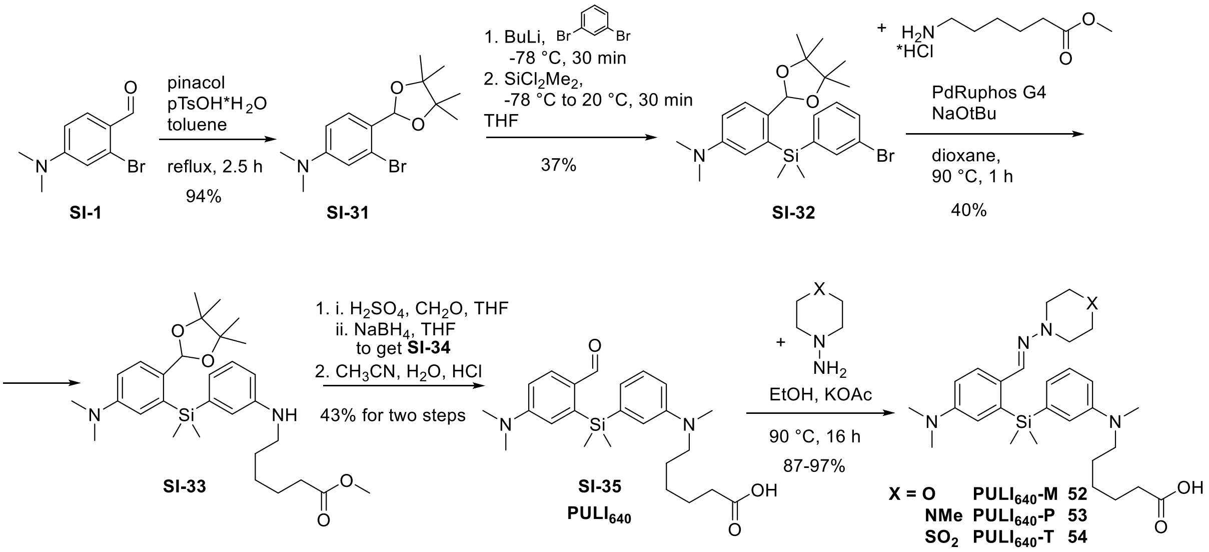

Scheme S2. Synthesis of Si-bridged PULI compounds for biological labelling

We also created compounds, where the Halotag ligand is directly attached to the PULI core. For this, we reacted 3-Bromoiodobenzene with 2-(2-Aminoethoxy)ethanol to get compound **SI-36**, then extended the linker further with 1-Chloro-6-iodohexane to get compound **SI-37** with the Halotag ligand attached directly to the aromatic core. Methylation of the secondary nitrogen (**SI-38**) was followed by mixing it with excess of compound **SI-31** and performed a lithium-bromide exchange using butyllithium, followed by the addition of dichlorodimethylsilane and separating the asymmetric product **SI-39**. Acidic hydrolysis of the pinacol protecting group (**SI-40**) was followed by reacting with the appropriate hydrazines to give **SI-41, SI-42** and **SI-43**. Unfortunately, labelling experiments with these compounds proved unsuccessful.

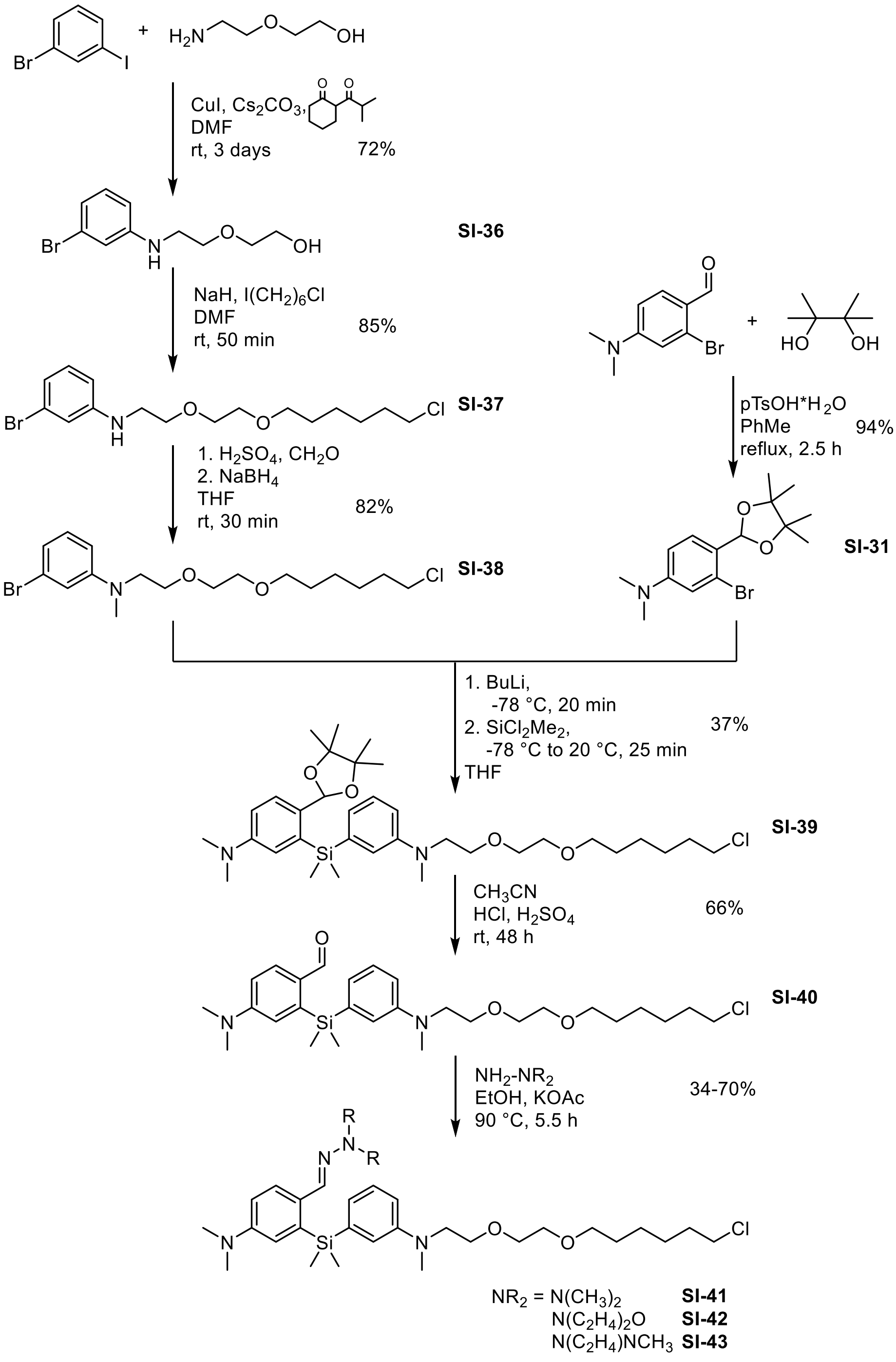

Scheme S3: Overall synthesis of silane compounds with a short HaloTag linker.

Synthesis and characterization of Si-bridged compounds

Compound **SI-26**

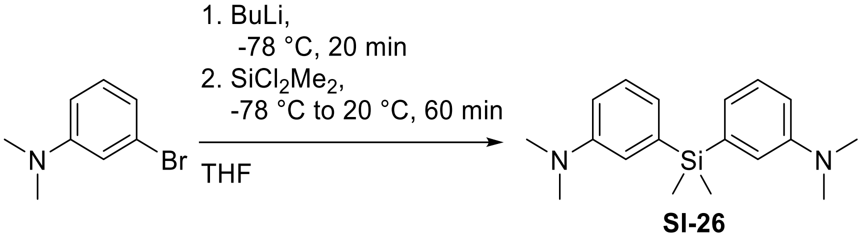

3-Bromo-dimethylaniline (1.0 g, 0.714 mL, 2.4 eq) was dissolved in 10 mL of dry Tetrahydrofuran in a flame dried flask filled with Argon and cooled to -78 °C. To the cooled solution 2.5 M *n*-Buthyllithium (2 mL, 2.4 eq) was added dropwise and the solution was stirred for 20 minutes, while white precipitate formed in the flask. To this suspension Dimethyldichlorosilane (0.269 g, 0.251 mL, 1 eq) dissolved in 2 mL of dry THF was added dropwise and the cleared out solution was let to warm up to room temperature. After stirring for an hour, saturated NH_4Cl solution (20 mL) was added to the reaction mixture and the product was extracted with ethyl acetate (3*20 mL). The combined organic phases were dried over Na_2SO_4 and evaporated. The product was purified with flash column chromatography (hexane:ethyl acetate 0 to 10% in 10 CV) to give 570 mg (92 %) of transparent oil.

^{1}H NMR (400 MHz, $CDCl_3$) δ 7.27 (dd, *J* = 8.3, 7.1 Hz, 2H), 6.97 (d, *J* = 2.8 Hz, 3H), 6.94 (dt, *J* = 7.1, 1.0 Hz, 4H), 6.79 (ddd, *J* = 8.3, 2.8, 1.0 Hz, 2H), 2.95 (s, 12H), 0.56 (s, 6H).

^{13}C NMR (101 MHz, $CDCl_3$) δ 150.07, 139.08, 128.61, 122.87, 118.47, 113.69, 40.81, -2.02.

HRMS (ESI-QTOF) m/z: $[M + H]^+$ Calcd for $C_{18}H_{26}N_2Si$: 299.1938; Found 299.1943

Compound **2**

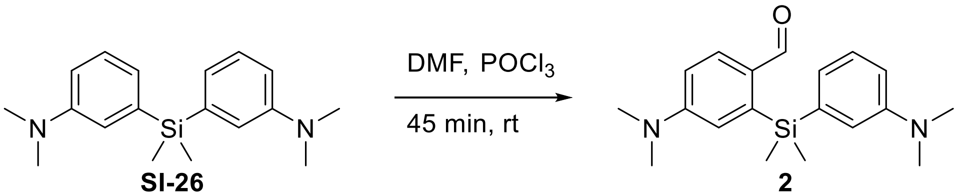

Dimethylformamide (2 ml) and $POCl_3$ (1 mL) was mixed slowly at room temperature, while the mixture warmed up. The mixture was let to cool back to room temperature (15 minutes), and then compound **SI-26** (230 mg) was added. The solution was stirred for 45 minutes, then poured on water (30 mL), saturated $NaHCO_3$ solution (20 mL) was added and washed with ethyl acetate (3*20 mL). The combined organic phases were dried over Na_2SO_4 and evaporated. The product was purified with flash column chromatography (hexane:ethyl acetate 0 to 30% in 10 CV) to give 45 mg (18 %) of faint yellow oil.

^{1}H NMR (400 MHz, $CDCl_3$) δ 9.85 (s, 1H), 7.82 (d, *J* = 8.7 Hz, 1H), 7.23 (dd, *J* = 8.3, 7.1 Hz, 1H), 6.94 (dd, *J* = 2.7, 1.0 Hz, 1H), 6.90 (d, *J* = 7.2 Hz, 1H), 6.81 (d, *J* = 2.7 Hz, 1H), 6.76 (ddd, *J* = 8.3, 2.8, 1.0 Hz, 1H), 6.71 (dd, *J* = 8.7, 2.7 Hz, 1H), 2.99 (s, 6H), 2.92 (s, 6H), 0.63 (s, 6H).

^{13}C NMR (101 MHz, $CDCl_3$) δ 190.97, 152.83, 150.13, 143.29, 139.78, 133.95, 129.81, 128.59, 122.87, 119.83, 118.46, 113.70, 111.29, 40.86, 39.97, -1.00.

For 2D HSQC and HMBC NMR see the NMR spectra

HRMS (ESI-QTOF) m/z: $[M + H]^+$ Calcd for $C_{19}H_{26}N_2OSi$: 327.1887; Found 327.1889

Compound **15**

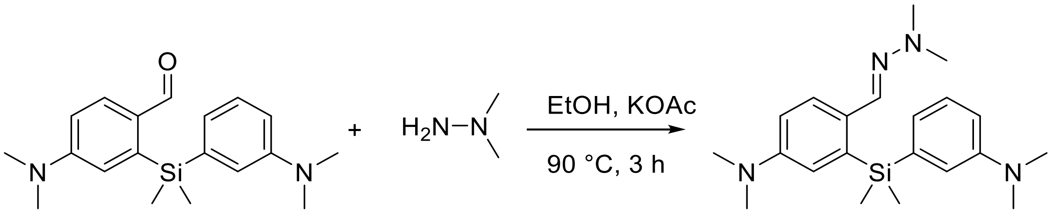

2 **15**

Compound **2** (18 mg, 1 eq), N, N-dimethylhydrazine (72 mg, 92 µL, 20 eq) and potassium acetate (46 mg, 6 eq) was dissolved in 1 mL of ethanol in a sealed screw cap vial and the solution was heated at 90 °C for 3 hours. Then the solution was diluted with ethyl acetate (10 mL) and washed with water (3*10 mL), the organic layer dried over Na_2SO_4 and evaporated. The product was further purified with preparative HPLC (CH_3CN:H_2O 10-80% in 30 minutes with 0.1 % formic acid) to give 4 mg (14 %) of product.

^{1}H NMR (400 MHz, $CDCl_3$) δ 7.80 (d, *J* = 8.7 Hz, 1H), 7.39 (s, 1H), 7.23 – 7.16 (m, 1H), 6.91 – 6.87 (m, 3H), 6.77 (dd, *J* = 8.7, 2.8 Hz, 1H), 6.73 (ddd, *J* = 8f.4, 2.5, 1.2 Hz, 1H), 2.95 (s, 6H), 2.89 (s, 6H), 2.64 (s, 6H), 0.57 (s, 6H).

^{13}C NMR (101 MHz, $CDCl_3$) δ 150.23, 149.34, 140.07, 137.31, 136.66, 130.66, 128.69, 125.99, 122.79, 119.25, 118.35, 114.13, 113.72, 43.34, 40.89, 40.63, -0.74.

HRMS (ESI-QTOF) m/z: $[M + H]^+$ Calcd for $C_{21}H_{32}N_4Si$: 369.2469; Found 369.2468

Compound **16**

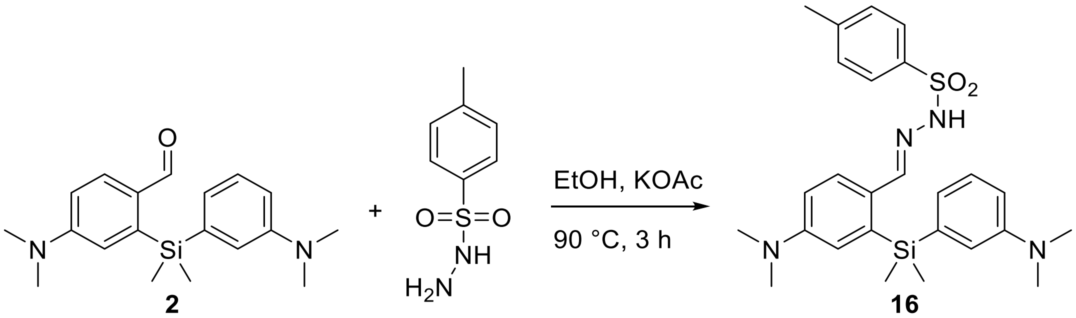

2 **16**

Compound **2** (18 mg, 1 eq), p-Toluolsulfonylhydrazid (205 mg, 20 eq) and potassium acetate (43 mg, 8 eq) was dissolved in 1 mL of ethanol in a sealed screw cap vial and stirred at 90°C for 3 hours. Then the solution was diluted with ethyl acetate (10 mL) and washed with water (3*10 mL), the organic layer dried over Na_2SO_4 and evaporated. The product was further purified with preparative HPLC (CH_3CN:H_2O 10-80% in 30 minutes with 0.1 % formic acid) to give 2 mg (7 %) of product.

^{1}H NMR (400 MHz, $CDCl_3$) δ 7.80 (d, *J* = 8.8 Hz, 1H), 7.78 – 7.73 (m, 3H), 7.26 (d, *J* = 7.7 Hz, 2H), 7.16 (dd, *J* = 8.2, 7.2 Hz, 1H), 7.10 (s, 1H), 6.81 (d, *J* = 2.8 Hz, 2H), 6.77 – 6.72 (m, 2H), 6.70 (dd, *J* = 8.8, 2.8 Hz, 1H), 2.98 (s, 6H), 2.89 (s, 6H), 2.40 (s, 3H), 0.49 (s, 6H).

^{13}C NMR (101 MHz, $CDCl_3$) δ 150.73, 143.92, 139.44, 139.25, 135.68, 129.65, 128.99, 128.08, 128.06, 126.17, 122.43, 118.30, 117.97, 113.82, 113.14, 40.75, 40.20, 21.72, -0.62.

HRMS (ESI-QTOF) m/z: $[M + 2H]^+$ Calcd for $C_{26}H_{34}N_4O_2SSi$: 248.1159; Found 248.1159

Compound **17**

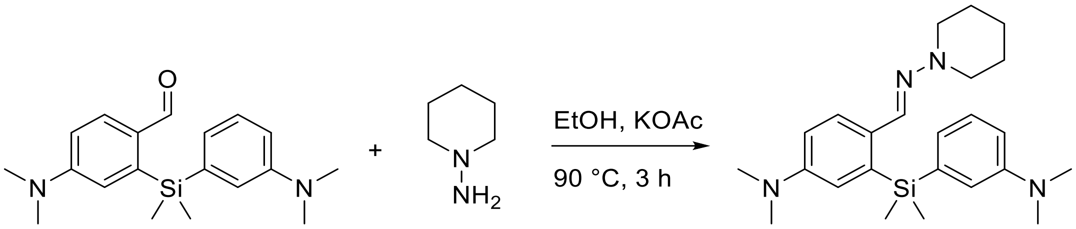

2 **17**

Compound **2** (18 mg, 1 eq), N-aminopiperidine (120 mg, 130 µL, 20 eq) and potassium acetate (65 mg, 12 eq) was dissolved in 1 mL of ethanol in a sealed screw cap vial and stirred at 90°C for 3 hours. Then the solution was diluted with ethyl acetate (10 mL) and washed with water (3*10 mL), the organic layer dried over Na_2SO_4 and evaporated. The product was further purified with preparative HPLC (CH_3CN:H_2O 10-80% in 30 minutes with 0.1 % formic acid) to give 9 mg (40 %) of product.

^{1}H NMR (400 MHz, $CDCl_3$) δ 7.84 (d, *J* = 8.8 Hz, 1H), 7.64 (s, 1H), 7.20 (dd, *J* = 8.3, 7.1 Hz, 1H), 6.92 – 6.86 (m, 3H), 6.79 – 6.70 (m, 2H), 2.95 (s, 6H), 2.90 (s, 6H), 2.83 – 2.77 (m, 5H), 1.64 – 1.58 (m, 4H), 1.47 – 1.39 (m, 2H), 0.58 (s, 6H).

^{13}C NMR (101 MHz, $CDCl_3$) δ 154.62, 151.02, 150.13, 140.18, 140.05, 129.16, 128.89, 126.12, 123.15, 119.25, 118.42, 114.00, 113.13, 54.61, 41.02, 40.32, 23.55, 22.75, -0.78.

HRMS (ESI-QTOF) m/z: $[M + H]^+$ Calcd for $C_{24}H_{36}N_4Si$: 409.2782; Found 409.2784

Compound **18**

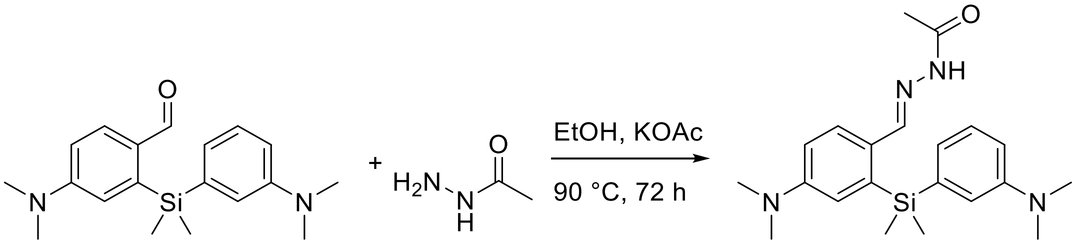

2 **18**

Compound **2** (10 mg, 1 eq), Acetohydrazide (45 mg, 20 eq) and potassium acetate (36 mg, 12 eq) was dissolved in 1 mL of ethanol in a sealed screw cap vial and stirred at 90°C for 3 days. Then the solution was diluted with ethyl acetate (10 mL) and washed with water (3*10 mL), the organic layer dried over Na_2SO_4 and evaporated. The product was purified with flash column chromatography (hexane:ethyl acetate 0 to 40% in 10 CV) to give 1 mg (9 %) of product.

^{1}H NMR (400 MHz, $CDCl_3$) δ 8.13 (s, 1H), 7.88 (d, *J* = 8.8 Hz, 1H), 7.69 (s, 1H), 7.25 – 7.20 (m, 1H), 6.89 (d, *J* = 2.8 Hz, 1H), 6.86 – 6.82 (m, 2H), 6.78 – 6.75 (m, 2H), 3.01 (s, 6H), 2.91 (s, 6H), 2.26 (s, 3H), 0.56 (s, 6H).

^{13}C NMR (101 MHz, $CDCl_3$) δ 172.64, 150.55, 150.18, 144.98, 139.25, 139.12, 129.08, 127.45, 126.64, 122.43, 118.54, 117.91, 113.98, 113.38, 40.78, 40.27, 20.53, -0.53.

HRMS (ESI-QTOF) m/z: $[M + H]^+$ Calcd for $C_{21}H_{30}N_4OSi$: 383.2262; Found 383.2252

Compound **19**

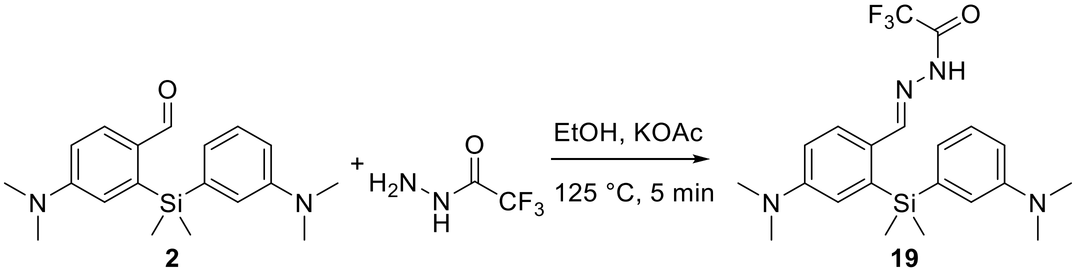

In a screw cap vial 1 mL of 1M N_2H_4 in THF (32.6 eq) was mixed with 210 mg (140 µL, 32.6 eq) of trifluoracetic anhydride and the mixture was heated at 90 °C for 5 minutes. The mixture was cooled down and added to 10 mg (1 eq) of compound **2** and 30 mg of potassium acetate (3.3 eq) and the closed vial was then heated to 125 °C for 5 minutes. The crude product was purified with flash column chromatography (hexane:ethyl acetate 0 to 30% in 10 CV), then the solution was dissolved in DCM (10 mL) and washed with water (3*10 mL), the organic layer dried over Na_2SO_4 and evaporated to give 6 mg (45 %) of product.

^{1}H NMR (400 MHz, CD_3CN) δ 10.21 (b1s, 1H), 8.34 (s, 1H), 7.86 (d, *J* = 8.9 Hz, 1H), 7.19 (dd, *J* = 8.4, 7.1 Hz, 1H), 6.93 – 6.85 (m, 2H), 6.82 – 6.74 (m, 3H), 2.97 (s, 6H), 2.86 (s, 6H), 0.61 (s, 6H).

^{13}C NMR (101 MHz, CD_3CN) δ 175.06, 154.35, 153.60 (q, *J* = 36.7 Hz), 152.23, 151.28, 141.31, 139.87, 129.51, 129.16, 126.12, 123.13, 119.67, 118.93, 114.54, 113.77, 40.73, 40.11, -0.62.

HRMS (ESI-QTOF) m/z: $[M + H]^+$ Calcd for $C_{21}H_{27}F_3N_4OSi$: 437.1979; Found 437.1983

Compound **20**

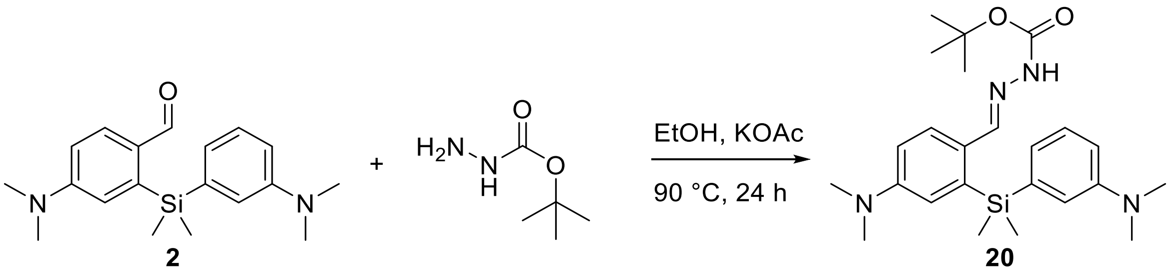

Compound **2** (10 mg, 1 eq), tert-Butyl carbazate (120 mg, 30 eq) and potassium acetate (33 mg, 11 eq) was dissolved in 1 mL of ethanol in a sealed screw cap vial and stirred at 90°C for 24 hours. Then the solution was diluted with ethyl acetate (10 mL) and washed with water (3*10 mL), the organic layer dried over Na_2SO_4 and evaporated to give 6 mg (44 %) of product.

^{1}H NMR (400 MHz, CD_3CN) δ 8.61 (bs, 1H), 7.95 (s, 1H), 7.74 (d, *J* = 8.8 Hz, 1H), 7.19 (dd, *J* = 8.3, 7.1 Hz, 1H), 6.88 (dd, *J* = 2.7, 0.9 Hz, 1H), 6.85 (d, *J* = 2.8 Hz, 1H), 6.83 – 6.73 (m, 3H), 2.93 (s, 6H), 2.87 (s, 6H), 1.45 (s, 9H), 0.58 (s, 6H).

^{13}C NMR (101 MHz, CD_3CN) δ 153.56, 151.32, 151.26, 145.57, 140.21, 139.27, 129.46, 128.21, 128.01, 123.11, 119.68, 118.92, 114.43, 114.09, 80.54, 40.77, 40.25, 28.48, -0.63.

HRMS (ESI-QTOF) m/z: $[M + H]^+$ Calcd for $C_{24}H_{36}N_4O_2Si$: 441.2680; Found 441.2691

Compound **21**

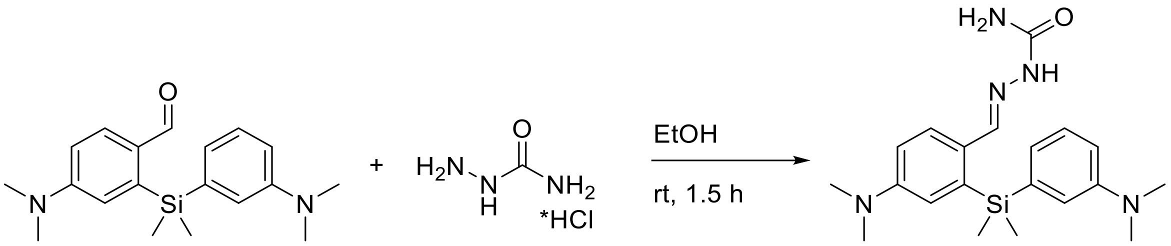

2 **21**

Compound **2** (10 mg, 1 eq) and Semicarbazide hydrochloride (22 mg, 6.5 eq) was dissolved in 1 mL of ethanol in a sealed screw cap vial and stirred at room temperature for 1.5 hours. Then the solution was diluted with ethyl acetate (10 mL) and washed with water (3*10 mL), the organic layer dried over Na_2SO_4 and evaporated to give 12 mg (93 %) of product.

^{1}H NMR (400 MHz, $CDCl_3$) δ 8.21 (s, 1H), 7.74 – 7.65 (m, 2H), 7.21 (dd, *J* = 8.3, 7.1 Hz, 1H), 6.93 (d, *J* = 2.8 Hz, 1H), 6.90 – 6.80 (m, 2H), 6.74 (dt, *J* = 8.4, 2.3 Hz, 2H), 5.98 – 4.41 (bs, 2H), 3.00 (s, 6H), 2.89 (s, 6H), 0.56 (s, 6H).

^{13}C NMR (101 MHz, $CDCl_3$) δ 157.47, 150.33, 150.19, 143.59, 139.57, 138.12, 128.96, 128.39, 127.06, 122.58, 119.22, 118.18, 113.89, 113.07, 40.81, 40.27, -0.38.

HRMS (ESI-QTOF) m/z: $[M + H]^+$ Calcd for $C_{20}H_{29}N_5OSi$: 384.2214; Found 384.2218

Compound **22**

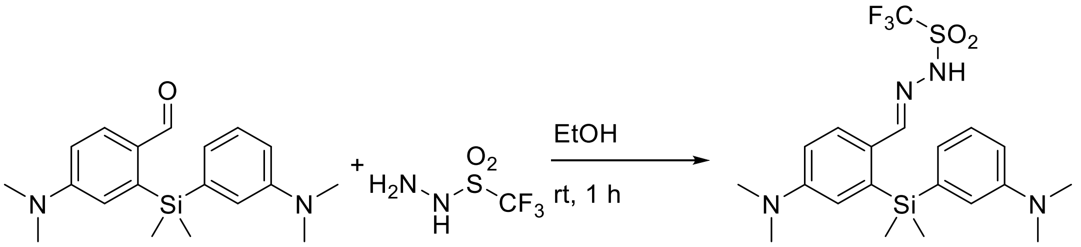

2 **22**

In a screw cap vial 1 mL of 1M N_2H_4 in THF (32.6 eq) was mixed with 282 mg (168 µL, 32.6 eq) of Trifluoromethanesulfonic anhydride and stirred at room temperature for 15 minutes, then to this 10 mg (1 eq) of compound **2** dissolved in 1 mL of ethanol was added and the reaction mixture was stirred at room temperature for 1 hour. The solution was diluted with ethyl acetate (10 mL) and washed with water (3*10 mL), the organic layer dried over Na_2SO_4 and evaporated. The product was further purified with preparative HPLC (CH_3CN:H_2O 10-80% in 30 minutes) to give 13 mg (90 %) of product.

^{1}H NMR (400 MHz, $CDCl_3$) δ 8.09 (s, 1H), 7.82 (d, *J* = 8.9 Hz, 1H), 7.71 (s, 1H), 7.35 (t, *J* = 7.7 Hz, 1H), 7.22 (d, *J* = 7.4 Hz, 1H), 7.14 (d, *J* = 8.3 Hz, 1H), 6.88 (d, *J* = 2.8 Hz, 1H), 6.72 (dd, *J* = 8.8, 2.8 Hz, 1H), 3.09 (s, 6H), 3.02 (s, 6H), 0.64 (s, 6H).

^{13}C NMR (101 MHz, $CDCl_3$) δ 150.89, 150.44, 138.28, 129.99, 128.70, 125.76, 122.66, 121.43, 118.89, 118.21, 117.74, 113.50, 111.31, 40.26, -1.50.

HRMS (ESI-QTOF) m/z: $[M + H]^+$ Calcd for $C_{20}H_{27}F_3N_4O_2SSi$: 473.1649; Found 473.1654

Compound **23**

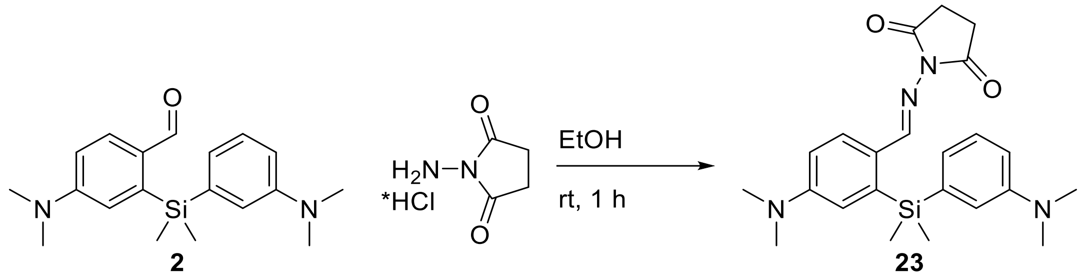

Compound **2** (10 mg, 1 eq) and N-Aminosuccinimide Hydrochloride (18 mg, 4 eq) was dissolved in 1 mL of ethanol in a sealed screw cap vial and stirred at room temperature for 1 hour. Then the solution was diluted with ethyl acetate (10 mL) and washed with water (3*10 mL), the organic layer dried over Na_2SO_4 and evaporated. The product was further purified with preparative HPLC (CH_3CN:H_2O 10-80% in 30 minutes) to give 3 mg (23 %) of product.

^{1}H NMR (400 MHz, $CDCl_3$) δ 9.01 (s, 1H), 8.16 (d, *J* = 8.9 Hz, 1H), 7.22 (t, *J* = 7.7 Hz, 1H), 6.98 – 6.57 (m, 5H), 3.00 (s, 6H), 2.92 (s, 6H), 2.72 (s, 4H), 0.64 (s, 6H).

^{13}C NMR (101 MHz, $CDCl_3$) δ 173.34, 163.59, 151.73, 150.11, 142.47, 139.07, 129.14, 128.79, 125.26, 122.83, 118.61, 118.11, 113.79, 112.70, 40.07, 26.86, -0.67.

HRMS (ESI-QTOF) m/z: $[M + H]^+$ Calcd for $C_{23}H_{30}N_4O_2Si$: 423.2211; Found 423.2207

Compound **24**

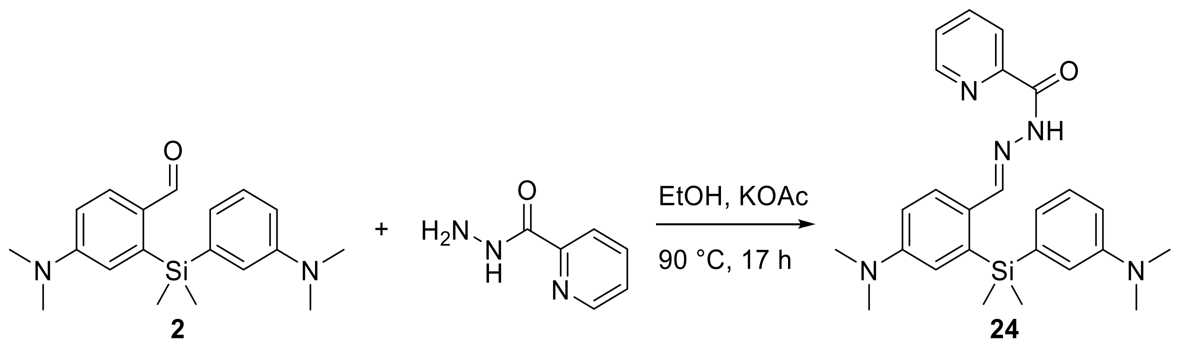

Compound **2** (10 mg, 1 eq), 2-Pyridinecarboxylic Acid Hydrazide (84 mg, 20 eq) and potassium acetate (33 mg, 11 eq) was dissolved in 1 mL of ethanol in a sealed screw cap vial and stirred at 90°C for 17 hours. Then the solution was diluted with DCM (10 mL) and washed with water (3*10 mL), the organic layer dried over Na_2SO_4 and evaporated. The product was purified with flash column chromatography (DCM:Methanol 0 to 5% in 10 CV) to give 10 mg (73 %) of product.

^{1}H NMR (400 MHz, CD_3CN) δ 10.67 (s, 1H), 8.59 (ddd, *J* = 4.8, 1.7, 1.0 Hz, 1H), 8.34 (s, 1H), 8.12 (dt, *J* = 7.8, 1.1 Hz, 1H), 7.95 (td, *J* = 7.7, 1.7 Hz, 1H), 7.86 (d, *J* = 8.8 Hz, 1H), 7.54 (ddd, *J* = 7.6, 4.7, 1.3 Hz, 1H), 7.19 (dd, *J* = 8.3, 7.2 Hz, 1H), 6.97 (dd, *J* = 2.8, 1.0 Hz, 1H), 6.91 (d, *J* = 2.8 Hz, 1H), 6.86 – 6.77 (m, 2H), 6.74 (ddd, *J* = 8.3, 2.8, 1.0 Hz, 1H), 2.97 (s, 6H), 2.86 (s, 6H), 0.64 (s, 6H).

^{13}C NMR (101 MHz, CD_3CN) δ 160.53, 151.77, 151.33, 150.88, 150.58, 149.26, 140.49, 140.44, 138.74, 129.55, 128.86, 127.61, 127.40, 123.08, 123.05, 119.67, 118.84, 114.45, 113.93, 40.74, 40.21, -0.70.

HRMS (ESI-QTOF) m/z: $[M + H]^+$ Calcd for $C_{25}H_{31}N_5OSi$: 446.2371; Found 446.2380

Compound **25**

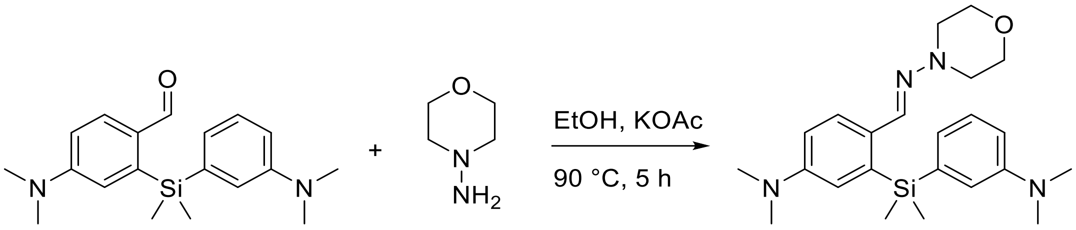

2 **25**

Compound **2** (22 mg, 1 eq), 4-aminomorpholine (138 mg, 130 µL, 20 eq) and potassium acetate (46 mg, 7 eq) was dissolved in 1 mL of ethanol in a sealed screw cap vial and stirred at 90°C for 5 hours. Then the solution was diluted with ethyl acetate (10 mL) and washed with water (3*10 mL), the organic layer dried over Na_2SO_4 and evaporated to give 22 mg (80 %) of product.

^{1}H NMR (400 MHz, $CDCl_3$) δ 7.84 (d, *J* = 8.8 Hz, 1H), 7.67 (s, 1H), 7.21 (dd, *J* = 8.0, 7.0 Hz, 1H), 6.93 (d, *J* = 2.8 Hz, 1H), 6.91 – 6.86 (m, 2H), 6.78 (dd, *J* = 8.8, 2.8 Hz, 1H), 6.74 (ddd, *J* = 8.3, 2.7, 1.1 Hz, 1H), 3.79 – 3.69 (m, 4H), 2.98 (s, 6H), 2.90 (s, 6H), 2.82 – 2.76 (m, 4H), 0.58 (s, 6H).

^{13}C NMR (101 MHz, $CDCl_3$) δ 150.24, 149.77, 140.03, 140.01, 137.64, 129.46, 128.81, 126.37, 122.57, 118.84, 118.10, 113.87, 113.63, 66.61, 52.34, 40.78, 40.47, -0.76.

HRMS (ESI-QTOF) m/z: $[M + H]^+$ Calcd for $C_{23}H_{34}N_4OSi$: 411.2575; Found 411.2575

Compound **26**

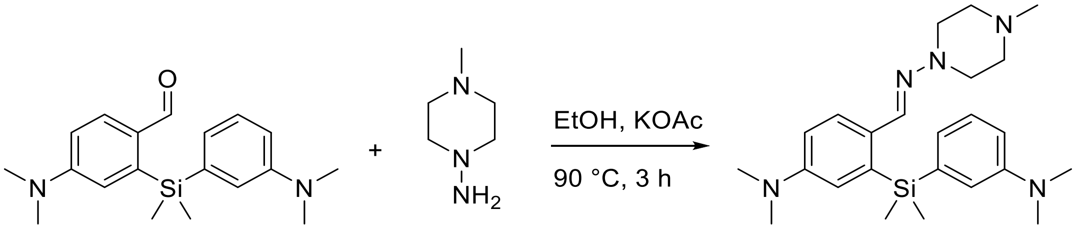

2 **26**

Compound **2** (18 mg, 1 eq), 1-Amino-4-methylpiperazin (139 mg, 145 µL, 20 eq) and potassium acetate (49 mg, 9 eq) was dissolved in 1 mL of ethanol in a sealed screw cap vial and stirred at 90°C for 3 hours. Then the solution was diluted with ethyl acetate (10 mL) and washed with water (3*10 mL), the organic layer dried over Na_2SO_4 and evaporated. The product was further purified with preparative HPLC (CH_3CN:H_2O 10-80% in 30 minutes with 0.1 % formic acid) to give 3 mg (13 %) of product.

^{1}H NMR (400 MHz, $CDCl_3$) δ 7.81 (d, *J* = 8.8 Hz, 1H), 7.64 (s, 1H), 7.20 (dd, *J* = 8.4, 7.3 Hz, 1H), 6.93 (d, *J* = 2.8 Hz, 1H), 6.87 – 6.83 (m, 2H), 6.76 (dd, *J* = 8.8, 2.8 Hz, 1H), 6.72 (ddd, *J* = 8.3, 2.7, 1.1 Hz, 1H), 3.06 (t, *J* = 5.1 Hz, 4H), 3.00 (s, 6H), 2.89 (s, 6H), 2.83 – 2.74 (m, 4H), 2.51 (s, 3H), 0.55 (s, 6H).

^{13}C NMR (101 MHz, $CDCl_3$) δ 150.32, 150.05, 143.12, 140.32, 137.91, 128.98, 128.65, 126.69, 122.40, 118.79, 117.90, 113.75, 113.69, 51.60, 49.13, 43.58, 40.78, 40.44, -0.67.

HRMS (ESI-QTOF) m/z: $[M + H]^+$ Calcd for $C_{24}H_{37}N_5Si$: 424.2891; Found 424.2891

Compound **SI-27**

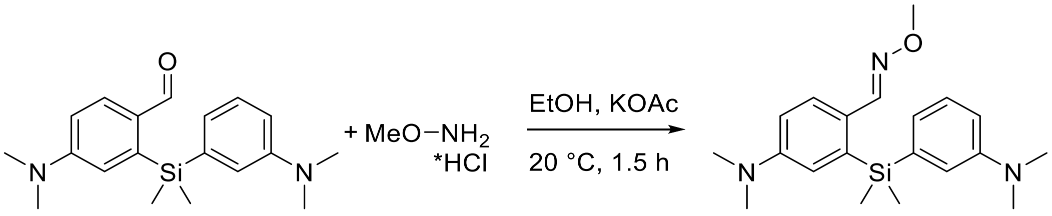

Compound **2** (24 mg, 1 eq), Methoxyamine hydrochloride (61 mg, 10 eq) and potassium acetate (30 mg, 4 eq) was dissolved in 1 mL of ethanol in a sealed screw cap vial and stirred for 90 minutes at room temperature. The solution was poured on water (10 mL), and washed with ethyl acetate (3*10 mL). The combined organic phases were dried over Na_2SO_4 and evaporated to give 22 mg (84 %) of product.

^{1}H NMR (400 MHz, $CDCl_3$) δ 8.21 (s, 1H), 7.75 (d, *J* = 8.7 Hz, 1H), 7.23 (dd, *J* = 8.3, 7.1 Hz, 1H), 6.90 (dd, *J* = 2.6, 1.0 Hz, 1H), 6.88 (dt, *J* = 7.1, 1.0 Hz, 1H), 6.84 (d, *J* = 2.8 Hz, 1H), 6.76 (ddd, *J* = 8.3, 2.7, 1.0 Hz, 1H), 6.72 (dd, *J* = 8.8, 2.9 Hz, 1H), 3.85 (s, 3H), 2.94 (s, 6H), 2.92 (s, 6H), 0.59 (s, 6H).

^{13}C NMR (101 MHz, $CDCl_3$) δ 150.30, 150.18, 149.82, 139.13, 138.64, 128.71, 127.71, 125.35, 122.78, 119.26, 118.41, 113.82, 113.17, 61.56, 40.82, 40.23, -0.49.

HRMS (ESI-QTOF) m/z: [M + 2H]$^+$ Calcd for $C_{20}H_{29}N_3OSi$: 178.6113; Found 178.6112

Compound **SI-28**

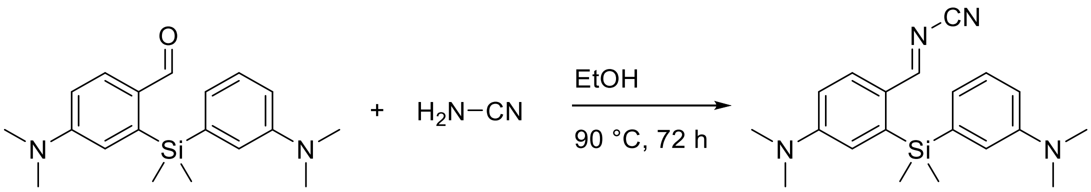

Compound **2** (10 mg, 1 eq) and Cyanamide (21 mg, 16 eq) was dissolved in 1 mL of ethanol in a sealed screw cap vial and stirred at 90°C for 3 days. Then the solution was diluted with ethyl acetate (10 mL) and washed with water (3*10 mL), the organic layer dried over Na_2SO_4 and evaporated. The product was further purified with preparative HPLC (CH_3CN:H_2O 10-80% in 30 minutes) to give 1.5 mg (14 %) of product.

^{1}H NMR (400 MHz, $CDCl_3$) δ 9.83 (s, 1H), 7.81 (d, *J* = 8.7 Hz, 1H), 7.25 – 7.19 (m, 1H), 6.96 – 6.75 (m, 4H), 6.71 (dd, *J* = 8.7, 2.7 Hz, 1H), 3.00 (s, 6H), 2.93 (s, 6H), 0.62 (s, 6H).

^{13}C NMR (101 MHz, $CDCl_3$) δ 190.97, 153.80, 152.86, 150.18, 134.01, 129.82, 129.08, 128.66, 122.93, 119.86, 117.89, 113.70, 112.24, 111.31, 40.09, 40.00, -1.01.

HRMS (ESI-QTOF) m/z: [M + H]$^+$ Calcd for $C_{20}H_{26}N_4Si$: 351.1999; Found 351.1999

Compound **SI-29**

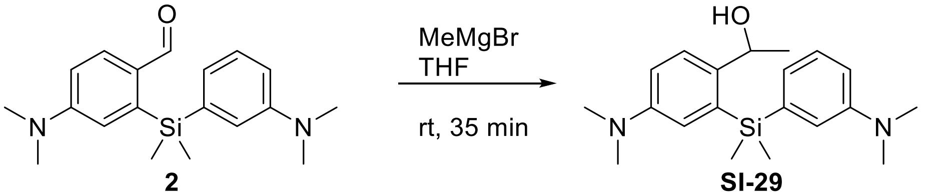

Compound **2** (70 mg, 1 eq) was dissolved in 3 mL of dry THF in a flame dried flask filled with Argon. To this solution 0.214 mL of 3M MeMgBr (3 eq) solution was added dropwise and the reaction mixture was stirred at room temperature for 35 minutes. The reaction was quenched by adding 10 mL of saturated NH_4Cl solution, then washed with ethyl acetate (3*10 mL), the combined organic phases were dried over Na_2SO_4 and evaporated to give 70 mg (99%) of product.

^{1}H NMR (400 MHz, $CDCl_3$) δ 7.48 (d, *J* = 8.6 Hz, 1H), 7.23 (dd, *J* = 8.3, 7.1 Hz, 1H), 6.95 – 6.89 (m, 2H), 6.89 – 6.82 (m, 2H), 6.76 (ddd, *J* = 8.3, 2.8, 1.0 Hz, 1H), 4.95 (q, *J* = 6.3 Hz, 1H), 2.95 (s, 6H), 2.92 (s, 6H), 1.31 (d, *J* = 6.3 Hz, 3H), 0.60 (d, *J* = 1.3 Hz, 6H).

^{13}C NMR (101 MHz, $CDCl_3$) δ 150.22, 149.33, 139.97, 139.82, 135.99, 128.83, 126.52, 122.38, 119.11, 117.85, 114.62, 113.76, 69.09, 40.78, 40.73, 24.19, -0.48, -0.55.

HRMS (ESI-QTOF) m/z: [M + 2H]$^+$ Calcd for $C_{20}H_{30}N_2OSi$: 172.1136; Found 172.1136

Compound **SI-30**

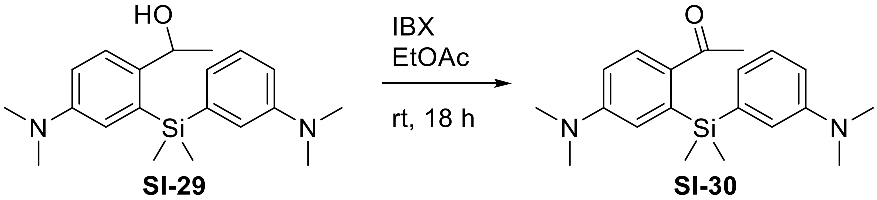

Compound **SI-29** (80 mg, 1 eq) and 2-Iodoxybenzoic acid (IBX, 160 mg, 2.44 eq) was dissolved in 8 mL of ethyl acetate and the reaction mixture stirred overnight at room temperature. Next day the solvent was evaporated and the crude product was purified with flash column chromatography (hexane:ethyl acetate 0 to 20% in 10 CV) to give 55 mg (69 %) of product.

^{1}H NMR (400 MHz, $CDCl_3$) δ 7.83 (d, *J* = 8.8 Hz, 1H), 7.21 (dd, *J* = 8.3, 7.1 Hz, 1H), 6.99 (d, *J* = 2.4 Hz, 1H), 6.93 (d, *J* = 7.1 Hz, 1H), 6.79 (d, *J* = 2.8 Hz, 1H), 6.77 – 6.69 (m, 1H), 6.60 (dd, *J* = 8.7, 2.8 Hz, 1H), 2.91 (s, 6H), 2.90 (s, 6H), 2.48 (s, 3H), 0.58 (s, 6H).

^{13}C NMR (101 MHz, $CDCl_3$) δ 196.71, 152.04, 142.41, 132.71, 130.20, 128.28, 121.57, 110.36, 39.90, 26.25, -1.08.

HRMS (ESI-QTOF) m/z: [M + H]$^+$ Calcd for $C_{20}H_{28}N_2OSi$: 341.2044; Found 341.2046

Compound **SI-31**

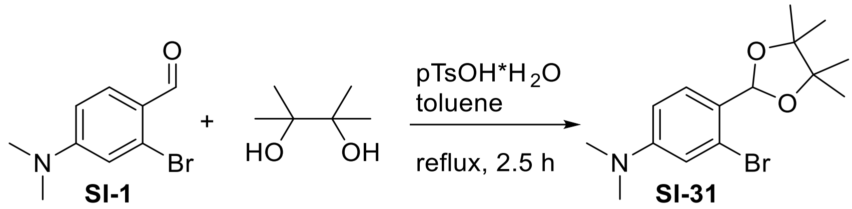

Compound **SI-1** (1 g, 1 eq), pinacol (1.55 g, 3 eq) and p-toluenesulfonic acid monohydrate (83 mg, 0.1 eq) was dissolved in 50 mL of toluene in a round bottom flask equipped with a Dean–Stark apparatus. The mixture was refluxed for 2.5 hours, then cooled, diluted with ethyl acetate (50 mL) and washed with water (3*50 mL), the organic layer dried over Na_2SO_4 and evaporated. The product was purified with flash column chromatography (hexane:ethyl acetate 0 to 20% in 10 CV) to give 1.35 g (94 %) of product.

^{1}H NMR (400 MHz, $CDCl_3$) δ 7.50 (d, *J* = 8.7 Hz, 1H), 6.83 (d, *J* = 2.6 Hz, 1H), 6.65 (dd, *J* = 8.8, 2.6 Hz, 1H), 6.17 (s, 1H), 2.93 (s, 6H), 1.33 (s, 6H), 1.29 (s, 6H).

^{13}C NMR (101 MHz, $CDCl_3$) δ 151.67, 128.30, 125.21, 124.34, 115.94, 111.39, 99.27, 82.55, 40.46, 24.65, 22.39.

HRMS (ESI-QTOF) m/z: $[M + H]^+$ Calcd for $C_{15}H_{22}BrNO_2$: 328.0907; Found 328.0901

Compound **SI-32**

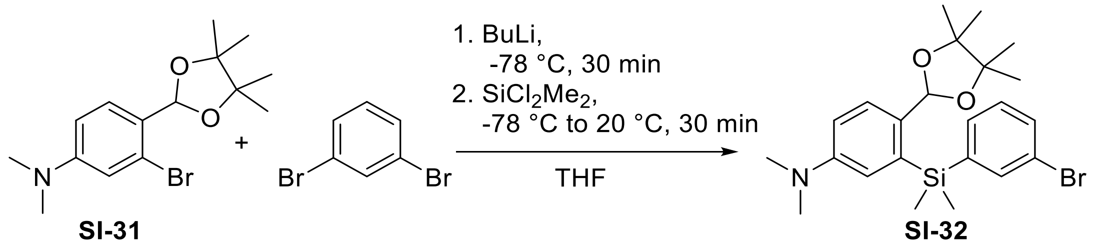

Compound **SI-31** (370 mg, 1 eq) and 1,3-Dibromobenzene (798 mg, 409 µL, 3 eq) was dissolved in 10 mL of dry THF in a flame dried flask filled with Argon and the solution was cooled to -78 °C. To the cooled solution 2.5 M *n*-Buthyllithium (2 mL, 4.4 eq) was added dropwise and the solution was stirred for 30 minutes, while white precipitate formed in the flask. To this suspension Dimethyldichlorosilane (320 mg, 0.3 mL, 2.2 eq) dissolved in 2 mL of dry THF was added dropwise and the cleared out solution was stirred at -78 °C for 30 further minutes, then it was let to warm up to room temperature. After stirring for another hour, water (20 mL) was added to the reaction mixture and the product was extracted with ethyl acetate (3*20 mL). The combined organic phases were dried over Na_2SO_4 and evaporated. The product was purified with flash column chromatography (hexane:ethyl acetate 0 to 10% in 10 CV) to give 416 mg (37 %) of oil as product.

^{1}H NMR (400 MHz, $CDCl_3$) δ 7.66 (dd, *J* = 2.2, 1.1 Hz, 1H), 7.58 (d, *J* = 8.6 Hz, 1H), 7.45 (ddd, *J* = 7.9, 2.1, 1.1 Hz, 1H), 7.40 (dt, *J* = 7.3, 1.1 Hz, 1H), 7.18 (t, *J* = 7.6 Hz, 1H), 6.83 (d, *J* = 2.8 Hz, 1H), 6.79 (dd, *J* = 8.6, 2.8 Hz, 1H), 5.84 (s, 1H), 2.91 (s, 6H), 1.21 (s, 6H), 1.13 (s, 6H), 0.60 (s, 6H).

^{13}C NMR (101 MHz, $CDCl_3$) δ 150.11, 143.25, 136.77, 136.05, 132.74, 132.72, 131.83, 129.59, 127.09, 122.83, 119.43, 114.01, 98.90, 82.33, 40.66, 24.69, 22.27, -0.46.

HRMS (ESI-QTOF) m/z: $[M + H]^+$ Calcd for $C_{23}H_{32}BrNO_2Si$: 462.1458; Found 462.1453

Compound **SI-33**

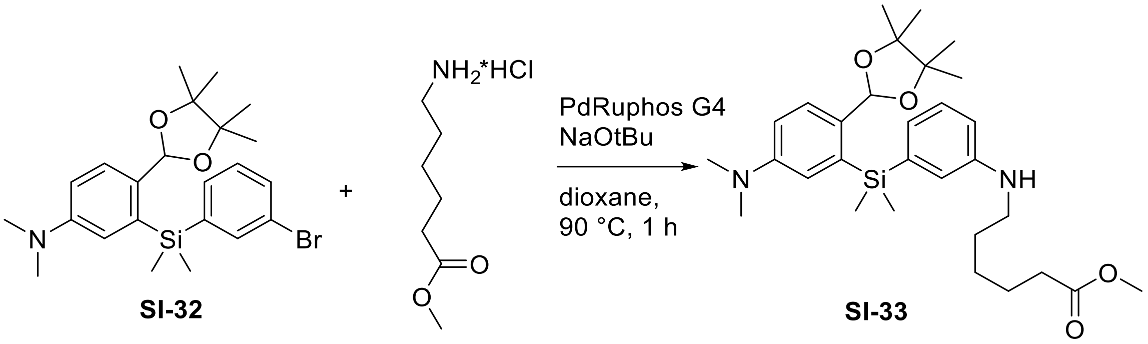

Compound **SI-32** (850 mg, 1 eq), Methyl 6-aminohexanoate hydrochloride (334 mg, 1 eq), Pd Ruphos G4 (78 mg, 0.05 eq) and NaOtBu (353 mg, 2 eq) was dissolved in 20 mL of dry dioxane in a flame dried flask filled with Argon. The mixture was stirred at 90 °C for 1 hour, the cooled down, the solvent evaporated and the product was purified with flash column chromatography (hexane:ethyl acetate 0 to 30% in 10 CV) to give 390 mg (40 %) of oil as product.

^{1}H NMR (400 MHz, $CDCl_3$) δ 7.60 (d, *J* = 8.6 Hz, 1H), 7.16 (t, *J* = 7.6 Hz, 1H), 6.92 – 6.83 (m, 2H), 6.79 (dd, *J* = 8.7, 2.8 Hz, 1H), 6.74 (d, *J* = 2.6 Hz, 1H), 6.58 (ddd, *J* = 8.1, 2.6, 1.1 Hz, 1H), 5.97 (s, 1H), 3.67 (s, 3H), 3.55 (bs, 1H), 3.09 (t, *J* = 7.1 Hz, 2H), 2.90 (s, 6H), 2.33 (t, *J* = 7.5 Hz, 2H), 1.74 – 1.64 (m, 2H), 1.64 – 1.54 (m, 2H), 1.47 – 1.37 (m, 2H), 1.26 (s, 6H), 1.16 (s, 6H), 0.58 (s, 6H).

^{13}C NMR (101 MHz, $CDCl_3$) δ 174.17, 150.14, 147.74, 140.21, 137.38, 132.45, 128.69, 127.09, 123.15, 119.65, 119.17, 113.88, 112.87, 99.03, 82.15, 51.61, 43.83, 40.66, 34.08, 29.36, 26.80, 24.85, 22.32, -0.32.

HRMS (ESI-QTOF) m/z: $[M + H]^+$ Calcd for $C_{30}H_{46}N_2O_4Si$: 527.3300; Found 527.3299

Compound **SI-34**

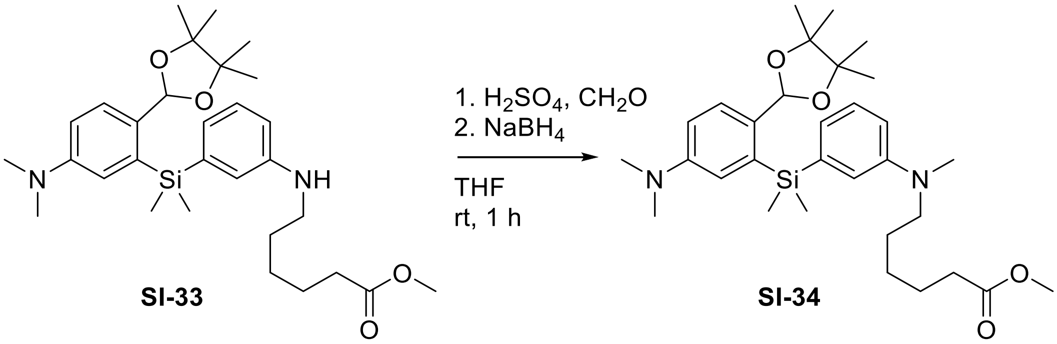

Compound **SI-34** was synthetized based on literature.[89] Aqueous formaldehyde (37%, 2 mL) was added to a mixture of THF (15 mL) and 3 M H_2SO_4 (2 mL) and stirred at room temperature for 10 minutes, then compound **SI-33** (390 mg) was added and the mixture was stirred for 1 hour. After 1 hour 1 g of $NaBH_4$ was slowly added to the flask while cooling it in an ice bath. The reaction mixture was diluted with ethyl acetate (50 mL) and washed with saturated $NaHCO_3$ solution (3*50 mL), the organic layer dried over Na_2SO_4 and evaporated. The product was purified with flash column chromatography (hexane:ethyl acetate 0 to 20% in 10 CV) to give 255 mg (64 %) of oil as product.

^{1}H NMR (400 MHz, $CDCl_3$) δ 7.59 (d, *J* = 8.7 Hz, 1H), 7.19 (dd, *J* = 8.3, 7.0 Hz, 1H), 6.88 – 6.81 (m, 3H), 6.77 (dd, *J* = 8.6, 2.8 Hz, 1H), 6.68 (ddd, *J* = 8.3, 2.8, 1.0 Hz, 1H), 5.98 (s, 1H), 3.66 (s, 3H), 3.30 – 3.21 (m, 2H), 2.91 – 2.85 (m, 9H), 2.30 (t, *J* = 7.5 Hz, 2H), 1.63 (p, *J* = 7.6 Hz, 2H), 1.54 (p, *J* = 7.6 Hz, 2H), 1.34 – 1.28 (m, 2H), 1.26 (s, 6H), 1.15 (s, 6H), 0.59 (s, 6H).

^{13}C NMR (101 MHz, $CDCl_3$) δ 174.25, 150.16, 148.71, 139.96, 137.54, 132.54, 128.60, 127.10, 122.20, 119.79, 118.11, 113.88, 113.11, 99.10, 82.17, 52.80, 51.61, 40.70, 38.49, 34.18, 26.84, 26.47, 24.98, 24.88, 22.37, -0.23.

HRMS (ESI-QTOF) m/z: $[M + H]^+$ Calcd for $C_{31}H_{48}N_2O_4Si$: 541.3456; Found 541.3448

Compound **SI-35 (PULI$_{640}$)**

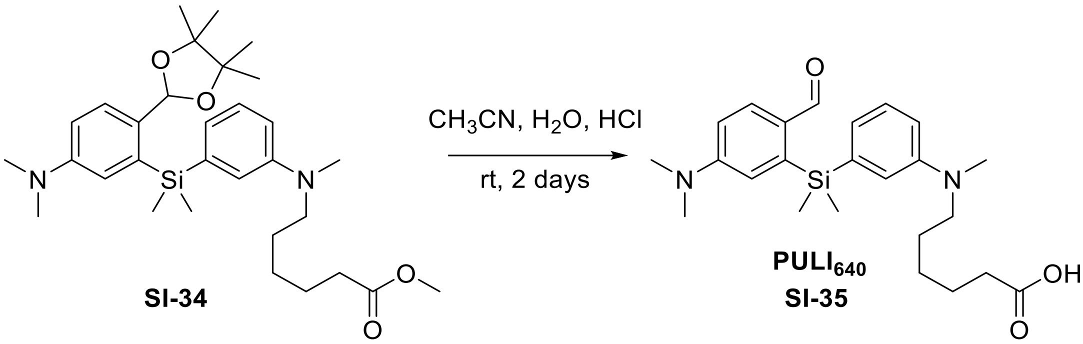

Compound **SI-34** (19 mg) was stirred in a mixture of 2 mL acetonitrile and 2 mL 1M HCl for 2 days at room temperature. After confirming full conversion with LC-MS, the solution was neutralized with 0.2 mL 10 M KOH and the product was purified with preparative HPLC (CH_3CN:H_2O 10-80% in 30 minutes) to give 10 mg (67 %) of product

^{1}H NMR (400 MHz, $CDCl_3$) δ 9.83 (s, 1H), 7.83 (d, *J* = 8.7 Hz, 1H), 7.20 (dd, *J* = 8.3, 7.1 Hz, 1H), 6.91 – 6.79 (m, 3H), 6.75 – 6.64 (m, 2H), 3.27 (t, *J* = 7.3 Hz, 2H), 3.01 (s, 6H), 2.88 (s, 3H), 2.33 (t, *J* = 7.4 Hz, 2H), 1.64 (p, *J* = 7.5 Hz, 2H), 1.55 (p, *J* = 7.5 Hz, 2H), 1.39 – 1.28 (m, 2H), 0.62 (s, 6H).

^{13}C NMR (101 MHz, $CDCl_3$) δ 191.23, 152.94, 148.75, 143.65, 139.72, 133.74, 129.69, 128.74, 122.11, 119.64, 117.99, 113.28, 111.40, 52.72, 40.00, 38.51, 26.71, 26.45, 24.74, -0.93.,

HRMS (ESI-QTOF) m/z: $[M + H]^+$ Calcd for $C_{25}H_{36}N_2O_3Si$: 427.2411; Found 427.2411

Compound **52 (PULI$_{640}$-M)**

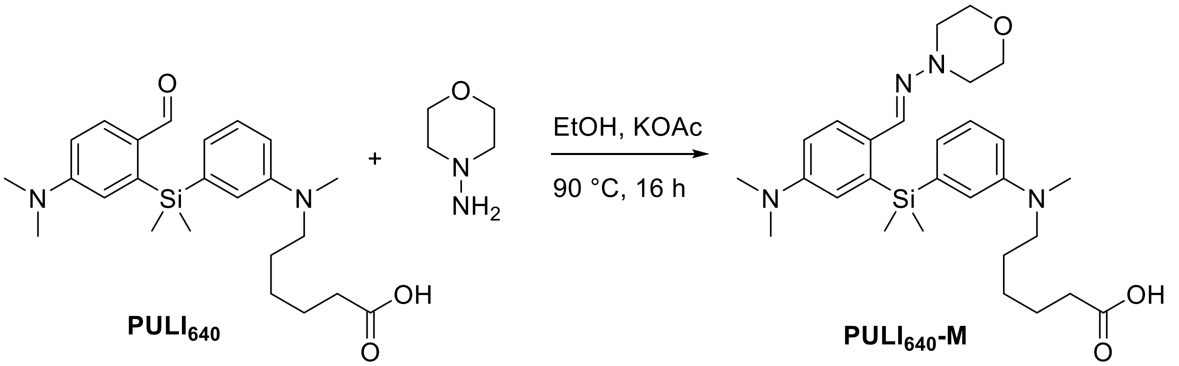

PULI$_{640}$ (10 mg, 1 eq), 4-Aminomorpholine (48 mg, 45 µL, 20 eq) and potassium acetate (32 mg, 14 eq) was dissolved in 1 mL of ethanol in a sealed screw cap vial and stirred at 90°C for 16 hours. Then the solution was diluted with ethyl acetate (10 mL) and washed with water (3*10 mL), the organic layer dried over Na_2SO_4 and evaporated to give 11 mg (92 %) of product.

^{1}H NMR (400 MHz, $CDCl_3$) δ 7.83 (d, *J* = 8.7 Hz, 1H), 7.65 (s, 1H), 7.15 (dd, *J* = 8.3, 7.1 Hz, 1H), 6.93 (d, *J* = 2.8 Hz, 1H), 6.84 – 6.73 (m, 3H), 6.64 (dd, *J* = 8.1, 2.7 Hz, 1H), 3.86 – 3.82 (m, 2H), 3.73 – 3.69 (m, 4H), 3.24 – 3.16 (m, 2H), 2.97 (s, 6H), 2.82 (s, 3H), 2.75 – 2.69 (m, 4H), 2.21 (t, *J* = 7.3 Hz, 2H), 1.58 (p, *J* = 7.3 Hz, 2H), 1.49 (p, *J* = 7.5 Hz, 2H), 0.55 (s, 6H).

^{13}C NMR (101 MHz, $CDCl_3$) δ 149.89, 148.89, 141.13, 140.09, 137.88, 136.70, 129.44, 128.96, 126.42, 121.70, 119.05, 117.52, 114.06, 113.04, 66.50, 52.74, 52.42, 51.73, 40.58, 38.42, 26.95, 26.59, 25.40, -0.73.

HRMS (ESI-QTOF) m/z: $[M + H]^+$ Calcd for $C_{28}H_{42}N_4O_3Si$: 511.3099; Found 511.3091

Compound **53 (PULI$_{640}$-P)**

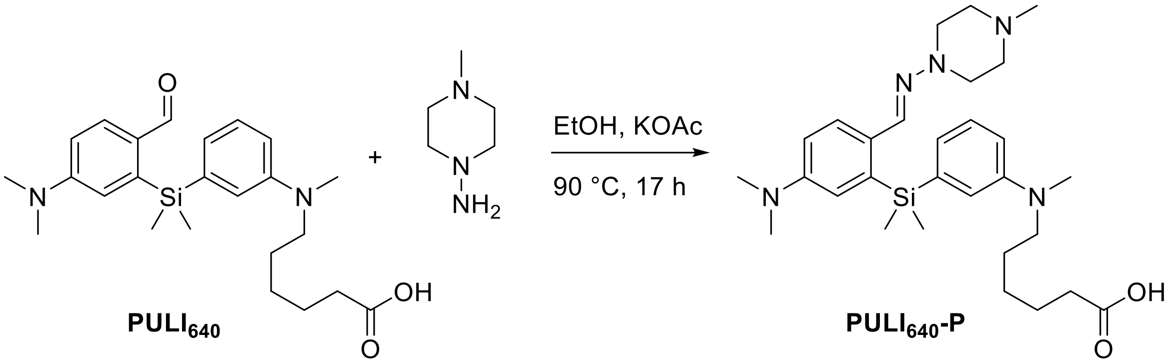

PULI$_{640}$ (15 mg, 1 eq), 1-Amino-4-methylpiperazin (81 mg, 85 µL, 20 eq) and potassium acetate (35 mg, 10 eq) was dissolved in 1 mL of ethanol in a sealed screw cap vial and stirred at 90°C for 17 hours. Then the solution was diluted with ethyl acetate (10 mL) and washed with water (3*10 mL), the organic layer dried over Na_2SO_4 and evaporated, then purified with preparative HPLC (CH_3CN:H_2O 10-80% in 30 minutes) to give 16 mg (87 %) of product.

^{1}H NMR (400 MHz, $CDCl_3$) δ 8.18 (bs, 1H), 7.85 (d, *J* = 8.8 Hz, 1H), 7.58 (s, 1H), 7.17 (dd, *J* = 8.2, 7.1 Hz, 1H), 6.97 (d, *J* = 2.8 Hz, 1H), 6.84 – 6.74 (m, 2H), 6.74 – 6.64 (m, 2H), 3.26 (t, *J* = 6.5 Hz, 2H), 3.01 (s, 6H), 2.84 – 2.74 (m, 7H), 2.73 – 2.66 (m, 4H), 2.38 (s, 3H), 2.29 – 2.23 (m, 2H), 1.66 (p, *J* = 7.0 Hz, 2H), 1.62 – 1.51 (m, 2H), 1.43 – 1.30 (m, 2H), 0.54 (s, 6H).

^{13}C NMR (101 MHz, $CDCl_3$) δ 177.73, 149.86, 149.04, 140.81, 140.14, 137.53, 129.34, 128.82, 126.36, 120.91, 118.68, 117.21, 113.89, 112.71, 53.23, 52.54, 49.70, 44.25, 40.51, 38.19, 35.29, 27.19, 27.01, 25.39, -0.54.

HRMS (ESI-QTOF) m/z: $[M + H]^+$ Calcd for $C_{29}H_{45}N_5O_2Si$: 524.3415; Found 524.3417

Compound **54 (PULI$_{640}$-T)**

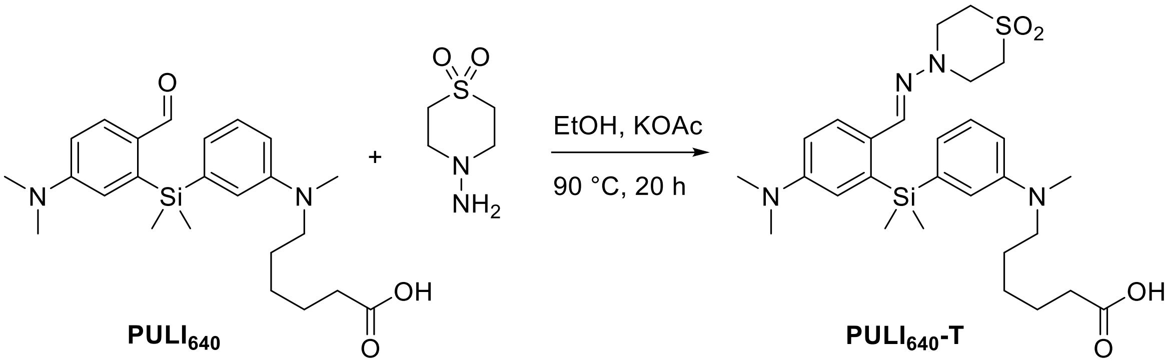

PULI$_{640}$ (11 mg, 1 eq), 4-Aminothiomorpholine 1,1-Dioxide (39 mg, 10 eq) and potassium acetate (25 mg, 10 eq) was dissolved in 1 mL of ethanol in a sealed screw cap vial and stirred at 90°C for 20 hours. Then the solution was diluted with ethyl acetate (10 mL) and washed with water (3*10 mL), the organic layer dried over Na_2SO_4 and evaporated to give 14 mg (97 %) of product.

^{1}H NMR (400 MHz, $CDCl_3$) δ 7.86 (d, *J* = 8.8 Hz, 1H), 7.63 (s, 1H), 7.18 (dd, *J* = 8.3, 7.1 Hz, 1H), 6.97 (d, *J* = 2.8 Hz, 1H), 6.81 – 6.74 (m, 2H), 6.73 (d, *J* = 2.8 Hz, 1H), 6.65 (ddd, *J* = 8.4, 2.8, 1.0 Hz, 1H), 3.51 – 3.43 (m, 4H), 3.24 – 3.20 (m, 2H), 3.03 (s, 6H), 2.87 (s, 3H), 2.69 – 2.57 (m, 4H), 2.31 (t, *J* = 7.3 Hz, 2H), 1.66 – 1.56 (m, 2H), 1.56 – 1.46 (m, 2H), 1.36 – 1.26 (m, 2H), 0.54 (s, 6H).

^{13}C NMR (101 MHz, $CDCl_3$) δ 177.79, 150.22, 149.01, 144.78, 140.34, 138.22, 129.39, 128.08, 126.34, 121.05, 118.76, 116.91, 113.89, 113.05, 52.59, 49.29, 47.71, 40.48, 38.36, 33.96, 26.69, 26.42, 24.76, -0.78.

HRMS (ESI-QTOF) m/z: $[M + H]^+$ Calcd for $C_{28}H_{42}N_4O_4SSi$: 559.2769; Found 559.2750

Compound **55 (PULI$_{640}$-M-NHS)**

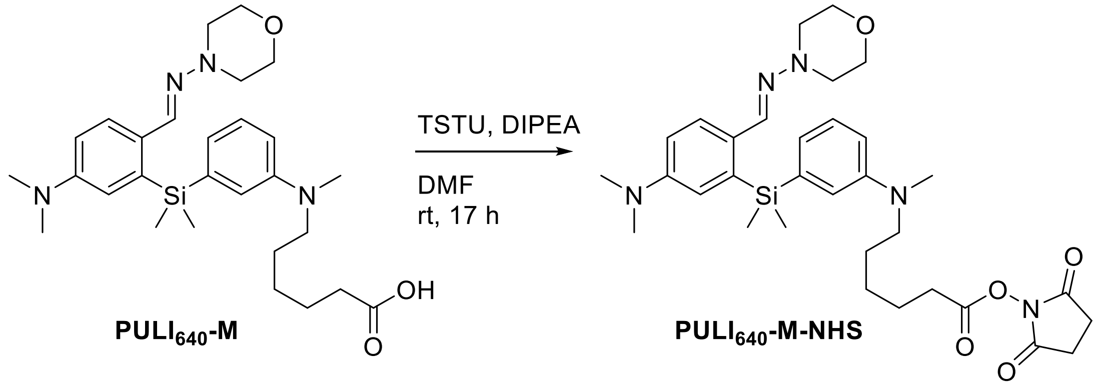

PULI$_{640}$-M (10 mg, 1 eq), TSTU (N,N,N′,N′-Tetramethyl-O-(N-succinimidyl)uroniumtetrafluorborat, 12 mg, 2 eq), and DIPEA (N,N-Diisopropylethylamine, 5 mg, 7 µL, 2 eq) was dissolved in 1 mL of dry DMF in a closed cap vial and stirred at room temperature for 17 hours. Next day the compound was diluted with acetonitrile and purified with preparative HPLC ($CH_3CN:H_2O$ 10-80% in 30 minutes) to give 3.3 mg (28 %) of product.

^{1}H NMR (400 MHz, $CDCl_3$) δ 7.83 (d, *J* = 8.7 Hz, 1H), 7.65 (s, 1H), 7.19 (t, *J* = 7.7 Hz, 1H), 6.91 (s, 1H), 6.84 (d, *J* = 6.6 Hz, 1H), 6.81 – 6.74 (m, 2H), 6.66 (d, *J* = 6.3 Hz, 1H), 3.73 (t, *J* = 4.9 Hz, 4H), 3.24 (t, *J* = 7.5 Hz, 2H), 2.98 (s, 6H), 2.87 (s, 3H), 2.85 – 2.80 (m, 4H), 2.76 (t, *J* = 4.9 Hz, 4H), 2.58 (t, *J* = 7.4 Hz, 2H), 1.73 (p, *J* = 7.4 Hz, 3H), 1.56 – 1.48 (m, 2H), 1.40 – 1.31 (m, 2H), 0.56 (s, 6H).

^{13}C NMR (101 MHz, $CDCl_3$) δ 169.26, 168.65, 140.23, 128.96, 126.58, 121.58, 118.93, 117.79, 113.83, 113.18, 108.13, 66.62, 52.36, 40.51, 31.08, 31.04, 26.41, 25.74, 24.61, -0.72.

HRMS (ESI-QTOF) m/z: $[M + H]^+$ Calcd for $C_{32}H_{45}N_5O_5Si$: 608.3263; Found 608.3270

Compound **56 (PULI$_{640}$-P-NHS)**

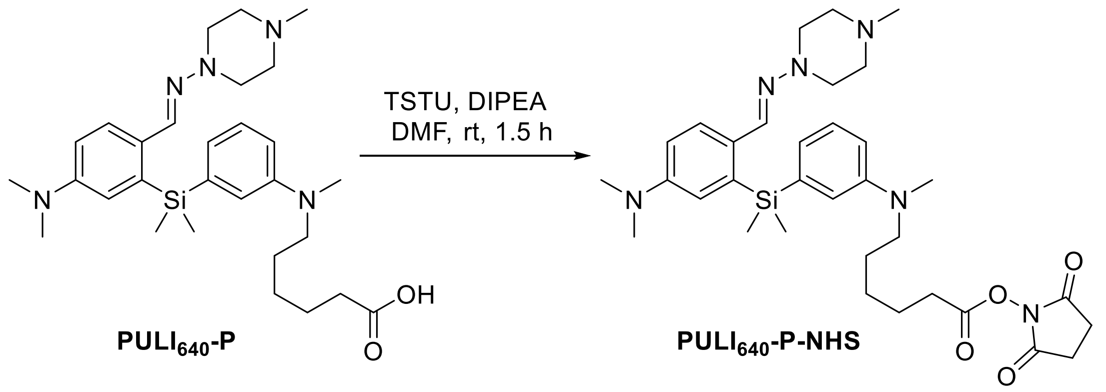

PULI$_{640}$-P (1.6 mg, 1 eq) TSTU (N,N,N′,N′-Tetramethyl-O-(N-succinimidyl)uroniumtetrafluorborat, 1.4 mg, 1.5 eq), and DIPEA (N,N-Diisopropylethylamine, 0.6 mg, 0.8 µL, 1.5 eq) was dissolved in 1 mL of dry DMF in a closed cap vial and stirred at room temperature for 1.5 hours. Then the compound was diluted with acetonitrile and purified with preparative HPLC ($CH_3CN:H_2O$ 10-80% in 30 minutes) to give 1.5 mg (79 %) of product.

^{1}H NMR (400 MHz, $CDCl_3$) δ 7.82 (d, *J* = 8.8 Hz, 1H), 7.62 (s, 1H), 7.18 (dd, *J* = 8.3, 7.0 Hz, 1H), 6.90 (d, *J* = 2.8 Hz, 1H), 6.83 (d, *J* = 7.1 Hz, 1H), 6.80 – 6.73 (m, 2H), 6.65 (dd, *J* = 8.4, 2.6 Hz, 1H), 3.23 (t, *J* = 7.4 Hz, 2H), 2.97 (s, 6H), 2.96 – 2.92 (m, 4H), 2.87 (s, 3H), 2.86 – 2.80 (m, 4H), 2.61 – 2.55 (m, 6H), 2.37 (s, 3H), 2.30 – 2.23 (m, 2H), 1.78 – 1.66 (m, 2H), 1.58 – 1.46 (m, 2H), 1.41 – 1.30 (m, 2H), 0.55 (s, 6H).

^{13}C NMR (101 MHz, $CDCl_3$) δ 169.29, 168.68, 149.84, 148.81, 140.17, 137.65, 129.39, 128.99, 126.44, 121.69, 118.91, 117.67, 113.84, 113.11, 53.45, 52.61, 50.48, 49.56, 45.13, 40.52, 40.50, 38.51, 31.04, 26.42, 26.33, 25.75, 24.63, -0.69.

HRMS (ESI-QTOF) m/z: $[M + H]^+$ Calcd for $C_{33}H_{48}N_6O_4Si$: 621.3579; Found 621.3597

Compound **57 (PULI$_{640}$-T-NHS)**

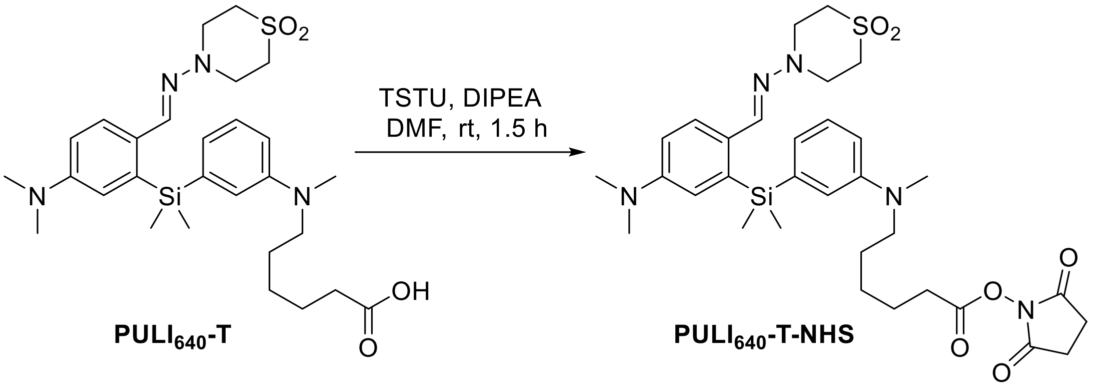

PULI$_{640}$-T (6 mg, 1 eq) TSTU (N,N,N′,N′-Tetramethyl-O-(N-succinimidyl)uroniumtetrafluorborat, 4.9 mg, 1.5 eq), and DIPEA (N,N-Diisopropylethylamine, 2.1 mg, 2.8 µL, 1.5 eq) was dissolved in 1 mL of dry DMF in a closed cap vial and stirred at room temperature for 1.5 hours. Then the compound was diluted with acetonitrile and purified with preparative HPLC (CH_3CN:H_2O 10-80% in 30 minutes) to give 3.2 mg (45 %) of product.

^{1}H NMR (400 MHz, $CDCl_3$) δ 7.86 (d, *J* = 8.8 Hz, 1H), 7.60 (s, 1H), 7.19 (dd, *J* = 8.3, 7.1 Hz, 1H), 6.96 (d, *J* = 2.8 Hz, 1H), 6.83 – 6.75 (m, 2H), 6.71 (d, *J* = 2.6 Hz, 1H), 6.67 (dd, *J* = 8.2, 2.7 Hz, 1H), 3.56 – 3.45 (m, 4H), 3.29 – 3.20 (m, 2H), 3.03 (s, 6H), 2.88 (s, 3H), 2.86 – 2.78 (m, 4H), 2.63 – 2.56 (m, 6H), 1.72 (p, *J* = 7.4 Hz, 2H), 1.54 – 1.47 (m, 2H), 1.39 – 1.30 (m, 2H), 0.54 (s, 6H).

^{13}C NMR (101 MHz, $CDCl_3$) δ 169.32, 168.65, 150.15, 143.69, 140.34, 138.02, 129.42, 128.09, 126.33, 121.07, 118.57, 117.01, 113.74, 113.20, 52.49, 49.14, 47.77, 40.43, 38.49, 31.05, 26.35, 26.27, 25.75, 24.66, -0.70.

HRMS (ESI-QTOF) m/z: $[M + H]^+$ Calcd for $C_{32}H_{45}N_5O_6SSi$: 656.2933; Found 656.2916

Compound **58 (PULI$_{640}$-M-Halo)**

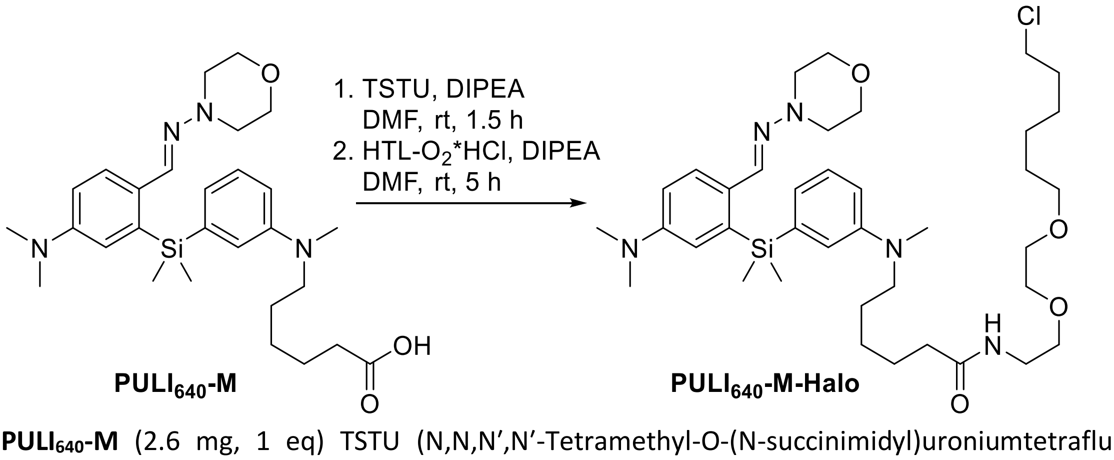

PULI$_{640}$-M (2.6 mg, 1 eq) TSTU (N,N,N′,N′-Tetramethyl-O-(N-succinimidyl)uroniumtetrafluorborat, 2.3 g, 1.5 eq), and DIPEA (N,N-Diisopropylethylamine, 1 mg, 1.3 µL, 1.5 eq) was dissolved in 1 mL of dry DMF in a closed cap vial and stirred at room temperature for 1.5 hours, then to the solution HaloTag ligand (2-(2-((6-Chlorohexyl)oxy)ethoxy)ethan-1-amine hydrochloride) (2.7 mg, 2 eq) and DIPEA (1.3 mg, 1.8 µL, 2 eq) was added and the solution was further stirred at room temperature for 5 hours. The solution was then diluted with acetonitrile and purified with preparative HPLC (CH_3CN:H_2O 10-80% in 30 minutes) to give 1.8 mg (49 %) of product.

^{1}H NMR (400 MHz, $CDCl_3$) δ 7.83 (d, *J* = 8.7 Hz, 1H), 7.65 (s, 1H), 7.17 (dd, *J* = 8.3, 7.1 Hz, 1H), 6.91 (d, *J* = 2.8 Hz, 1H), 6.82 (d, *J* = 7.1 Hz, 1H), 6.80 – 6.73 (m, 2H), 6.66 (dd, *J* = 8.2, 2.7 Hz, 1H), 6.04 (t, *J* = 5.8 Hz, 1H), 3.77 – 3.69 (m, 4H), 3.62 – 3.50 (m, 8H), 3.46 (t, *J* = 6.5 Hz, 4H), 3.23 (t, *J* = 7.5 Hz, 2H), 2.98 (s, 6H), 2.86 (s, 3H), 2.78 – 2.70 (m, 4H), 2.13 (t, *J* = 7.6 Hz, 2H), 1.81 – 1.72 (m, 2H), 1.67 – 1.60 (m, 4H), 1.54 – 1.48 (m, 2H), 1.48 – 1.40 (m, 2H), 1.42 – 1.34 (m, 2H), 1.31 – 1.25 (m, 2H), 0.55 (s, 6H).

^{13}C NMR (101 MHz, $CDCl_3$) δ 173.04, 149.82, 148.84, 140.18, 140.07, 137.77, 129.44, 128.93, 126.36, 121.74, 118.88, 117.62, 113.88, 113.09, 71.42, 70.41, 70.16, 70.09, 66.64, 52.70, 52.36, 45.17, 40.52, 39.26, 38.49, 36.69, 32.66, 29.60, 26.94, 26.82, 26.52, 25.66, 25.56, -0.72.

HRMS (ESI-QTOF) m/z: $[M + H]^+$ Calcd for $C_{38}H_{62}ClN_5O_4Si$: 716.4332; Found 716.4339

Compound **59 (PULI$_{640}$-P-Halo)**

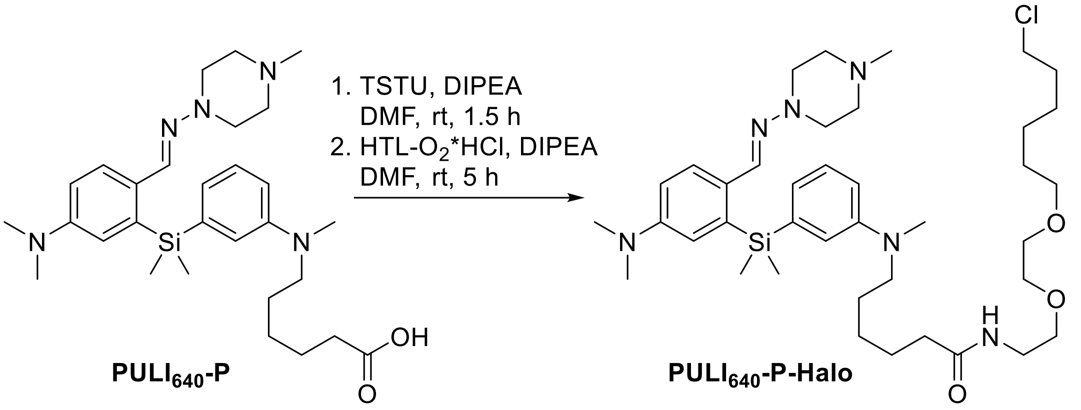

PULI$_{640}$-P (1.6 mg, 1 eq) TSTU (N,N,N',N'-Tetramethyl-O-(N-succinimidyl)uroniumtetrafluorborat, 1.4 mg, 1.5 eq), and DIPEA (N,N-Diisopropylethylamine, 0.6 mg, 0.8 µL, 1.5 eq) was dissolved in 1 mL of dry DMF in a closed cap vial and stirred at room temperature for 1.5 hours, then to the solution HaloTag ligand (2-(2-((6-Chlorohexyl)oxy)ethoxy)ethan-1-amine hydrochloride) (1.6 mg, 2 eq) and DIPEA (0.8 mg, 1.1 µL, 2 eq) was added and the solution was further stirred at room temperature for 5 hours. The solution then was diluted with acetonitrile and purified with preparative HPLC (CH_3CN:H_2O 10-80% in 30 minutes) to give 1.0 mg (44 %) of product.

^{1}H NMR (400 MHz, $CDCl_3$) δ 7.83 (d, *J* = 8.8 Hz, 1H), 7.62 (s, 1H), 7.17 (dd, *J* = 8.3, 7.1 Hz, 1H), 6.90 (d, *J* = 2.8 Hz, 1H), 6.82 (d, *J* = 7.1 Hz, 1H), 6.78 (d, *J* = 2.7 Hz, 1H), 6.76 (dd, *J* = 8.8, 2.7 Hz, 1H), 6.65 (dd, *J* = 8.0, 2.8 Hz, 1H), 6.11 (s, 1H), 3.62 – 3.50 (m, 8H), 3.46 (t, *J* = 6.6 Hz, 4H), 3.23 (t, *J* = 7.5 Hz, 2H), 2.97 (s, 6H), 2.92 – 2.75 (m, 7H), 2.48 (s, 4H), 2.31 (s, 2H), 2.13 (t, *J* = 7.6 Hz, 2H), 2.01 (s, 3H), 1.81 – 1.72 (m, 2H), 1.51 – 1.24 (m, 10H), 0.55 (s, 6H).

^{13}C NMR (101 MHz, $CDCl_3$) δ 173.07, 149.77, 148.85, 128.94, 126.30, 121.73, 118.93, 117.57, 113.92, 113.09, 71.42, 70.41, 70.16, 70.10, 52.72, 45.17, 40.54, 39.27, 38.50, 36.70, 32.66, 29.60, 26.96, 26.83, 26.54, 25.66, 25.56, 2.05, -0.73.

HRMS (ESI-QTOF) m/z: $[M + H]^+$ Calcd for $C_{39}H_{65}ClN_6O_3Si$: 729.4649; Found 729.4663

Compound **60 (PULI$_{640}$-T-Halo)**

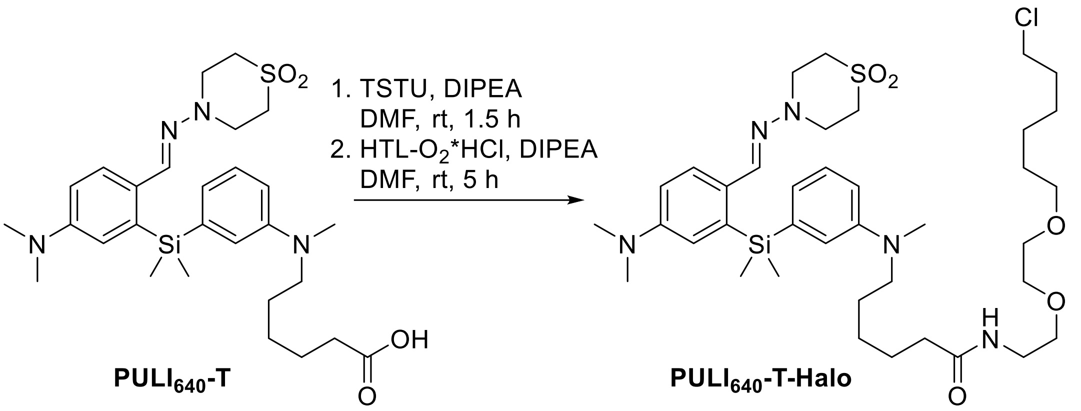

PULI$_{640}$-T (6 mg, 1 eq) TSTU (N,N,N′,N′-Tetramethyl-O-(N-succinimidyl)uroniumtetrafluorborat, 4.9 mg, 1.5 eq), DIPEA (N,N-Diisopropylethylamine, 4.1 mg, 5.6 µL, 1.5 eq) and HaloTag ligand (2-(2-((6-Chlorohexyl)oxy)ethoxy)ethan-1-amine hydrochloride) (4.2 mg, 1.5 eq) was dissolved in 1 mL of dry DMF in a closed cap vial and stirred at room temperature for 22 hours. The solution then was diluted with acetonitrile and purified with preparative HPLC (CH_3CN:H_2O 10-80% in 30 minutes) to give 3.5 mg (43 %) of product.

^{1}H NMR (400 MHz, $CDCl_3$) δ 7.85 (d, *J* = 8.8 Hz, 1H), 7.61 (s, 1H), 7.18 (dd, *J* = 8.3, 7.1 Hz, 1H), 6.96 (d, *J* = 2.8 Hz, 1H), 6.81 – 6.75 (m, 2H), 6.72 (d, *J* = 2.7 Hz, 1H), 6.66 (ddd, *J* = 8.4, 2.8, 1.0 Hz, 1H), 6.08 (s, 1H), 3.62 – 3.42 (m, 16H), 3.26 – 3.19 (m, 2H), 3.03 (s, 6H), 2.87 (s, 3H), 2.65 – 2.55 (m, 4H), 2.19 – 2.11 (m, 2H), 1.81 – 1.72 (m, 2H), 1.68 – 1.63 (m, 2H), 1.53 – 1.32 (m, 6H), 1.32 – 1.25 (m, 2H), 0.54 (s, 6H).

^{13}C NMR (101 MHz, $CDCl_3$) δ 173.05, 150.17, 149.00, 143.93, 140.43, 138.11, 129.39, 128.02, 126.29, 121.10, 118.54, 116.77, 113.74, 113.13, 71.40, 70.38, 70.16, 70.06, 52.59, 49.19, 47.76, 45.18, 40.44, 39.25, 38.47, 36.66, 32.66, 29.60, 26.90, 26.82, 26.55, 25.70, 25.56, -0.73.

HRMS (ESI-QTOF) m/z: $[M + H]^+$ Calcd for $C_{38}H_{62}ClN_5O_5SSi$: 764.4002; Found 764.4006

Compound **PULI$_{640}$-M-SNAP**

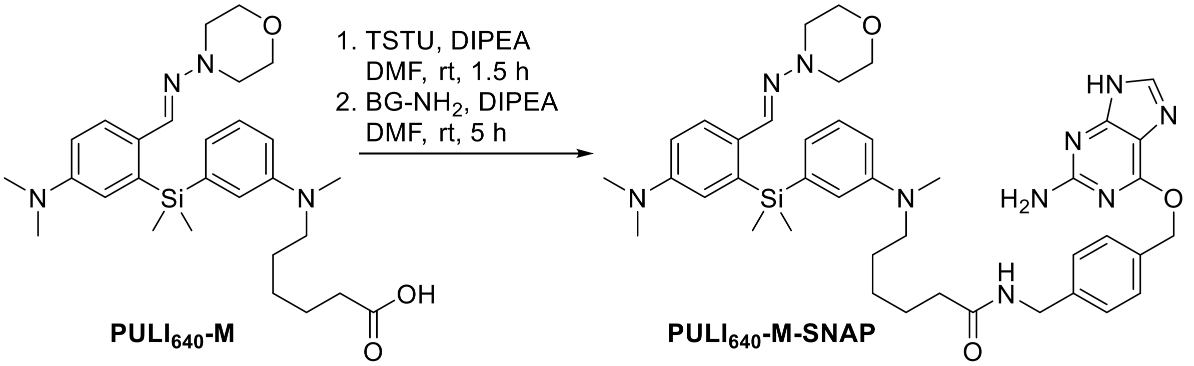

PULI$_{640}$-M (2.6 mg, 1 eq) TSTU (N,N,N′,N′-Tetramethyl-O-(N-succinimidyl)uroniumtetrafluorborat, 2.3 mg, 1.5 eq), and DIPEA (N,N-Diisopropylethylamine, 1 mg, 1.3 µL, 1.5 eq) was dissolved in 1 mL of dry DMF in a closed cap vial and stirred at room temperature for 1.5 hours, then to the solution BG-NH_2 (O6-(4-Aminomethyl-benzyl)guanine) (2.8 mg, 2 eq) and DIPEA (1.3 mg, 1.8 µL, 2 eq) was added and the solution was further stirred at room temperature for 5 hours. The solution then was diluted with acetonitrile and purified with preparative HPLC (CH_3CN:H_2O 10-80% in 30 minutes) to give 1.5 mg (39 %) of product.

^{1}H NMR (400 MHz, $CDCl_3$) δ 7.81 (d, *J* = 8.7 Hz, 1H), 7.65 (s, 1H), 7.40 (d, *J* = 7.6 Hz, 2H), 7.29 (d, *J* = 8.0 Hz, 2H), 7.16 (dd, *J* = 8.3, 7.1 Hz, 1H), 6.92 (d, *J* = 2.8 Hz, 1H), 6.82 (d, *J* = 7.1 Hz, 1H), 6.77 (d, *J* = 2.7 Hz, 1H), 6.74 (dd, *J* = 8.8, 2.8 Hz, 1H), 6.63 (dd, *J* = 8.2, 2.6 Hz, 1H), 5.38 (s, 2H), 4.85 (s, 2H), 4.45 (d, *J* = 5.3 Hz, 2H), 3.67 (t, *J* = 1.9 Hz, 4H), 3.20 (t, *J* = 7.4 Hz, 2H), 2.97 (s, 6H), 2.81 (s, 3H), 2.70 (t, *J* = 4.8 Hz, 4H), 2.28 (t, *J* = 7.5 Hz, 2H), 1.69 – 1.65 (m, 2H), 1.52 – 1.42 (m, 2H), 1.31 – 1.25 (m, 2H), 0.55 (s, 6H).

^{13}C NMR (101 MHz, $CDCl_3$) δ 149.88, 148.87, 140.06, 139.47, 137.94, 129.24, 128.96, 126.35, 121.64, 118.87, 117.67, 113.87, 113.10, 68.70, 66.56, 52.64, 52.33, 43.83, 40.51, 38.44, 36.35, 29.85, 26.80, 26.34, 25.76, -0.72.

HRMS (ESI-QTOF) m/z: $[M + H]^+$ Calcd for $C_{41}H_{54}N_{10}O_3Si$: 763.4222; Found 763.4241

Compound **PULI$_{640}$-P-SNAP**

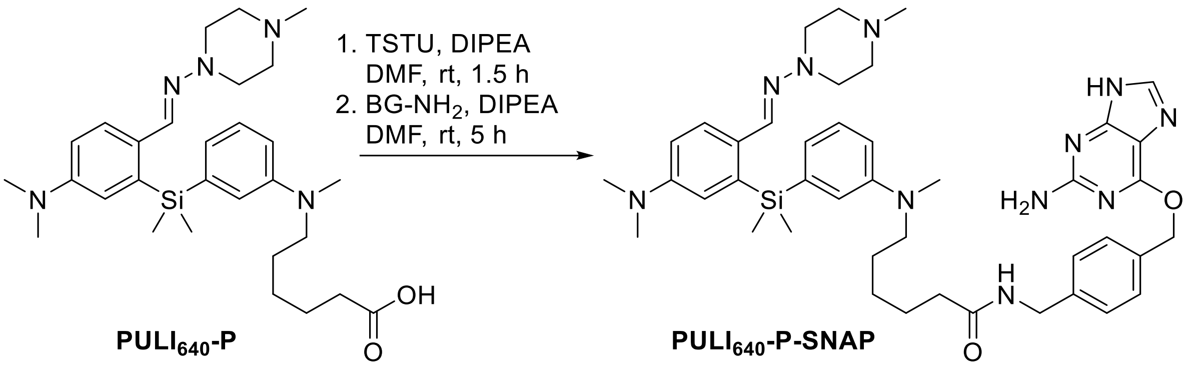

PULI$_{640}$-P (1.6 mg, 1 eq) TSTU (N,N,N′,N′-Tetramethyl-O-(N-succinimidyl)uroniumtetrafluorborat, 1.4 mg, 1.5 eq), and DIPEA (N,N-Diisopropylethylamine, 0.6 mg, 0.8 µL, 1.5 eq) was dissolved in 1 mL of dry DMF in a closed cap vial and stirred at room temperature for 1.5 hours, then to the solution BG-NH_2 (O6-(4-Aminomethyl-benzyl)guanine) (1.7 mg, 2 eq) and DIPEA (0.8 mg, 1.1 µL, 2 eq) was added and the solution was further stirred at room temperature for 5 hours. The solution then was diluted with acetonitrile and purified with preparative HPLC (CH_3CN:H_2O 10-80% in 30 minutes) to give 0.8 mg (34 %) of product.

^{1}H NMR (400 MHz, CD_3CN) δ 7.70 (d, *J* = 8.8 Hz, 2H), 7.67 (s, 1H), 7.54 (s, 1H), 7.44 (d, *J* = 7.8 Hz, 2H), 7.28 (d, *J* = 7.7 Hz, 2H), 7.13 (t, *J* = 7.7 Hz, 1H), 7.04 (t, *J* = 6.1 Hz, 1H), 6.94 (d, *J* = 2.8 Hz, 1H), 6.78 (dd, *J* = 8.8, 2.8 Hz, 1H), 6.76 – 6.68 (m, 2H), 6.66 – 6.59 (m, 1H), 5.46 (s, 2H), 5.26 (s, 2H), 4.31 (d, *J* = 6.0 Hz, 2H), 3.19 (t, *J* = 7.3 Hz, 2H), 2.96 (s, 6H), 2.87 – 2.80 (m, 4H), 2.80 (s, 3H), 2.79 – 2.74 (m, 4H), 2.47 (s, 3H), 2.14 (t, *J* = 7.4 Hz, 2H), 1.58 – 1.50 (m, 2H), 1.47 – 1.37 (m, 2H), 1.23 – 1.12 (m, 2H), 0.51 (s, 6H).

^{13}C NMR (101 MHz, CD_3CN) δ 173.96, 161.02, 160.86, 151.05, 149.80, 142.16, 141.09, 140.91, 139.20, 138.52, 136.48, 129.96, 129.86, 129.46, 128.58, 127.29, 121.76, 119.57, 114.43, 113.76, 68.39, 56.59, 53.36, 52.91, 49.96, 47.38, 44.12, 43.37, 40.48, 38.71, 36.69, 36.11, 28.88, 27.32, 26.93, 26.44, -0.72.

HRMS (ESI-QTOF) m/z: $[M + H]^+$ Calcd for $C_{42}H_{57}N_{11}O_2Si$: 776.4539; Found 776.4566

Compound **SI-36**

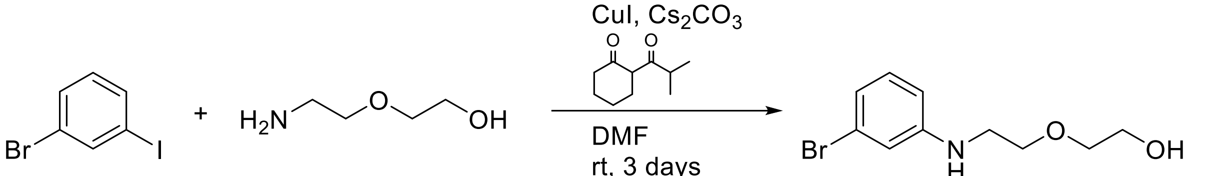

SI-36

Compound **SI-36** was synthetized based on literature.[92] 3-Bromoiodobenzene (1 g, 0.45 mL, 1 eq), 2-(2-Aminoethoxy)ethanol (0.56 g, 0.53 mL, 1.5 eq), CuI (33 mg, 0.05 eq), 2-Isobutyrylcyclohexanone (118 mg, 0.118 mL, 0.2 eq) and Cs_2CO_3 (2.3 g, 2 eq) was dissolved in 10 mL of dry DMF in a flame dried flask filled with Argon and stirred at room temperature for 3 days. After 3 days the mixture was diluted with ethyl acetate (50 mL) and washed with water (3*50 mL), the organic layer dried over Na_2SO_4 and evaporated. The product was purified with flash column chromatography (hexane:ethyl acetate 20 to 80% in 10 CV) to give 659 mg (72 %) of oil as product.

^{1}H NMR (400 MHz, $CDCl_3$) δ 7.02 (t, *J* = 8.0 Hz, 1H), 6.84 (ddd, *J* = 7.8, 1.8, 0.9 Hz, 1H), 6.79 (t, *J* = 2.1 Hz, 1H), 6.57 (ddd, *J* = 8.2, 2.4, 0.9 Hz, 1H), 3.84 – 3.73 (m, 2H), 3.76 – 3.67 (m, 3H), 3.64 – 3.58 (m, 2H), 3.33 – 3.23 (m, 2H).

^{13}C NMR (101 MHz, $CDCl_3$) δ 149.28, 130.65, 123.43, 120.81, 115.90, 112.23, 72.33, 69.50, 61.95, 43.75.

HRMS (ESI-QTOF) m/z: $[M + H]^+$ Calcd for $C_{10}H_{14}BrNO_2$: 260.0281; Found 260.0290

Compound **SI-37**

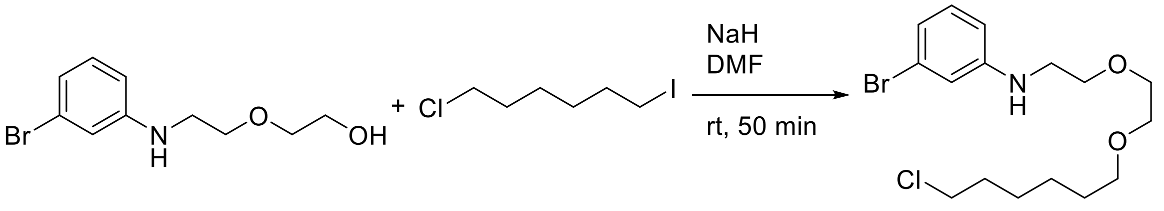

SI-36 **SI-37**

Compound **SI-36** (535 mg, 1 eq) and 1-Chloro-6-iodohexane (1.52 g, 0.937 mL, 3 eq) was dissolved in 5 mL of dry DMF. Slowly NaH (60 % suspension, 247 g, 3 eq) was added to the solution and it was stirred for 50 minutes. After full conversion was confirmed by LC-MS, the reaction mixture was diluted with ethyl acetate (50 mL) and washed with water (3*50 mL), the organic layer dried over Na_2SO_4 and evaporated. The product was purified with flash column chromatography (hexane:ethyl acetate 0 to 30% in 10 CV) to give 665 mg (85 %) of oil as product.

^{1}H NMR (400 MHz, $CDCl_3$) δ 7.02 (t, *J* = 8.0 Hz, 1H), 6.83 (ddd, *J* = 7.9, 1.8, 0.9 Hz, 1H), 6.78 (t, *J* = 2.1 Hz, 1H), 6.56 (ddd, *J* = 8.2, 2.3, 0.9 Hz, 1H), 3.71 (dd, *J* = 5.7, 4.8 Hz, 2H), 3.67 – 3.62 (m, 2H), 3.61 – 3.56 (m, 2H), 3.53 (t, *J* = 6.7 Hz, 2H), 3.47 (t, *J* = 6.6 Hz, 2H), 3.27 (dd, *J* = 5.7, 4.8 Hz, 2H), 1.83 – 1.72 (m, 2H), 1.65 – 1.57 (m, 2H), 1.50 – 1.42 (m, 2H), 1.42 – 1.33 (m, 2H).

^{13}C NMR (101 MHz, $CDCl_3$) δ 149.29, 130.61, 123.40, 120.76, 116.02, 112.33, 71.47, 70.49, 70.20, 69.31, 45.20, 43.75, 32.67, 29.63, 26.84, 25.59.

HRMS (ESI-QTOF) m/z: $[M + H]^+$ Calcd for $C_{16}H_{25}BrClNO_2$: 380.0808; Found 380.0822

Compound **SI-38**

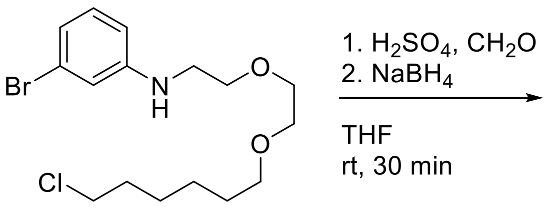

SI-37 **SI-38**

Compound **SI-38** was synthetized based on literature.[89] Aqueous formaldehyde (37%, 0.5 mL) was added to a mixture of THF (10 mL) and 3 M H_2SO_4 (1 mL) and stirred at room temperature for 10 minutes, then the solution was added to 455 mg of compound **SI-36** and the mixture was stirred for 30 minutes. After 30 minutes 250 mg of $NaBH_4$ was slowly added to the flask while cooling it in an ice bath. The reaction mixture was diluted with DCM (50 mL) and washed with saturated $NaHCO_3$ solution (3*50 mL), the organic layer dried over Na_2SO_4 and evaporated. The product was purified with flash column chromatography (hexane:ethyl acetate 0 to 20% in 10 CV) to give 386 mg (82 %) of oil as product.

^{1}H NMR (400 MHz, $CDCl_3$) δ 7.05 (t, *J* = 8.1 Hz, 1H), 6.89 – 6.82 (m, 1H), 6.80 (dd, *J* = 7.7, 1.6 Hz, 1H), 6.67 – 6.58 (m, 1H), 3.64 (t, *J* = 6.2 Hz, 2H), 3.62 – 3.57 (m, 2H), 3.57 – 3.49 (m, 6H), 3.45 (t, *J* = 6.6 Hz, 2H), 2.97 (s, 3H), 1.83 – 1.71 (m, 2H), 1.65 – 1.55 (m, 2H), 1.49 – 1.40 (m, 2H), 1.40 – 1.31 (m, 2H).

^{13}C NMR (101 MHz, $CDCl_3$) δ 150.26, 130.32, 123.47, 119.10, 114.95, 110.76, 71.33, 70.80, 70.17, 68.43, 52.39, 45.07, 39.05, 32.55, 29.47, 26.71, 25.43.

HRMS (ESI-QTOF) m/z: $[M + H]^+$ Calcd for $C_{17}H_{27}BrClNO_2$: 394.0965; Found 394.0969

Compound **SI-39**

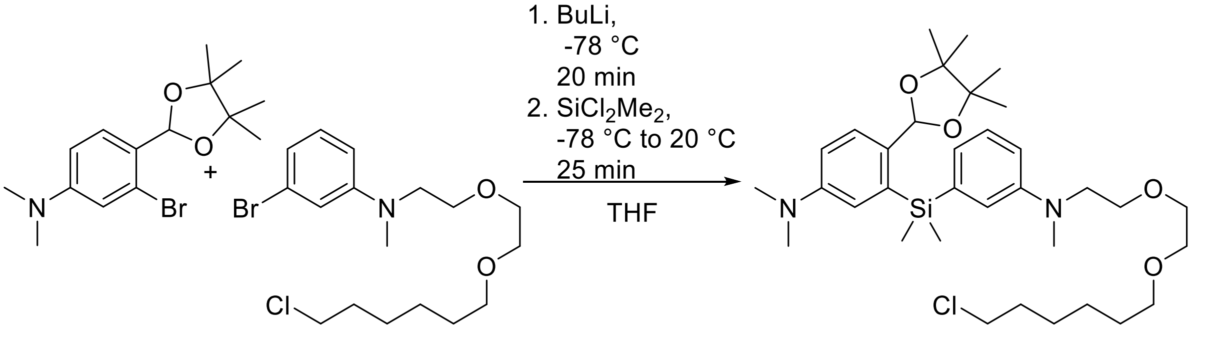

SI-31 **SI-38** **SI-39**

Compound **SI-31** (652 mg, 3 eq) and compound **SI-38** (260 mg, 1 eq) was dissolved in 5 mL of dry THF in a flame dried flask filled with Argon and the solution was cooled to -78 °C. To the cooled solution 2.5 M *n*-Buthyllithium (1.17 mL, 4.4 eq) was added dropwise and the solution was stirred for 20 minutes, while white precipitate formed in the flask. To this suspension Dimethyldichlorosilane (188 mg, 0.176 mL, 2.2 eq) dissolved in 2 mL of dry THF was added dropwise and the cleared out solution was stirred at -78 °C for 25 further minutes, then it was let to warm up to room temperature. After stirring for another 30 minutes, water (20 mL) was added to the reaction mixture and the product was extracted with ethyl acetate (3*20 mL). The combined organic phases were dried over Na_2SO_4 and evaporated. The product was purified with flash column chromatography (hexane:ethyl acetate 0 to 10% in 10 CV) to give 152 mg (37 %) of product.

^{1}H NMR (400 MHz, $CDCl_3$) δ 7.58 (d, *J* = 8.6 Hz, 1H), 7.19 (dd, *J* = 8.3, 7.1 Hz, 1H), 6.90 – 6.82 (m, 3H), 6.77 (dd, *J* = 8.7, 2.8 Hz, 1H), 6.75 – 6.68 (m, 1H), 5.97 (s, 1H), 3.63 – 3.58 (m, 2H), 3.57 – 3.48 (m, 8H), 3.45 (t, *J* = 6.6 Hz, 2H), 2.94 (s, 3H), 2.88 (s, 6H), 1.82 – 1.73 (m, 2H), 1.63 – 1.57 (m, 2H), 1.49 – 1.40 (m, 2H), 1.40 – 1.32 (m, 2H), 1.26 (s, 6H), 1.15 (s, 6H), 0.59 (s, 6H).

^{13}C NMR (101 MHz, $CDCl_3$) δ 150.15, 148.63, 140.04, 137.50, 132.51, 128.61, 127.12, 122.48, 119.74, 117.89, 113.87, 113.08, 99.08, 82.17, 71.41, 70.76, 70.28, 68.68, 52.52, 45.19, 40.69, 39.07, 32.69, 29.61, 26.85, 25.57, 24.88, 22.37, -0.23.

HRMS (ESI-QTOF) m/z: $[M + H]^+$ Calcd for $C_{34}H_{55}ClN_2O_4Si$: 619.3692; Found 619.3704

Compound **SI-40**

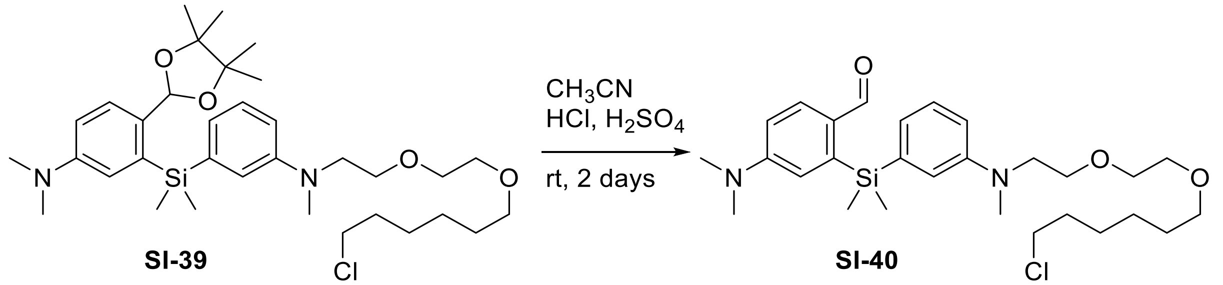

Compound **SI-39** (110 mg) was dissolved in a mixture of 10 mL of acetonitrile and 10 mL of 1M HCl. After 6 hours, according to LC-MS there was barely any deprotection, so 1 mL of ccH_2SO_4 was further added to the reaction mixture. The solution was stirred at room temperature for 2 days, then diluted with ethyl acetate (to 30 mL) and washed with water (3*30 mL), the organic phase dried over Na_2SO_4 and evaporated. The product was purified with preparative HPLC (CH_3CN:H_2O 10-80% in 30 minutes) to give 61 mg (66 %) of product.

^{1}H NMR (400 MHz, $CDCl_3$) δ 9.84 (s, 1H), 7.81 (d, *J* = 8.7 Hz, 1H), 7.20 (dd, *J* = 8.3, 7.1 Hz, 1H), 6.89 (d, *J* = 2.8 Hz, 1H), 6.85 (d, *J* = 7.1 Hz, 1H), 6.81 (d, *J* = 2.7 Hz, 1H), 6.76 – 6.68 (m, 2H), 3.61 (t, *J* = 6.4 Hz, 2H), 3.59 – 3.48 (m, 8H), 3.44 (t, *J* = 6.6 Hz, 2H), 3.00 (s, 6H), 2.95 (s, 3H), 1.82 – 1.71 (m, 2H), 1.64 – 1.53 (m, 2H), 1.50 – 1.40 (m, 2H), 1.40 – 1.30 (m, 2H), 0.62 (s, 6H).

^{13}C NMR (101 MHz, $CDCl_3$) δ 190.94, 152.84, 148.69, 143.30, 139.86, 133.83, 129.83, 128.71, 122.40, 119.76, 117.80, 113.14, 111.34, 71.42, 70.81, 70.27, 68.68, 52.54, 45.20, 39.99, 39.05, 32.69, 29.60, 26.85, 25.57, -0.96.

HRMS (ESI-QTOF) m/z: $[M + H]^+$ Calcd for $C_{28}H_{43}ClN_2O_3Si$: 519.2804; Found 519.2800

Compound **SI-41**

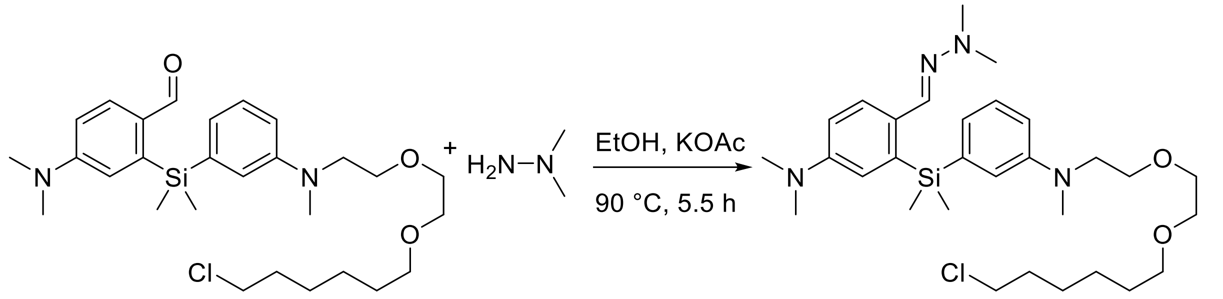

SI-40 **SI-41**

Compound **SI-40** (10 mg, 1 eq), N, N-dimethylhydrazine (23 mg, 29 µL, 20 eq) and potassium acetate (15 mg, 8 eq) was dissolved in 1 mL of ethanol in a sealed screw cap vial and the solution was heated at 90 °C for 5.5 hours. Then the solution was diluted with ethyl acetate (10 mL) and washed with water (3*10 mL), the organic layer dried over Na_2SO_4 and evaporated. The product was further purified with preparative HPLC (CH_3CN:H_2O 10-80% in 30 minutes) to give 2.6 mg (34 %) of product.

^{1}H NMR (400 MHz, $CDCl_3$) δ 7.83 – 7.75 (m, 1H), 7.37 (s, 1H), 7.18 (dd, *J* = 8.3, 7.1 Hz, 1H), 6.89 (d, *J* = 2.8 Hz, 1H), 6.86 (dt, *J* = 7.1, 1.0 Hz, 1H), 6.84 (d, *J* = 2.8 Hz, 1H), 6.76 (dd, *J* = 8.7, 2.8 Hz, 1H), 6.70 (ddd, *J* = 8.3, 2.8, 1.0 Hz, 1H), 3.57 (dd, *J* = 6.6, 5.5 Hz, 2H), 3.55 – 3.50 (m, 6H), 3.50 – 3.42 (m, 4H), 2.95 (s, 6H), 2.92 (s, 3H), 2.63 (s, 6H), 1.83 – 1.73 (m, 2H), 1.65 – 1.52 (m, 2H), 1.49 – 1.40 (m, 2H), 1.41 – 1.31 (m, 2H), 0.56 (s, 6H).

^{13}C NMR (101 MHz, $CDCl_3$) δ 149.32, 148.74, 140.17, 137.20, 136.60, 130.66, 128.79, 125.94, 122.27, 119.23, 117.70, 114.12, 113.12, 71.43, 70.78, 70.28, 68.62, 52.47, 45.20, 43.32, 40.63, 39.07, 32.70, 29.62, 26.86, 25.58, -0.73.

HRMS (ESI-QTOF) m/z: $[M + H]^+$ Calcd for $C_{30}H_{49}ClN_4O_2Si$: 561.3386; Found 561.3387

Compound **SI-42**

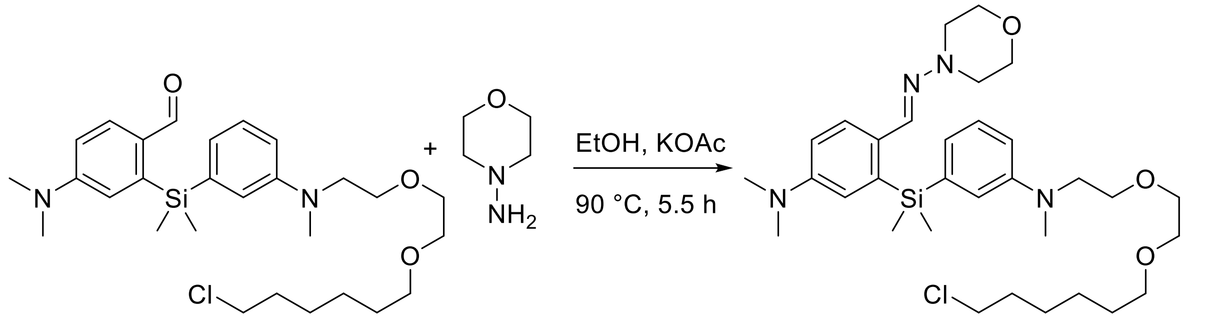

SI-40 **SI-42**

Compound **SI-40** (10 mg, 1 eq), 4-Aminomorpholine (39 mg, 37 µL, 20 eq) and potassium acetate (15 mg, 8 eq) was dissolved in 1 mL of ethanol in a sealed screw cap vial and the solution was heated at 90 °C for 5.5 hours. Then the solution was diluted with ethyl acetate (10 mL) and washed with water (3*10 mL), the organic layer dried over Na_2SO_4 and evaporated. The product was further purified with preparative HPLC (CH_3CN:H_2O 10-80% in 30 minutes) to give 5.7 mg (70 %) of product.

^{1}H NMR (400 MHz, $CDCl_3$) δ 7.82 (d, *J* = 8.7 Hz, 1H), 7.64 (s, 1H), 7.18 (dd, *J* = 8.3, 7.0 Hz, 1H), 6.90 (d, *J* = 2.8 Hz, 1H), 6.87 – 6.80 (m, 2H), 6.76 (dd, *J* = 8.8, 2.8 Hz, 1H), 6.70 (ddd, *J* = 8.4, 2.8, 1.0 Hz, 1H), 3.77 – 3.68 (m, 4H), 3.58 (t, *J* = 6.1 Hz, 2H), 3.56 – 3.50 (m, 6H), 3.50 – 3.42 (m, 4H), 2.97 (s, 6H), 2.92 (s, 3H), 2.79 – 2.69 (m, 4H), 1.82 – 1.72 (m, 2H), 1.62 – 1.56 (m, 2H), 1.48 – 1.40 (m, 2H), 1.40 – 1.33 (m, 2H), 0.56 (s, 6H).

^{13}C NMR (101 MHz, $CDCl_3$) δ 149.79, 148.84, 140.15, 139.99, 137.65, 129.47, 128.92, 126.38, 122.13, 118.87, 117.53, 113.88, 113.10, 71.43, 70.80, 70.28, 68.66, 66.64, 52.45, 52.37, 45.20, 40.50, 39.02, 32.70, 29.61, 26.85, 25.58, -0.73.

HRMS (ESI-QTOF) m/z: $[M + H]^+$ Calcd for $C_{32}H_{51}ClN_4O_3Si$: 603.3492; Found 603.3497

Compound **SI-43**

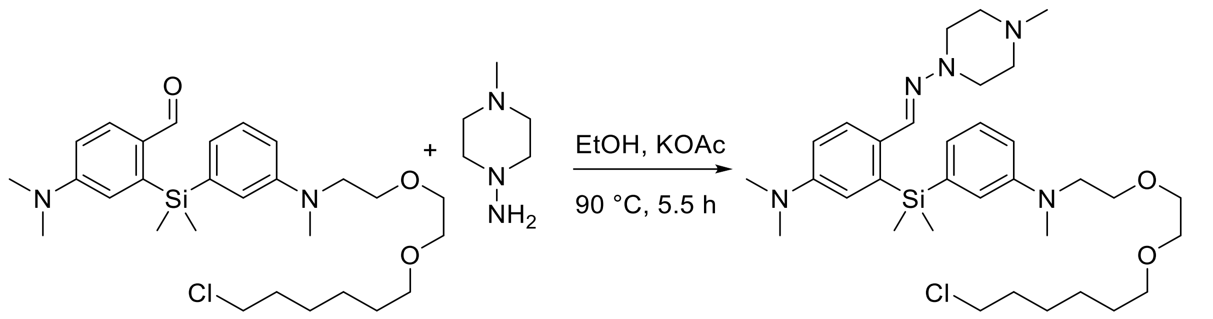

SI-40 **SI-43**

Compound **SI-40** (10 mg, 1 eq), 1-Amino-4-methylpiperazin (44 mg, 46 µL, 20 eq) and potassium acetate (15 mg, 8 eq) was dissolved in 1 mL of ethanol in a sealed screw cap vial and the solution was heated at 90 °C for 5.5 hours. Then the solution was diluted with ethyl acetate (10 mL) and washed with water (3*10 mL), the organic layer dried over Na_2SO_4 and evaporated. The product was further purified with preparative HPLC (CH_3CN:H_2O 10-80% in 30 minutes) to give 6.5 mg (55 %) of product.

^1H NMR (400 MHz, $CDCl_3$) δ 7.83 (d, *J* = 8.7 Hz, 1H), 7.61 (s, 1H), 7.17 (dd, *J* = 8.3, 7.1 Hz, 1H), 6.89 (d, *J* = 2.8 Hz, 1H), 6.87 – 6.81 (m, 2H), 6.75 (dd, *J* = 8.8, 2.8 Hz, 1H), 6.72 – 6.66 (m, 1H), 3.61 – 3.50 (m, 8H), 3.50 – 3.41 (m, 4H), 2.96 (s, 6H), 2.92 (s, 3H), 2.85 (t, *J* = 5.0 Hz, 4H), 2.48 (t, *J* = 5.1 Hz, 4H), 1.83 – 1.72 (m, 2H), 1.59 (p, *J* = 6.8 Hz, 2H), 1.49 – 1.41 (m, 2H), 1.41 – 1.34 (m, 2H), 0.55 (s, 6H).

^{13}C NMR (101 MHz, $CDCl_3$) δ 149.69, 148.80, 140.12, 139.64, 137.45, 129.84, 128.89, 126.28, 122.16, 118.95, 117.50, 113.94, 113.11, 71.43, 70.78, 70.28, 68.64, 54.57, 52.45, 51.37, 45.97, 45.20, 40.53, 39.03, 32.70, 29.61, 26.85, 25.58, -0.74.

HRMS (ESI-QTOF) m/z: $[M + H]^+$ Calcd for $C_{33}H_{54}ClN_5O_2Si$: 616.3808; Found 616.3808

Compound **SI-44**

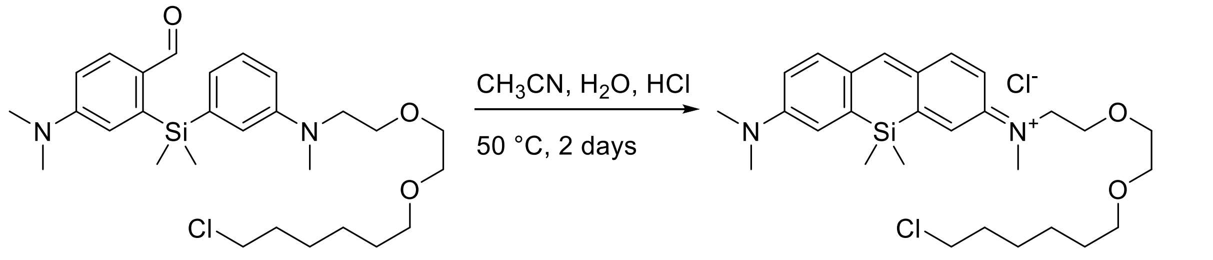

SI-40 **SI-44**

Compound **SI-40** (4 mg) was dissolved in a mixture of 4 mL of acetonitrile and 4 mL of 2M HCl and stirred in a closed flask at 50 °C for 2 days, then full conversion was confirmed by LC-MS. The acid was neutralised with 8 mL of 1M NaOH, then most of the solvents were evaporated and the crude product was purified with preparative HPLC (CH_3CN:H_2O 10-80% in 30 minutes) to give 1.0 mg (24 %) of product.

^1H NMR (400 MHz, $CDCl_3$) δ 7.86 (s, 1H), 7.73 (dd, *J* = 9.2, 3.4 Hz, 2H), 7.29 (s, 1H), 7.17 (d, *J* = 2.7 Hz, 1H), 6.94 (dd, *J* = 9.2, 2.7 Hz, 1H), 6.88 (dd, *J* = 9.2, 2.7 Hz, 1H), 3.98 (t, *J* = 5.2 Hz, 2H), 3.83 (t, *J* = 5.2 Hz, 2H), 3.63 – 3.51 (m, 8H), 3.42 – 3.40 (m, 9H), 1.77 – 1.72 (m, 2H), 1.46 – 1.41 (m, 2H), 1.35 – 1.31 (m, 2H), 0.56 (s, 6H).

^{13}C NMR (101 MHz, $CDCl_3$) δ 160.36, 155.25, 155.19, 148.03, 143.92, 143.82, 128.05, 127.92, 121.60, 121.01, 116.49, 114.67, 114.32, 71.40, 71.05, 70.23, 69.00, 45.23, 41.37, 40.61, 32.65, 31.07, 29.60, 26.81, 25.56, -0.89.

HRMS (ESI-QTOF) m/z: $[M]^+$ Calcd for $C_{28}H_{42}ClN_2O_2Si^+$: 501.2699; Found 501.2717

Literature references

[74] K. Uno, M. L. Bossi, T. Konen, V. N. Belov, M. Irie, S. W. Hell, Asymmetric Diarylethenes with Oxidized 2-Alkylbenzothiophen-3-yl Units: Chemistry, Fluorescence, and Photoswitching, *Adv Opt Mater*, **2019**, *7*, 1801746.

[75] R. Lincoln, M. L. Bossi, M. Remmel, E. D'Este, A. N. Butkevich, S. W. Hell, A general design of caging-group-free photoactivatable fluorophores for live-cell nanoscopy, *Nat Chem*, **2022**, *14*, 1013-1020.

[76] M. S. Frei, M. Tarnawski, M. J. Roberti, B. Koch, J. Hiblot, K. Johnsson, Engineered HaloTag variants for fluorescence lifetime multiplexing, *Nat Methods*, **2022**, *19*, 65-70.

[77] J. M. Schleske, J. Hubrich, J. O. Wirth, E. D'Este, J. Engelhardt, S. W. Hell, MINFLUX reveals dynein stepping in live neurons, *P Natl Acad Sci USA*, **2024**, *121*, e2412241121.

[78] R. Schmidt, T. Weihs, C. A. Wurm, I. Jansen, J. Rehman, S. J. Sahl, S. W. Hell, MINFLUX nanometer-scale 3D imaging and microsecond-range tracking on a common fluorescence microscope, *Nat Commun*, **2021**, *12*, 1478.

[79] M. Remmel, J. Matthias, R. Lincoln, J. Keller-Findeisen, A. N. Butkevich, M. L. Bossi, S. W. Hell, Photoactivatable Xanthone (PaX) Dyes Enable Quantitative, Dual Color, and Live-Cell MINFLUX Nanoscopy, *Small Methods*, **2024**, *8*, e2301497.

[80] A. N. Butkeyich, M. L. Bossi, G. Lukinavicius, S. W. Hell, Triarylmethane Fluorophores Resistant to Oxidative Photobluing, *J Am Chem Soc*, **2019**, *141*, 981-989.

[81] M. S. Frei, P. Hoess, M. Lampe, B. Nijmeijer, M. Kueblbeck, J. Ellenberg, H. Wadepohl, J. Ries, S. Pitsch, L. Reymond, K. Johnsson, Photoactivation of silicon rhodamines via a light-induced protonation, *Nat Commun*, **2019**, *10*, 4580

[82] V. N. Belov, G. Y. Mitronova, M. L. Bossi, V. P. Boyarskiy, E. Hebisch, C. Geisler, K. Kolmakov, C. A. Wurm, K. I. Willig, S. W. Hell, Masked Rhodamine Dyes of Five Principal Colors Revealed by Photolysis of a 2-Diazo-1-Indanone Caging Group: Synthesis, Photophysics, and Light Microscopy Applications, *Chem-Eur J*, **2014**, *20*, 13162-13173.

[83] J. B. Grimm, B. P. English, H. Choi, A. K. Muthusamy, B. P. Mehl, P. Dong, T. A. Brown, J. Lippincott-Schwartz, Z. Liu, T. Lionnet, L. D. Lavis, Bright photoactivatable fluorophores for single-molecule imaging, *Nat Methods*, **2016**, *13*, 985-988.

[84] A. Eördögh, C. Paganini, D. Pinotsi, P. Arosio, P. Rivera-Fuentes, A Molecular Logic Gate Enables Single-Molecule Imaging and Tracking of Lipids in Intracellular Domains, *Acs Chem Biol*, **2020**, *15*, 2597-2604.

[85] J. Tang, M. A. Robichaux, K. L. Wu, J. Q. Pei, N. T. Nguyen, Y. B. Zhou, T. G. Wensel, H. Xiao, Single-Atom Fluorescence Switch: A General Approach toward Visible-Light-Activated Dyes for Biological Imaging, *J Am Chem Soc*, **2019**, *141*, 14699-14706.

[86] S. J. Lord, H. L. D. Lee, R. Samuel, R. Weber, N. Liu, N. R. Conley, M. A. Thompson, R. J. Twieg, W. E. Moerner, Azido Push-Pull Fluorogens Photoactivate to Produce Bright Fluorescent Labels, *J Phys Chem B*, **2010**, *114*, 14157-14167.

[87] A. Aktalay, T. A. Khan, M. L. Bossi, V. N. Belov, S. W. Hell, Photoactivatable Carbo- and Silicon-Rhodamines and Their Application in MINFLUX Nanoscopy, *Angew Chem Int Edit*, **2023**, *62*, e202302781.

[88] H. Okamura, M. Iida, Y. Kaneyama, F. Nagatsugi, o-Nitrobenzyl Oxime Ethers Enable Photoinduced Cyclization Reaction to Provide Phenanthridines under Aqueous Conditions, *Org Lett*, **2023**, *25*, 466-470.

[89] J. D. Smith, E. Chih, W. E. Piers, D. M. Spasyuk, Tuning iridium (I) PCcarbeneP frameworks for facile cooperative N2O reduction, *Polyhedron*, **2018**, *155*, 281-290.

[90] F. Grimm, S. Nizamov, V. N. Belov, Green-Emitting Rhodamine Dyes for Vital Labeling of Cell Organelles Using STED Super-Resolution Microscopy, *Chembiochem*, **2019**, *20*, 2248-2254.

[91] Y. Koide, Y. Urano, K. Hanaoka, W. Piao, M. Kusakabe, N. Saito, T. Terai, T. Okabe, T. Nagano, Development of NIR fluorescent dyes based on Si-rhodamine for in vivo imaging, *J Am Chem Soc*, **2012**, *134*, 5029-5031.

[92] A. Shafir, P. A. Lichtor, S. L. Buchwald, N- versus O-arylation of aminoalcohols: Orthogonal selectivity in copper-based catalysts, *J Am Chem Soc*, **2007**, *129*, 3490-3491.

Compound 1

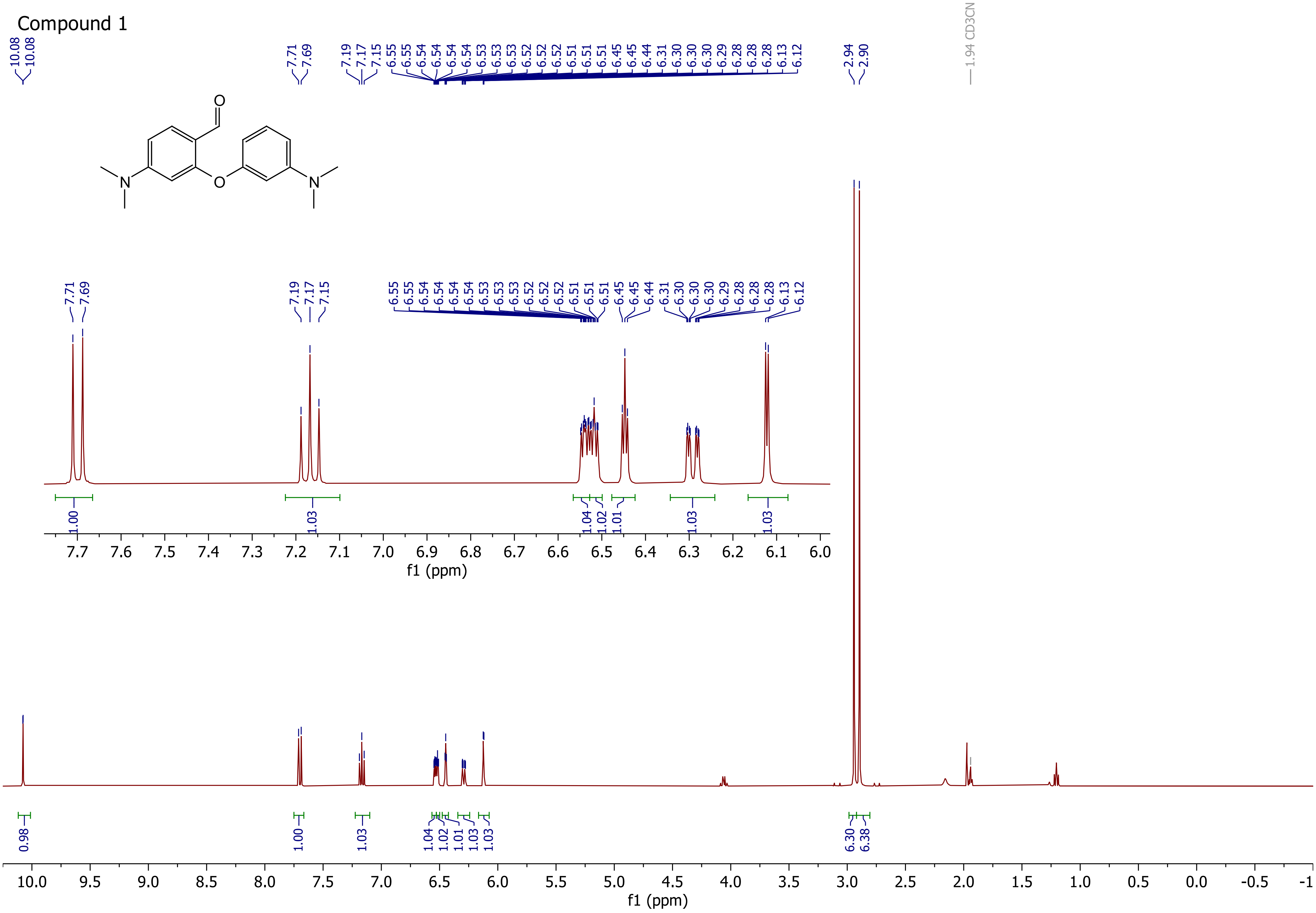

10.08
10.08
7.71
7.69
7.19
7.17
7.15
6.55
6.55
6.54
6.54
6.54
6.54
6.53
6.53
6.53
6.52
6.52
6.52
6.51
6.51
6.51
6.45
6.45
6.44
6.31
6.30
6.30
6.30
6.29
6.28
6.28
6.13
6.12
2.94
2.90
1.94 CD3CN
O
N
O
N
1.00
1.03
1.04
1.02
1.01
1.03
1.03
f1 (ppm)
0.98
6.30
6.38
f1 (ppm)

Compound 1

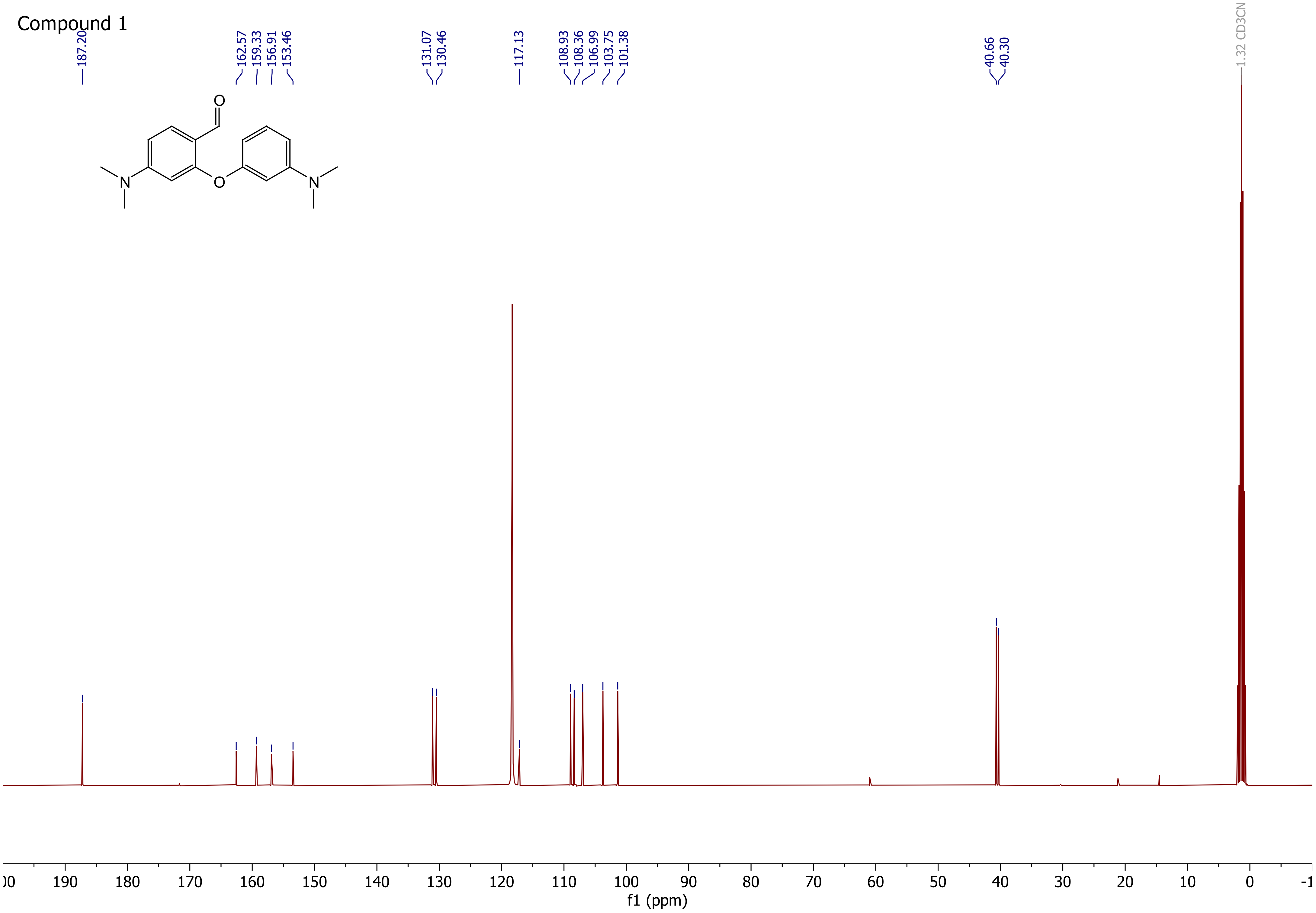

187.20
162.57
159.33
156.91
153.46
131.07
130.46
117.13
108.93
108.36
106.99
103.75
101.38
40.66
40.30
1.32 CD3CN
f1 (ppm)

Compound 2

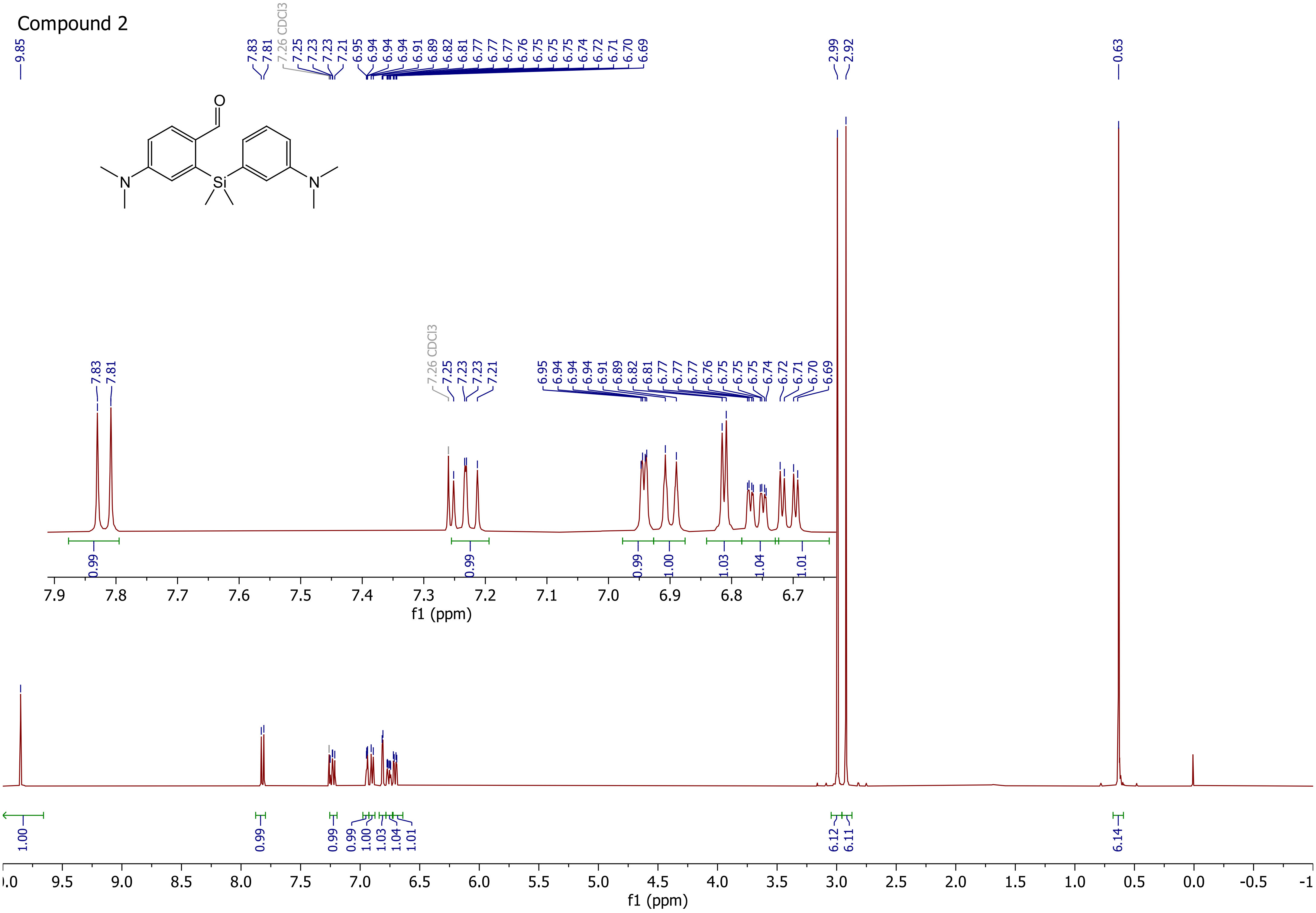

9.85
7.83
7.81
7.26 CDCl3
7.25
7.23
7.23
7.21
6.95
6.94
6.94
6.94
6.91
6.89
6.82
6.81
6.77
6.77
6.77
6.76
6.75
6.75
6.75
6.74
6.72
6.71
6.70
6.69
2.99
2.92
0.63
O
N
Si
N
0.99
0.99
0.99
1.00
1.03
1.04
1.01
f1 (ppm)
1.00
6.12
6.11
6.14
f1 (ppm)

Compound 2

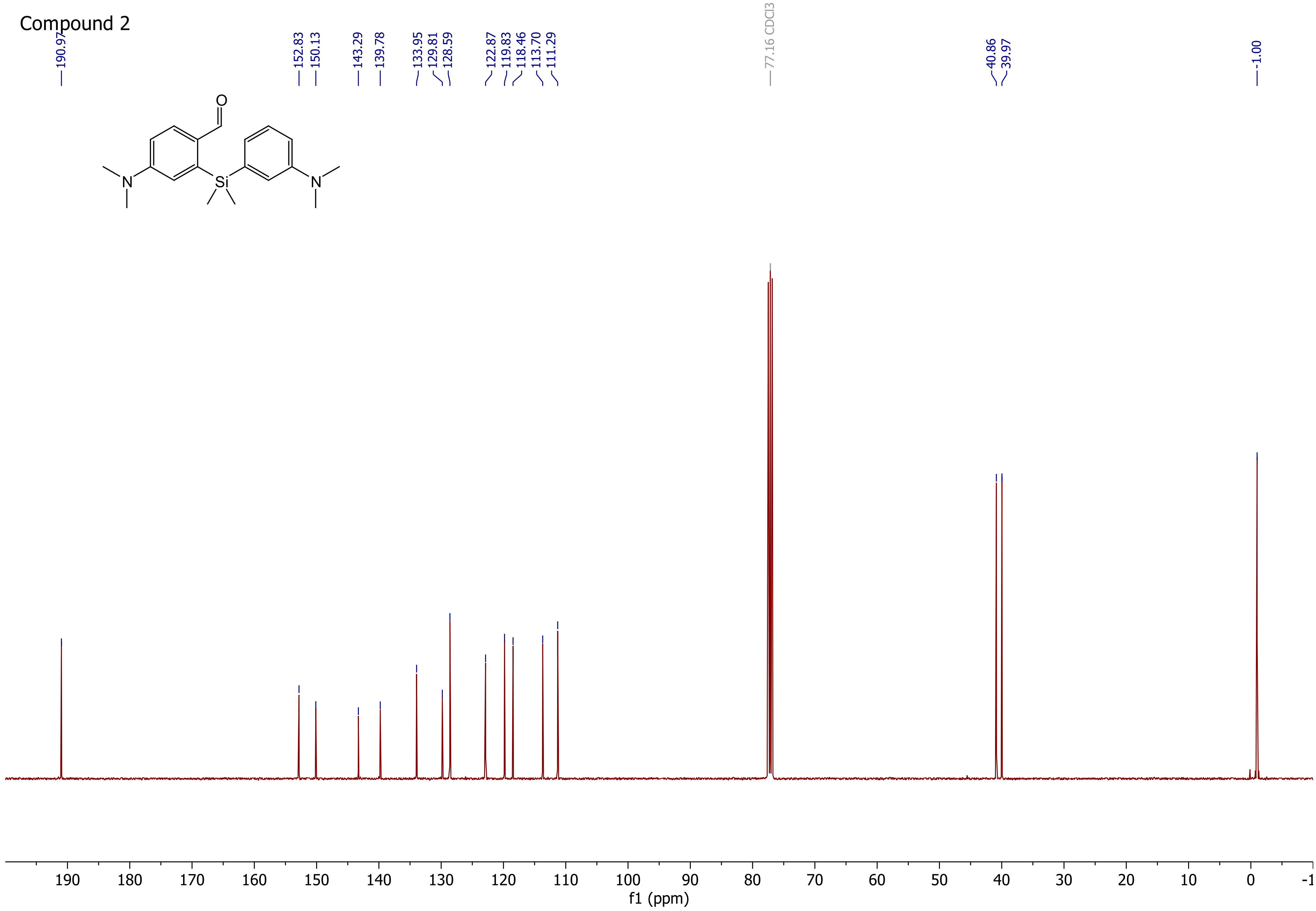

190.97
152.83
150.13
143.29
139.78
133.95
129.81
128.59
122.87
119.83
118.46
113.70
111.29
77.16 CDCl3
40.86
39.97
-1.00
O
N
Si
N
190
180
170
160
150
140
130
120
110
100
90
80
70
60
50
40
30
20
10
0
-1
f1 (ppm)

Compound 2
HSQC

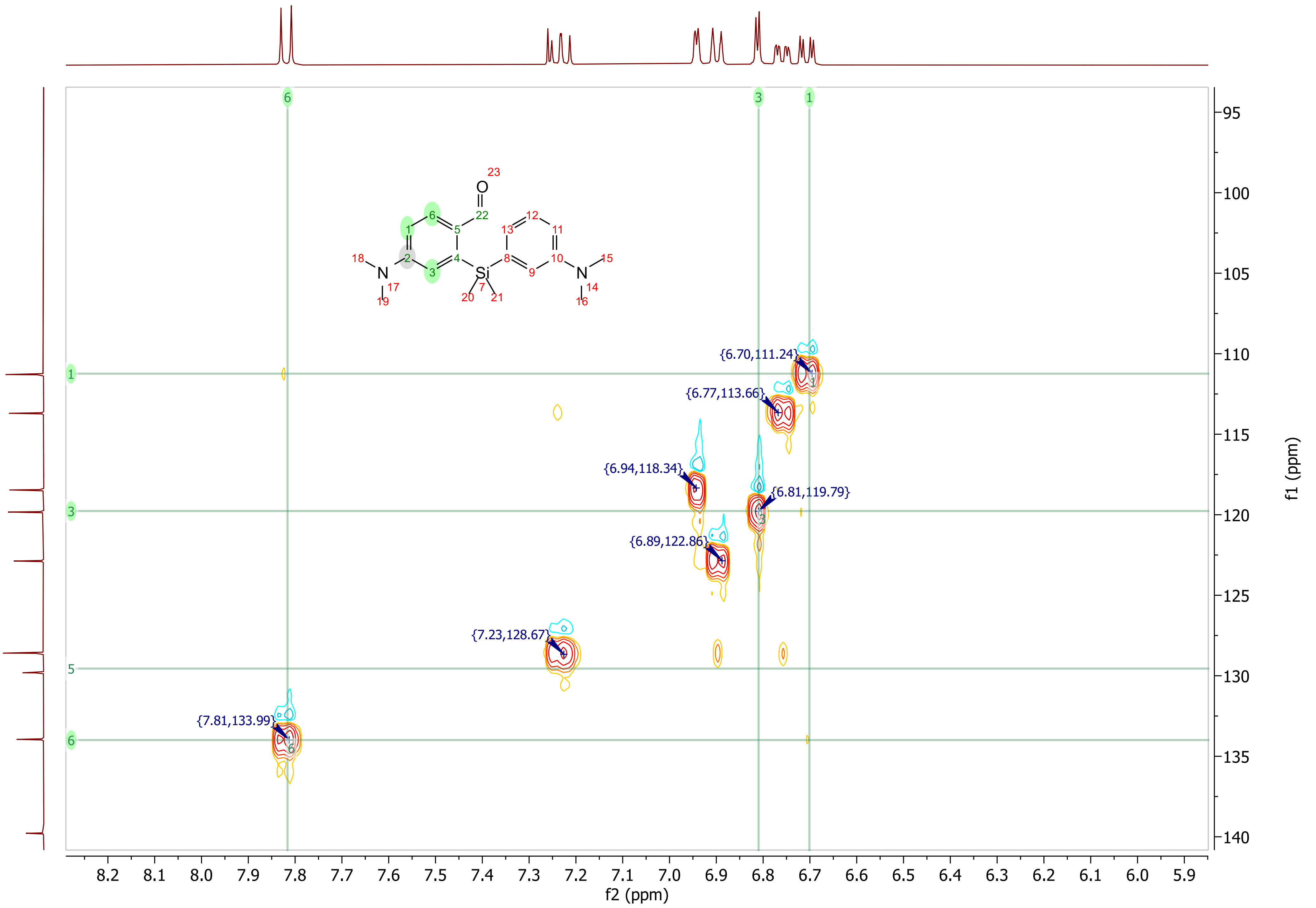

Compound 2
HMBC

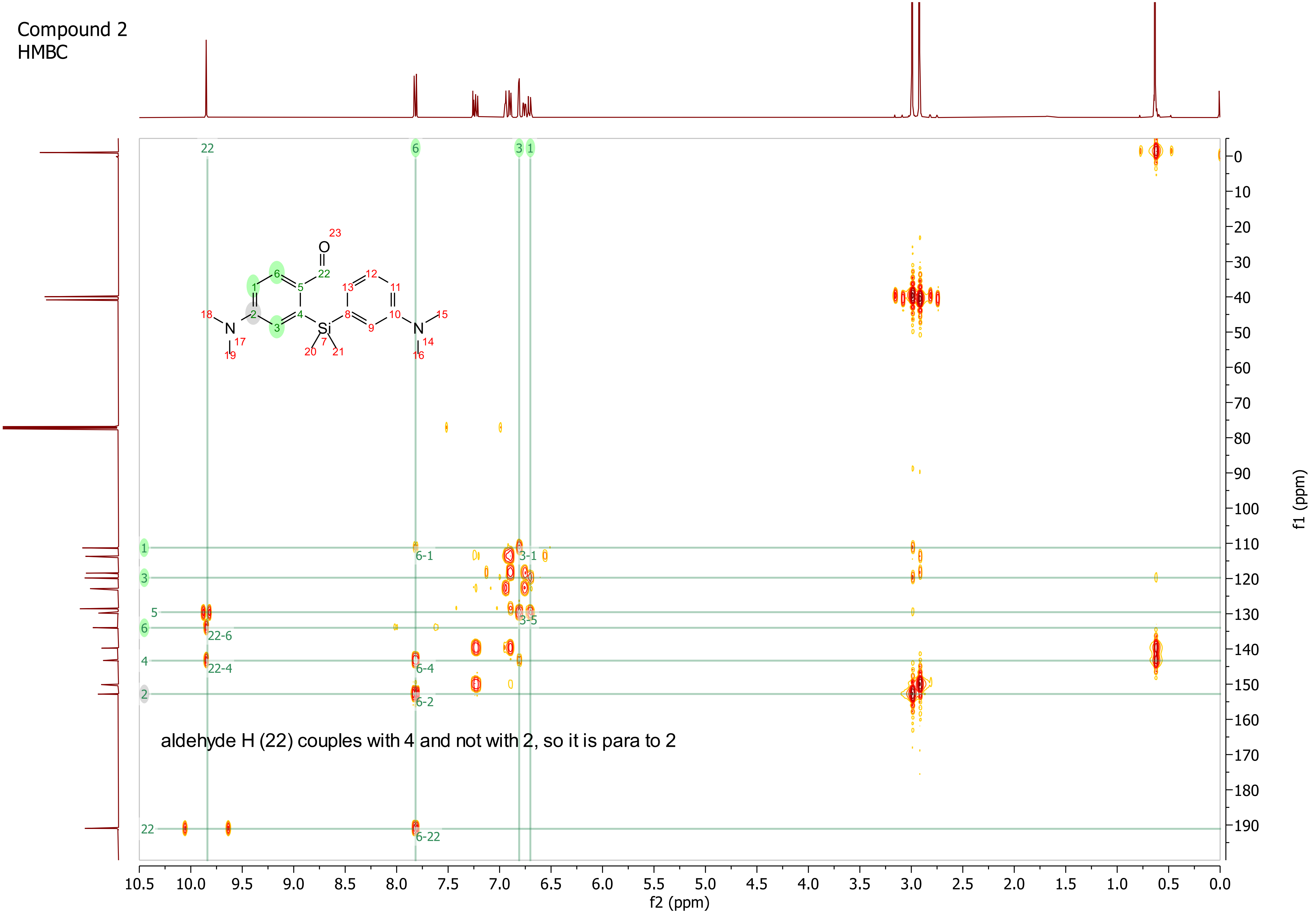

Compound 3

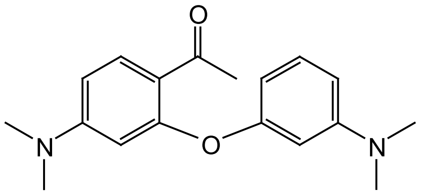

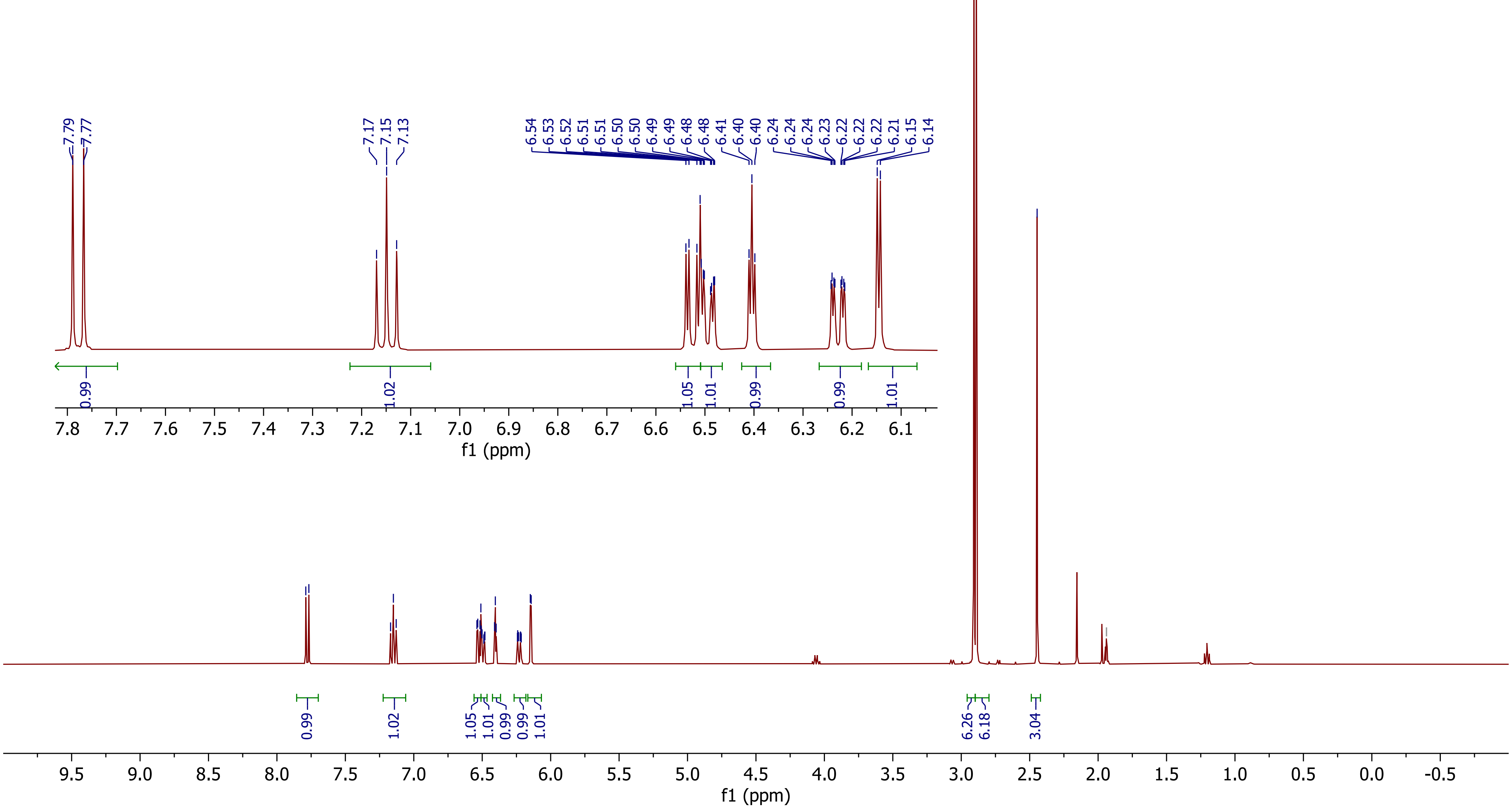
7.79
7.77
7.17
7.15
7.13
6.54
6.53
6.52
6.51
6.51
6.50
6.50
6.49
6.49
6.48
6.48
6.41
6.40
6.40
6.24
6.24
6.24
6.23
6.22
6.22
6.22
6.21
6.15
6.14
2.91
2.89
2.45
1.94 CD3CN
0.99
1.02
1.05
1.01
0.99
0.99
1.01
6.26
6.18
3.04
f1 (ppm)

Compound 3

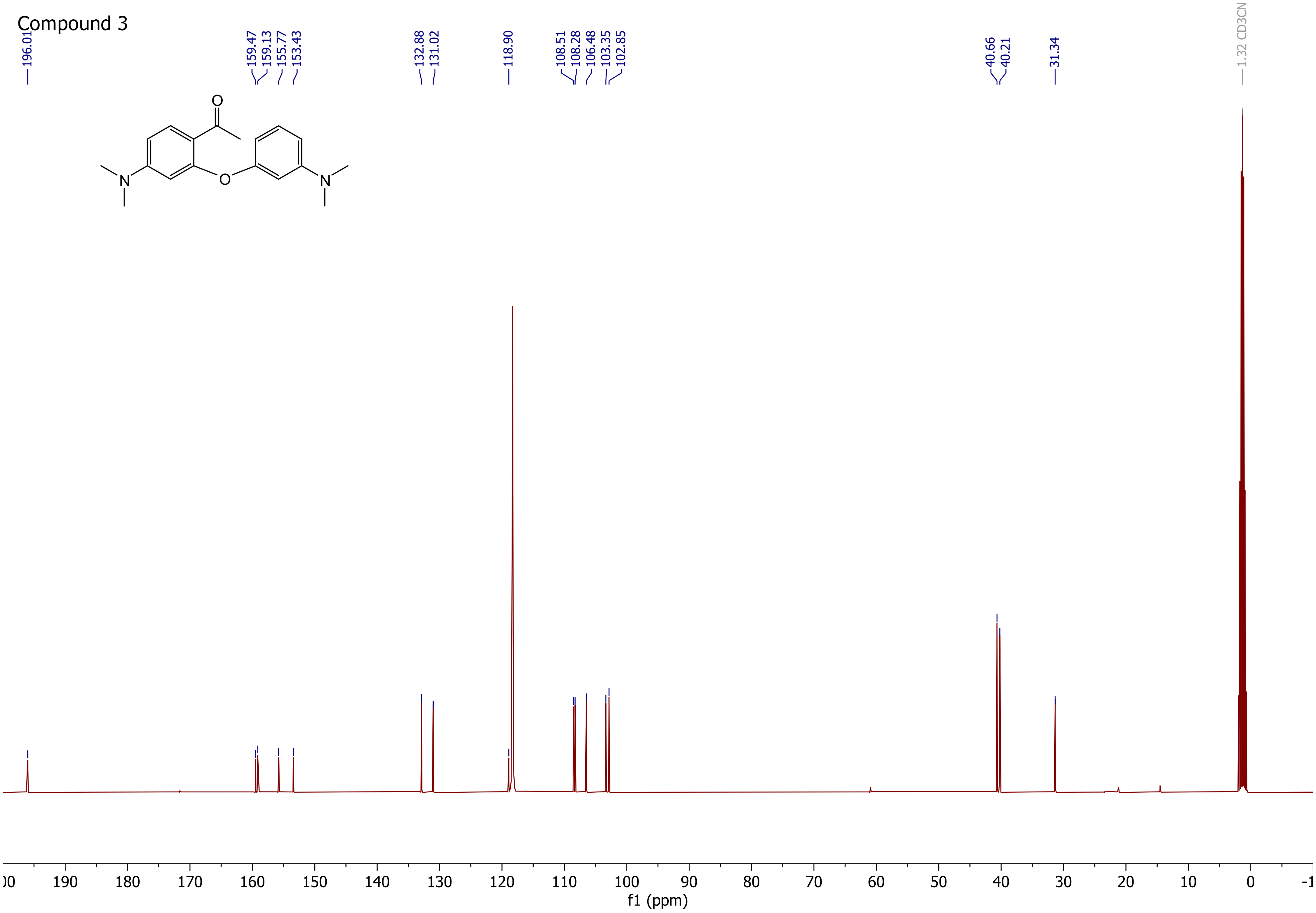
196.01
159.47
159.13
155.77
153.43
132.88
131.02
118.90
108.51
108.28
106.48
103.35
102.85
40.66
40.21
31.34
1.32 CD3CN
O
N
O
N
190
180
170
160
150
140
130
120
110
100
90
80
70
60
50
40
30
20
10
0
f1 (ppm)

Compound 4

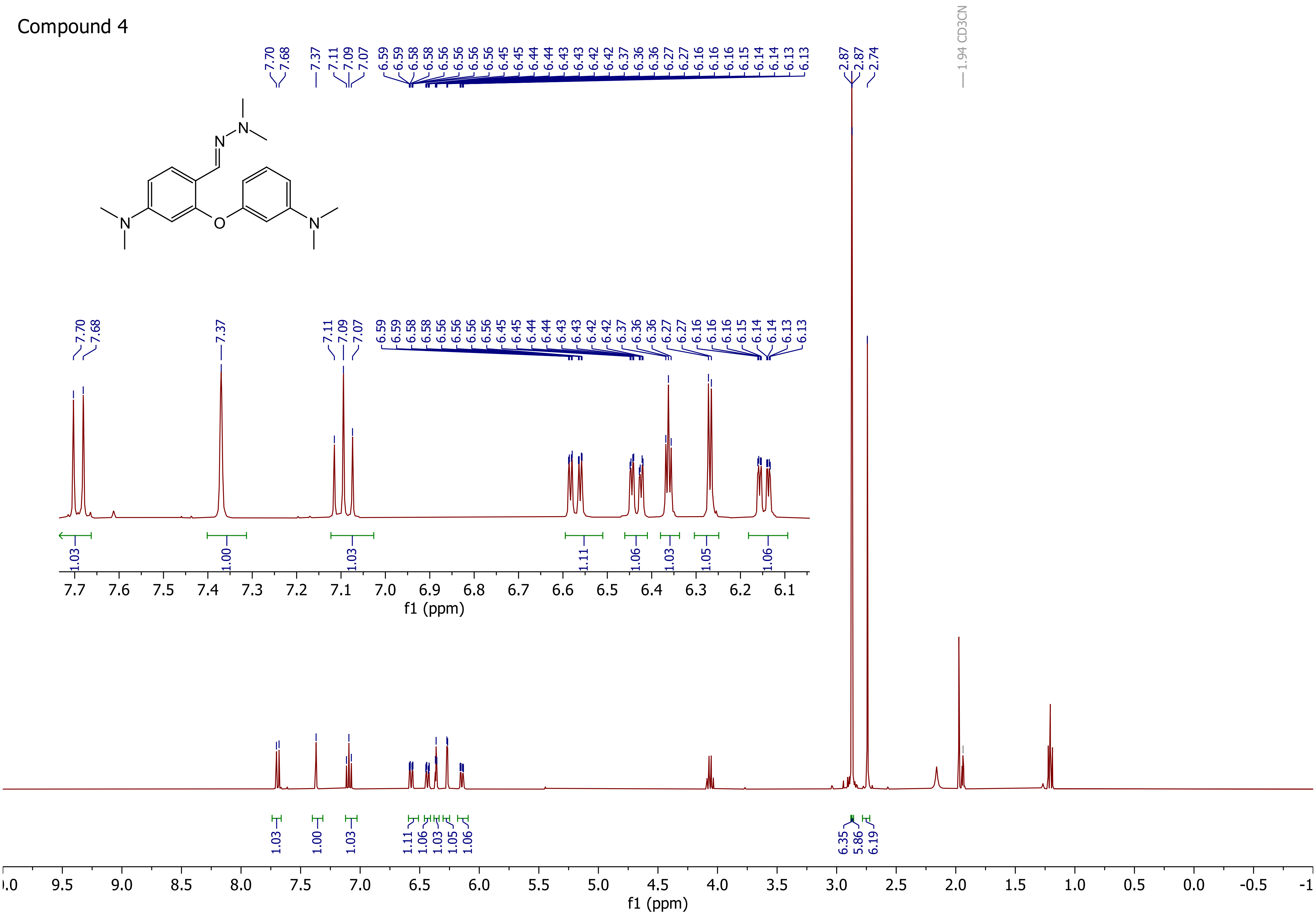

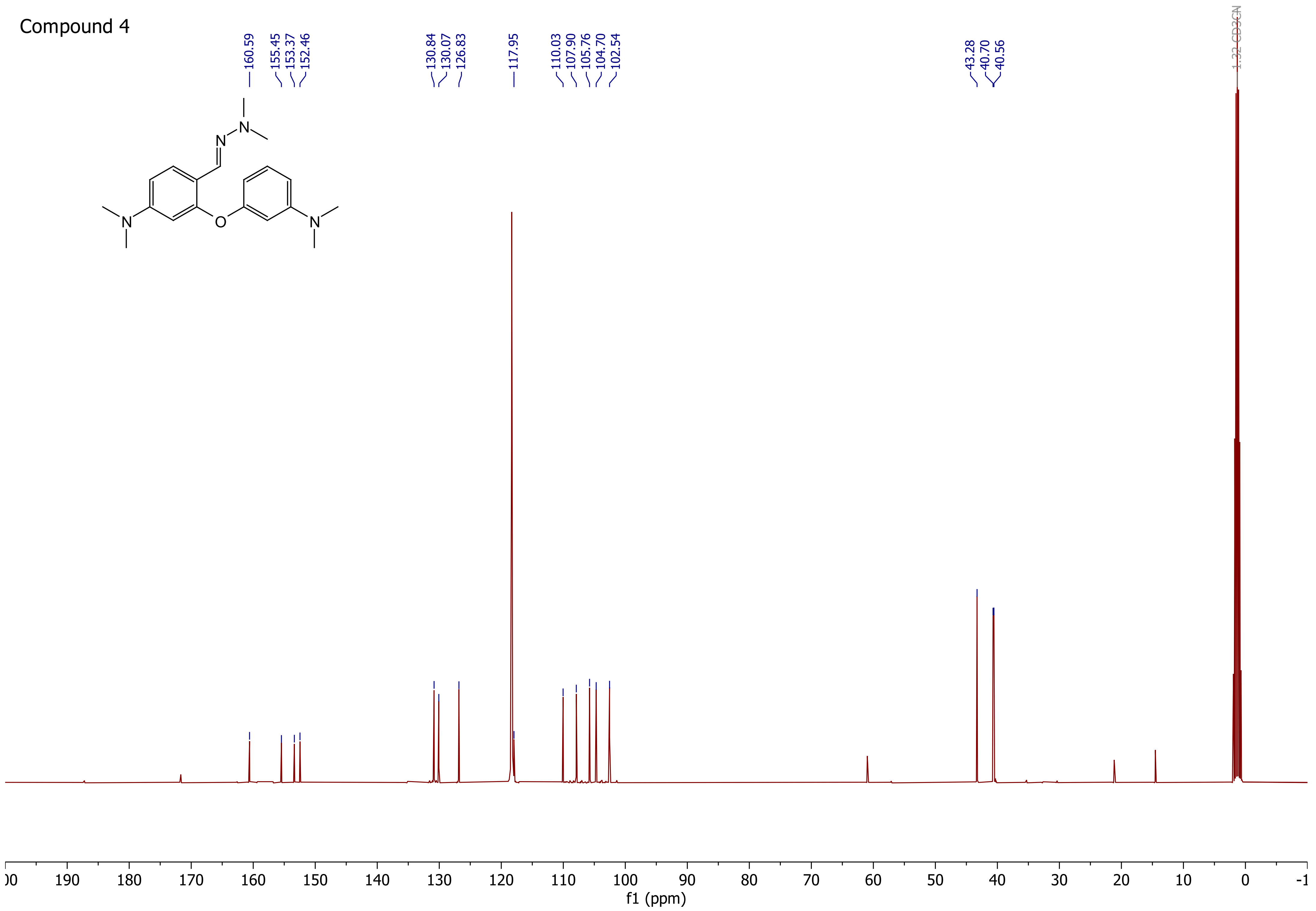

Compound 4
160.59
155.45
153.37
152.46
130.84
130.07
126.83
117.95
110.03
107.90
105.76
104.70
102.54
43.28
40.70
40.56
1.32 CD3CN
N
N
O
N
N
190
180
170
160
150
140
130
120
110
100
90
80
70
60
50
40
30
20
10
0
f1 (ppm)

Compound 5

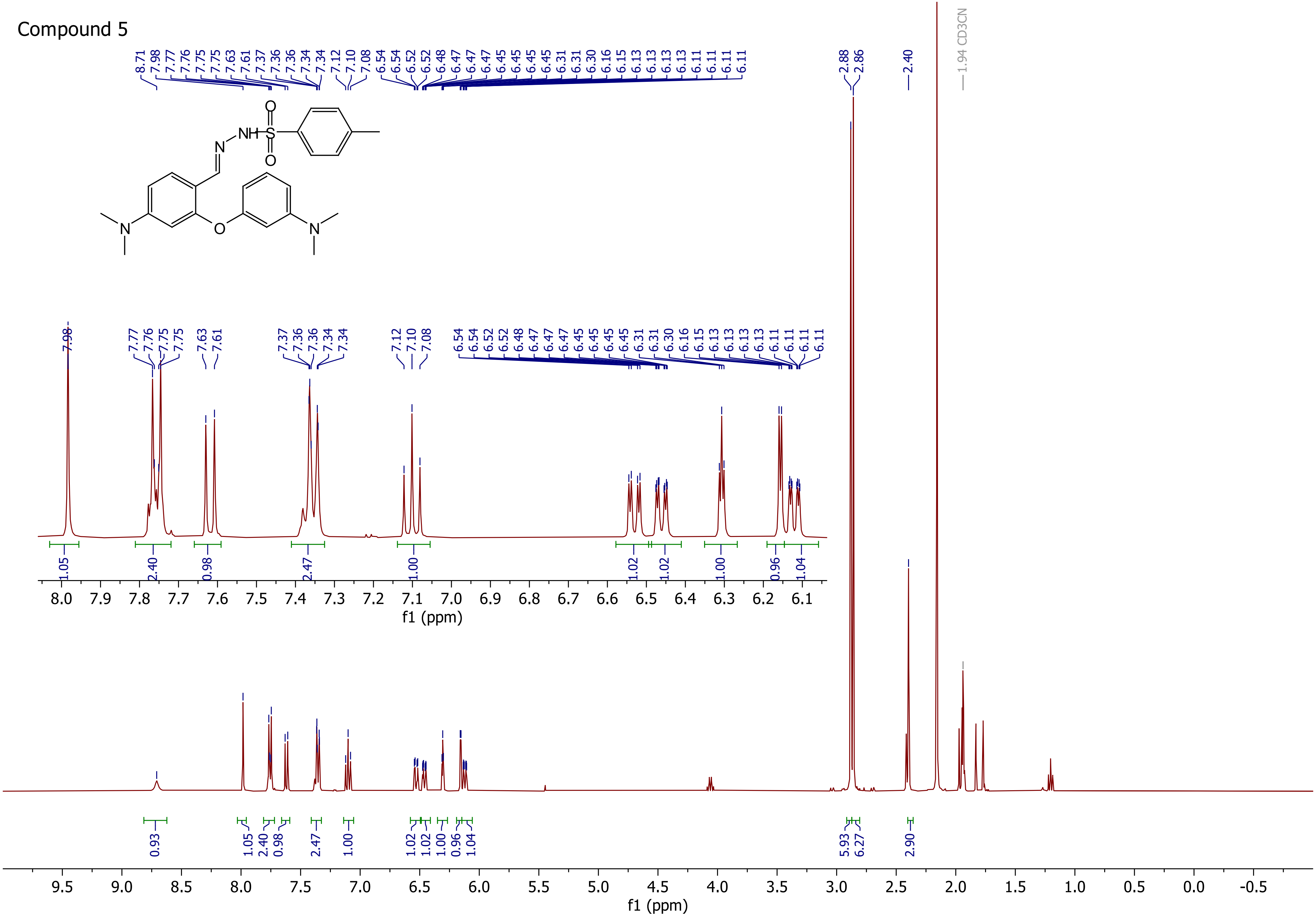

8.71 7.98 7.77 7.76 7.75 7.75 7.63 7.61 7.37 7.36 7.36 7.34 7.34 7.12 7.10 7.08 6.54 6.54 6.52 6.52 6.48 6.47 6.47 6.47 6.45 6.45 6.45 6.45 6.31 6.31 6.30 6.16 6.15 6.13 6.13 6.13 6.13 6.11 6.11 6.11 6.11
2.88 2.86
2.40
1.94 CD3CN
NH
S
O
O
N
O
N
N
1.05 2.40 0.98 2.47 1.00 1.02 1.02 1.00 0.96 1.04
8.0 7.9 7.8 7.7 7.6 7.5 7.4 7.3 7.2 7.1 7.0 6.9 6.8 6.7 6.6 6.5 6.4 6.3 6.2 6.1
f1 (ppm)
0.93 1.05 2.40 0.98 2.47 1.00 1.02 1.02 1.00 0.96 1.04 5.93 6.27 2.90
9.5 9.0 8.5 8.0 7.5 7.0 6.5 6.0 5.5 5.0 4.5 4.0 3.5 3.0 2.5 2.0 1.5 1.0 0.5 0.0 -0.5
f1 (ppm)

Compound 5

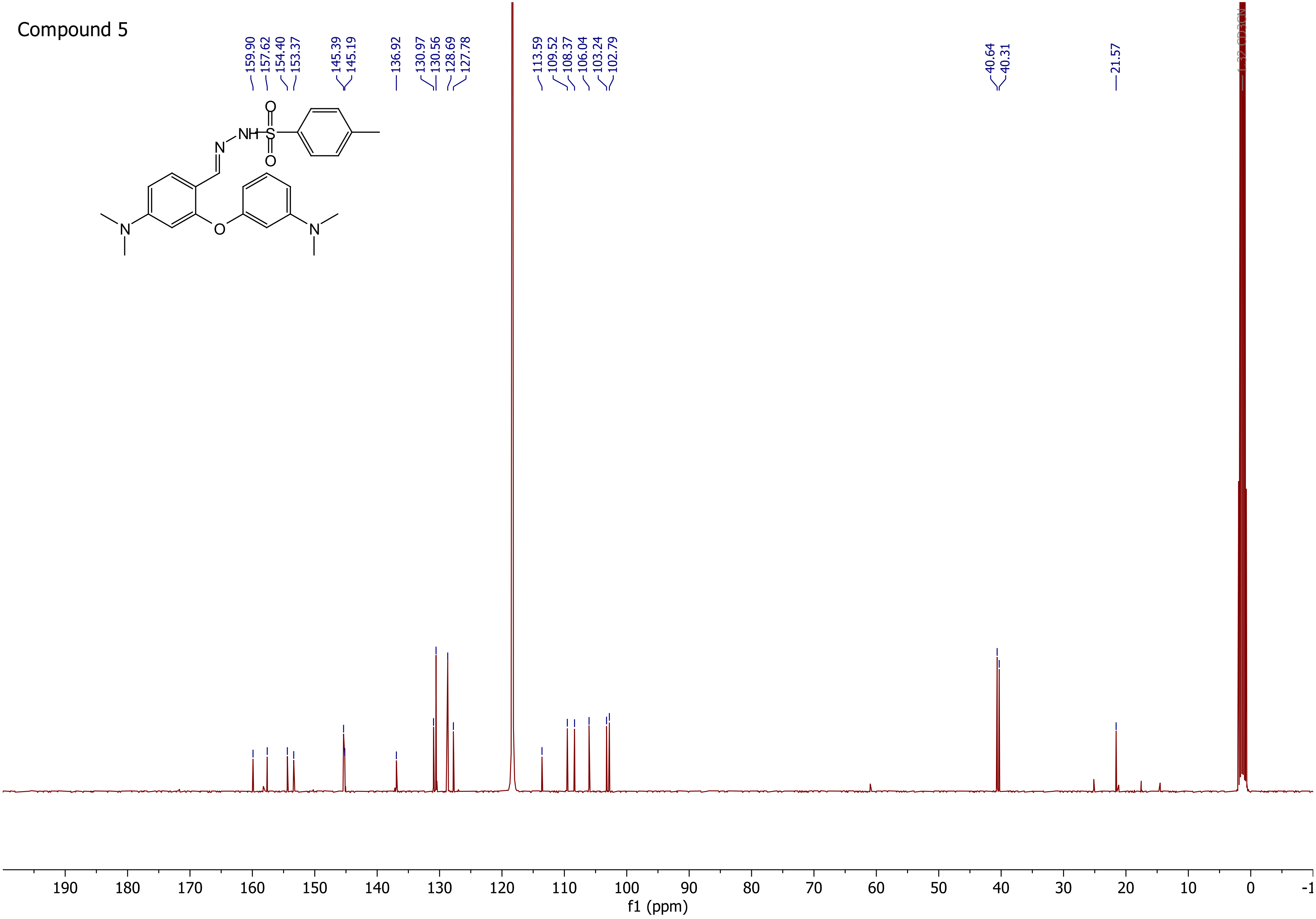

159.90
157.62
154.40
153.37
145.39
145.19
136.92
130.97
130.56
128.69
127.78
113.59
109.52
108.37
106.04
103.24
102.79
40.64
40.31
21.57
1.32 CD3CN
O
NH
S
N
O
N
O
N
190 180 170 160 150 140 130 120 110 100 90 80 70 60 50 40 30 20 10 0 -1
f1 (ppm)

Compound 6

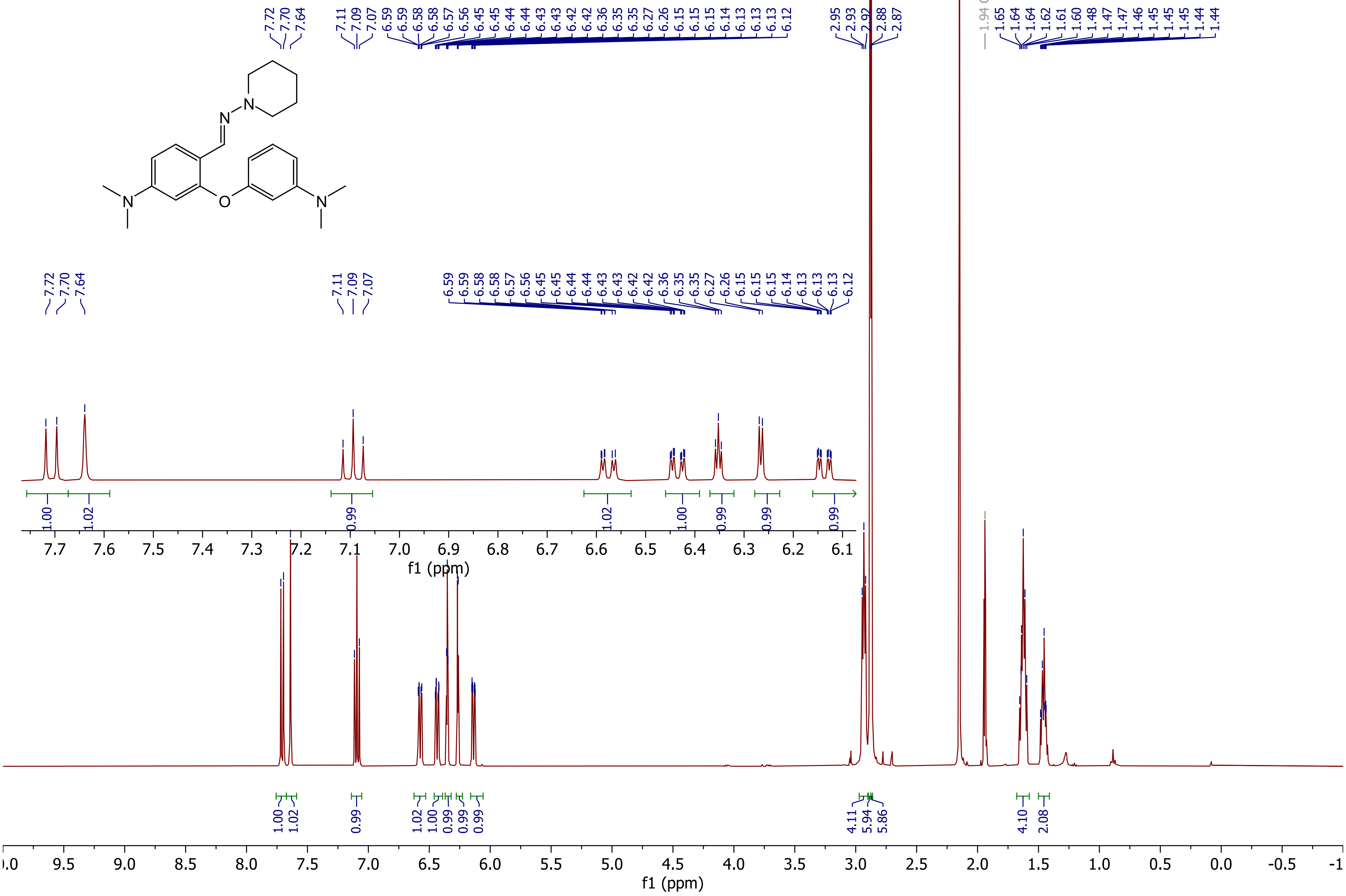

7.72
7.70
7.64
7.11
7.09
7.07
6.59
6.59
6.58
6.58
6.57
6.56
6.45
6.45
6.44
6.44
6.43
6.43
6.42
6.42
6.36
6.35
6.35
6.27
6.26
6.15
6.15
6.15
6.14
6.13
6.13
6.13
6.12
2.95
2.93
2.92
2.88
2.87
1.94 CD3CN
1.65
1.64
1.64
1.62
1.61
1.60
1.48
1.47
1.47
1.46
1.45
1.45
1.45
1.44
1.44
N
N
O
N
N
1.00
1.02
0.99
1.02
1.00
0.99
0.99
0.99
4.11
5.94
5.86
4.10
2.08
f1 (ppm)
f1 (ppm)

Compound 6

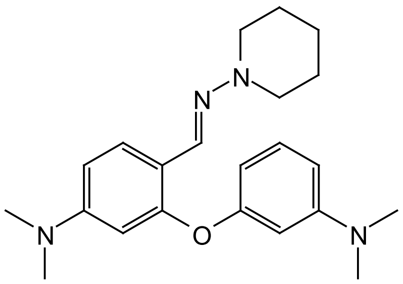

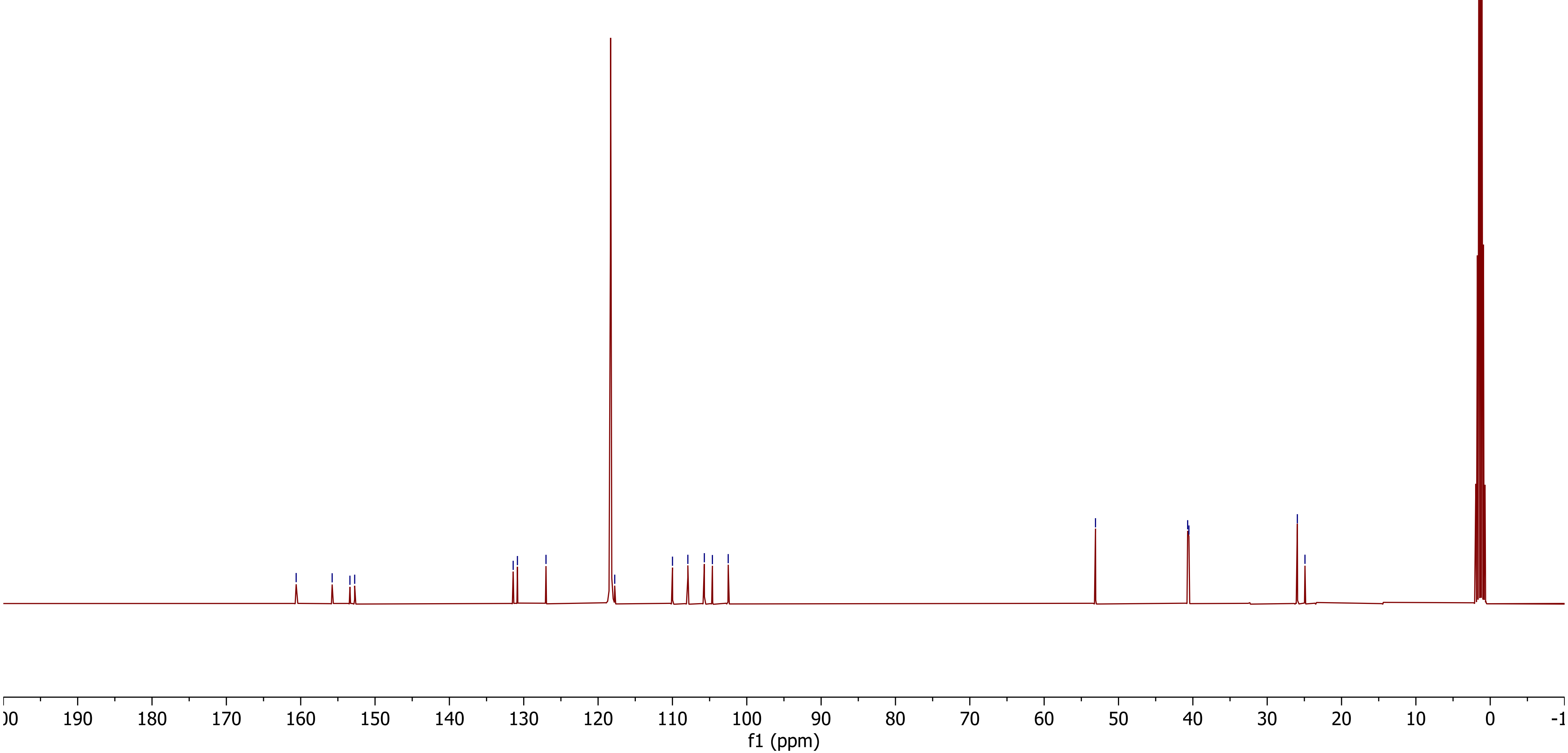

160.61
155.77
153.38
152.74
131.42
130.85
127.01
117.76
109.99
107.93
105.71
104.63
102.50
53.10
40.70
40.53
25.94
24.92
1.32 CD3CN
200
190
180
170
160
150
140
130
120
110
100
90
80
70
60
50
40
30
20
10
0
-1
f1 (ppm)

Compound 7

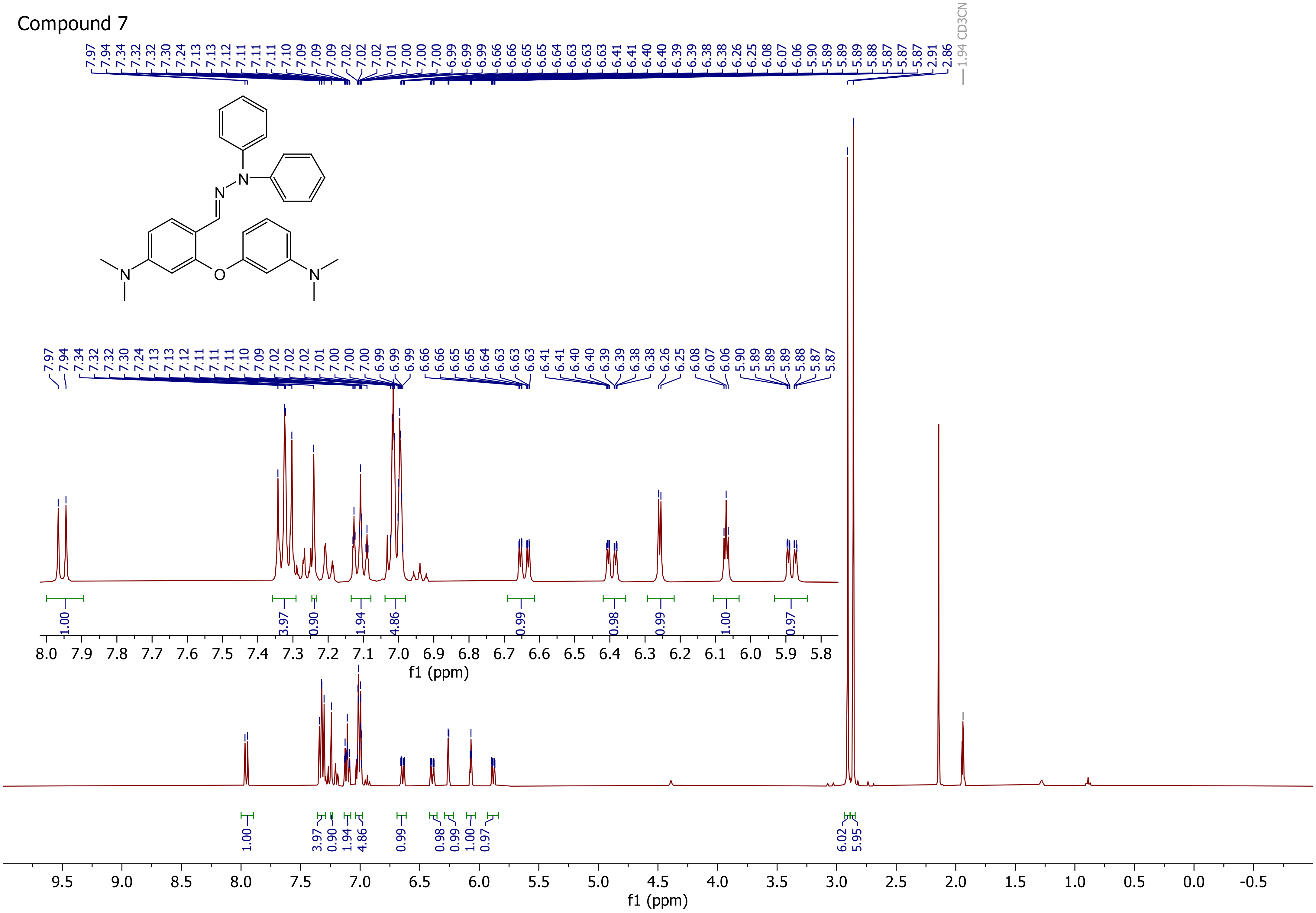

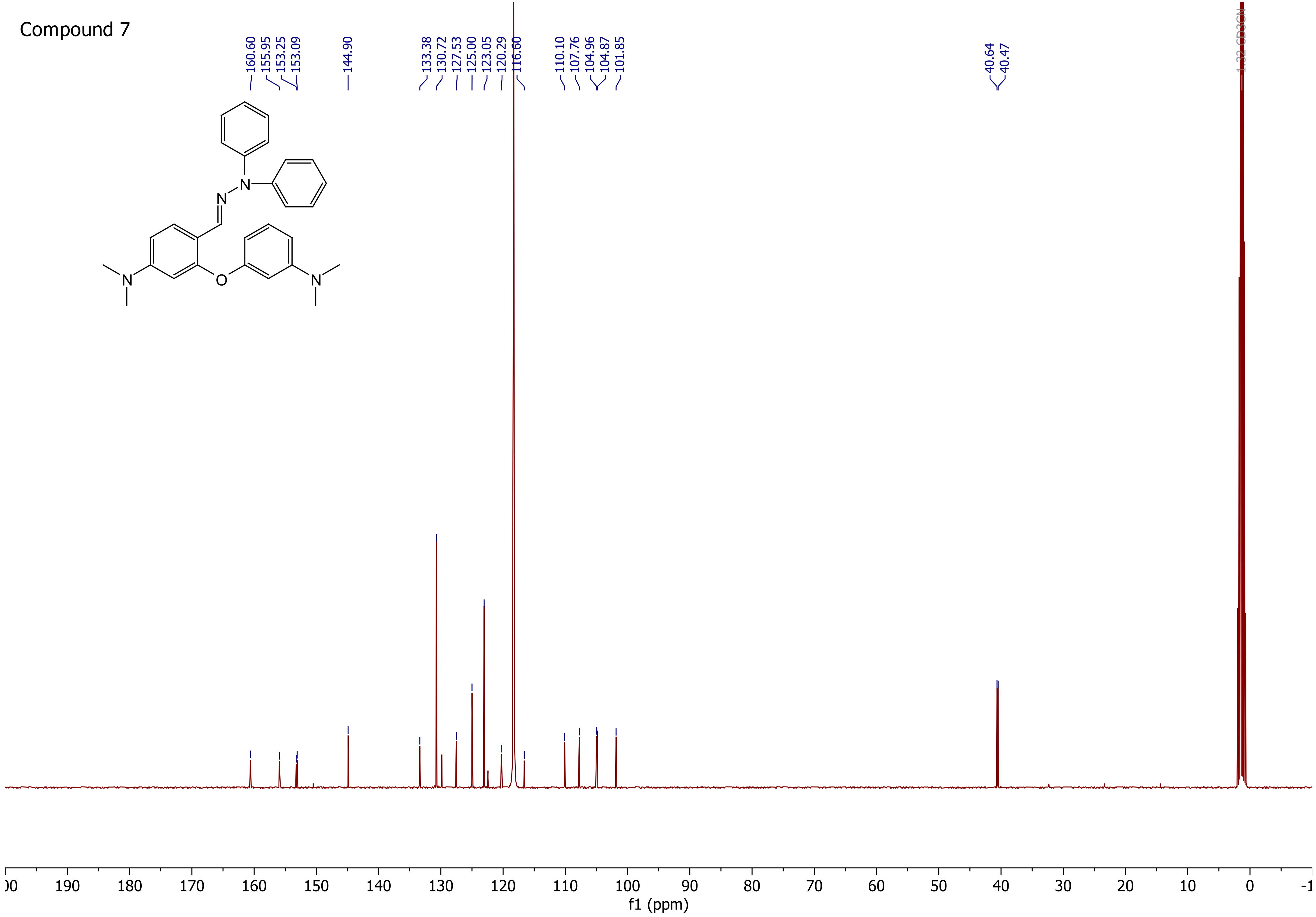

Compound 7
160.60
155.95
153.25
153.09
144.90
133.38
130.72
127.53
125.00
123.05
120.29
116.60
110.10
107.76
104.96
104.87
101.85
40.64
40.47
1.32 CD3CN
N
N
O
N
N
190 180 170 160 150 140 130 120 110 100 90 80 70 60 50 40 30 20 10 0 -1
f1 (ppm)

Compound 8

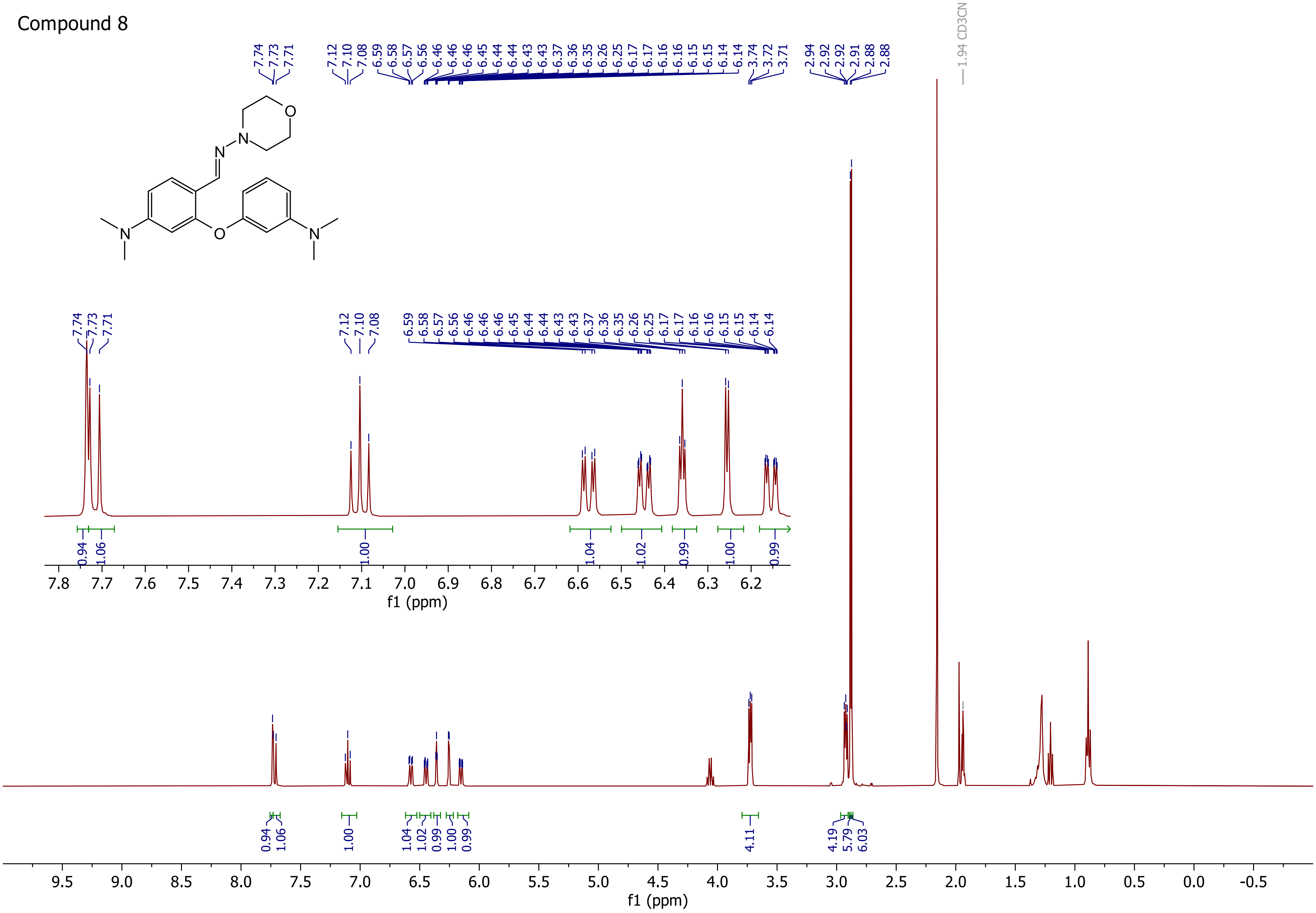

7.74
7.73
7.71
7.12
7.10
7.08
6.59
6.58
6.57
6.56
6.46
6.46
6.46
6.45
6.44
6.44
6.43
6.43
6.37
6.36
6.35
6.26
6.25
6.17
6.17
6.16
6.16
6.15
6.15
6.14
6.14
3.74
3.72
3.71
2.94
2.92
2.92
2.91
2.88
2.88
1.94 CD3CN
O
N
N
O
N
N
0.94
1.06
1.00
1.04
1.02
0.99
1.00
0.99
4.11
4.19
5.79
6.03
f1 (ppm)

Compound 8

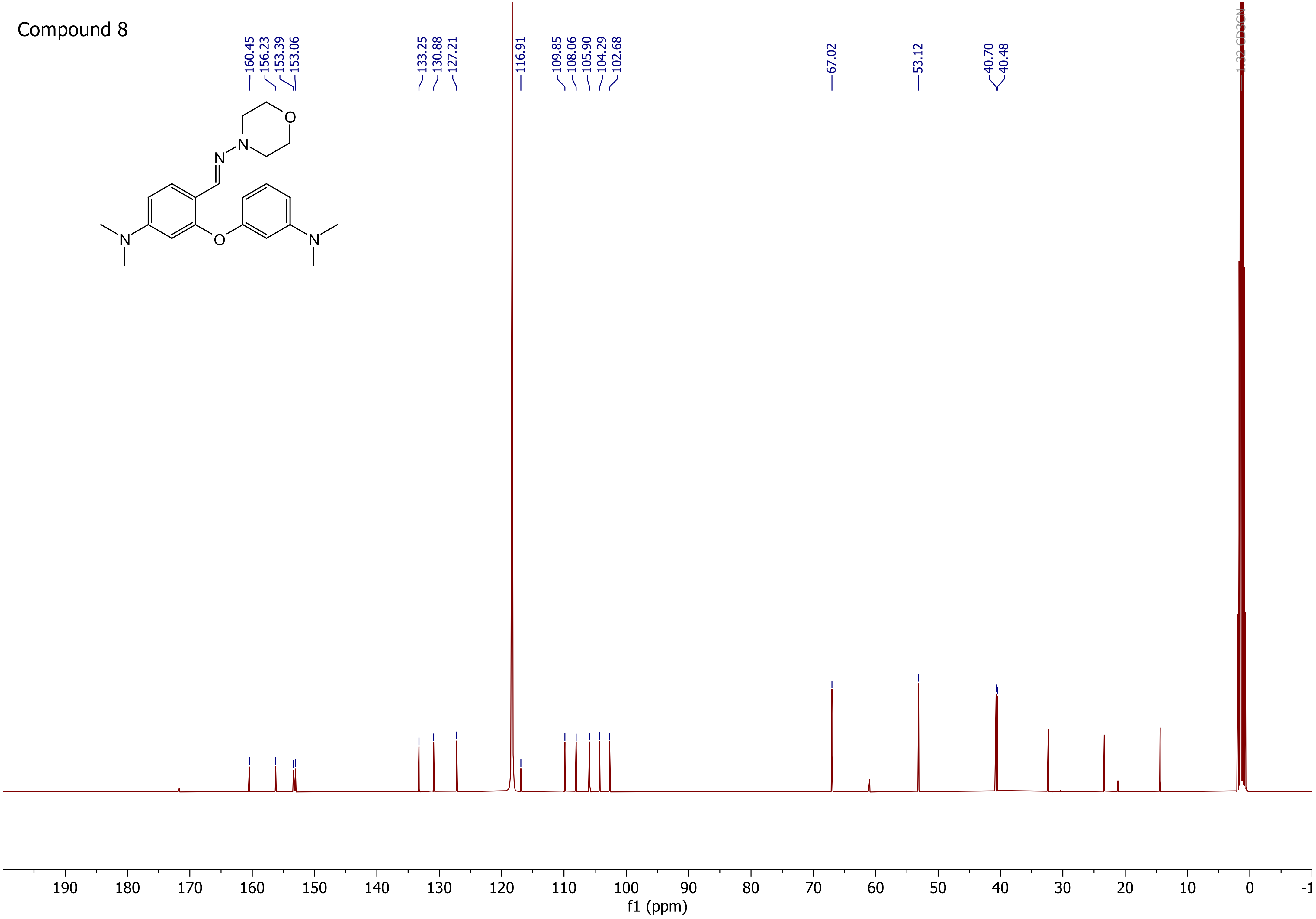

160.45
156.23
153.39
153.06
133.25
130.88
127.21
116.91
109.85
108.06
105.90
104.29
102.68
67.02
53.12
40.70
40.48
1.32 CD3CN
N
O
N
N
O
N
190 180 170 160 150 140 130 120 110 100 90 80 70 60 50 40 30 20 10 0 -1
f1 (ppm)

Compound 9

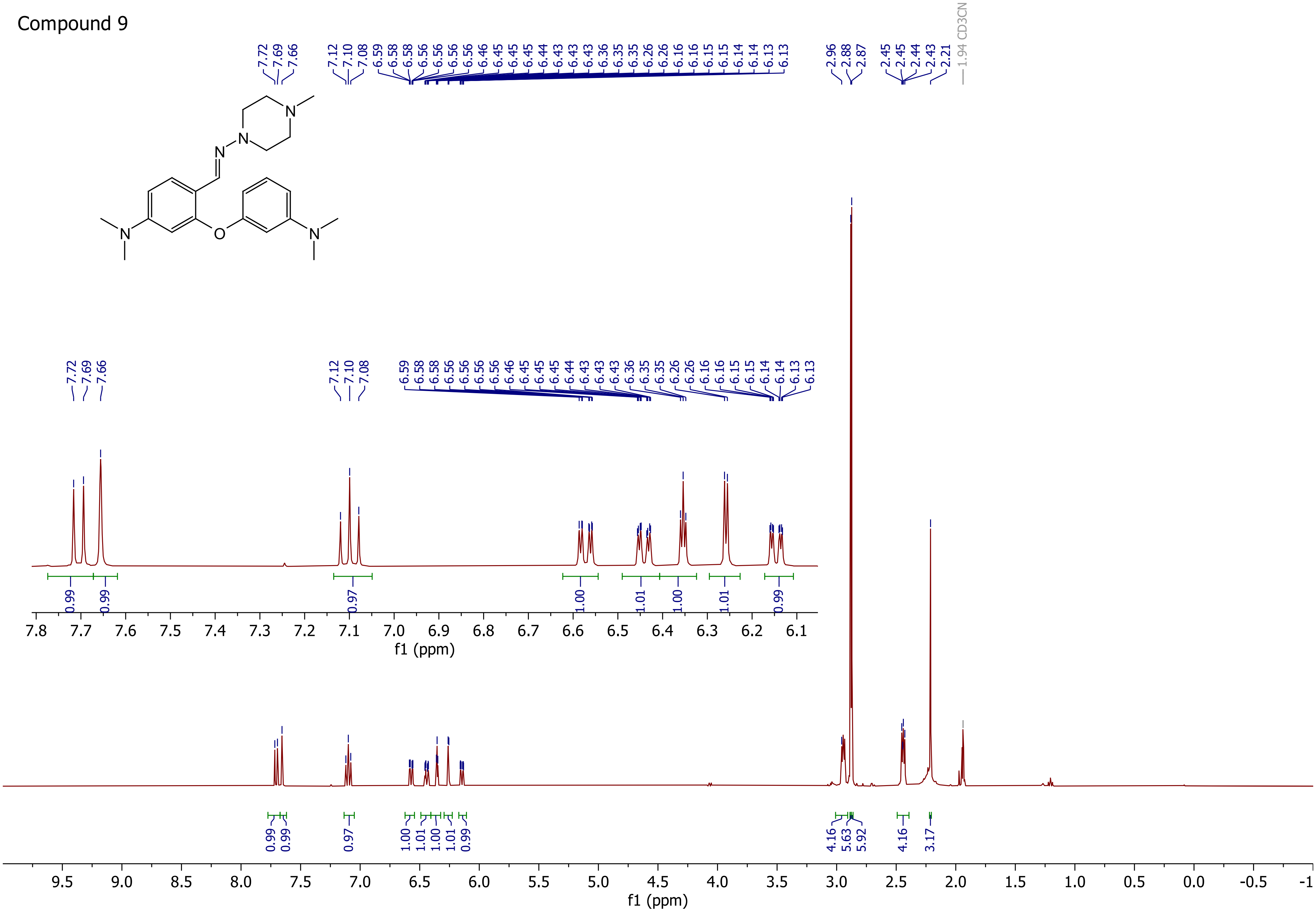

Compound 9

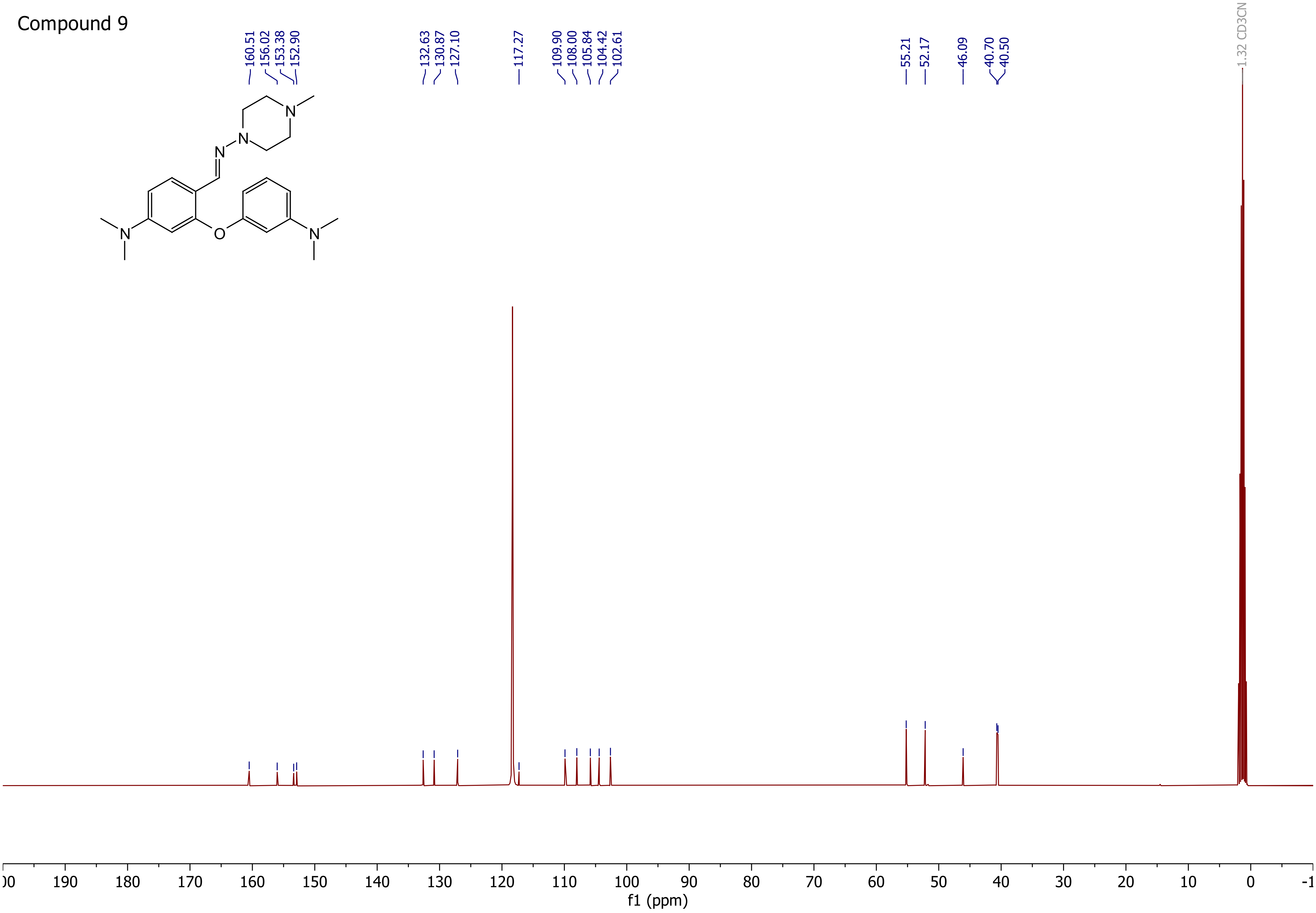

160.51
156.02
153.38
152.90
132.63
130.87
127.10
117.27
109.90
108.00
105.84
104.42
102.61
55.21
52.17
46.09
40.70
40.50
1.32 CD3CN
f1 (ppm)

Compound 10

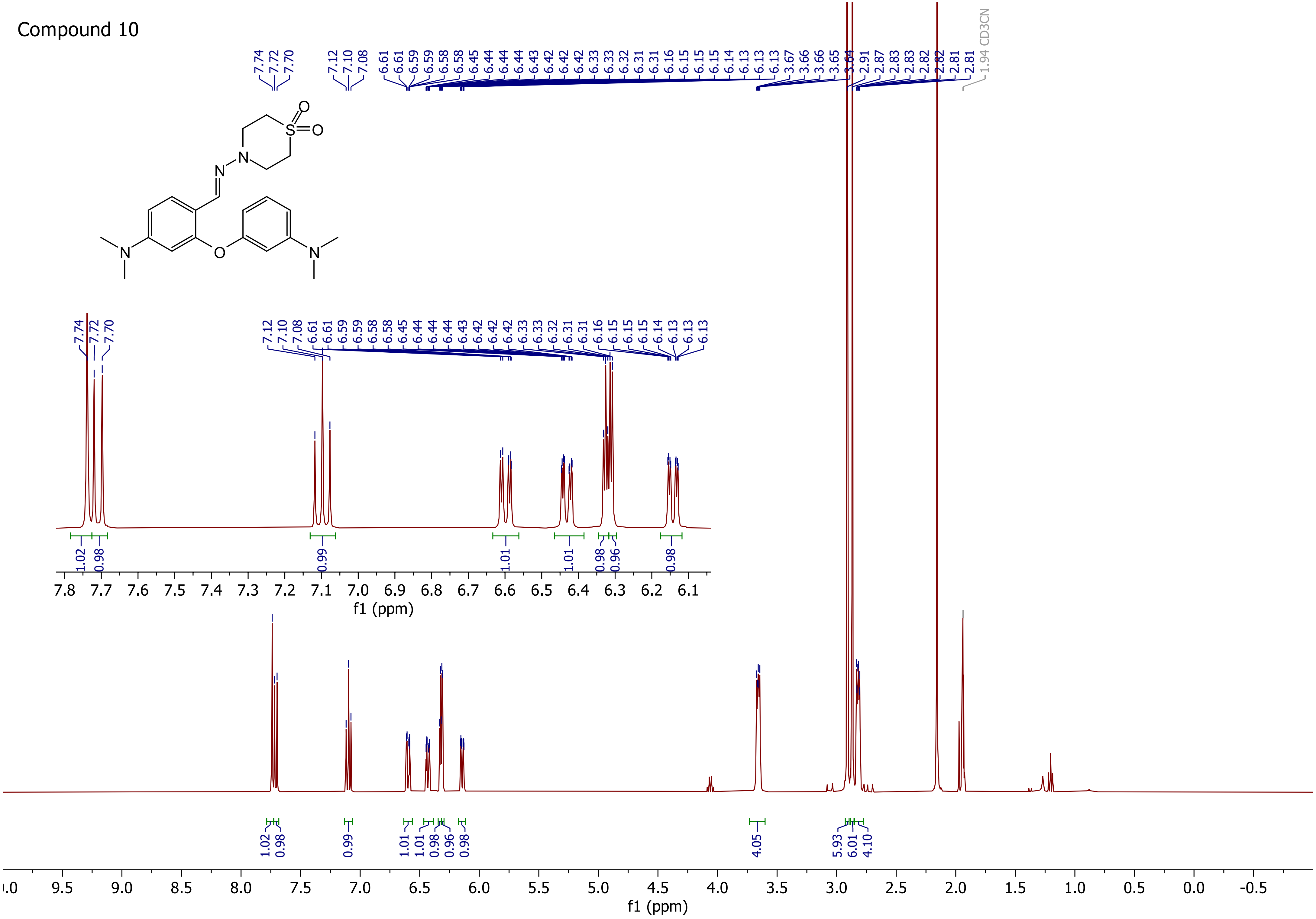
7.74
7.72
7.70
7.12
7.10
7.08
6.61
6.61
6.59
6.59
6.58
6.58
6.45
6.44
6.44
6.44
6.43
6.42
6.42
6.42
6.33
6.33
6.32
6.31
6.31
6.16
6.15
6.15
6.15
6.14
6.13
6.13
6.13
3.67
3.66
3.66
3.65
3.64
2.91
2.87
2.83
2.83
2.82
2.82
2.81
1.94 CD3CN
1.02
0.98
0.99
1.01
1.01
0.98
0.96
0.98
4.05
5.93
6.01
4.10
f1 (ppm)

Compound 10

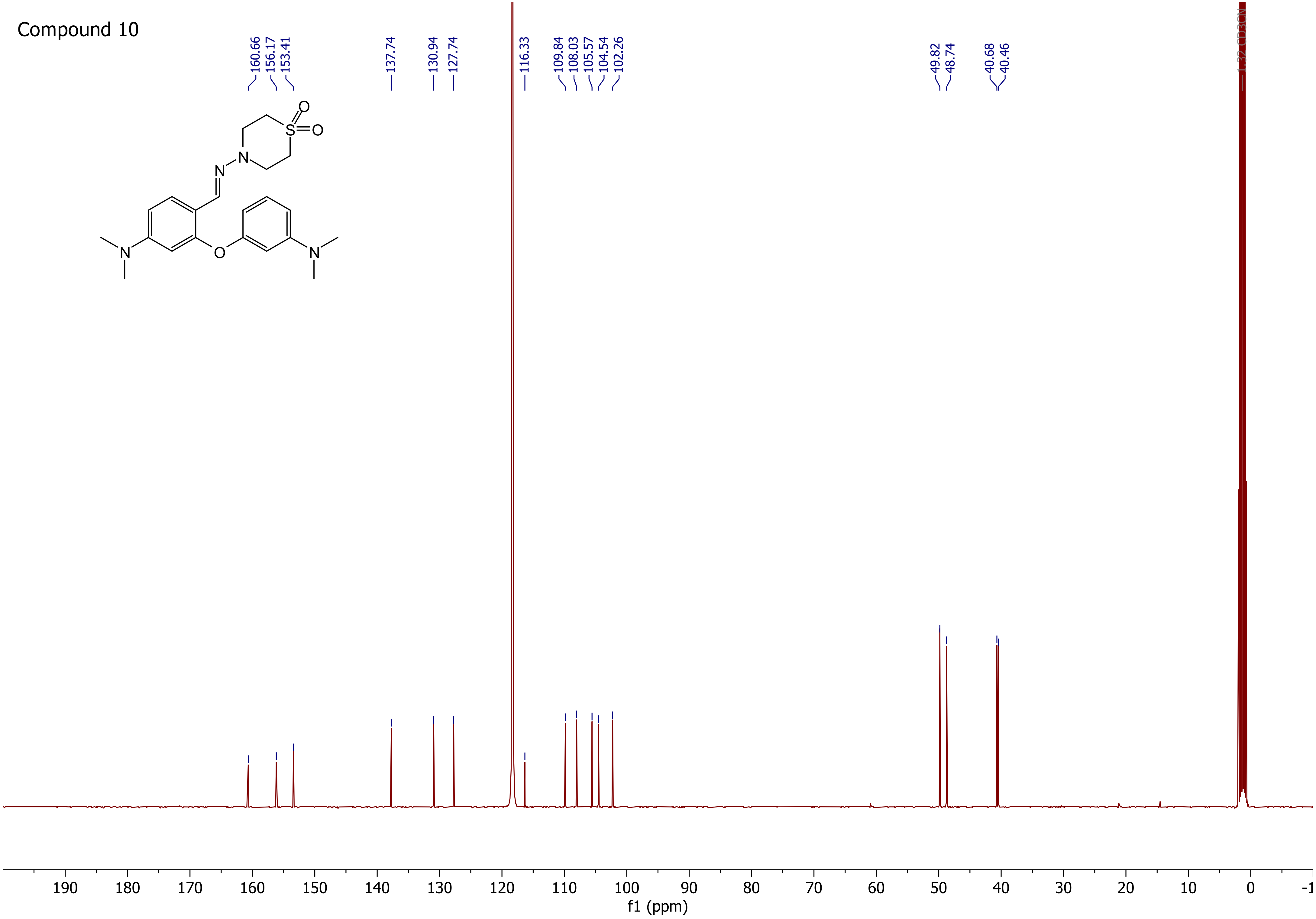
160.66
156.17
153.41
137.74
130.94
127.74
116.33
109.84
108.03
105.57
104.54
102.26
49.82
48.74
40.68
40.46
1.32 CD3CN
190 180 170 160 150 140 130 120 110 100 90 80 70 60 50 40 30 20 10 0 -1
f1 (ppm)

Compound 11

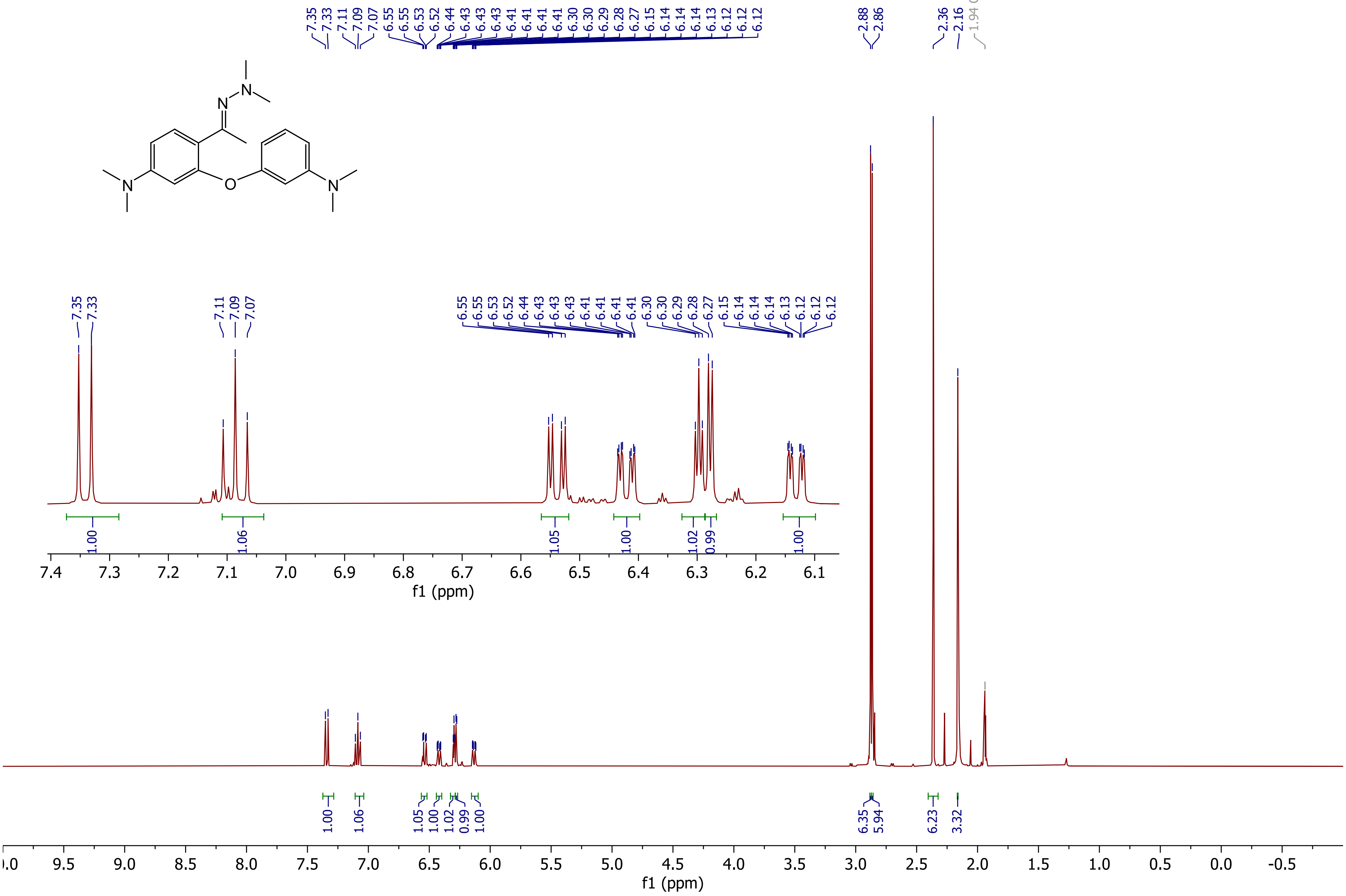
7.35
7.33
7.11
7.09
7.07
6.55
6.55
6.53
6.52
6.44
6.43
6.43
6.43
6.41
6.41
6.41
6.41
6.30
6.30
6.29
6.28
6.27
6.15
6.14
6.14
6.14
6.13
6.12
6.12
6.12
2.88
2.86
2.36
2.16
1.94 CD3CN
1.00
1.06
1.05
1.00
1.02
0.99
1.00
6.35
5.94
6.23
3.32
f1 (ppm)

Compound 11

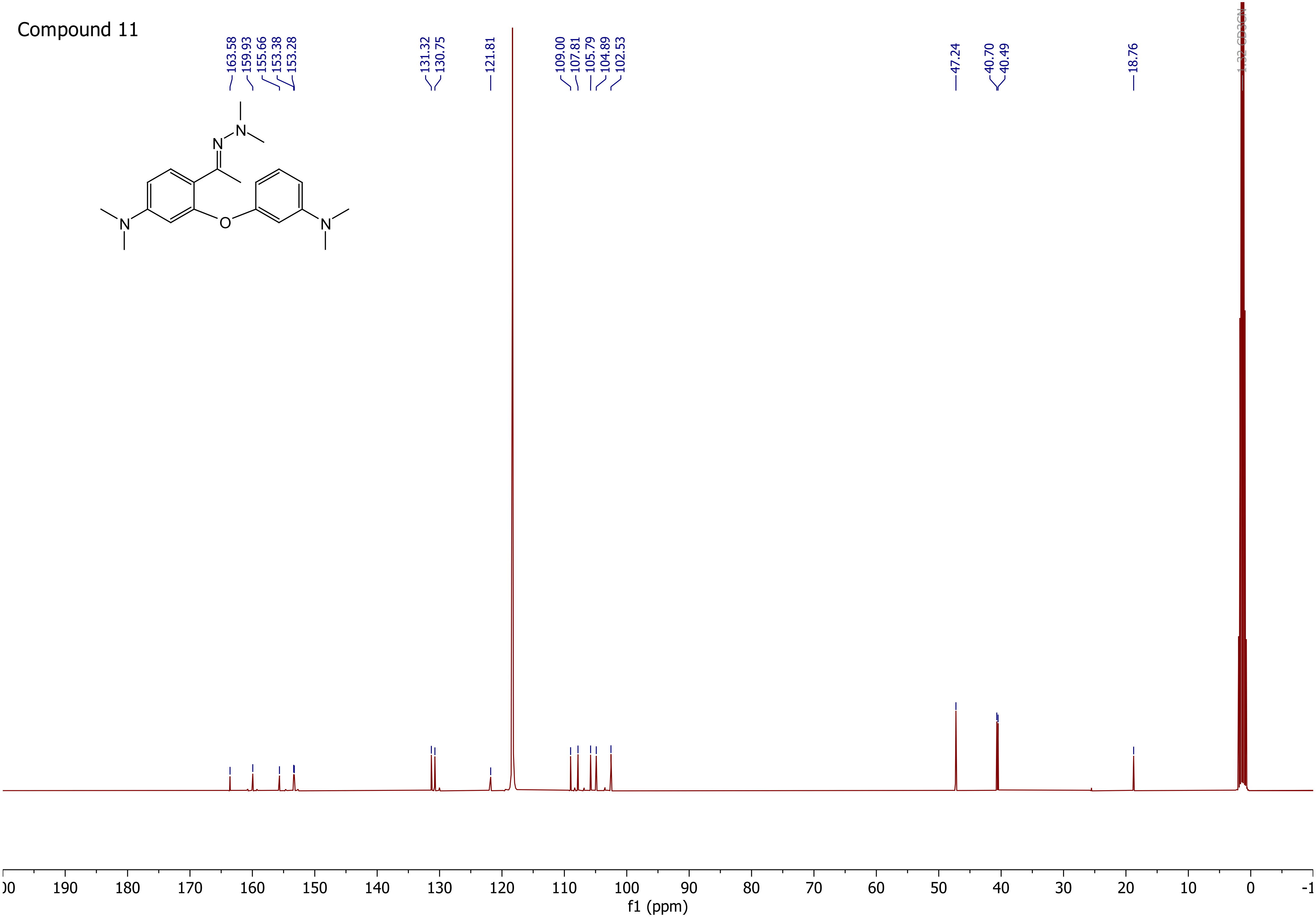

163.58
159.93
155.66
153.38
153.28
131.32
130.75
121.81
109.00
107.81
105.79
104.89
102.53
47.24
40.70
40.49
18.76
1.32 CD3CN
N
N
O
N
N
190
180
170
160
150
140
130
120
110
100
90
80
70
60
50
40
30
20
10
0
-1
f1 (ppm)

Compound 14

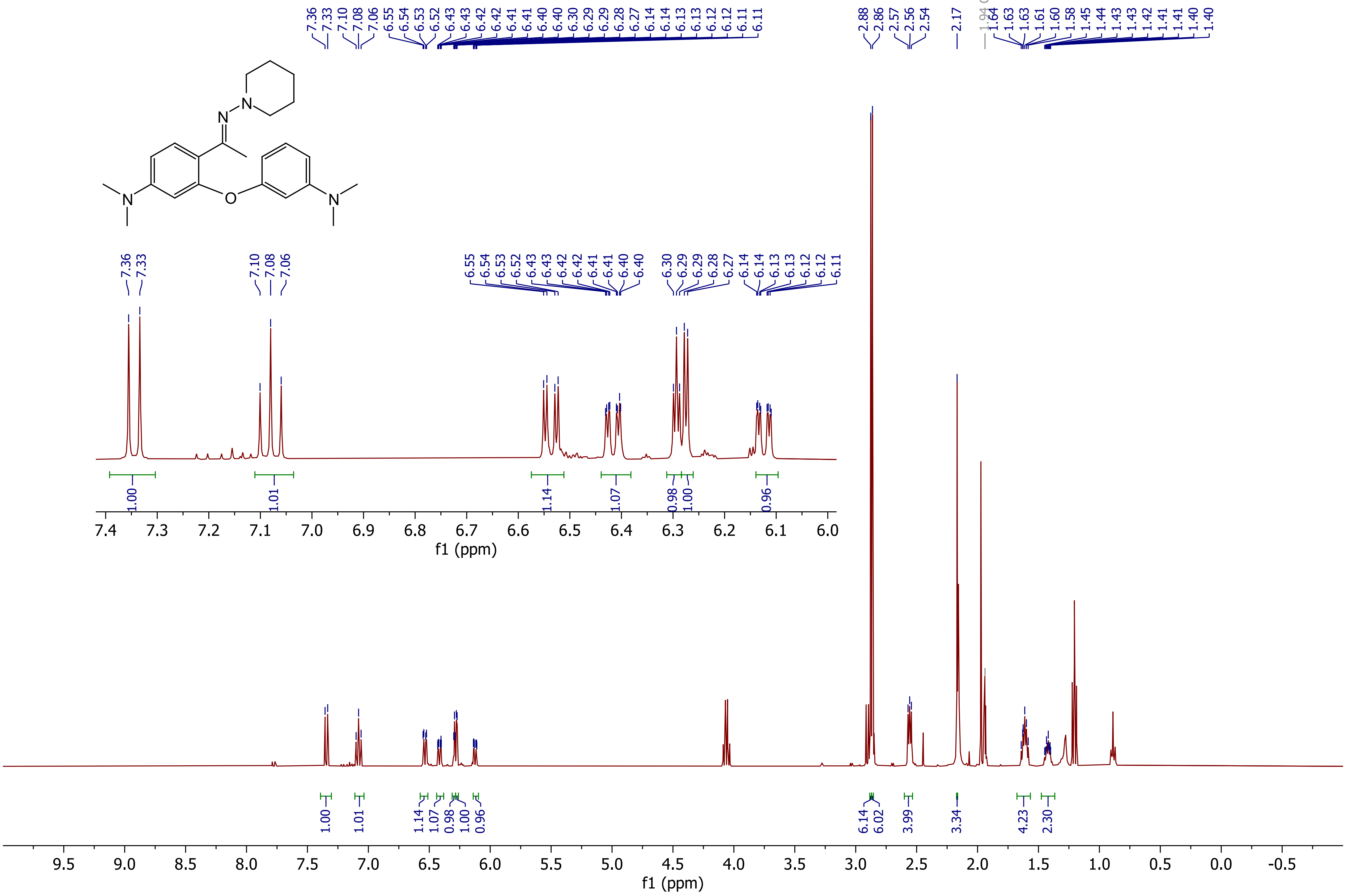

7.36
7.33
7.10
7.08
7.06
6.55
6.54
6.53
6.52
6.43
6.43
6.42
6.42
6.41
6.41
6.40
6.40
6.30
6.29
6.29
6.28
6.27
6.14
6.14
6.13
6.13
6.12
6.12
6.11
6.11
2.88
2.86
2.57
2.56
2.54
2.17
1.94 CD3CN
1.64
1.63
1.63
1.61
1.60
1.58
1.45
1.44
1.43
1.43
1.42
1.41
1.41
1.40
1.40
1.00
1.01
1.14
1.07
0.98
1.00
0.96
6.14
6.02
3.99
3.34
4.23
2.30
f1 (ppm)

Compound 14

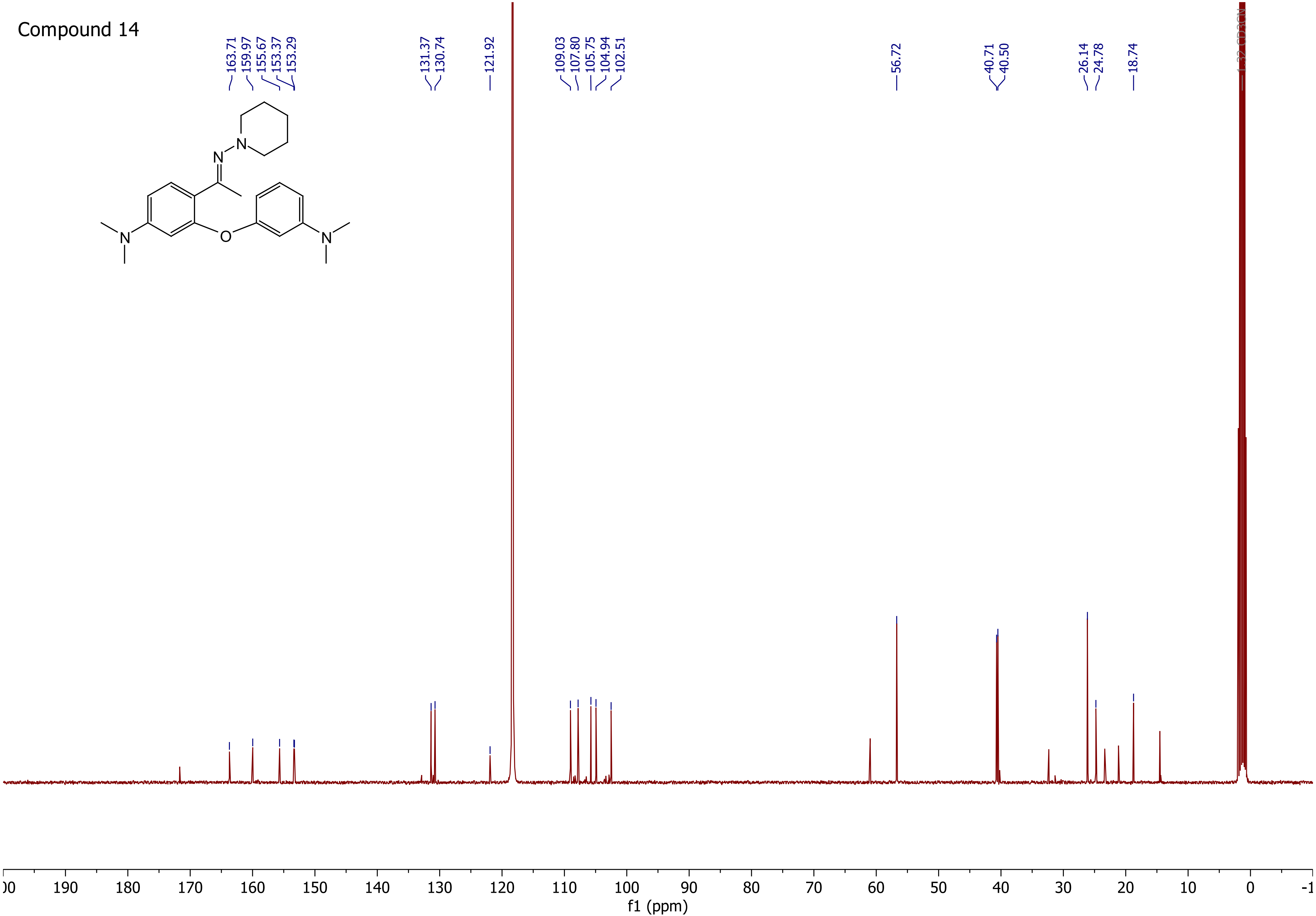

163.71
159.97
155.67
153.37
153.29
131.37
130.74
121.92
109.03
107.80
105.75
104.94
102.51
56.72
40.71
40.50
26.14
24.78
18.74
1.32 CD3CN
f1 (ppm)

Compound 13

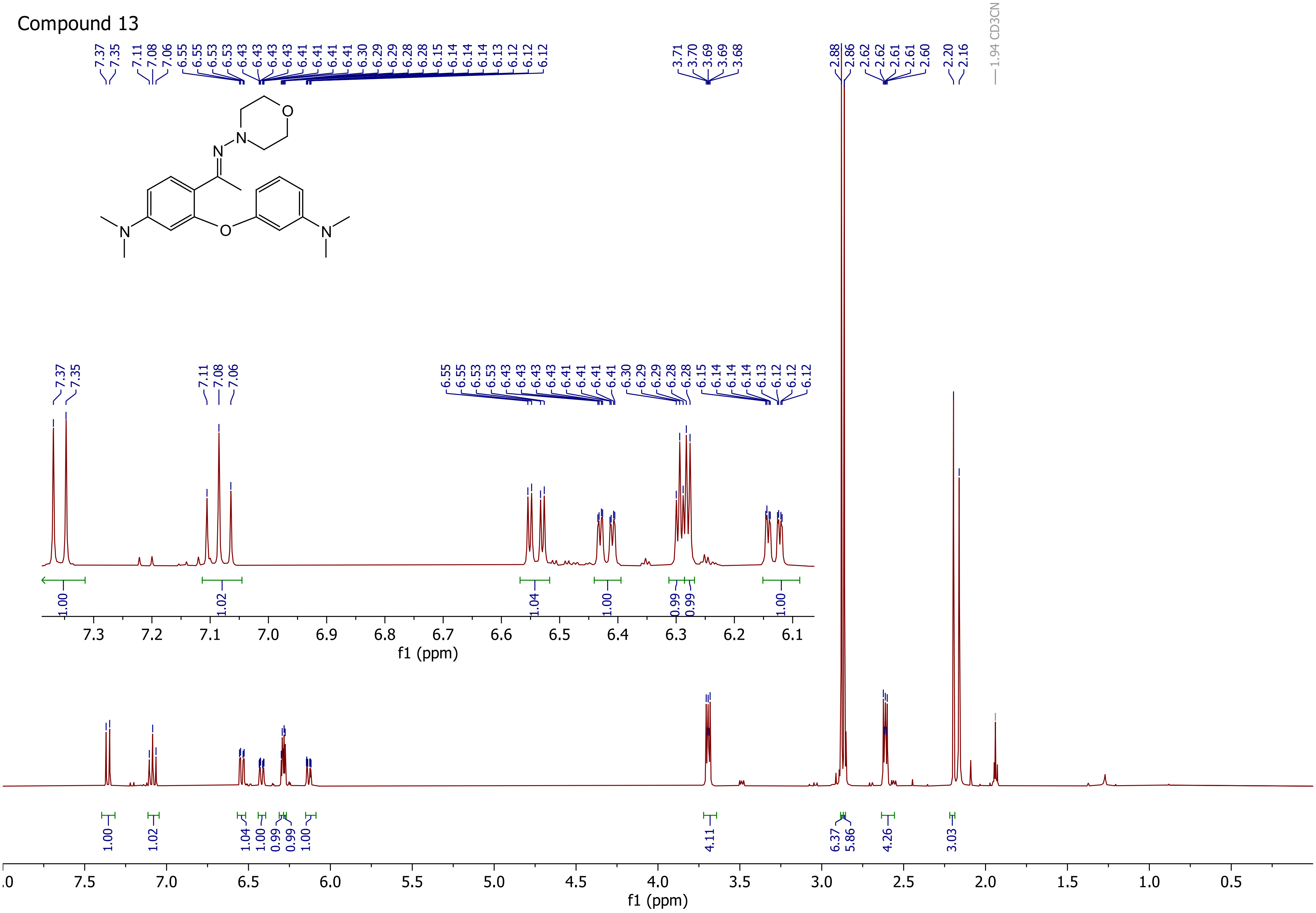
7.37
7.35
7.11
7.08
7.06
6.55
6.55
6.53
6.53
6.43
6.43
6.43
6.41
6.41
6.41
6.41
6.30
6.29
6.29
6.28
6.28
6.15
6.14
6.14
6.14
6.13
6.12
6.12
6.12
3.71
3.70
3.69
3.69
3.68
2.88
2.86
2.62
2.62
2.61
2.61
2.60
2.20
2.16
1.94 CD3CN
O
N
N
O
N
N
1.00
1.02
1.04
1.00
0.99
0.99
1.00
4.11
6.37
5.86
4.26
3.03
f1 (ppm)

Compound 13

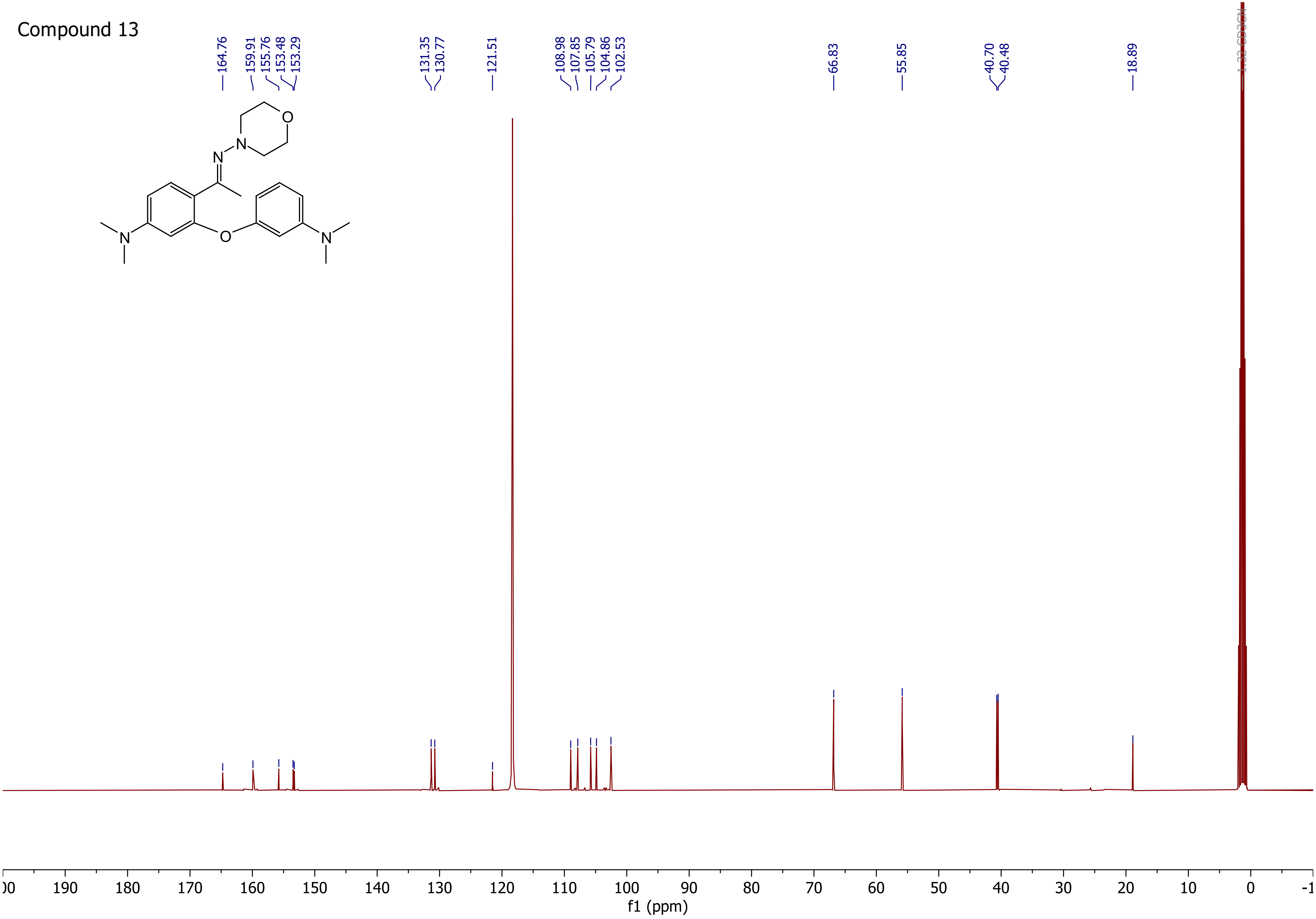

164.76
159.91
155.76
153.48
153.29
131.35
130.77
121.51
108.98
107.85
105.79
104.86
102.53
66.83
55.85
40.70
40.48
18.89
1.32 CD3CN
N
O
N
O
N
N
190 180 170 160 150 140 130 120 110 100 90 80 70 60 50 40 30 20 10 0 -1
f1 (ppm)

Compound 14

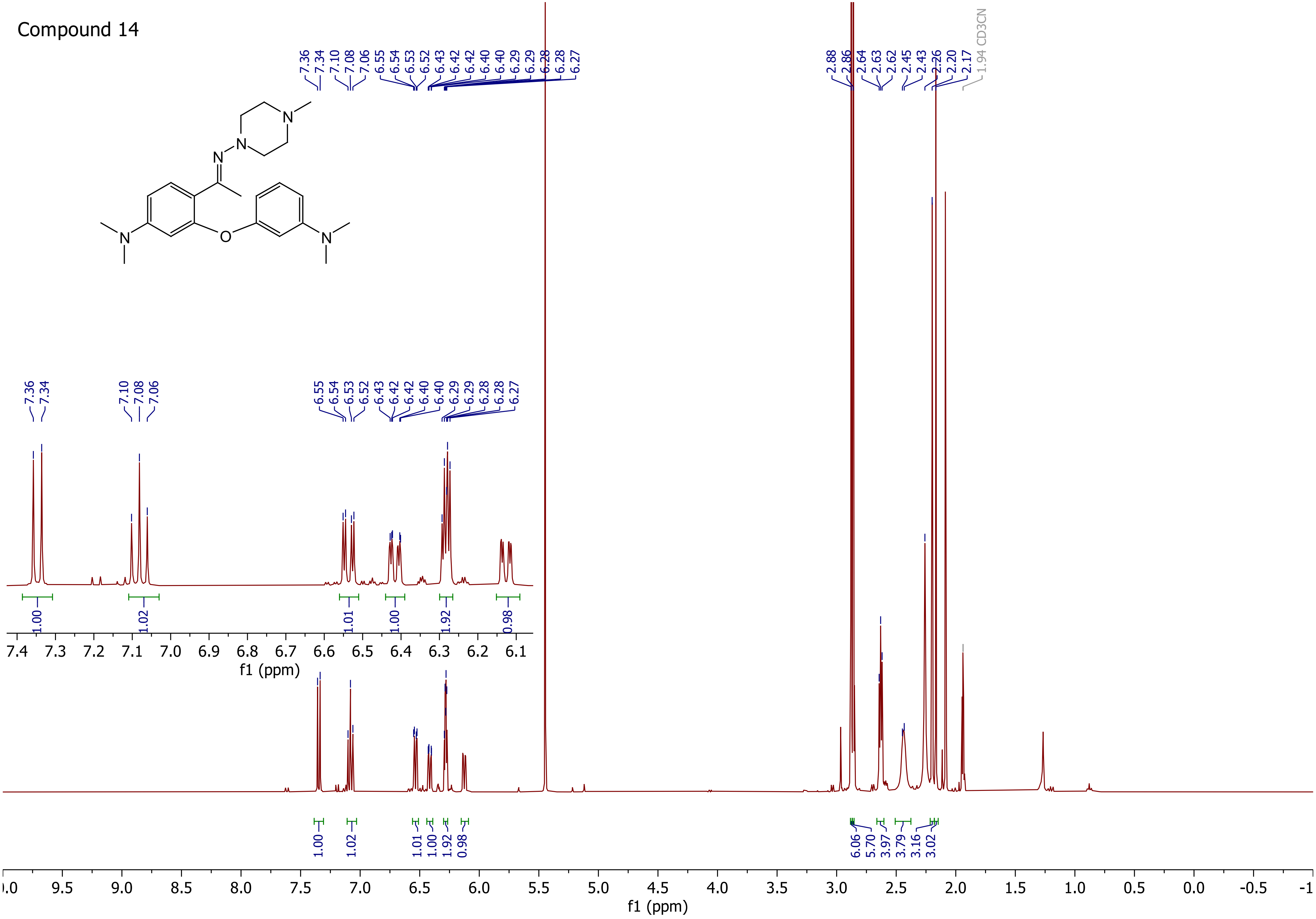
7.36
7.34
7.10
7.08
7.06
6.55
6.54
6.53
6.52
6.43
6.42
6.42
6.40
6.40
6.29
6.29
6.28
6.28
6.27
2.88
2.86
2.64
2.63
2.62
2.45
2.43
2.26
2.20
2.17
1.94 CD3CN
1.00
1.02
1.01
1.00
1.92
0.98
6.06
5.70
3.97
3.79
3.16
3.02
f1 (ppm)

Compound 14

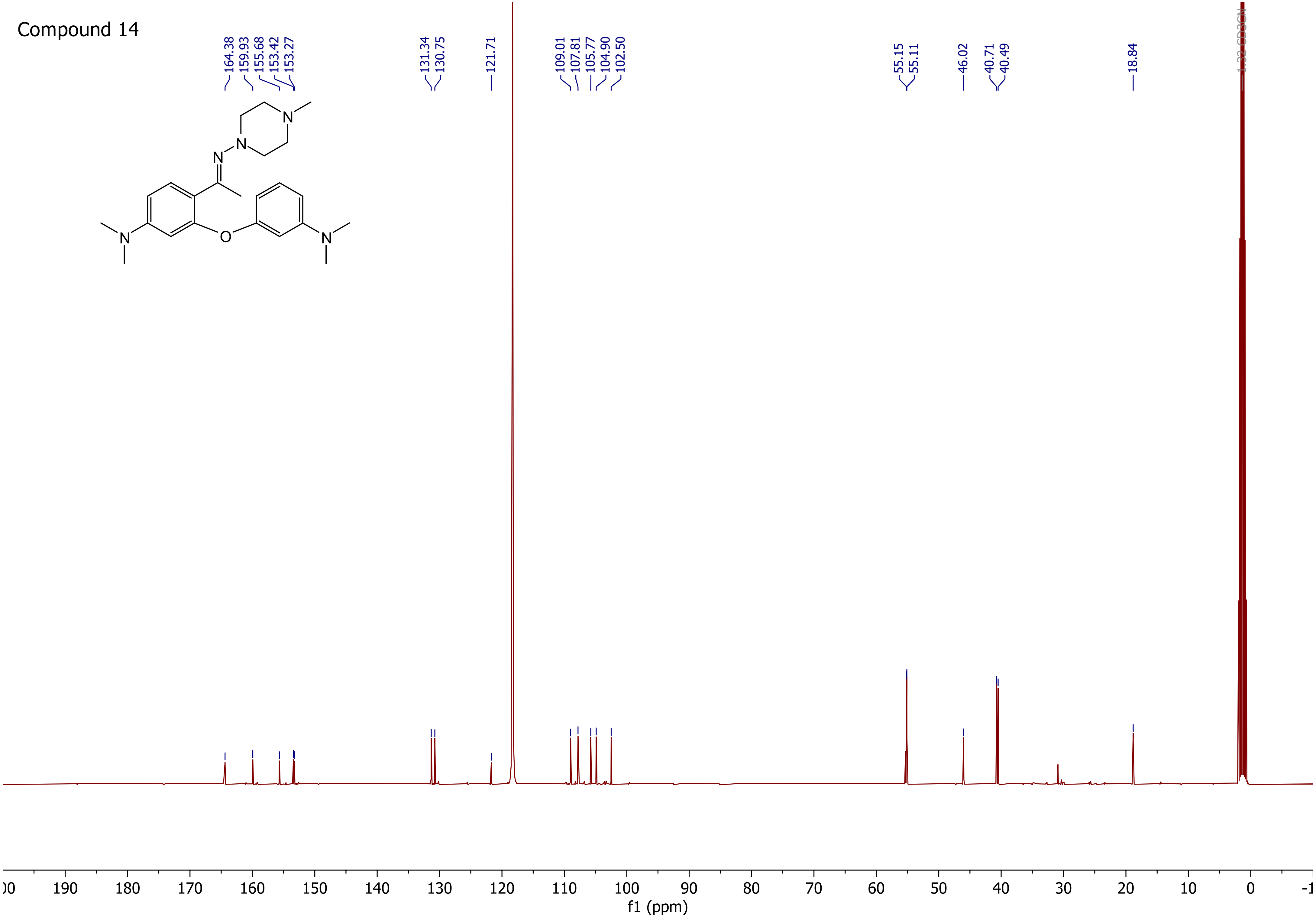

164.38
159.93
155.68
153.42
153.27
131.34
130.75
121.71
109.01
107.81
105.77
104.90
102.50
55.15
55.11
46.02
40.71
40.49
18.84
1.32 CD3CN
N
N
N
N
O
N
f1 (ppm)

Compound 15

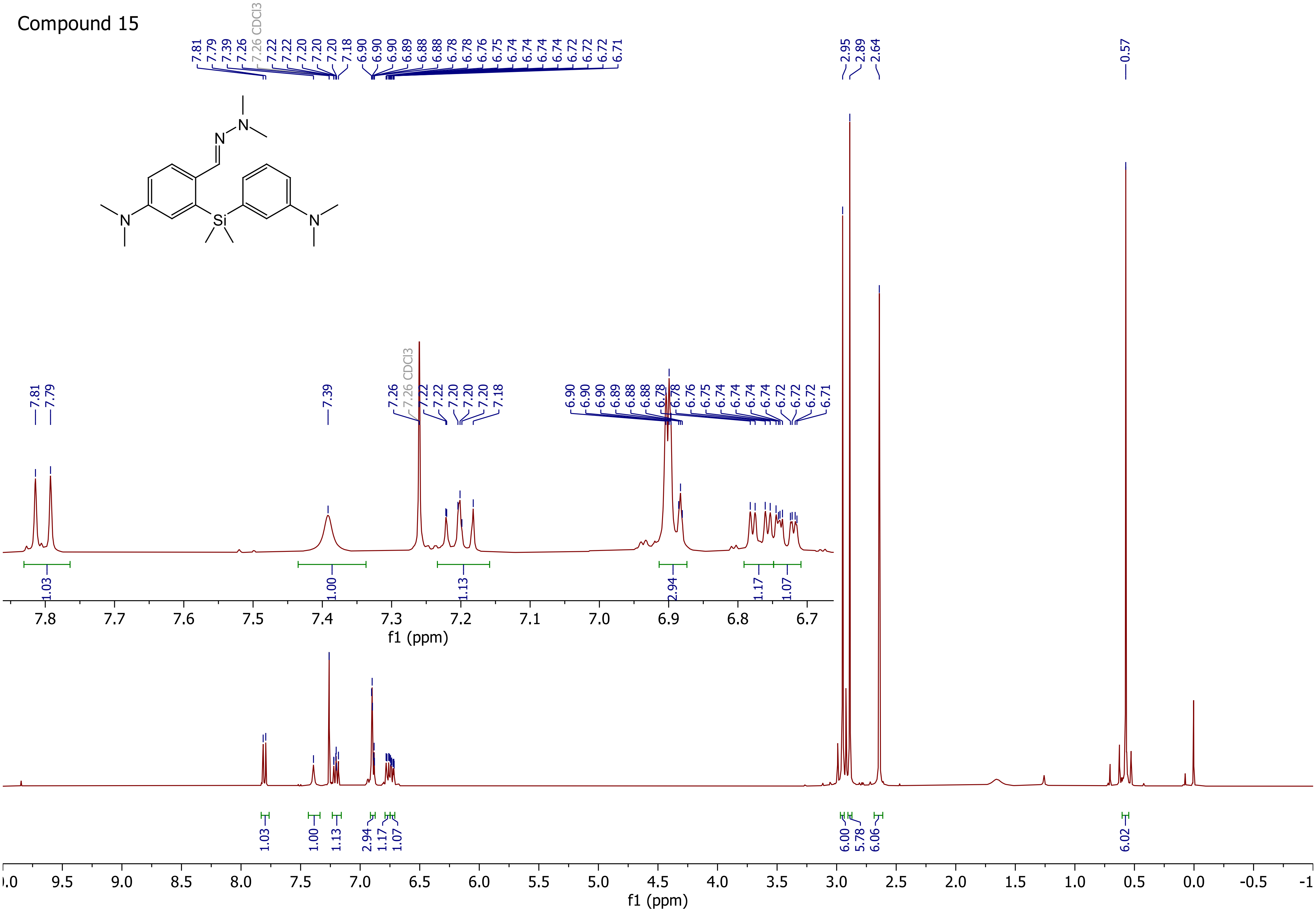

N
N
Si
N
N
7.81
7.79
7.39
7.26
7.26 CDCl3
7.22
7.22
7.20
7.20
7.20
7.18
6.90
6.90
6.90
6.89
6.88
6.88
6.78
6.78
6.76
6.75
6.74
6.74
6.74
6.74
6.72
6.72
6.72
6.71
2.95
2.89
2.64
0.57
1.03
1.00
1.13
2.94
1.17
1.07
6.00
5.78
6.06
6.02
f1 (ppm)

Compound 15

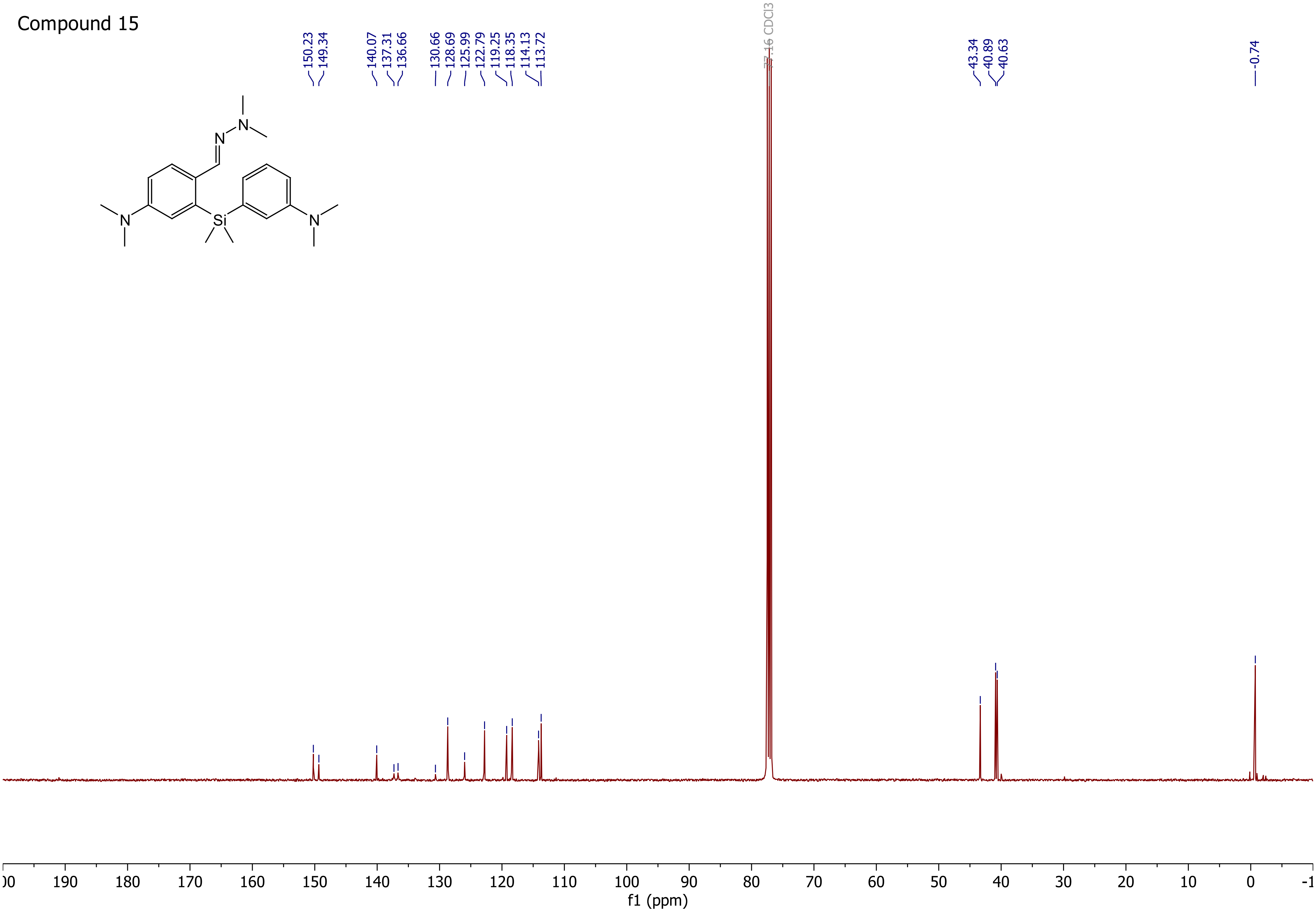
150.23
149.34
140.07
137.31
136.66
130.66
128.69
125.99
122.79
119.25
118.35
114.13
113.72
77.16 CDCl3
43.34
40.89
40.63
-0.74
N
N
Si
N
N
f1 (ppm)

Compound 16

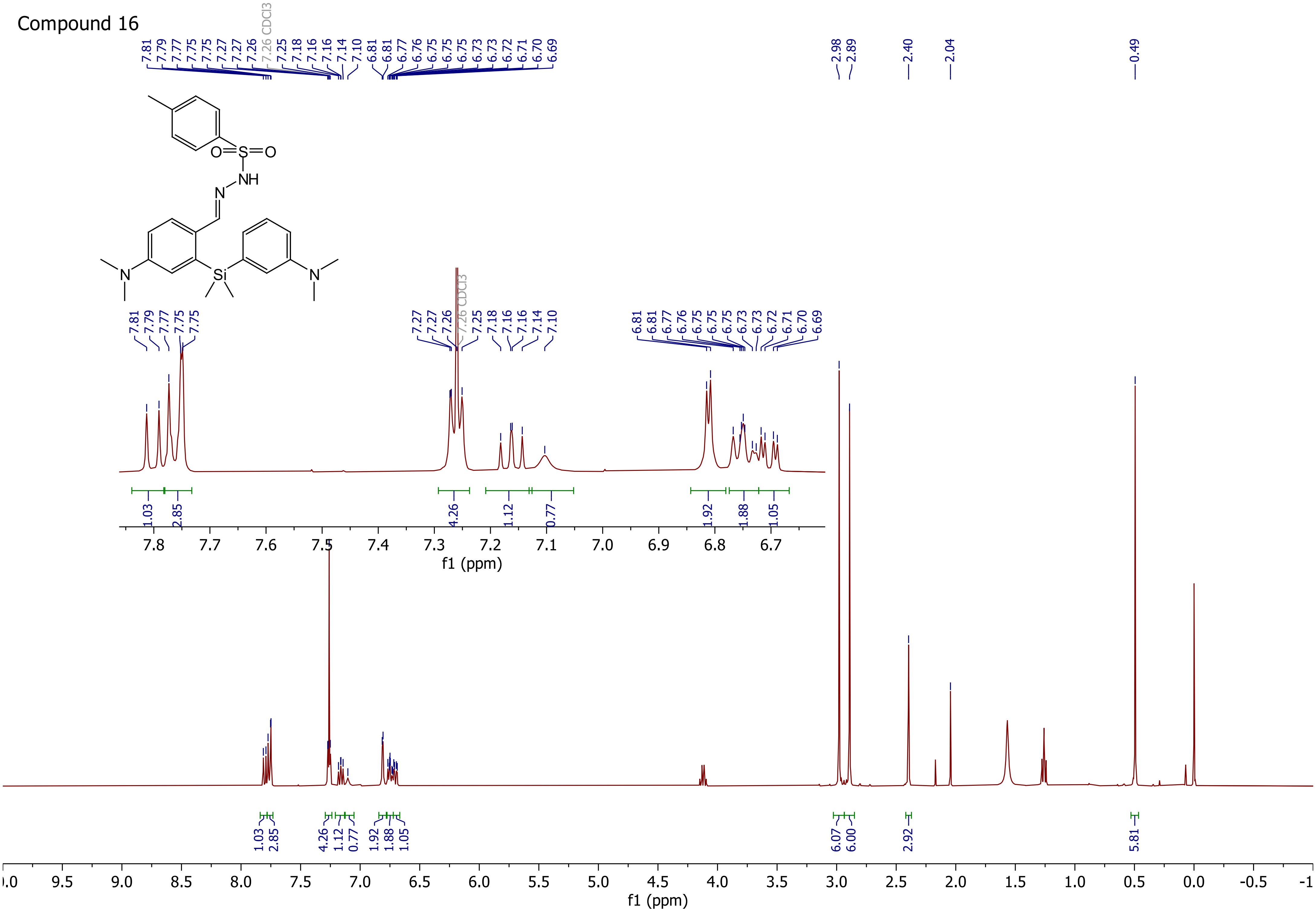

7.81
7.79
7.77
7.75
7.75
7.27
7.27
7.26
7.26 CDCl3
7.25
7.18
7.16
7.16
7.14
7.10
6.81
6.81
6.77
6.76
6.75
6.75
6.75
6.73
6.73
6.72
6.71
6.70
6.69
2.98
2.89
2.40
2.04
0.49
O=S=O
NH
N
N
Si
N
1.03
2.85
4.26
1.12
0.77
1.92
1.88
1.05
6.07
6.00
2.92
5.81
f1 (ppm)

Compound 16

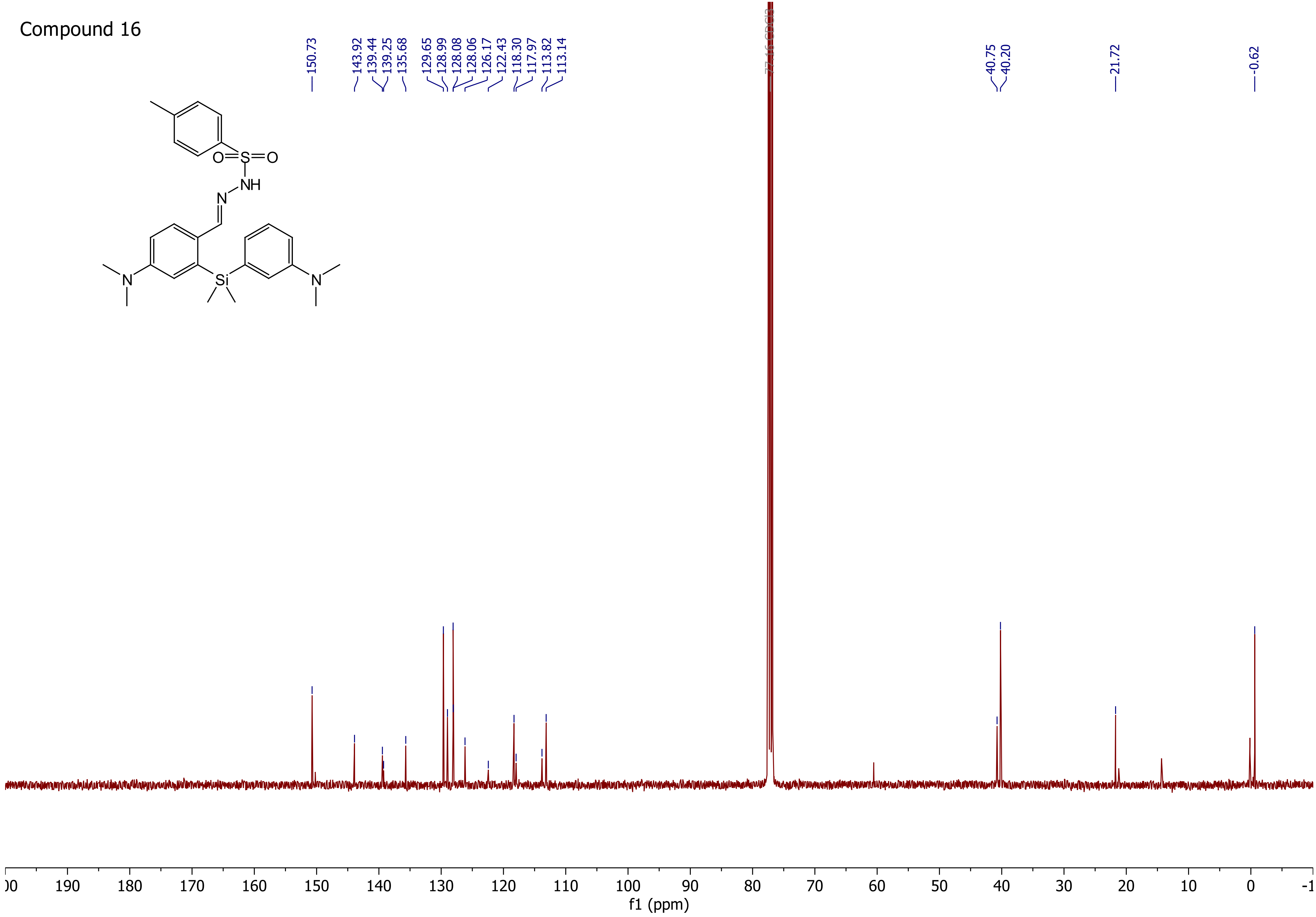
150.73
143.92
139.44
139.25
135.68
129.65
128.99
128.08
128.06
126.17
122.43
118.30
117.97
113.82
113.14
77.16 CDCl3
40.75
40.20
21.72
-0.62
f1 (ppm)

Compound 17

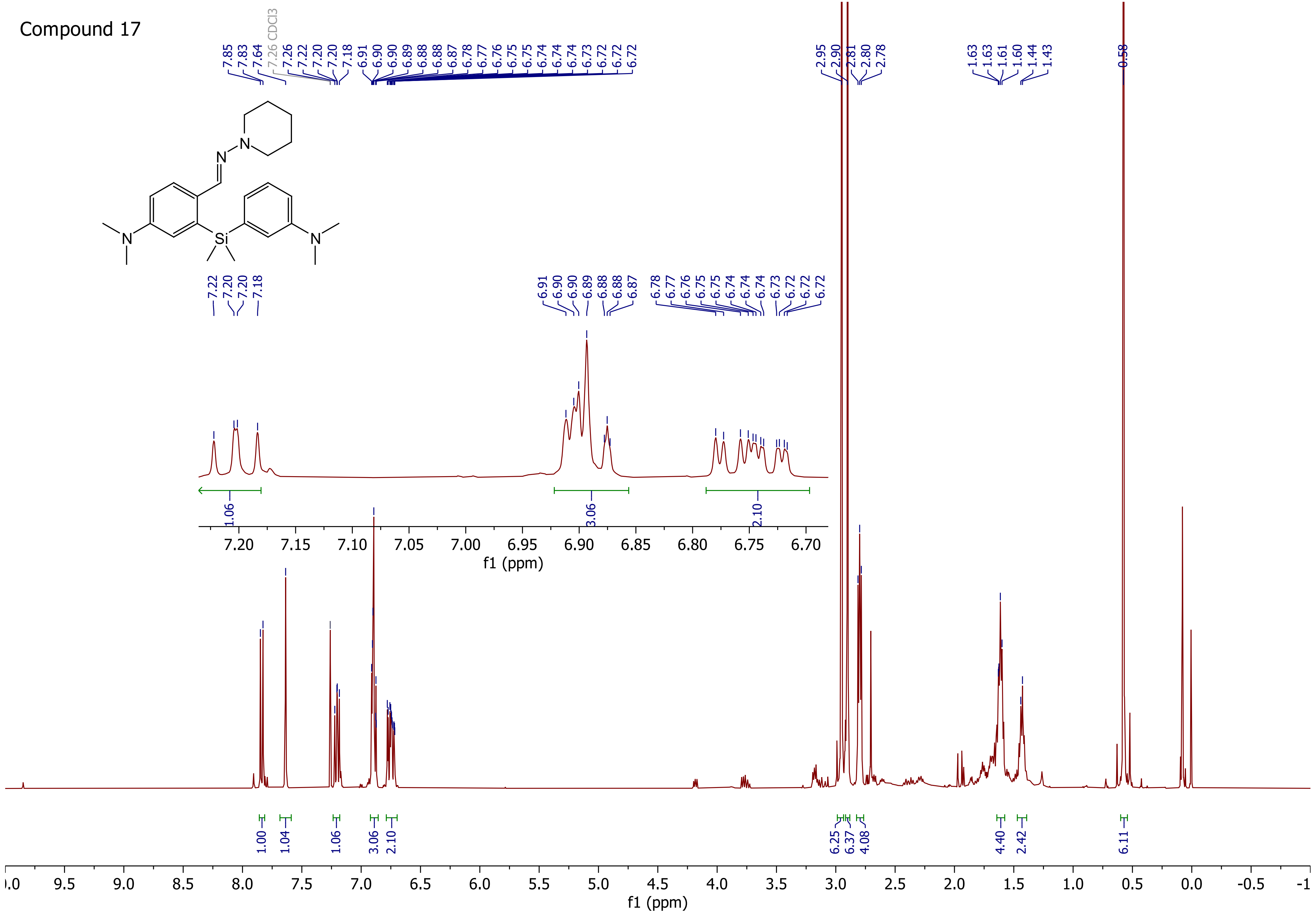

7.85
7.83
7.64
7.26 CDCl3
7.26
7.22
7.20
7.20
7.18
6.91
6.90
6.90
6.89
6.88
6.88
6.87
6.78
6.77
6.76
6.75
6.75
6.74
6.74
6.74
6.73
6.72
6.72
6.72
2.95
2.90
2.81
2.80
2.78
1.63
1.63
1.61
1.60
1.44
1.43
0.58
N
N
N
Si
N
1.06
3.06
2.10
7.20 7.15 7.10 7.05 7.00 6.95 6.90 6.85 6.80 6.75 6.70
f1 (ppm)
1.00
1.04
1.06
3.06
2.10
6.25
6.37
4.08
4.40
2.42
6.11
10.0 9.5 9.0 8.5 8.0 7.5 7.0 6.5 6.0 5.5 5.0 4.5 4.0 3.5 3.0 2.5 2.0 1.5 1.0 0.5 0.0 -0.5 -1
f1 (ppm)

Compound 17

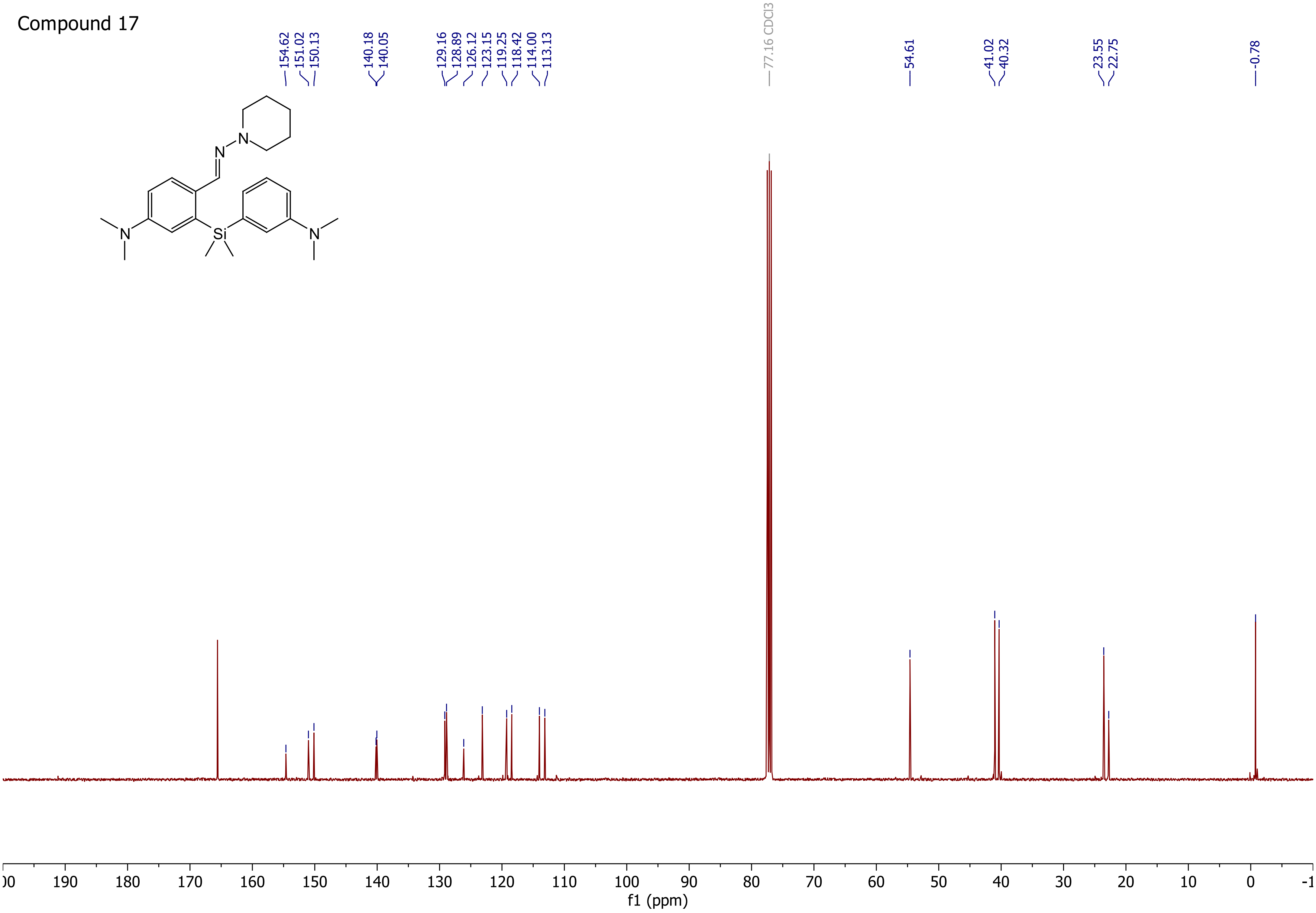

154.62
151.02
150.13
140.18
140.05
129.16
128.89
126.12
123.15
119.25
118.42
114.00
113.13
77.16 CDCl3
54.61
41.02
40.32
23.55
22.75
-0.78
N
N
N
Si
N
f1 (ppm)

Compound 18

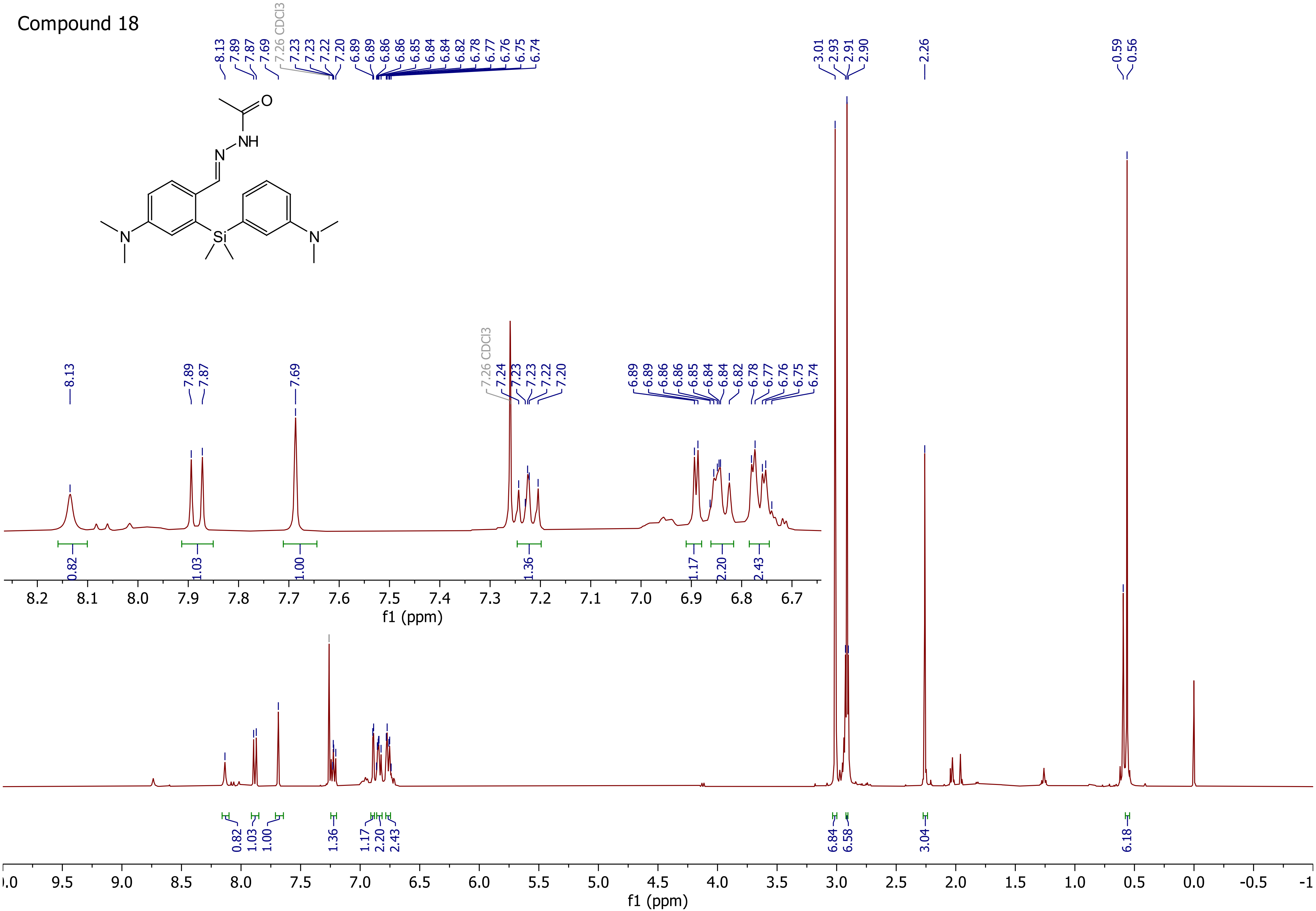
8.13
7.89
7.87
7.69
7.26 CDCl3
7.23
7.23
7.22
7.20
6.89
6.89
6.86
6.86
6.85
6.84
6.84
6.82
6.78
6.77
6.76
6.75
6.74
3.01
2.93
2.91
2.90
2.26
0.59
0.56
O
NH
N
N
Si
N
0.82
1.03
1.00
1.36
1.17
2.20
2.43
6.84
6.58
3.04
6.18
f1 (ppm)

Compound 18

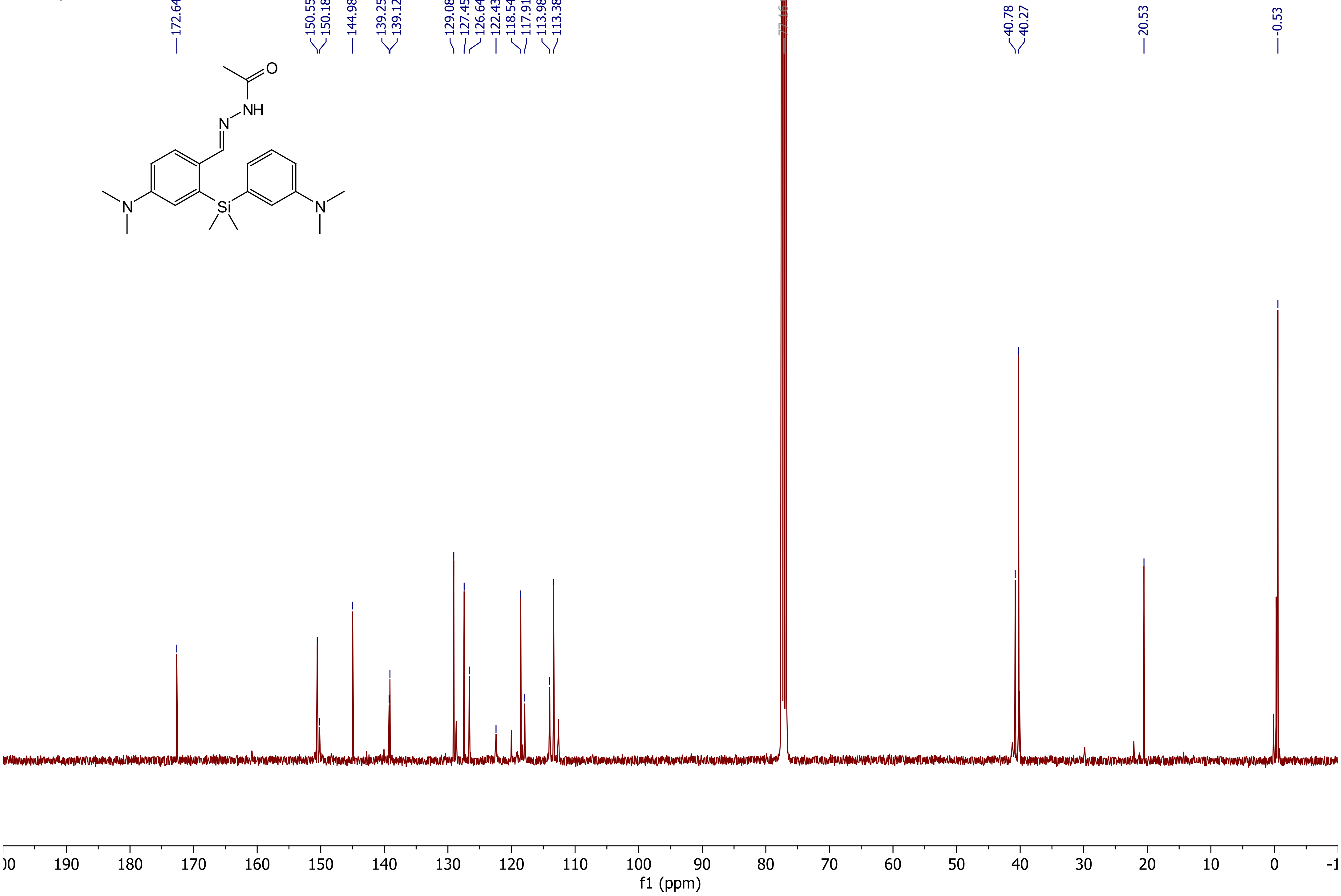

172.64
150.55
150.18
144.98
139.25
139.12
129.08
127.45
126.64
122.43
118.54
117.91
113.98
113.38
77.16 CDCl3
40.78
40.27
20.53
-0.53
O
NH
N
N
Si
N
f1 (ppm)
190 180 170 160 150 140 130 120 110 100 90 80 70 60 50 40 30 20 10 0

Compound 19

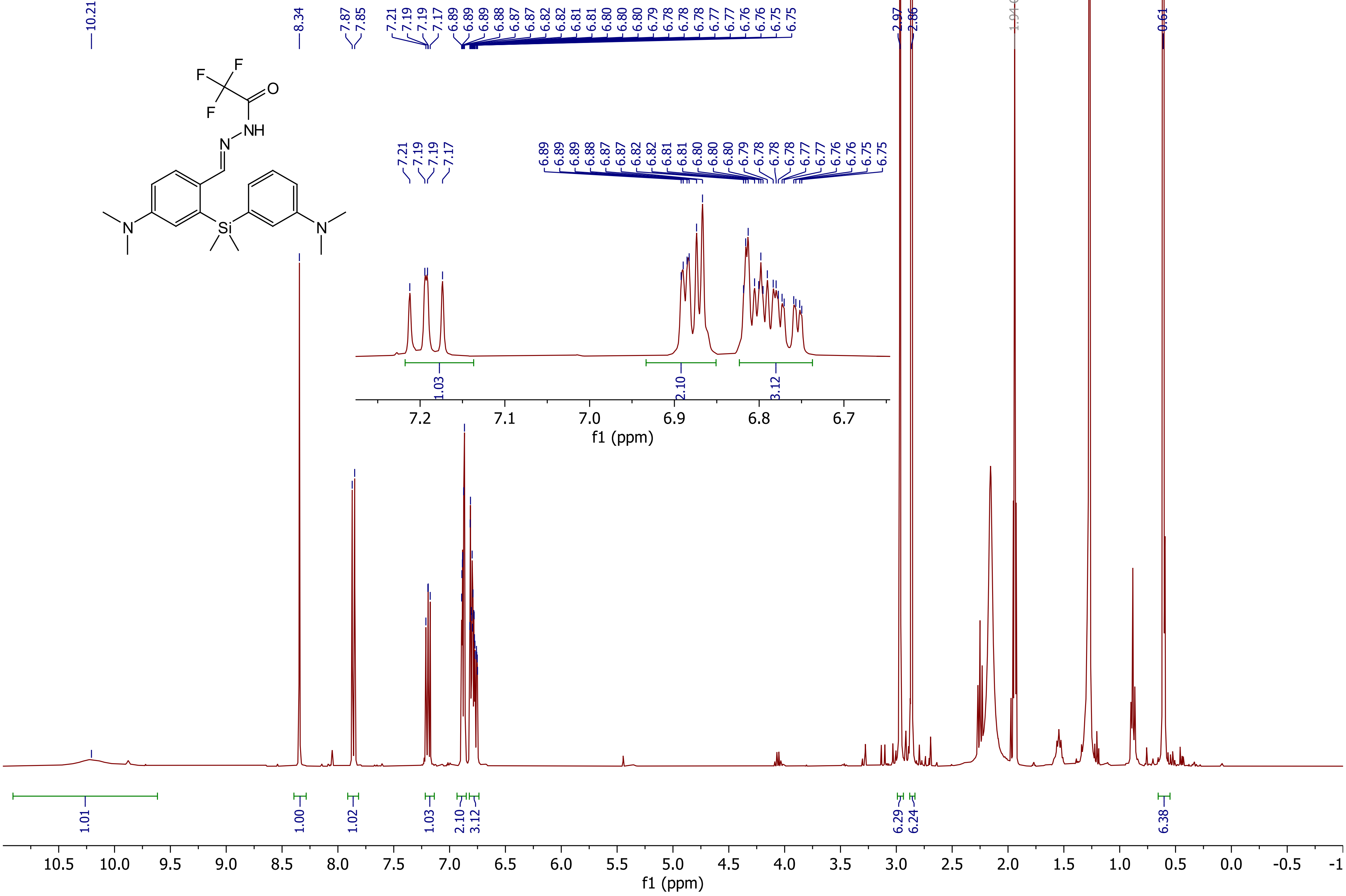

10.21
8.34
7.87
7.85
7.21
7.19
7.19
7.17
6.89
6.89
6.88
6.87
6.87
6.82
6.82
6.81
6.81
6.80
6.80
6.79
6.78
6.78
6.78
6.77
6.77
6.76
6.76
6.75
6.75
2.97
2.86
1.94 CD3CN
0.61
F
F
F
O
NH
N
N
Si
N
1.03
2.10
3.12
7.2
7.1
7.0
6.9
6.8
6.7
f1 (ppm)
1.01
1.00
1.02
1.03
2.10
3.12
6.29
6.24
6.38
10.5
10.0
9.5
9.0
8.5
8.0
7.5
7.0
6.5
6.0
5.5
5.0
4.5
4.0
3.5
3.0
2.5
2.0
1.5
1.0
0.5
0.0
-0.5
-1
f1 (ppm)

Compound 19

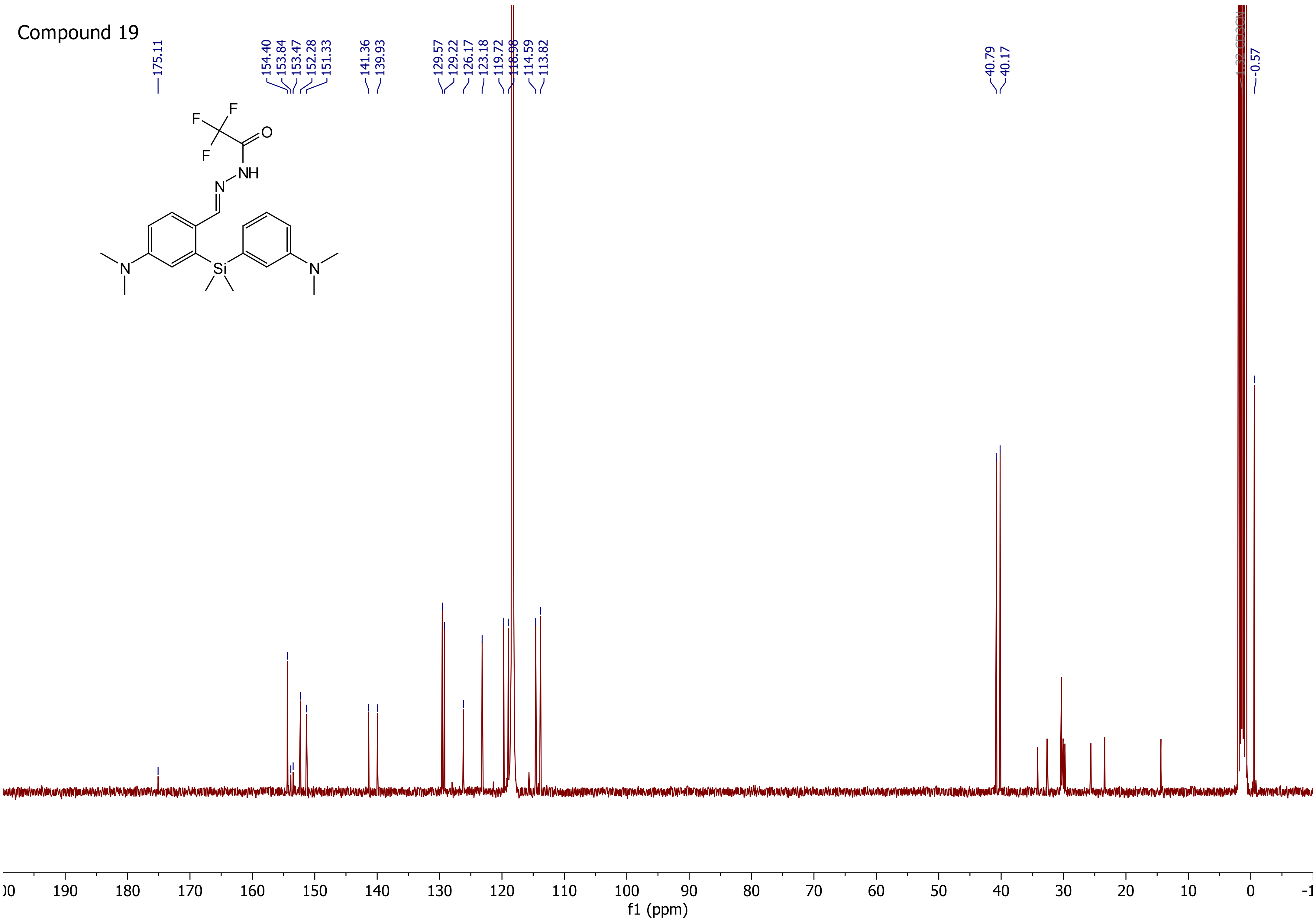

175.11
154.40
153.84
153.47
152.28
151.33
141.36
139.93
129.57
129.22
126.17
123.18
119.72
118.98
114.59
113.82
40.79
40.17
1.32 CD3CN
-0.57
F
F
F
O
NH
N
N
N
Si
f1 (ppm)

Compound 20

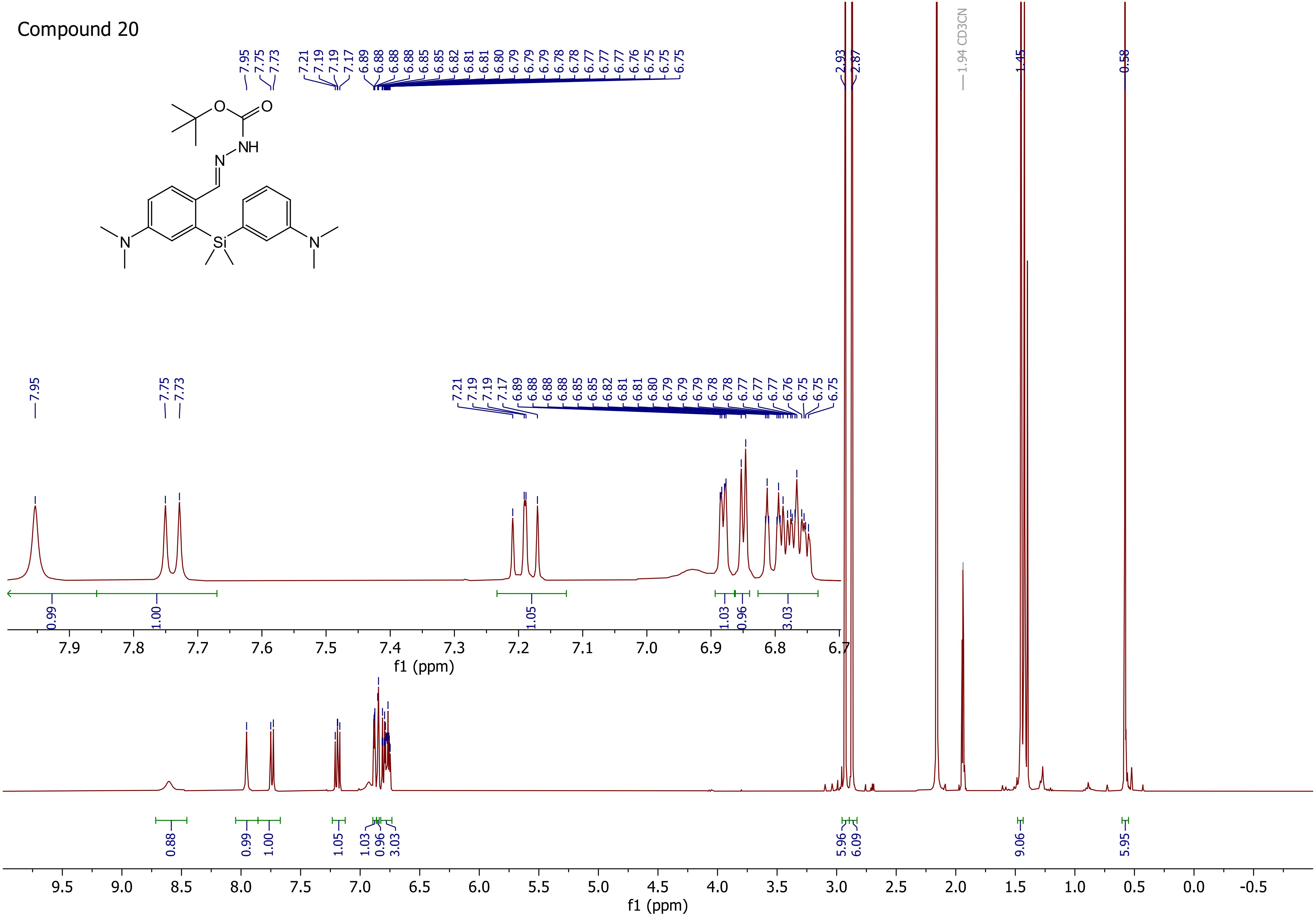

7.95
7.75
7.73
7.21
7.19
7.19
7.17
6.89
6.88
6.88
6.88
6.85
6.85
6.82
6.81
6.81
6.80
6.79
6.79
6.79
6.78
6.78
6.77
6.77
6.77
6.76
6.75
6.75
6.75
2.93
2.87
1.94 CD3CN
1.45
0.58
O
O
NH
N
N
Si
N
0.99
1.00
1.05
1.03
0.96
3.03
f1 (ppm)
0.88
5.96
6.09
9.06
5.95

Compound 20

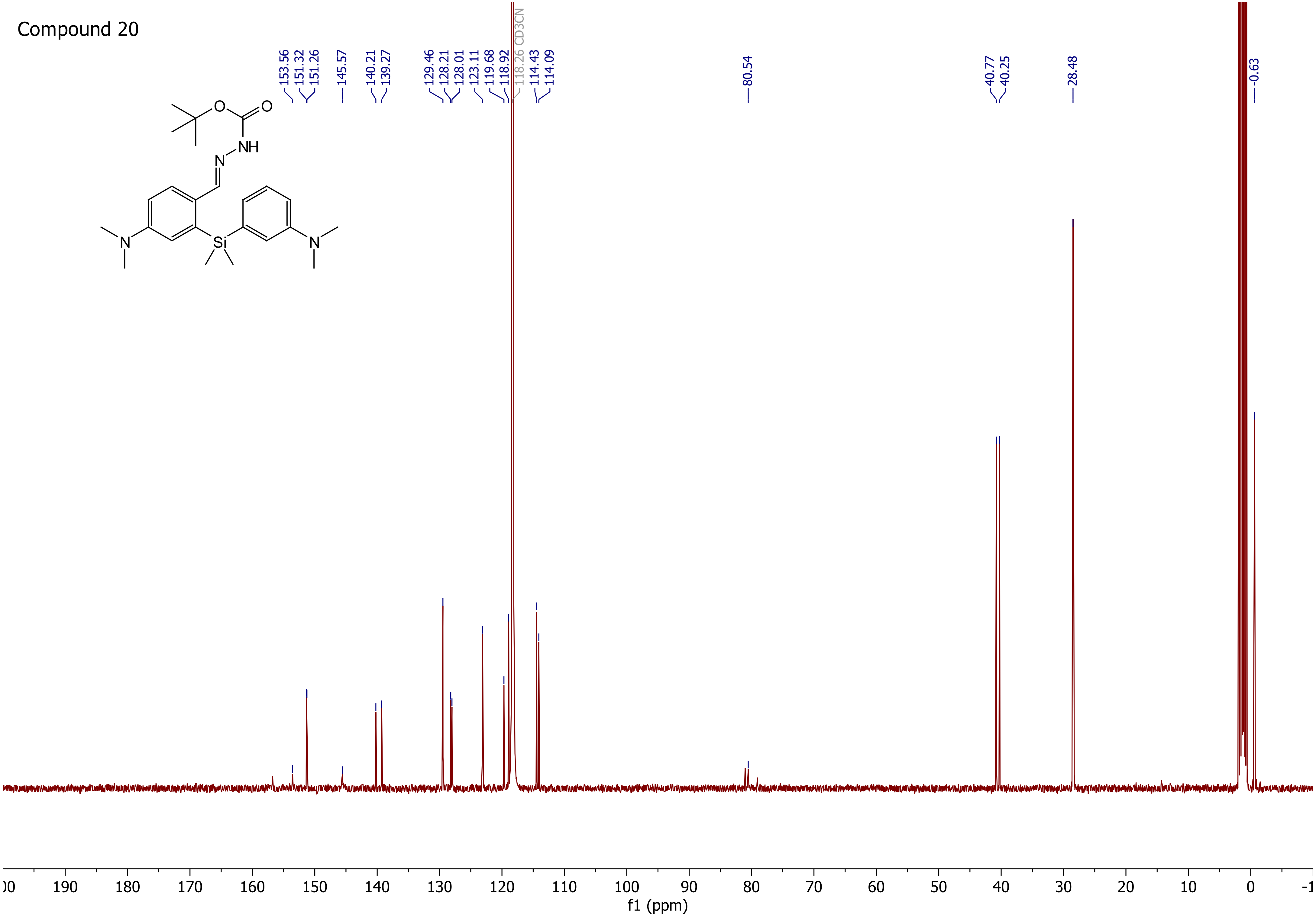

153.56
151.32
151.26
145.57
140.21
139.27
129.46
128.21
128.01
123.11
119.68
118.92
118.26 CD3CN
114.43
114.09
80.54
40.77
40.25
28.48
-0.63
190
180
170
160
150
140
130
120
110
100
90
80
70
60
50
40
30
20
10
0
f1 (ppm)

Compound 21

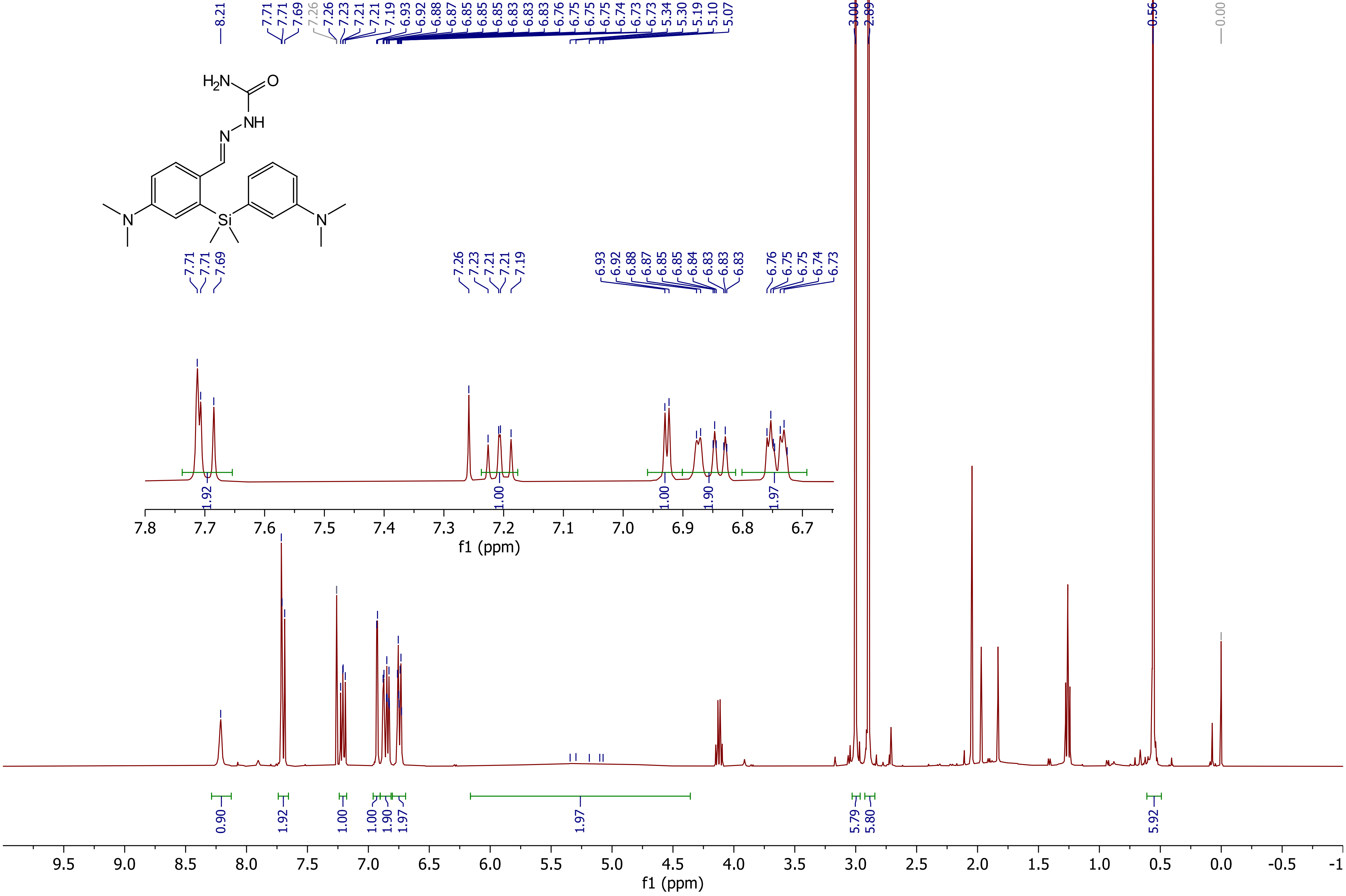
8.21
7.71 7.71 7.69
7.26 CDCl3
7.26 7.23 7.21 7.21 7.19 6.93 6.92 6.88 6.87 6.85 6.85 6.85 6.83 6.83 6.83 6.76 6.75 6.75 6.75 6.74 6.73 6.73 5.34 5.30 5.19 5.10 5.07
3.00 2.89
0.56
0.00
H2N O NH N Si N N
7.71 7.71 7.69
7.26 7.23 7.21 7.21 7.19
6.93 6.92 6.88 6.87 6.85 6.85 6.84 6.83 6.83 6.83
6.76 6.75 6.75 6.74 6.73
1.92 1.00 1.00 1.90 1.97
7.8 7.7 7.6 7.5 7.4 7.3 7.2 7.1 7.0 6.9 6.8 6.7
f1 (ppm)
0.90 1.92 1.00 1.00 1.90 1.97 1.97 5.79 5.80 5.92
9.5 9.0 8.5 8.0 7.5 7.0 6.5 6.0 5.5 5.0 4.5 4.0 3.5 3.0 2.5 2.0 1.5 1.0 0.5 0.0 -0.5 -1
f1 (ppm)

Compound 21

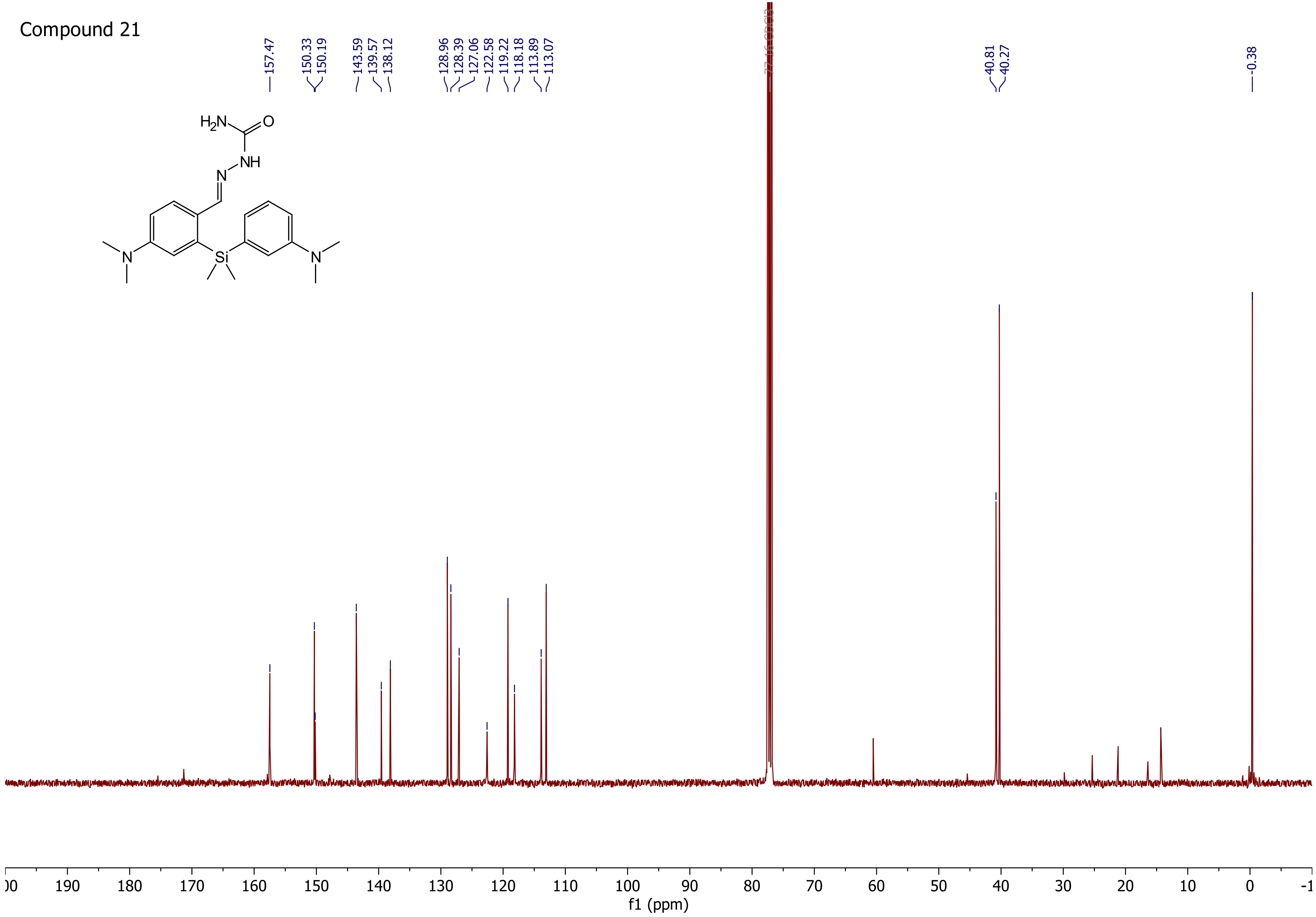

157.47
150.33
150.19
143.59
139.57
138.12
128.96
128.39
127.06
122.58
119.22
118.18
113.89
113.07
77.16 CDCl3
40.81
40.27
-0.38
H2N
O
NH
N
N
Si
N
f1 (ppm)

Compound 22

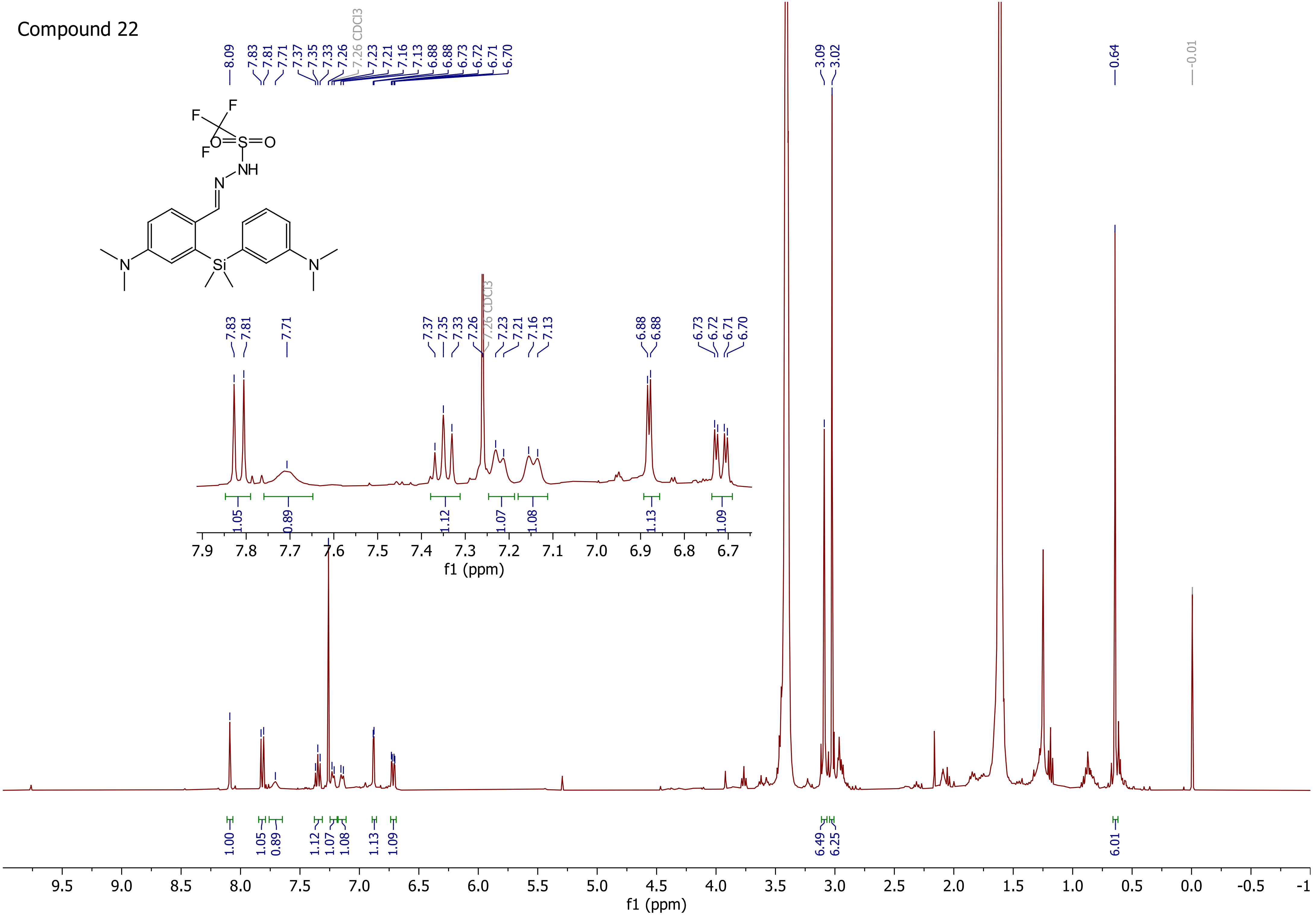

8.09
7.83
7.81
7.71
7.37
7.35
7.33
7.26
7.26 CDCl3
7.23
7.21
7.16
7.13
6.88
6.88
6.73
6.72
6.71
6.70
3.09
3.02
0.64
-0.01
1.00
1.05
0.89
1.12
1.07
1.08
1.13
1.09
6.49
6.25
6.01
F
O
S
NH
N
Si
f1 (ppm)

Compound 22

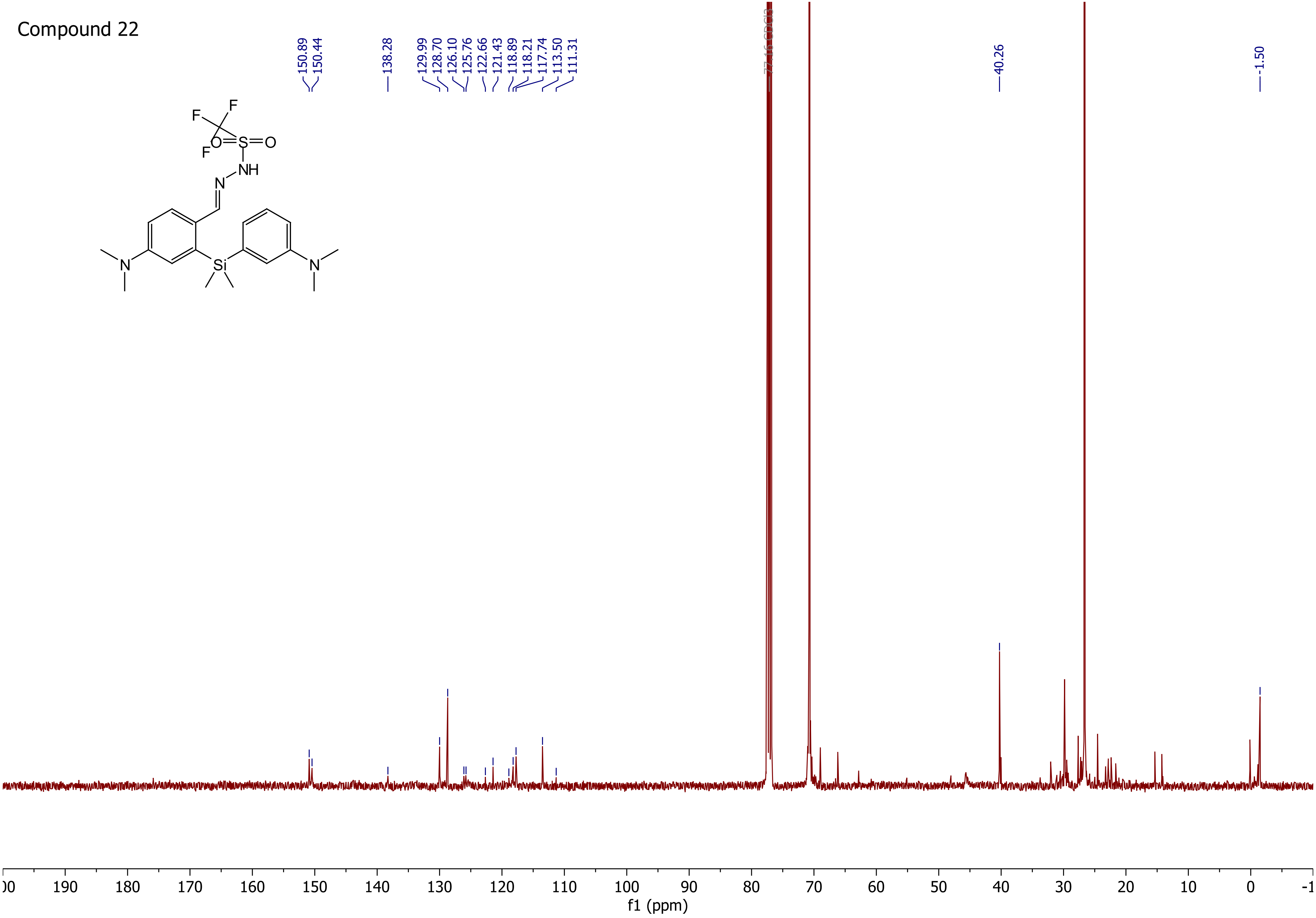
F
F
F
O
S
O
NH
N
N
Si
N
150.89
150.44
138.28
129.99
128.70
126.10
125.76
122.66
121.43
118.89
118.21
117.74
113.50
111.31
77.16 CDCl3
40.26
-1.50
190
180
170
160
150
140
130
120
110
100
90
80
70
60
50
40
30
20
10
0
f1 (ppm)

Compound 23

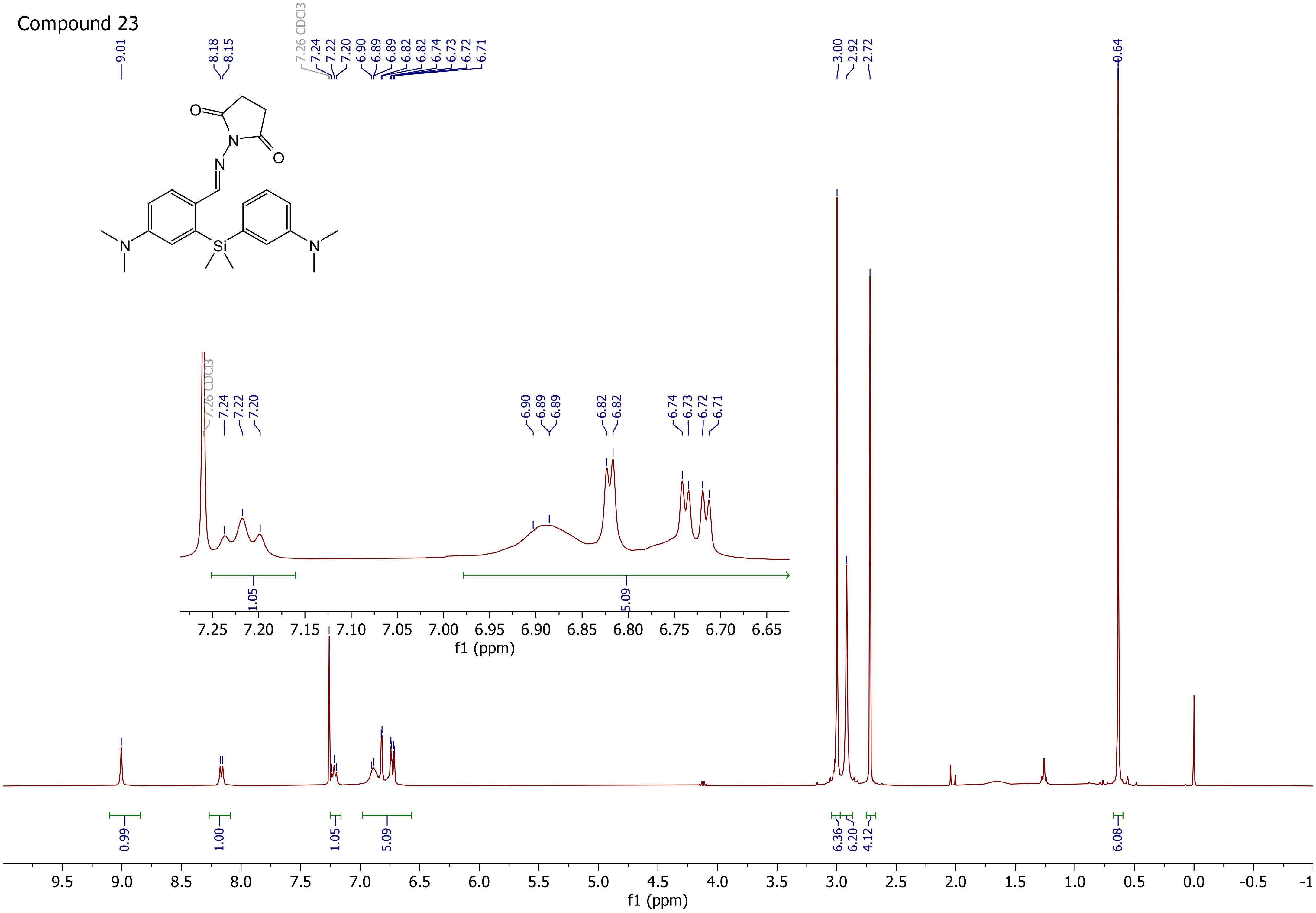

9.01
8.18
8.15
7.26 CDCl3
7.24
7.22
7.20
6.90
6.89
6.89
6.82
6.82
6.74
6.73
6.72
6.71
3.00
2.92
2.72
0.64
O
N
N
O
N
Si
N
1.05
5.09
f1 (ppm)
0.99
1.00
1.05
5.09
6.36
6.20
4.12
6.08
f1 (ppm)

Compound 23

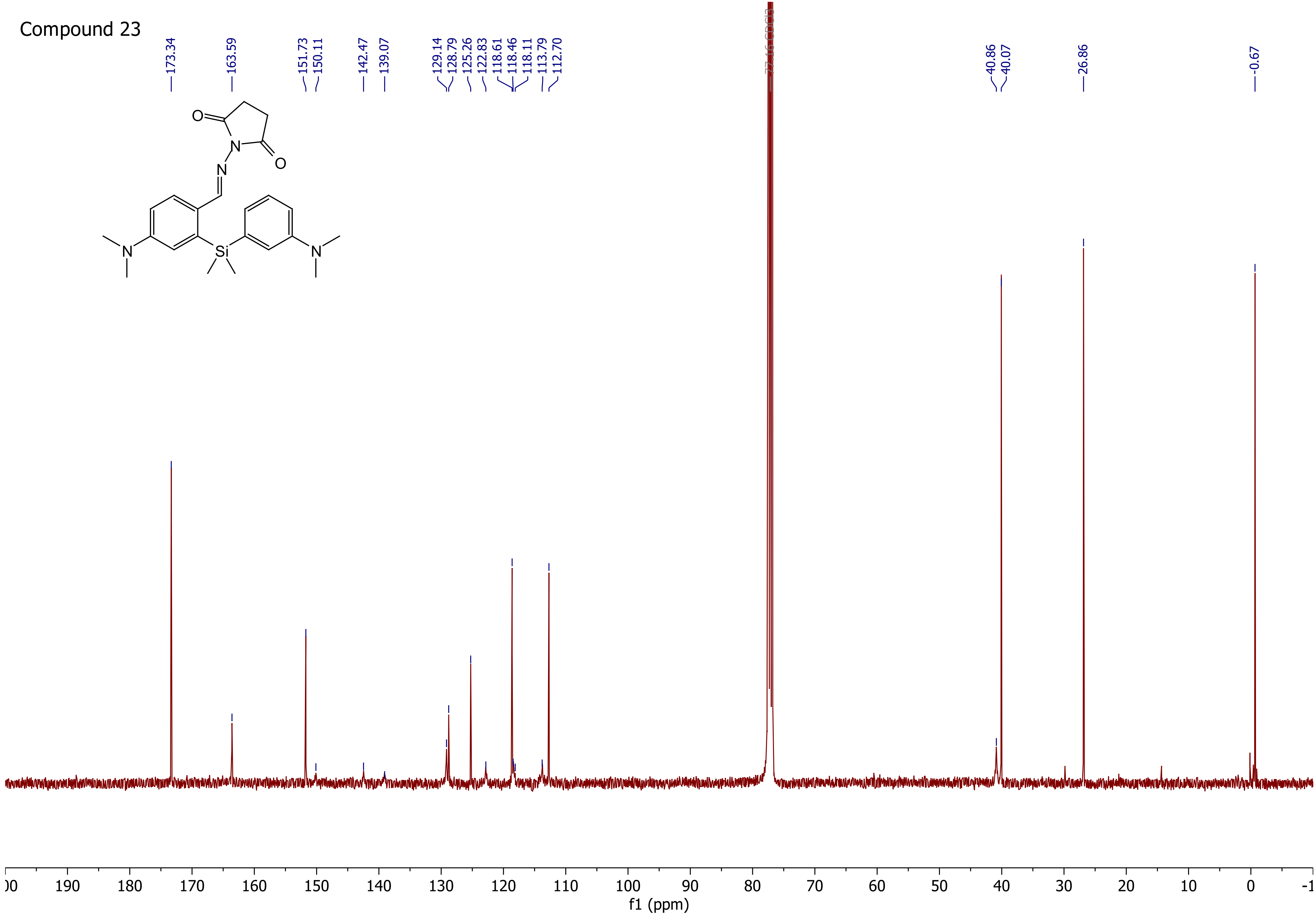

173.34
163.59
151.73
150.11
142.47
139.07
129.14
128.79
125.26
122.83
118.61
118.46
118.11
113.79
112.70
77.16 CDCl3
40.86
40.07
26.86
-0.67
O
N
N
O
N
Si
N
f1 (ppm)

Compound 24

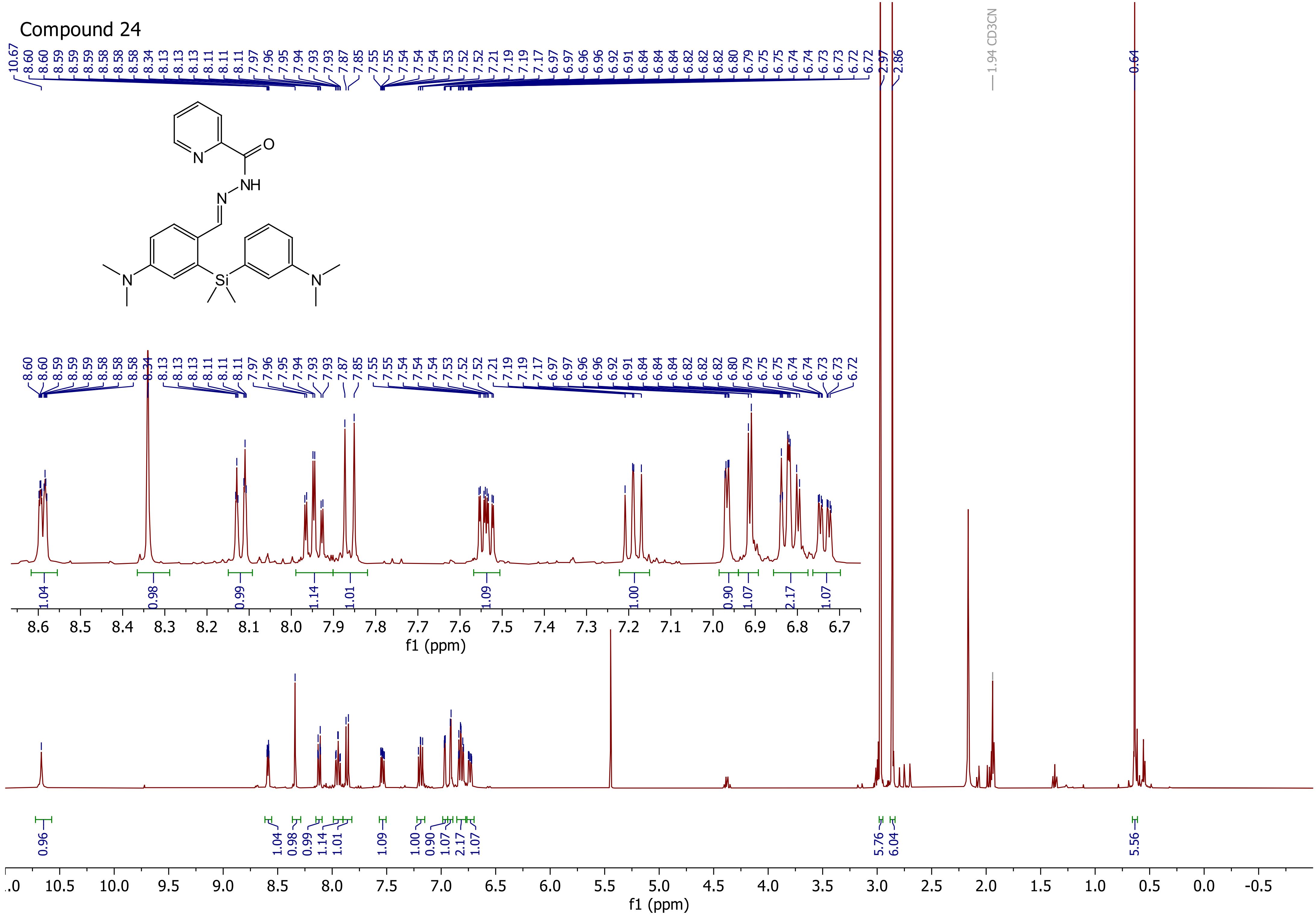

Compound 24

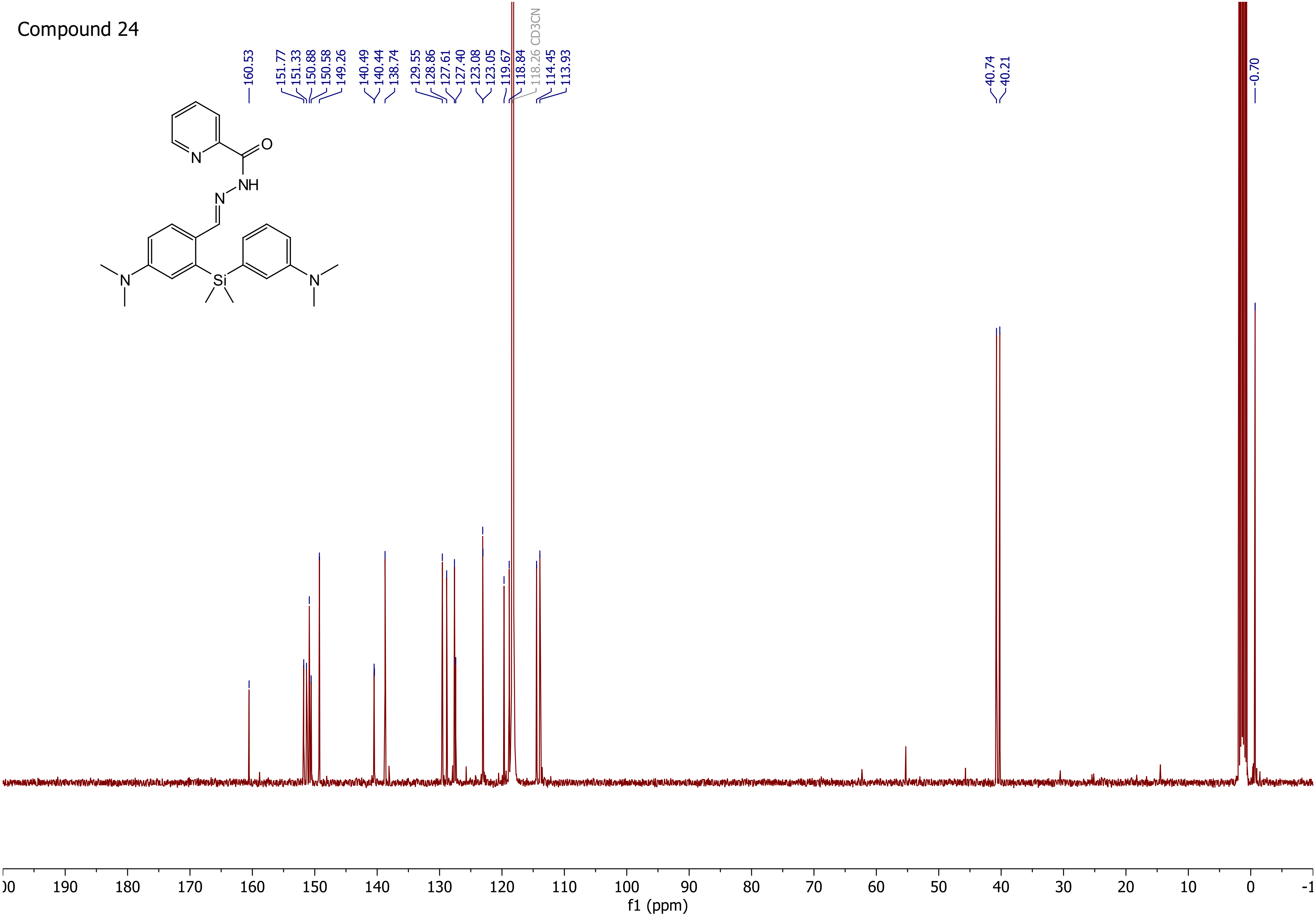

160.53
151.77
151.33
150.88
150.58
149.26
140.49
140.44
138.74
129.55
128.86
127.61
127.40
123.08
123.05
119.67
118.84
118.26 CD3CN
114.45
113.93
40.74
40.21
-0.70
O
NH
N
N
Si
N
N
190 180 170 160 150 140 130 120 110 100 90 80 70 60 50 40 30 20 10 0 -1
f1 (ppm)

Compound 25

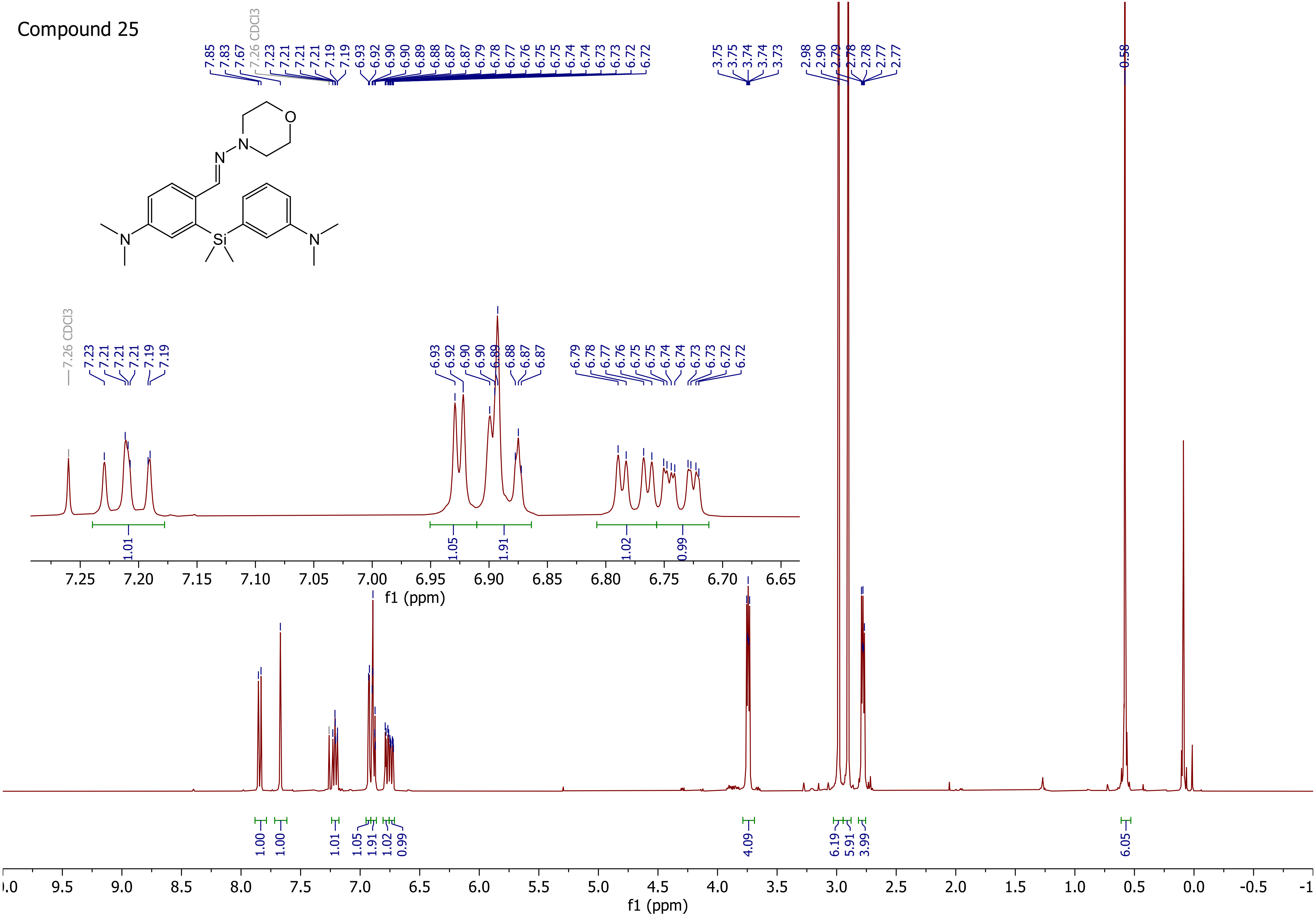
7.85
7.83
7.67
7.26 CDCl3
7.23
7.21
7.21
7.21
7.19
7.19
6.93
6.92
6.90
6.90
6.89
6.88
6.87
6.87
6.79
6.78
6.77
6.76
6.75
6.75
6.74
6.74
6.73
6.73
6.72
6.72
3.75
3.75
3.74
3.74
3.73
2.98
2.90
2.79
2.78
2.78
2.77
2.77
0.58
O
N
N
N
Si
N
1.01
1.05
1.91
1.02
0.99
f1 (ppm)
1.00
1.00
1.01
1.05
1.91
1.02
0.99
4.09
6.19
5.91
3.99
6.05
f1 (ppm)

Compound 25

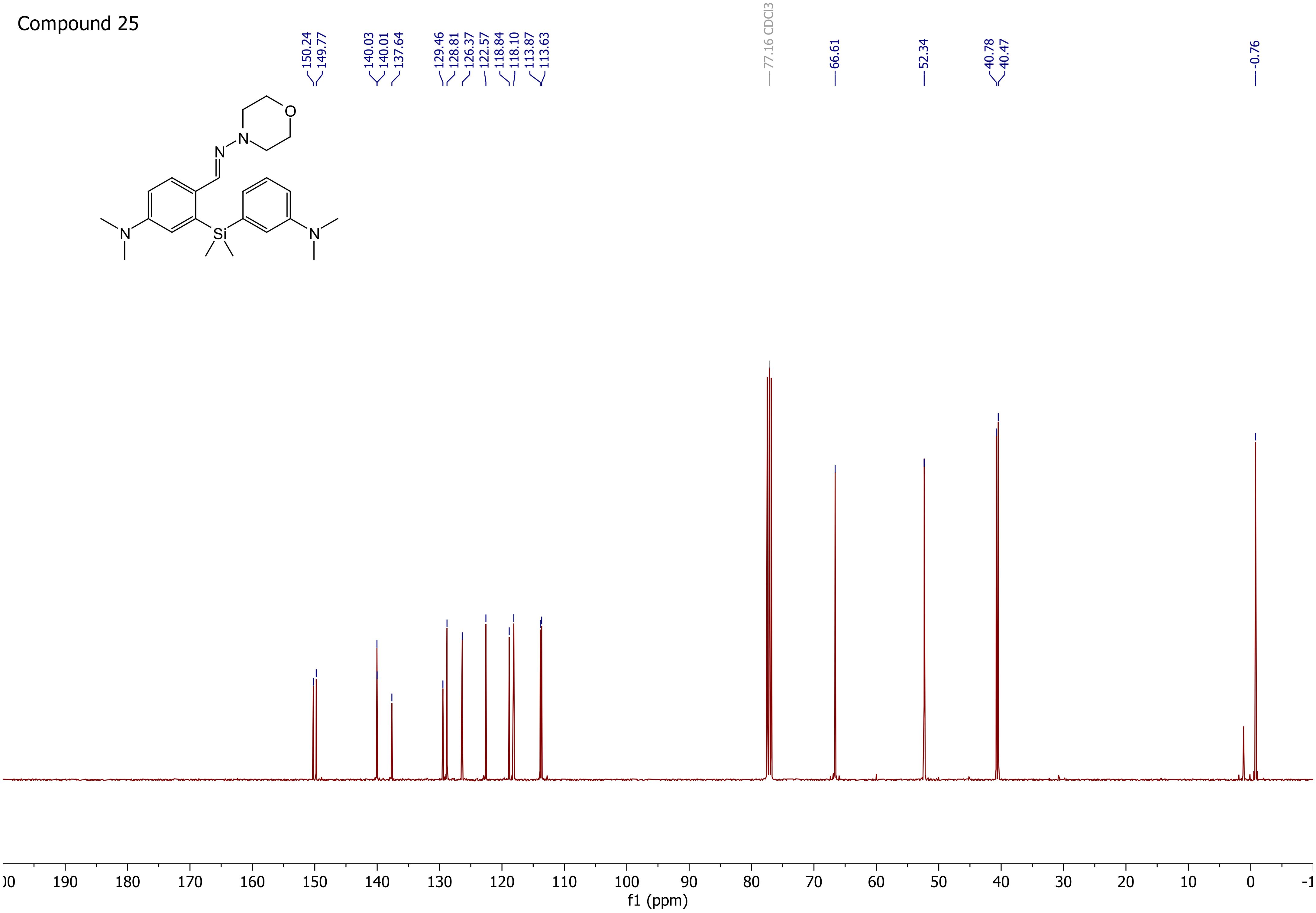
150.24
149.77
140.03
140.01
137.64
129.46
128.81
126.37
122.57
118.84
118.10
113.87
113.63
77.16 CDCl3
66.61
52.34
40.78
40.47
-0.76
f1 (ppm)

Compound 26

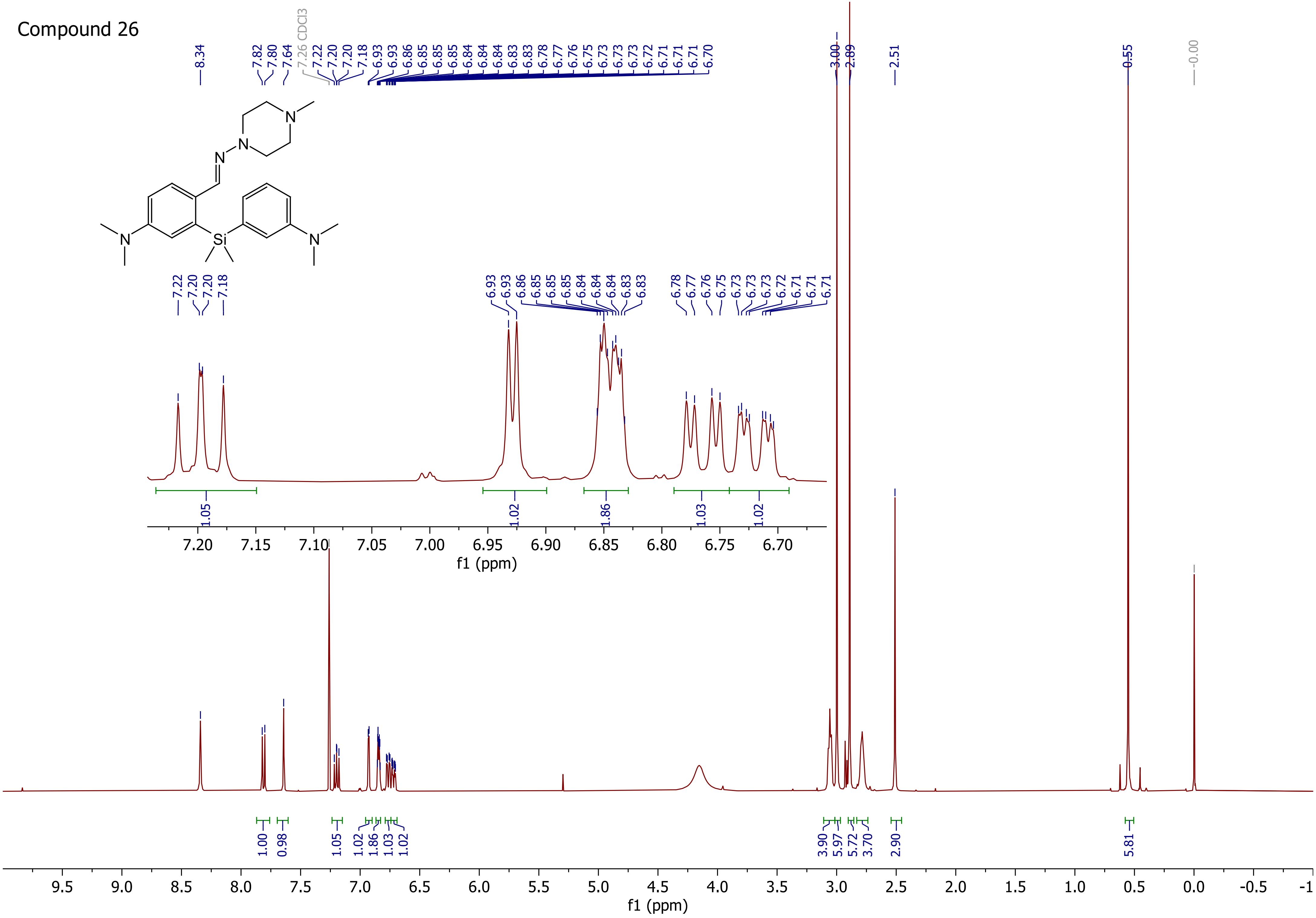

Compound 26

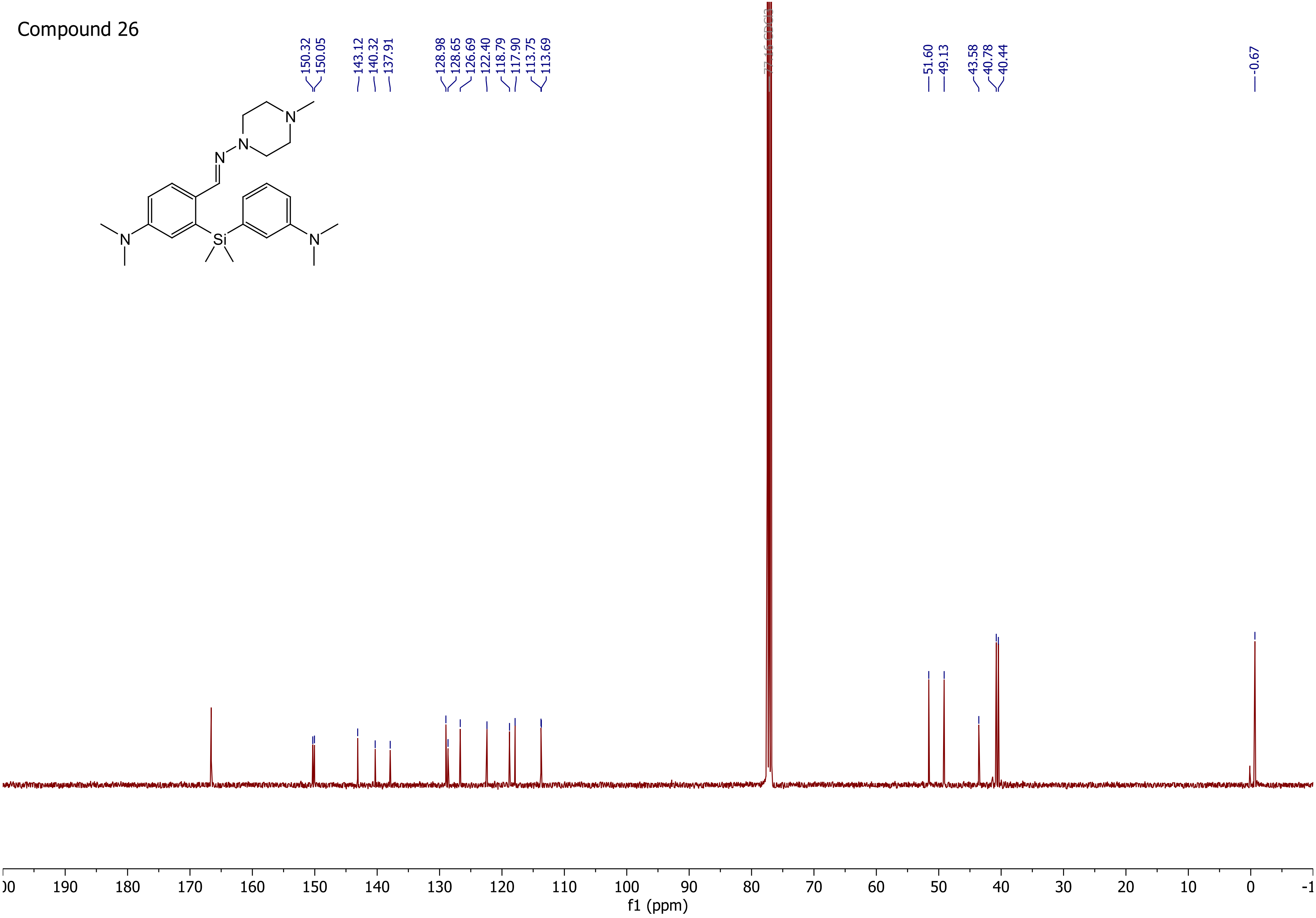
150.32
150.05
143.12
140.32
137.91
128.98
128.65
126.69
122.40
118.79
117.90
113.75
113.69
77.16 CDCl3
51.60
49.13
43.58
40.78
40.44
-0.67
N
N
N
N
N
Si
200
190
180
170
160
150
140
130
120
110
100
90
80
70
60
50
40
30
20
10
0
-1
f1 (ppm)

Compound 27

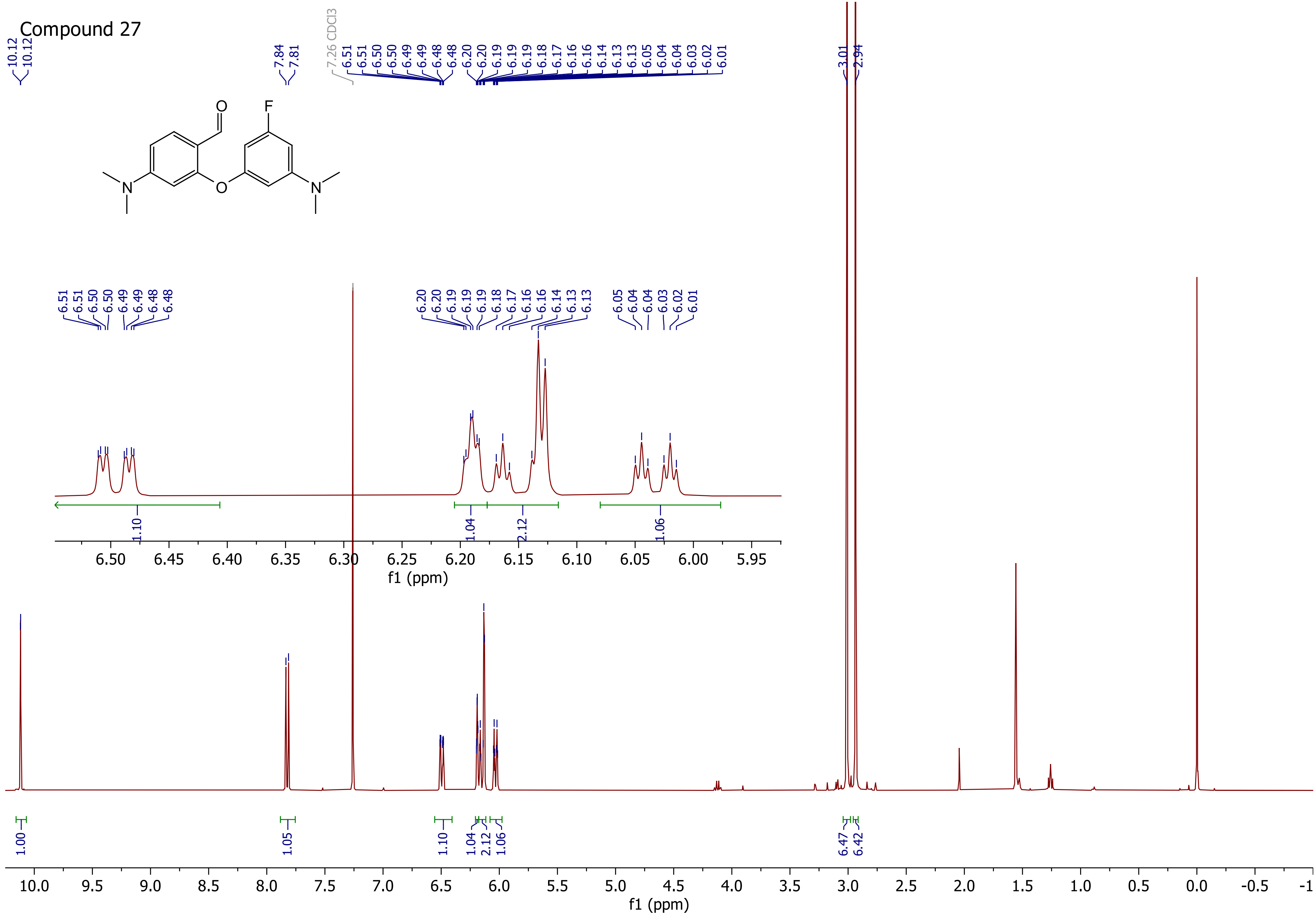
O
F
N
O
N
10.12
10.12
7.84
7.81
7.26 CDCl3
6.51
6.51
6.50
6.50
6.49
6.49
6.48
6.48
6.20
6.20
6.19
6.19
6.19
6.18
6.17
6.16
6.16
6.14
6.13
6.13
6.05
6.04
6.04
6.03
6.02
6.01
3.01
2.94
1.10
1.04
2.12
1.06
f1 (ppm)
1.00
1.05
6.47
6.42
f1 (ppm)

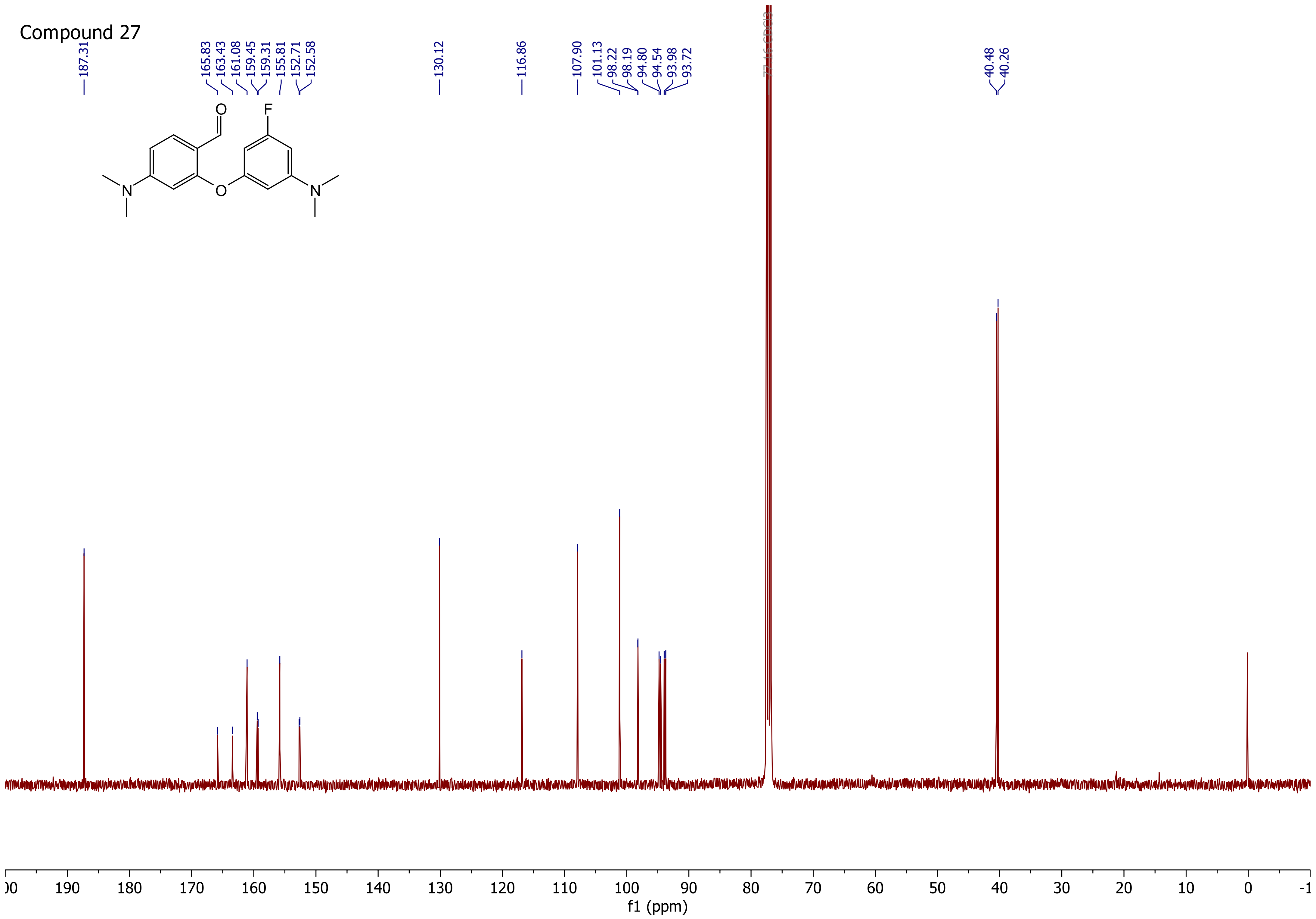

Compound 27
187.31
165.83
163.43
161.08
159.45
159.31
155.81
152.71
152.58
130.12
116.86
107.90
101.13
98.22
98.19
94.80
94.54
93.98
93.72
77.16 CDCl3
40.48
40.26
O
F
N
O
N
f1 (ppm)

Compound 28

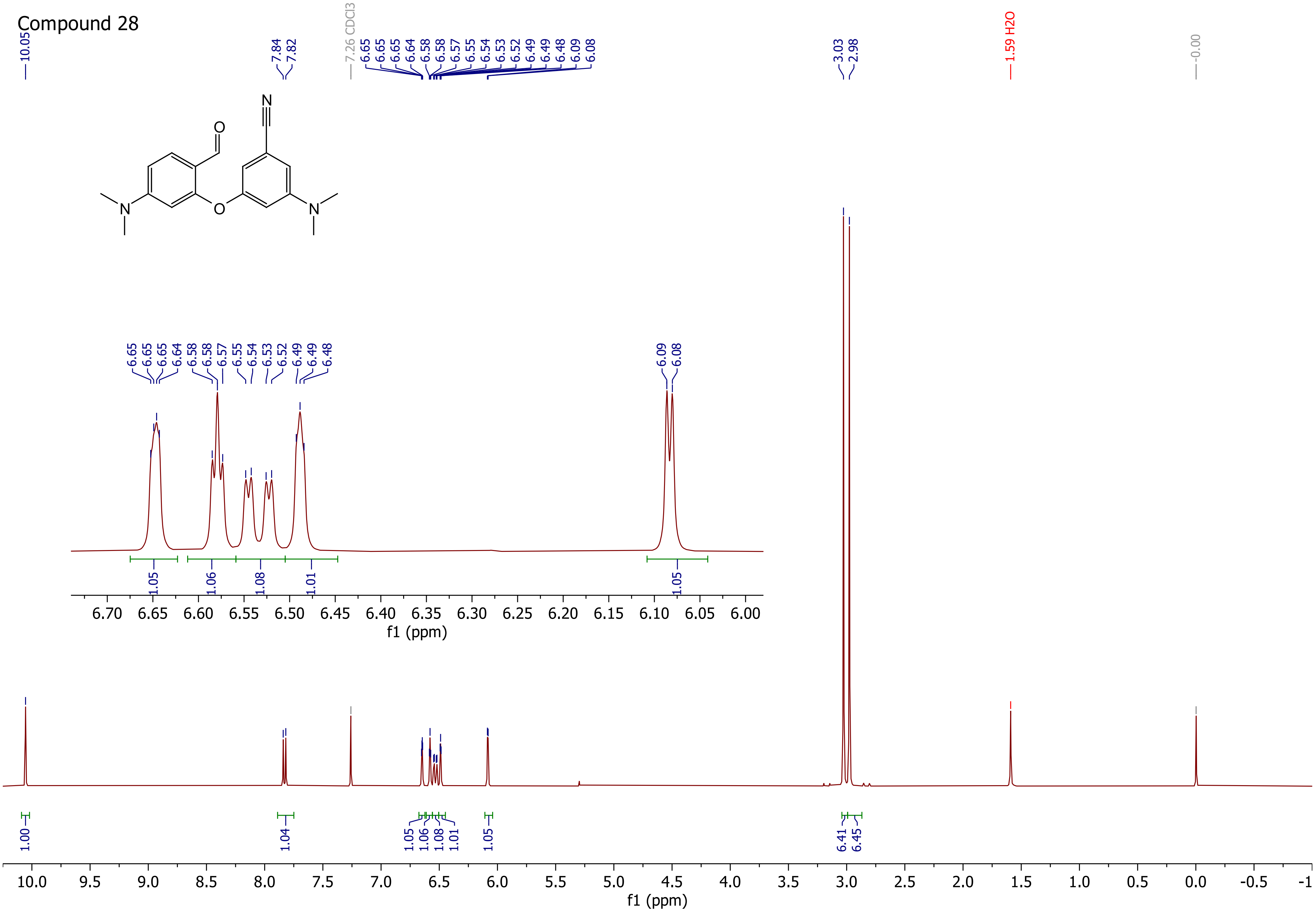

10.05
7.84
7.82
7.26 CDCl3
6.65
6.65
6.65
6.64
6.58
6.58
6.57
6.55
6.54
6.53
6.52
6.49
6.49
6.48
6.09
6.08
3.03
2.98
1.59 H2O
-0.00
O
N
N
N
O
1.05
1.06
1.08
1.01
1.05
f1 (ppm)
1.00
1.04
1.05
1.06
1.08
1.01
1.05
6.41
6.45
f1 (ppm)

Compound 28

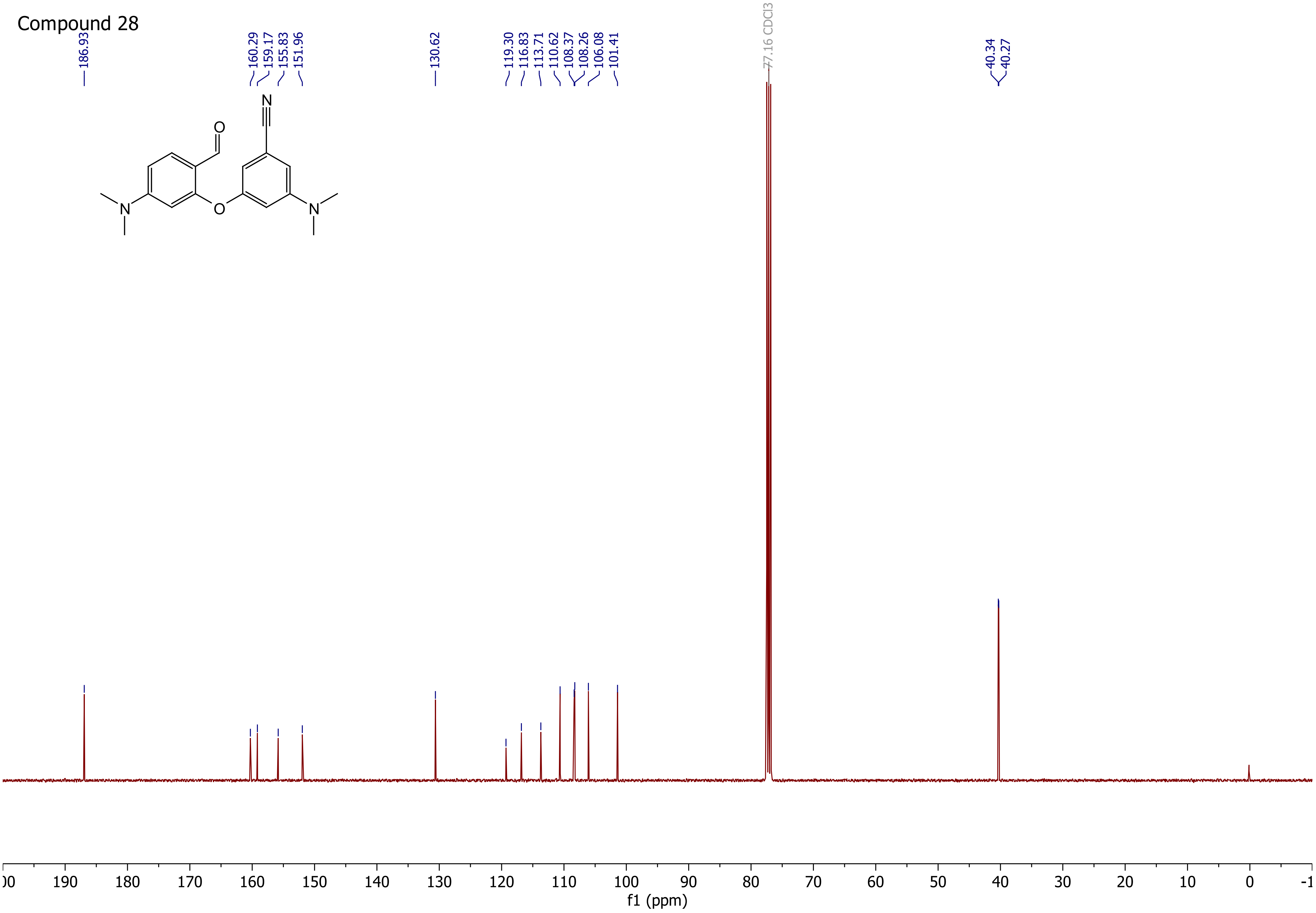

186.93
160.29
159.17
155.83
151.96
130.62
119.30
116.83
113.71
110.62
108.37
108.26
106.08
101.41
77.16 CDCl3
40.34
40.27
O
N
N
O
N
190
180
170
160
150
140
130
120
110
100
90
80
70
60
50
40
30
20
10
0
f1 (ppm)

Compound 29

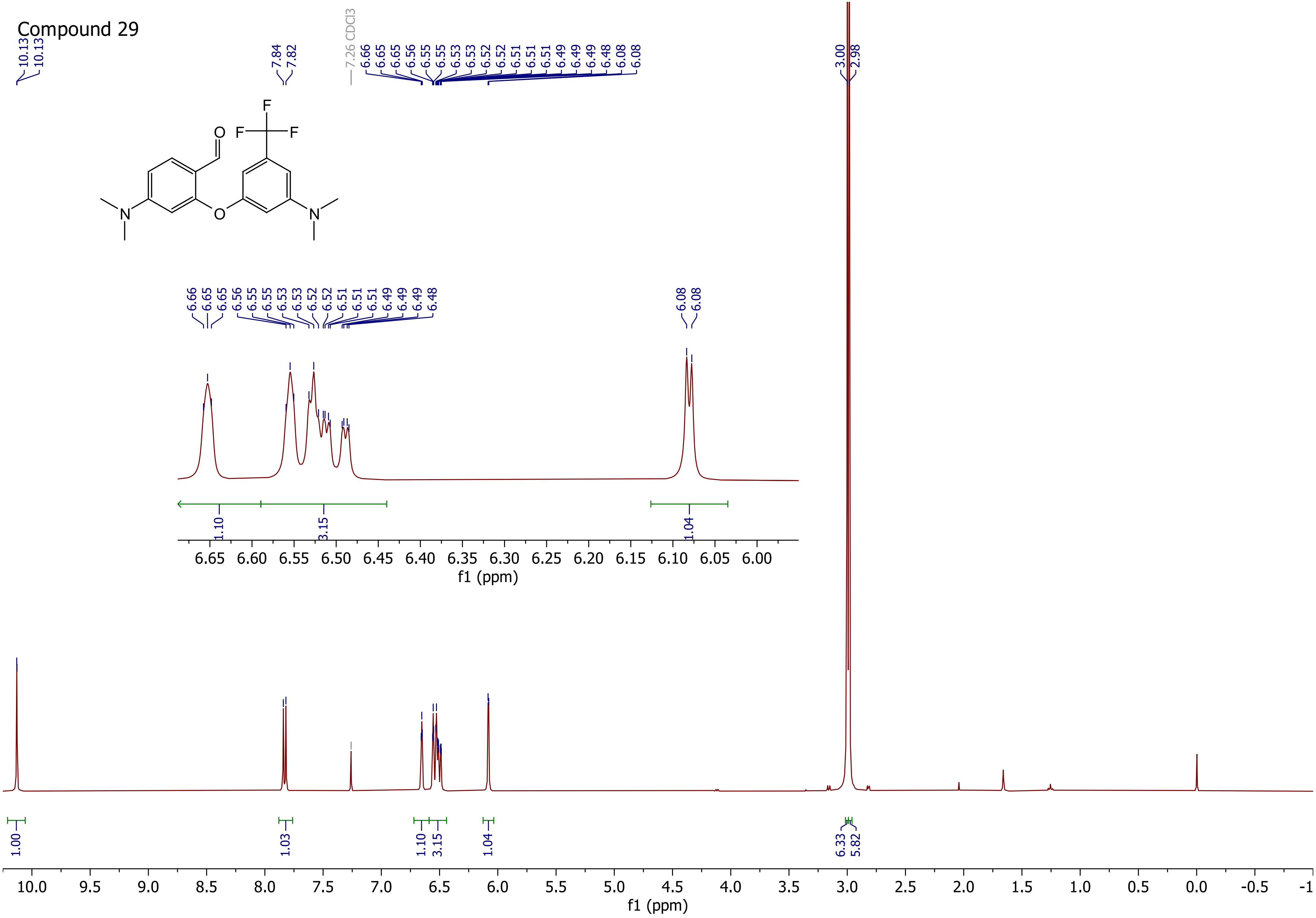

10.13
10.13
7.84
7.82
7.26 CDCl3
6.66
6.65
6.65
6.56
6.55
6.55
6.53
6.53
6.52
6.52
6.51
6.51
6.51
6.49
6.49
6.49
6.48
6.08
6.08
3.00
2.98
F
O
F
F
N
O
N
1.10
3.15
1.04
f1 (ppm)
1.00
1.03
6.33
5.82

Compound 29

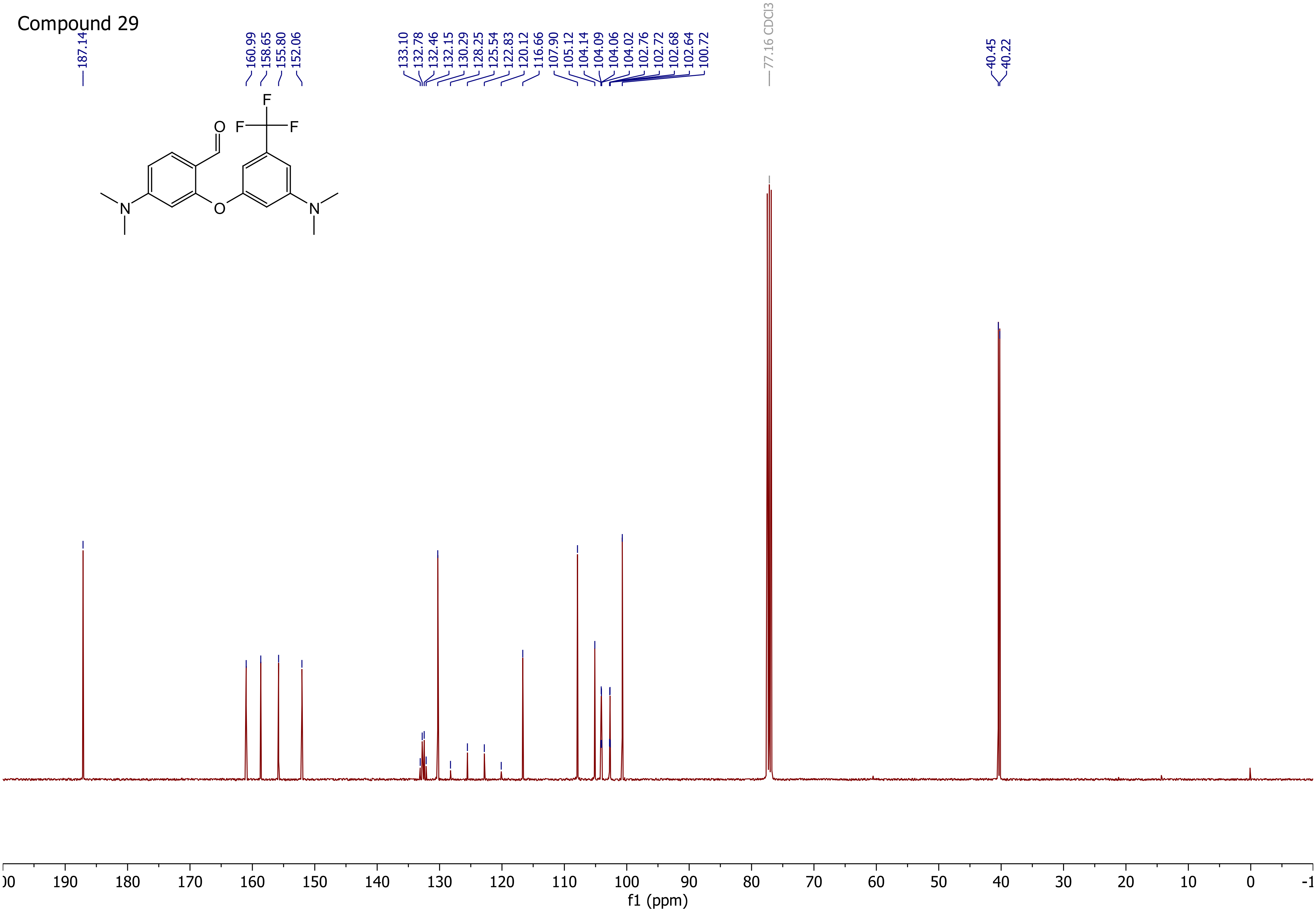
187.14
160.99
158.65
155.80
152.06
133.10
132.78
132.46
132.15
130.29
128.25
125.54
122.83
120.12
116.66
107.90
105.12
104.14
104.09
104.06
104.02
102.76
102.72
102.68
102.64
100.72
77.16 CDCl3
40.45
40.22
O
F
F
F
N
O
N
f1 (ppm)

Compound 31

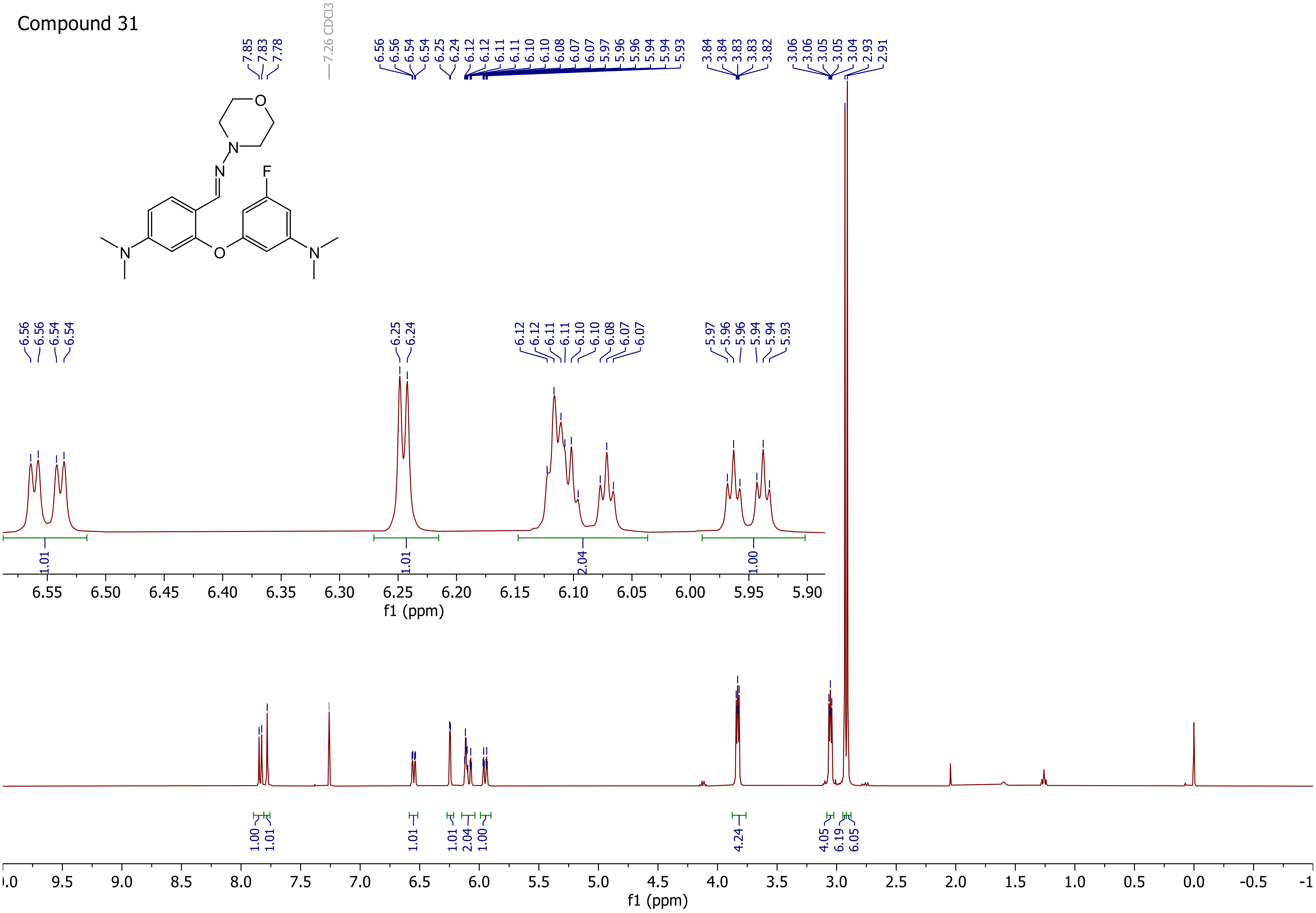
7.85
7.83
7.78
7.26 CDCl3
6.56
6.56
6.54
6.54
6.25
6.24
6.12
6.12
6.11
6.11
6.10
6.10
6.08
6.07
6.07
5.97
5.96
5.96
5.94
5.94
5.93
3.84
3.84
3.83
3.83
3.82
3.06
3.06
3.05
3.05
3.04
2.93
2.91
O
N
N
F
N
O
N
1.01
1.01
2.04
1.00
6.55
6.50
6.45
6.40
6.35
6.30
6.25
6.20
6.15
6.10
6.05
6.00
5.95
5.90
f1 (ppm)
1.00
1.01
1.01
1.01
2.04
1.00
4.24
4.05
6.19
6.05
9.5
9.0
8.5
8.0
7.5
7.0
6.5
6.0
5.5
5.0
4.5
4.0
3.5
3.0
2.5
2.0
1.5
1.0
0.5
0.0
-0.5
f1 (ppm)

Compound 31

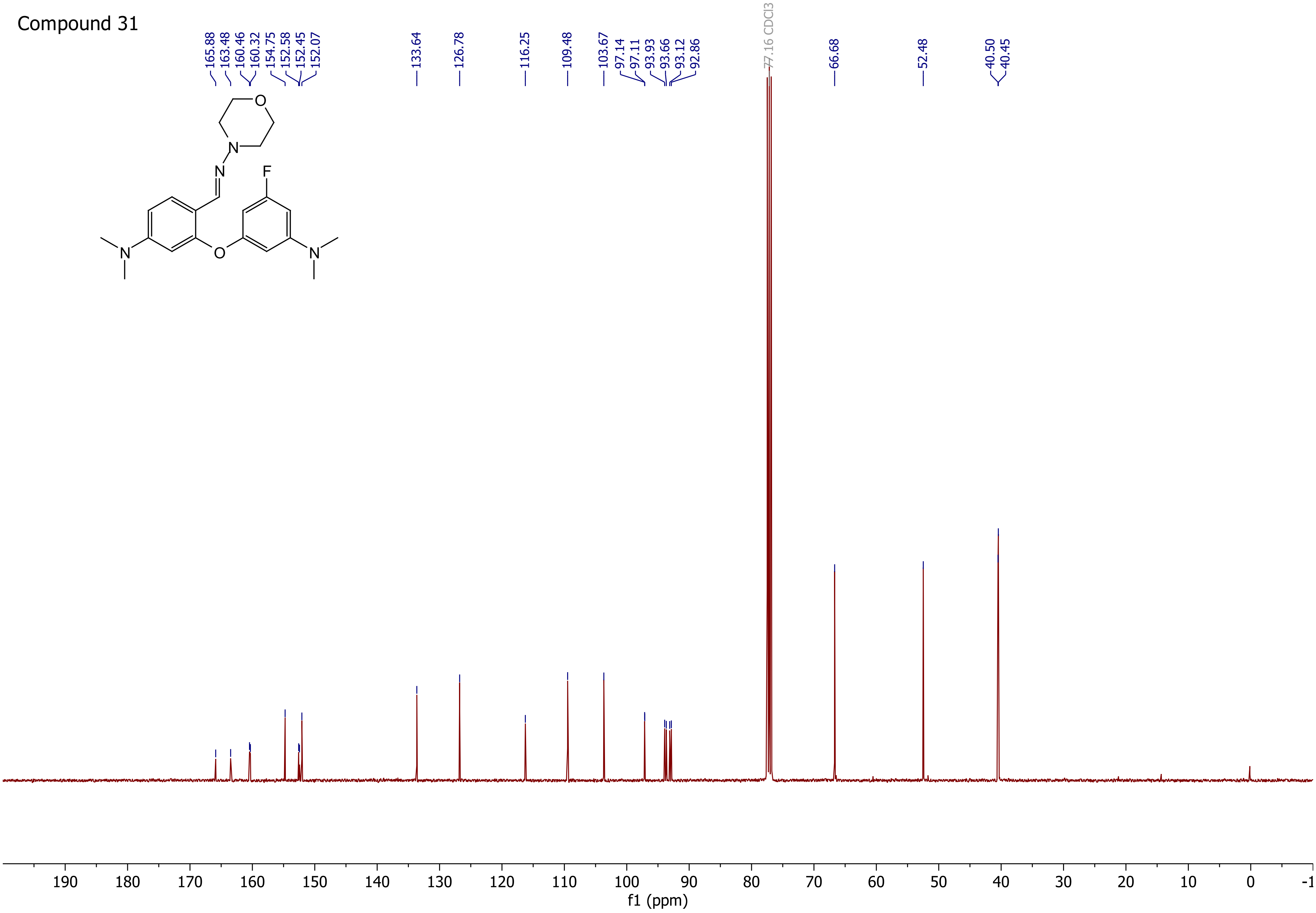

165.88
163.48
160.46
160.32
154.75
152.58
152.45
152.07
133.64
126.78
116.25
109.48
103.67
97.14
97.11
93.93
93.66
93.12
92.86
77.16 CDCl3
66.68
52.48
40.50
40.45
F
O
N
190 180 170 160 150 140 130 120 110 100 90 80 70 60 50 40 30 20 10 0 -1
f1 (ppm)

Compound 32

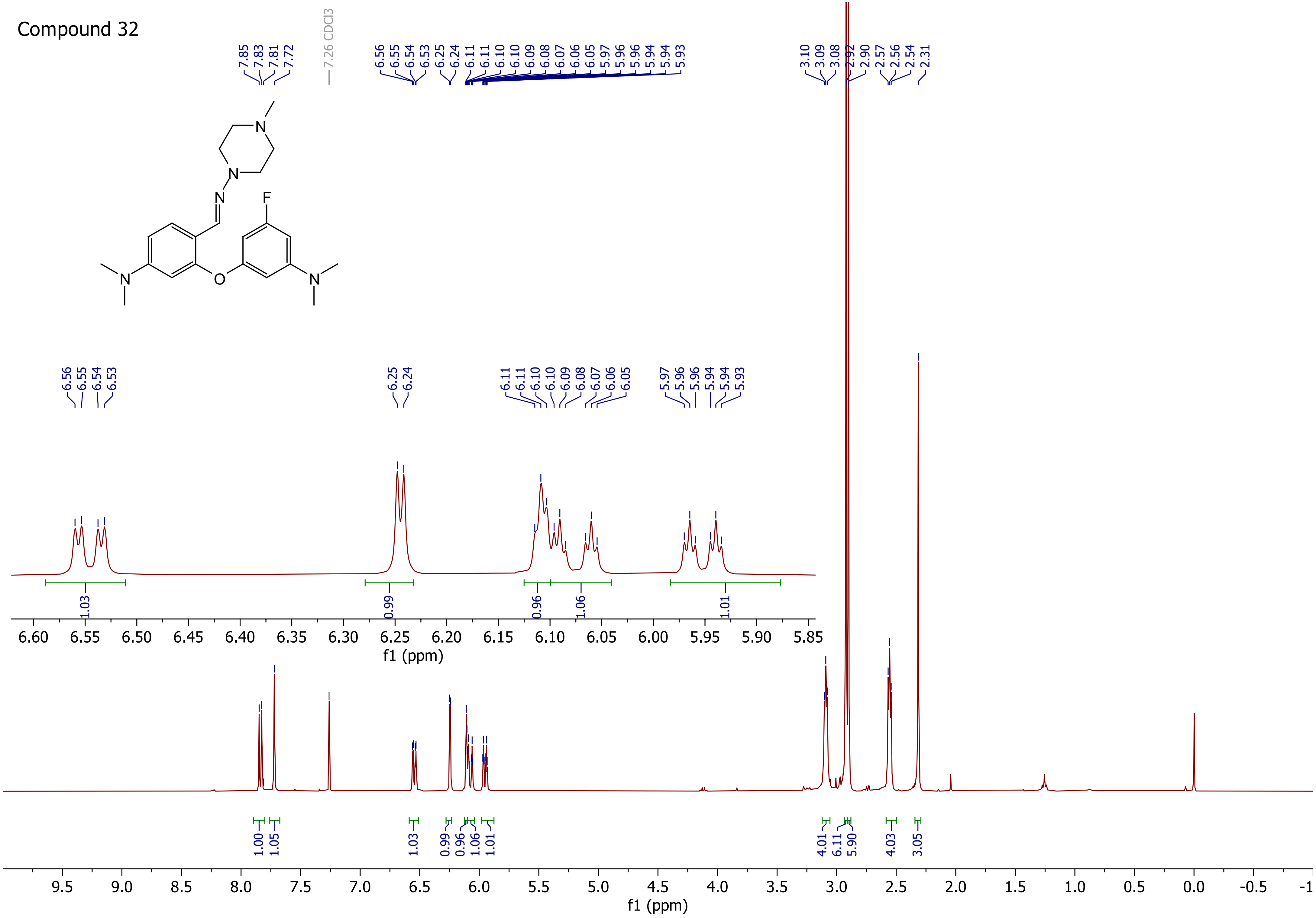

7.85
7.83
7.81
7.72
7.26 CDCl3
6.56
6.55
6.54
6.53
6.25
6.24
6.11
6.11
6.10
6.10
6.09
6.08
6.07
6.06
6.05
5.97
5.96
5.96
5.94
5.94
5.93
3.10
3.09
3.08
2.92
2.90
2.57
2.56
2.54
2.31
N
N
N
F
N
O
N
1.03
0.99
0.96
1.06
1.01
f1 (ppm)
1.00
1.05
1.03
0.99
0.96
1.06
1.01
4.01
6.11
5.90
4.03
3.05
f1 (ppm)

Compound 32

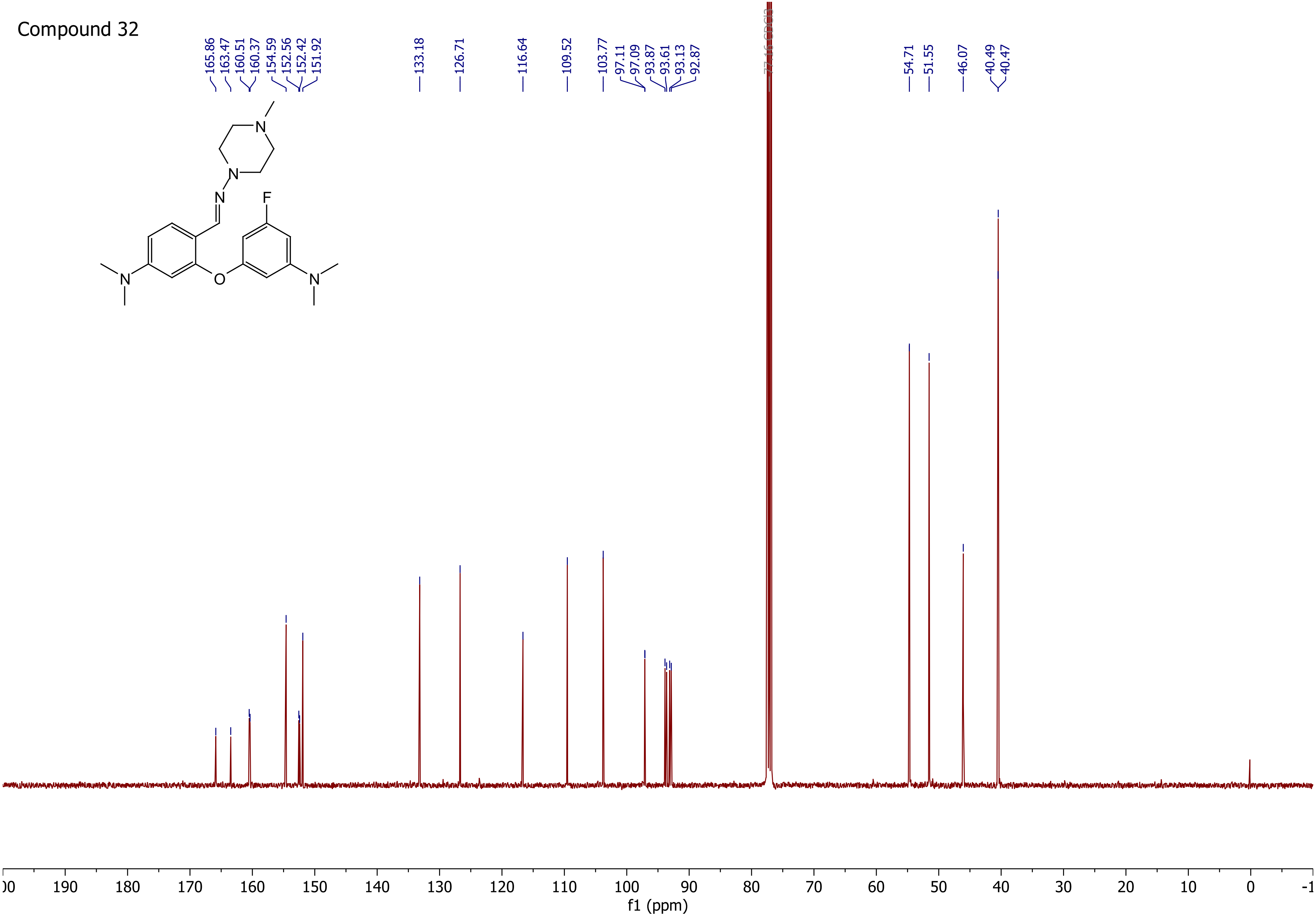

165.86
163.47
160.51
160.37
154.59
152.56
152.42
151.92
133.18
126.71
116.64
109.52
103.77
97.11
97.09
93.87
93.61
93.13
92.87
77.16 CDCl3
54.71
51.55
46.07
40.49
40.47
F
N
O
)0 190 180 170 160 150 140 130 120 110 100 90 80 70 60 50 40 30 20 10 0 -1
f1 (ppm)

Compound 33

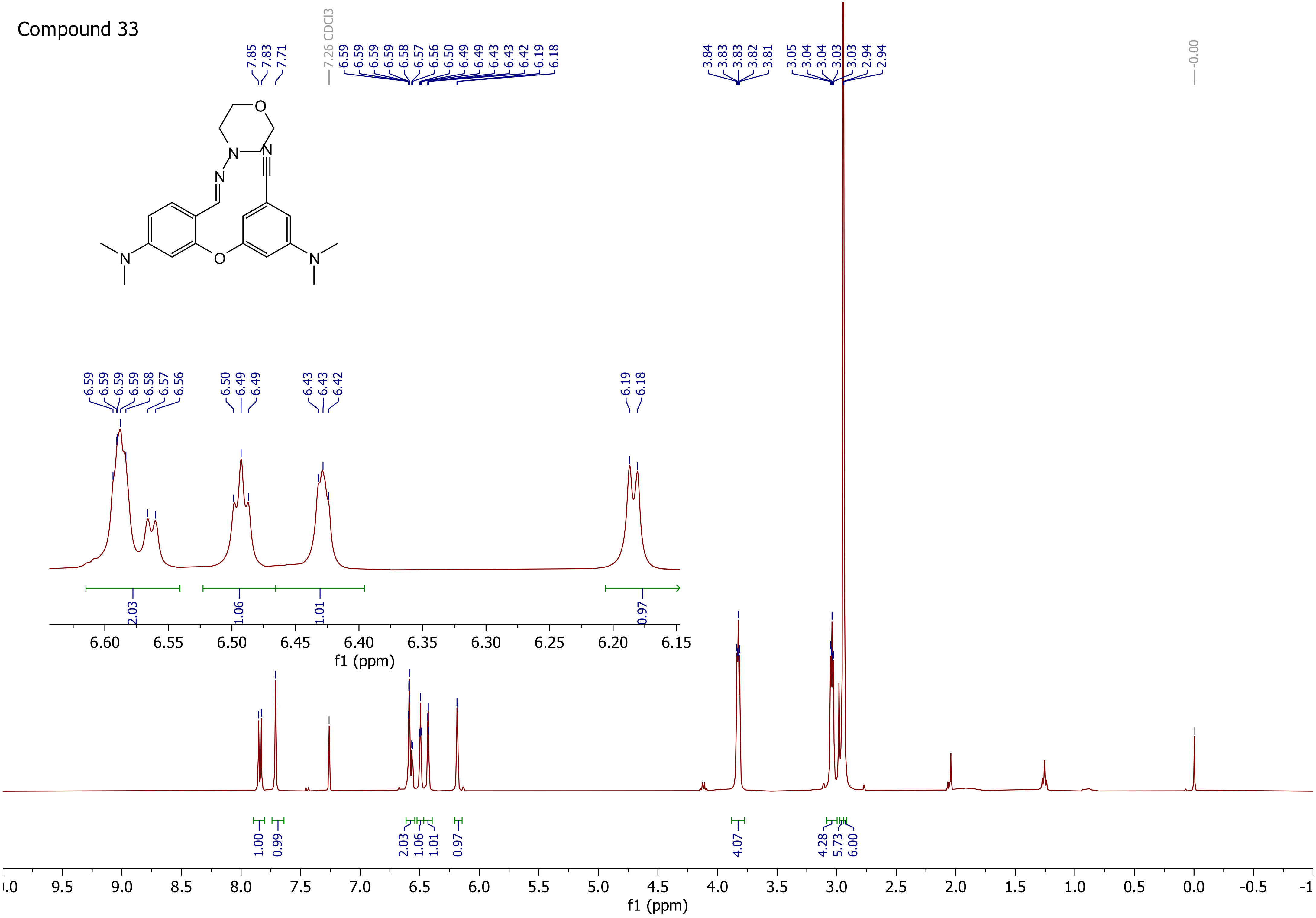
7.85
7.83
7.71
7.26 CDCl3
6.59
6.59
6.59
6.59
6.58
6.57
6.56
6.50
6.49
6.49
6.43
6.43
6.42
6.19
6.18
3.84
3.83
3.83
3.82
3.81
3.05
3.04
3.04
3.03
3.03
2.94
2.94
-0.00
2.03
1.06
1.01
0.97
6.60
6.55
6.50
6.45
6.40
6.35
6.30
6.25
6.20
6.15
f1 (ppm)
1.00
0.99
2.03
1.06
1.01
0.97
4.07
4.28
5.73
6.00
9.5
9.0
8.5
8.0
7.5
7.0
6.5
6.0
5.5
5.0
4.5
4.0
3.5
3.0
2.5
2.0
1.5
1.0
0.5
0.0
-0.5
f1 (ppm)

Compound 33

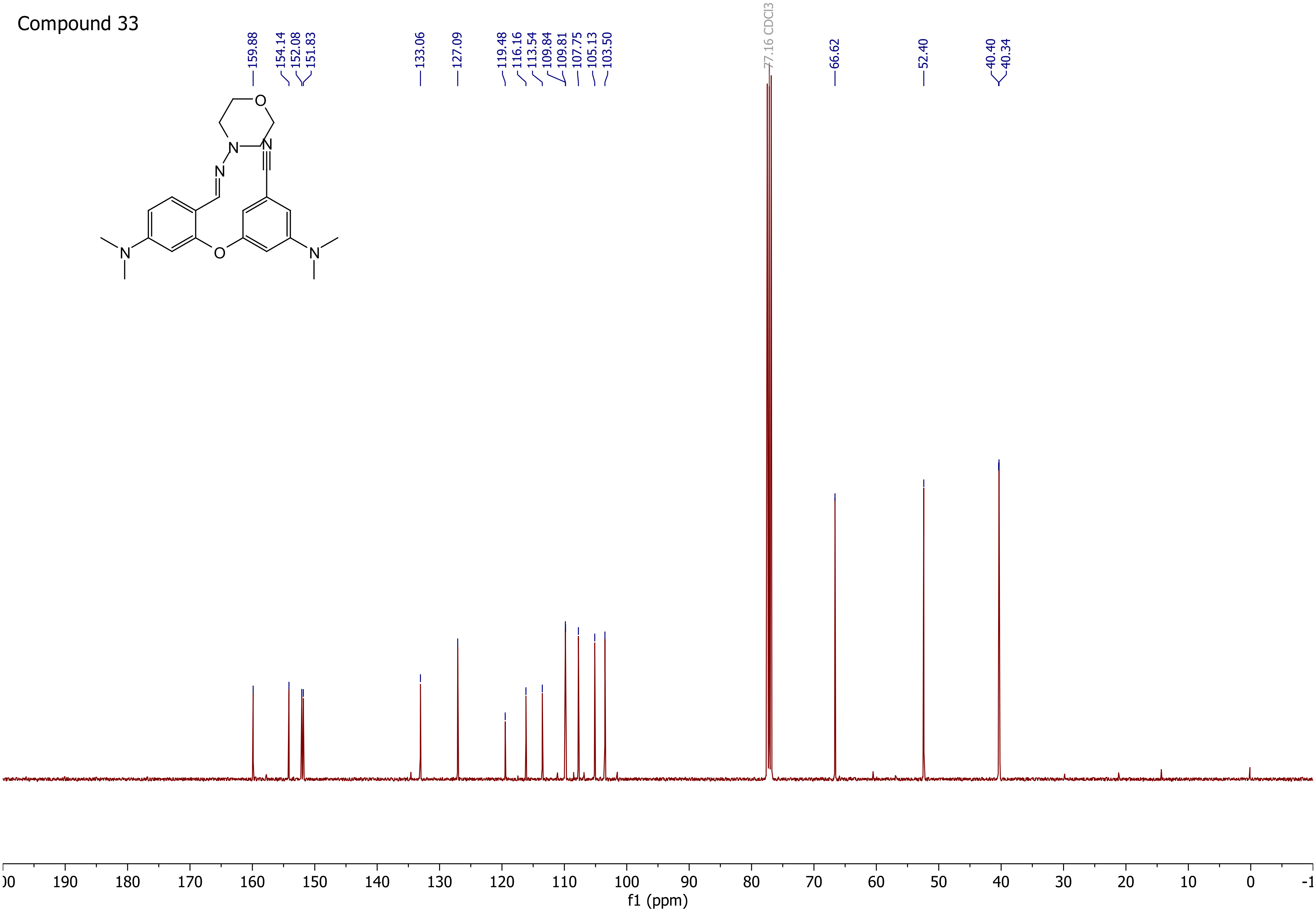
159.88
154.14
152.08
151.83
133.06
127.09
119.48
116.16
113.54
109.84
109.81
107.75
105.13
103.50
77.16 CDCl3
66.62
52.40
40.40
40.34
f1 (ppm)

Compound 34

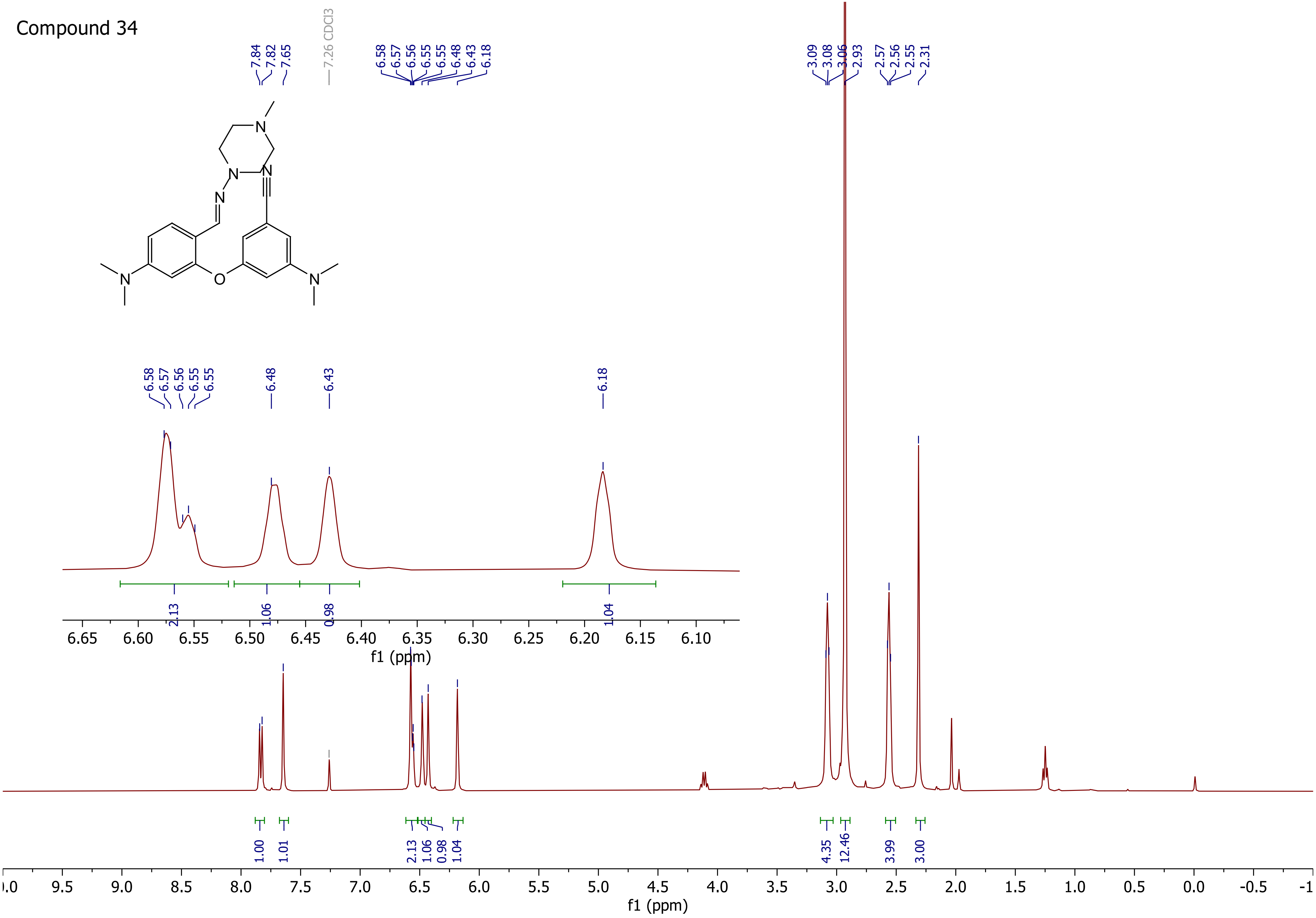

7.84
7.82
7.65
7.26 CDCl3
6.58
6.57
6.56
6.55
6.55
6.48
6.43
6.18
3.09
3.08
3.06
2.93
2.57
2.56
2.55
2.31
2.13
1.06
0.98
1.04
f1 (ppm)
1.00
1.01
4.35
12.46
3.99
3.00

Compound 34

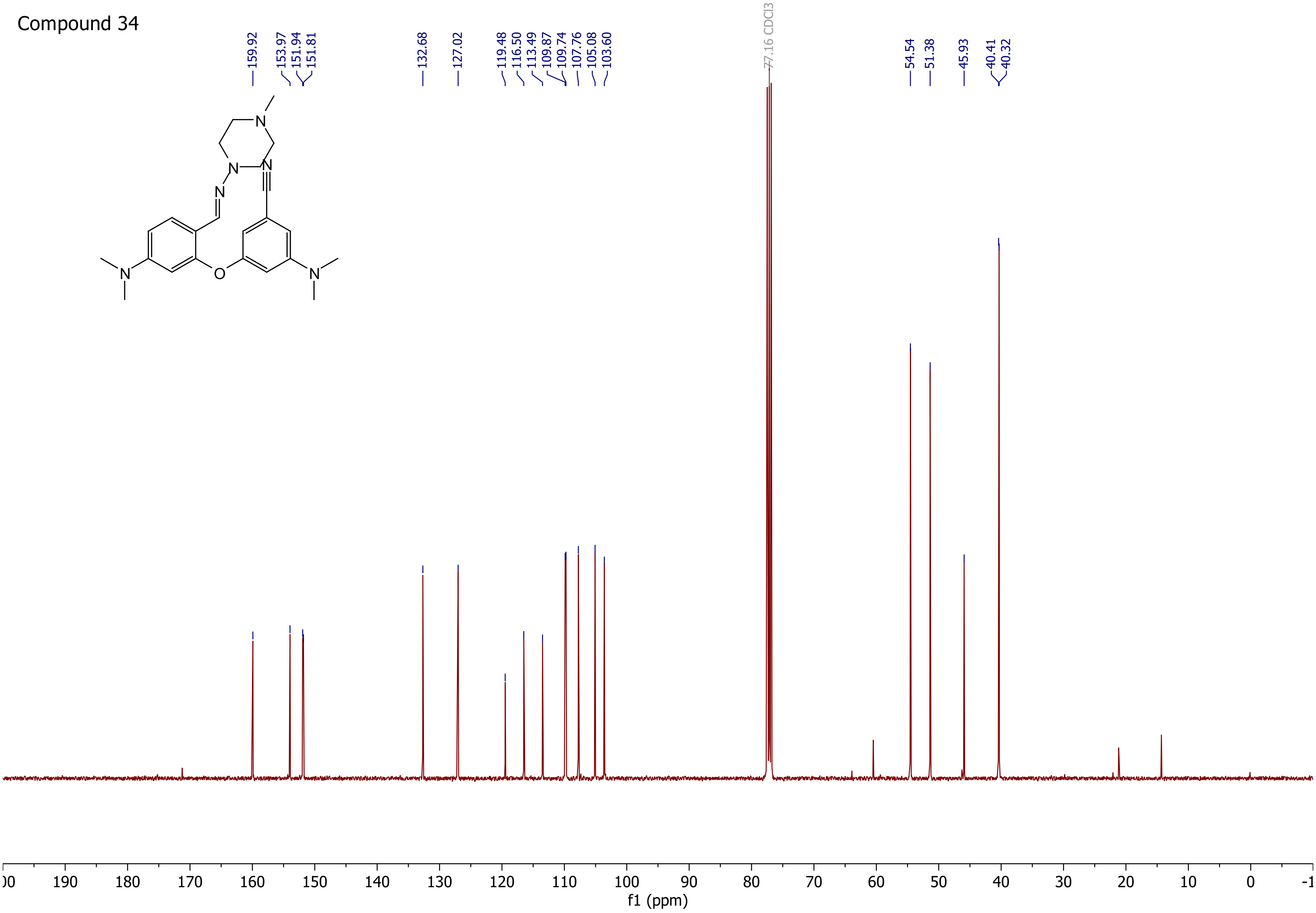

159.92
153.97
151.94
151.81
132.68
127.02
119.48
116.50
113.49
109.87
109.74
107.76
105.08
103.60
77.16 CDCl3
54.54
51.38
45.93
40.41
40.32
N
N
N
N
O
N
N
f1 (ppm)

Compound 35

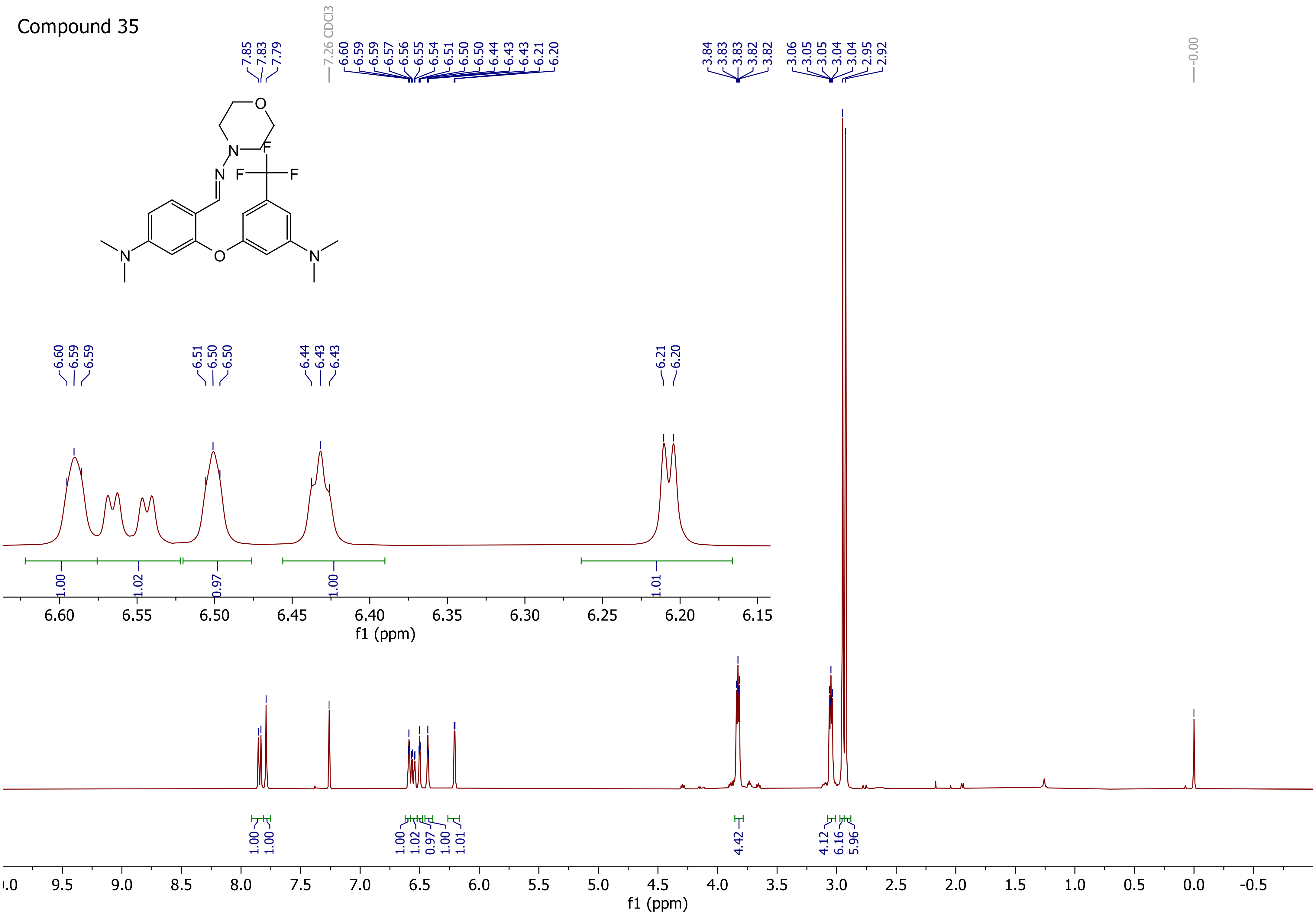

7.85
7.83
7.79
7.26 CDCl3
6.60
6.59
6.59
6.57
6.56
6.55
6.54
6.51
6.50
6.50
6.44
6.43
6.43
6.21
6.20
3.84
3.83
3.83
3.82
3.82
3.06
3.05
3.05
3.04
3.04
2.95
2.92
-0.00
1.00
1.02
0.97
1.00
1.01
4.42
4.12
6.16
5.96
f1 (ppm)

Compound 35

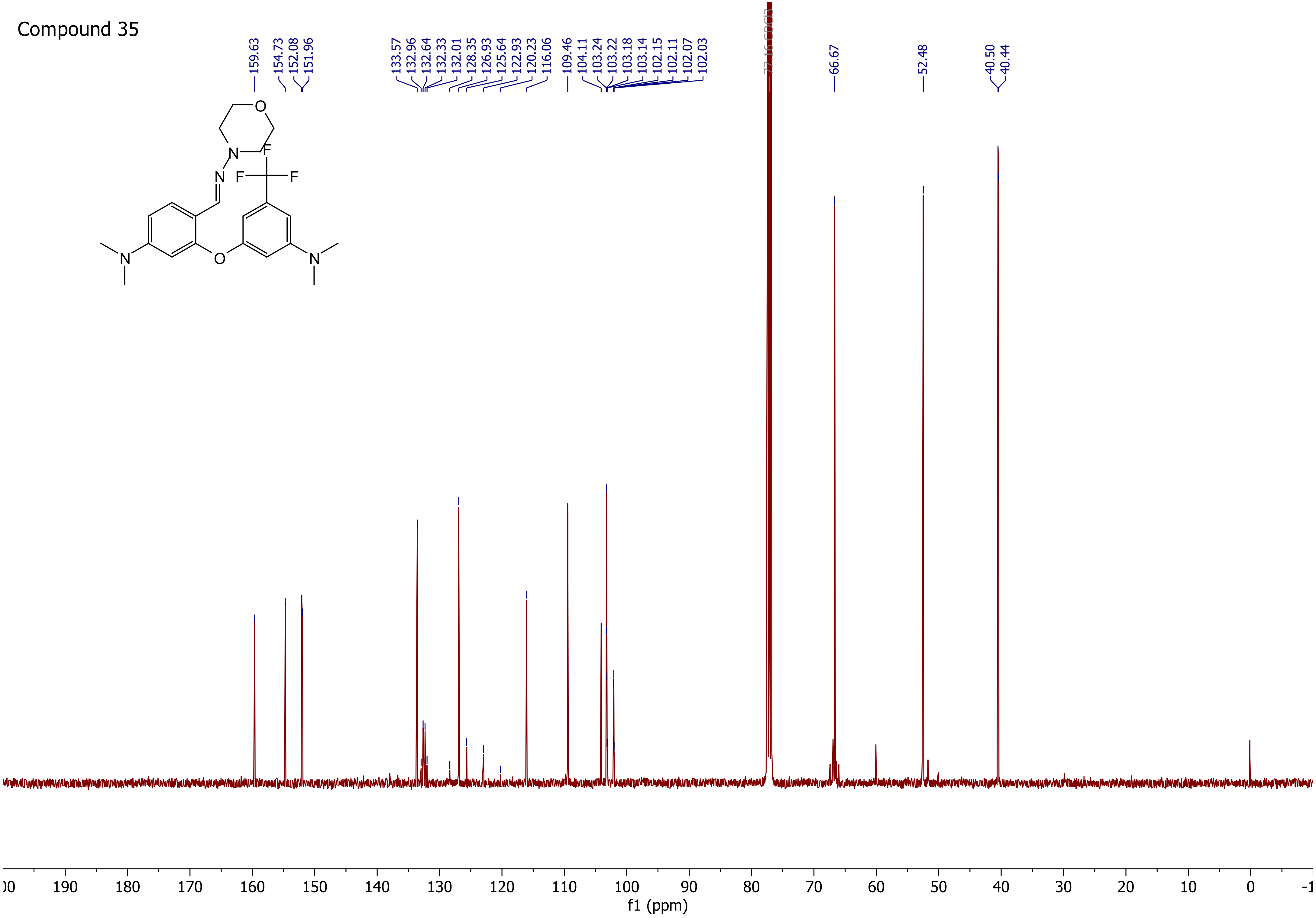

159.63
154.73
152.08
151.96
133.57
132.96
132.64
132.33
132.01
128.35
126.93
125.64
122.93
120.23
116.06
109.46
104.11
103.24
103.22
103.18
103.14
102.15
102.11
102.07
102.03
77.16 CDCl3
66.67
52.48
40.50
40.44
O
N
N
F
F
F
O
N
N
190
180
170
160
150
140
130
120
110
100
90
80
70
60
50
40
30
20
10
0
f1 (ppm)

Compound 36

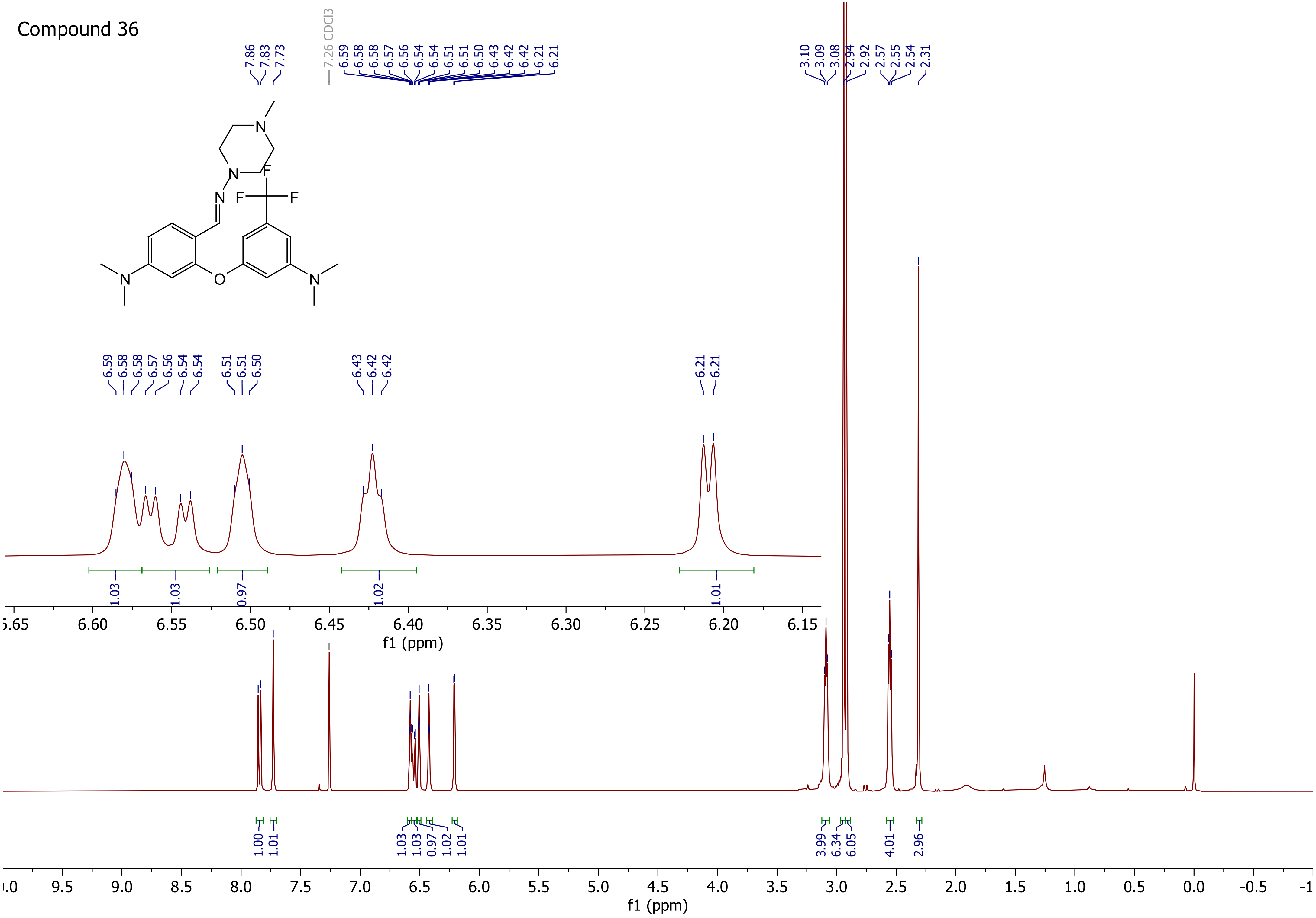

7.86
7.83
7.73
7.26 CDCl3
6.59
6.58
6.58
6.57
6.56
6.54
6.54
6.51
6.51
6.50
6.43
6.42
6.42
6.21
6.21
3.10
3.09
3.08
2.94
2.92
2.57
2.55
2.54
2.31
1.03
1.03
0.97
1.02
1.01
1.00
1.01
3.99
6.34
6.05
4.01
2.96
f1 (ppm)
f1 (ppm)

Compound 36

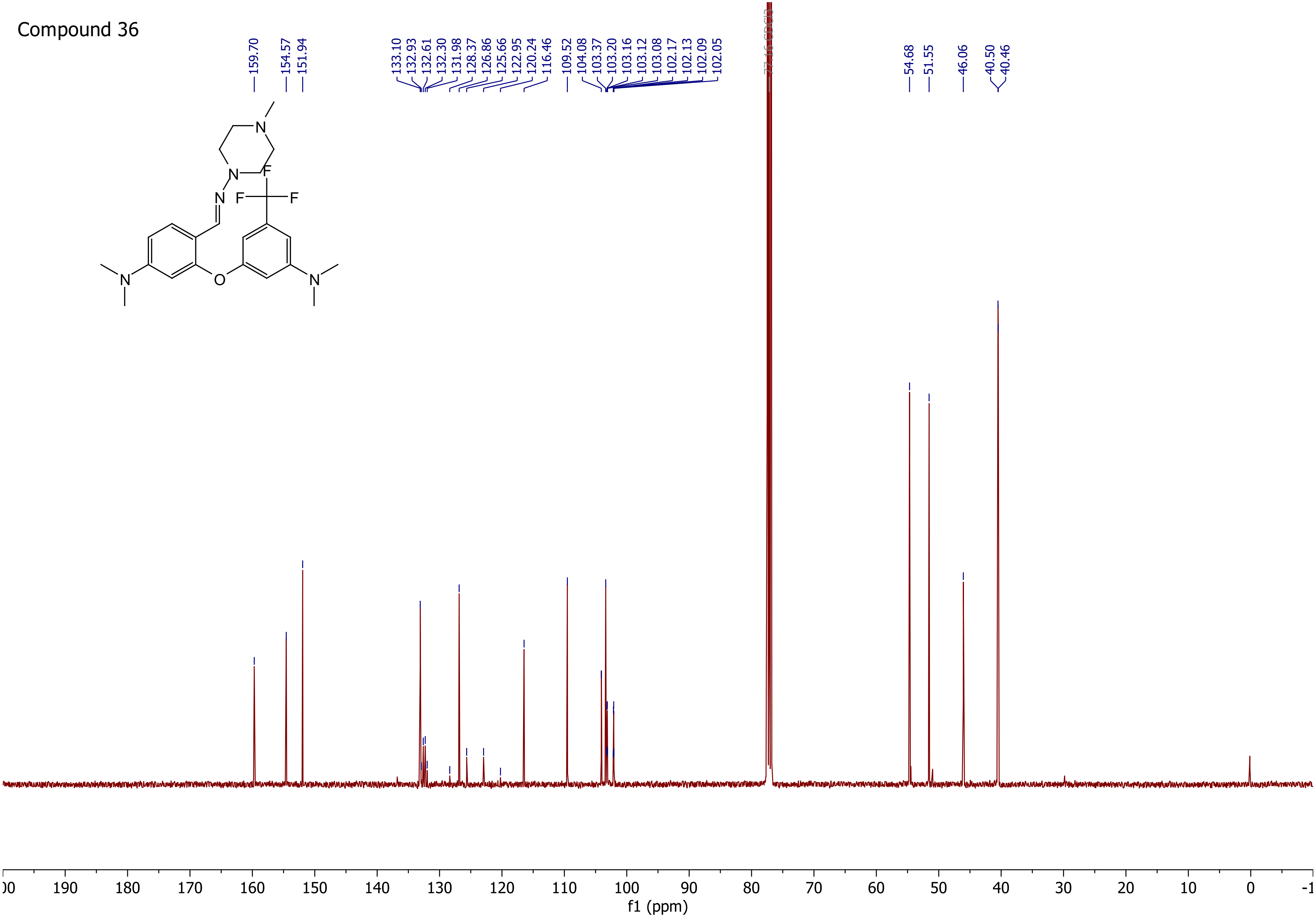

159.70
154.57
151.94
133.10
132.93
132.61
132.30
131.98
128.37
126.86
125.66
122.95
120.24
116.46
109.52
104.08
103.37
103.20
103.16
103.12
103.08
102.17
102.13
102.09
102.05
77.16 CDCl3
54.68
51.55
46.06
40.50
40.46
f1 (ppm)

Compound 40

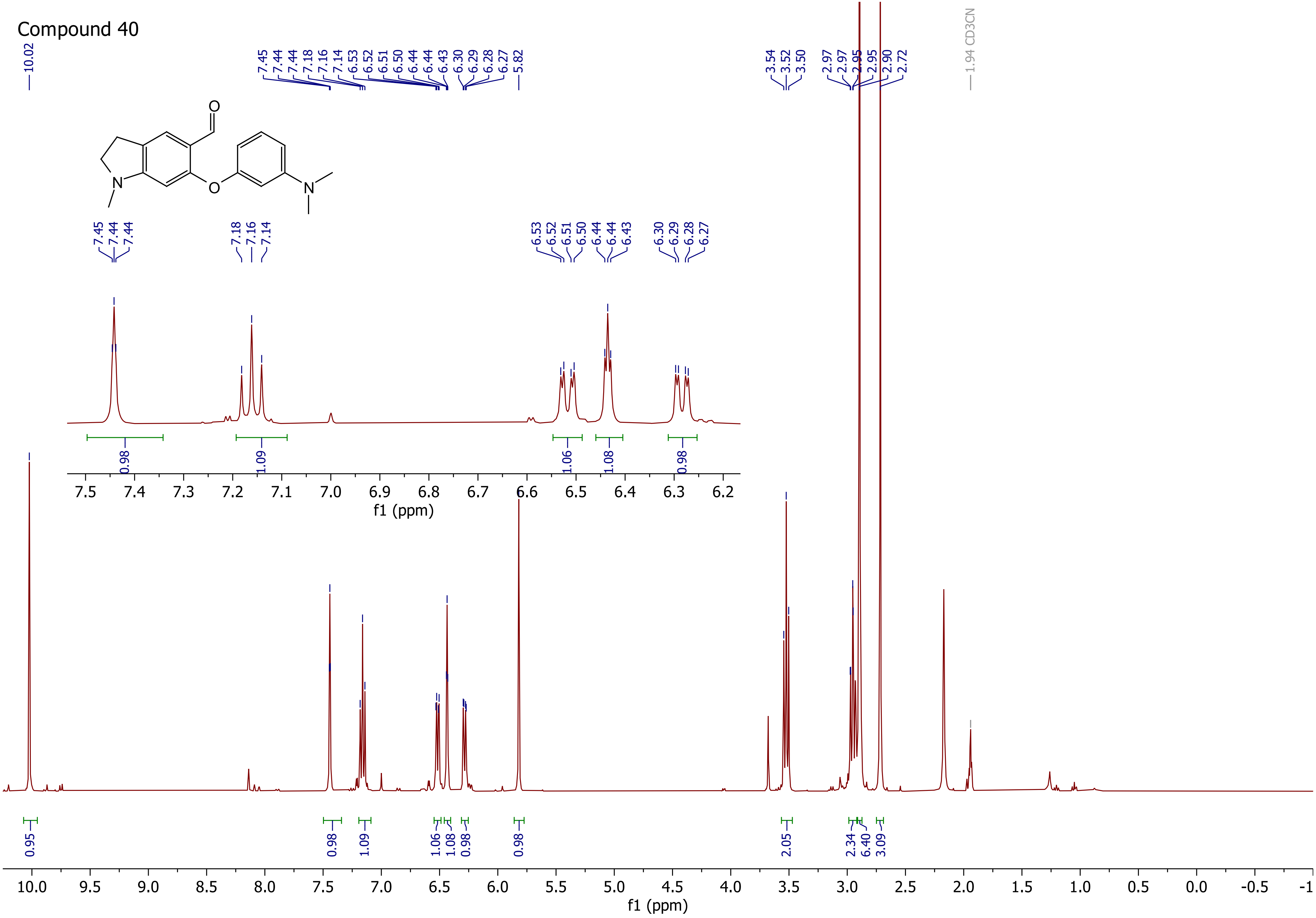
10.02
7.45
7.44
7.44
7.18
7.16
7.14
6.53
6.52
6.51
6.50
6.44
6.44
6.43
6.30
6.29
6.28
6.27
5.82
3.54
3.52
3.50
2.97
2.97
2.95
2.95
2.90
2.72
1.94 CD3CN
O
N
O
N
0.98
1.09
1.06
1.08
0.98
f1 (ppm)
0.95
2.05
2.34
6.40
3.09
f1 (ppm)

Compound 40

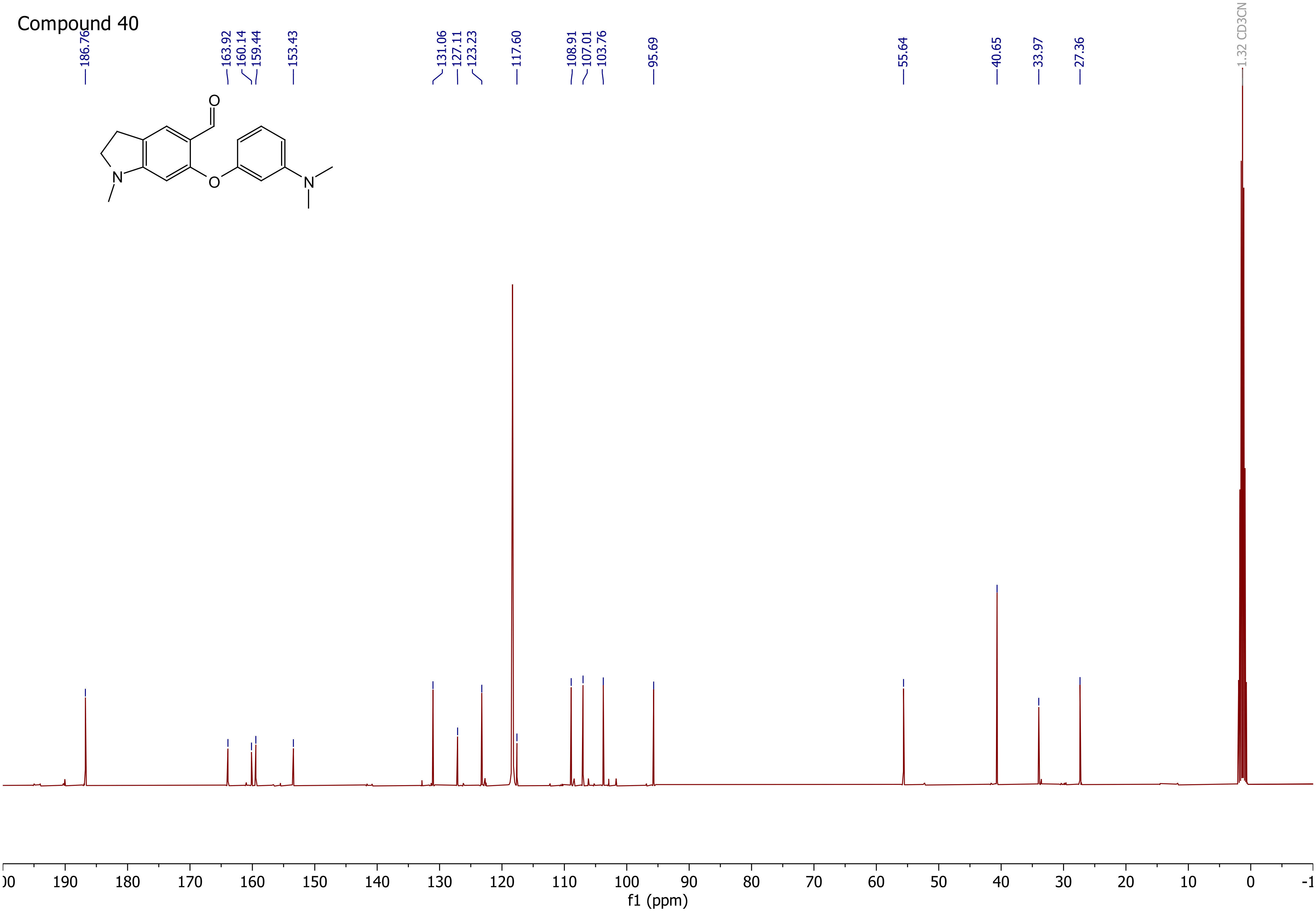

186.76
163.92
160.14
159.44
153.43
131.06
127.11
123.23
117.60
108.91
107.01
103.76
95.69
55.64
40.65
33.97
27.36
1.32 CD3CN
O
N
O
N
190 180 170 160 150 140 130 120 110 100 90 80 70 60 50 40 30 20 10 0
f1 (ppm)

Compound 41

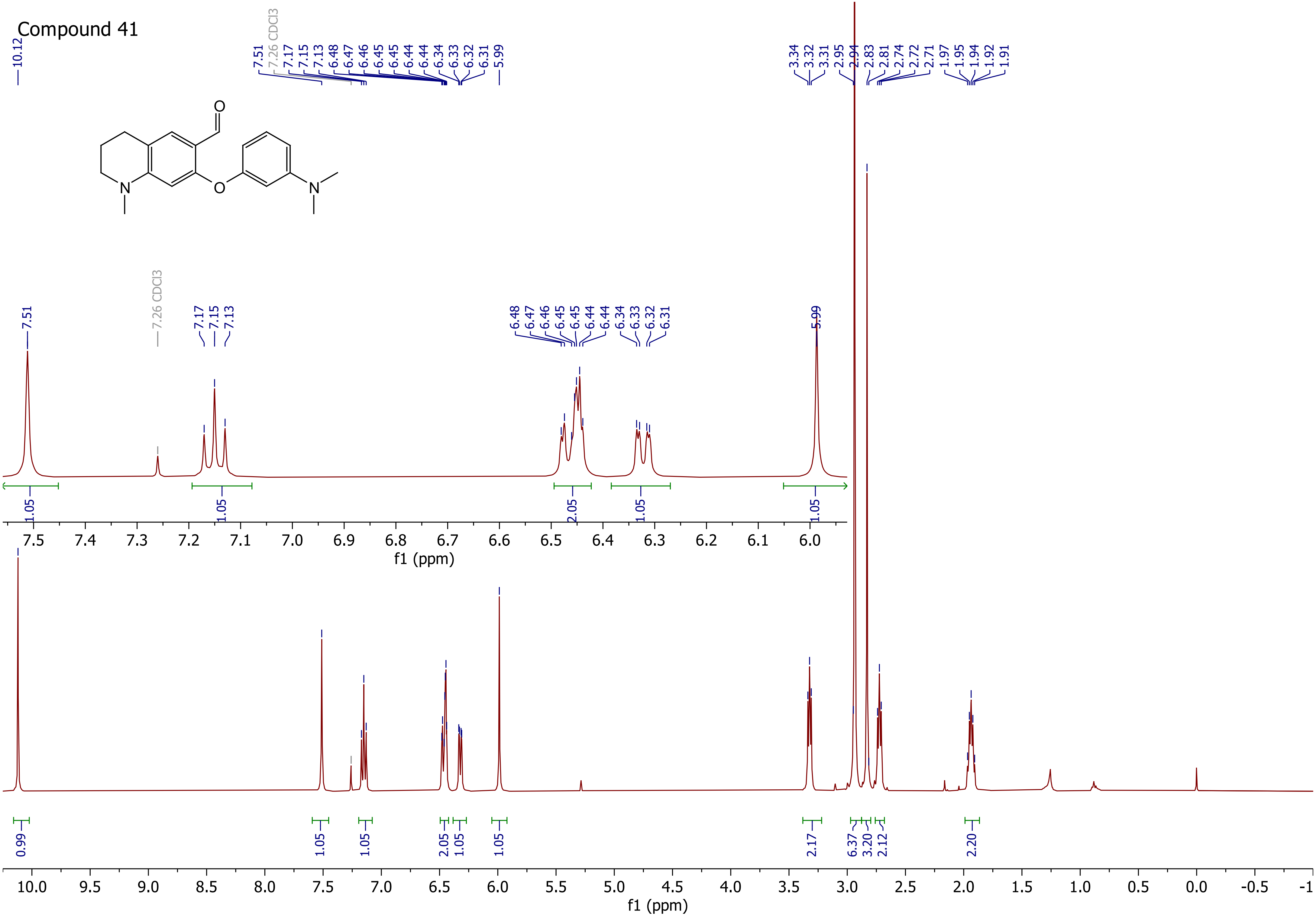
10.12
7.51
7.26 CDCl3
7.17
7.15
7.13
6.48
6.47
6.46
6.45
6.45
6.44
6.44
6.34
6.33
6.32
6.31
5.99
3.34
3.32
3.31
2.95
2.94
2.83
2.81
2.74
2.72
2.71
1.97
1.95
1.94
1.92
1.91
O
N
O
N
1.05
1.05
2.05
1.05
1.05
f1 (ppm)
0.99
1.05
1.05
2.05
1.05
1.05
2.17
6.37
3.20
2.12
2.20
f1 (ppm)

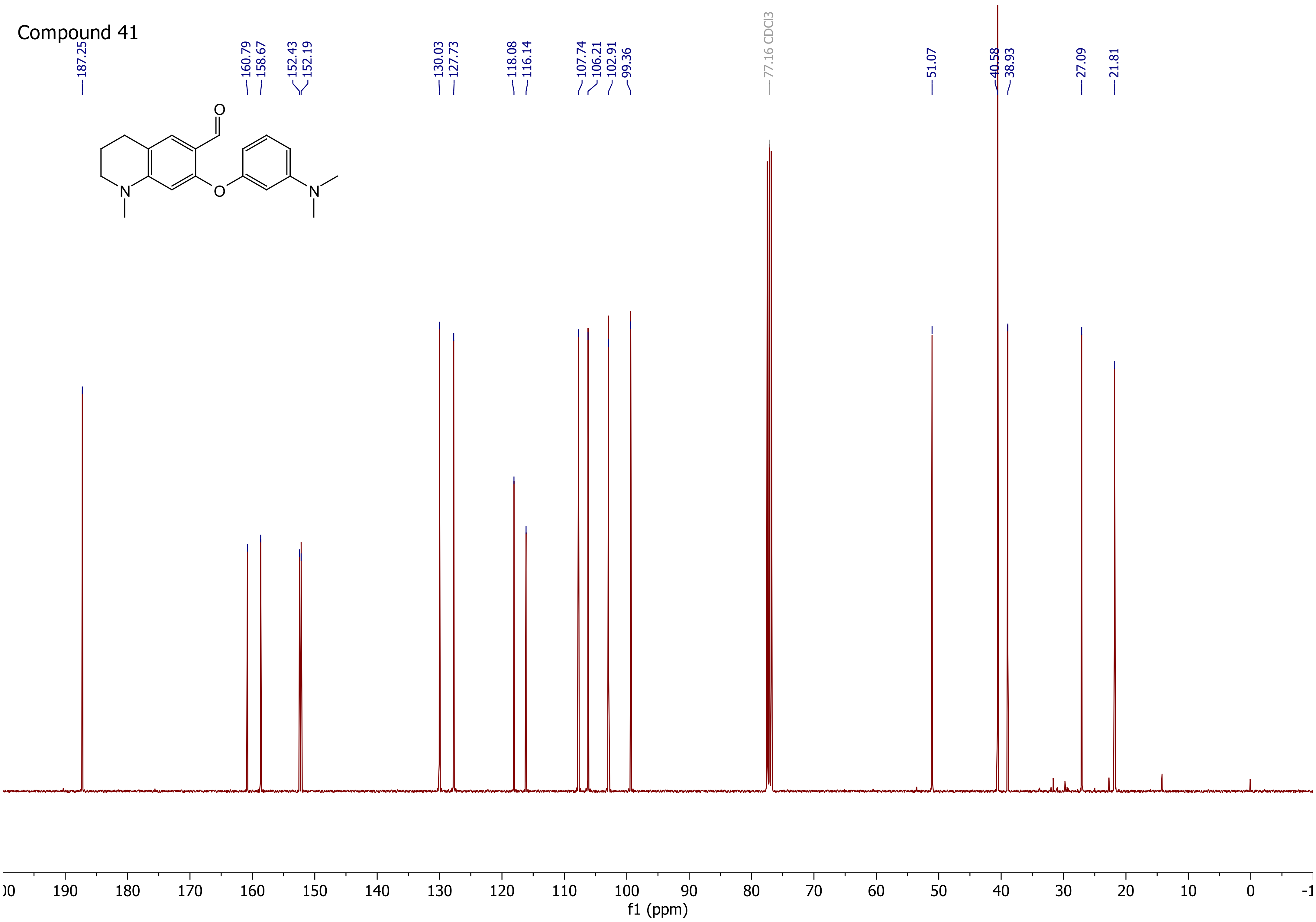
Compound 41
187.25
160.79
158.67
152.43
152.19
130.03
127.73
118.08
116.14
107.74
106.21
102.91
99.36
77.16 CDCl3
51.07
40.58
38.93
27.09
21.81
O
N
O
N
190
180
170
160
150
140
130
120
110
100
90
80
70
60
50
40
30
20
10
0
f1 (ppm)

Compound 42

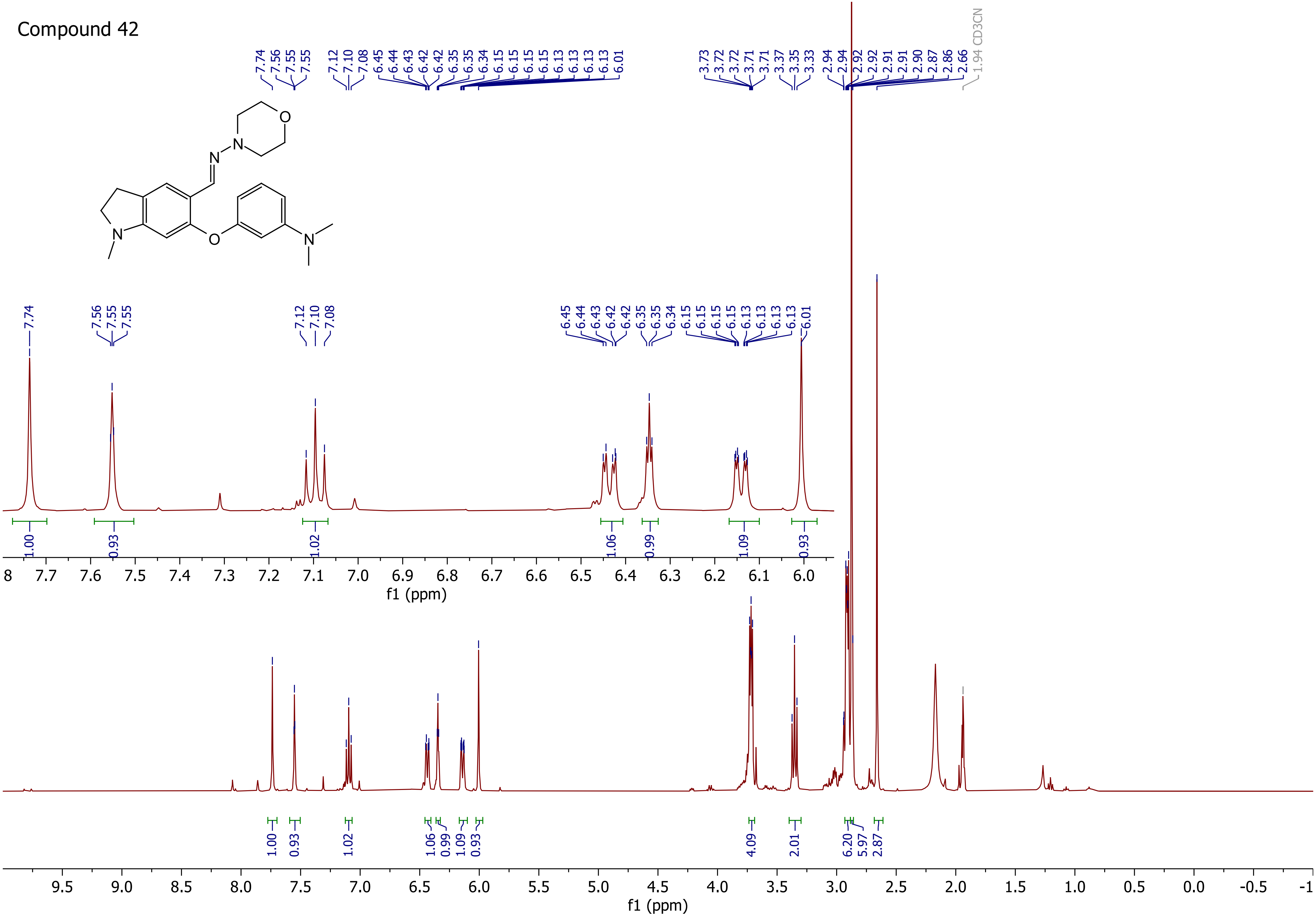

7.74
7.56
7.55
7.55
7.12
7.10
7.08
6.45
6.44
6.43
6.42
6.42
6.35
6.35
6.34
6.15
6.15
6.15
6.15
6.13
6.13
6.13
6.13
6.01
3.73
3.72
3.72
3.71
3.71
3.37
3.35
3.33
2.94
2.94
2.92
2.92
2.91
2.91
2.90
2.87
2.86
2.66
1.94 CD3CN
N
N
O
N
O
N
1.00
0.93
1.02
1.06
0.99
1.09
0.93
4.09
2.01
6.20
5.97
2.87
f1 (ppm)
f1 (ppm)

Compound 42

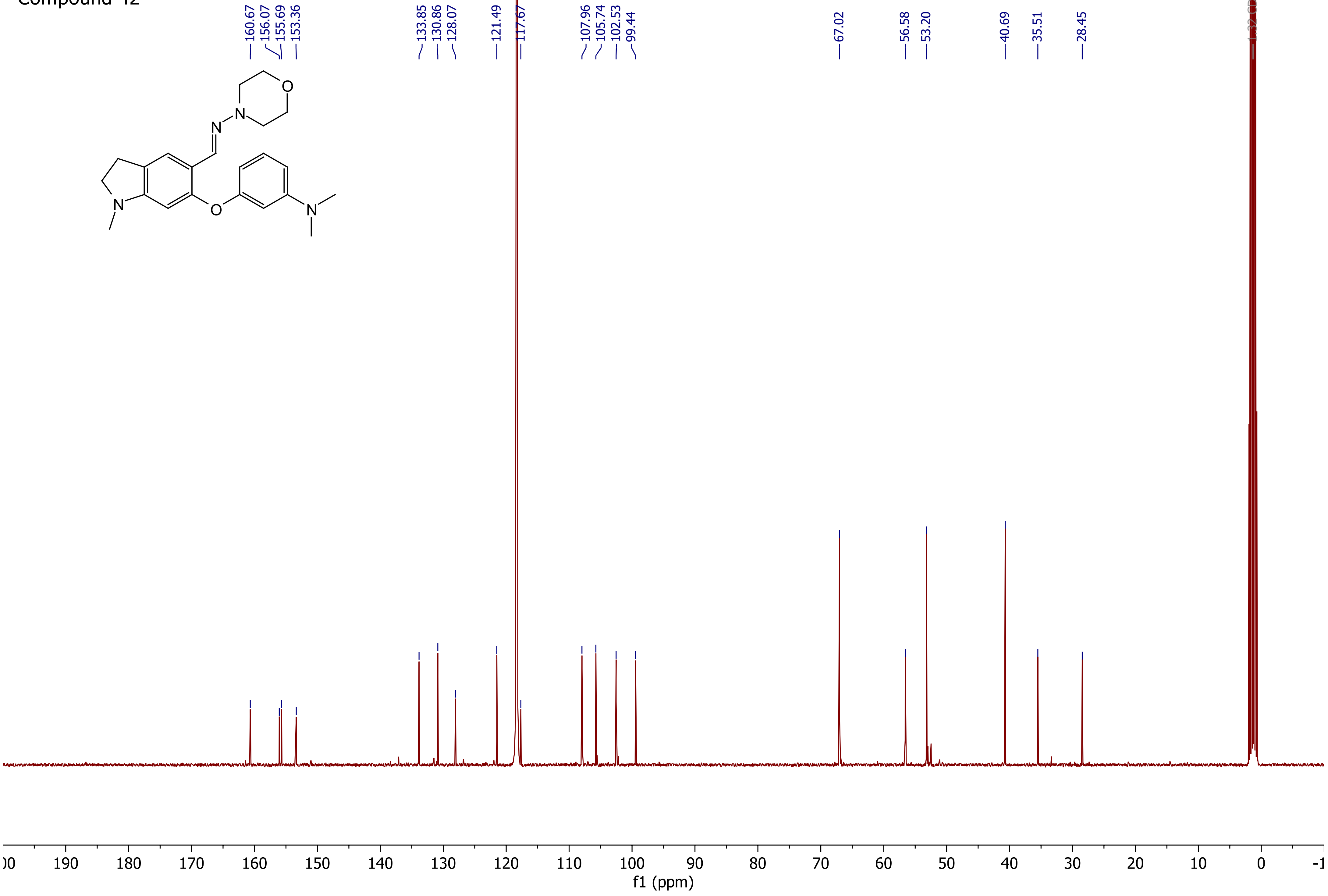

160.67
156.07
155.69
153.36
133.85
130.86
128.07
121.49
117.67
107.96
105.74
102.53
99.44
67.02
56.58
53.20
40.69
35.51
28.45
1.32 CD3CN
O
N
N
N
O
N
f1 (ppm)

Compound 43

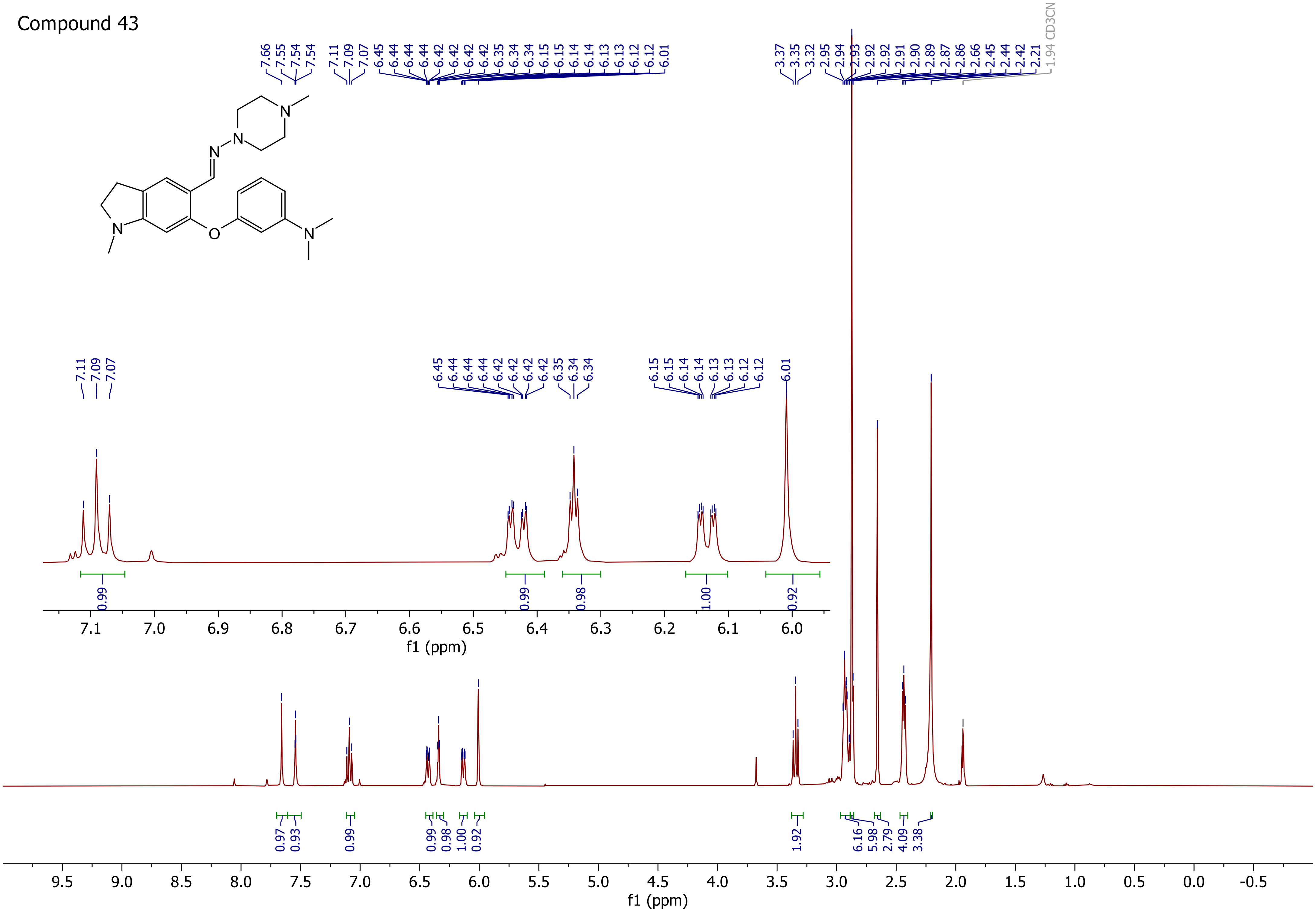
7.66 7.55 7.54 7.54 7.11 7.09 7.07 6.45 6.44 6.44 6.42 6.42 6.42 6.42 6.35 6.34 6.34 6.15 6.15 6.14 6.14 6.13 6.13 6.12 6.12 6.01
3.37 3.35 3.32 2.95 2.94 2.93 2.92 2.92 2.91 2.90 2.89 2.87 2.86 2.66 2.45 2.44 2.42 2.21 1.94 CD3CN
N
N
N
N
O
N
7.11 7.09 7.07
6.45 6.44 6.44 6.44 6.42 6.42 6.42 6.42 6.35 6.34 6.34
6.15 6.15 6.14 6.14 6.13 6.13 6.12 6.12 6.01
0.99 0.99 0.98 1.00 0.92
7.1 7.0 6.9 6.8 6.7 6.6 6.5 6.4 6.3 6.2 6.1 6.0
f1 (ppm)
0.97 0.93 0.99 0.99 0.98 1.00 0.92 1.92 6.16 5.98 2.79 4.09 3.38
9.5 9.0 8.5 8.0 7.5 7.0 6.5 6.0 5.5 5.0 4.5 4.0 3.5 3.0 2.5 2.0 1.5 1.0 0.5 0.0 -0.5
f1 (ppm)

Compound 43

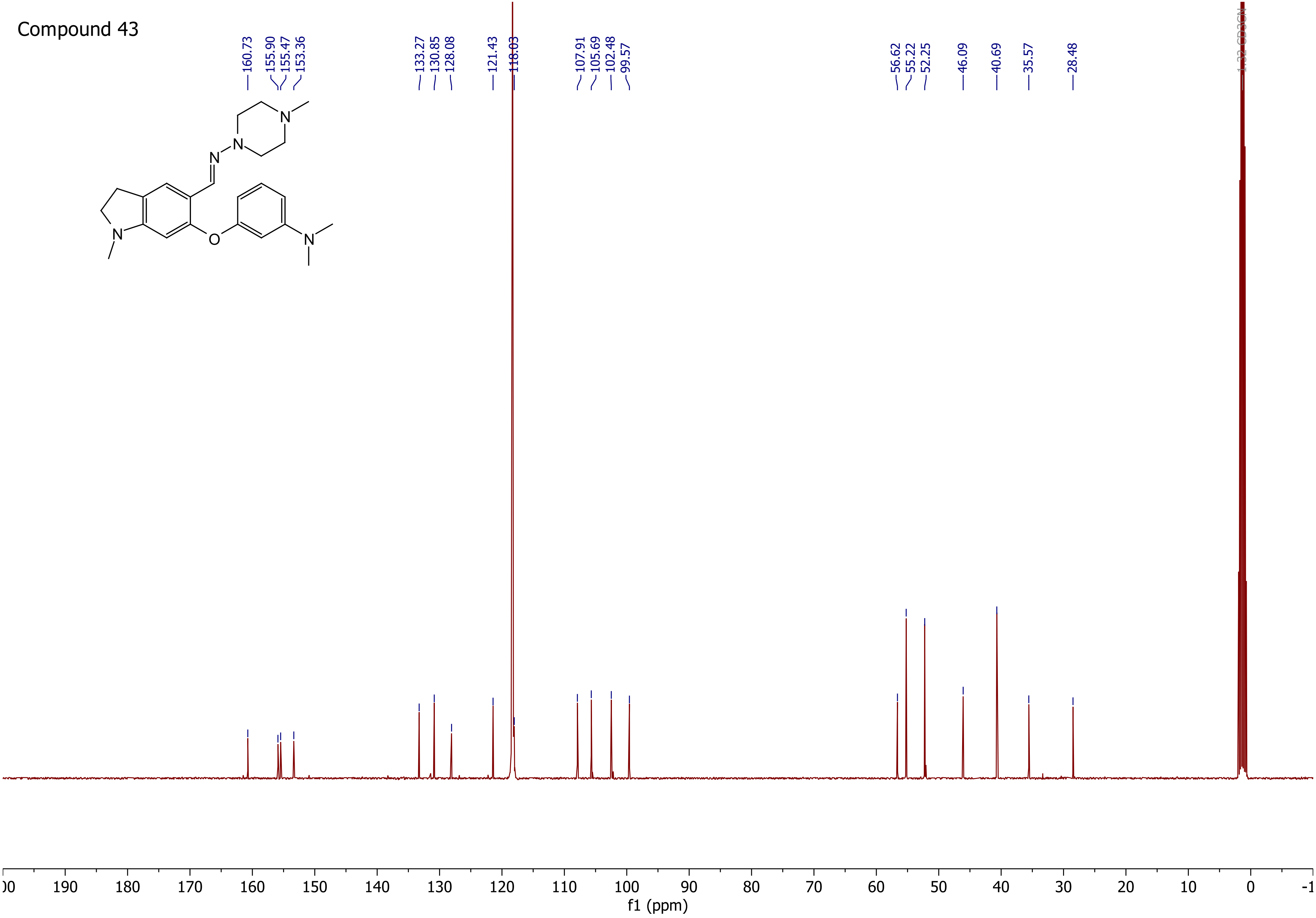
160.73
155.90
155.47
153.36
133.27
130.85
128.08
121.43
118.03
107.91
105.69
102.48
99.57
56.62
55.22
52.25
46.09
40.69
35.57
28.48
1.32 CD3CN
N
N
N
N
O
N
f1 (ppm)
190 180 170 160 150 140 130 120 110 100 90 80 70 60 50 40 30 20 10 0 -1

Compound 44

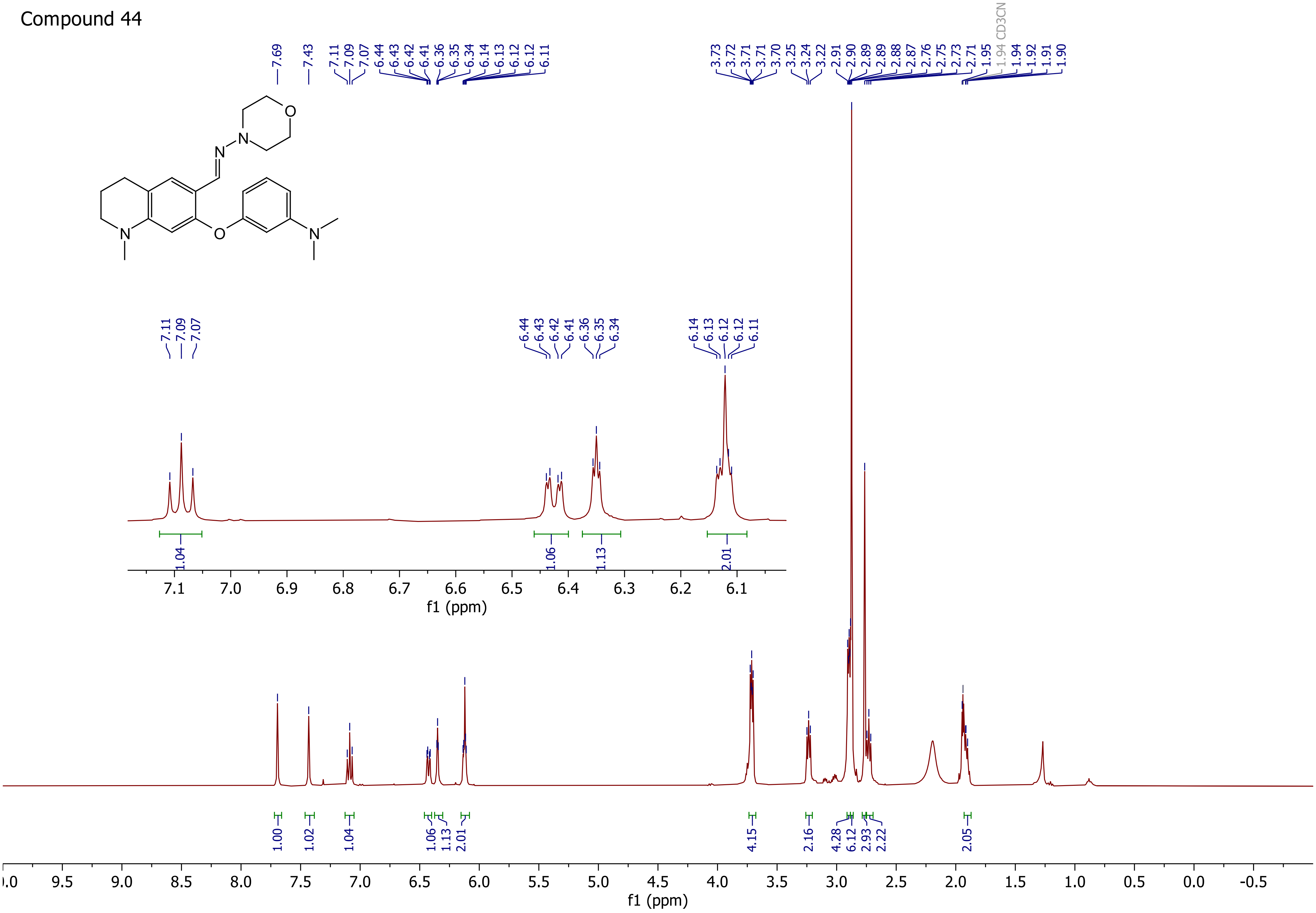

7.69
7.43
7.11
7.09
7.07
6.44
6.43
6.42
6.41
6.36
6.35
6.34
6.14
6.13
6.12
6.12
6.11
3.73
3.72
3.71
3.71
3.70
3.25
3.24
3.22
2.91
2.90
2.89
2.89
2.88
2.87
2.76
2.75
2.73
2.71
1.95
1.94 CD3CN
1.94
1.92
1.91
1.90
O
N
N
N
O
N
1.04
1.06
1.13
2.01
f1 (ppm)
1.00
1.02
1.04
1.06
1.13
2.01
4.15
2.16
4.28
6.12
2.93
2.22
2.05
f1 (ppm)

Compound 44

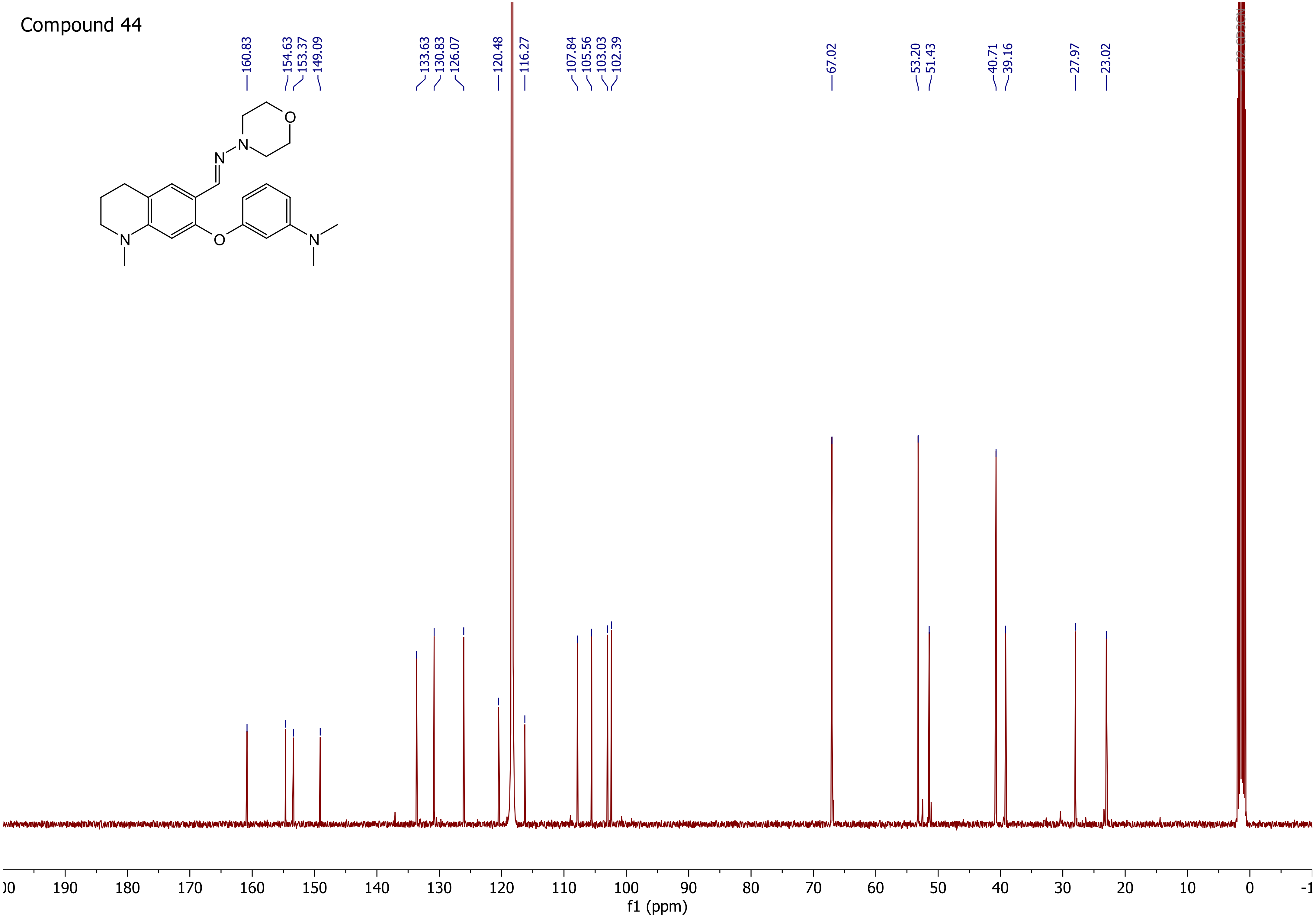

160.83
154.63
153.37
149.09
133.63
130.83
126.07
120.48
116.27
107.84
105.56
103.03
102.39
67.02
53.20
51.43
40.71
39.16
27.97
23.02
1.32 CD3CN
N
O
N
N
O
N
190 180 170 160 150 140 130 120 110 100 90 80 70 60 50 40 30 20 10 0 -1
f1 (ppm)

Compound 45

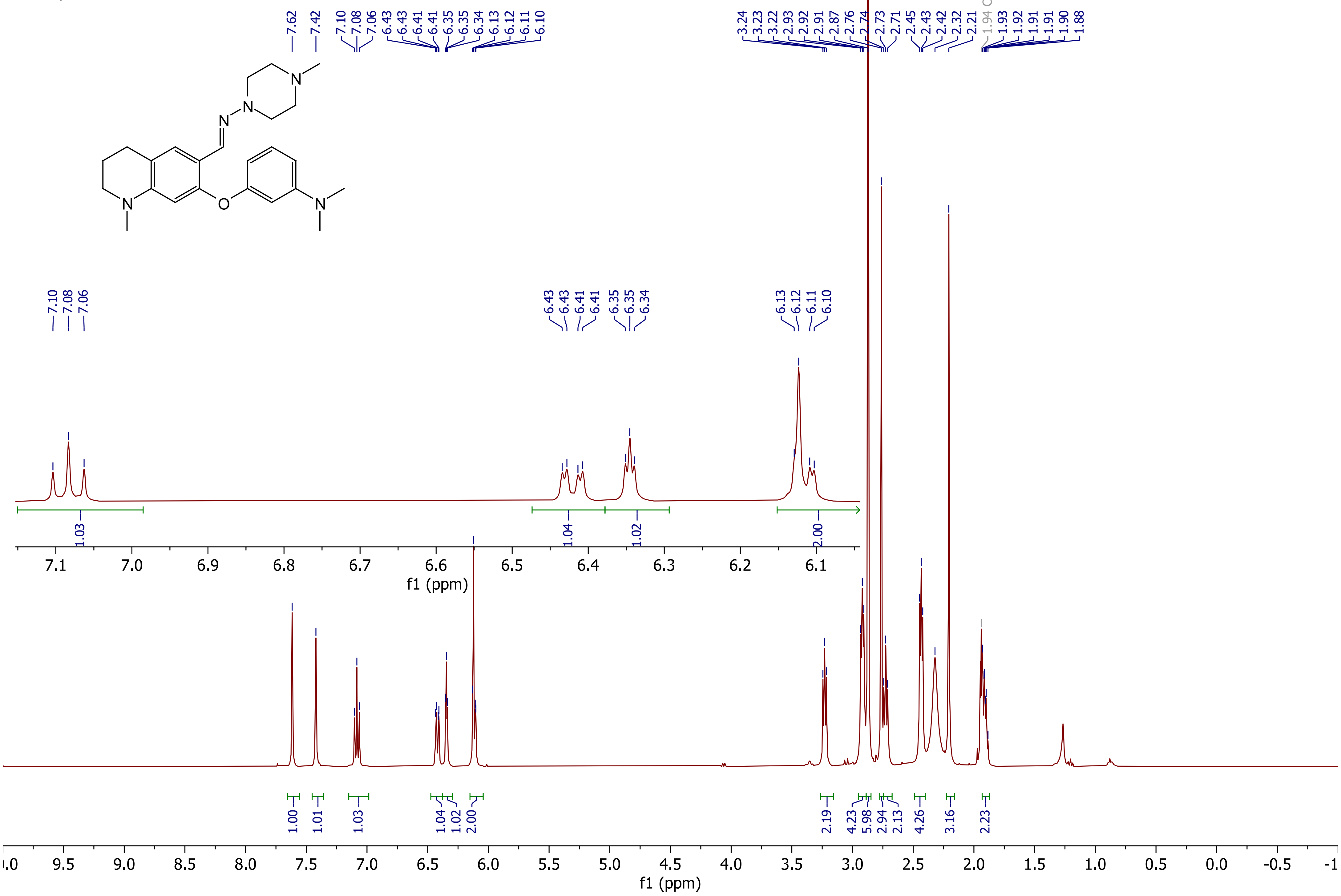

7.62
7.42
7.10
7.08
7.06
6.43
6.43
6.41
6.41
6.35
6.35
6.34
6.13
6.12
6.11
6.10
3.24
3.23
3.22
2.93
2.92
2.91
2.87
2.76
2.74
2.73
2.71
2.45
2.43
2.42
2.32
2.21
1.94 CD3CN
1.93
1.92
1.91
1.91
1.90
1.88
1.03
1.04
1.02
2.00
f1 (ppm)
1.00
1.01
1.03
1.04
1.02
2.00
2.19
4.23
5.98
2.94
2.13
4.26
3.16
2.23
f1 (ppm)

Compound 45

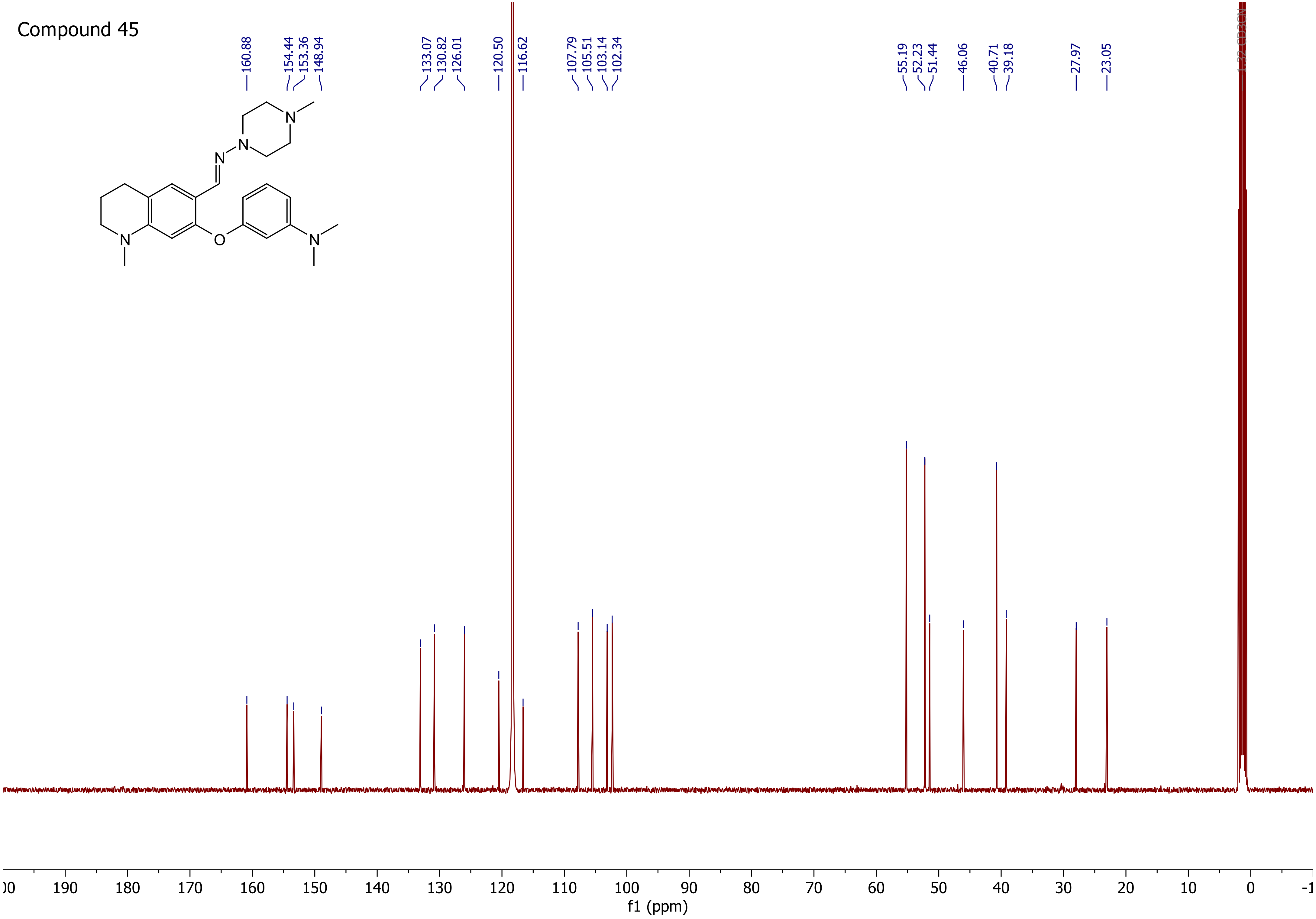

160.88
154.44
153.36
148.94
133.07
130.82
126.01
120.50
116.62
107.79
105.51
103.14
102.34
55.19
52.23
51.44
46.06
40.71
39.18
27.97
23.05
1.32 CD3CN
N
N
N
N
O
N
f1 (ppm)

Compound SI-1

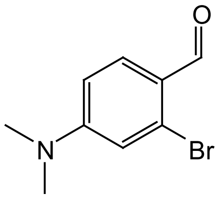

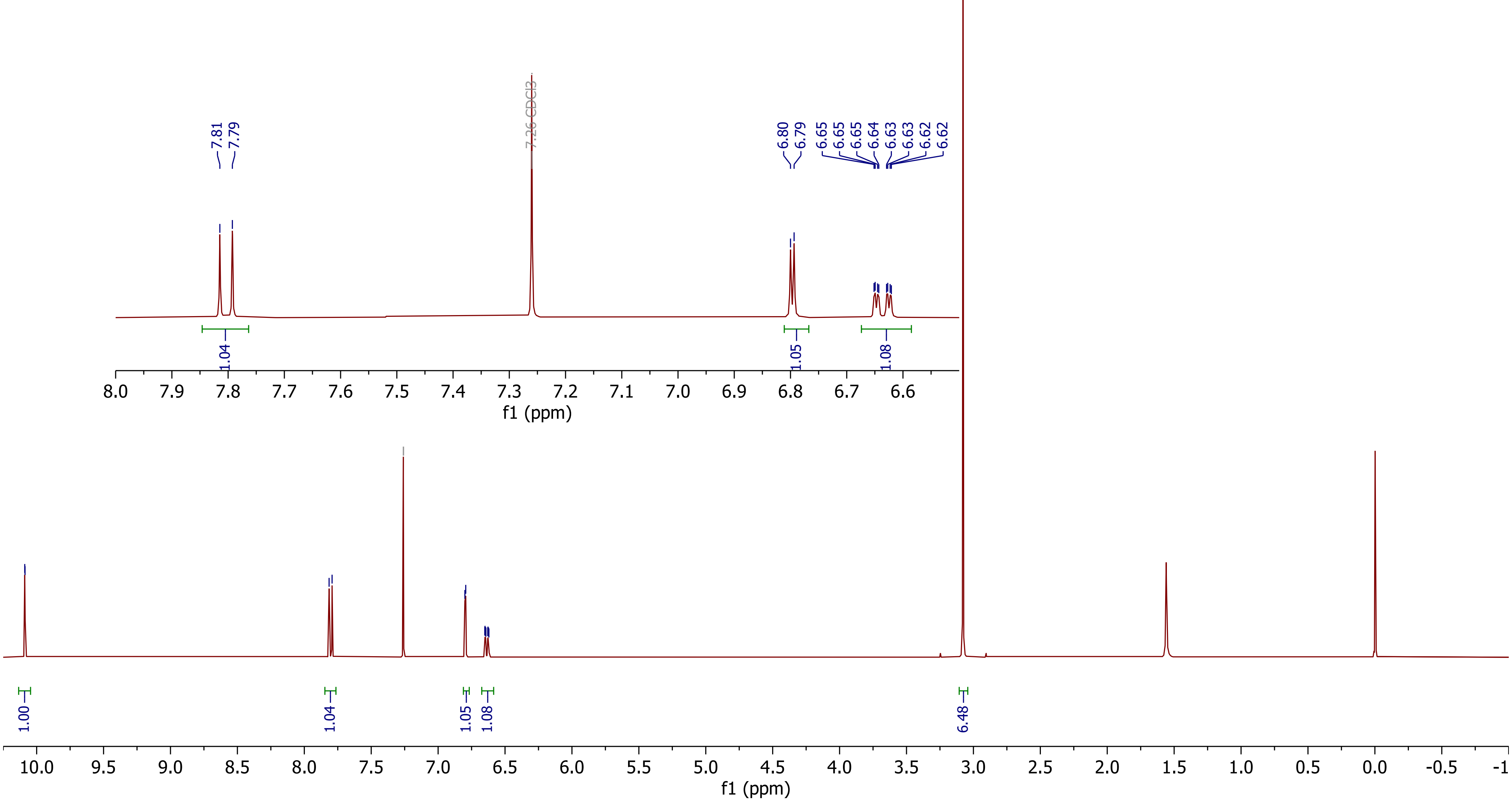

Compound SI-1

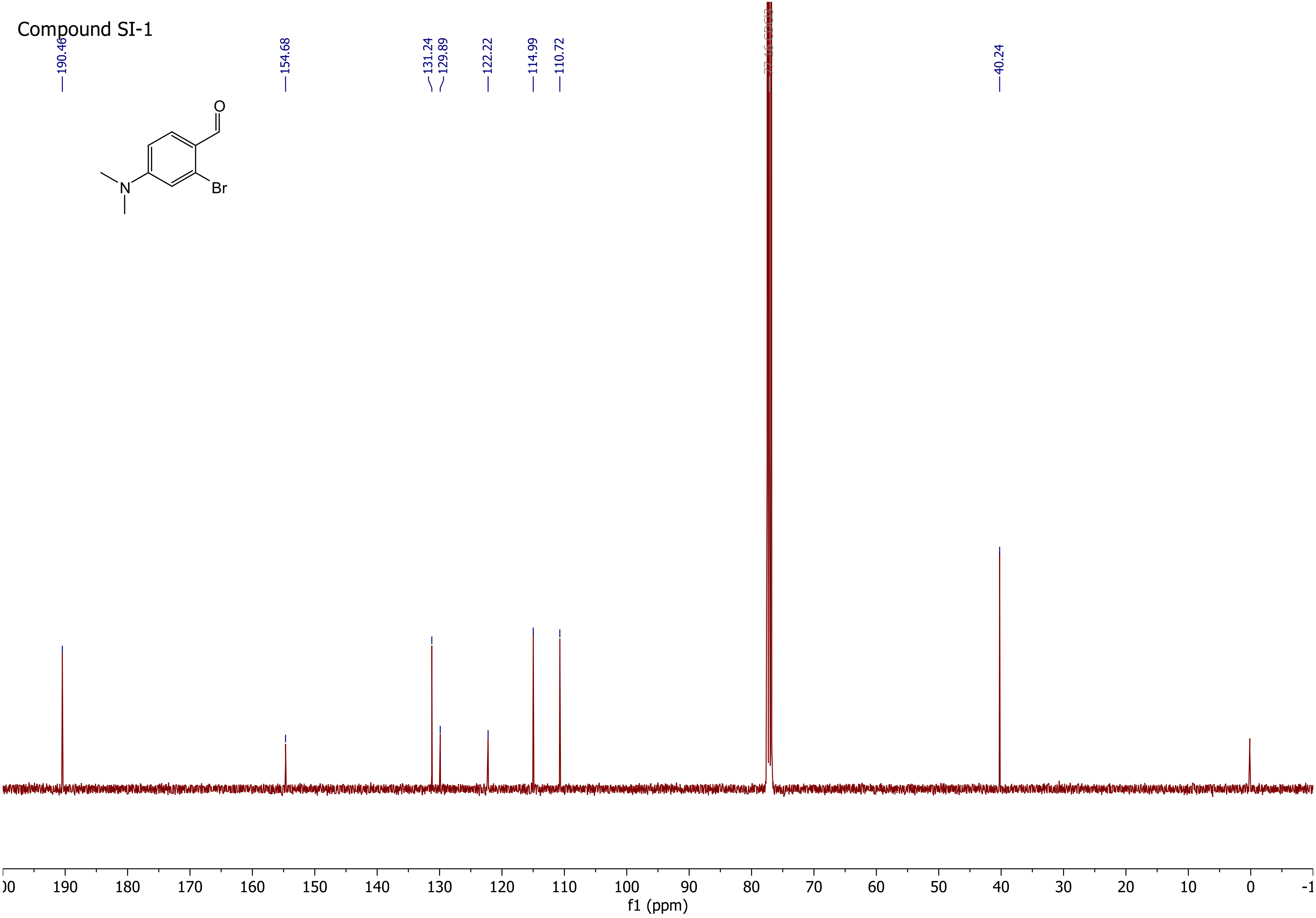

Compound SI-2

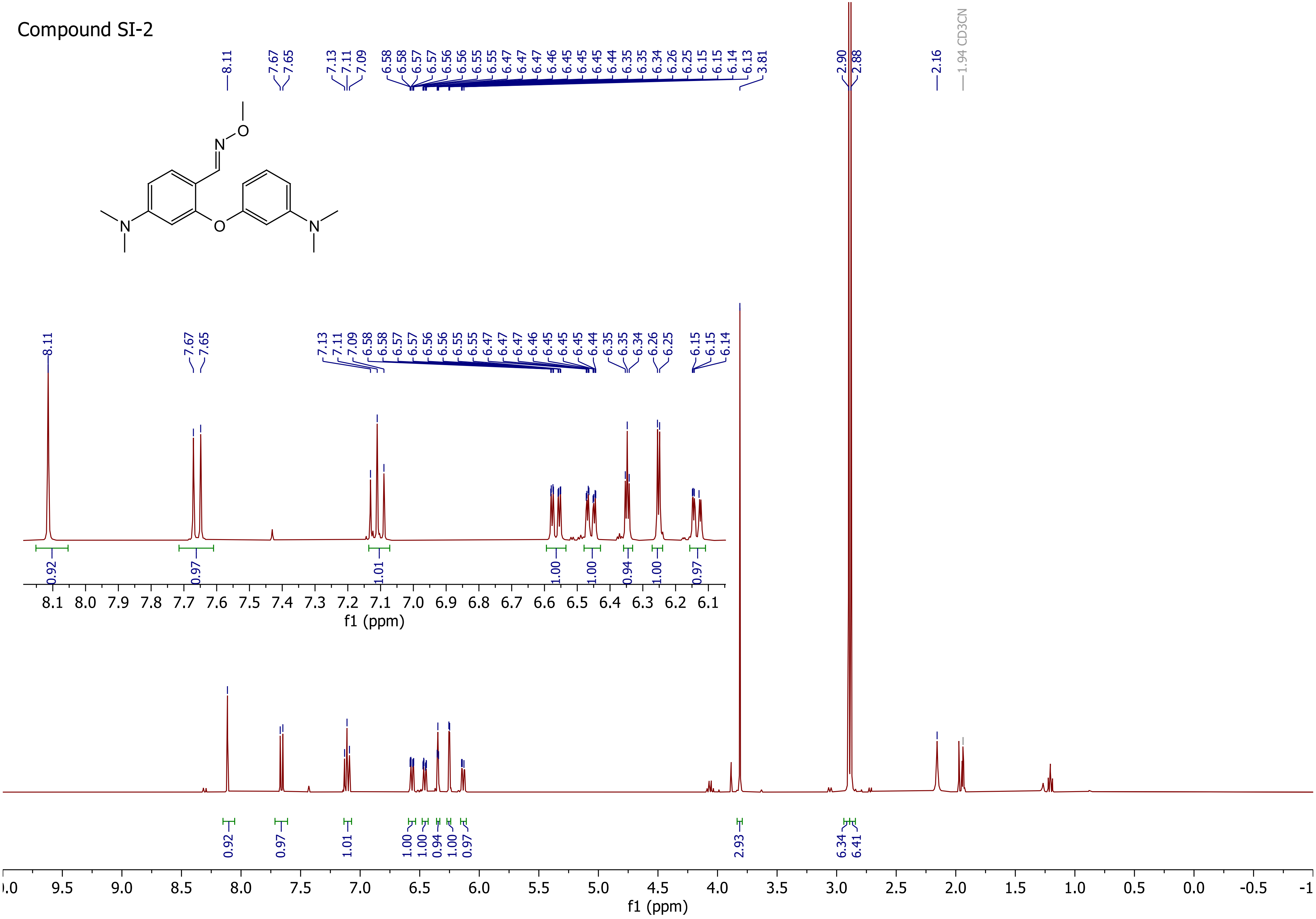

Compound SI-2

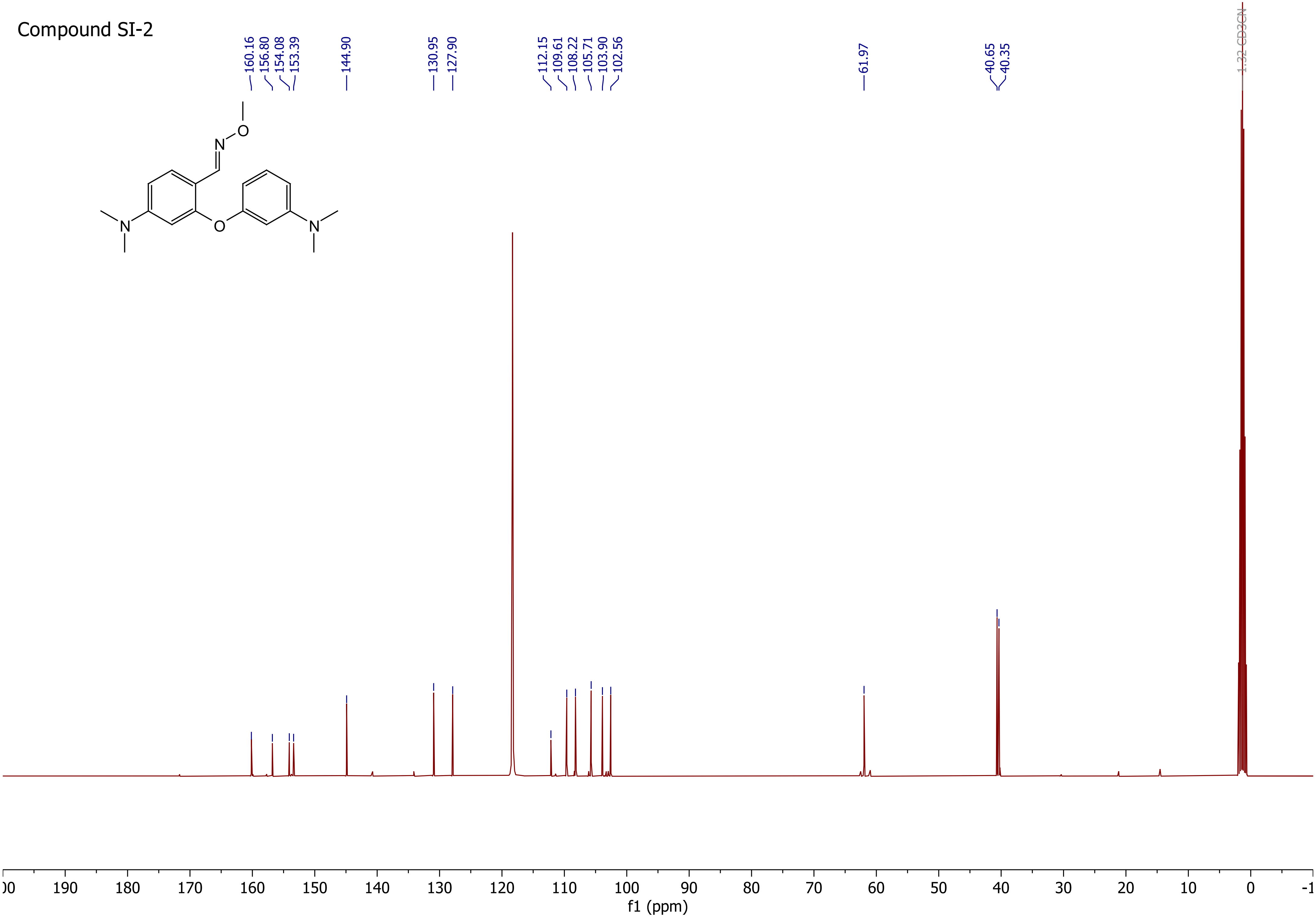

Compound SI-3

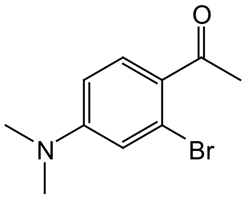

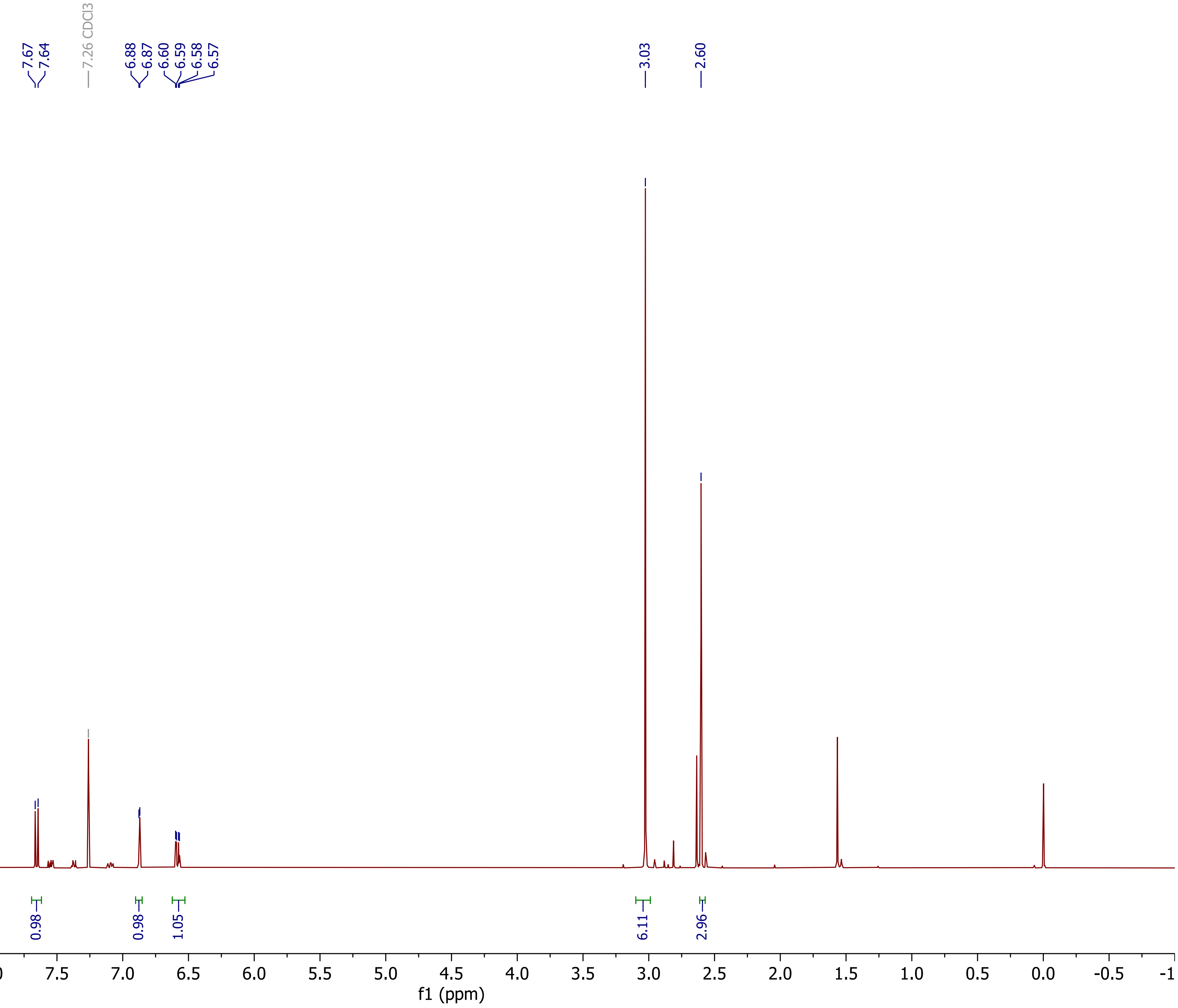

Compound SI-3

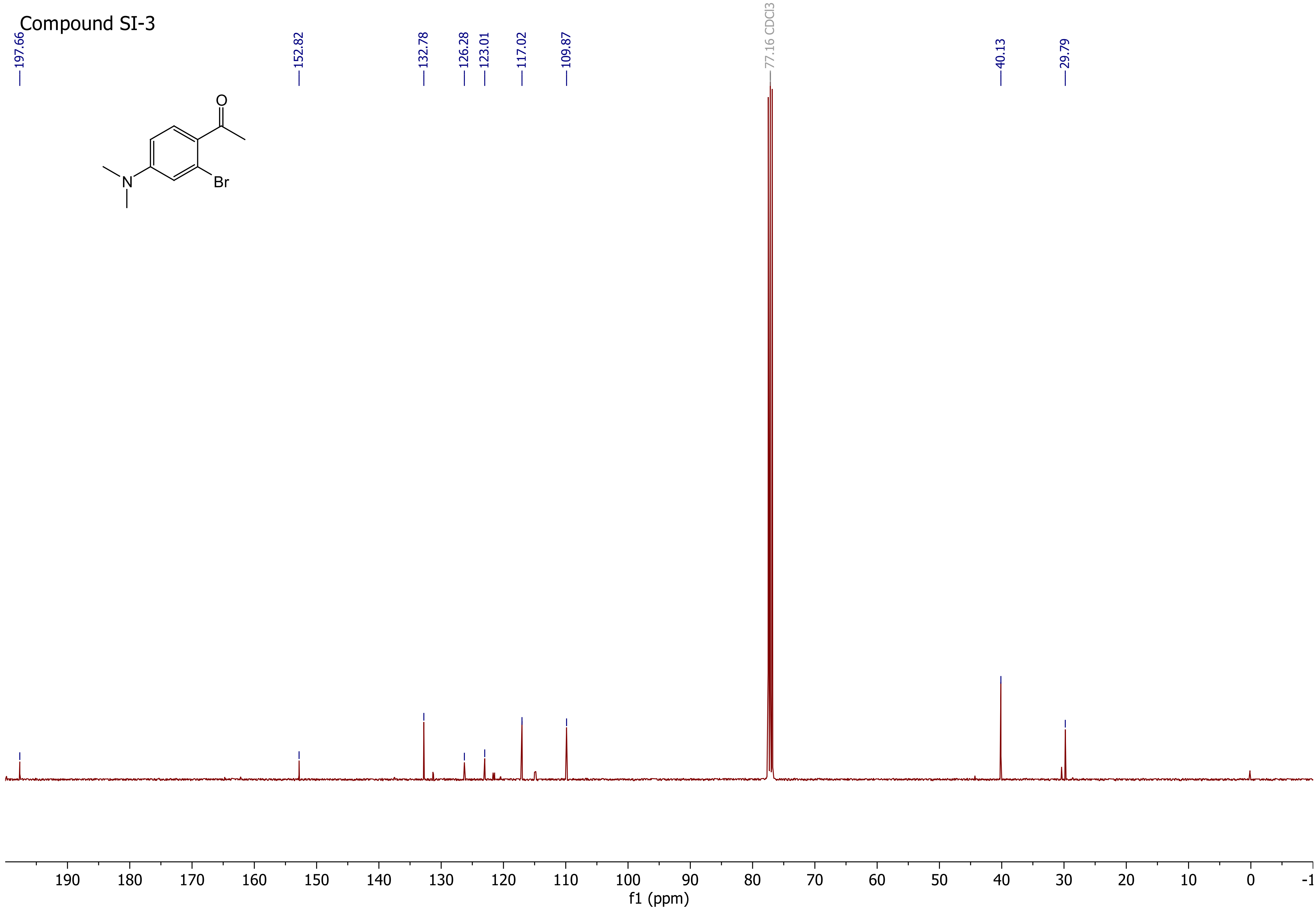

Compound SI-4

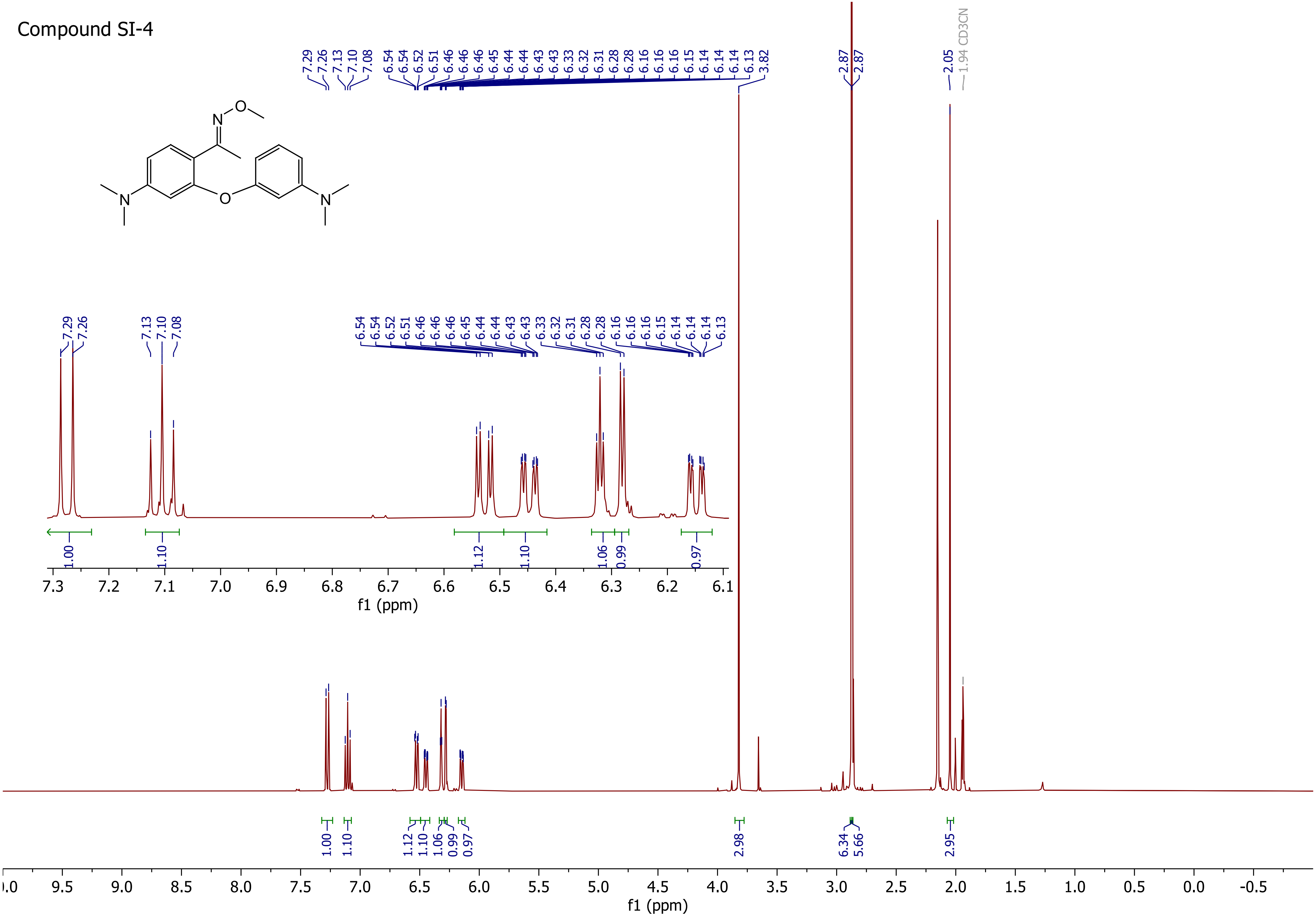

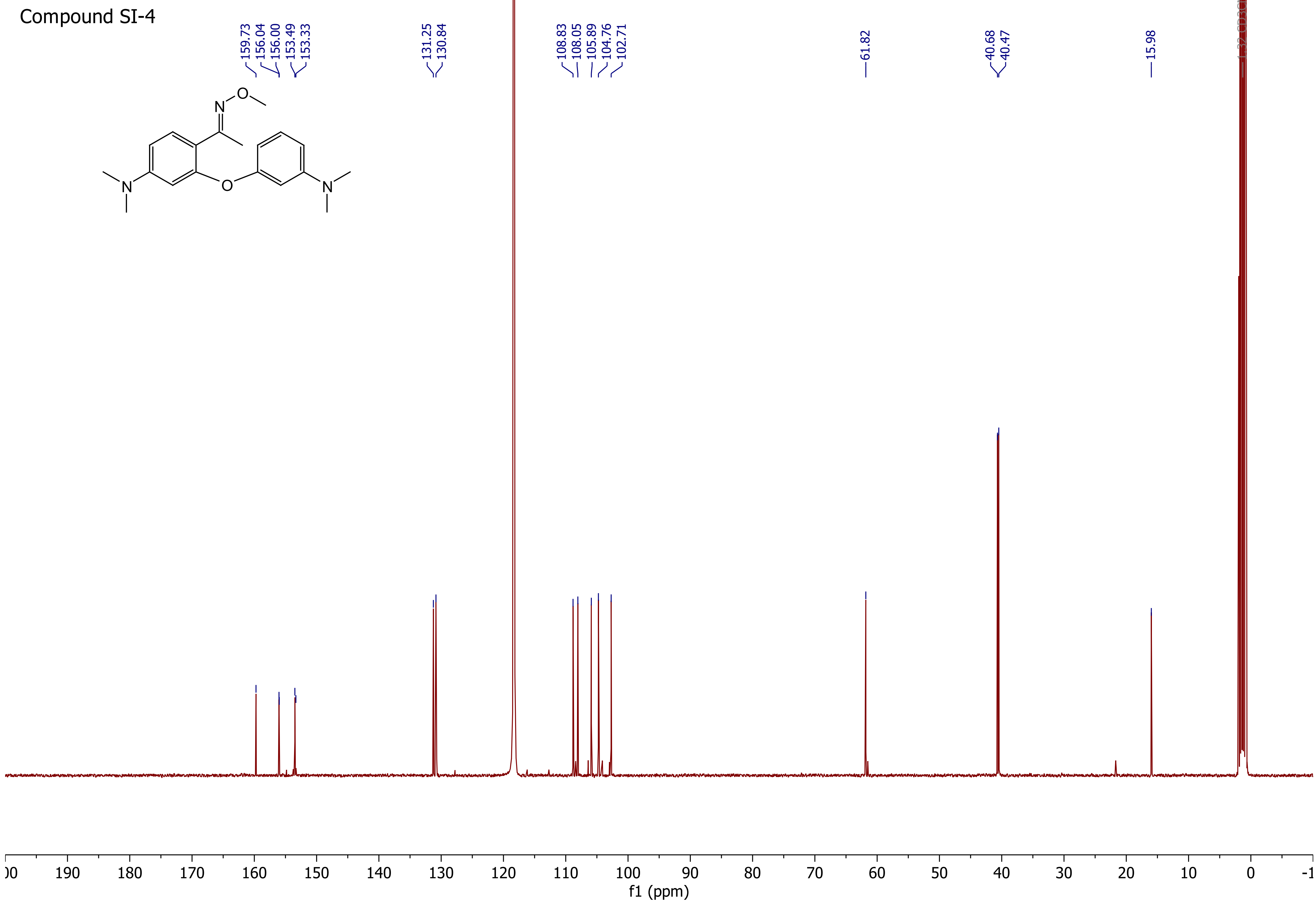

Compound SI-4
159.73
156.04
156.00
153.49
153.33
131.25
130.84
108.83
108.05
105.89
104.76
102.71
61.82
40.68
40.47
15.98
1.32 CD3CN
N
O
O
N
N
190
180
170
160
150
140
130
120
110
100
90
80
70
60
50
40
30
20
10
0
f1 (ppm)

Compound SI-5

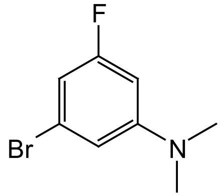

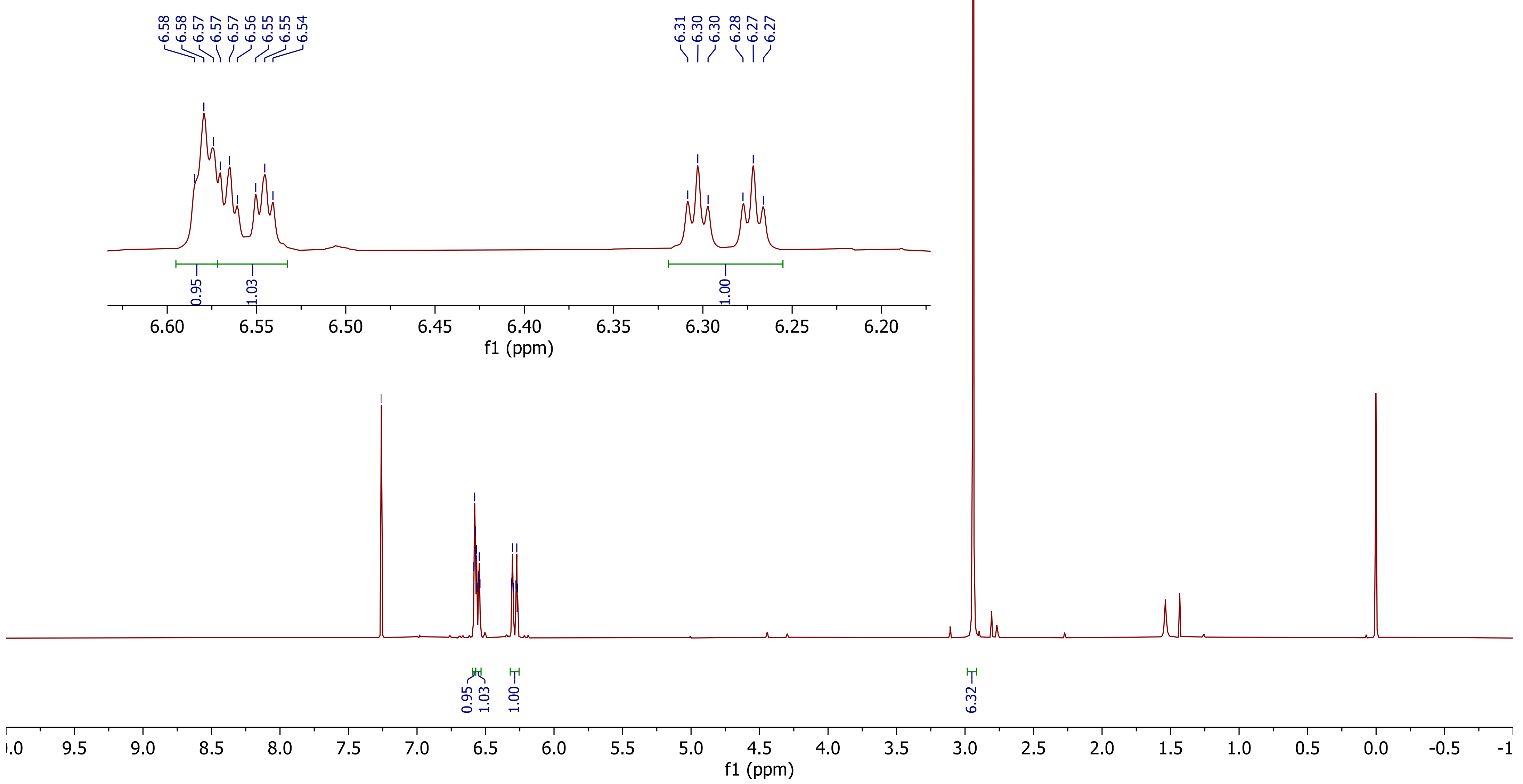

Compound SI-5

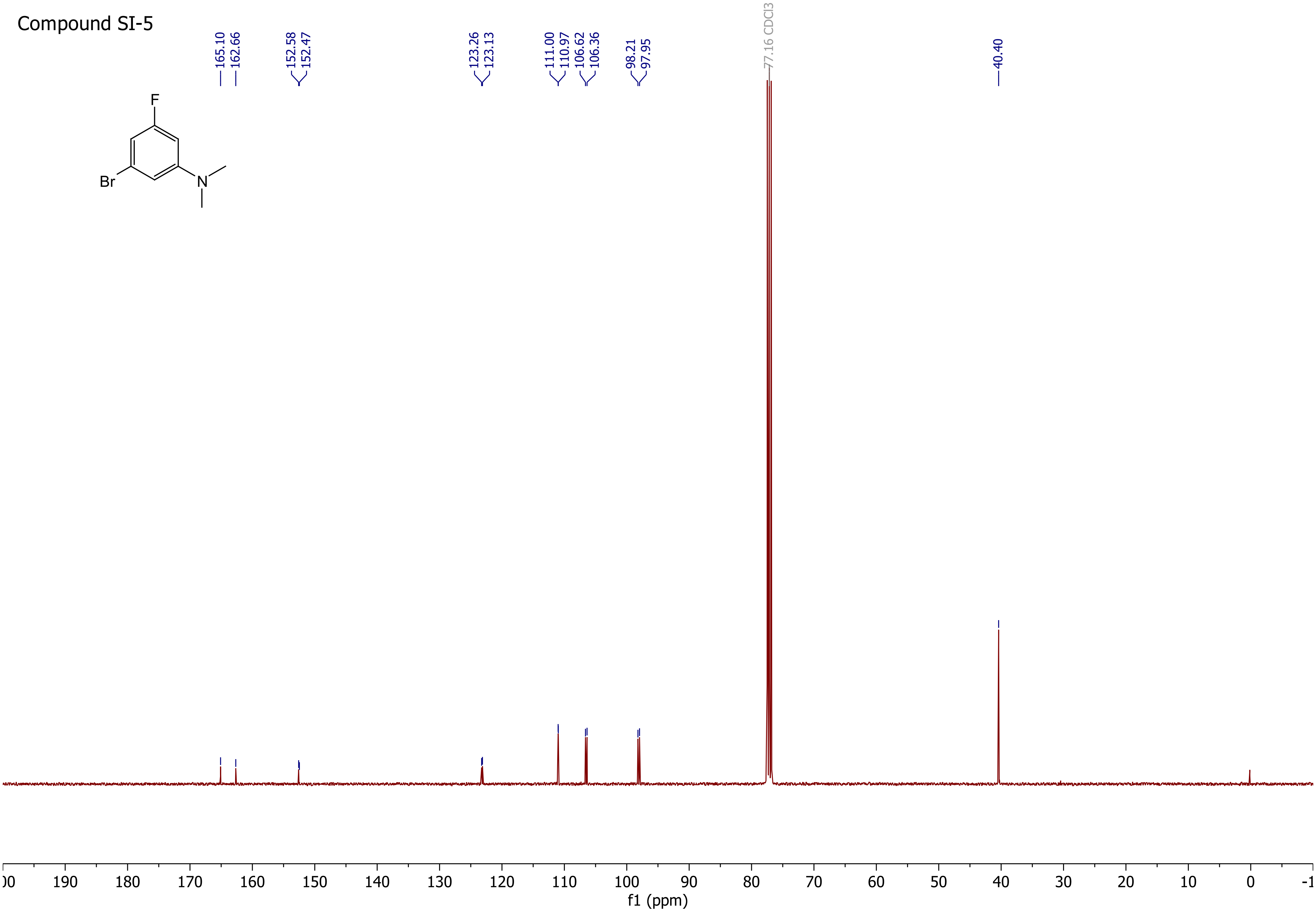

Compound SI-6

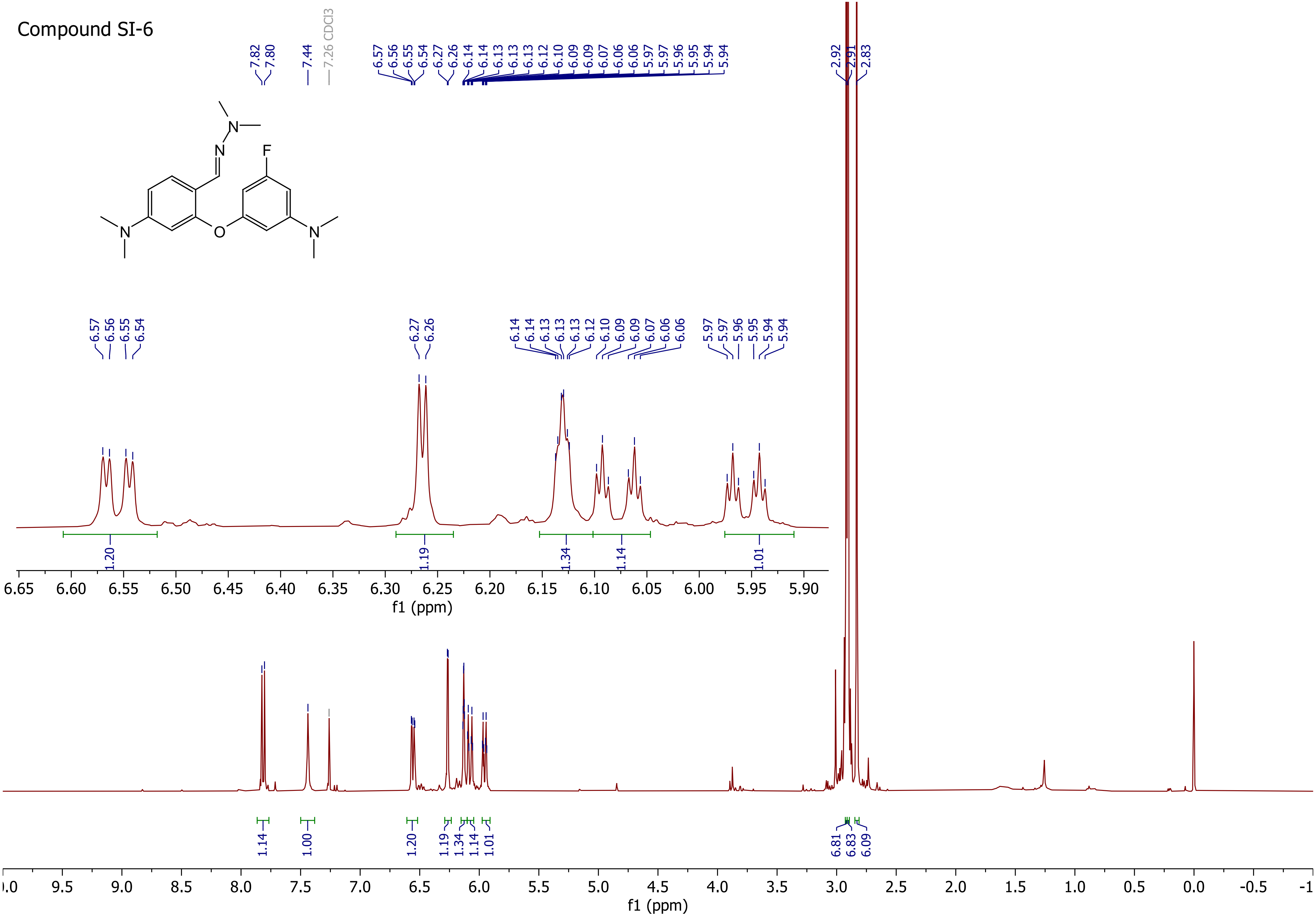

Compound SI-6

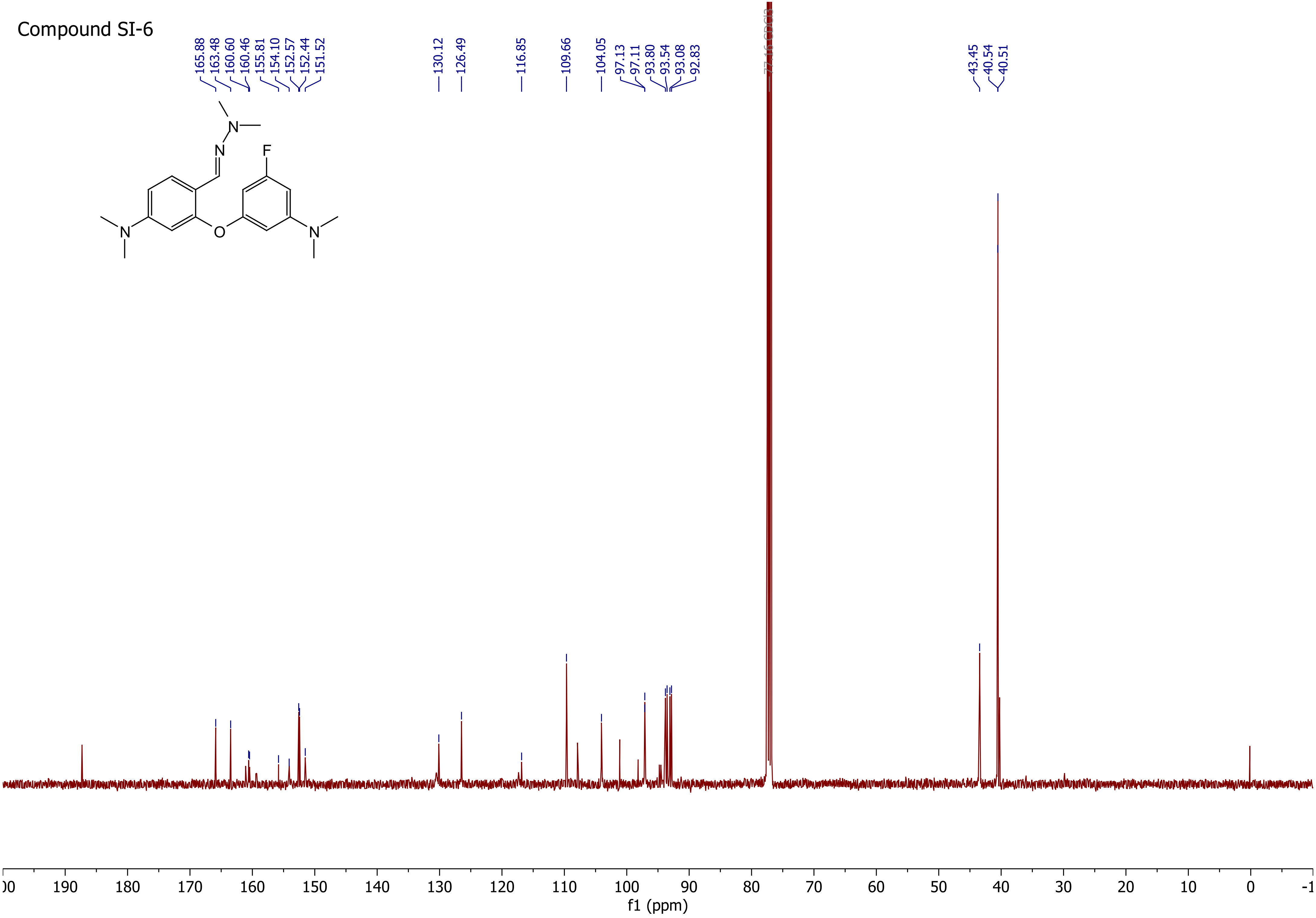

Compound SI-7

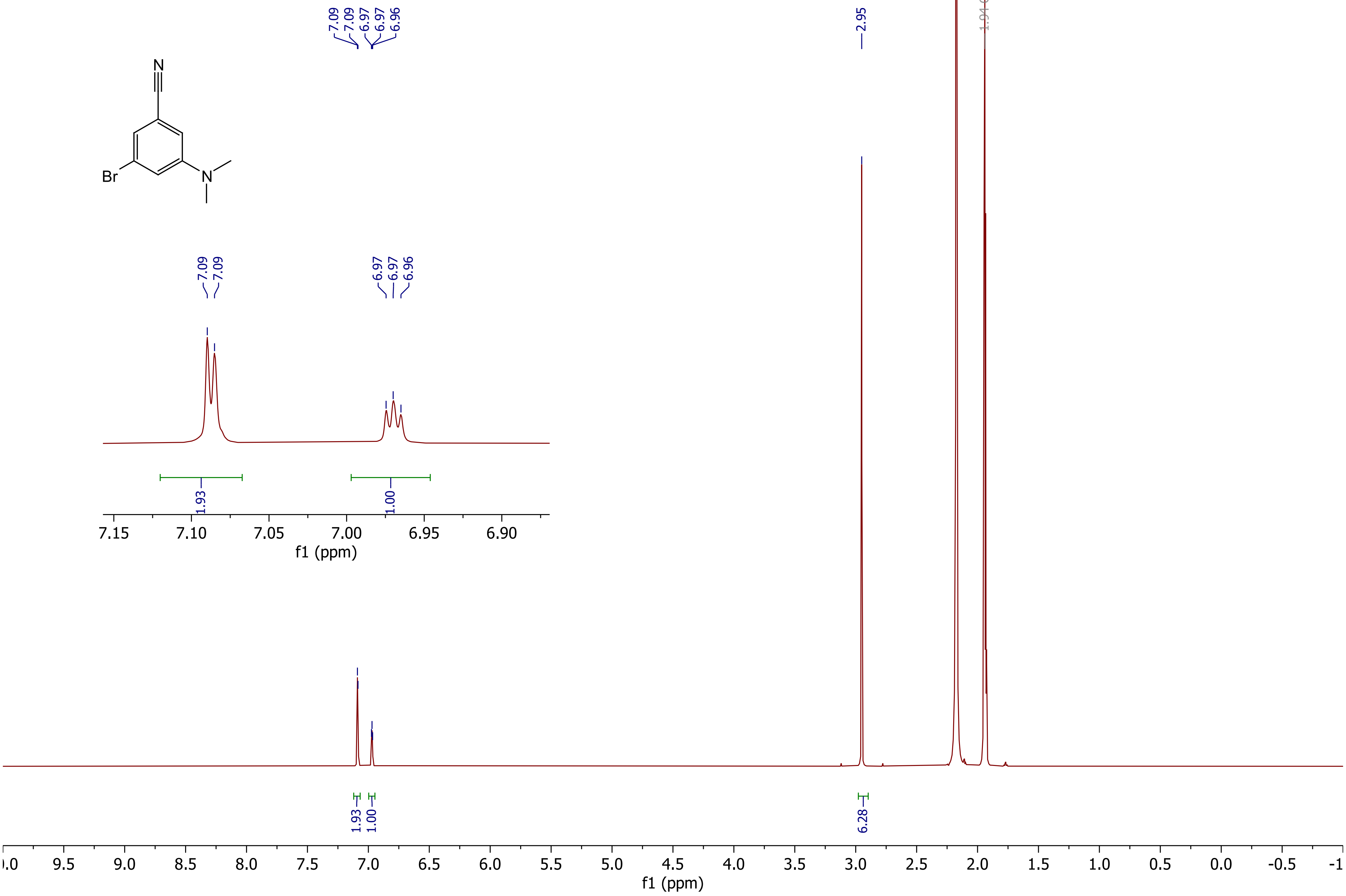

Compound SI-7

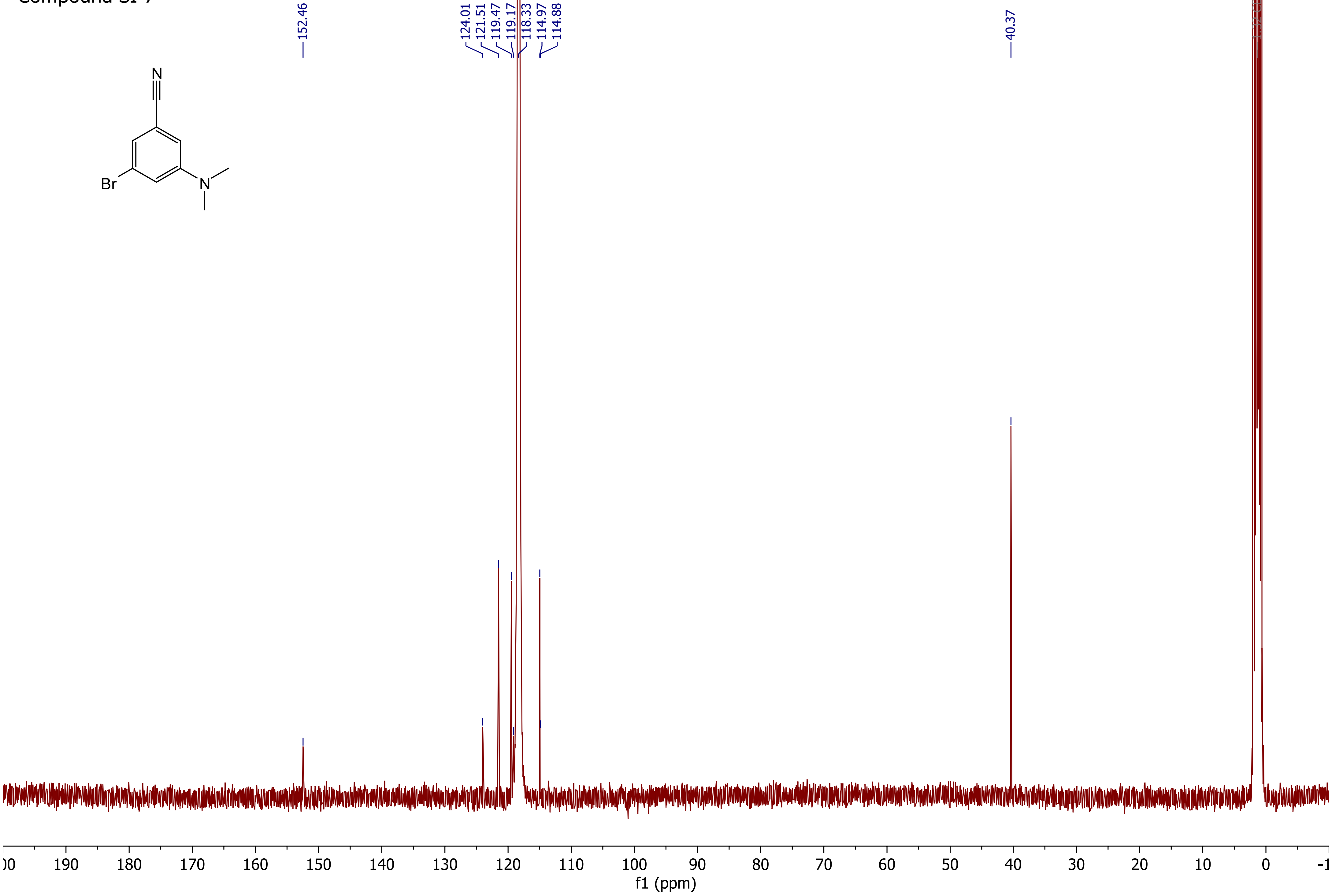

Compound SI-8

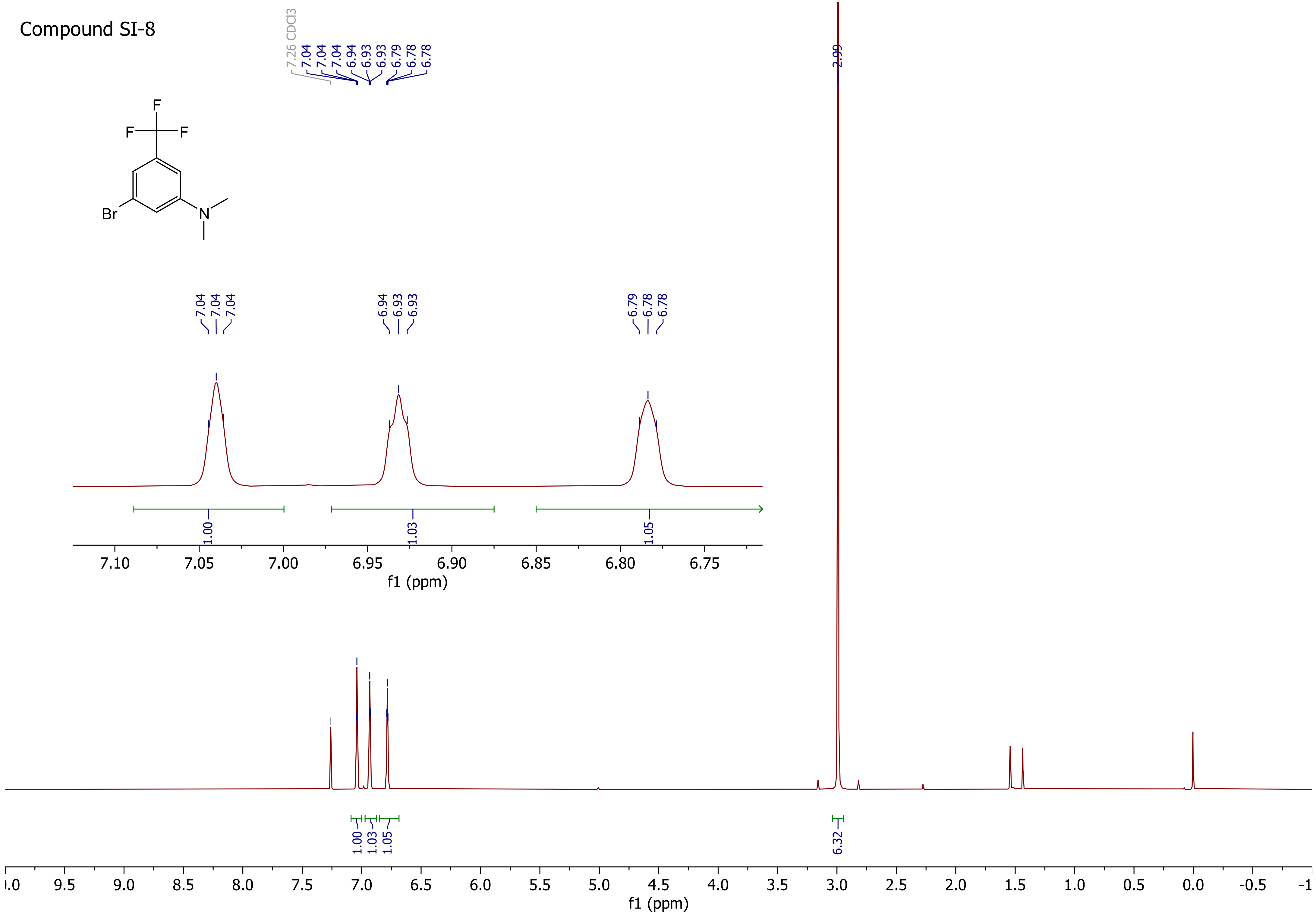

Compound SI-8

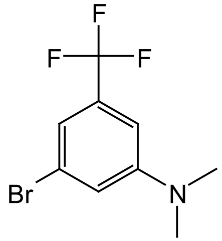

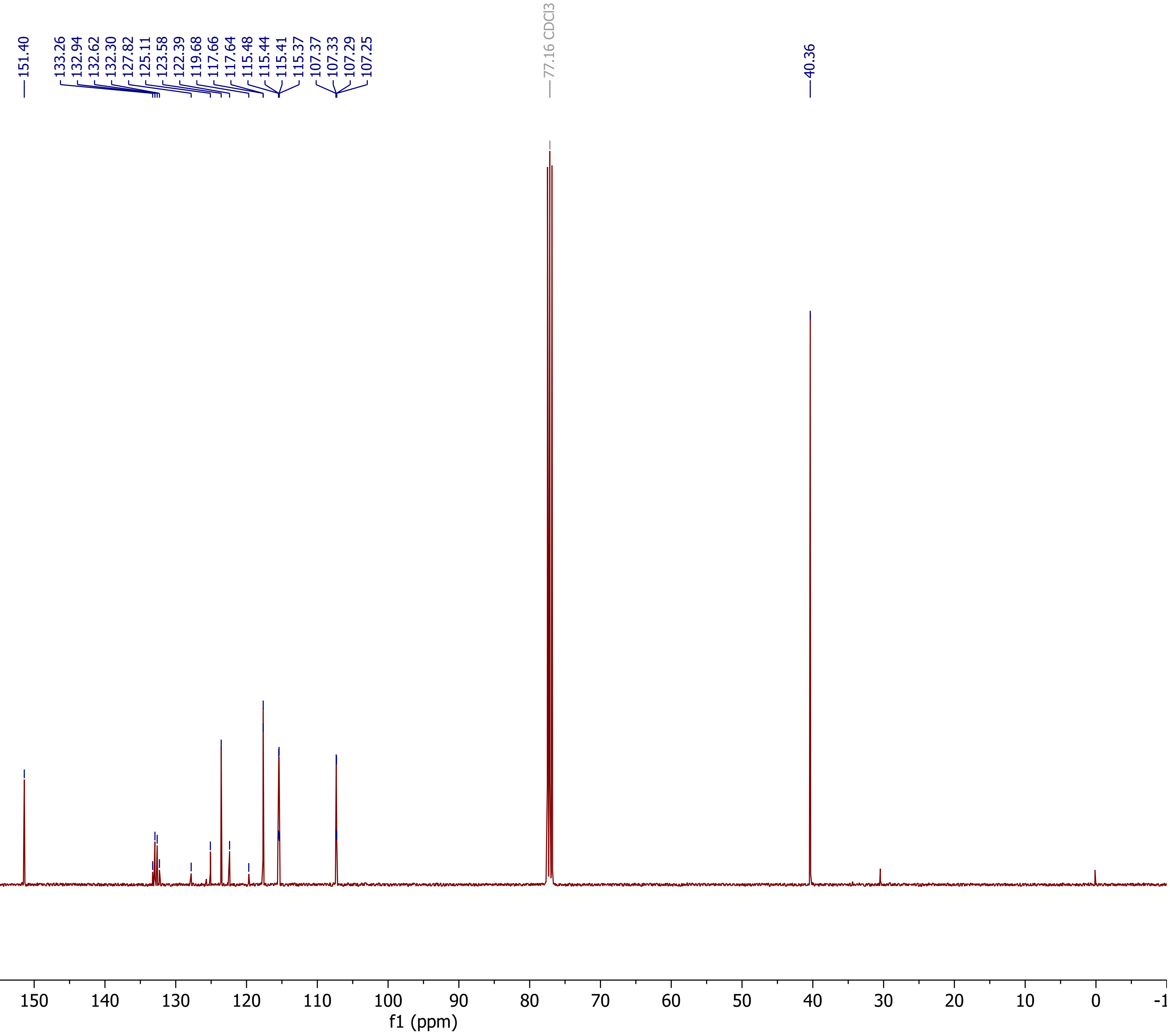

Compound SI-9

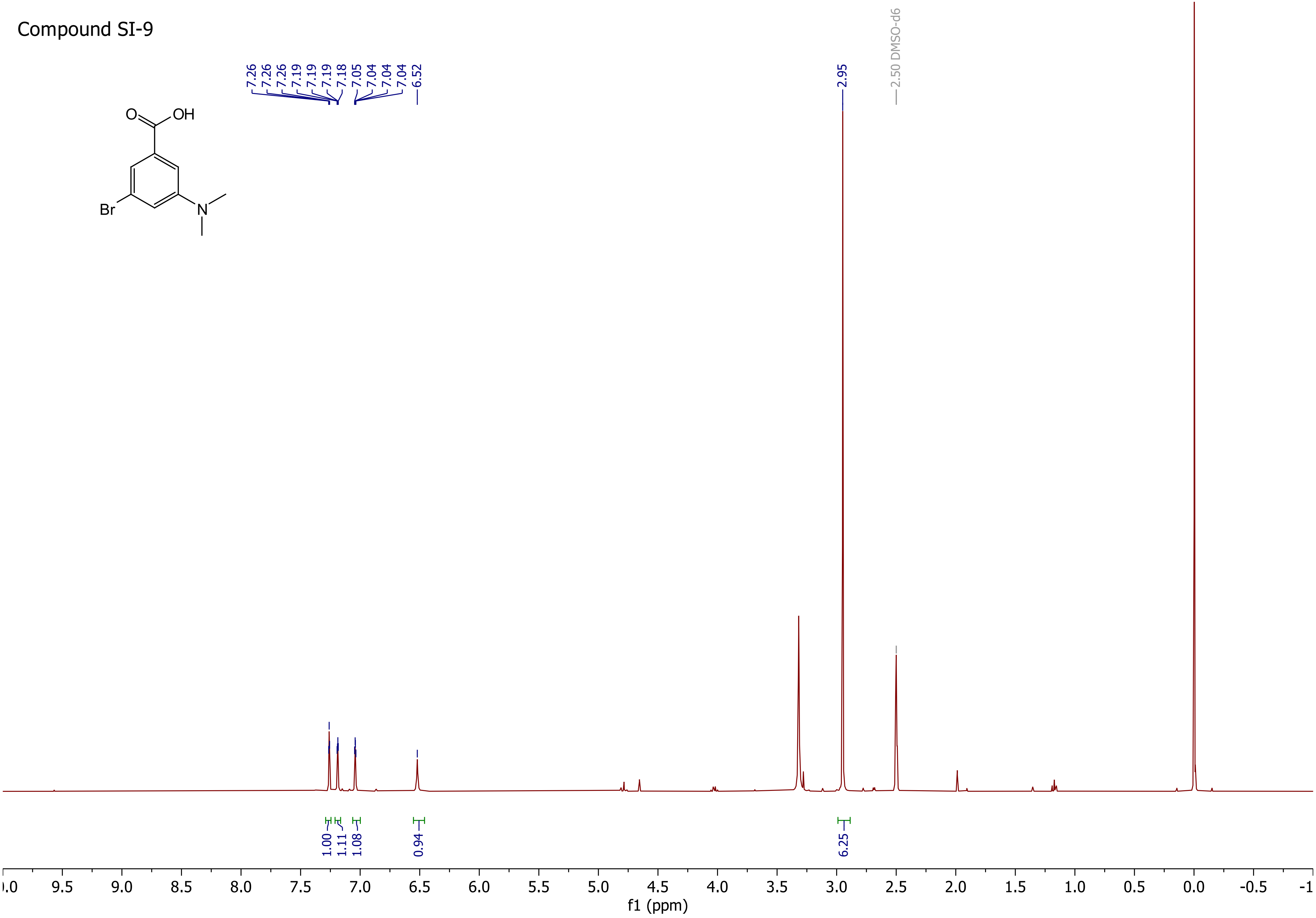

Compound SI-9

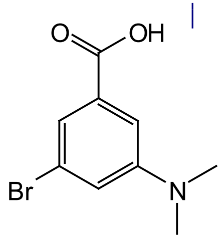

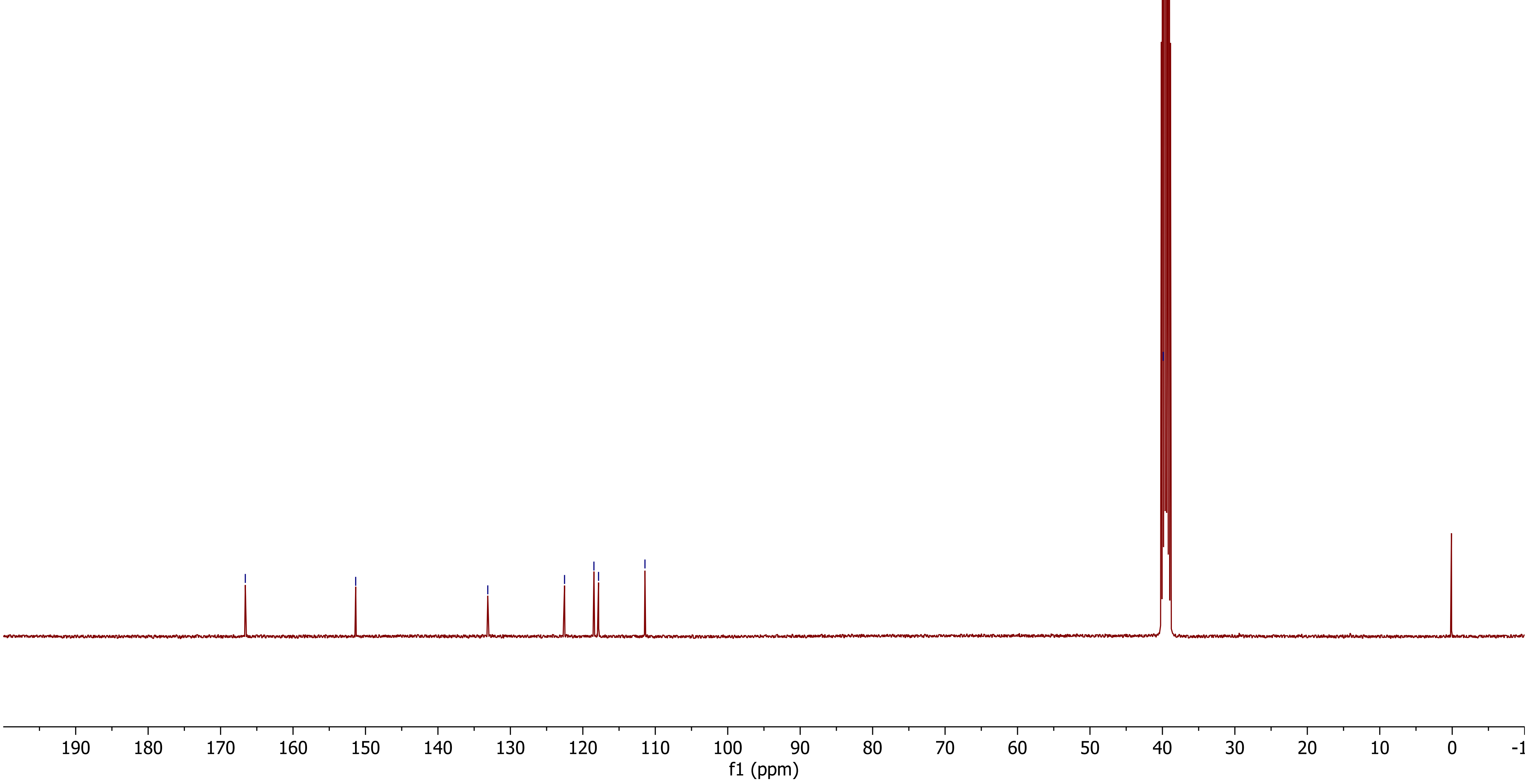

Compound SI-10

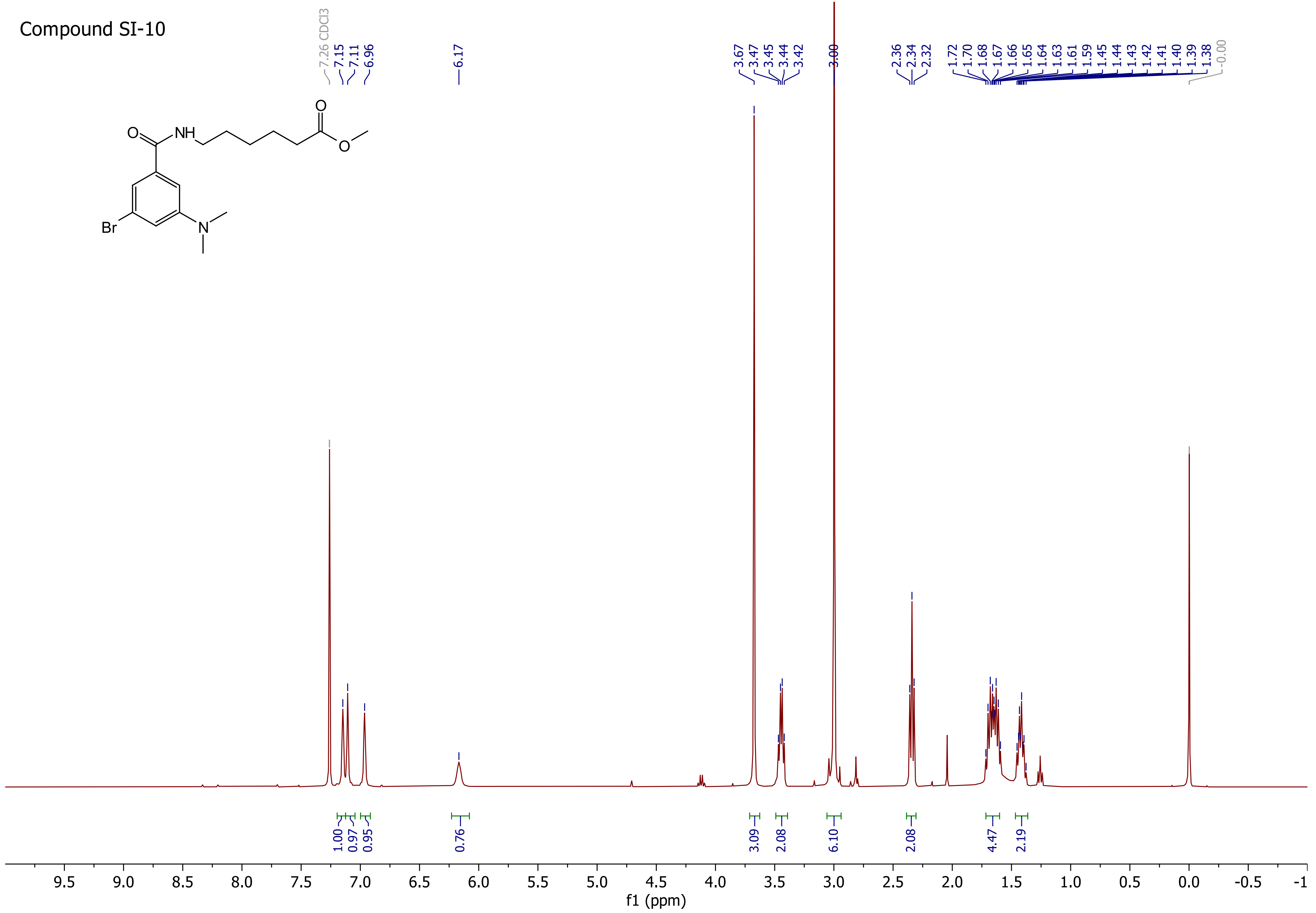

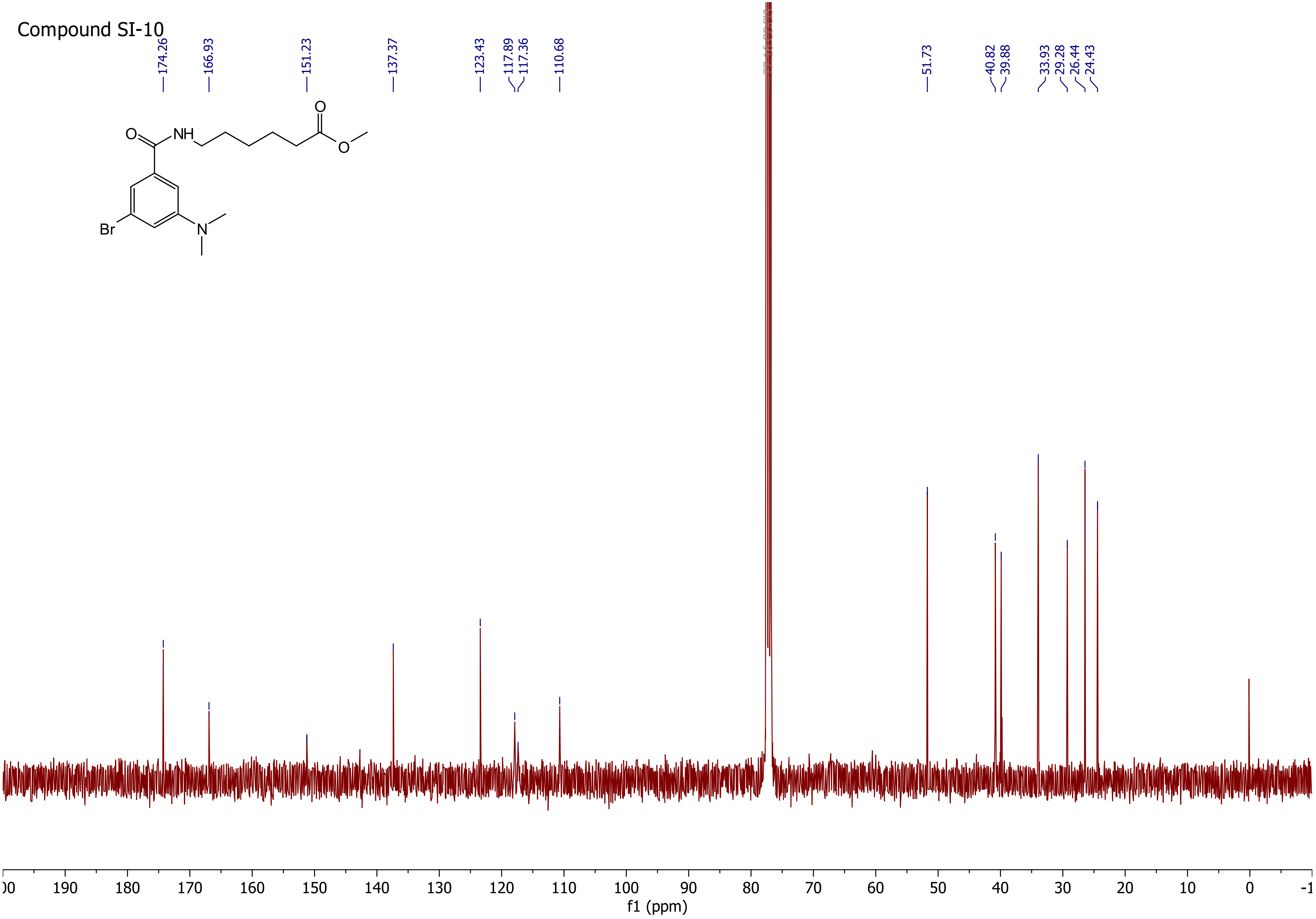

Compound SI-10
174.26
166.93
151.23
137.37
123.43
117.89
117.36
110.68
51.73
40.82
39.88
33.93
29.28
26.44
24.43
O
NH
O
O
Br
N
f1 (ppm)

Compound SI-11

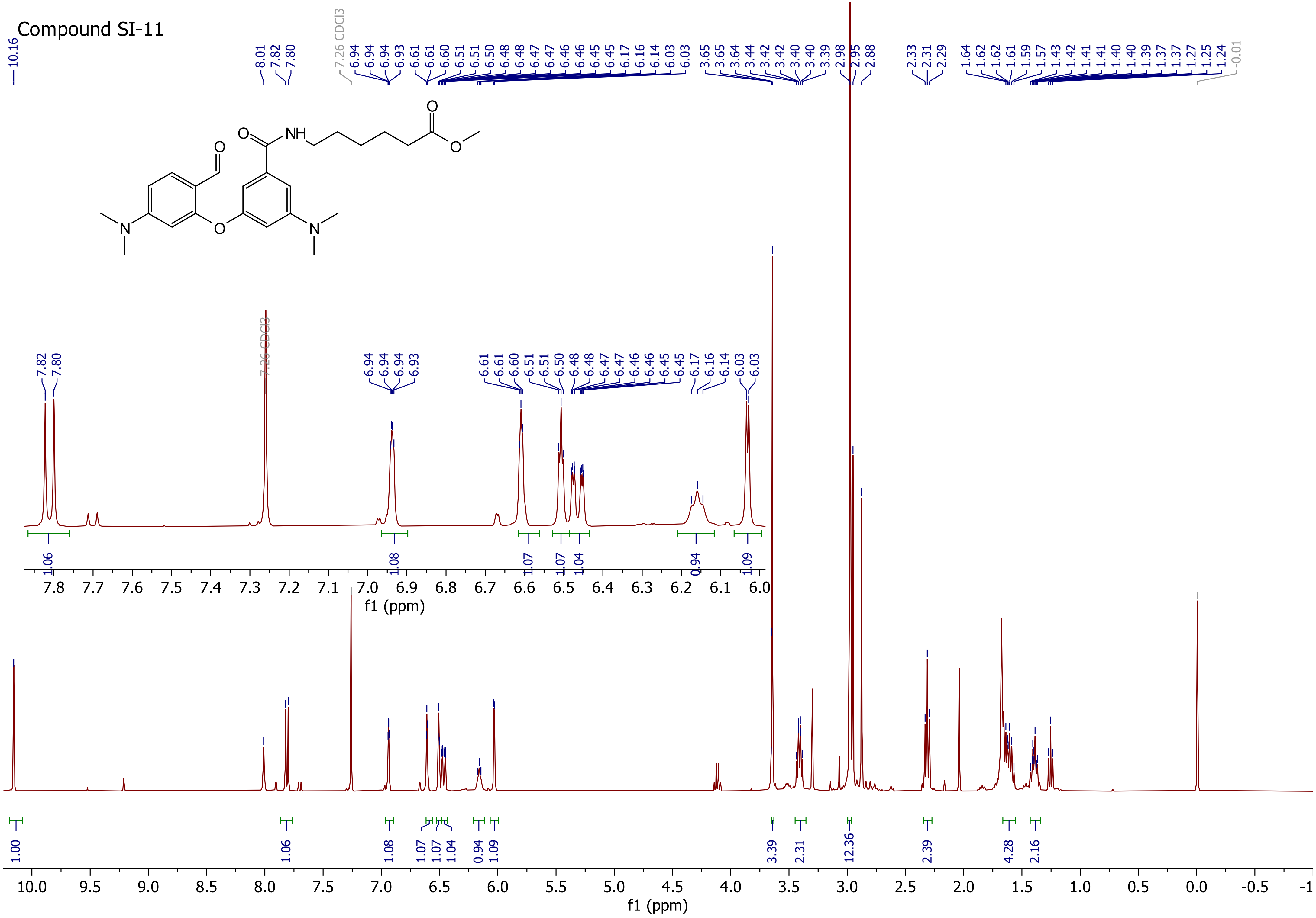

Compound SI-11

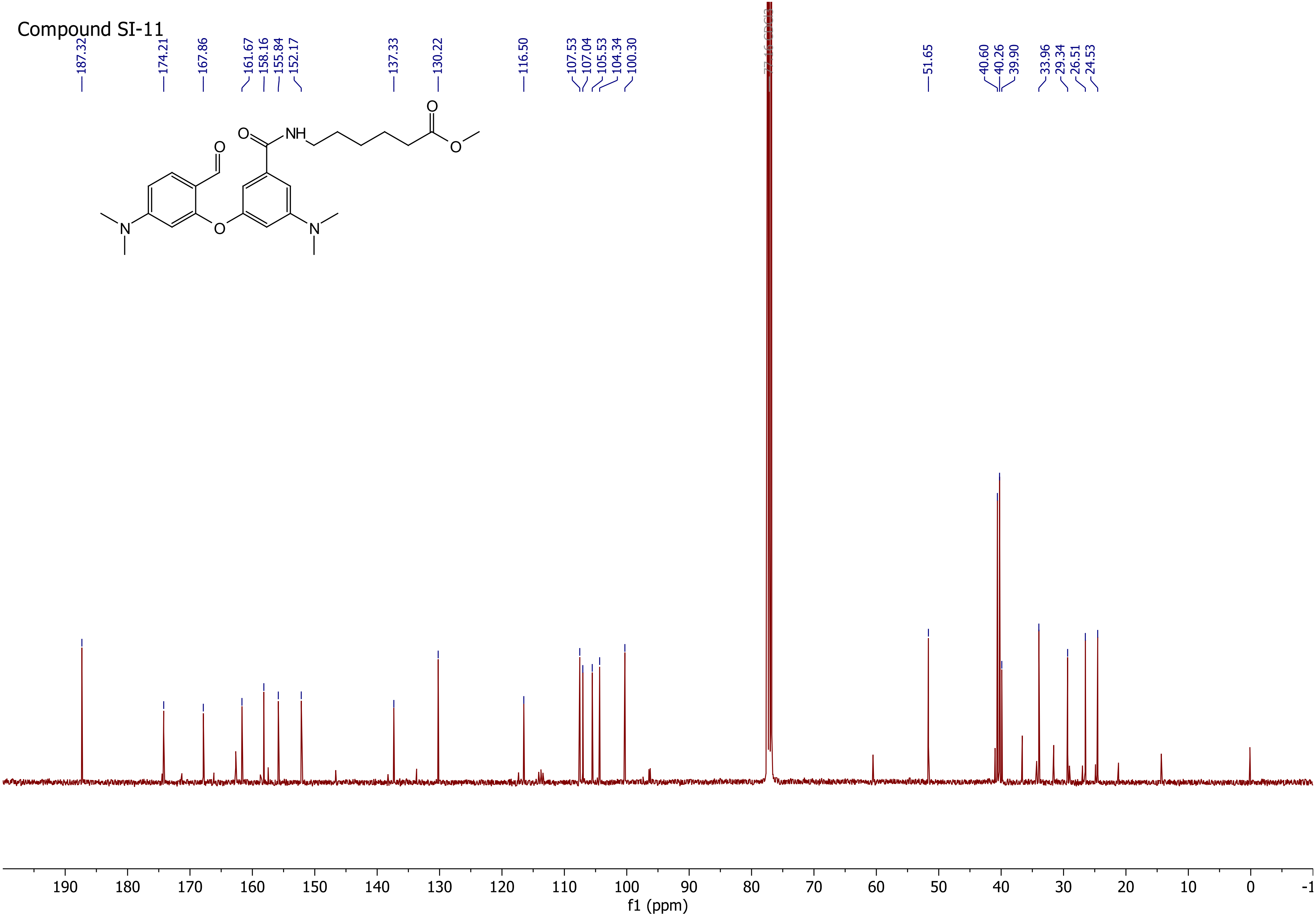

Compound SI-12

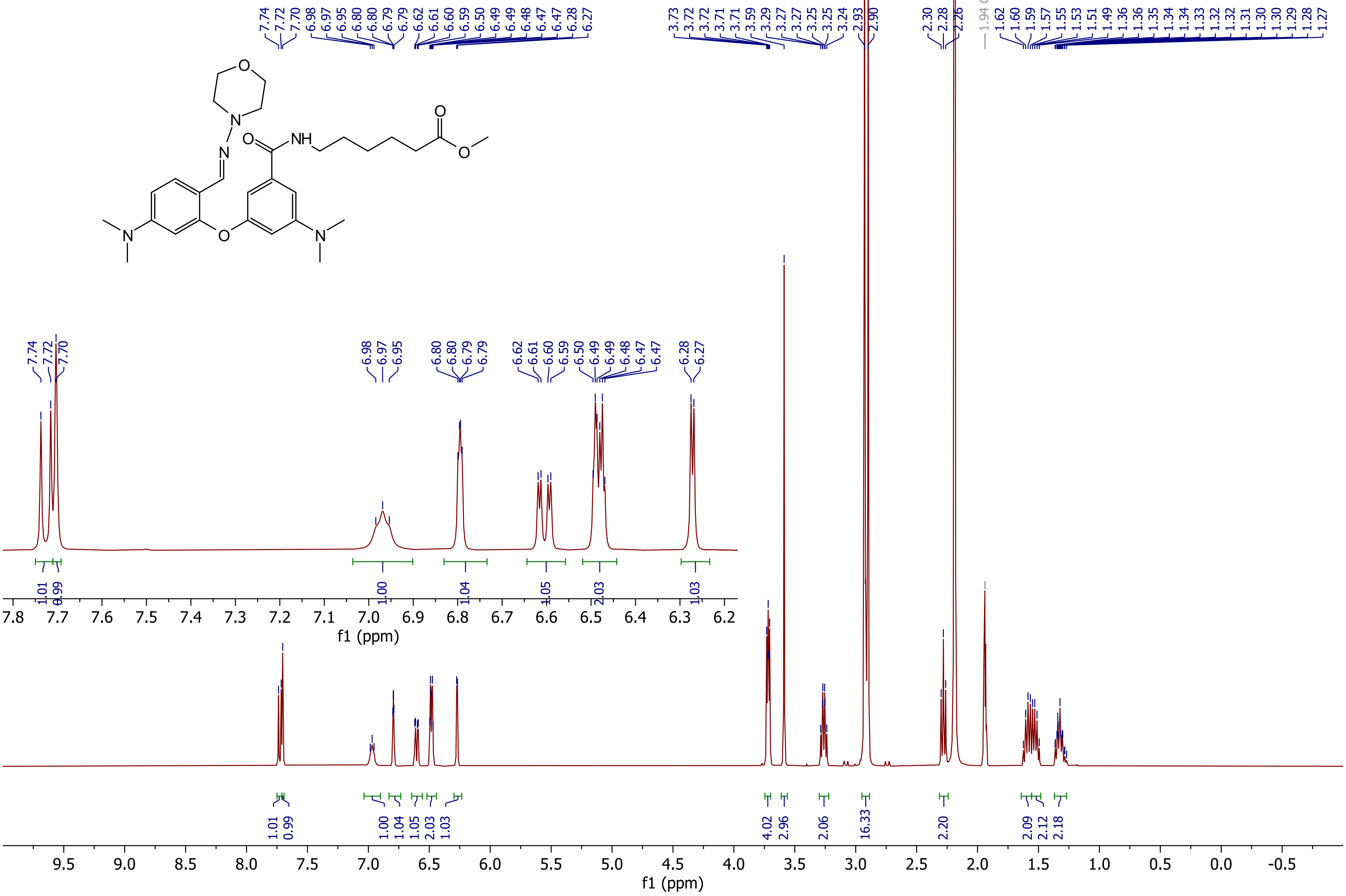

Compound SI-12

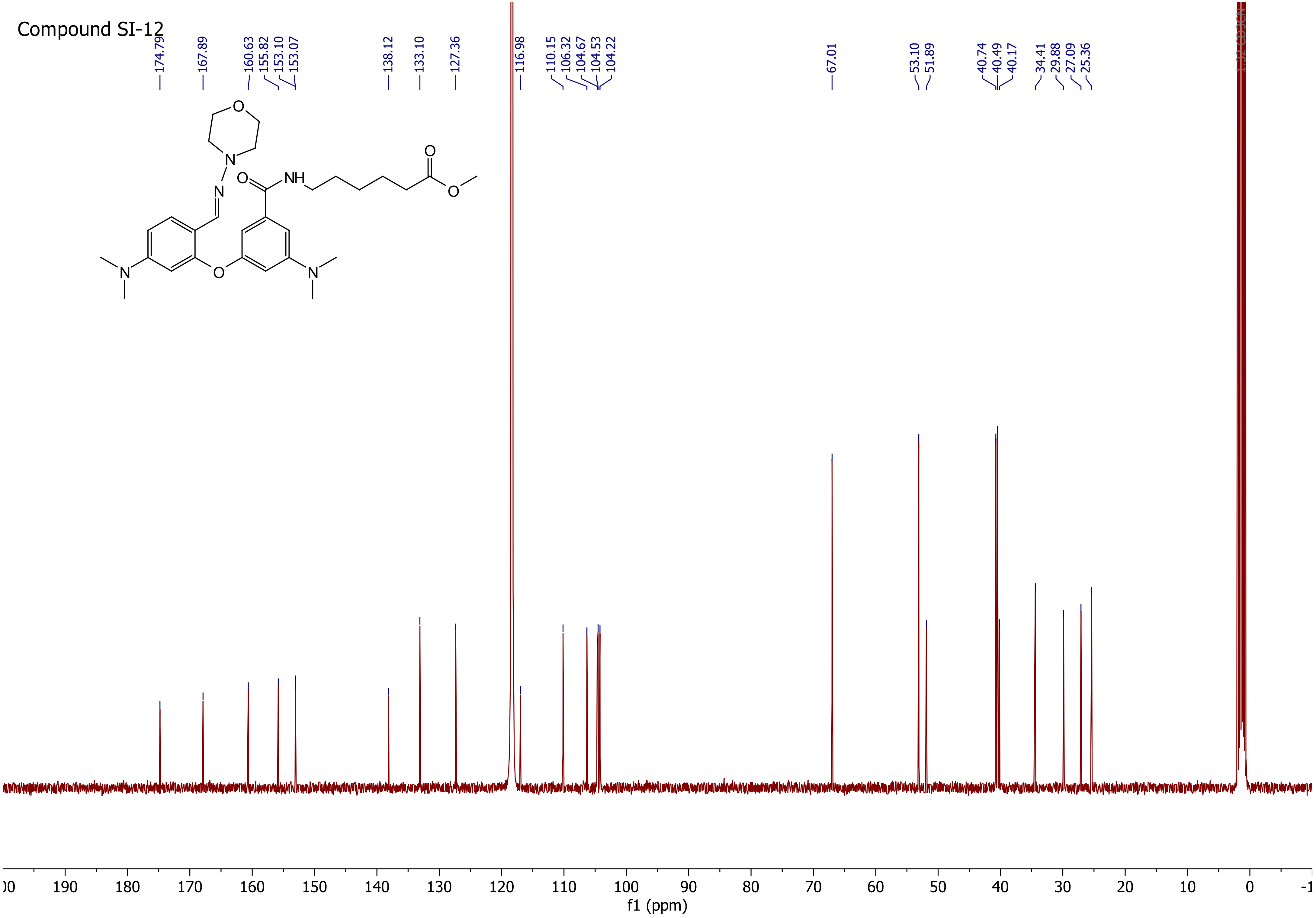

Compound SI-13

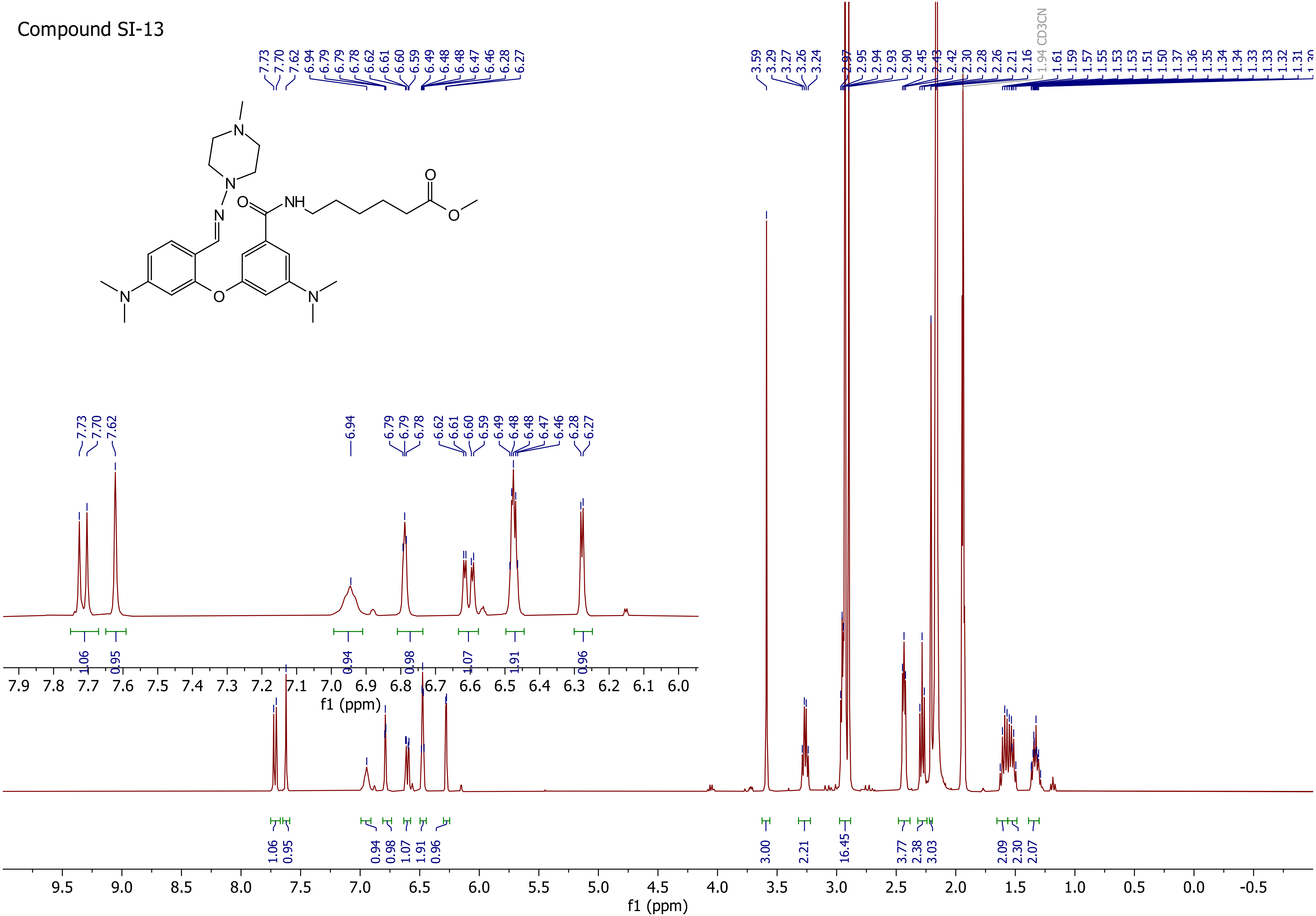

Compound SI-13

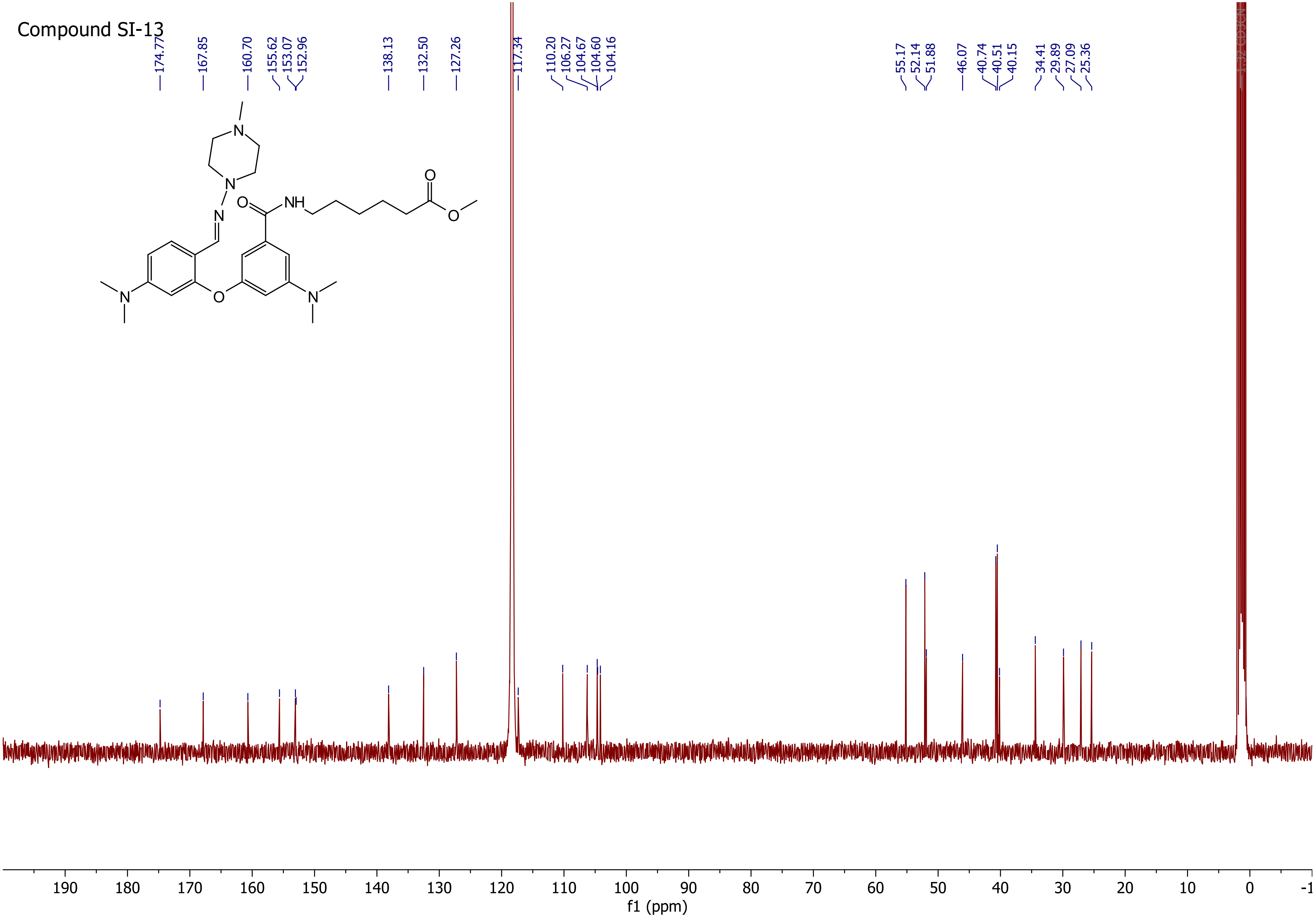

Compound SI-14

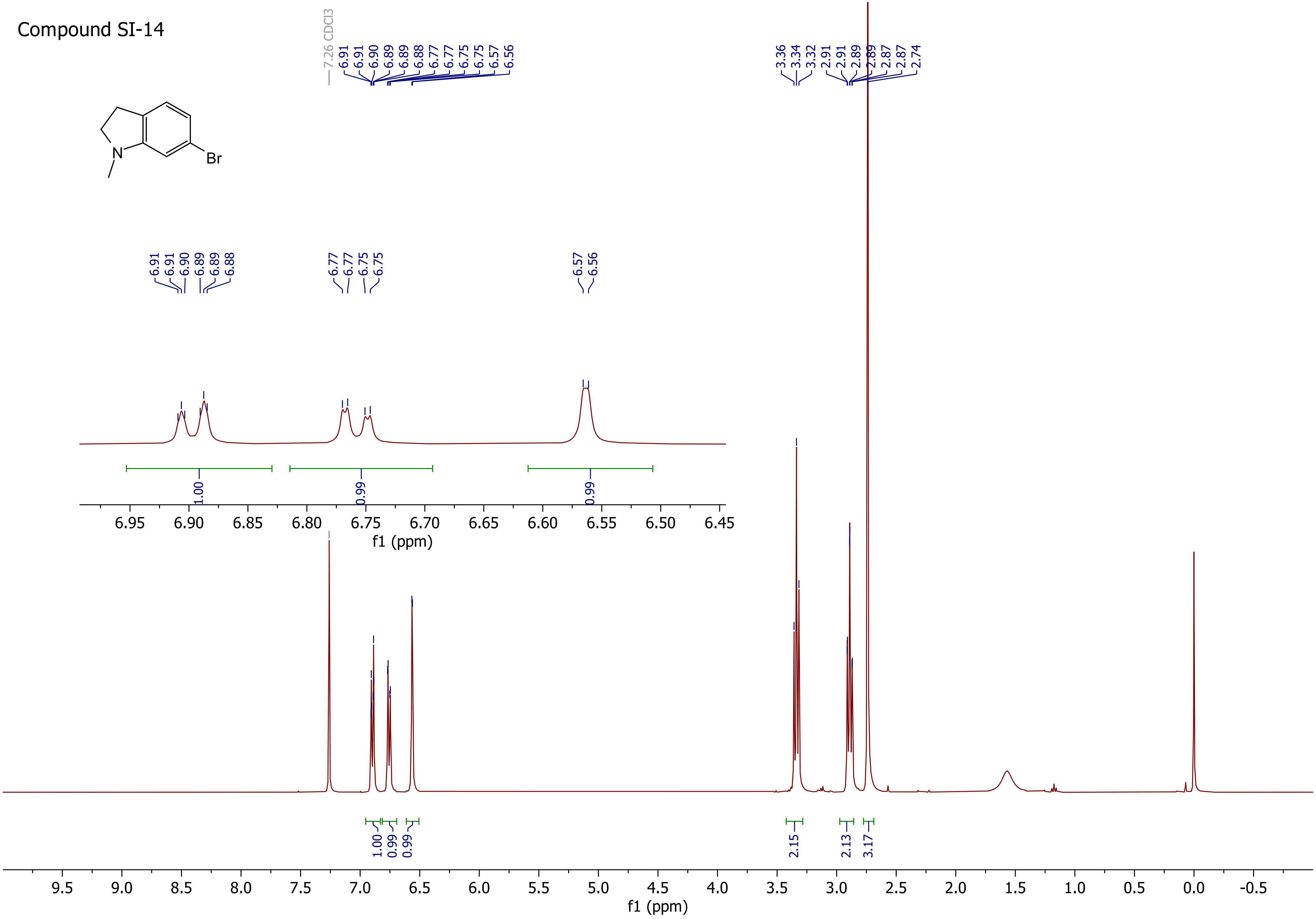

Compound SI-14

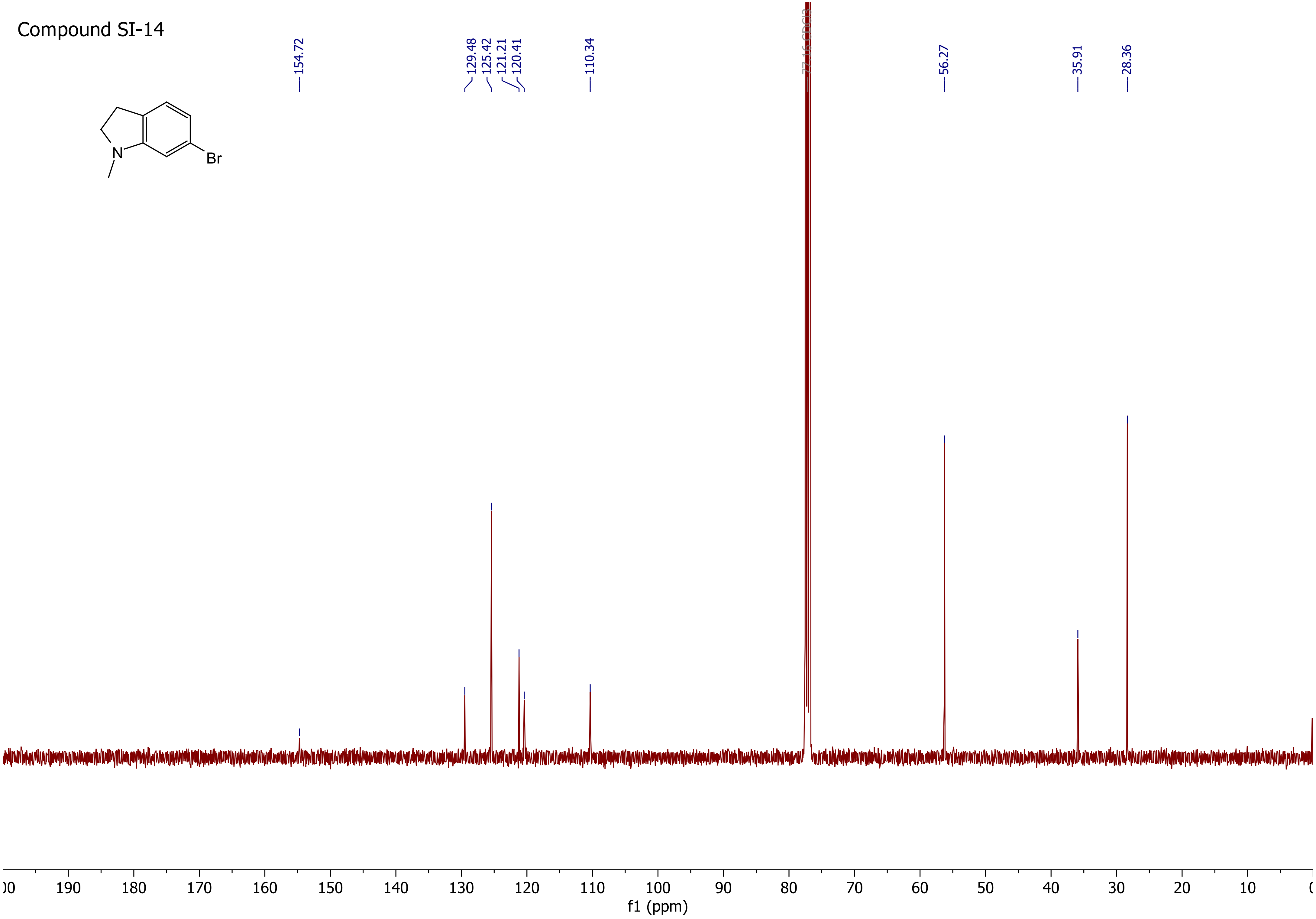

Compound SI-15

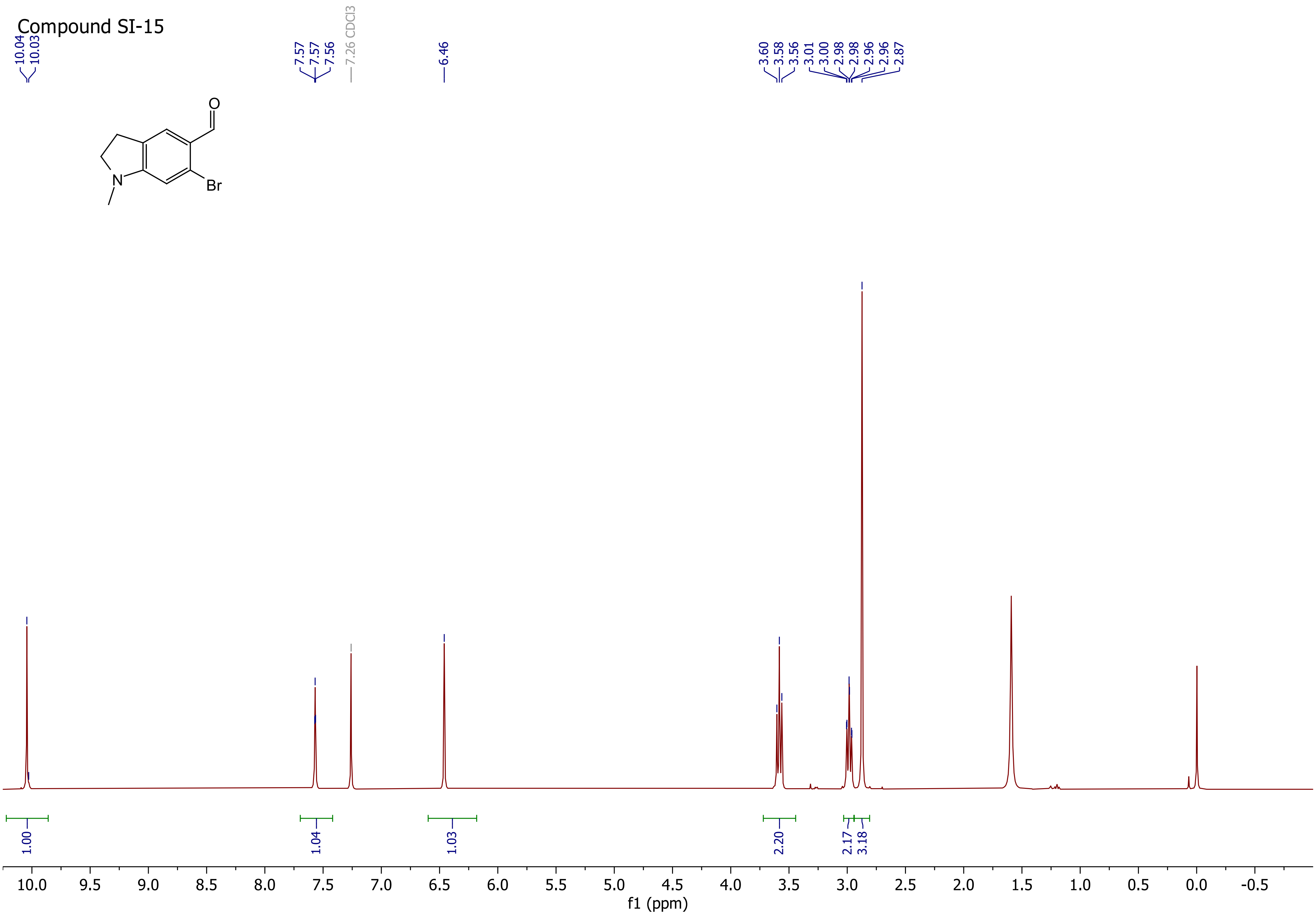

Compound SI-15

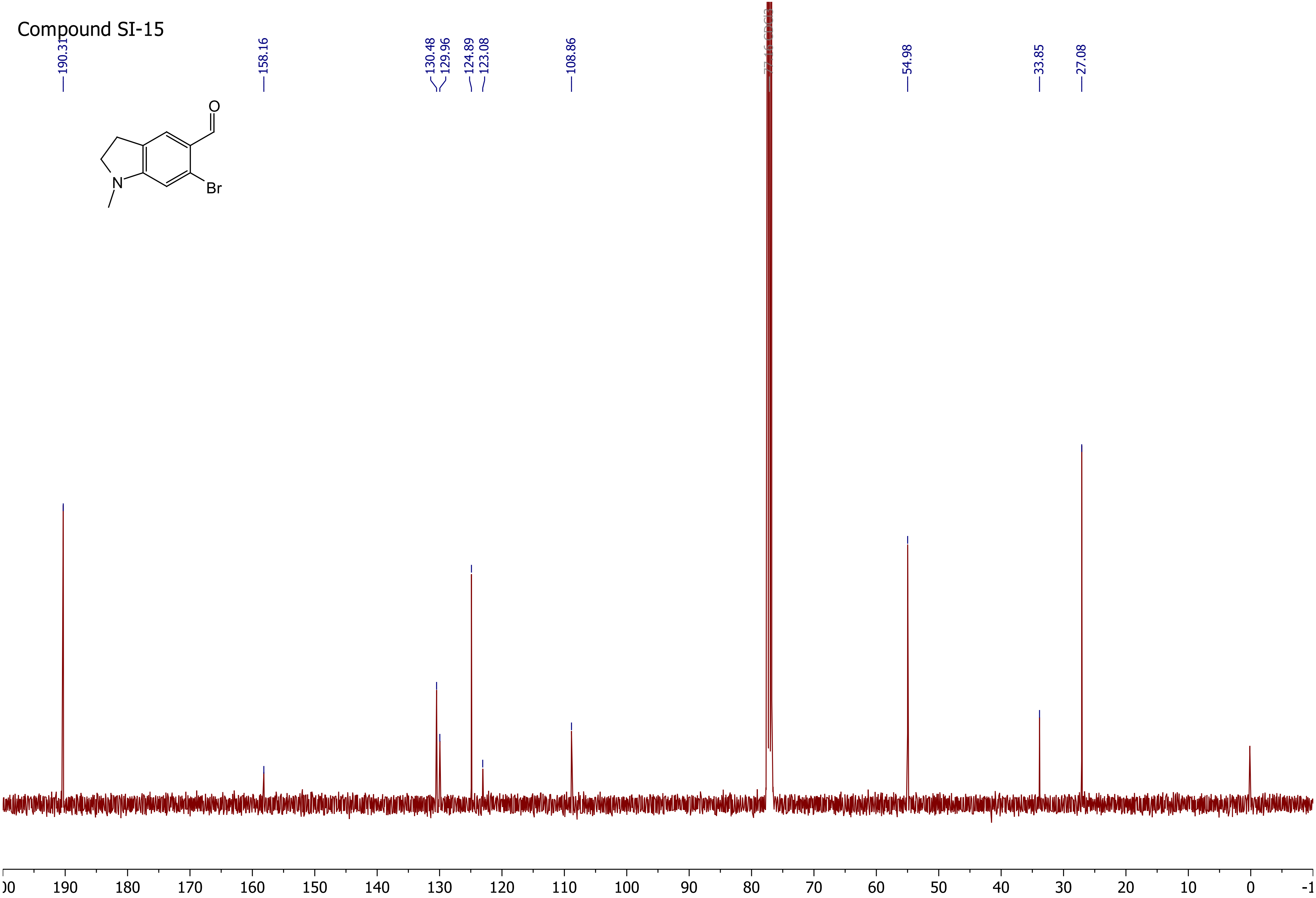

Compound SI-16

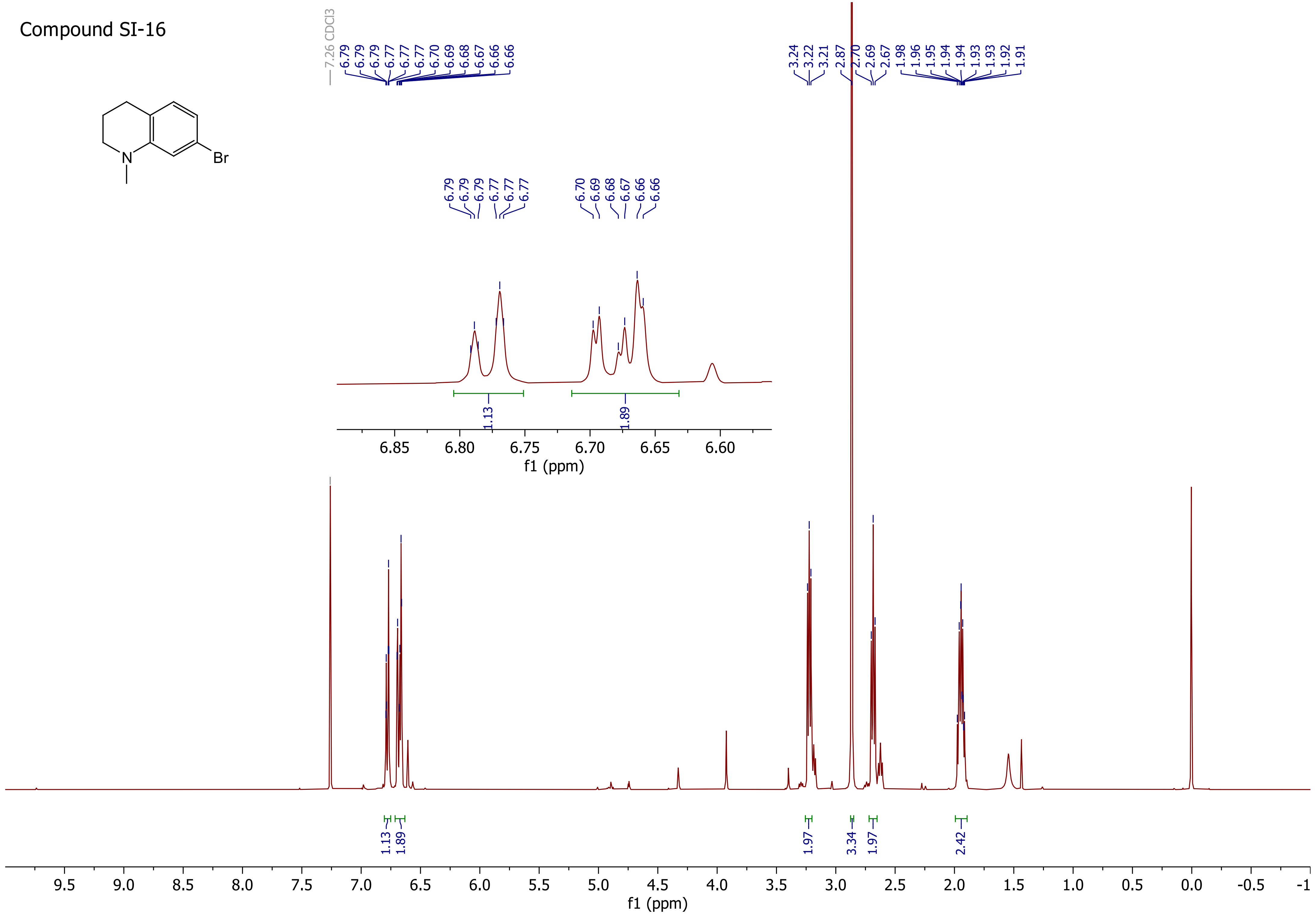

Compound SI-16

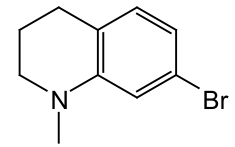

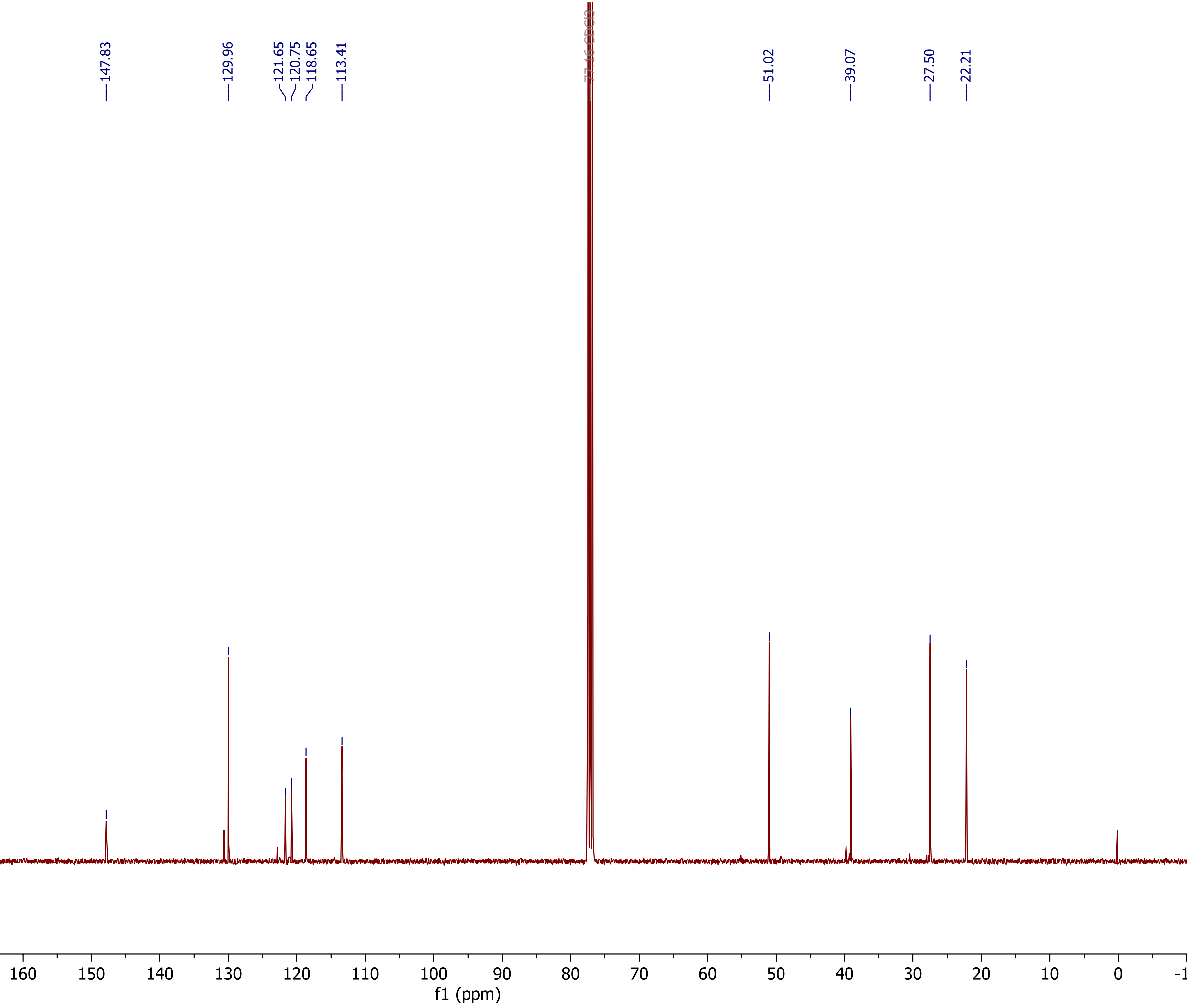

Compound SI-17

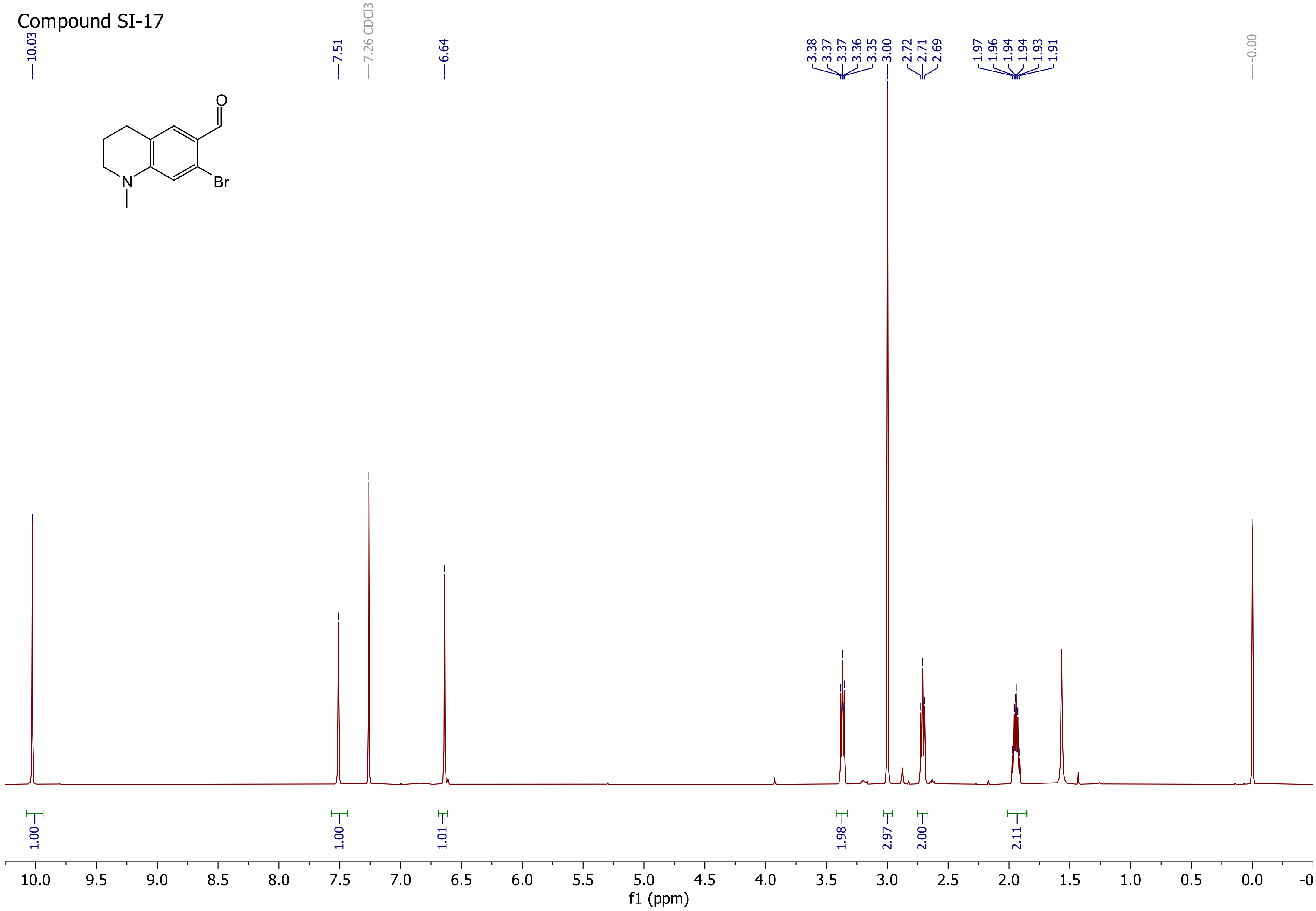

Compound SI-17

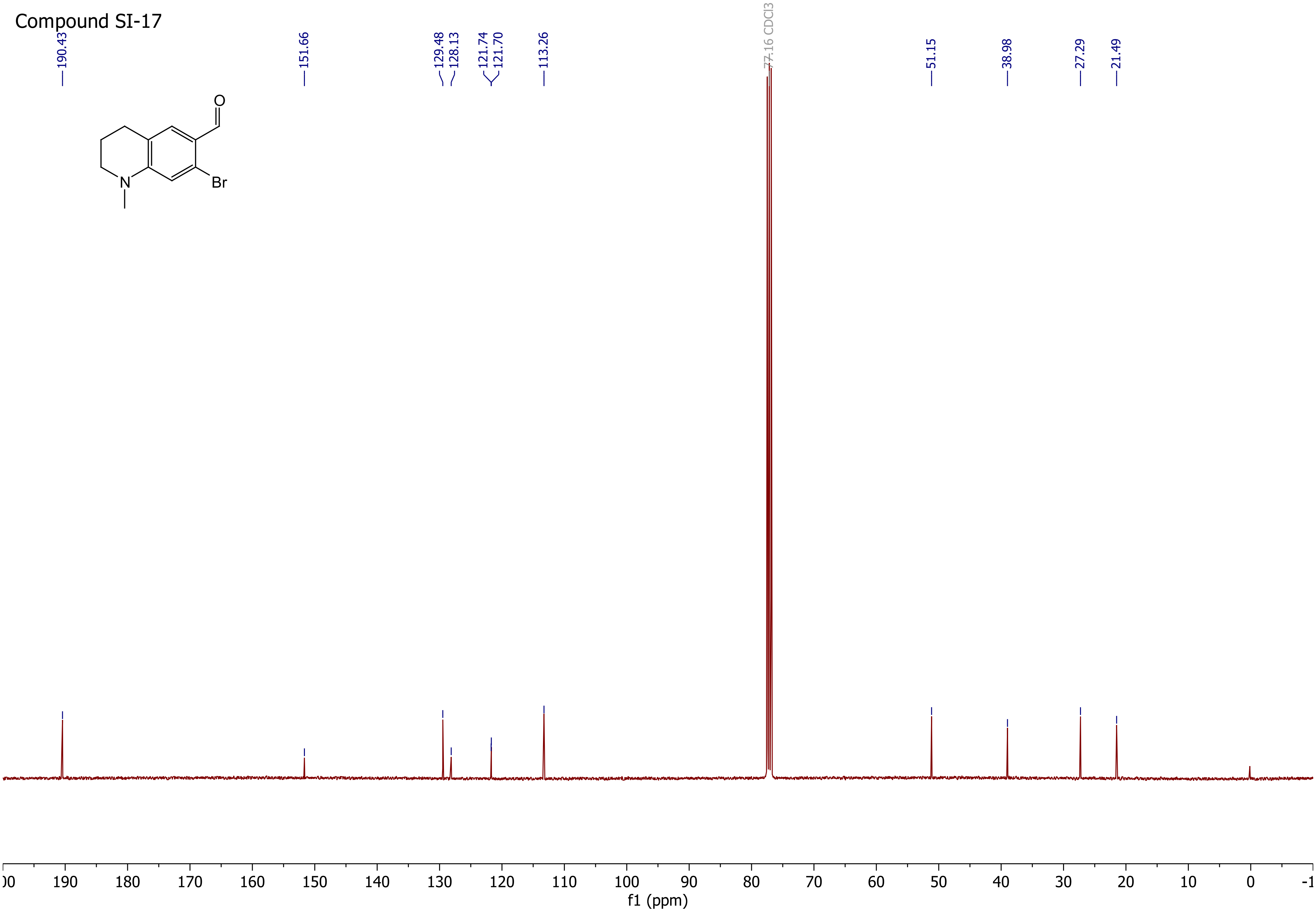

Compound SI-18

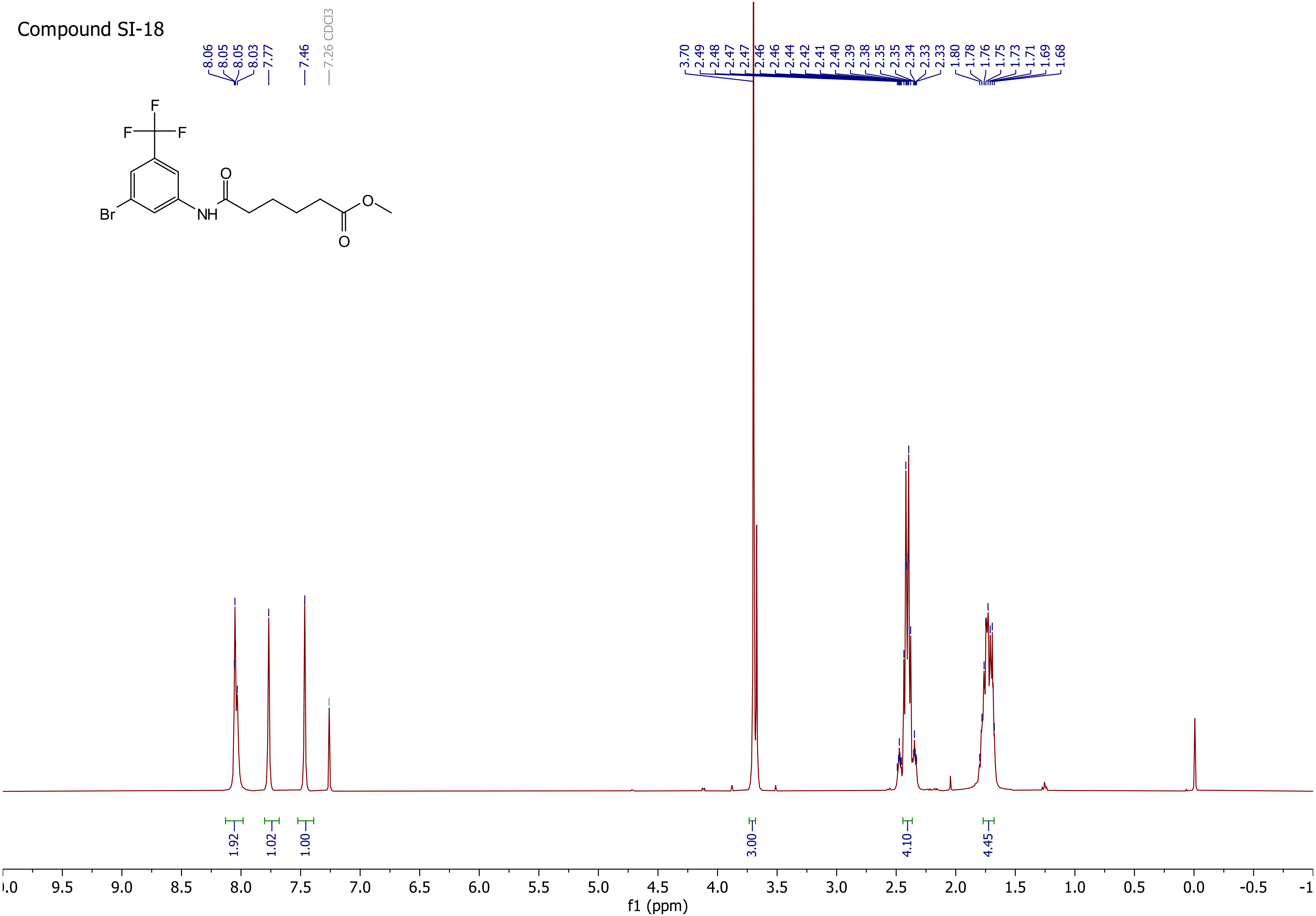

Compound SI-18

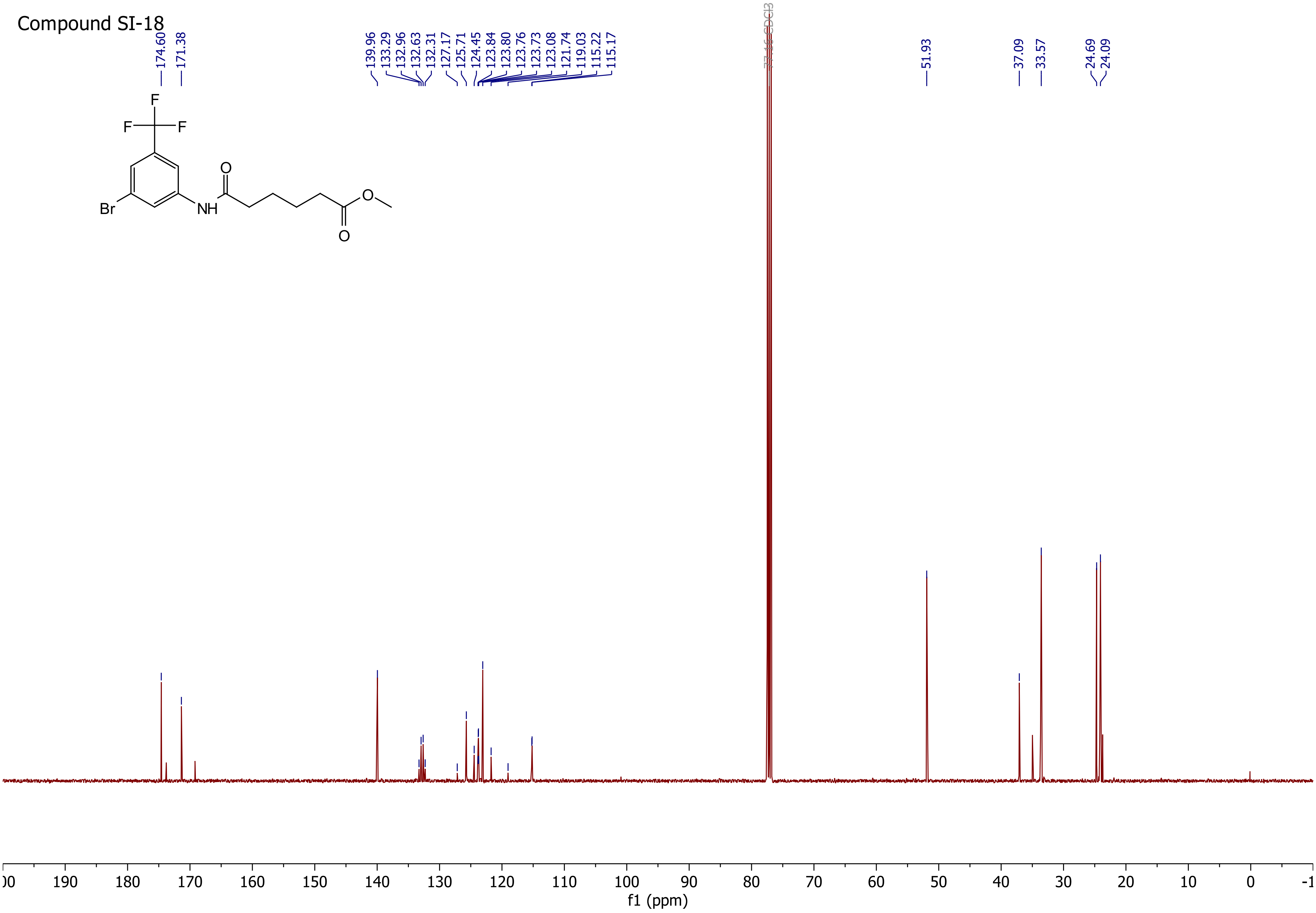

Compound SI-19

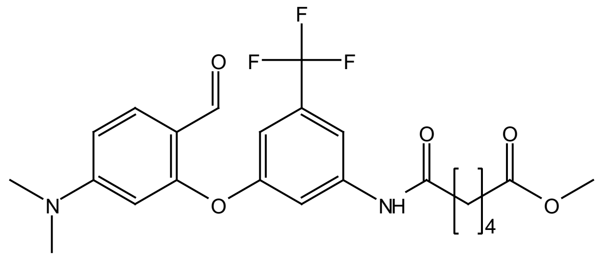

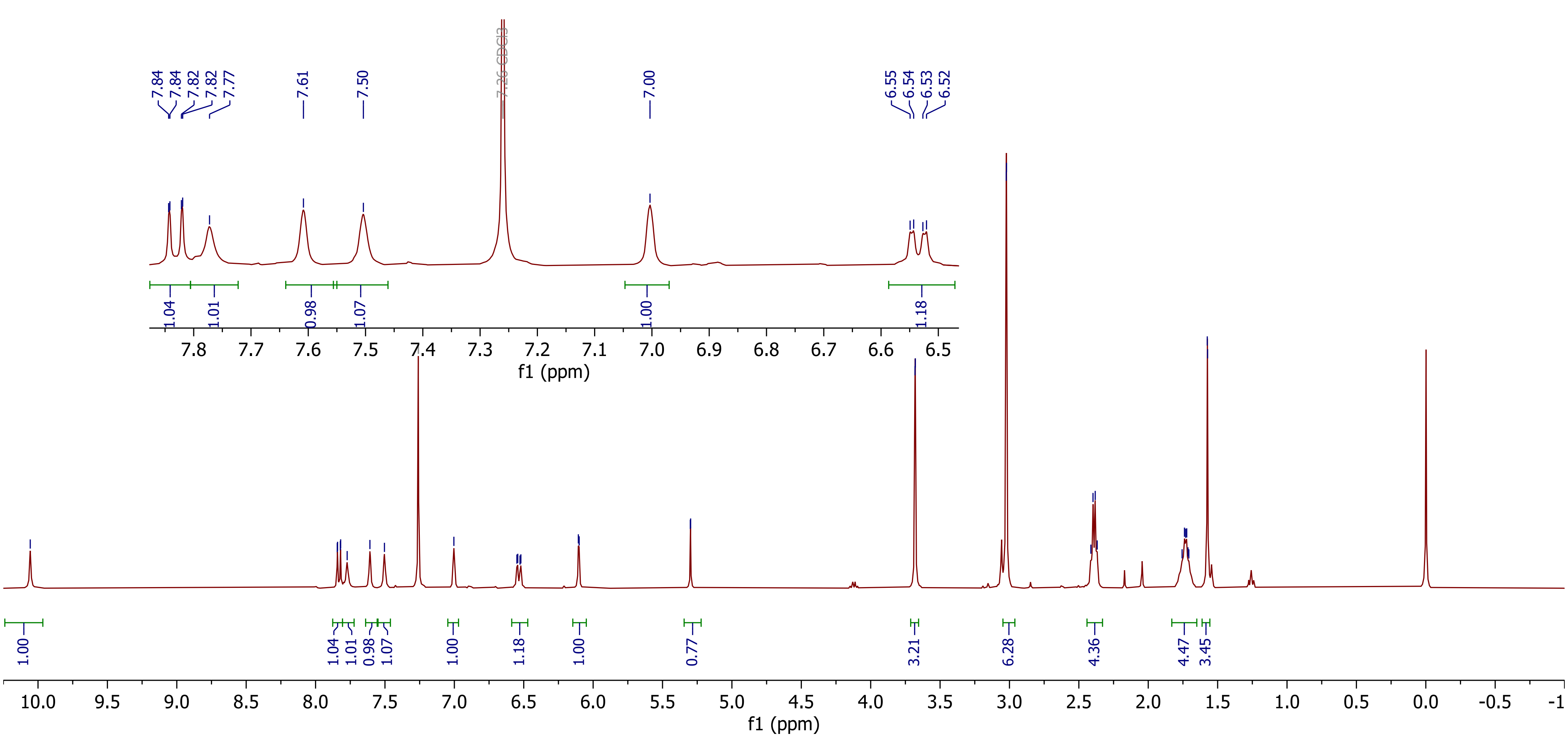

Compound SI-19

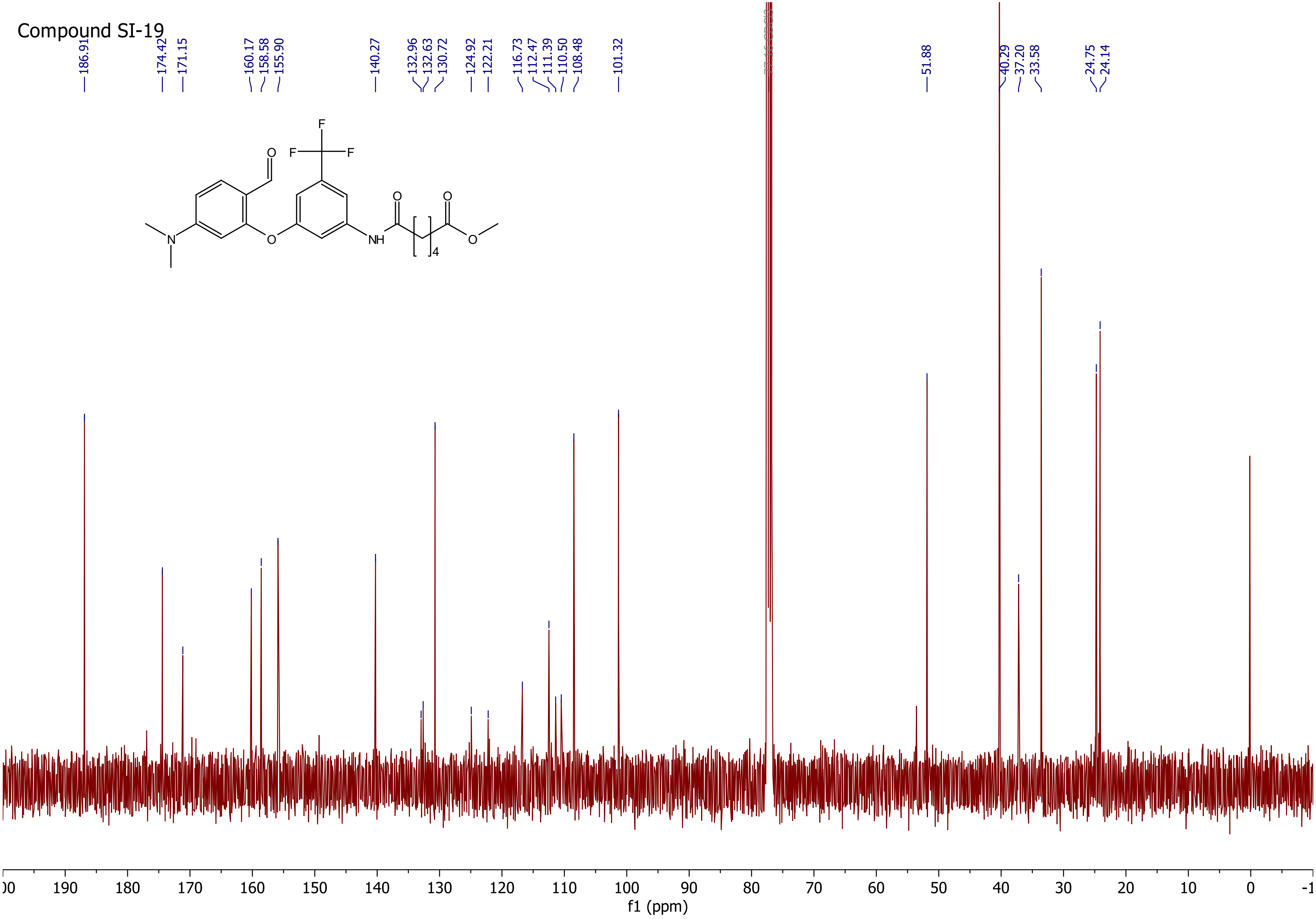

Compound SI-20

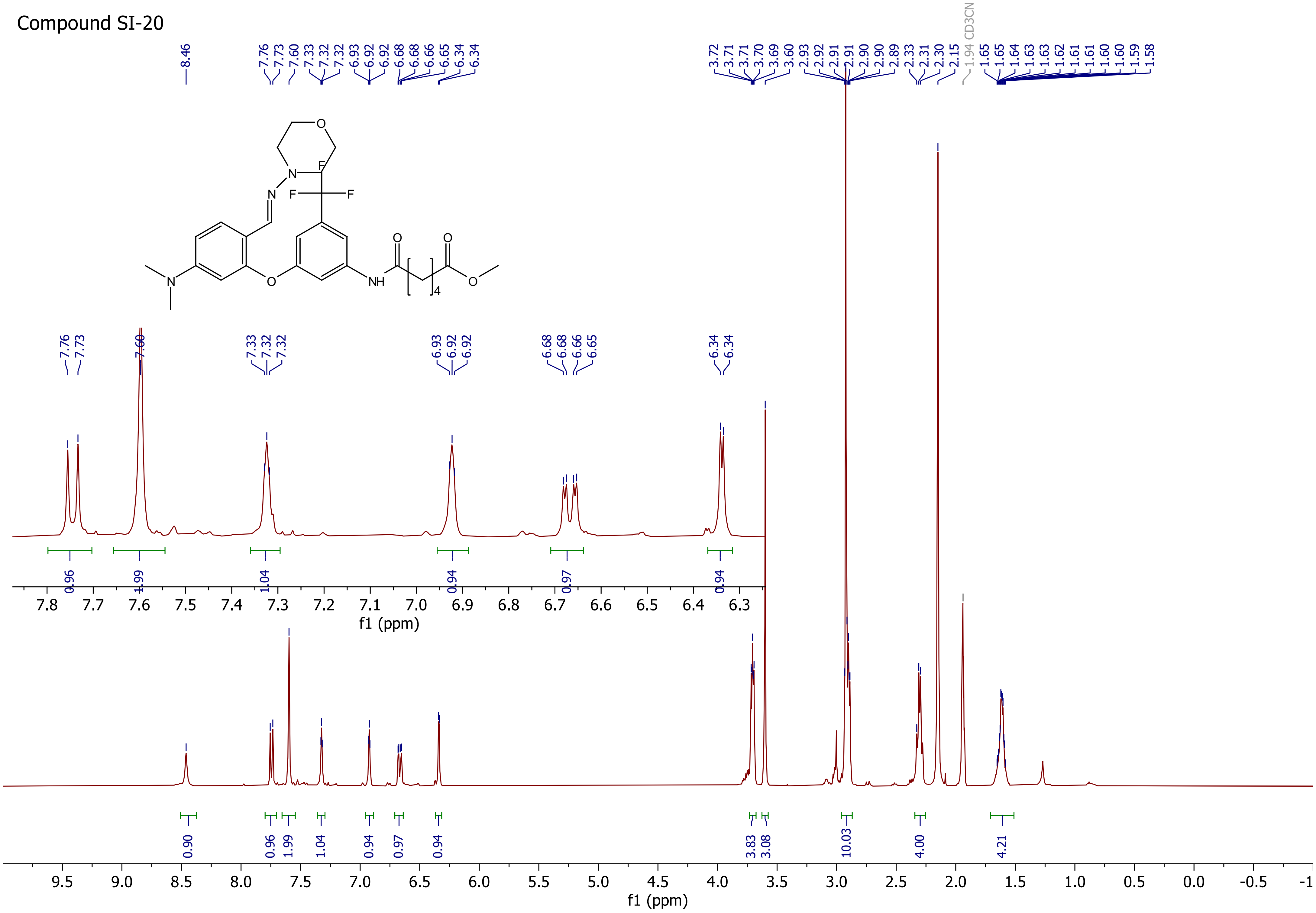

Compound SI-20

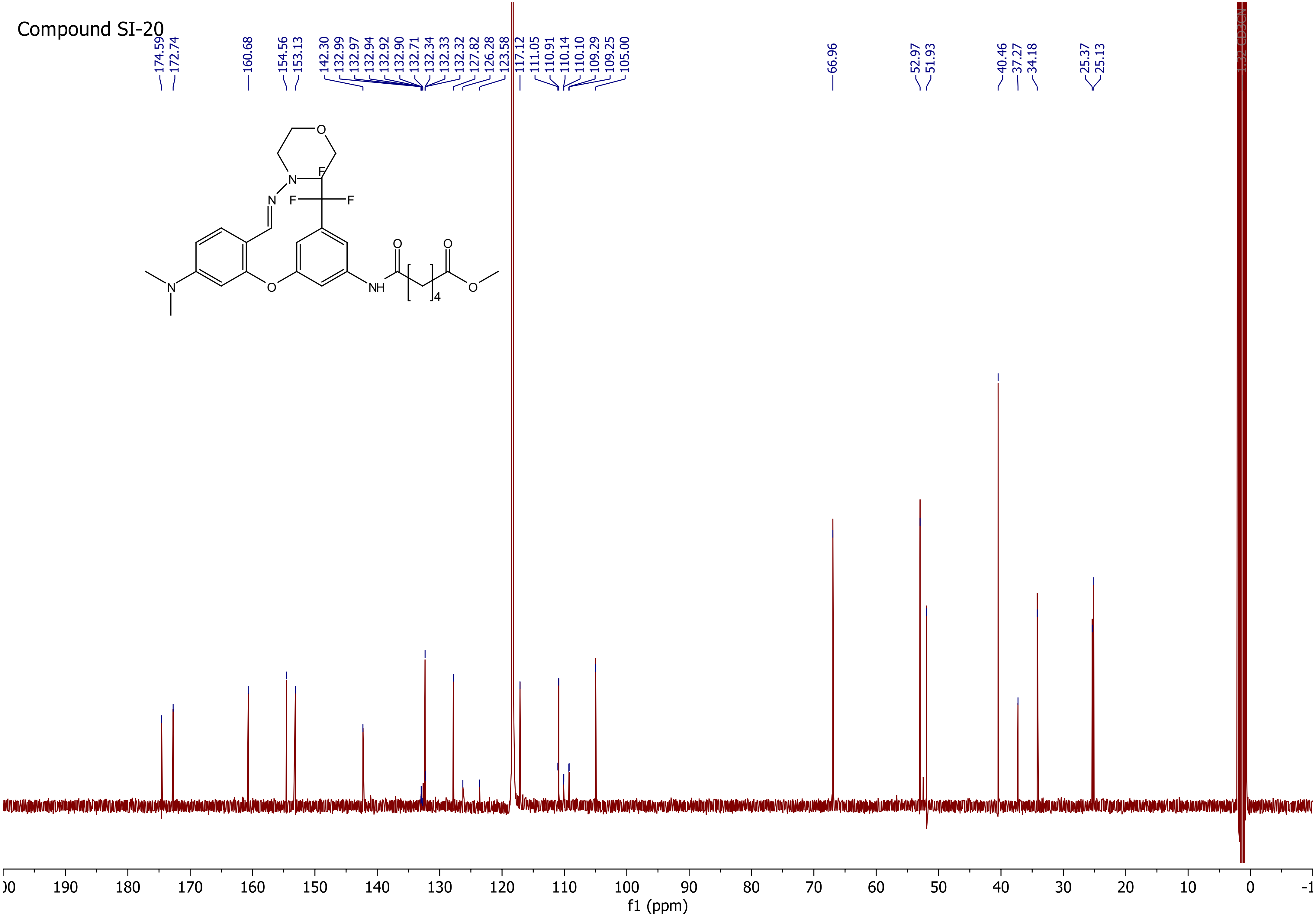

Compound SI-21

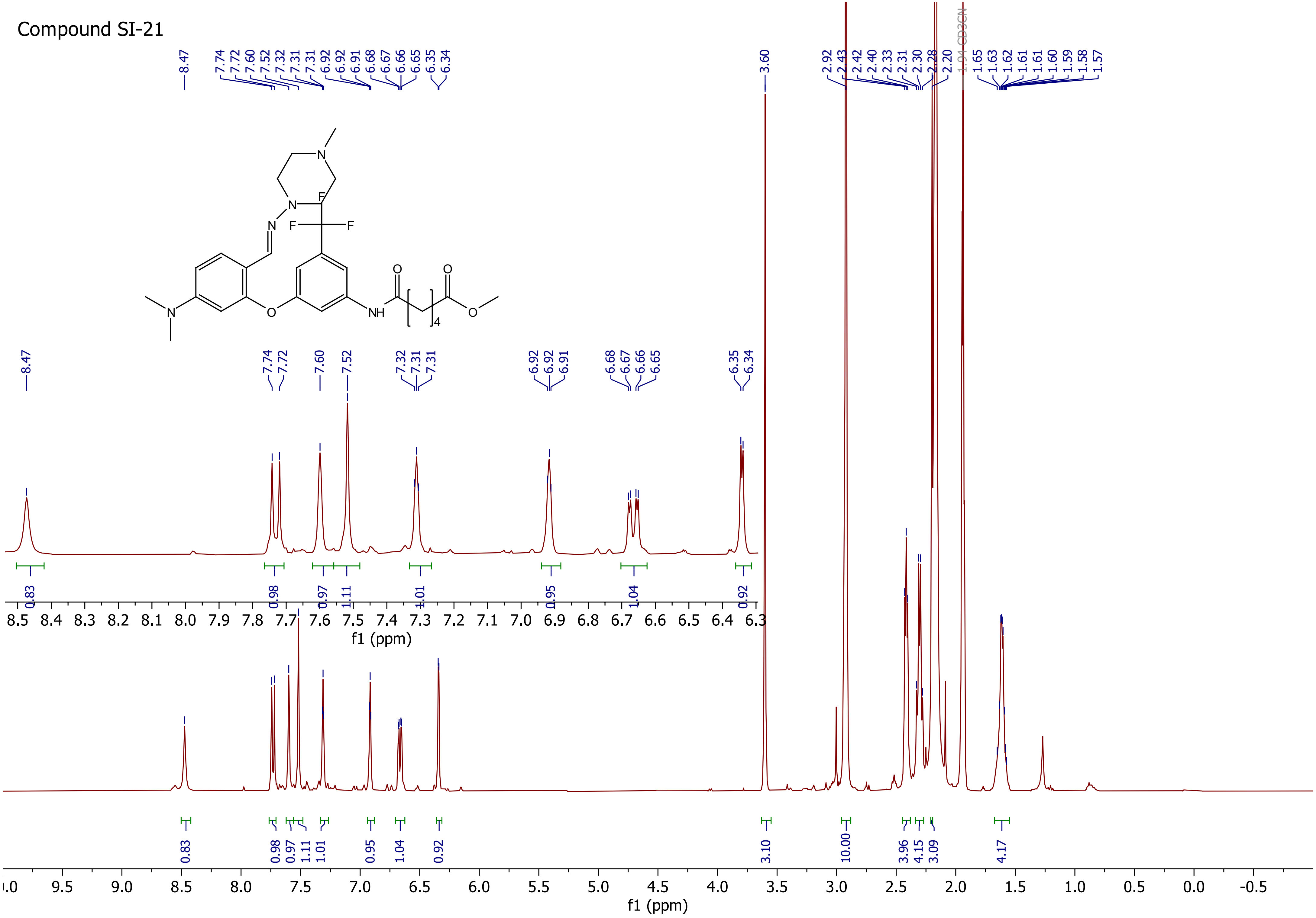

Compound SI-21

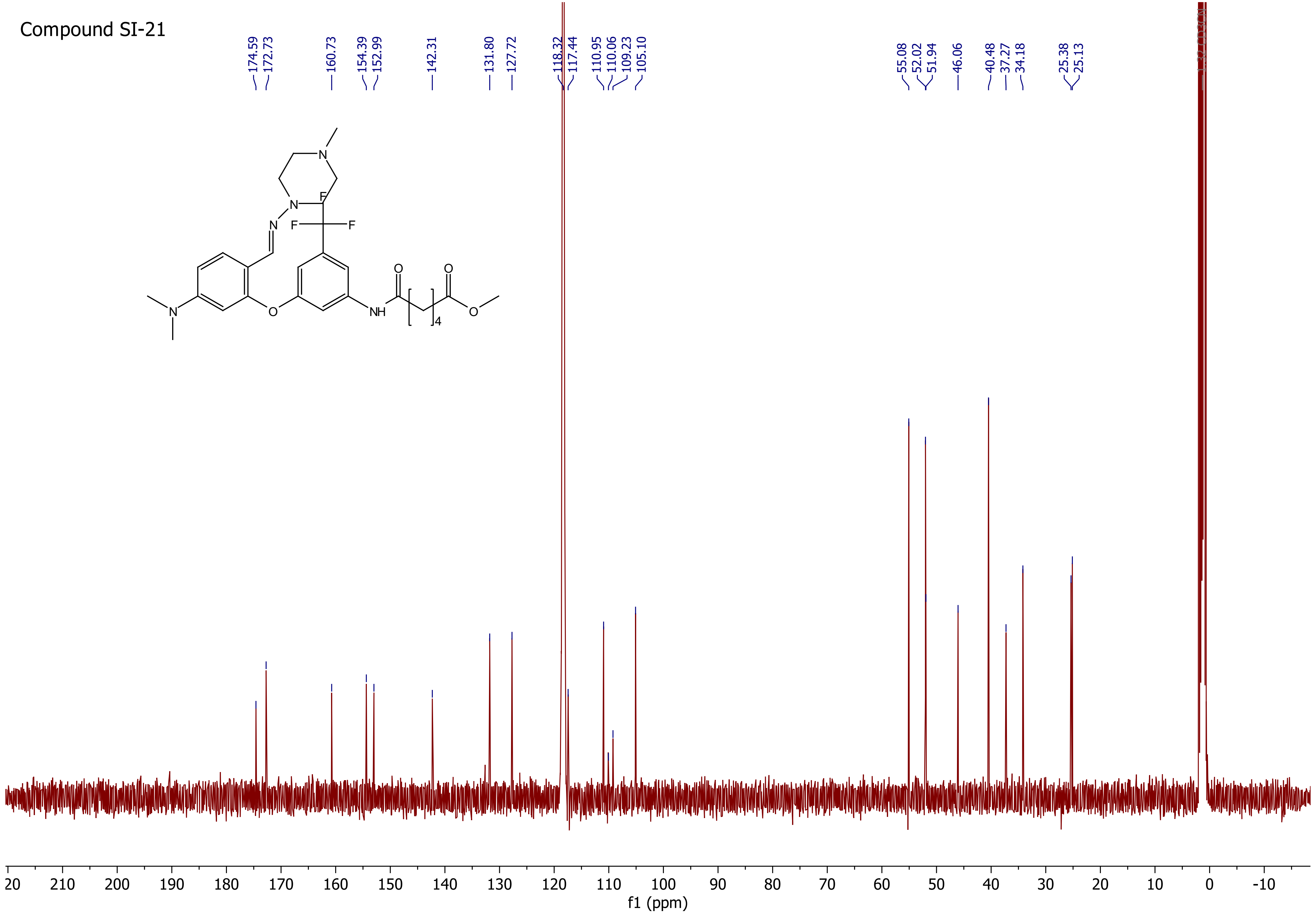

Compound SI-22

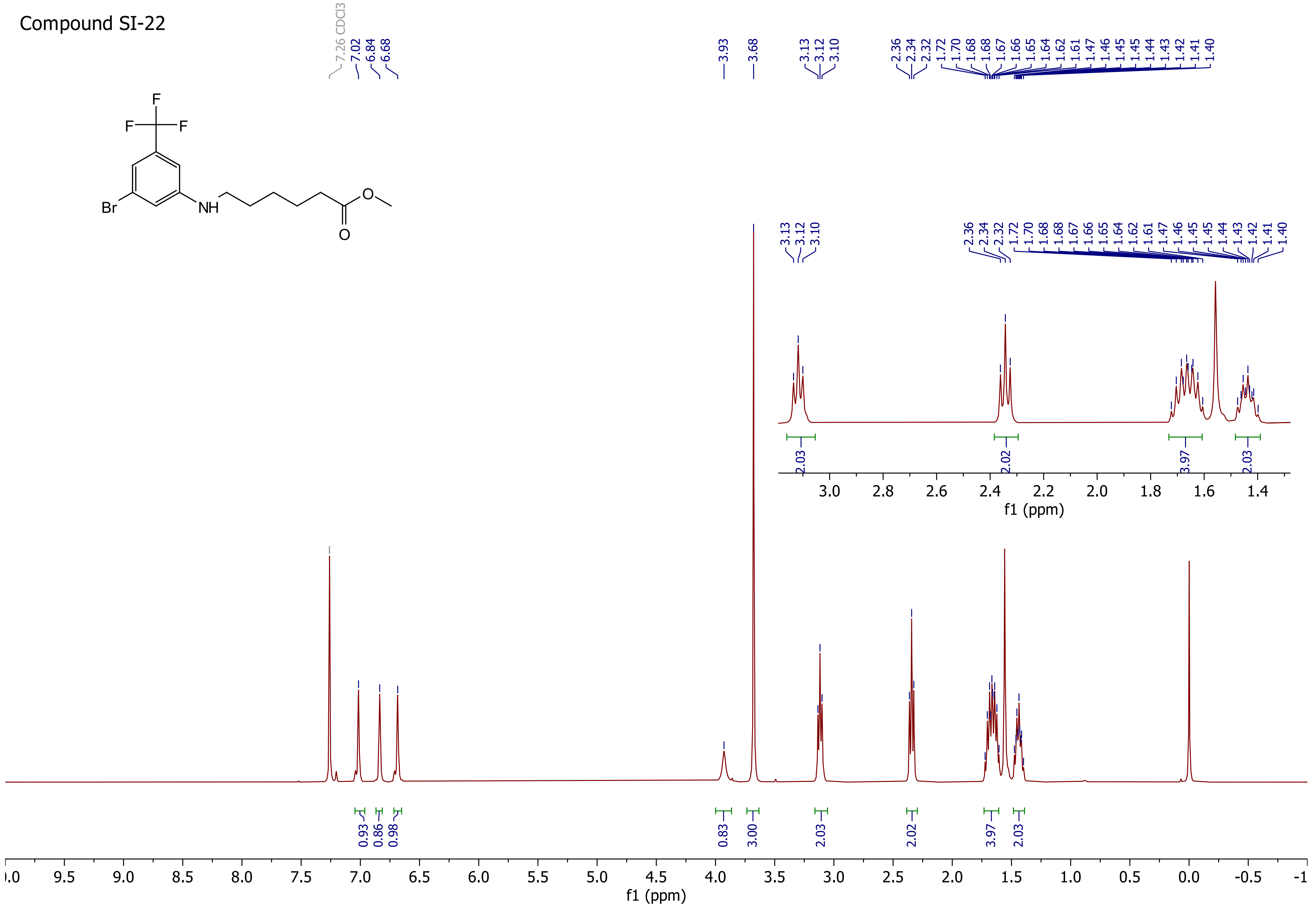

Compound SI-22

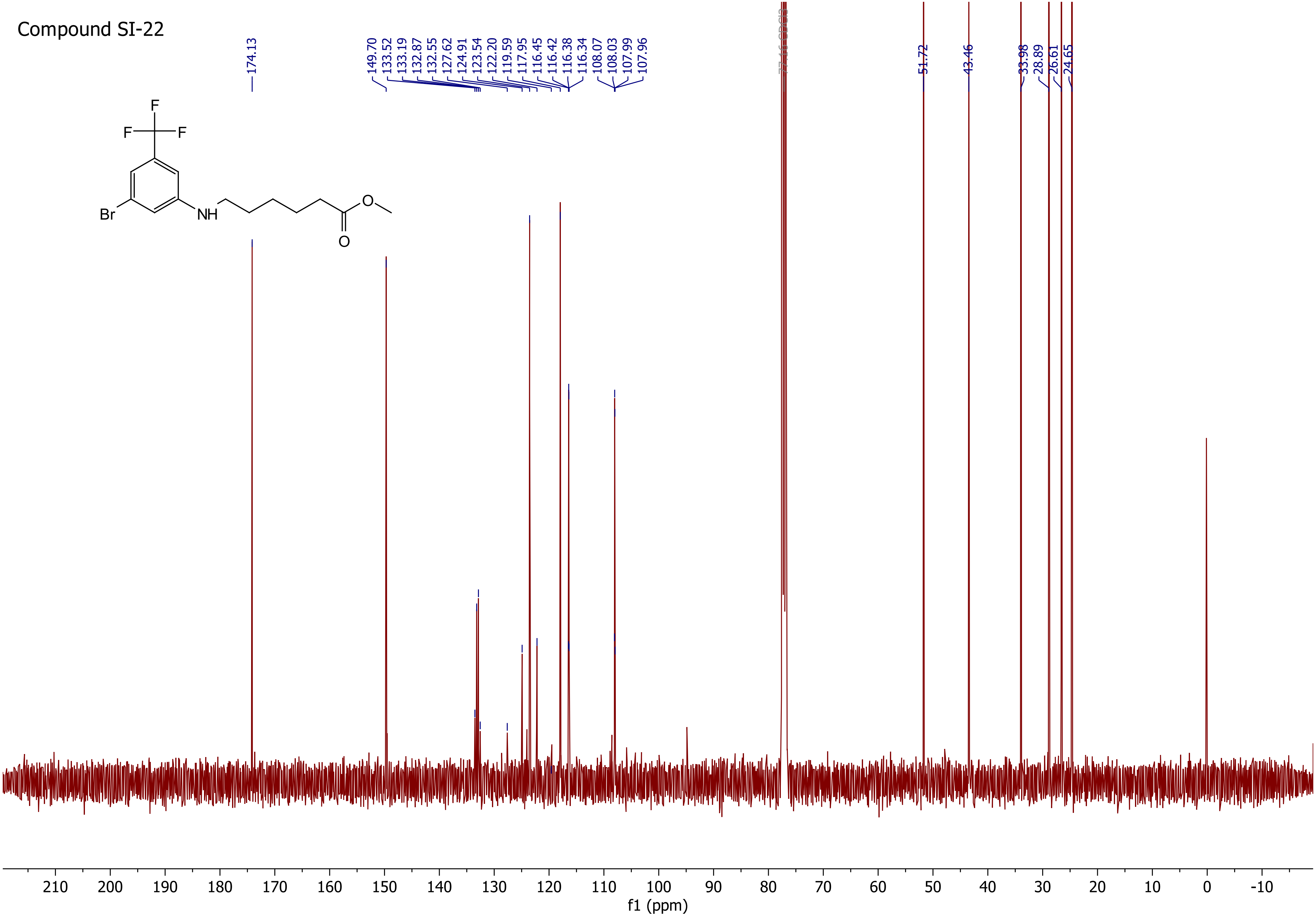

Compound SI-23

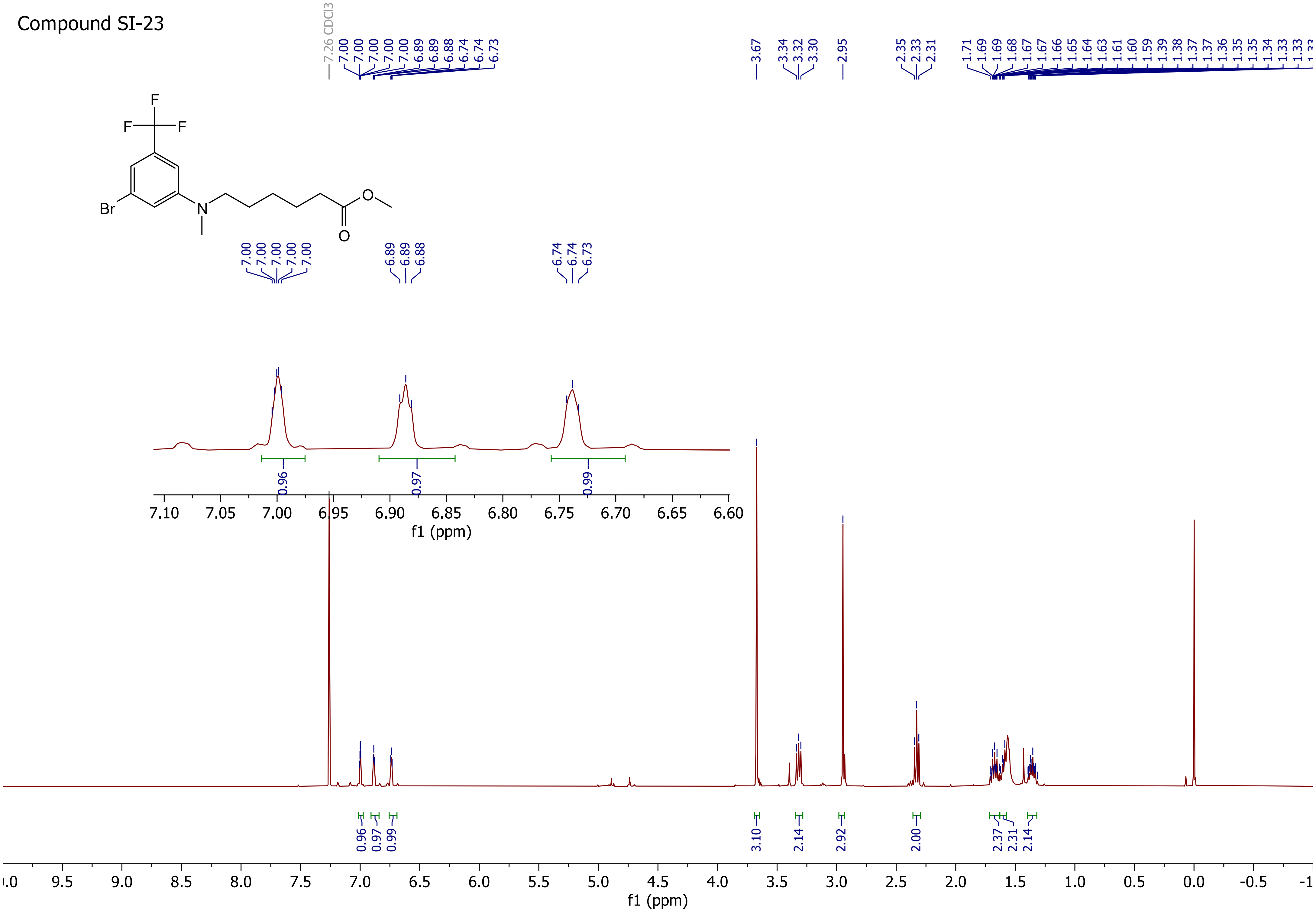

Compound SI-23

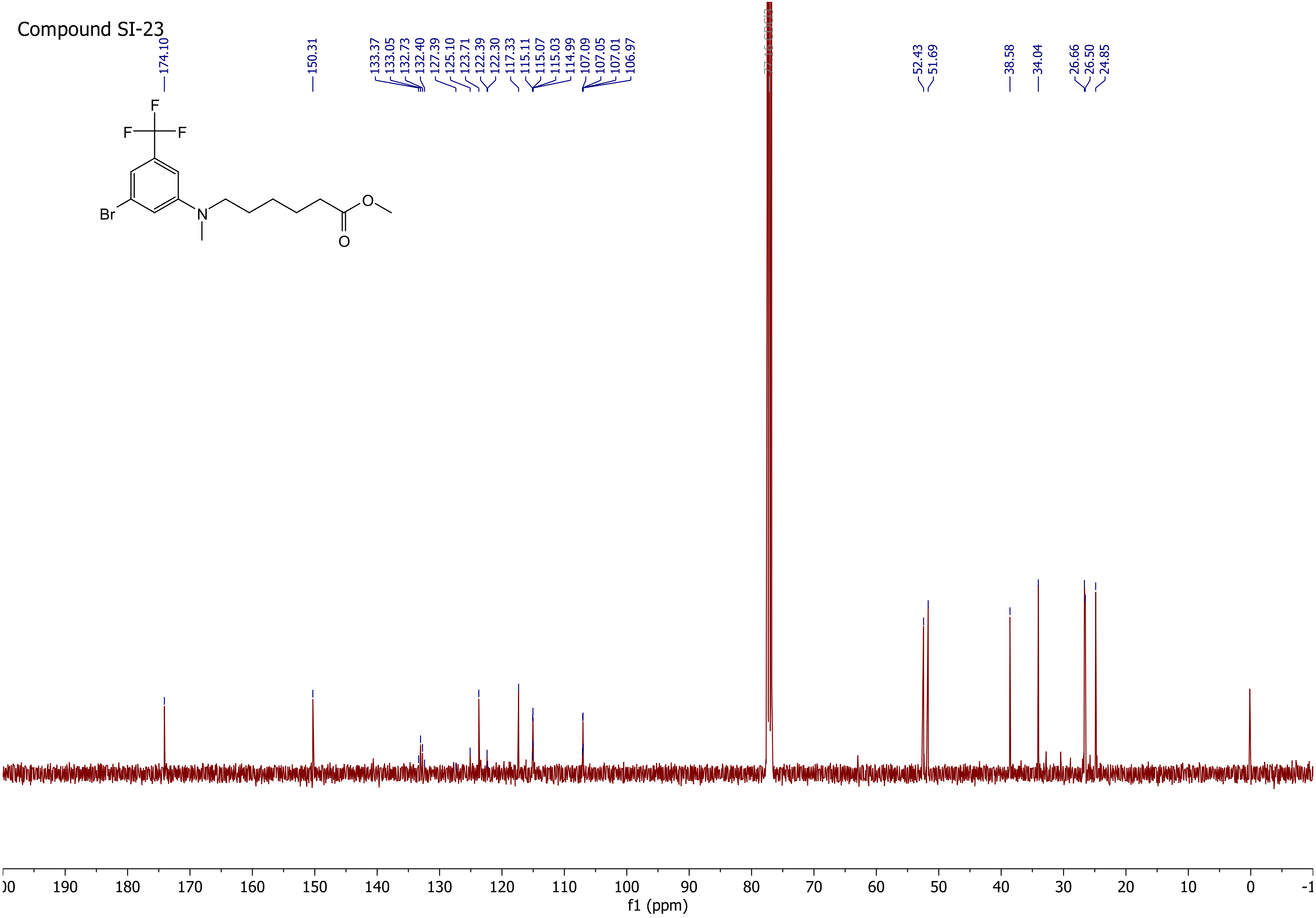

Compound SI-24

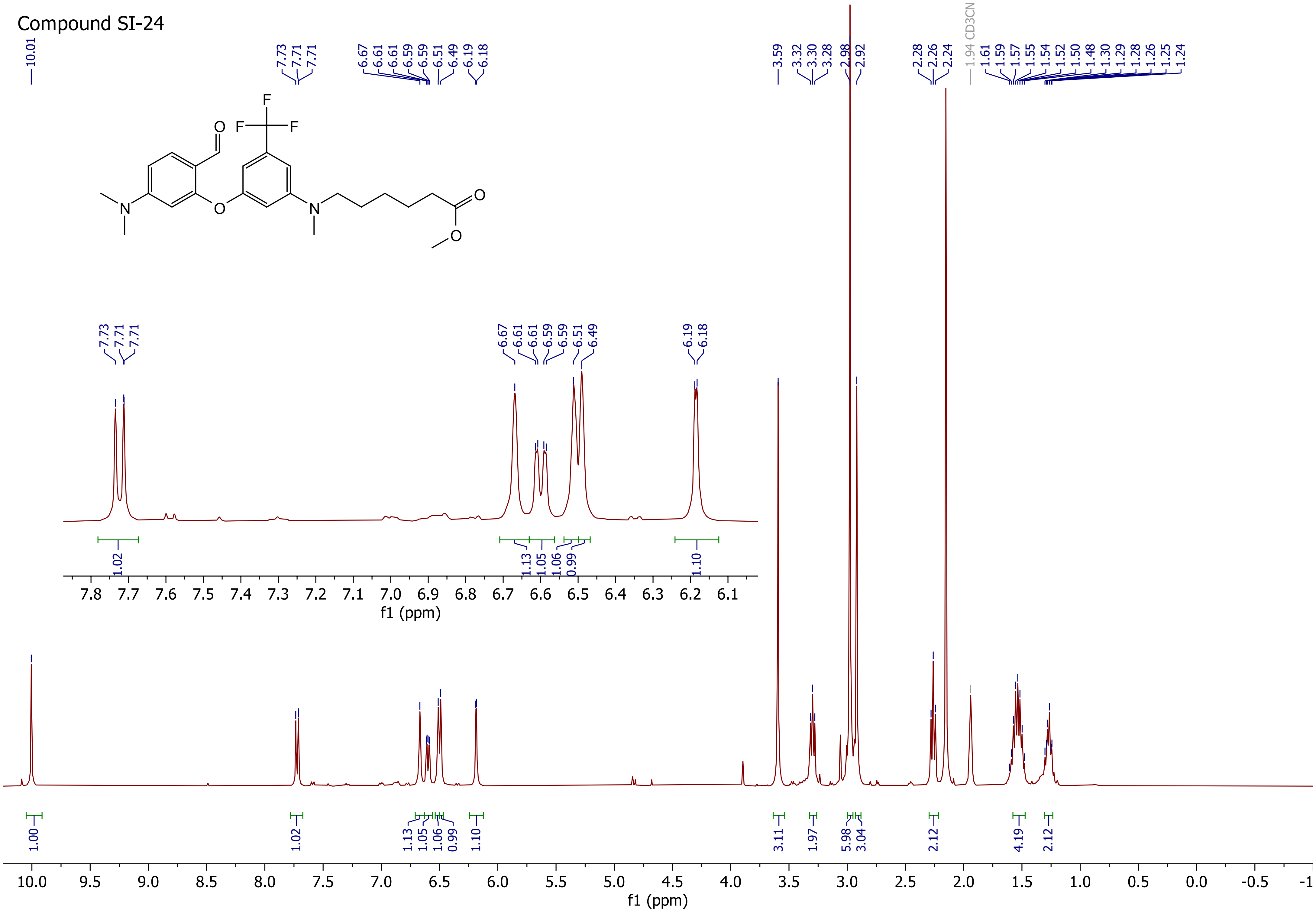

Compound SI-24

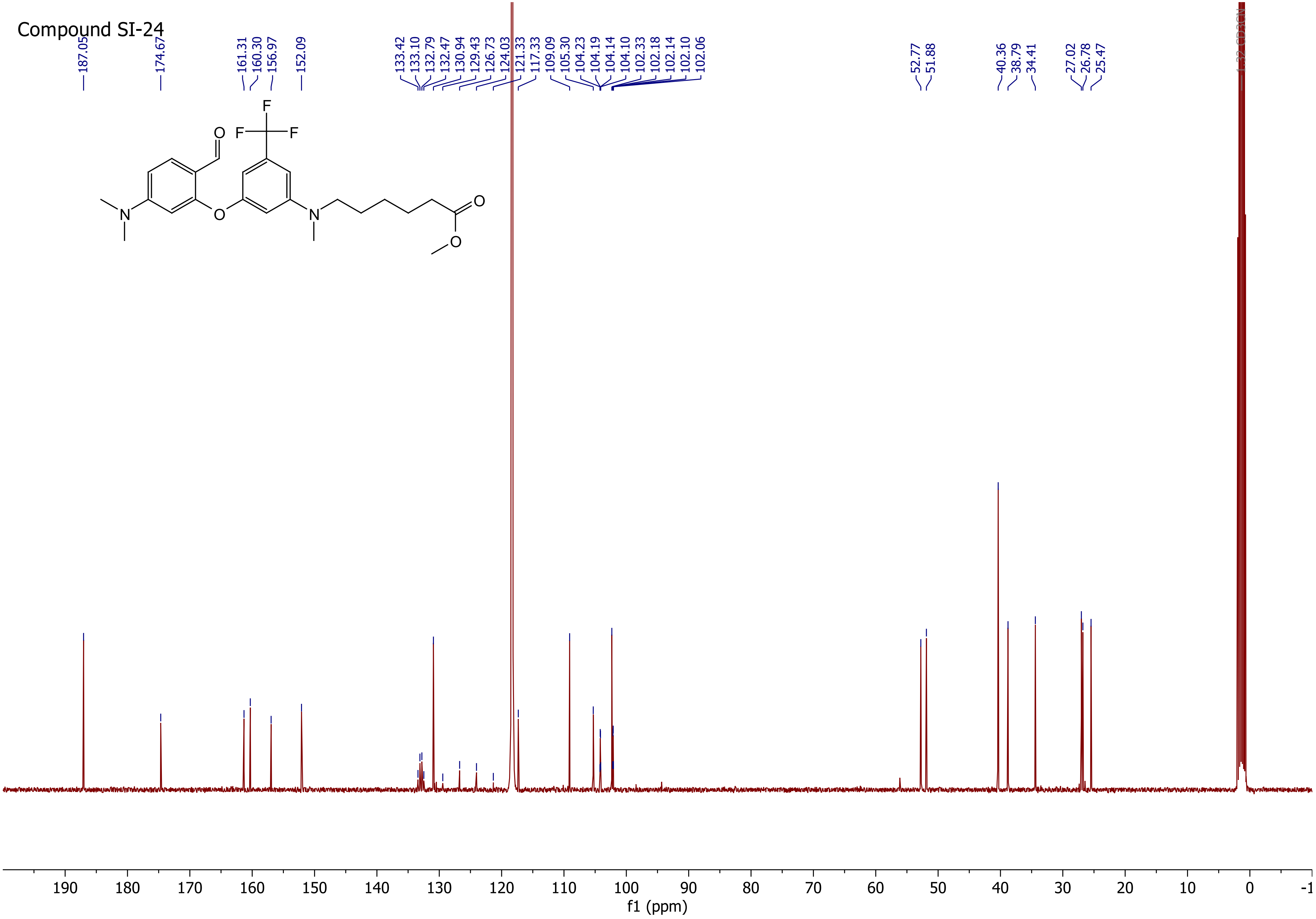

Compound SI-25

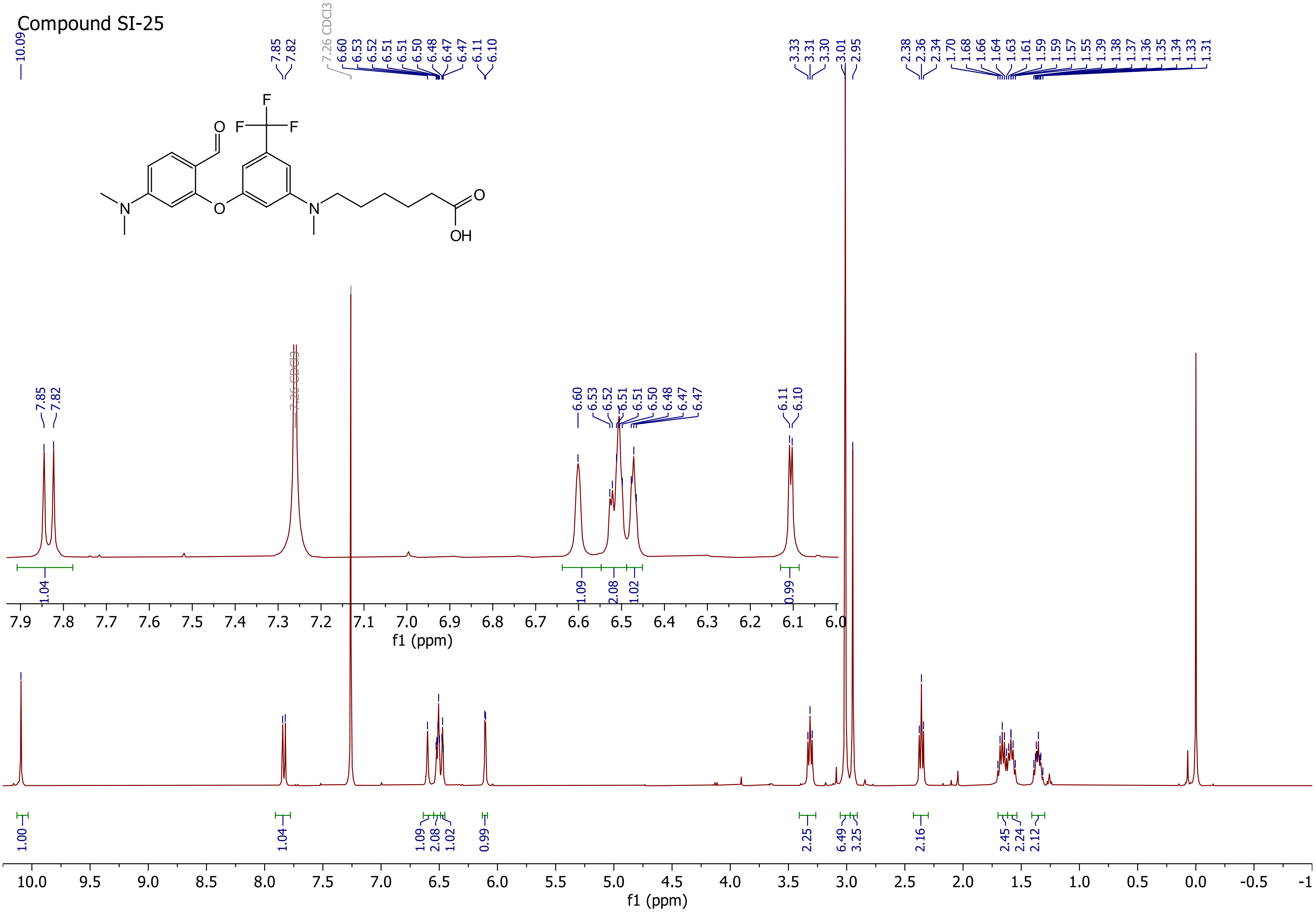

Compound SI-25

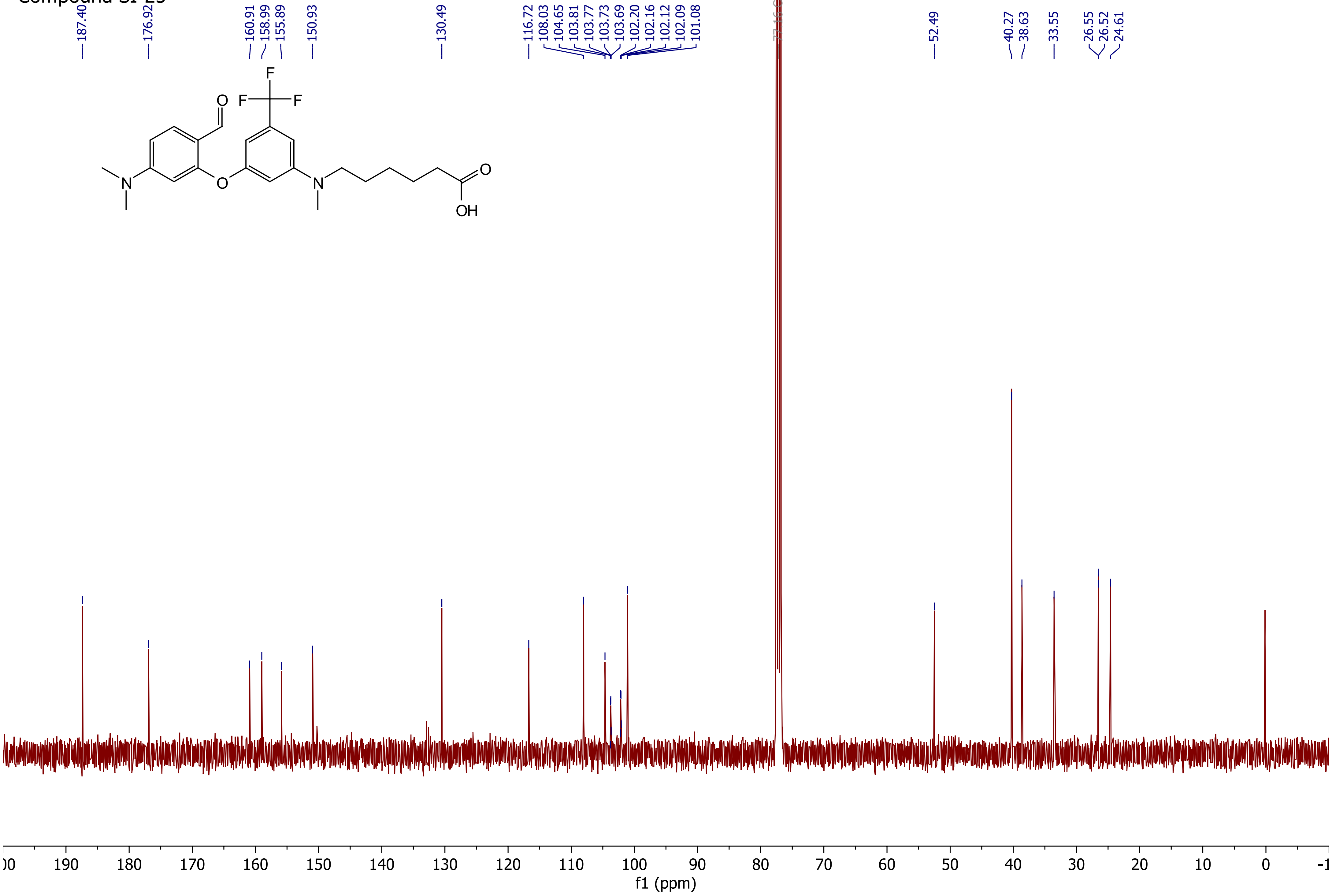

Compound SI-25-T

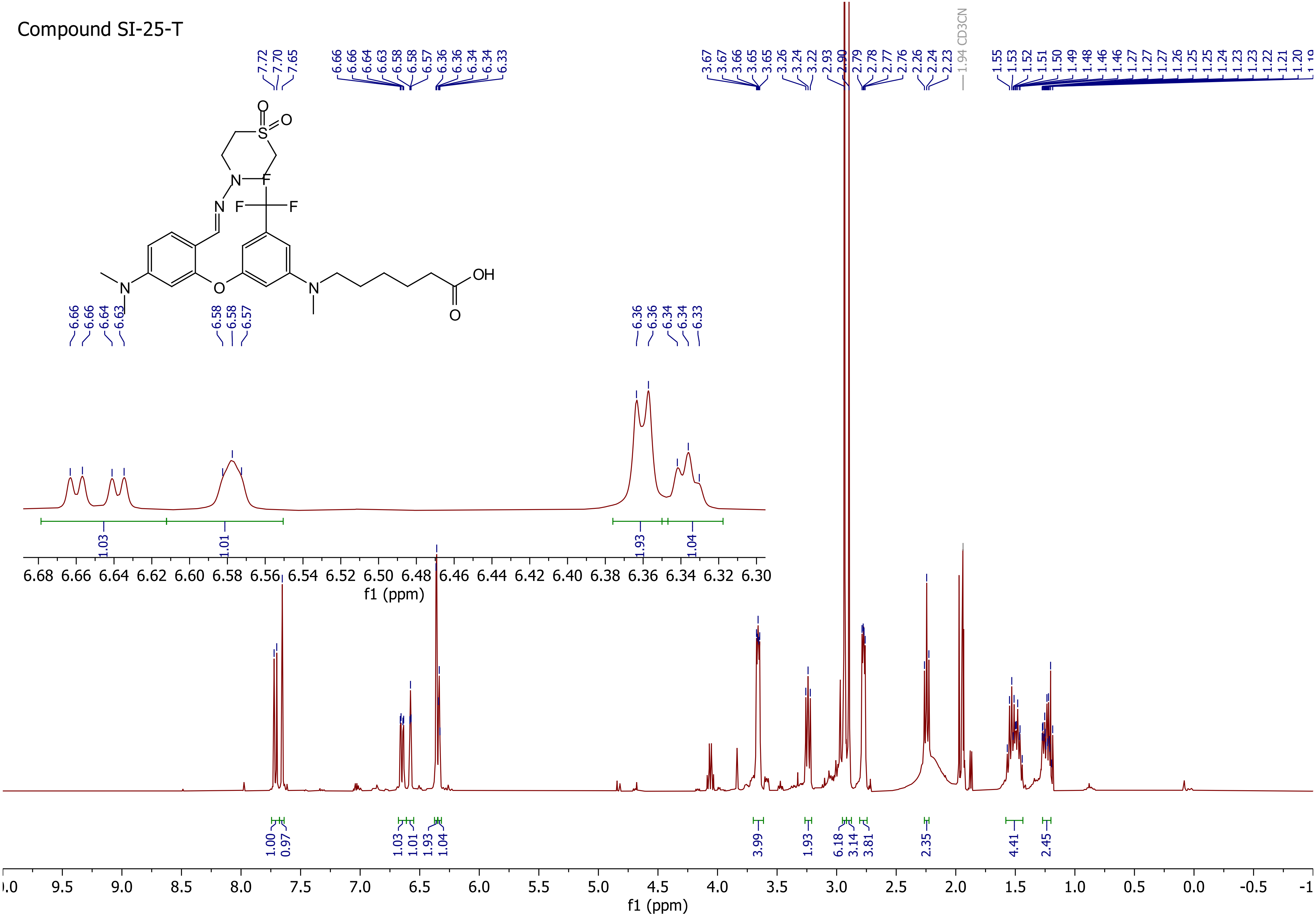

Compound SI-25-T

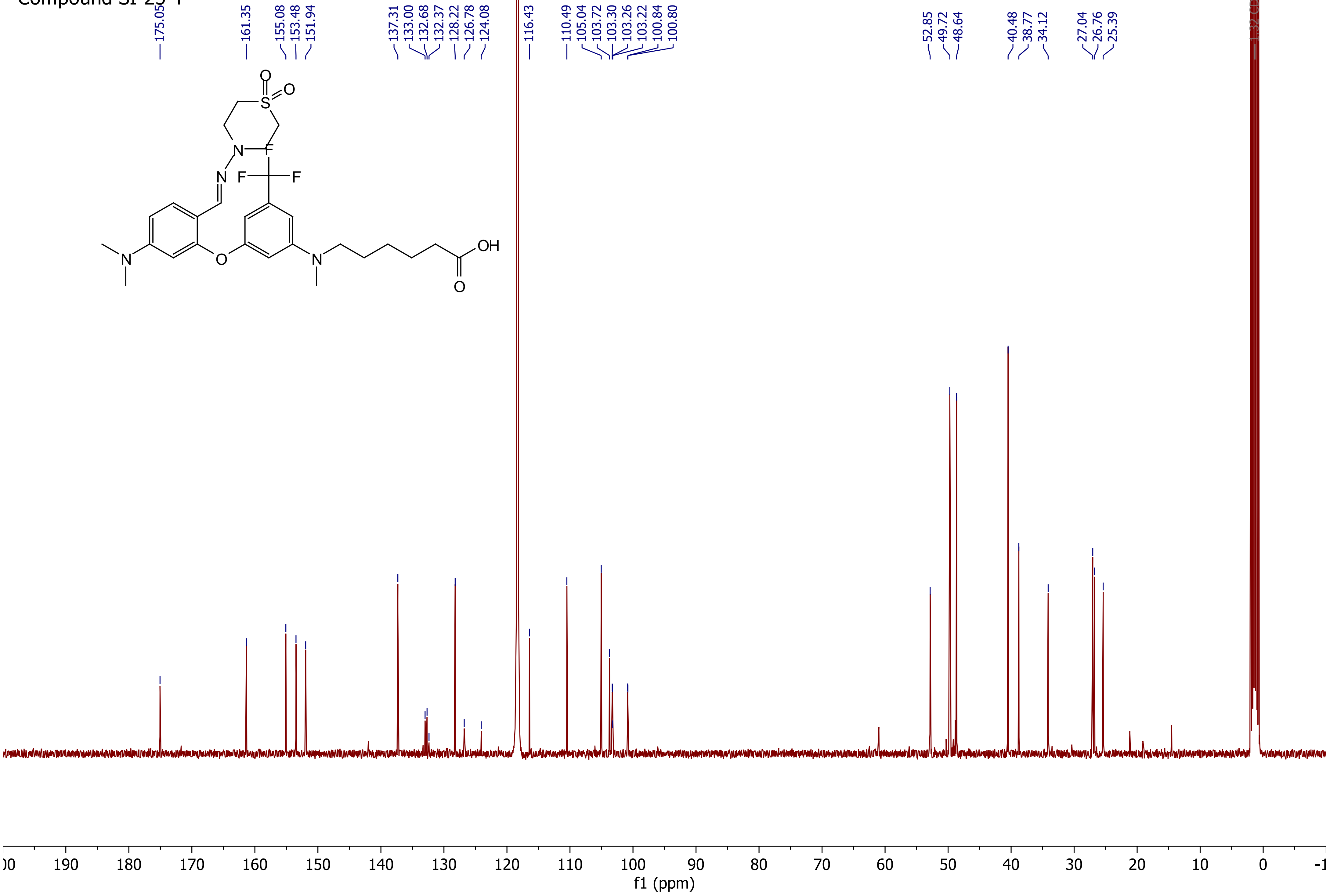

Compound SI-25-Halo

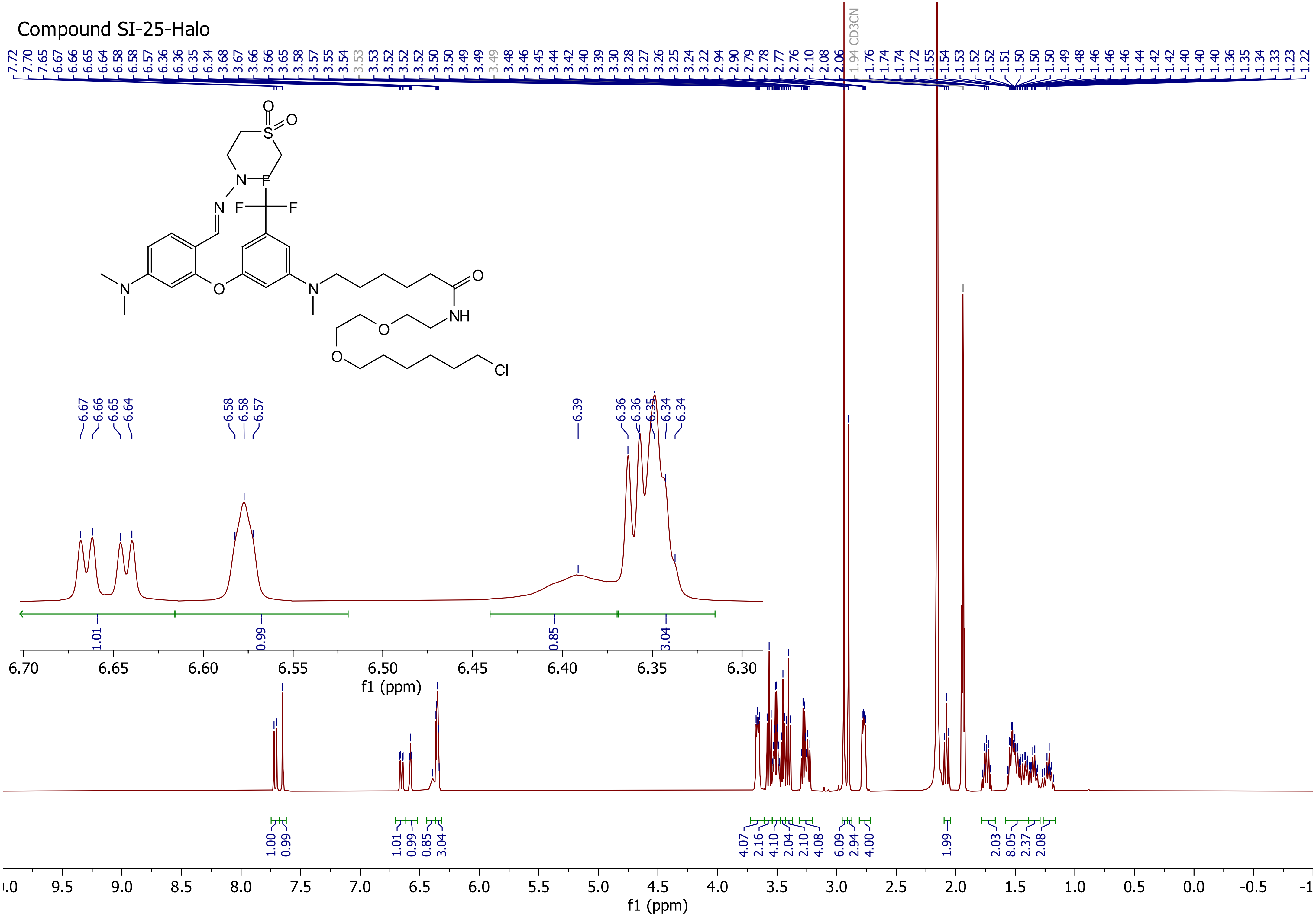

Compound SI-25-Halo

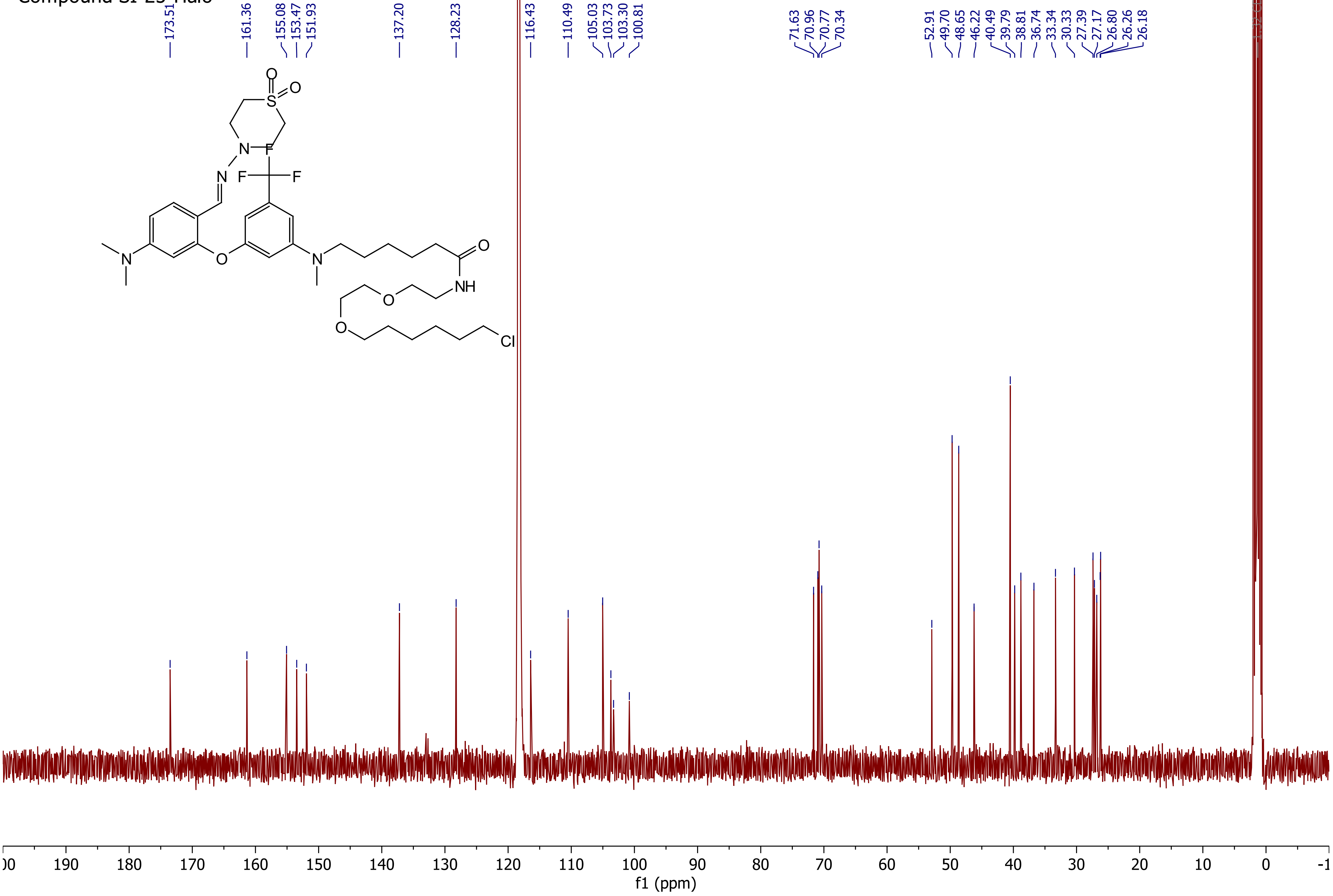

Compound SI-25-T-NHS

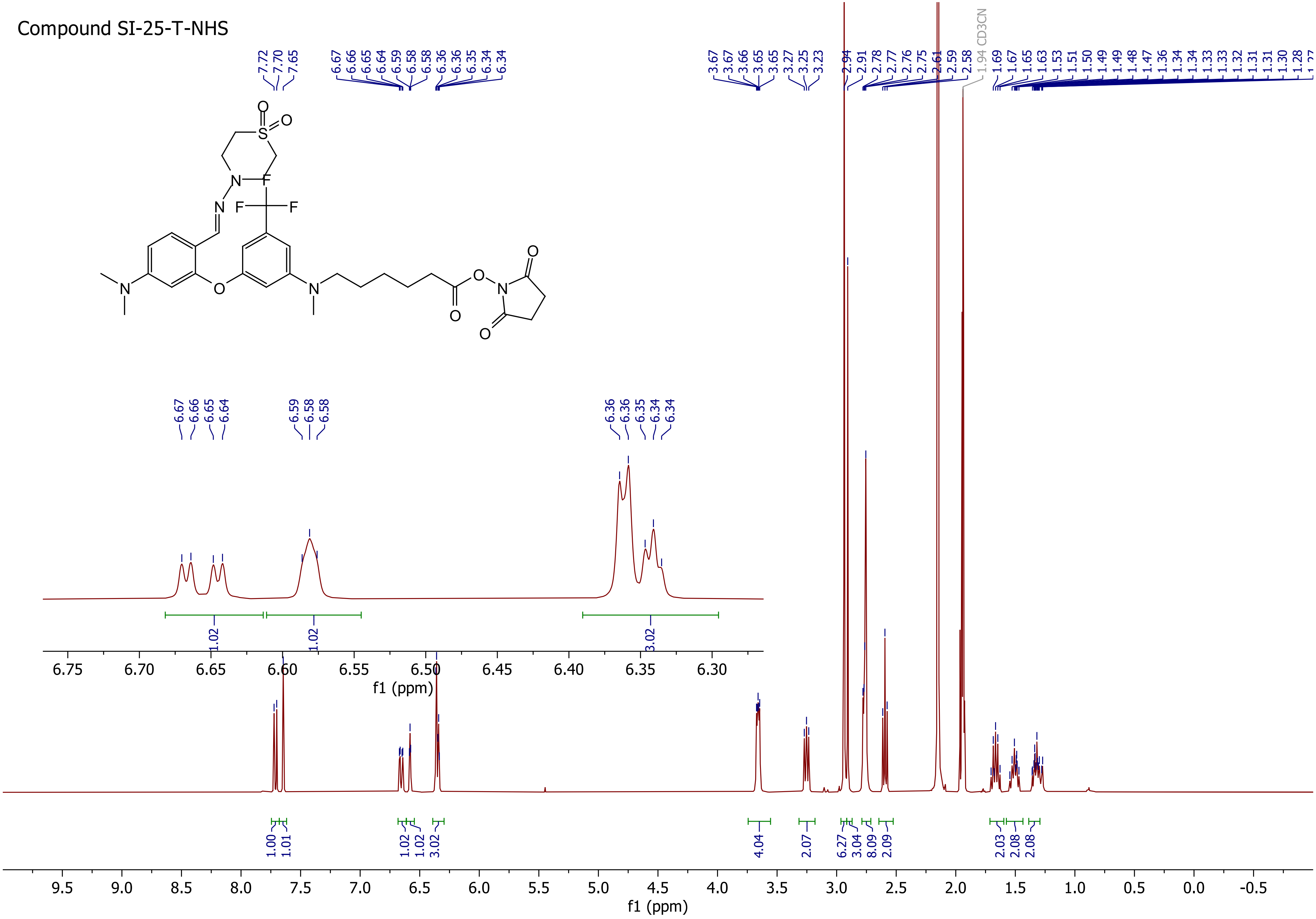

Compound SI-25-T-NHS

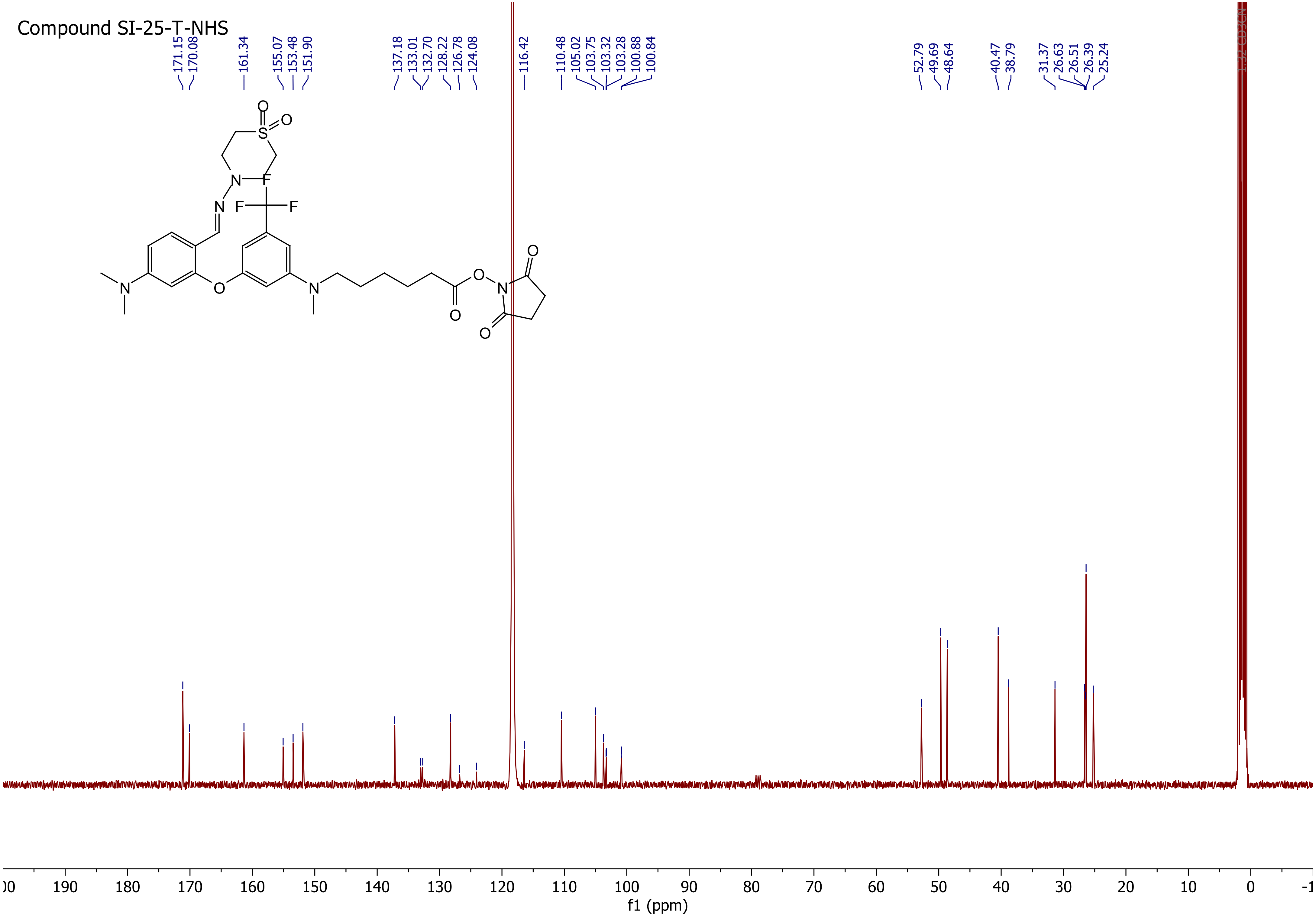

Compound SI-26

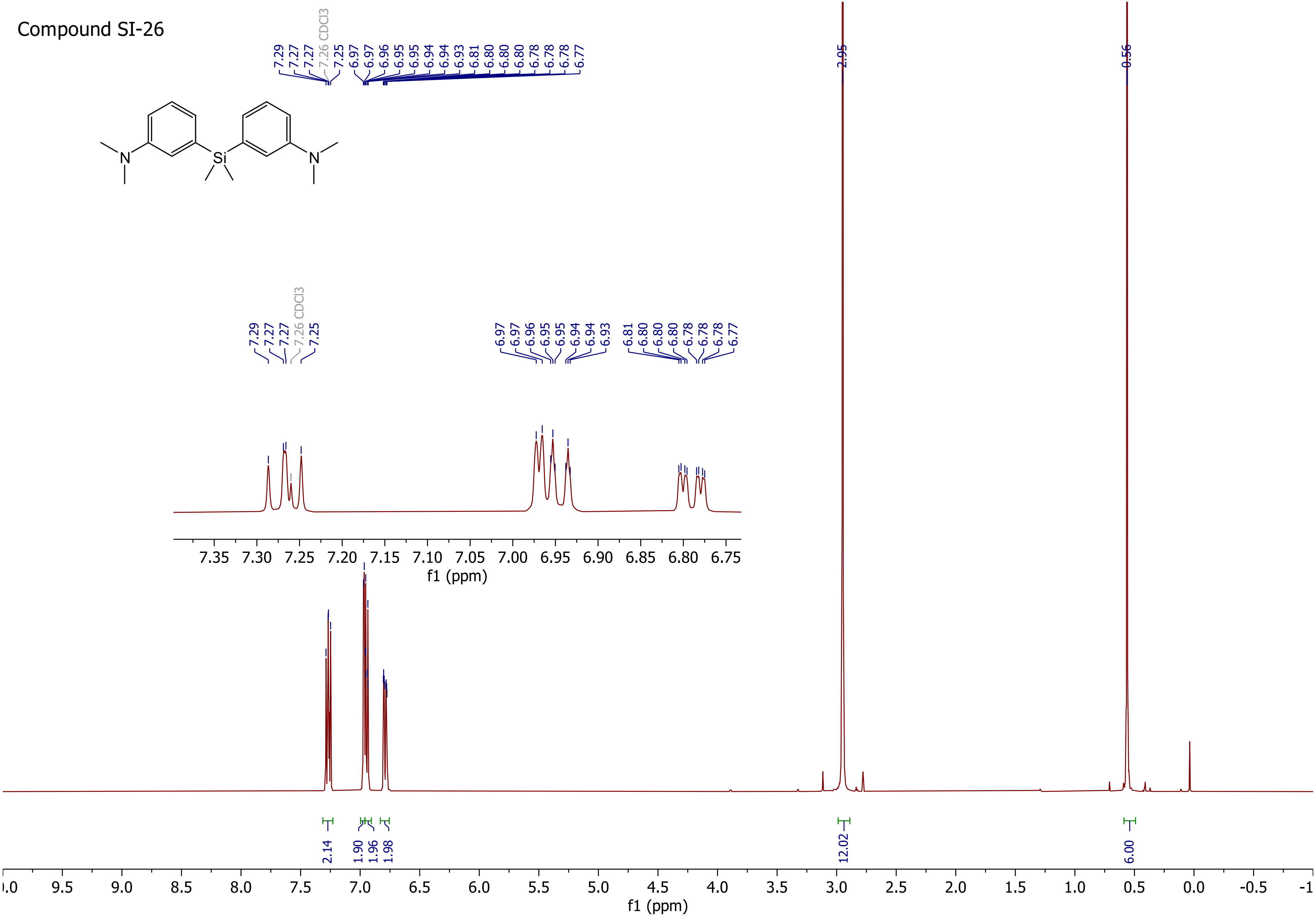

Compound SI-26

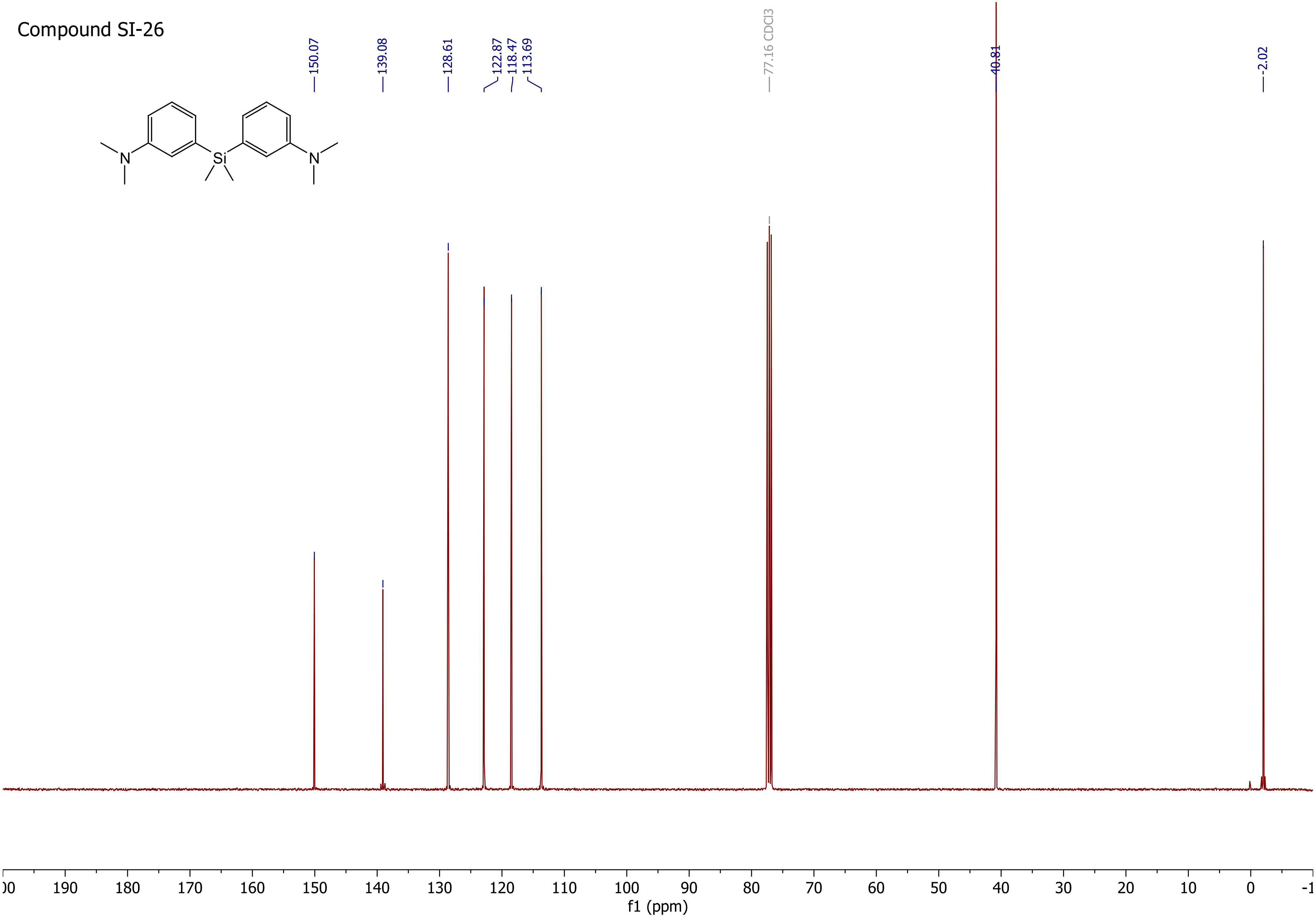

Compound SI-27

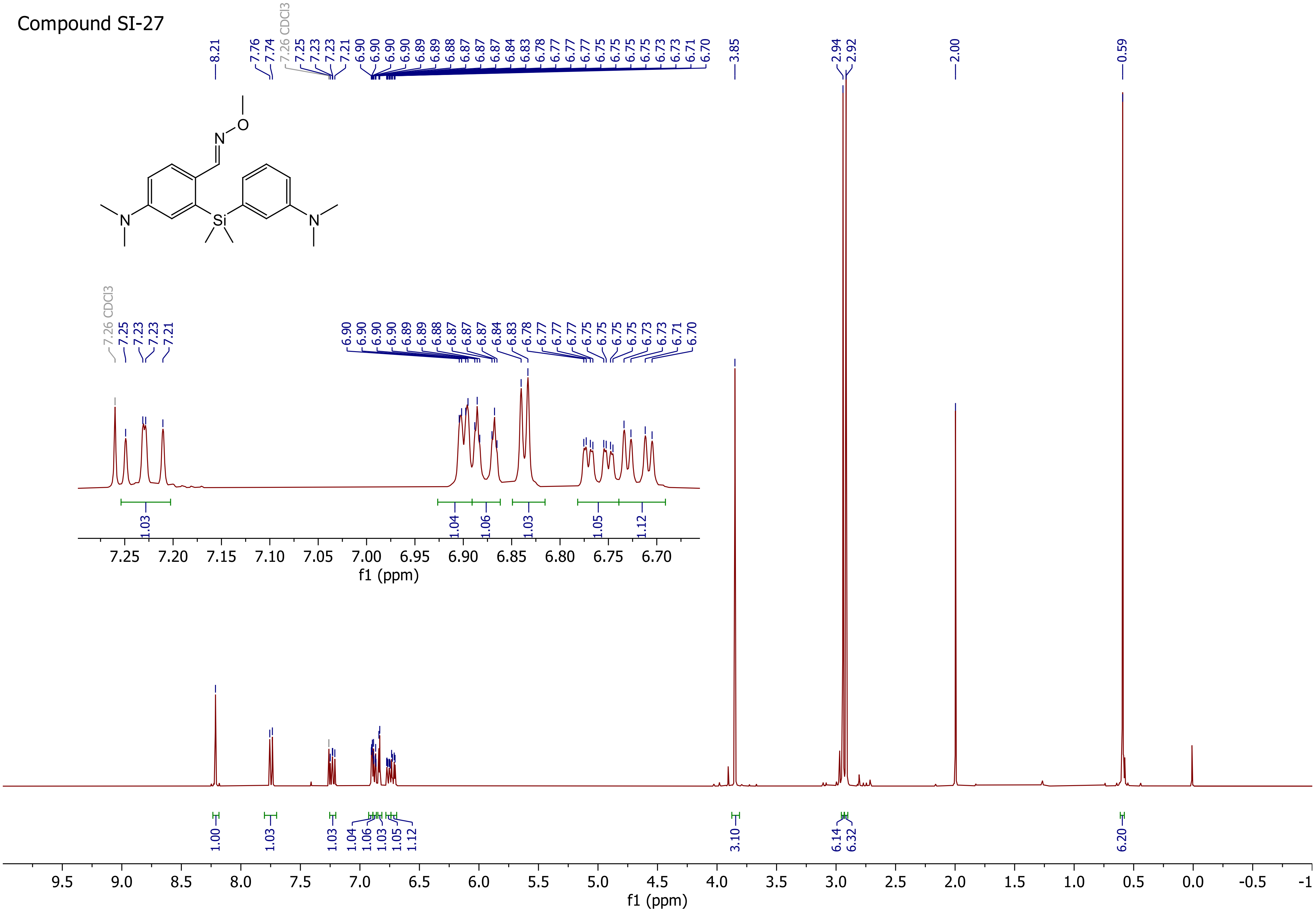

Compound SI-27

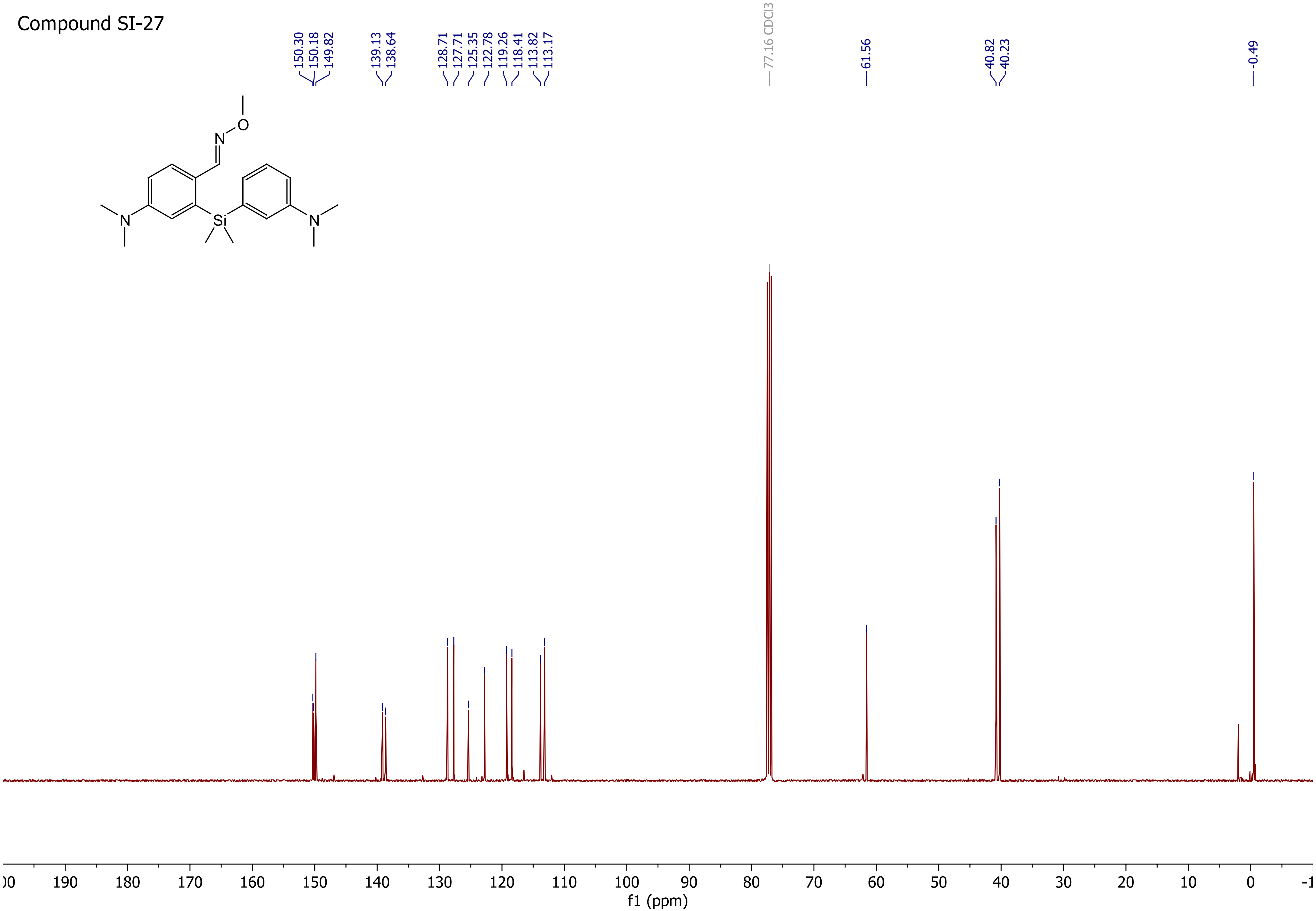

Compound SI-28

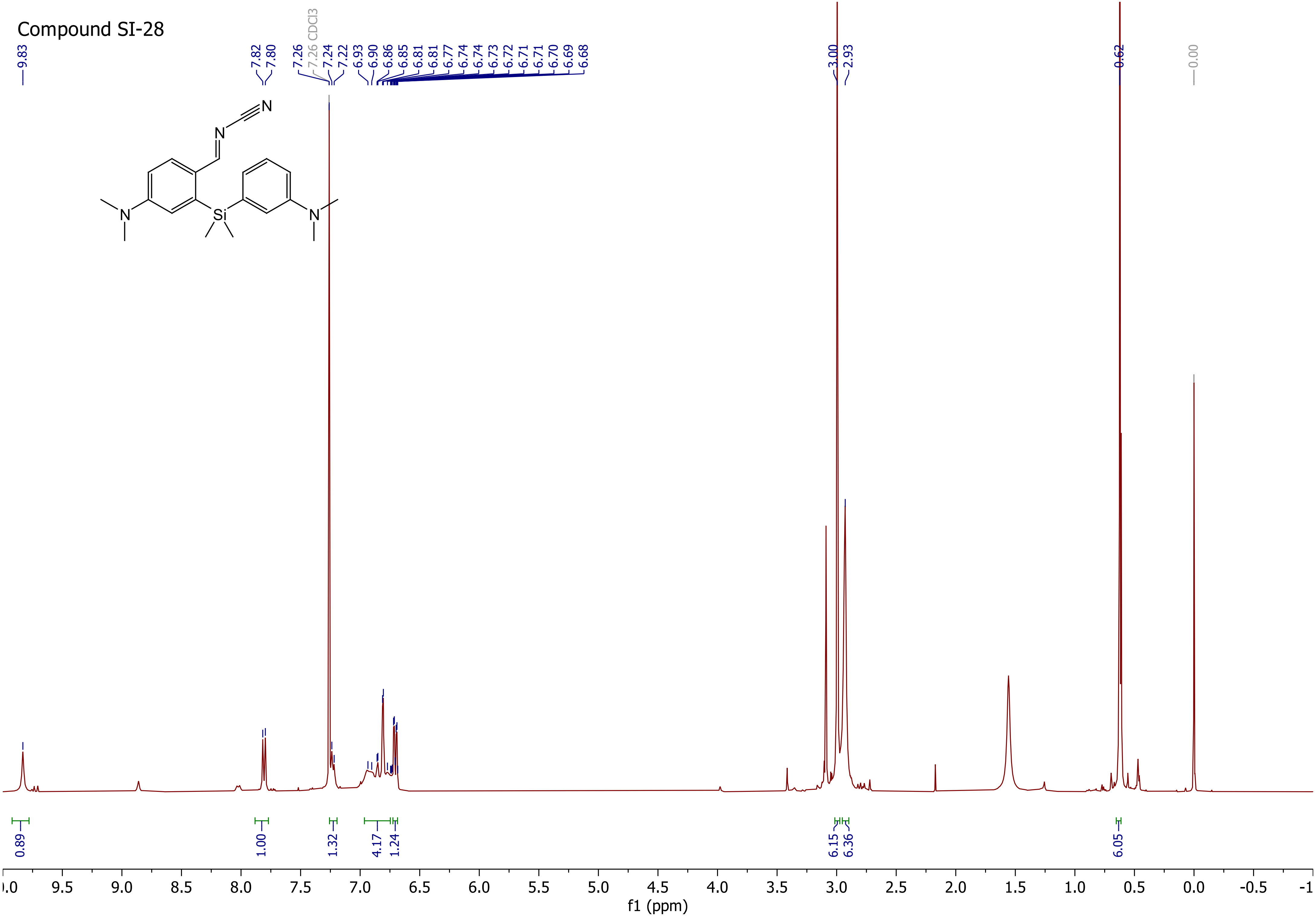

Compound SI-28

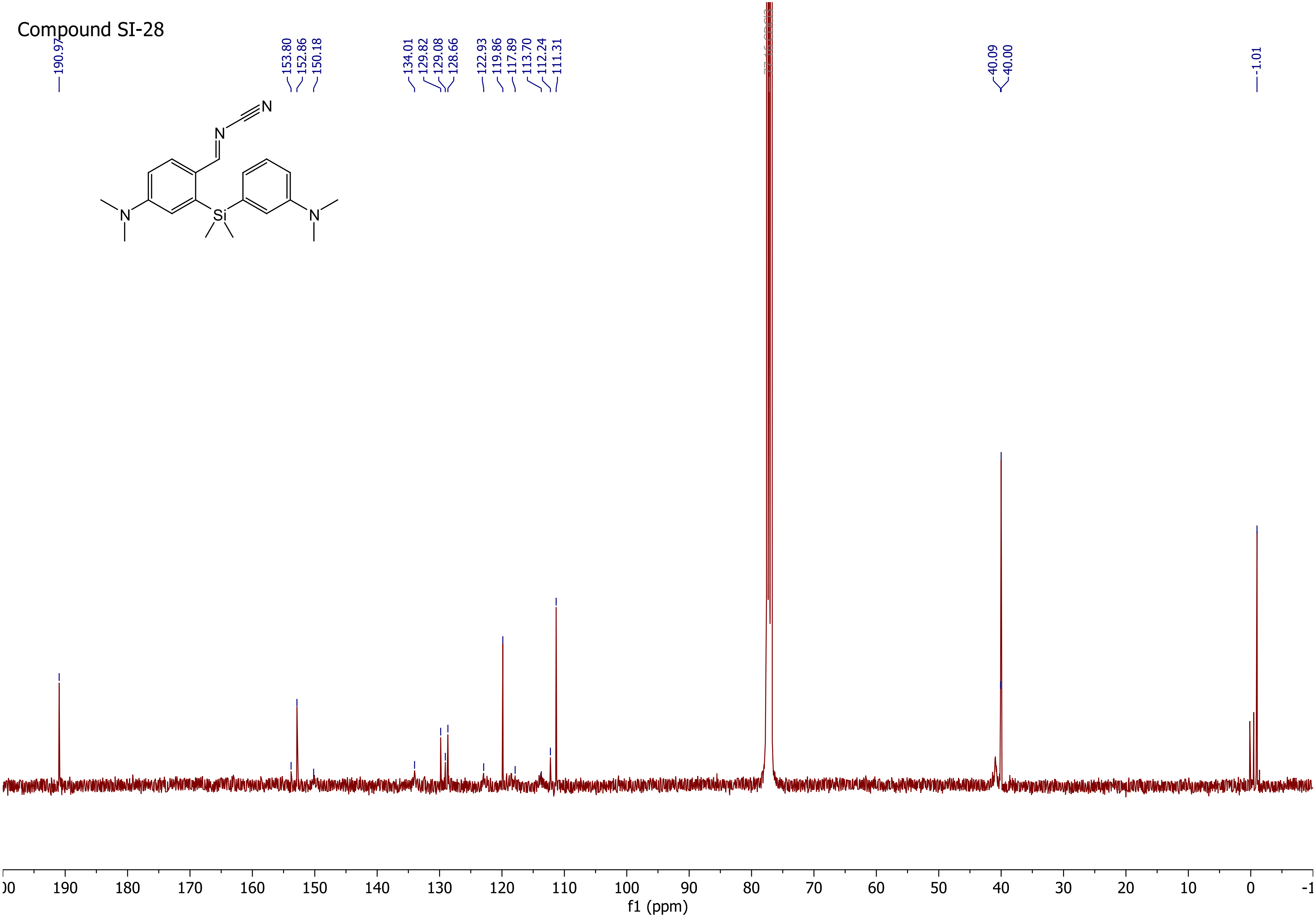

Compound SI-29

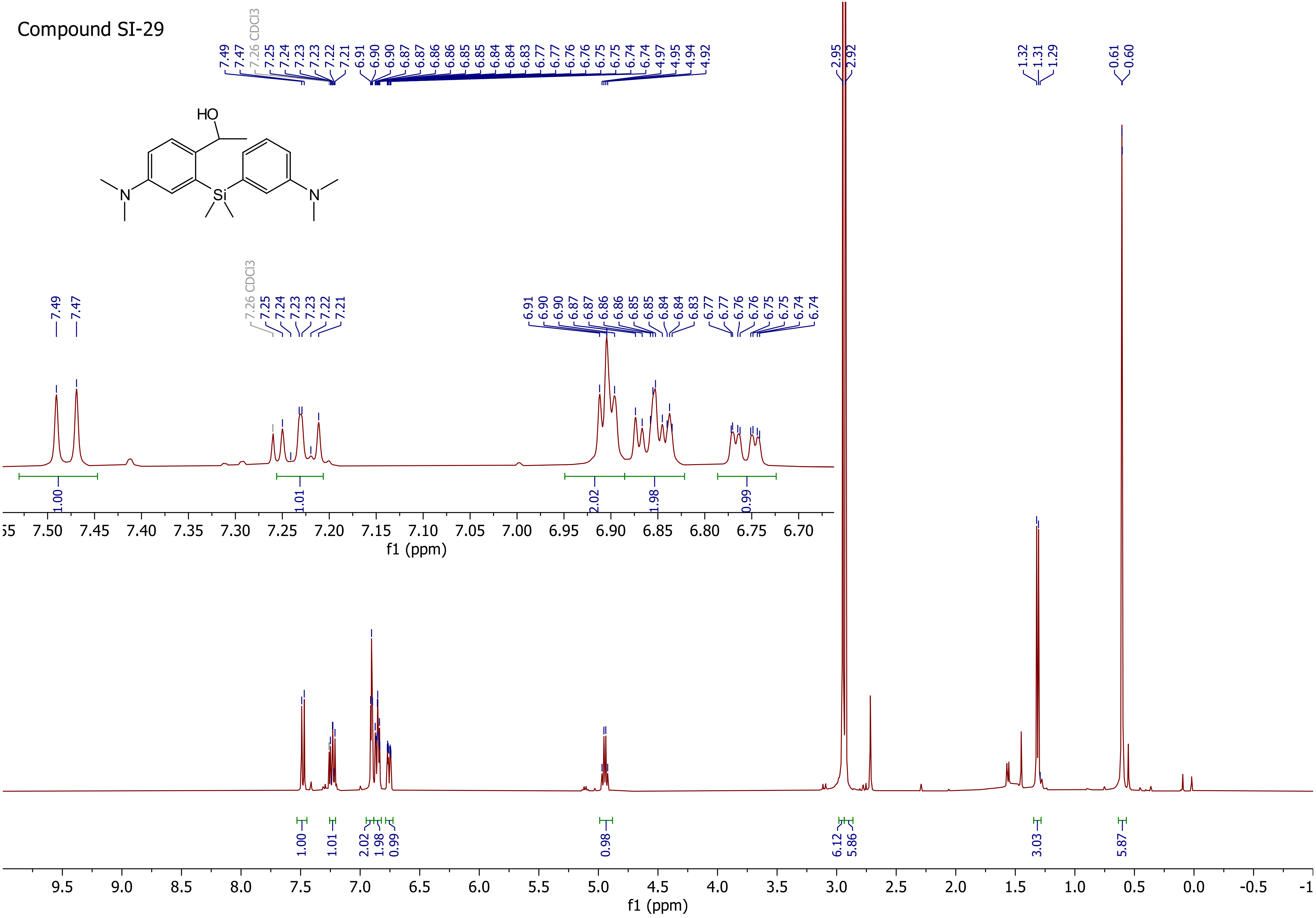

Compound SI-29

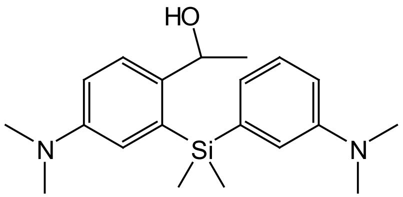

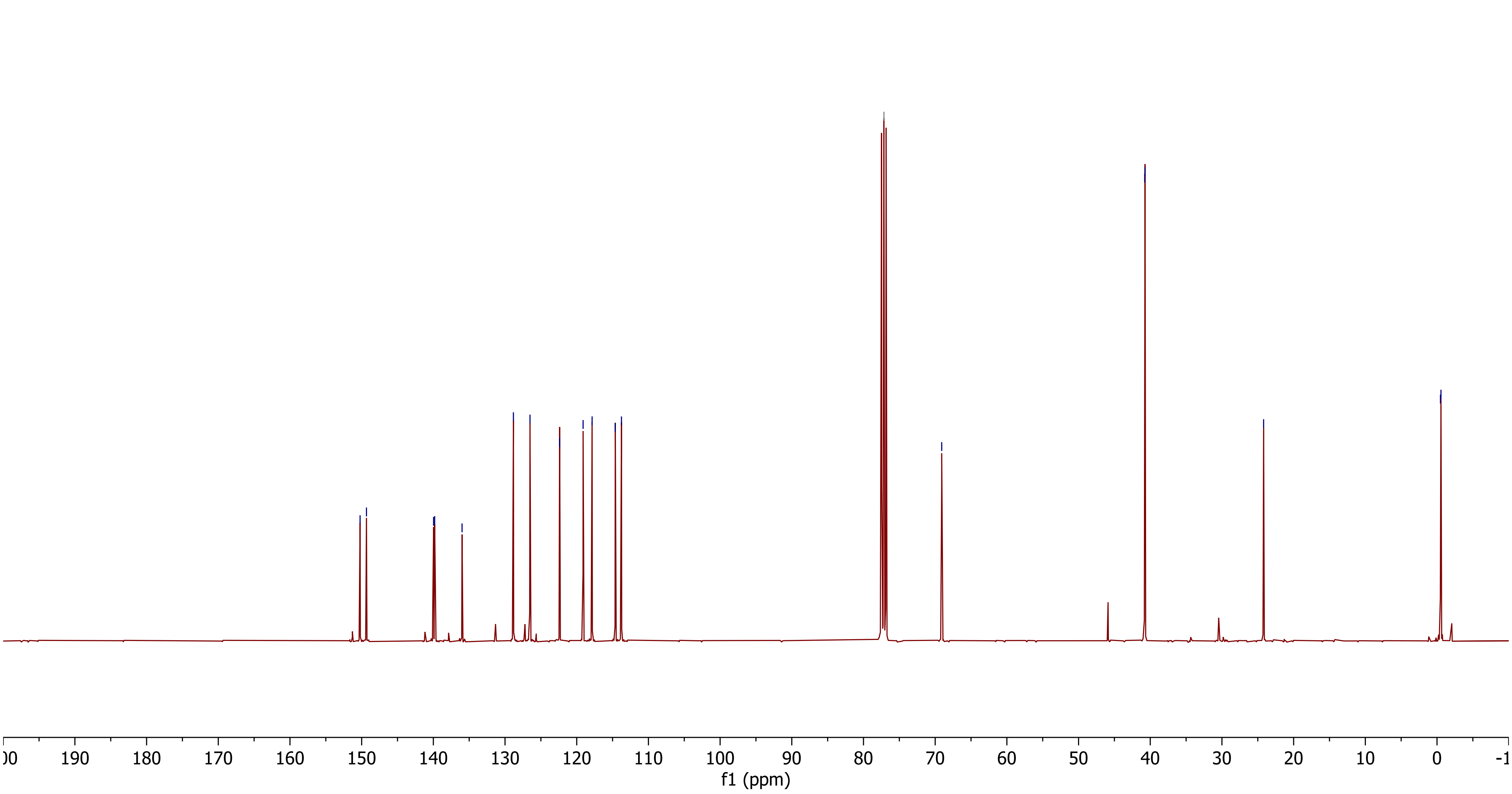

Compound SI-30

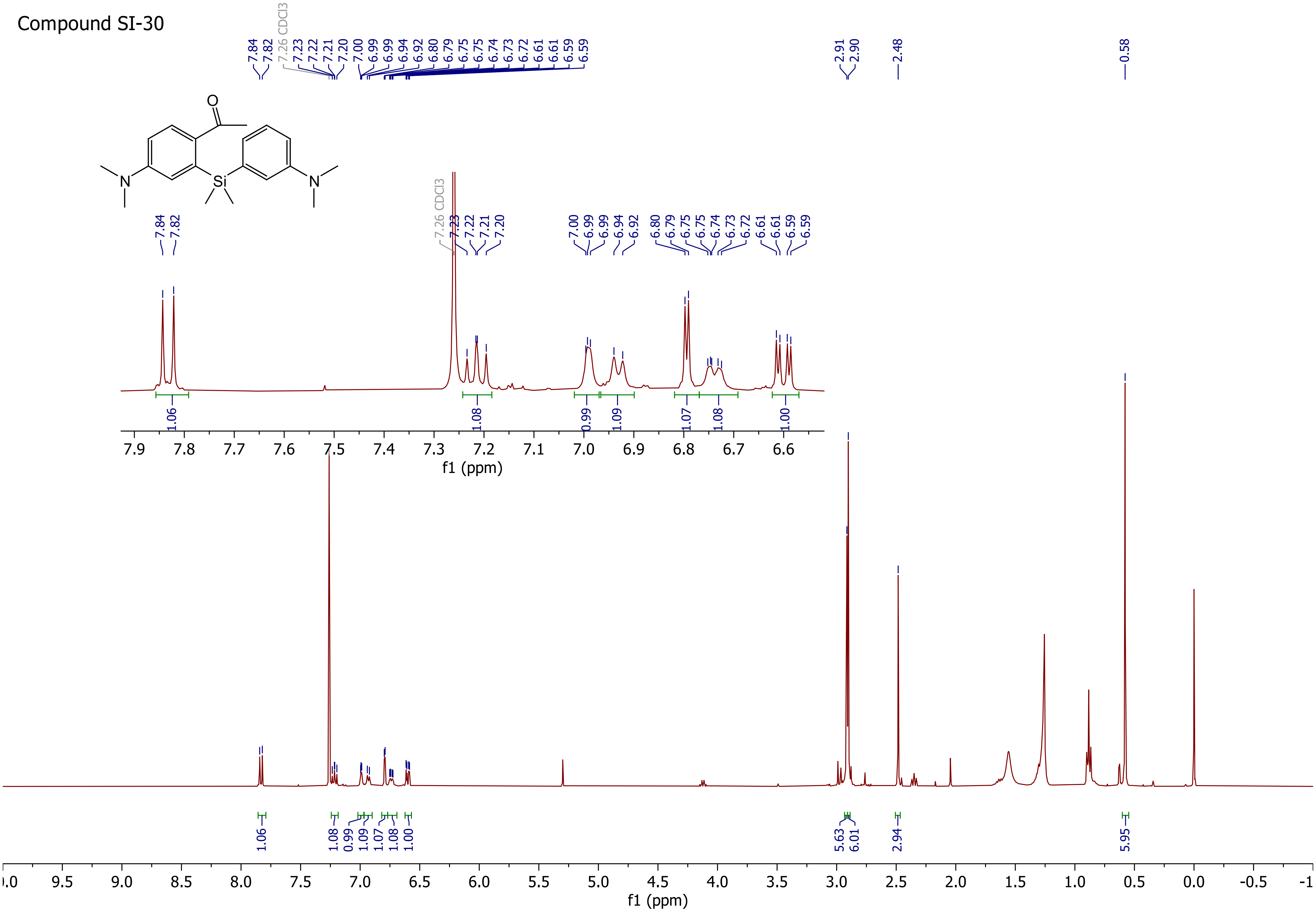

Compound SI-30

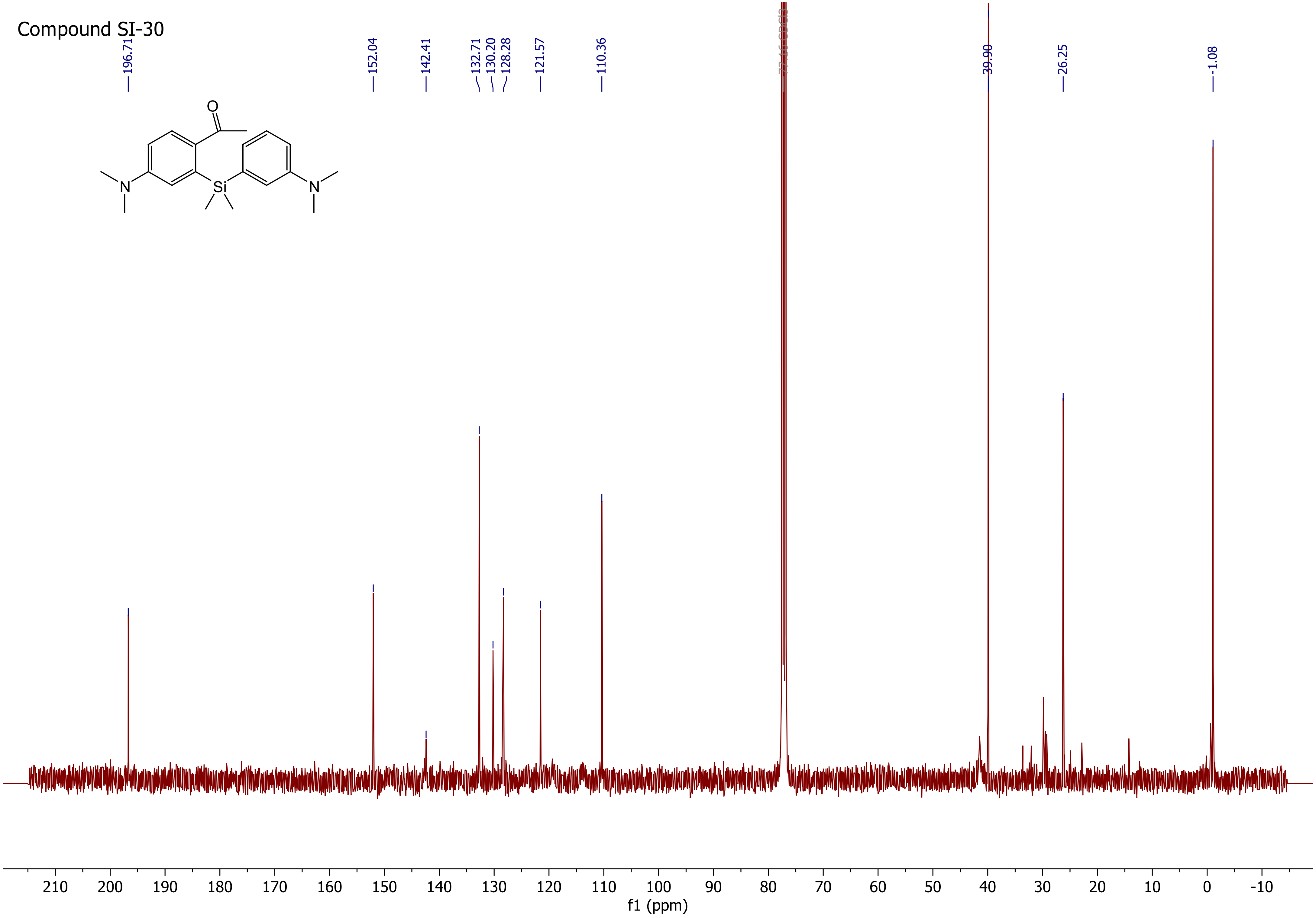

Compound SI-31

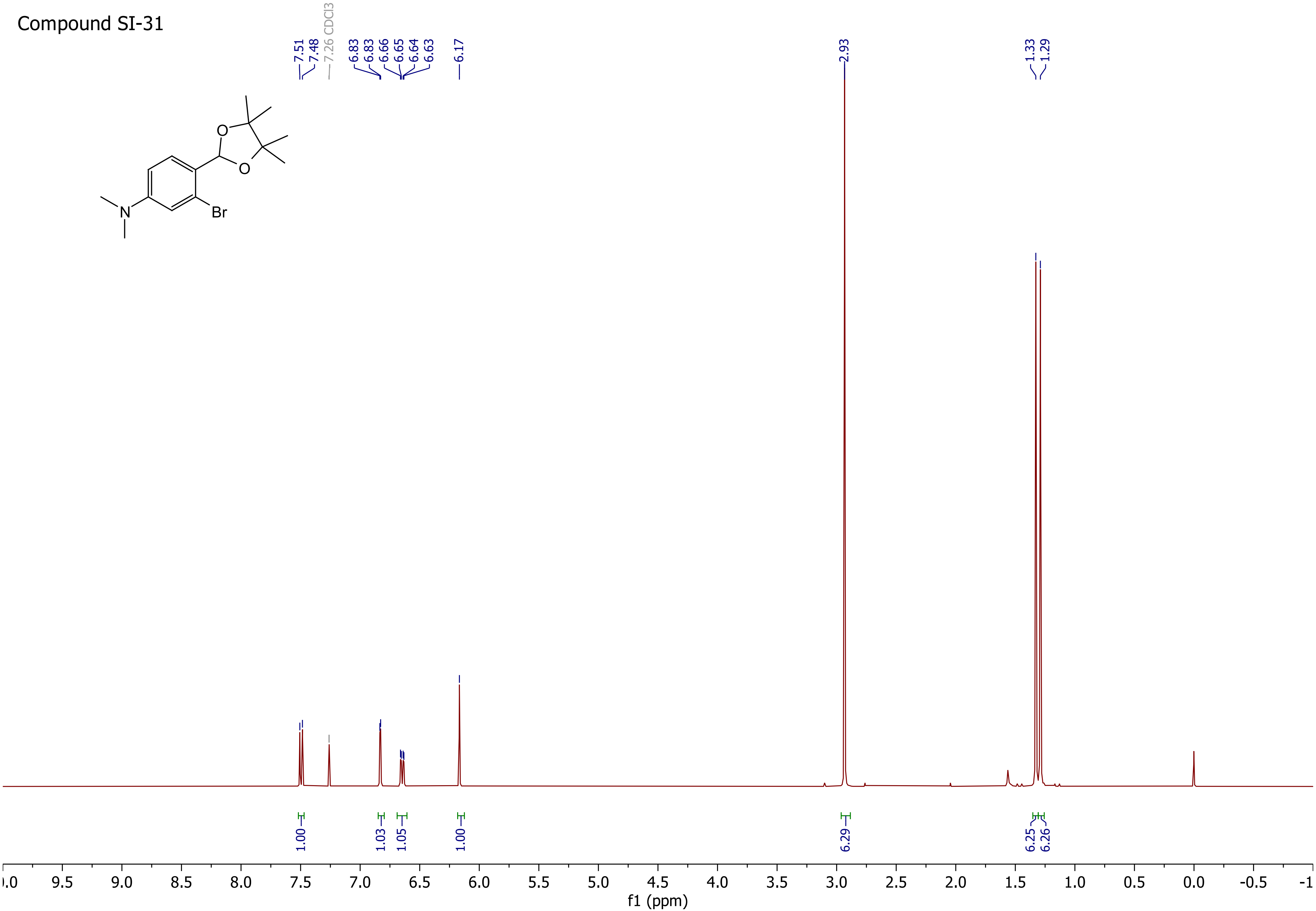

Compound SI-31

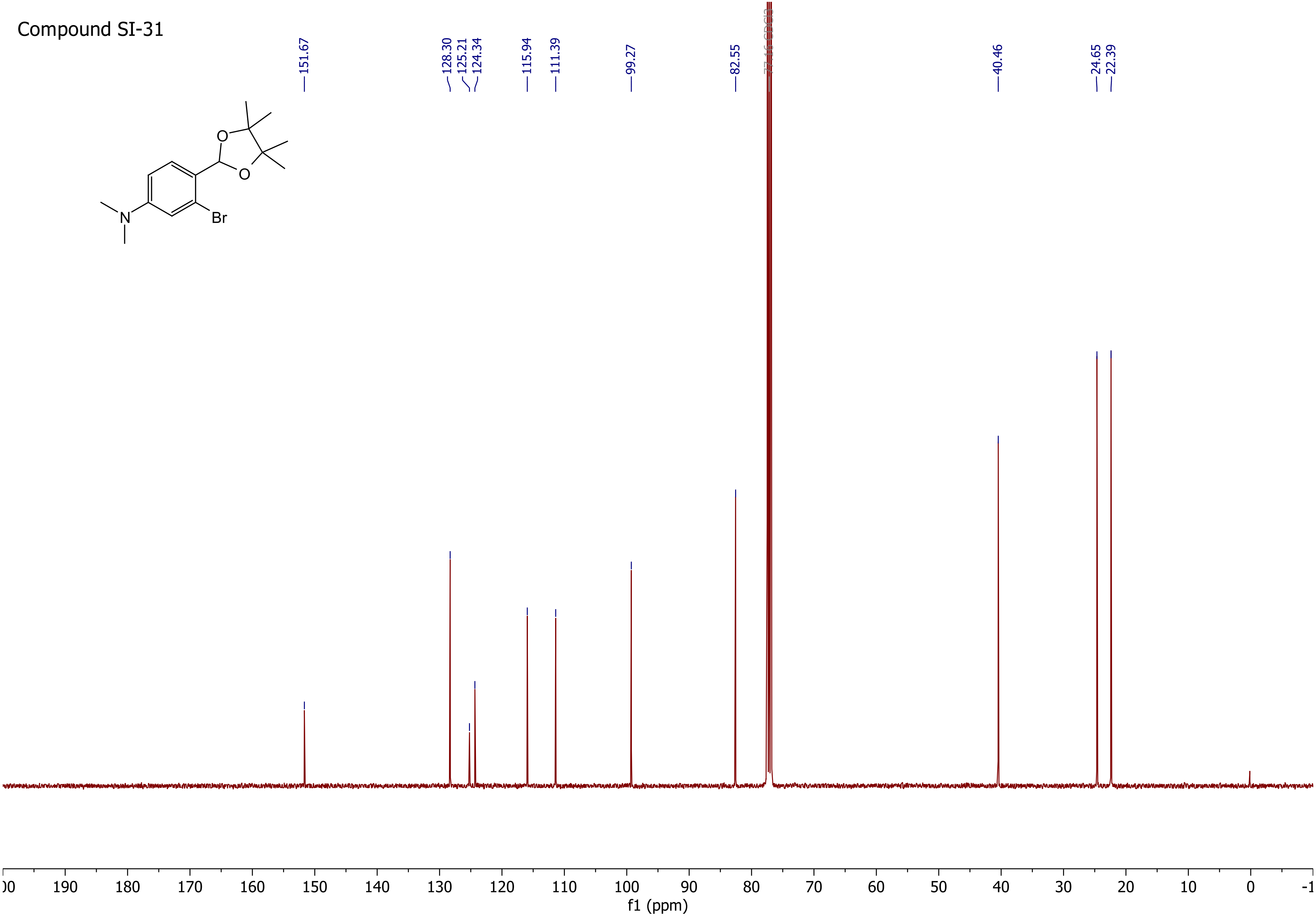

Compound SI-32

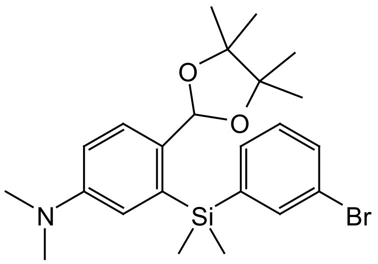

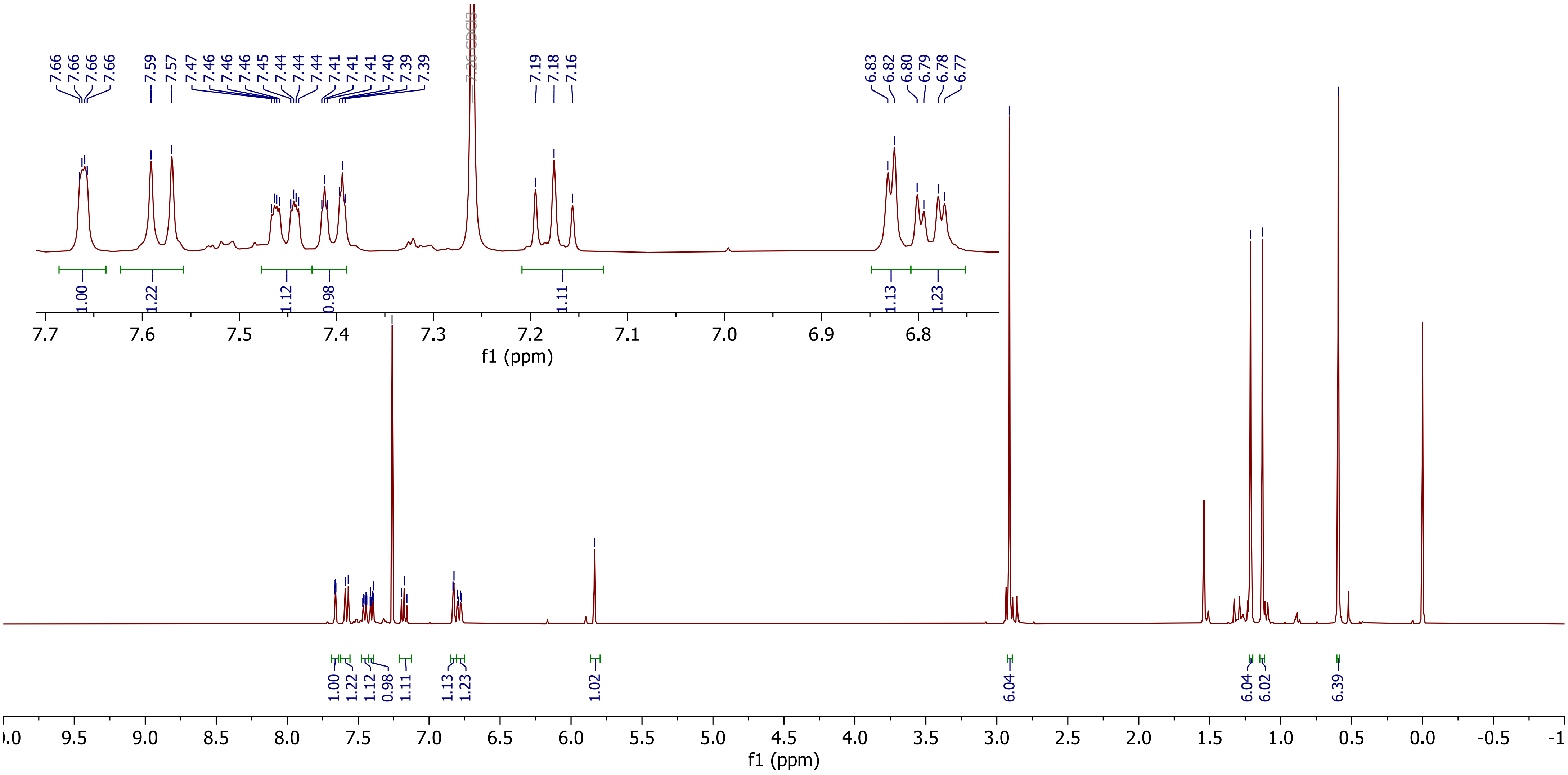

Compound SI-32

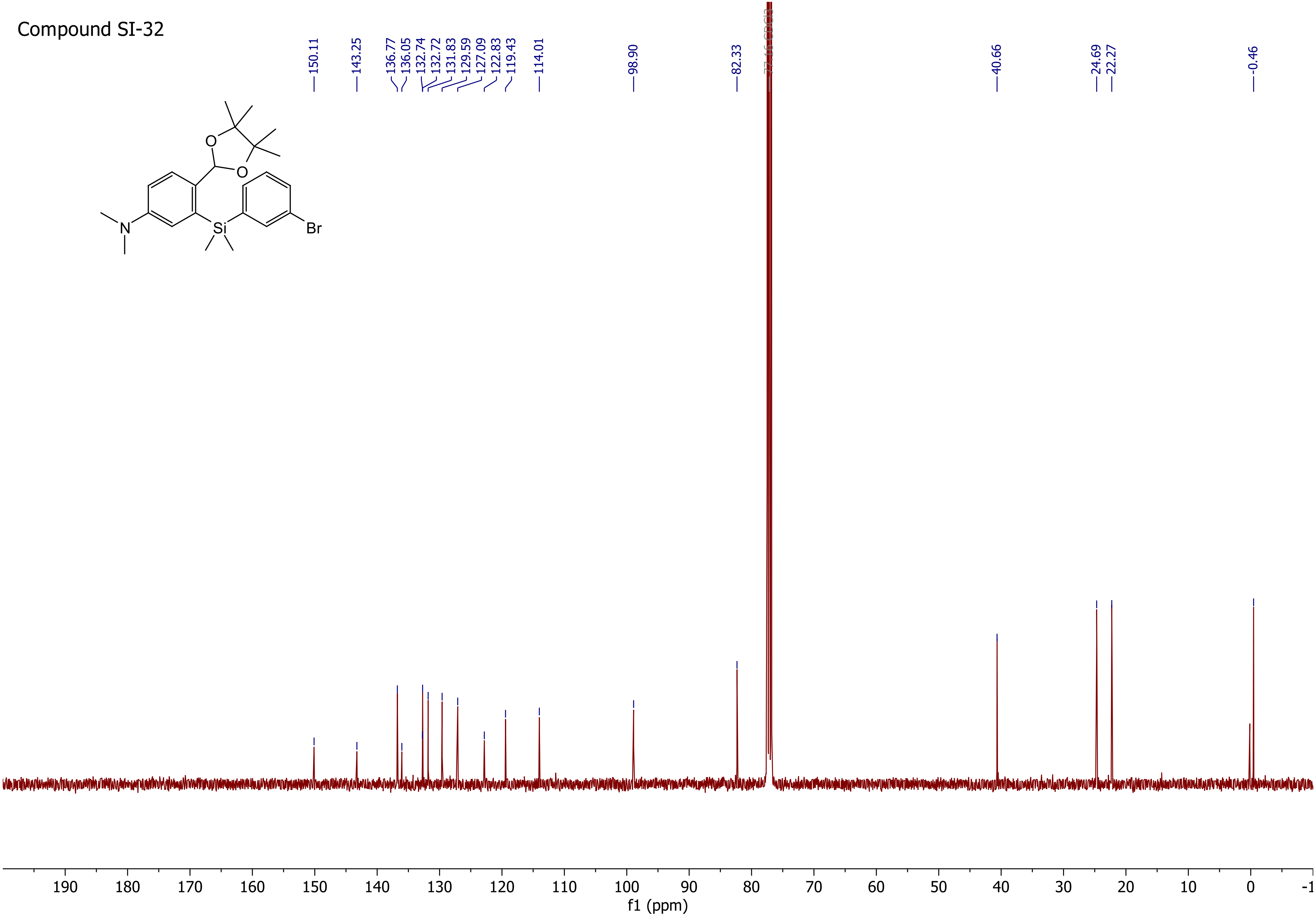

Compound SI-33

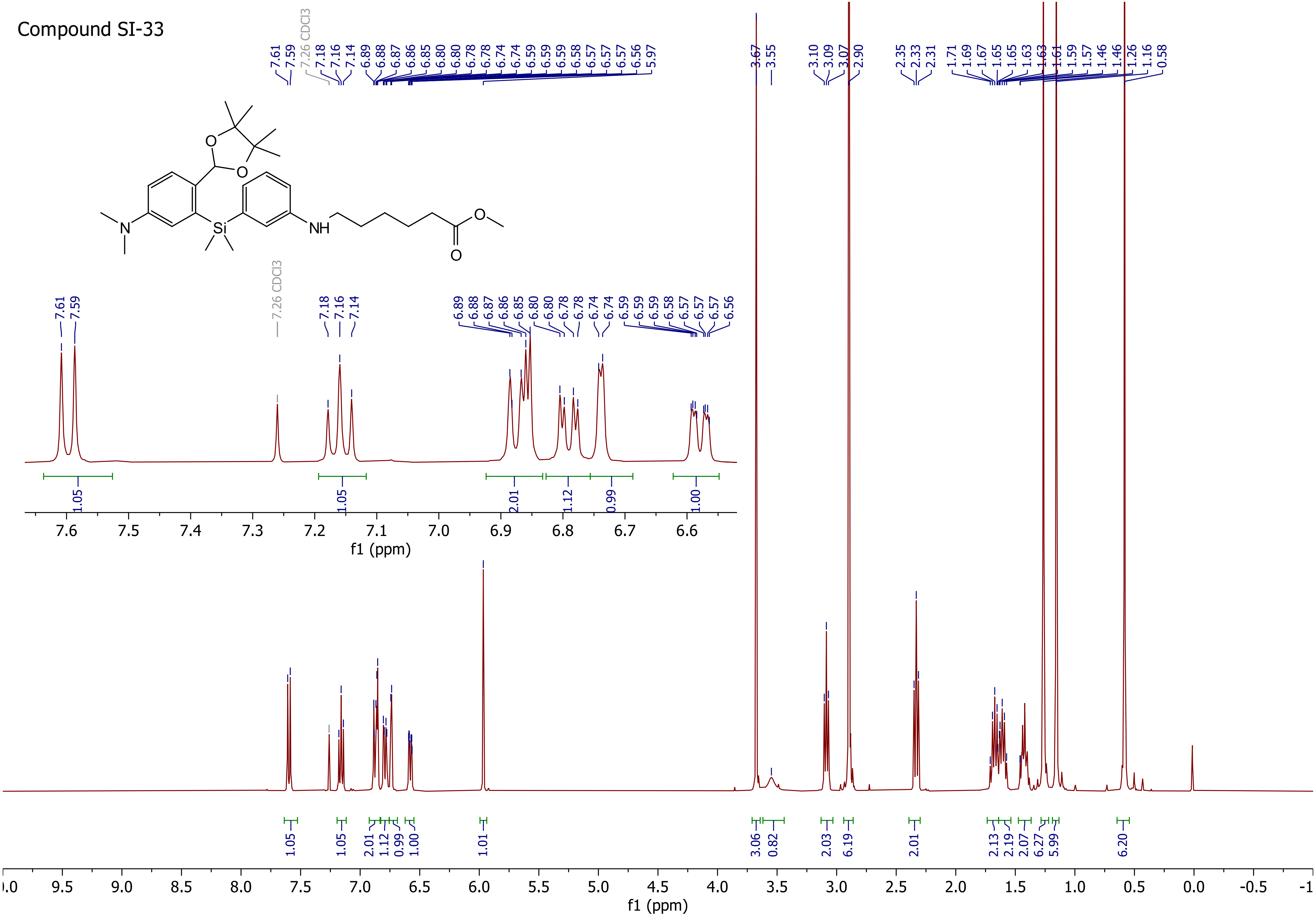

Compound SI-33

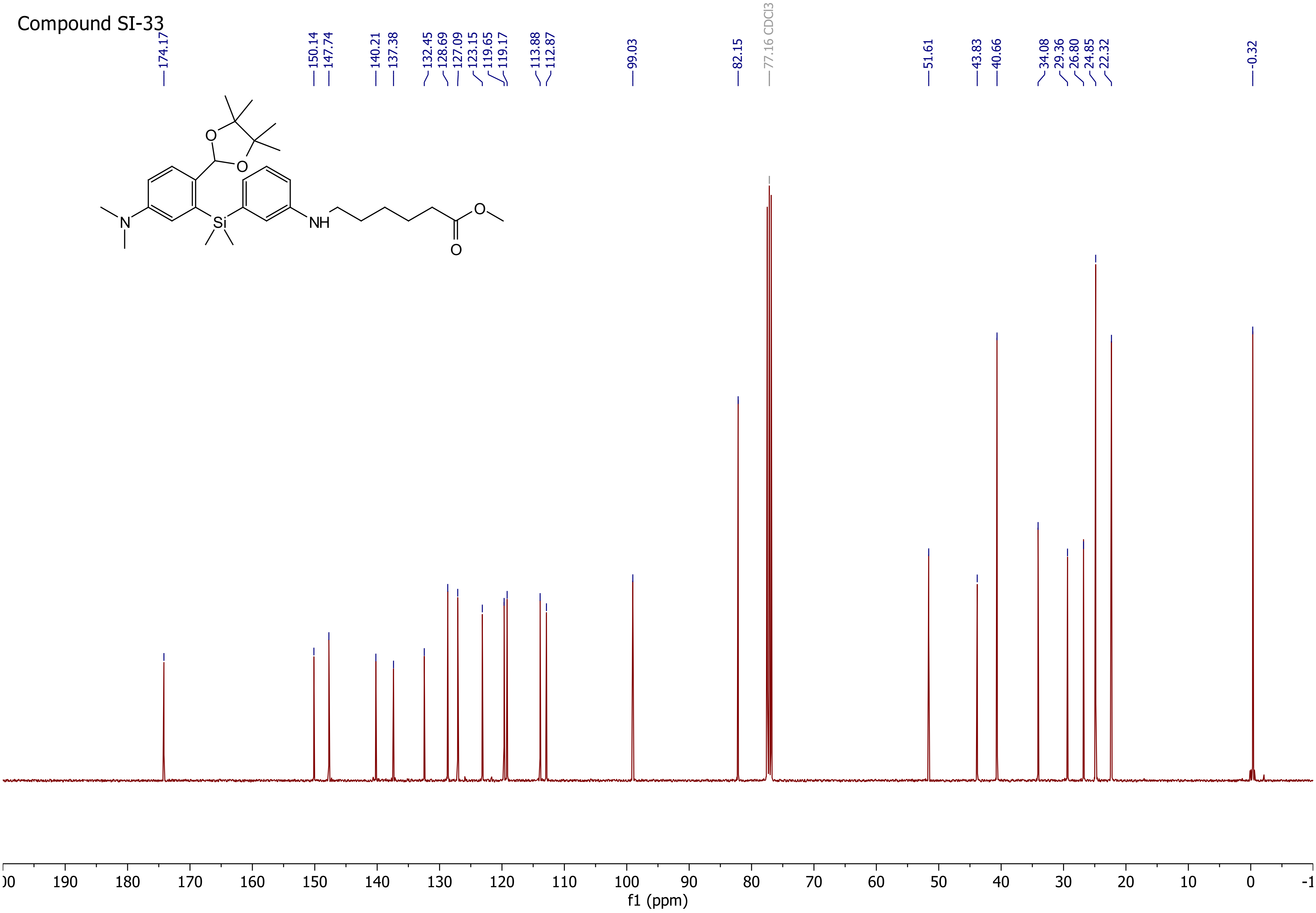

Compound SI-34

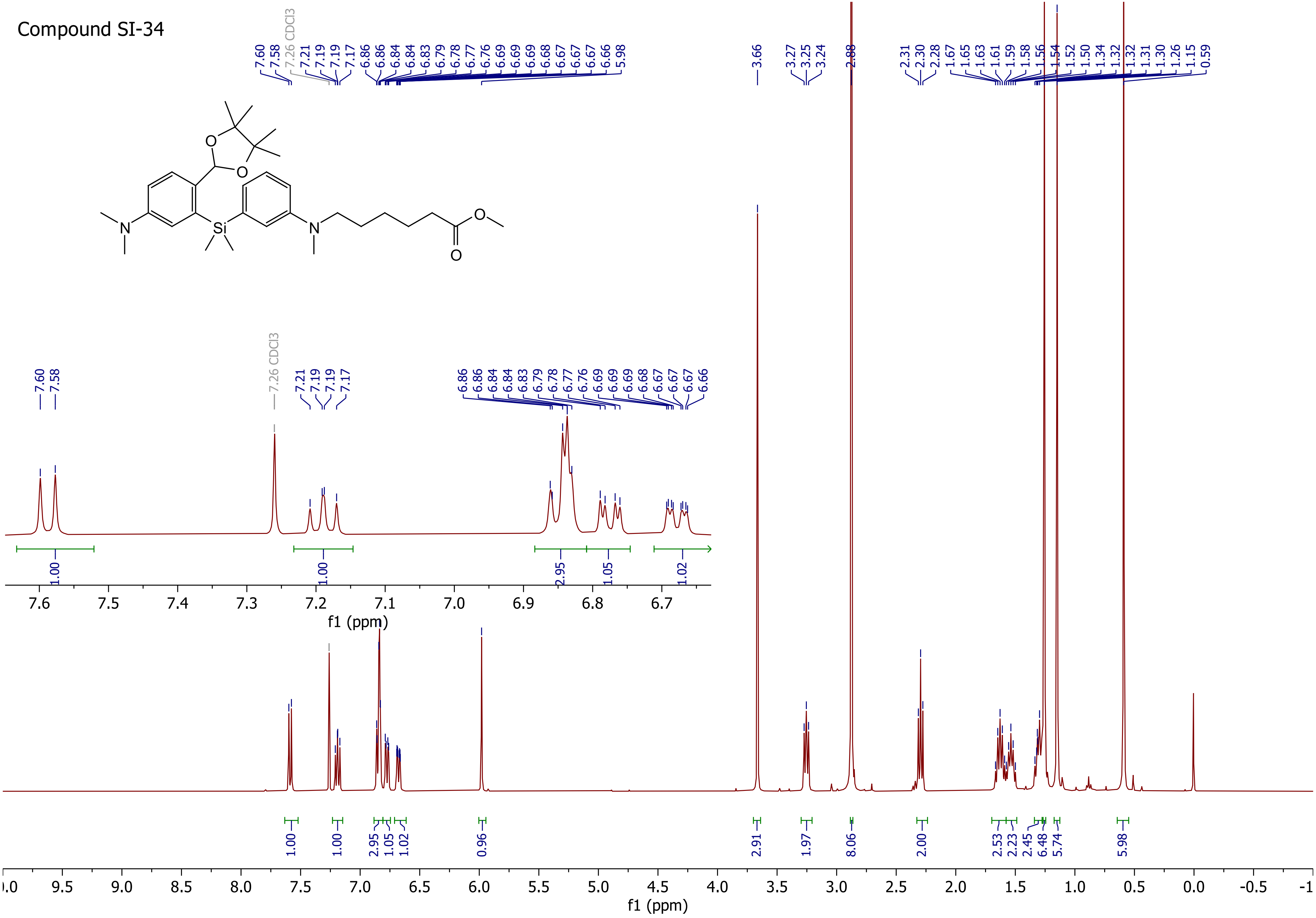

Compound SI-34

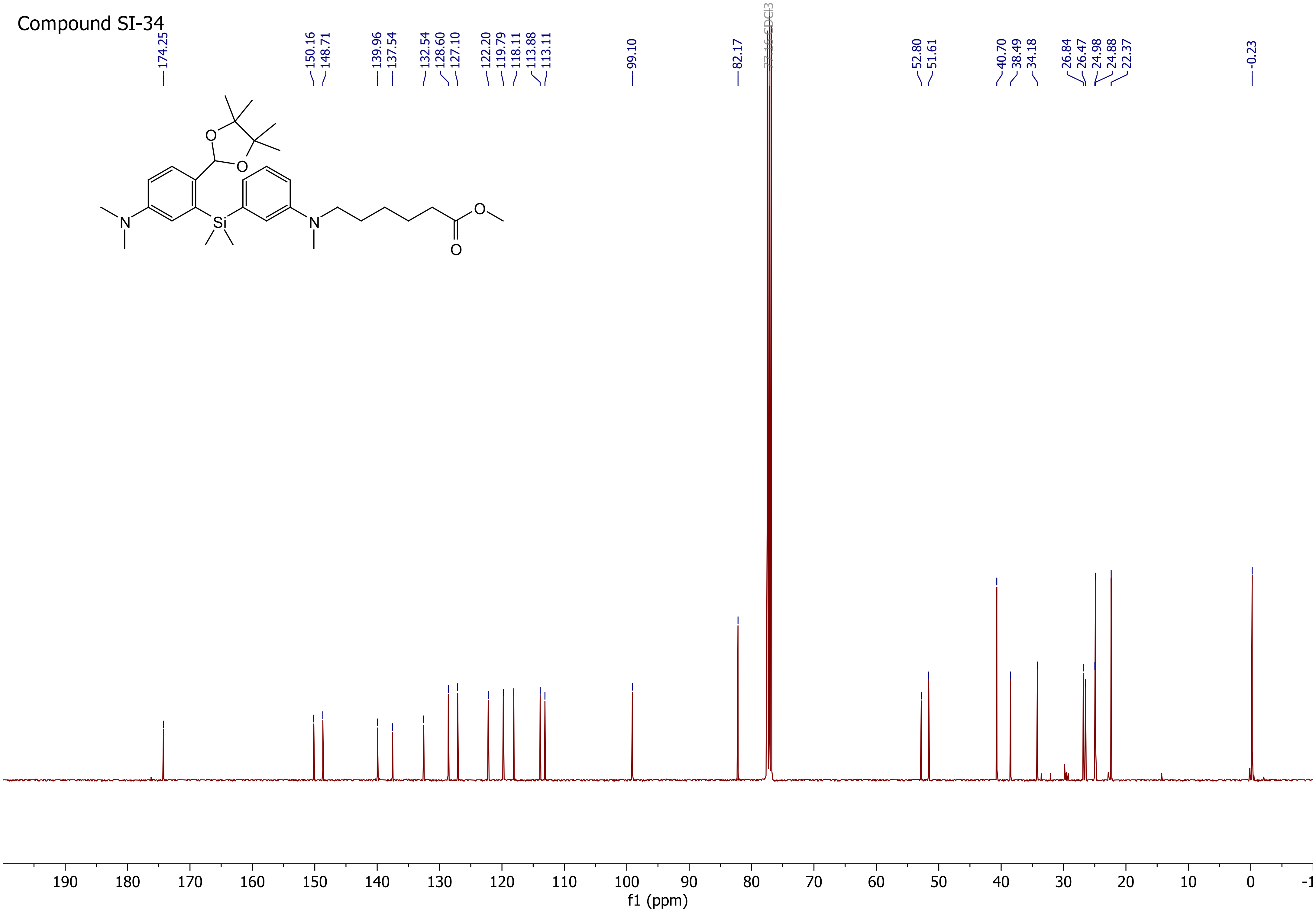

Compound SI-35

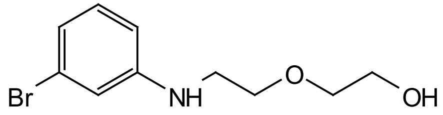

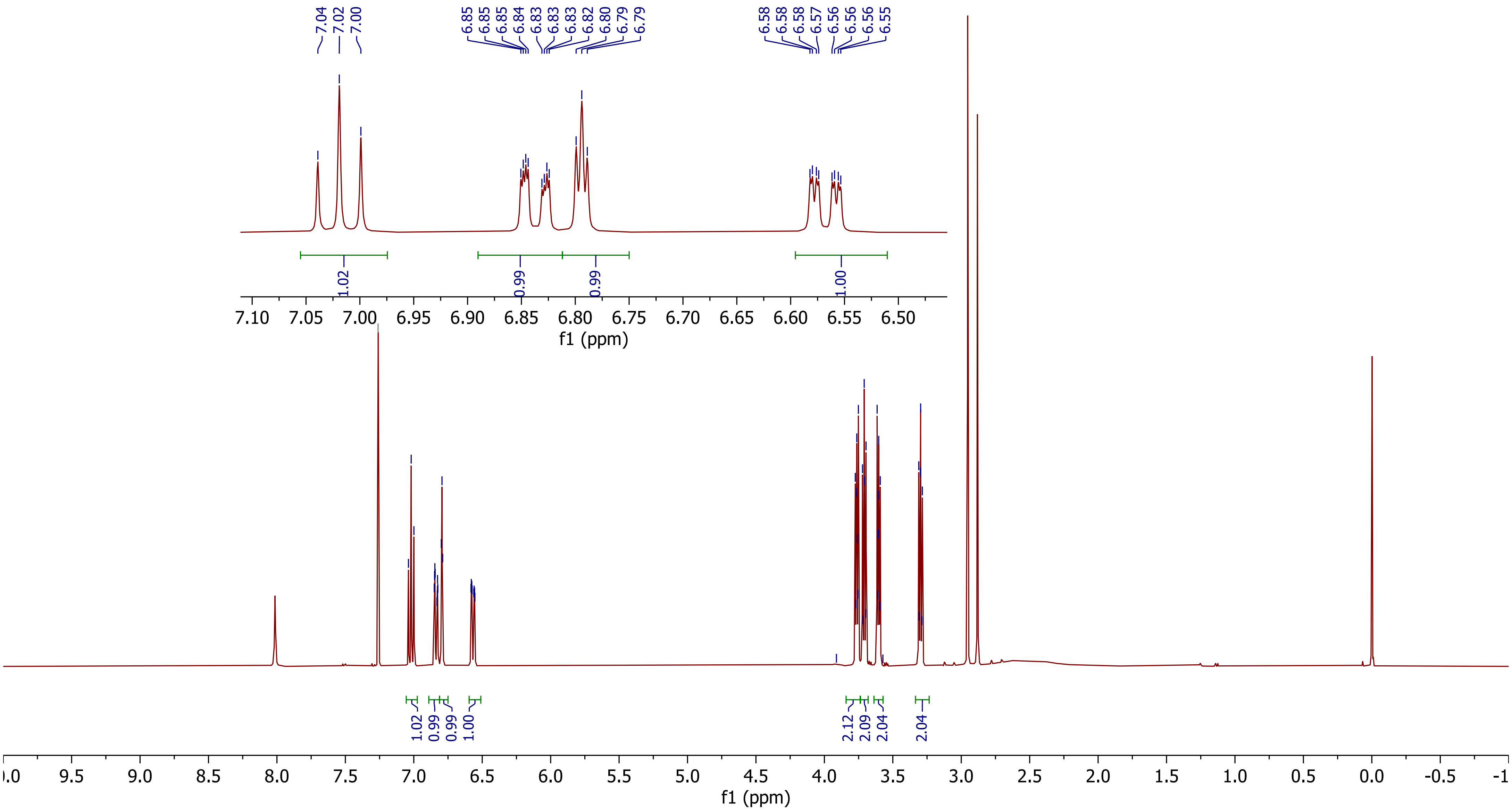

Compound SI-35

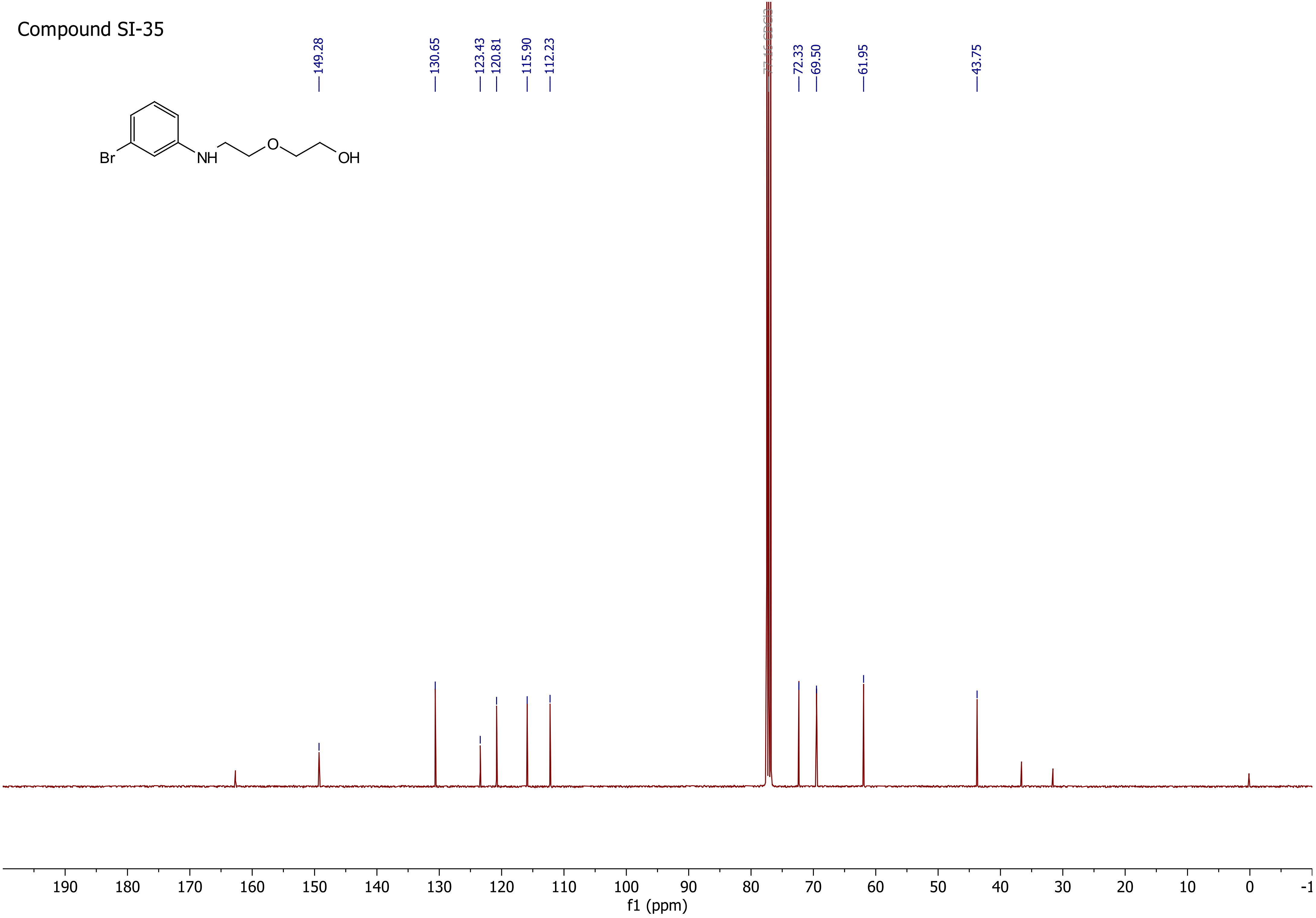

Compound SI-36

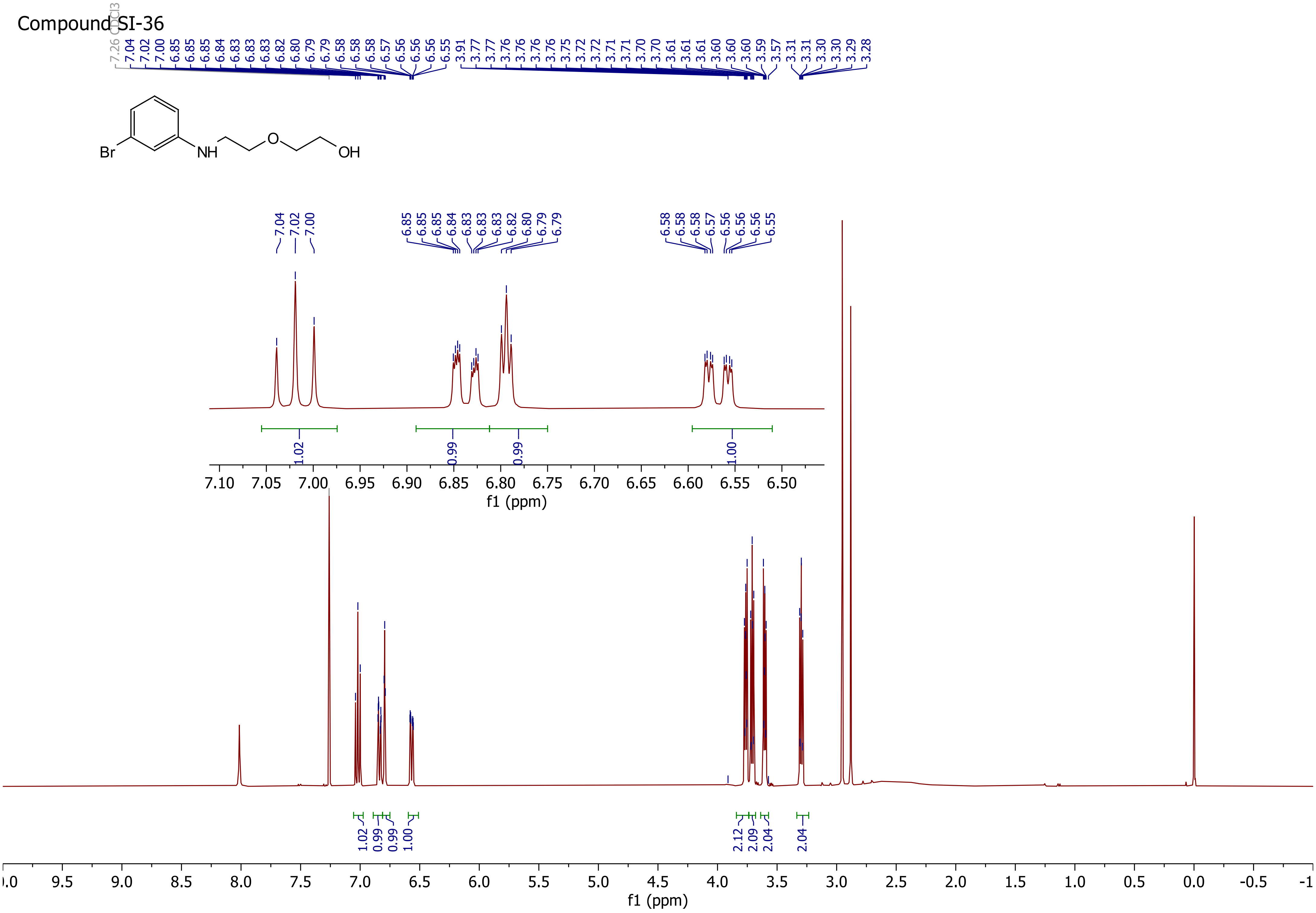

Compound SI-36

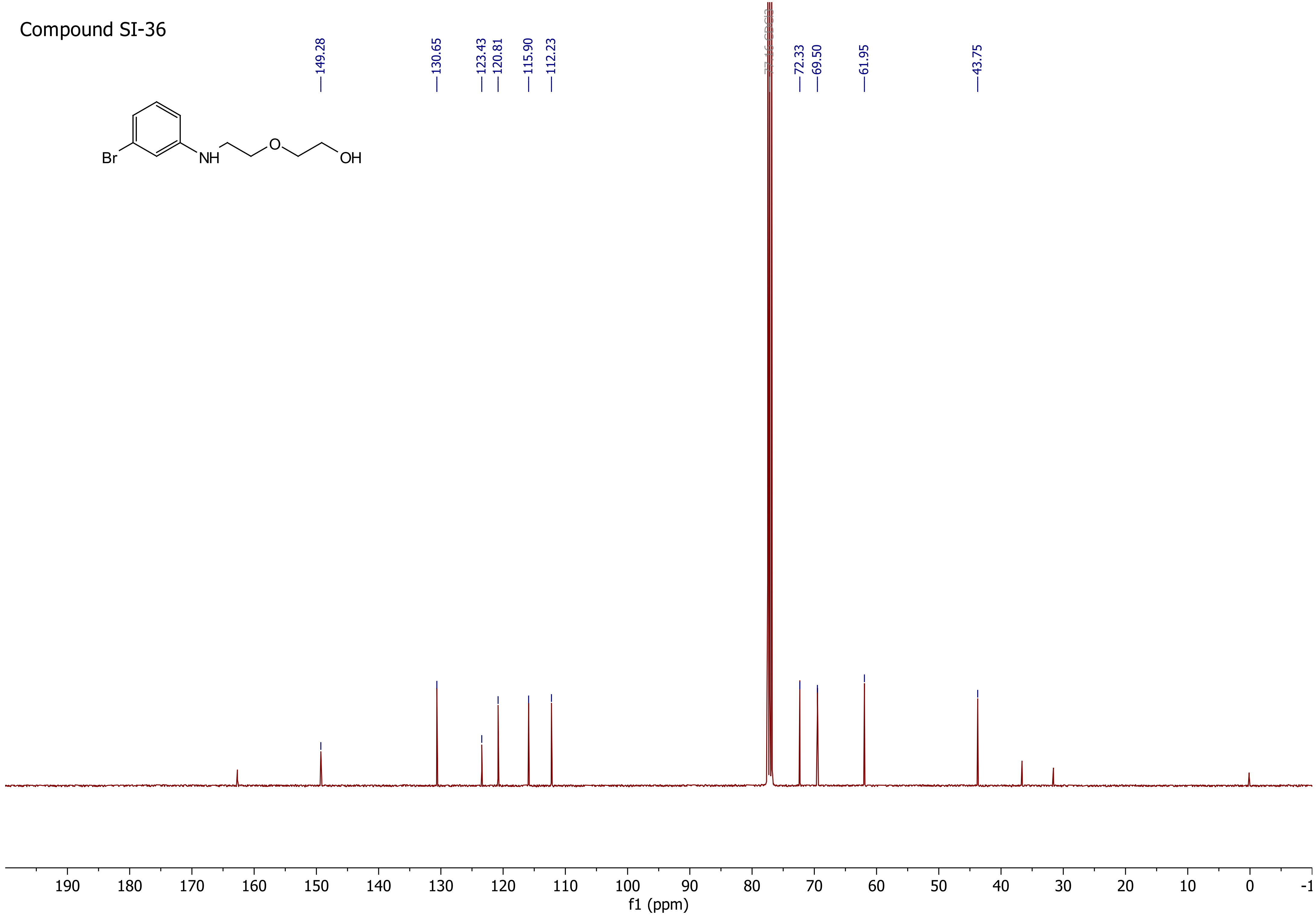

Compound SI-37

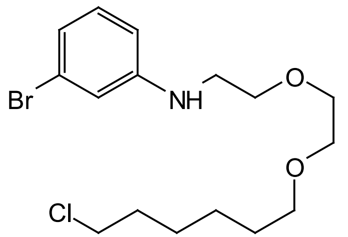

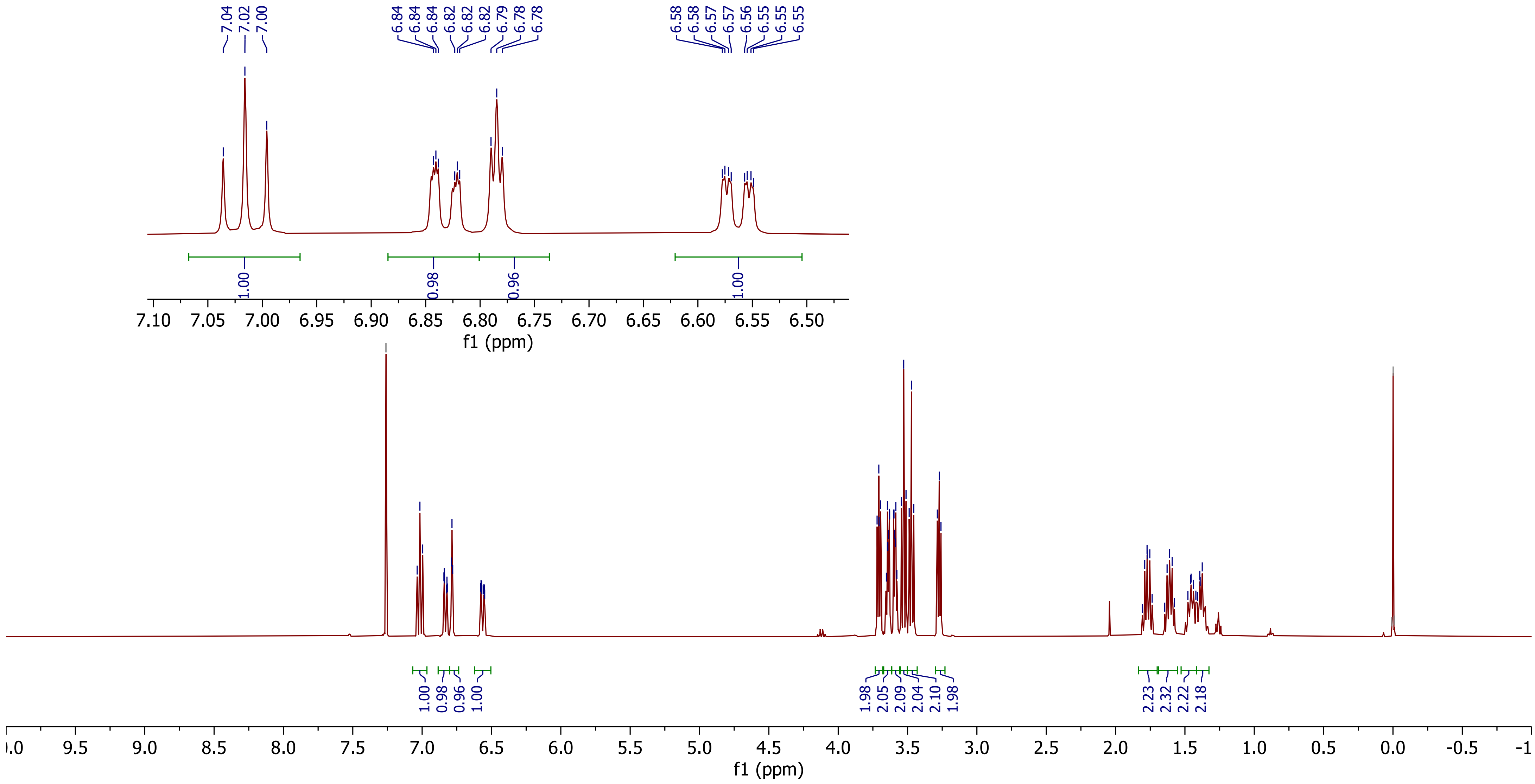

Compound SI-37

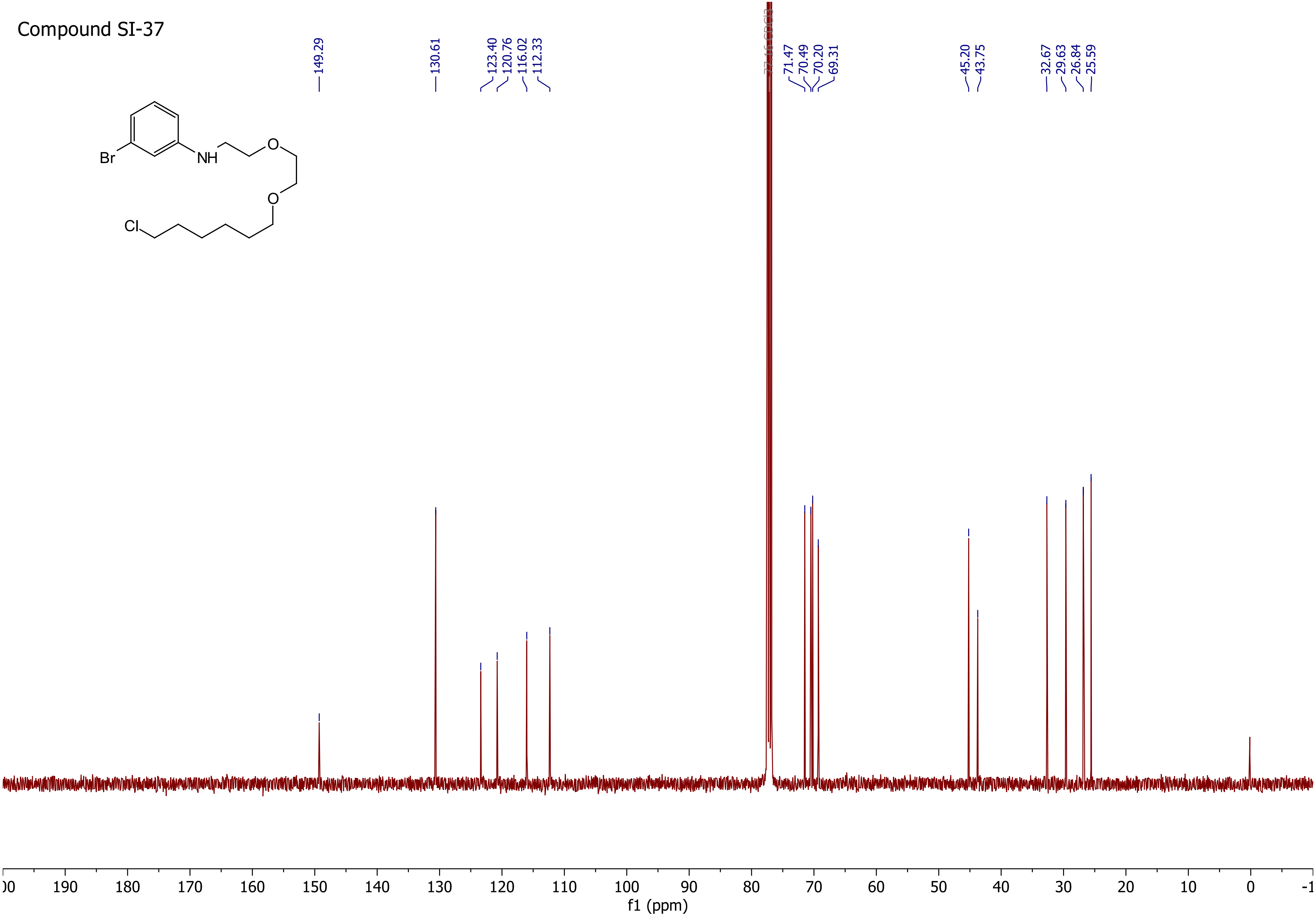

Compound SI-38

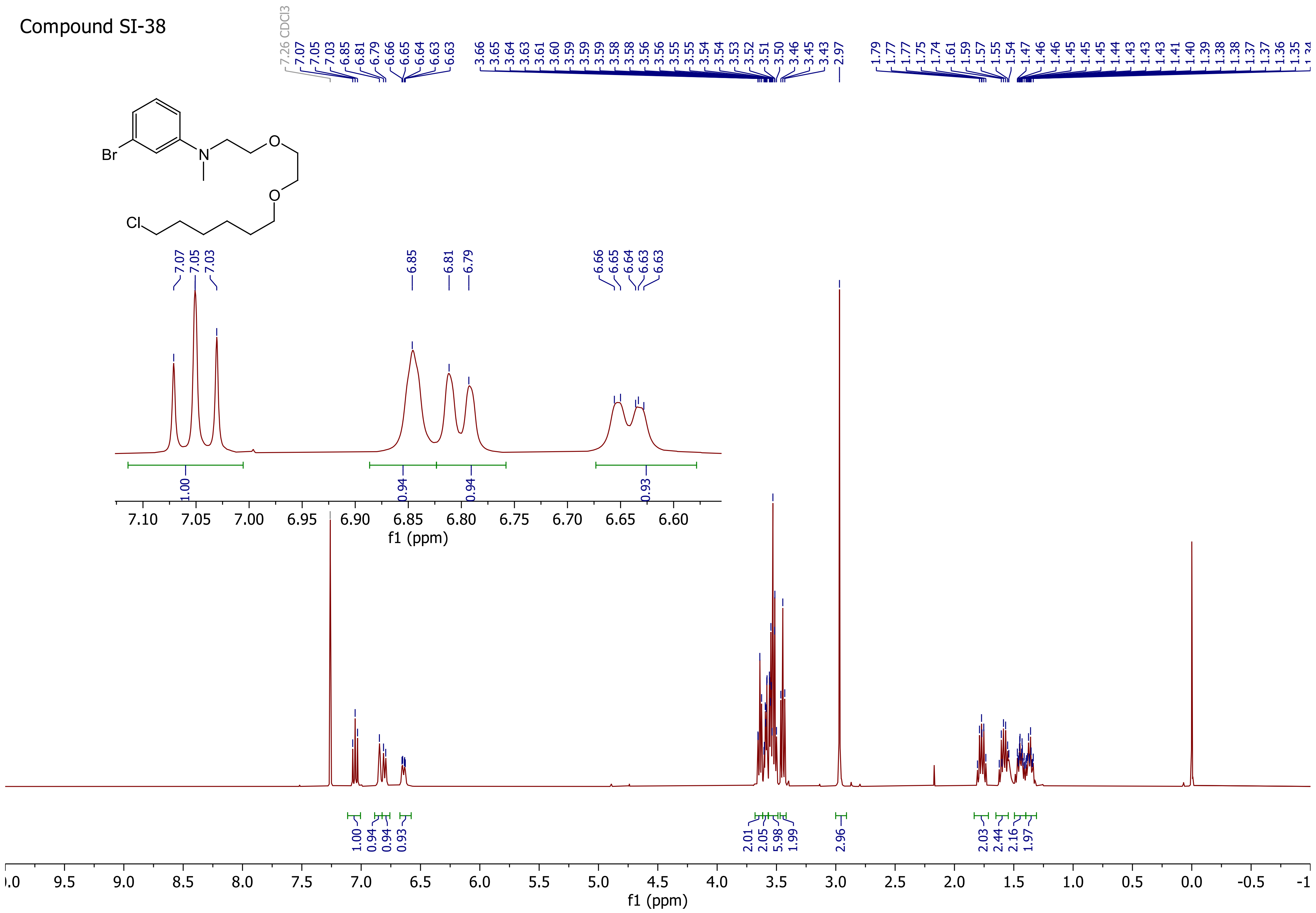

Compound SI-38

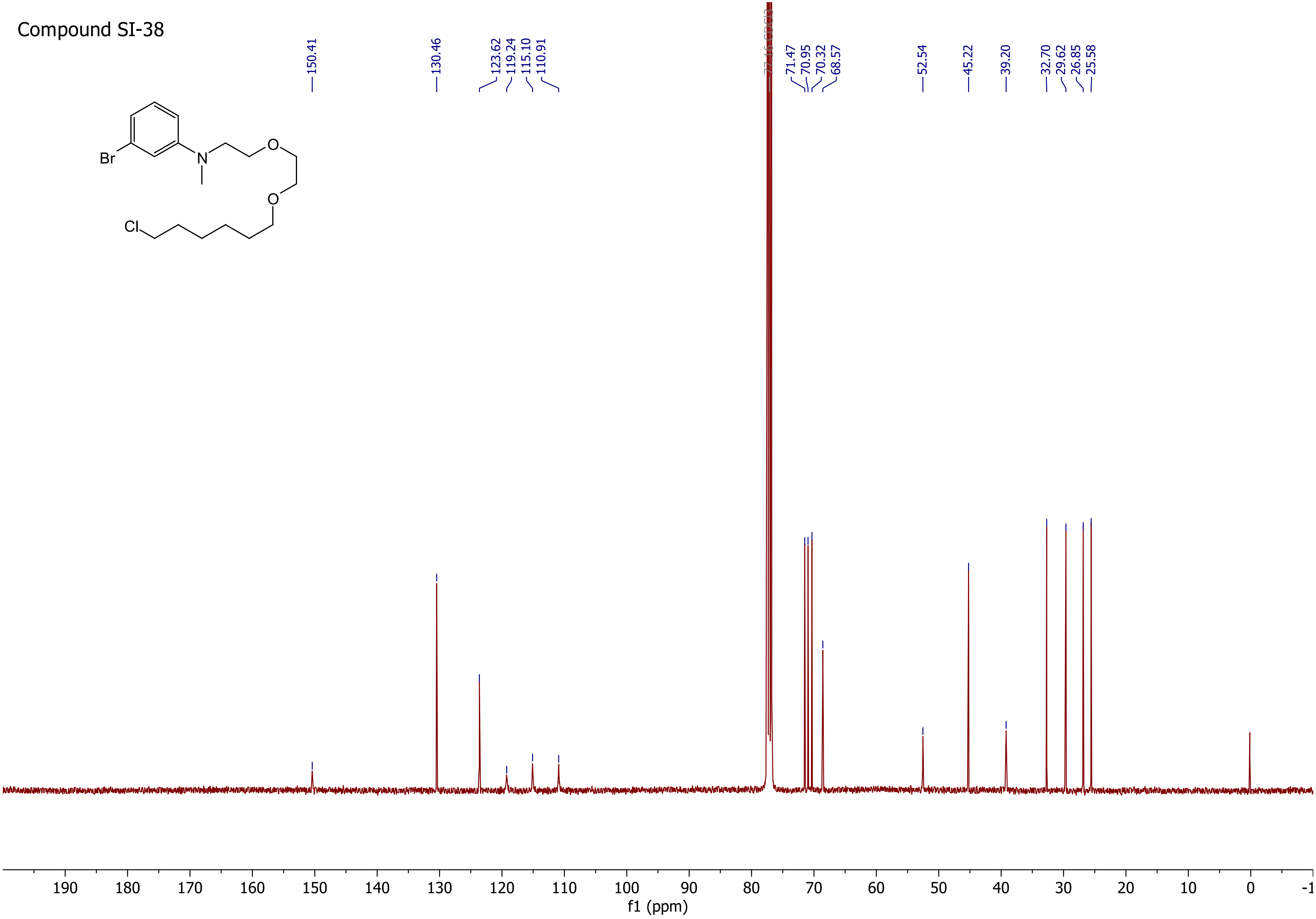

Compound SI-39

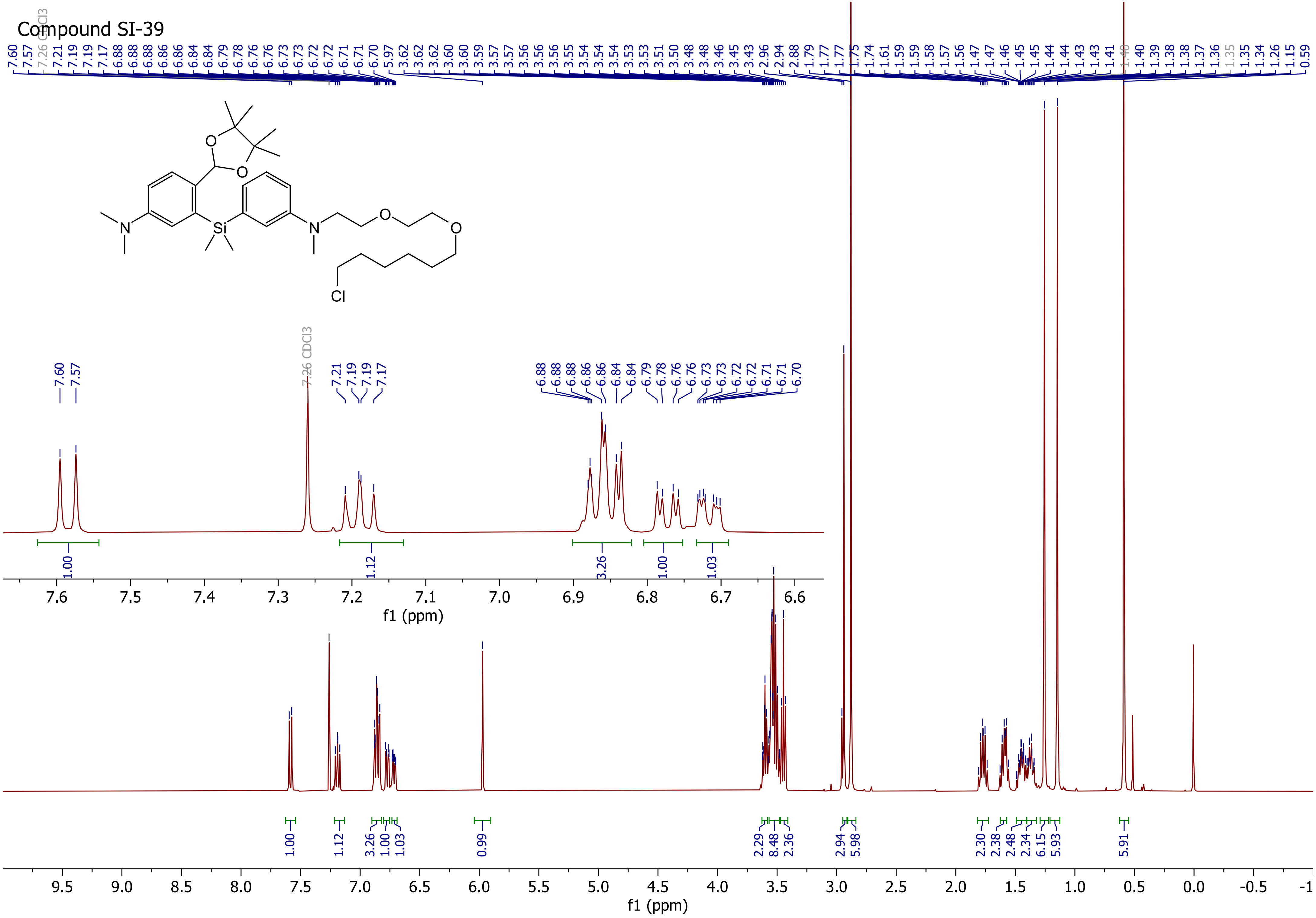

Compound SI-39

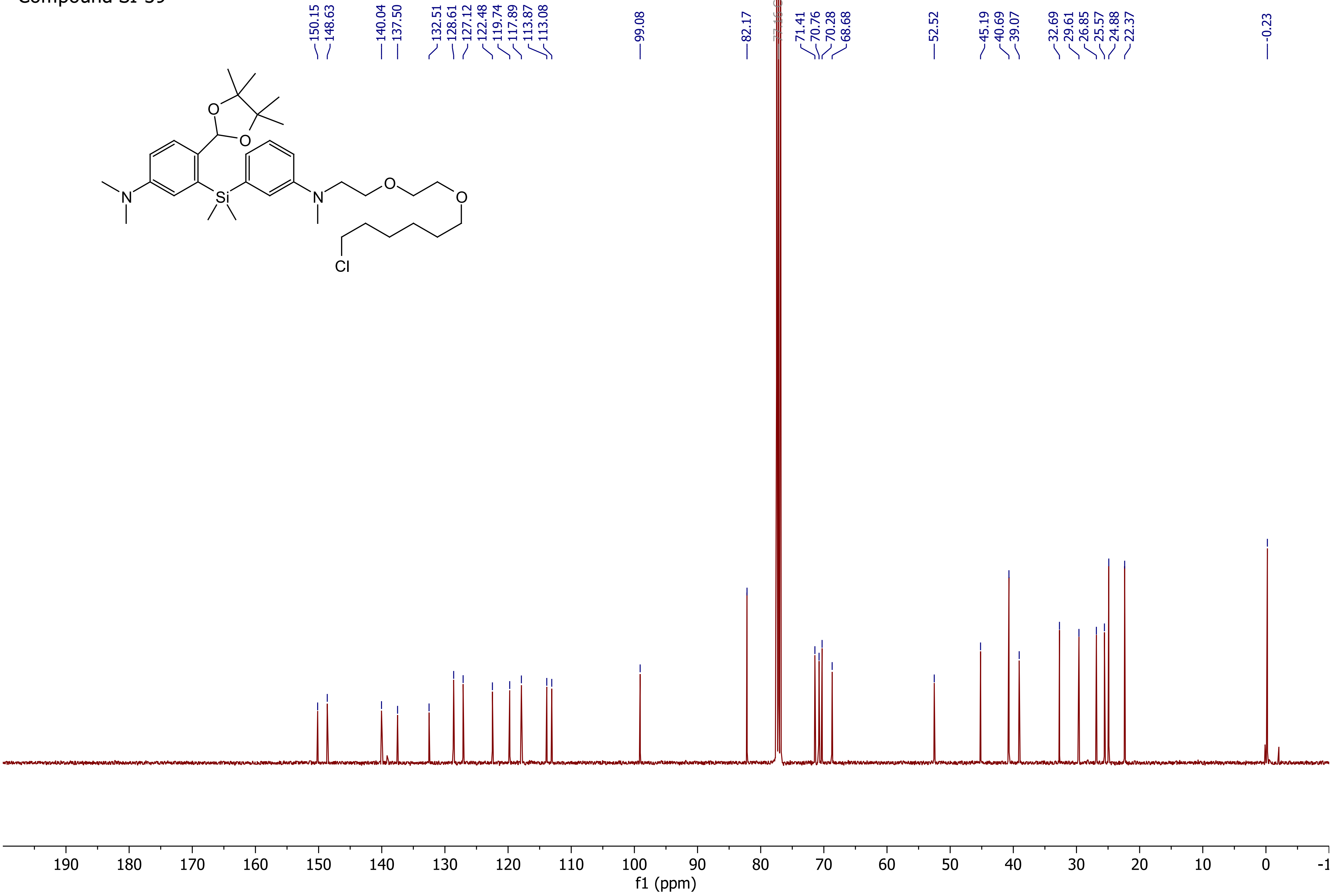

Compound SI-40

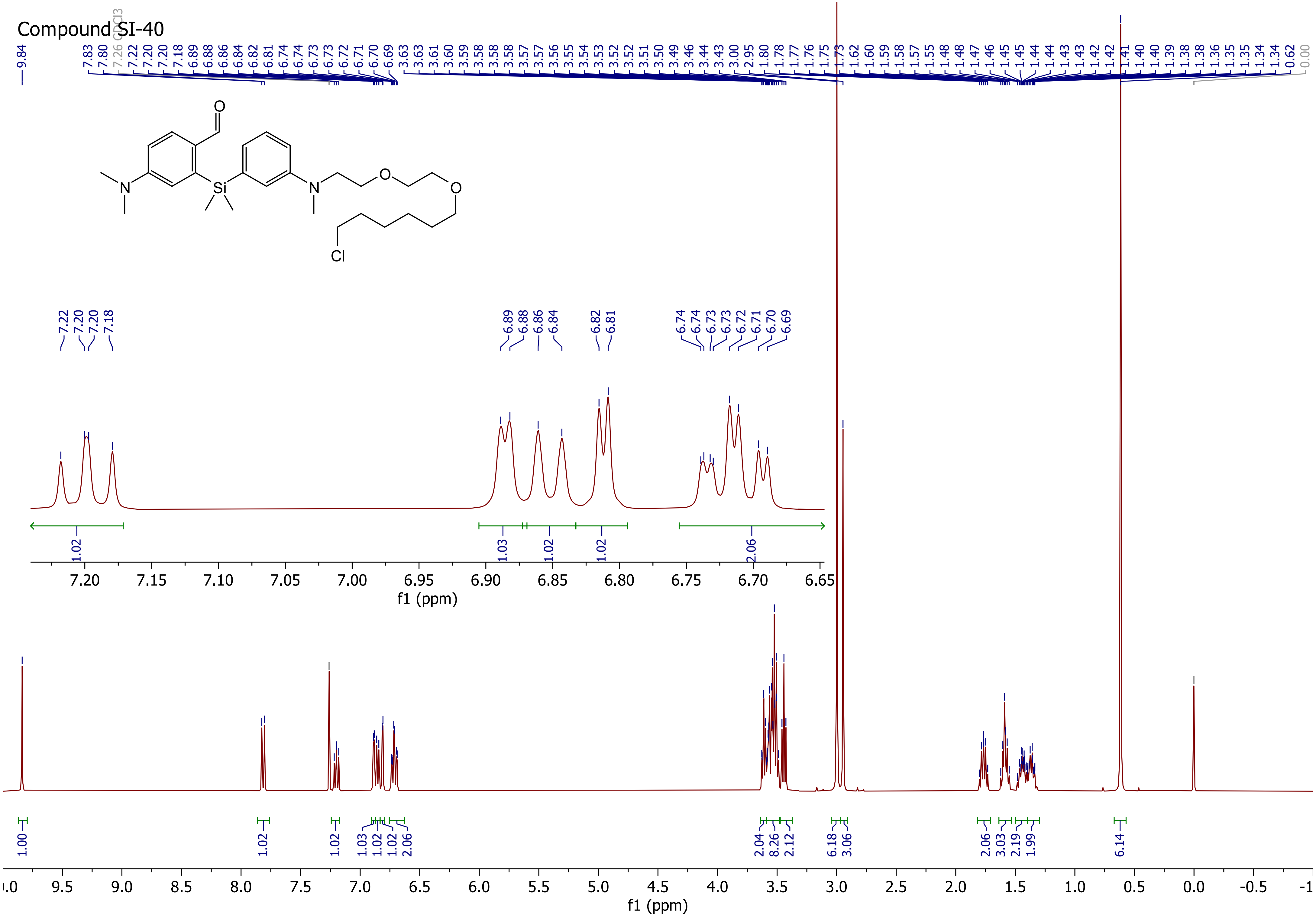

Compound SI-40

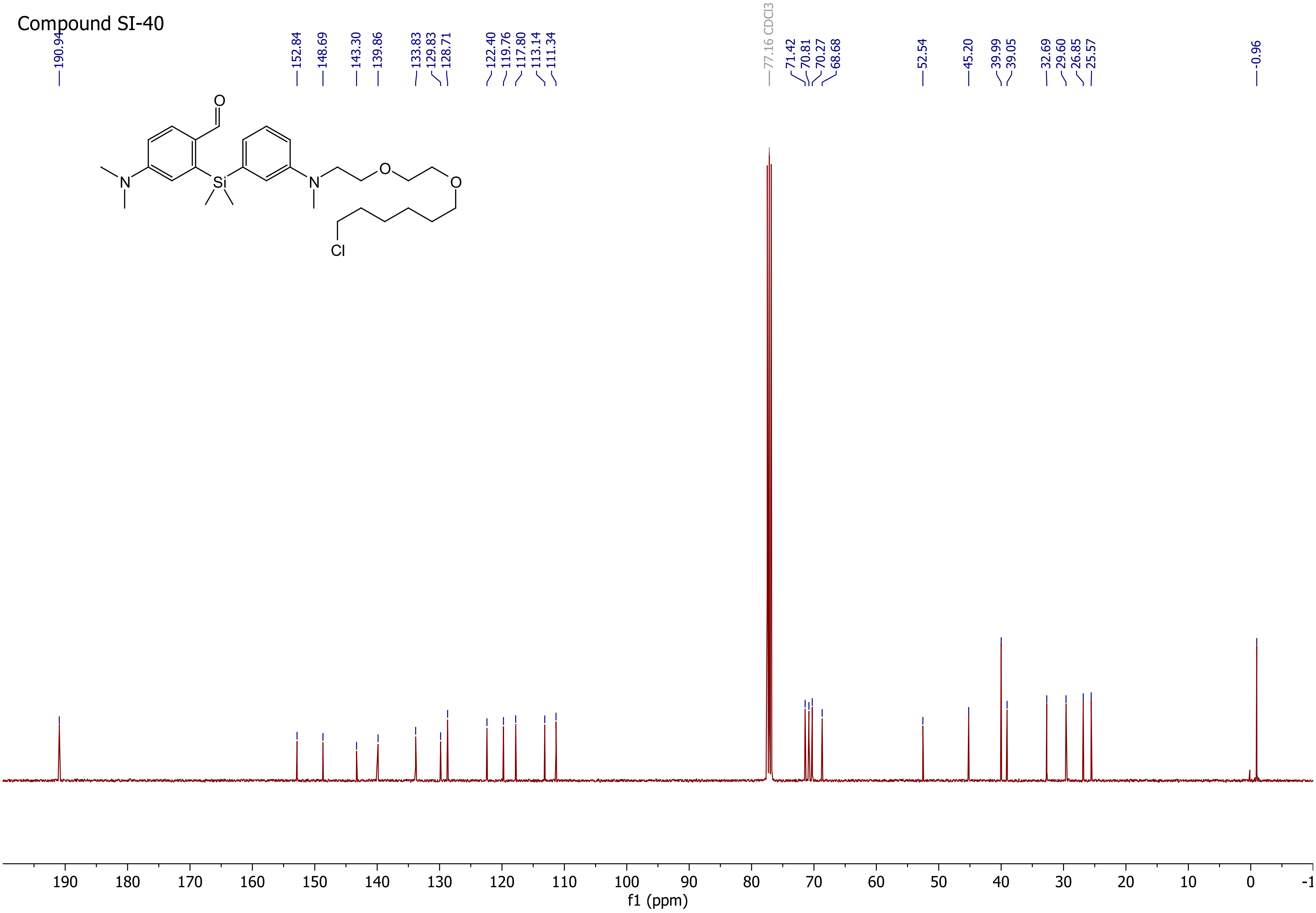

Compound SI-41

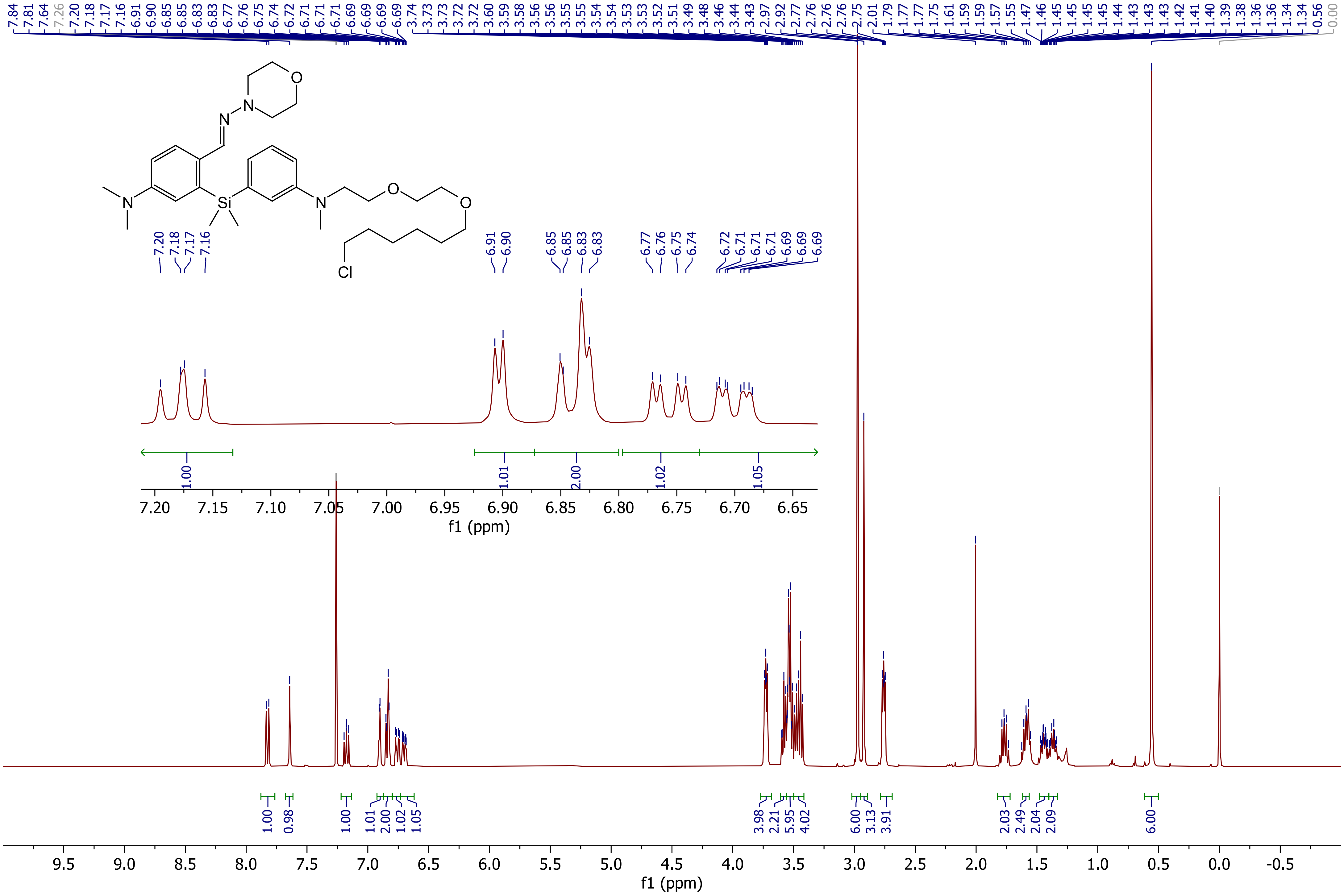

Compound SI-41

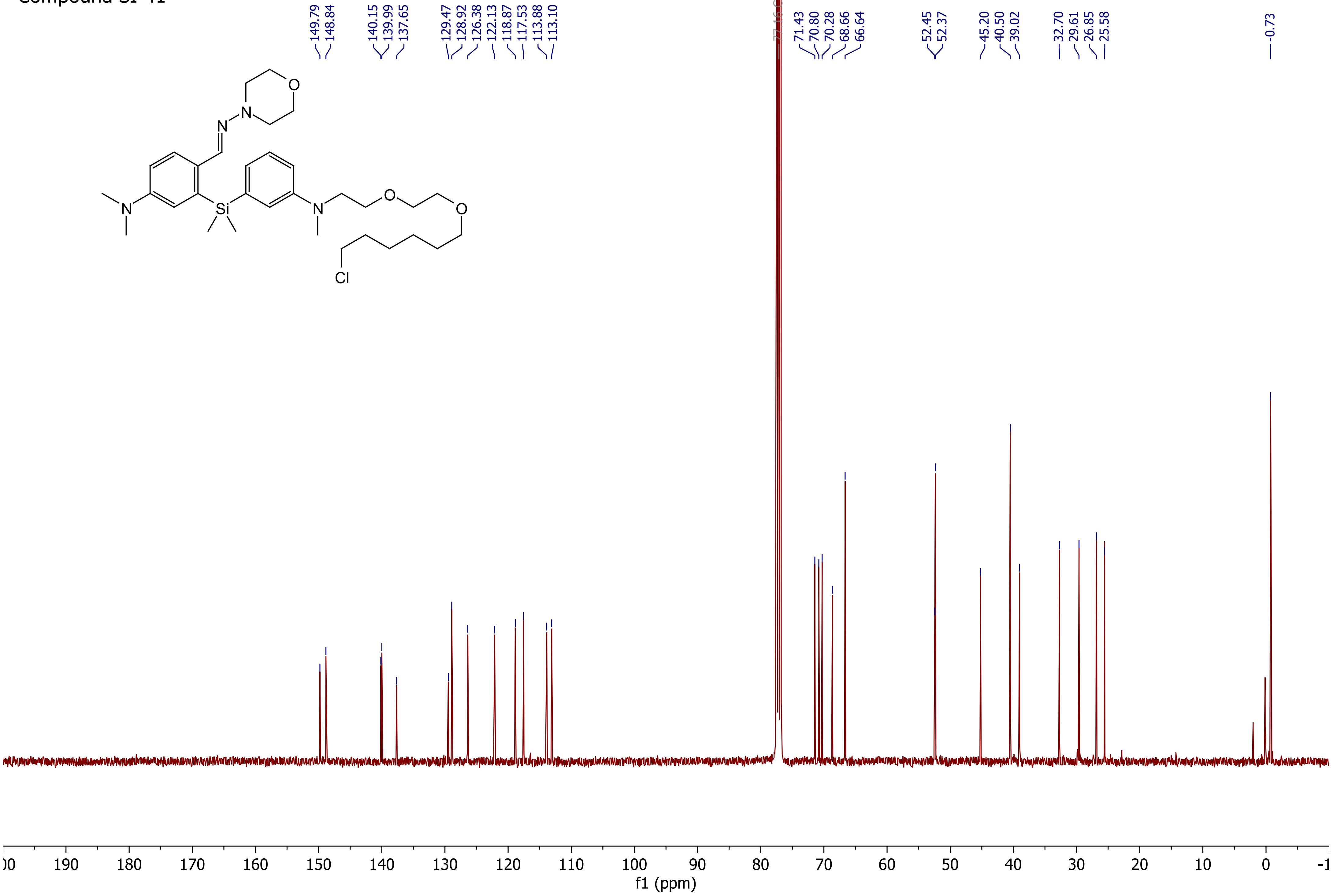

Compound SI-42

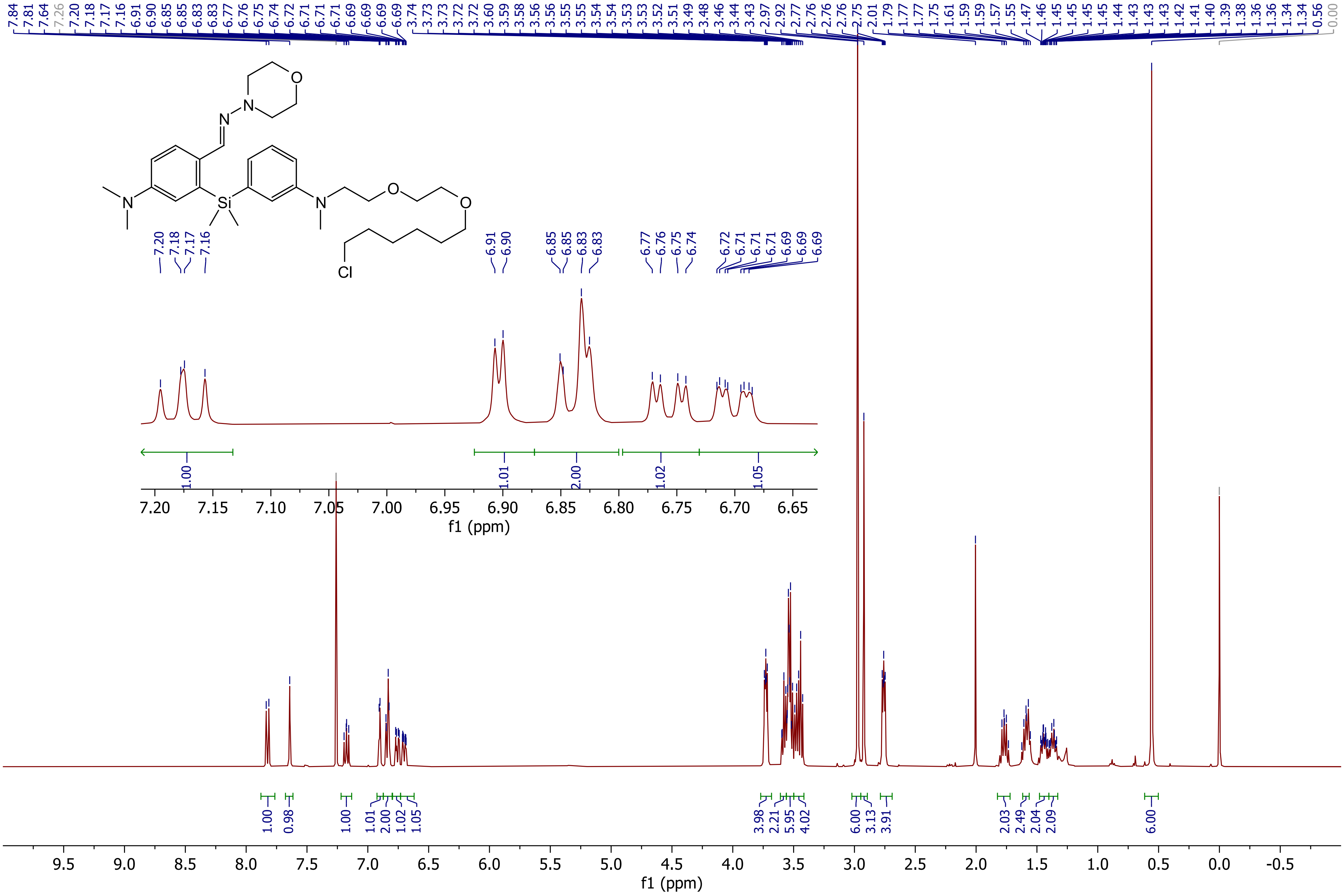

Compound SI-42

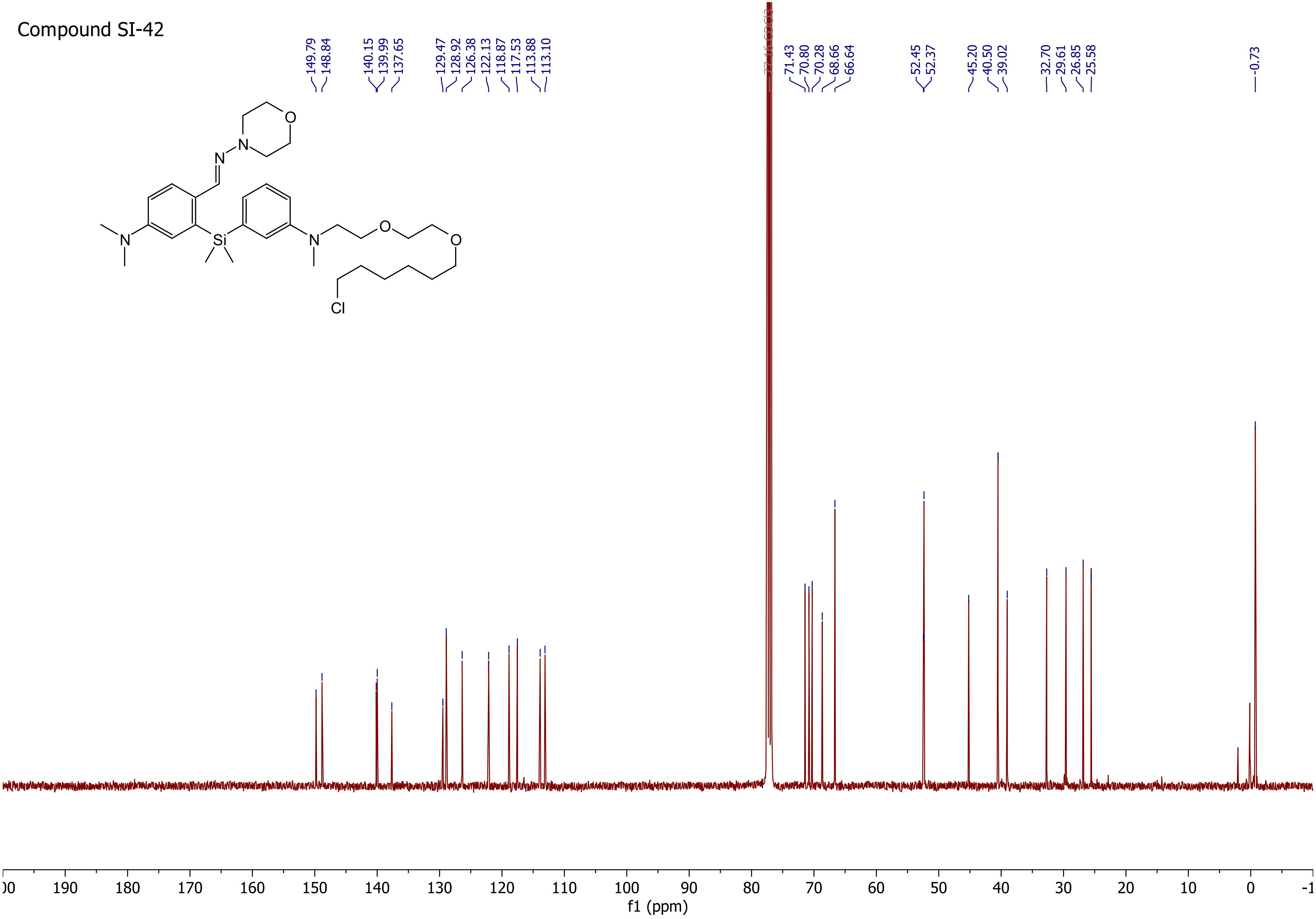

Compound SI-43

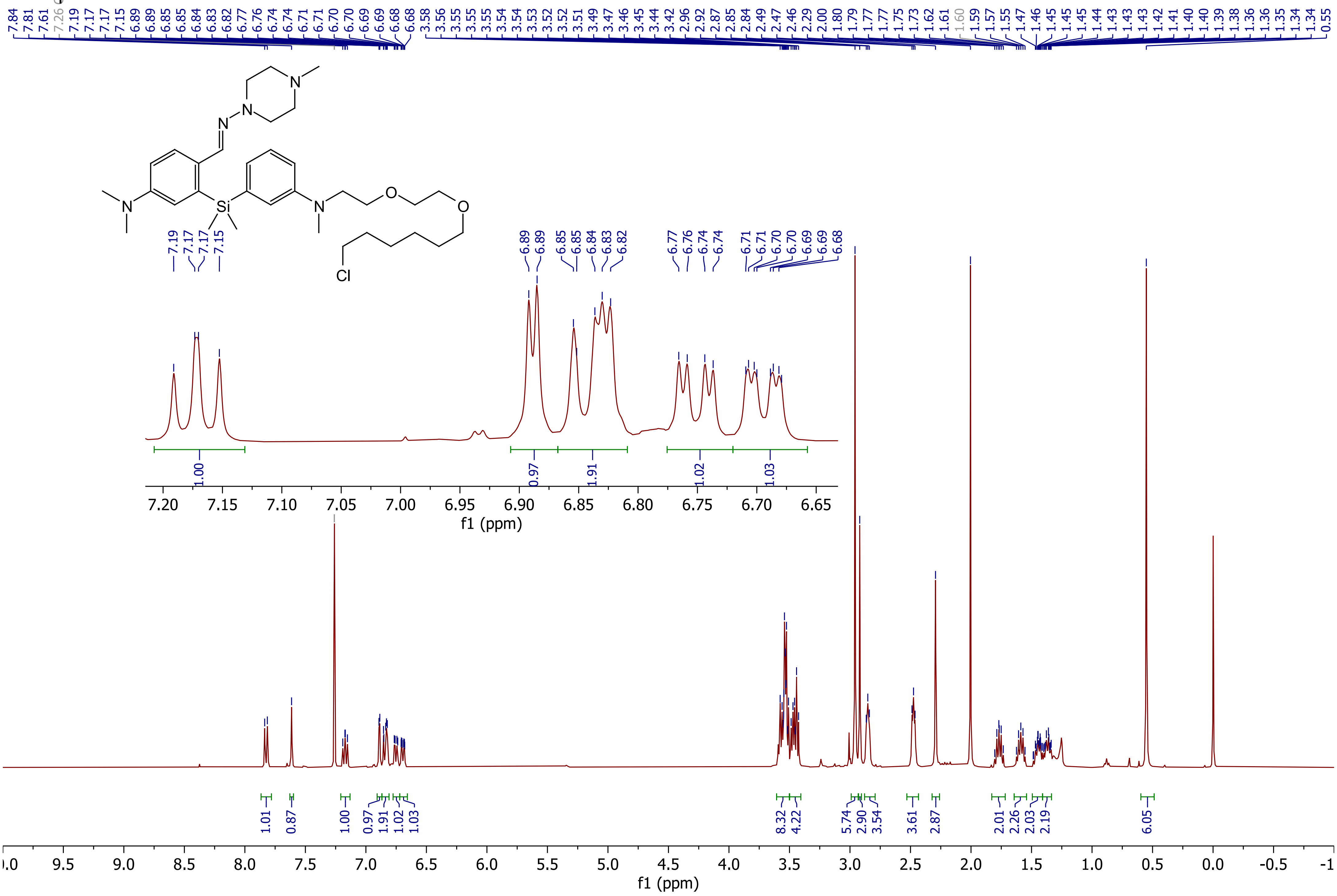

Compound SI-43

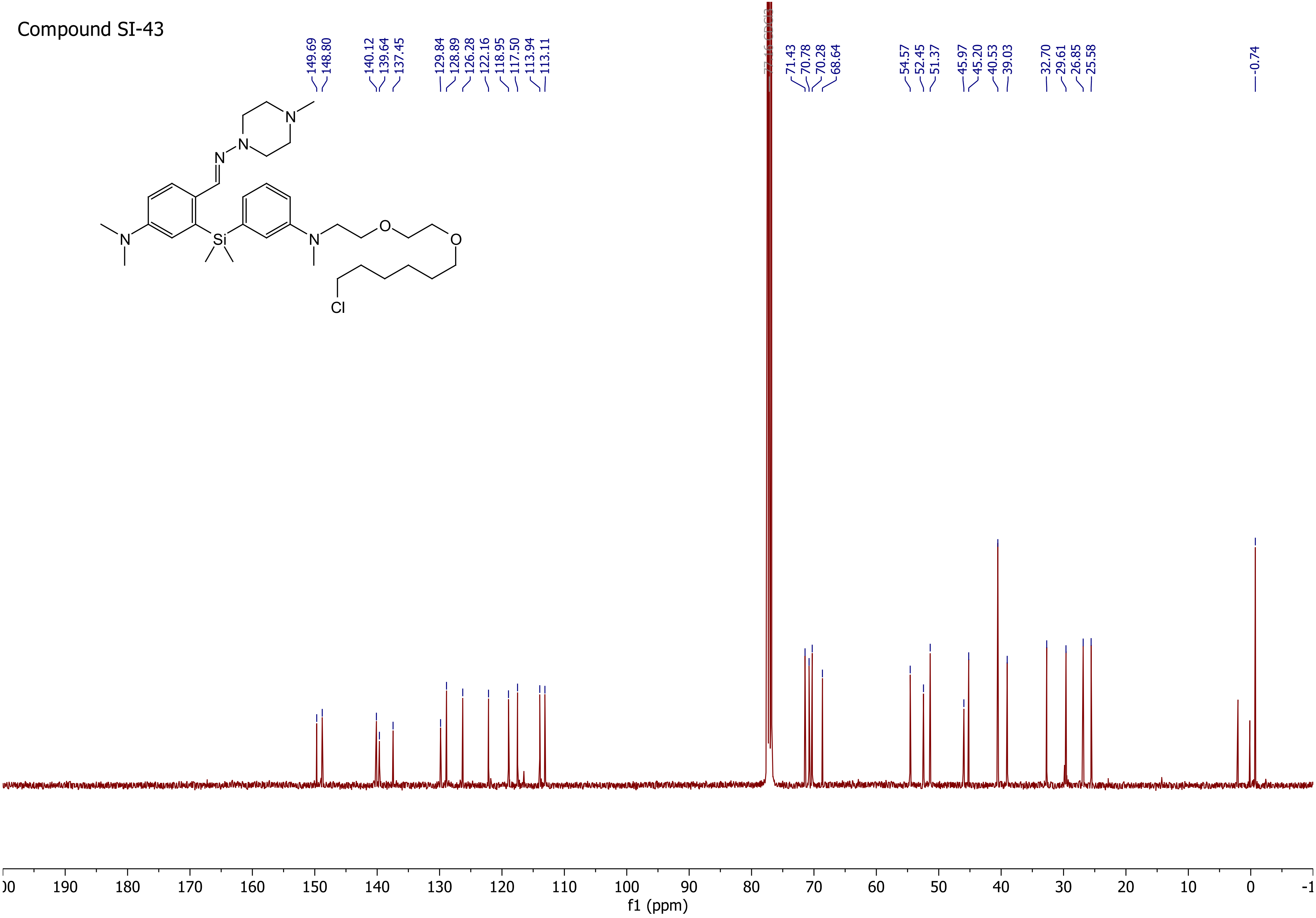

Compound SI-44

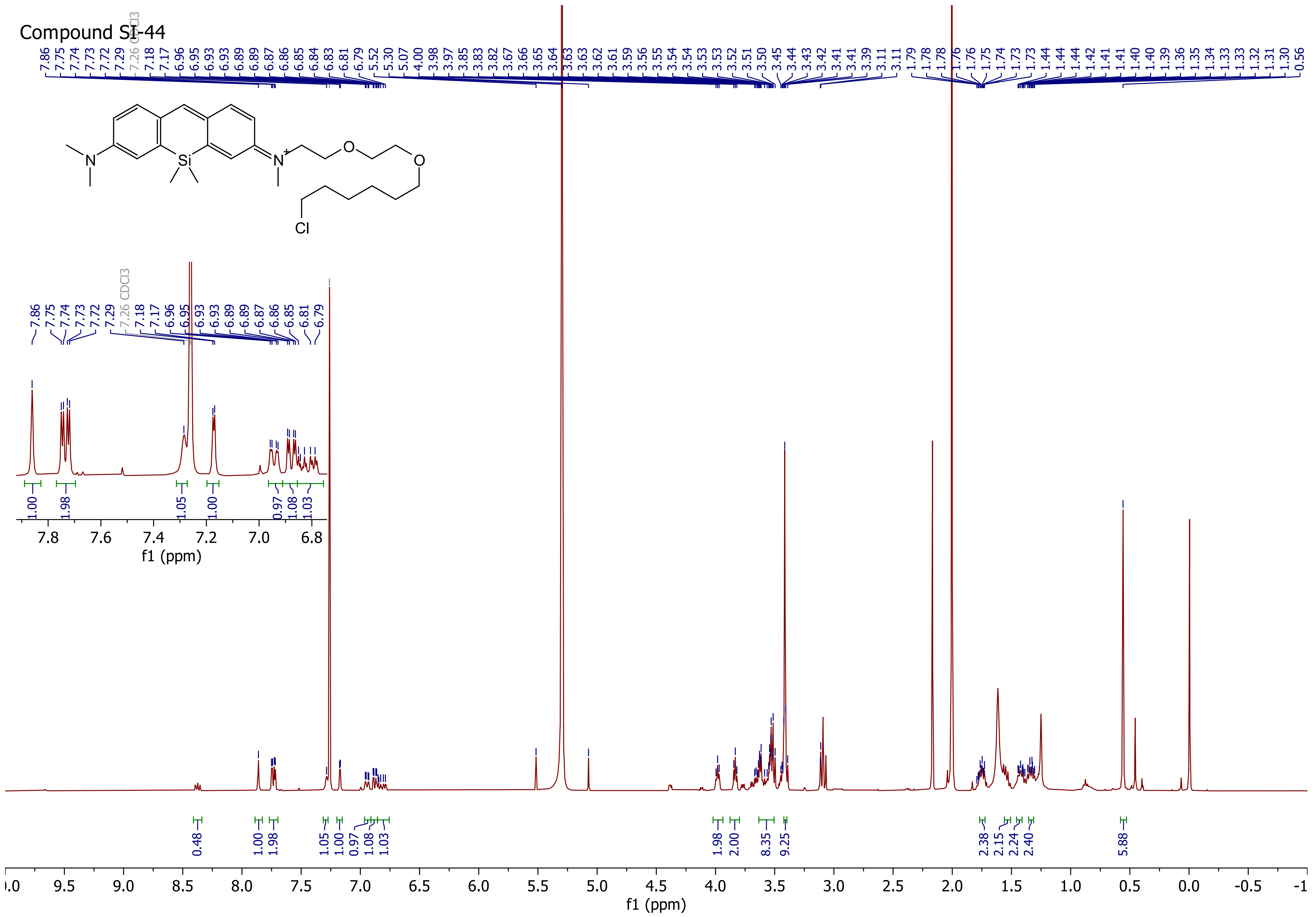

Compound SI-44

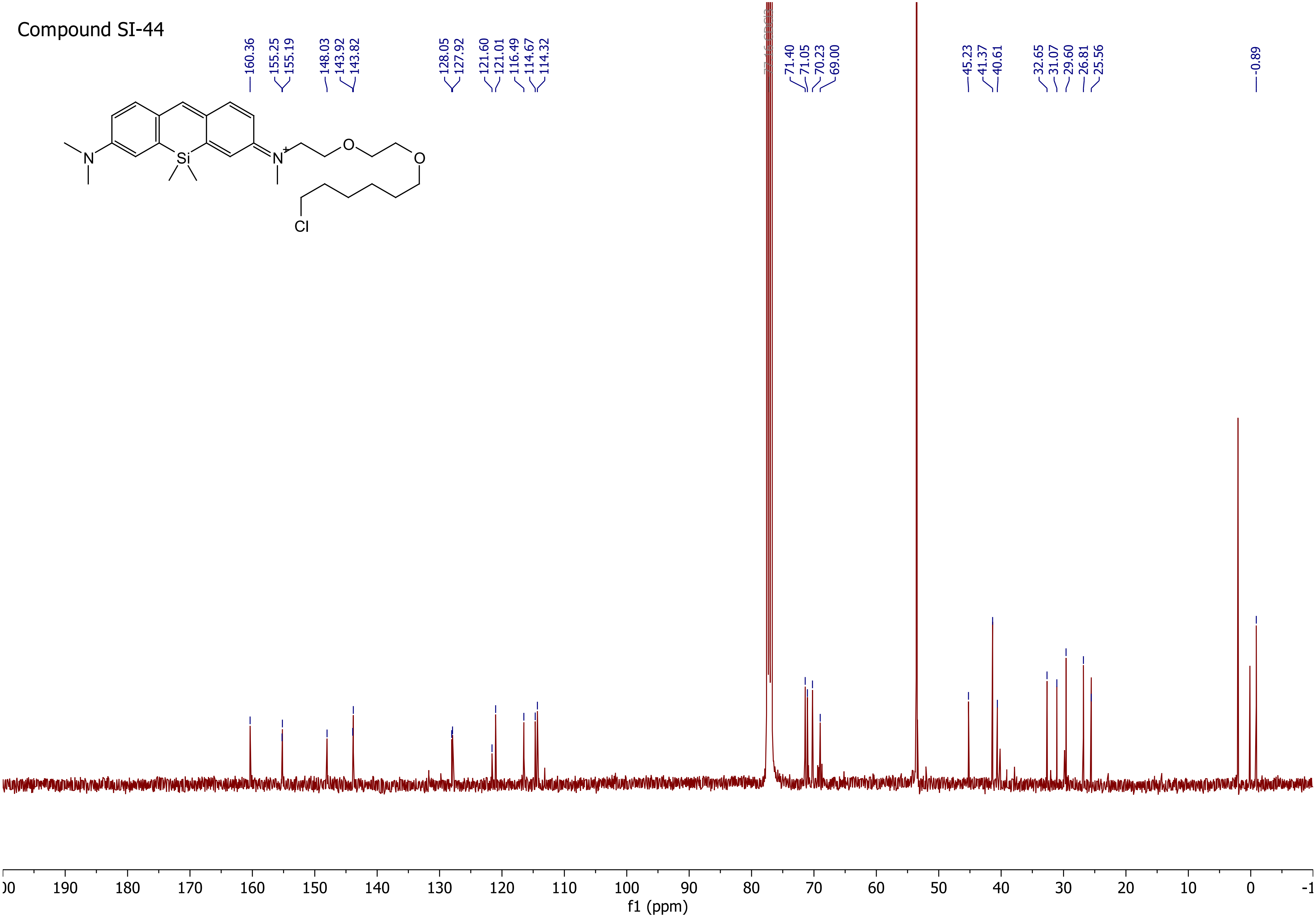

Compound PULI560

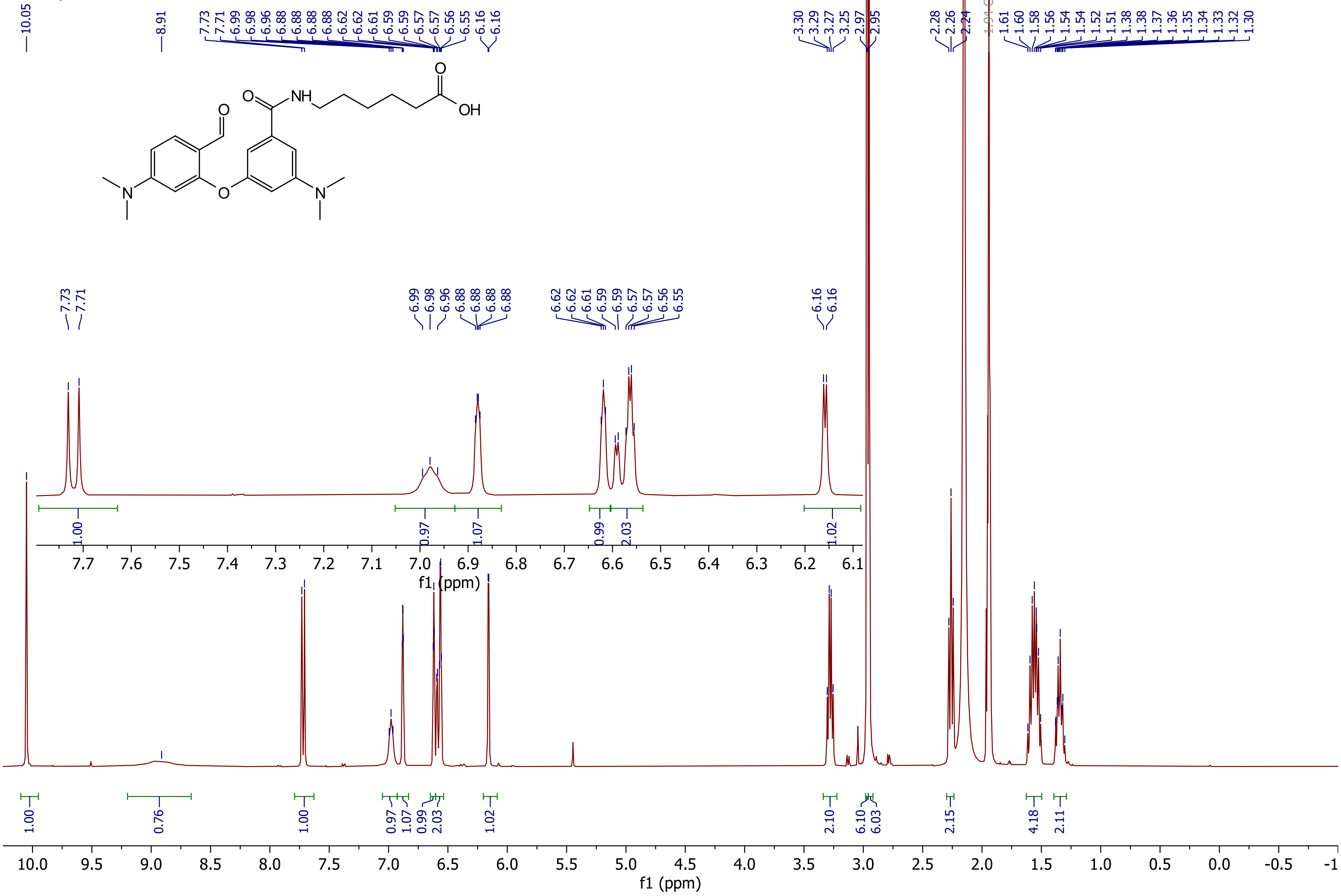

Compound PULI560

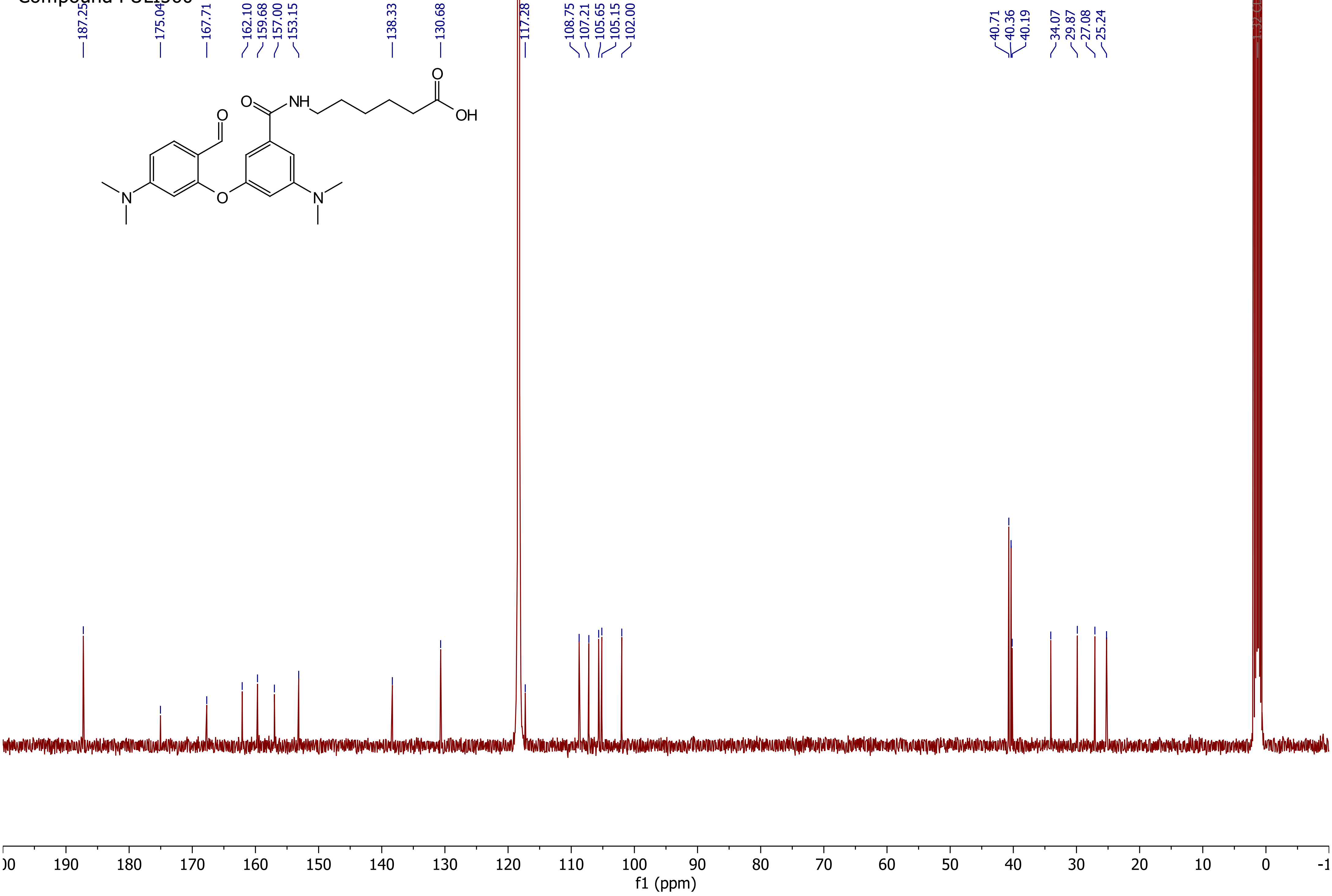

Compound PULI560-M

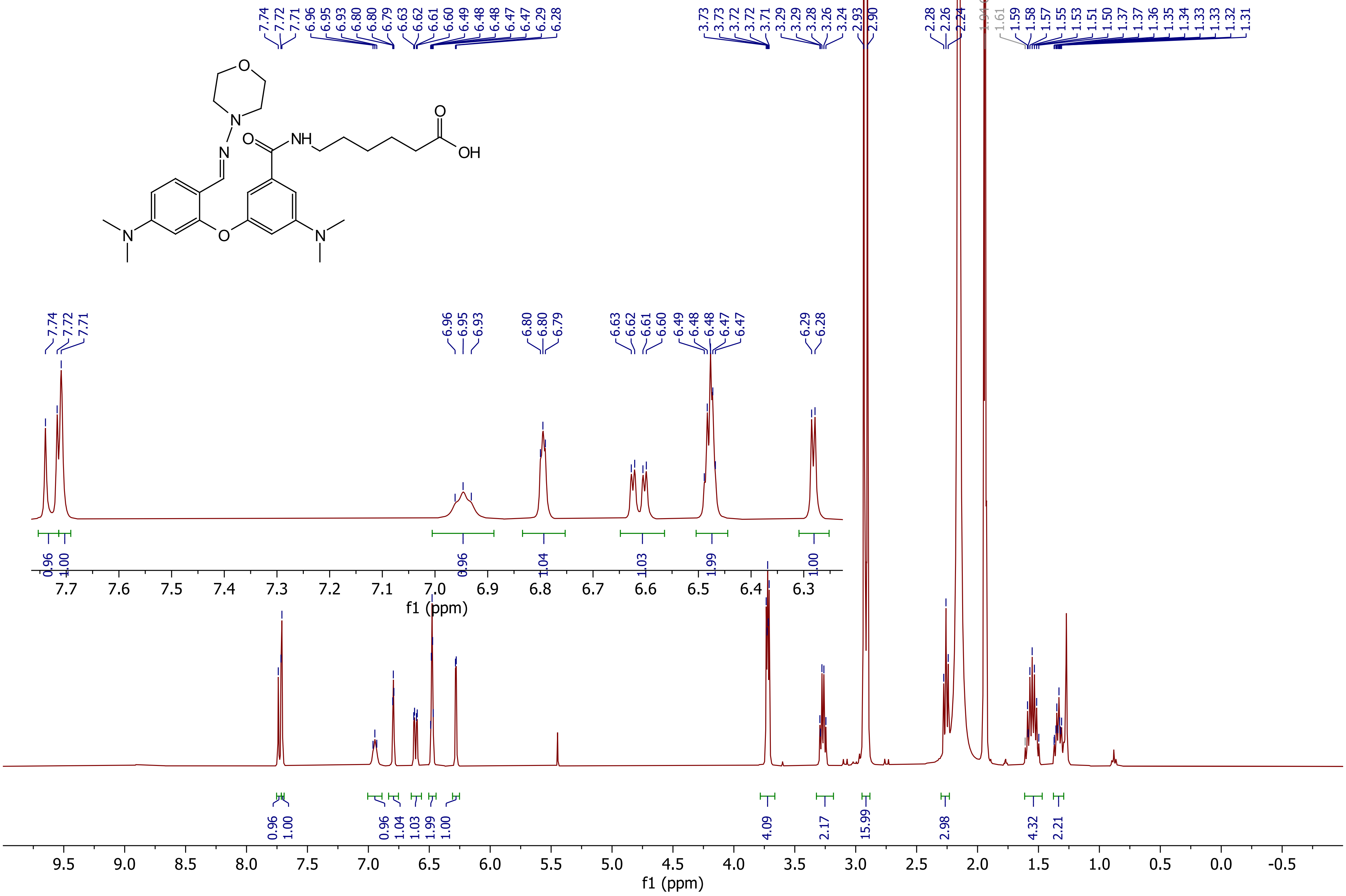

Compound PULI560-M

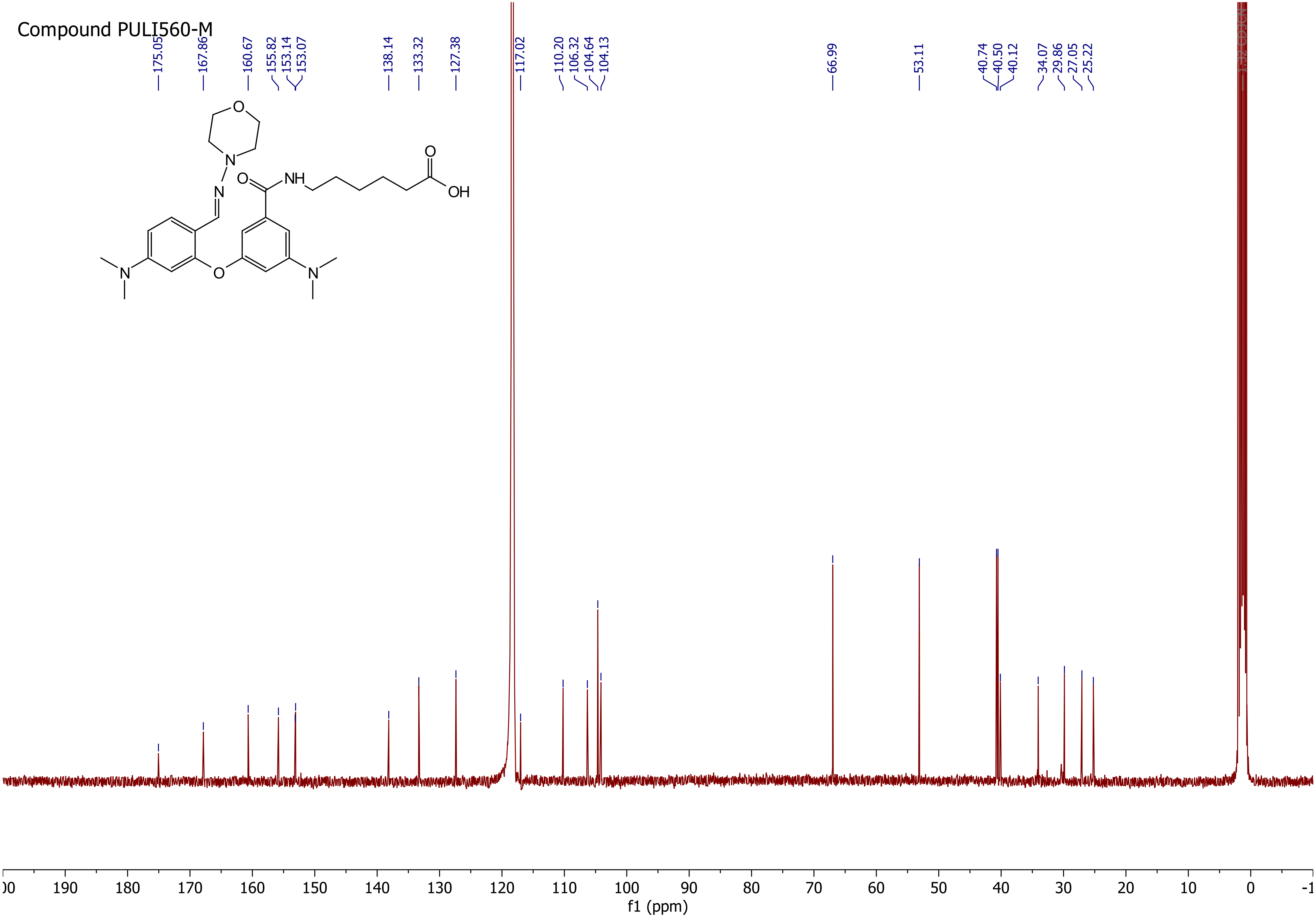

Compound PULI560-M-Halo

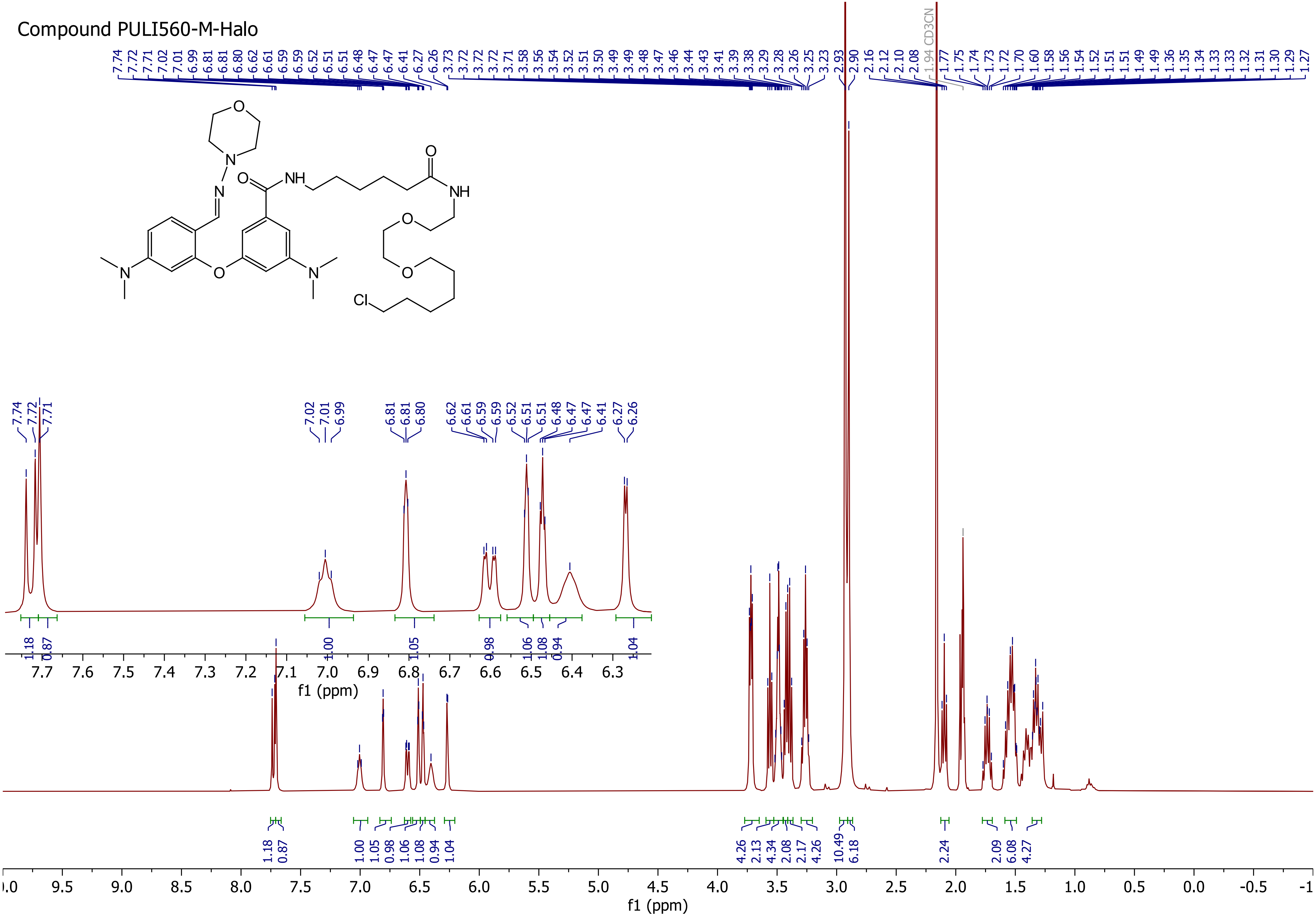

Compound PULI560-M-Halo

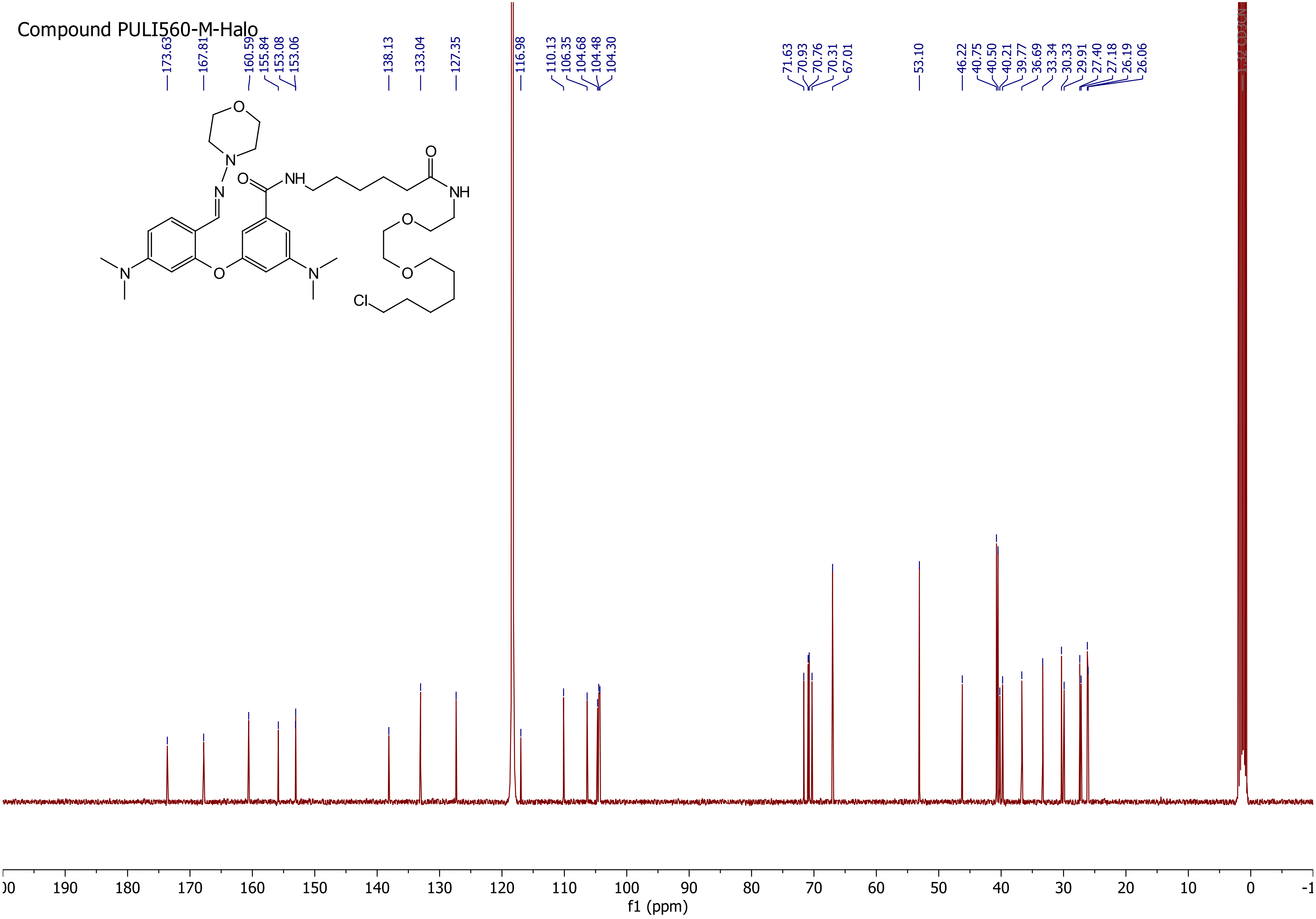

Compound PULI560-M-maleimide

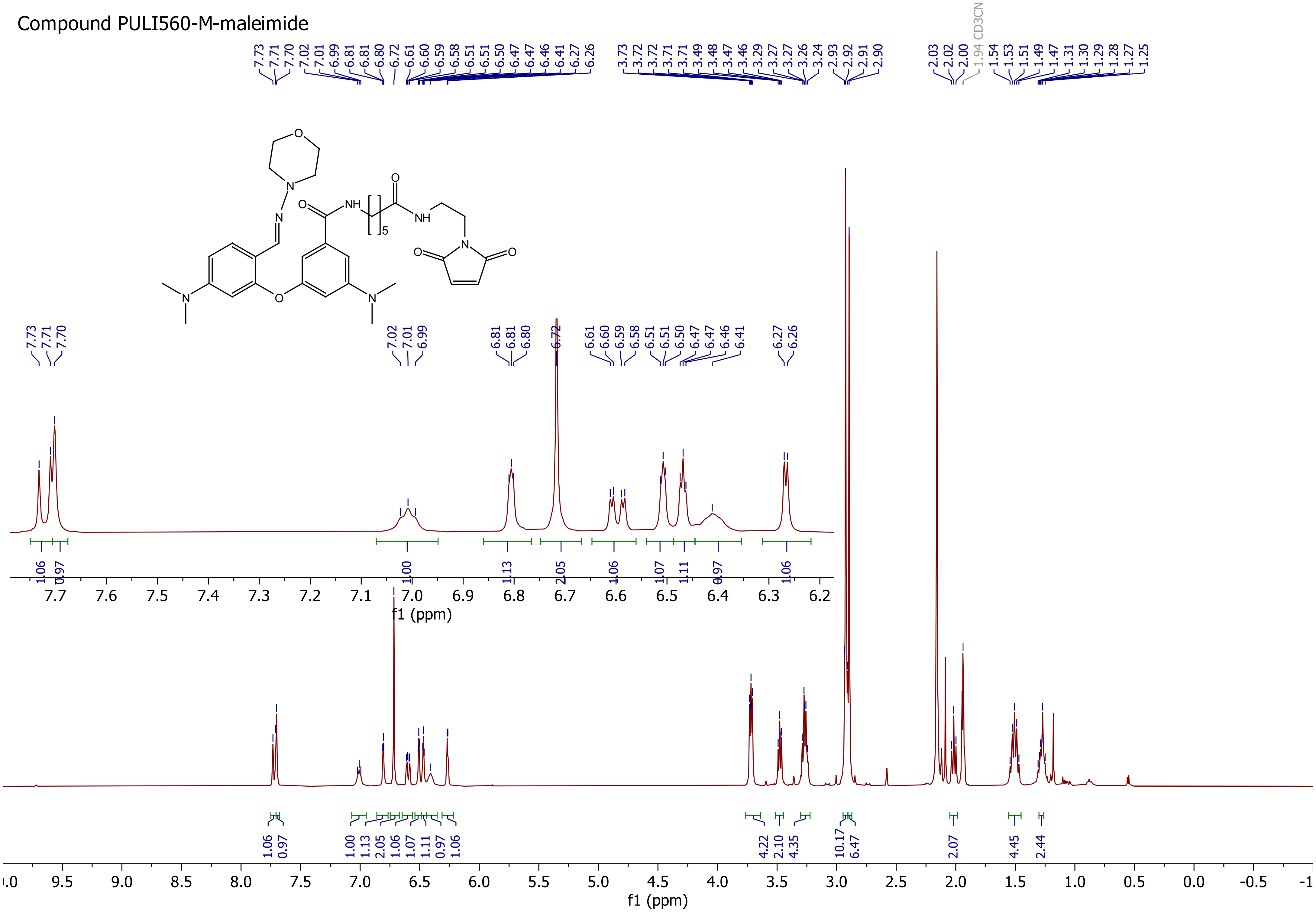

Compound PULI560-M-maleimide

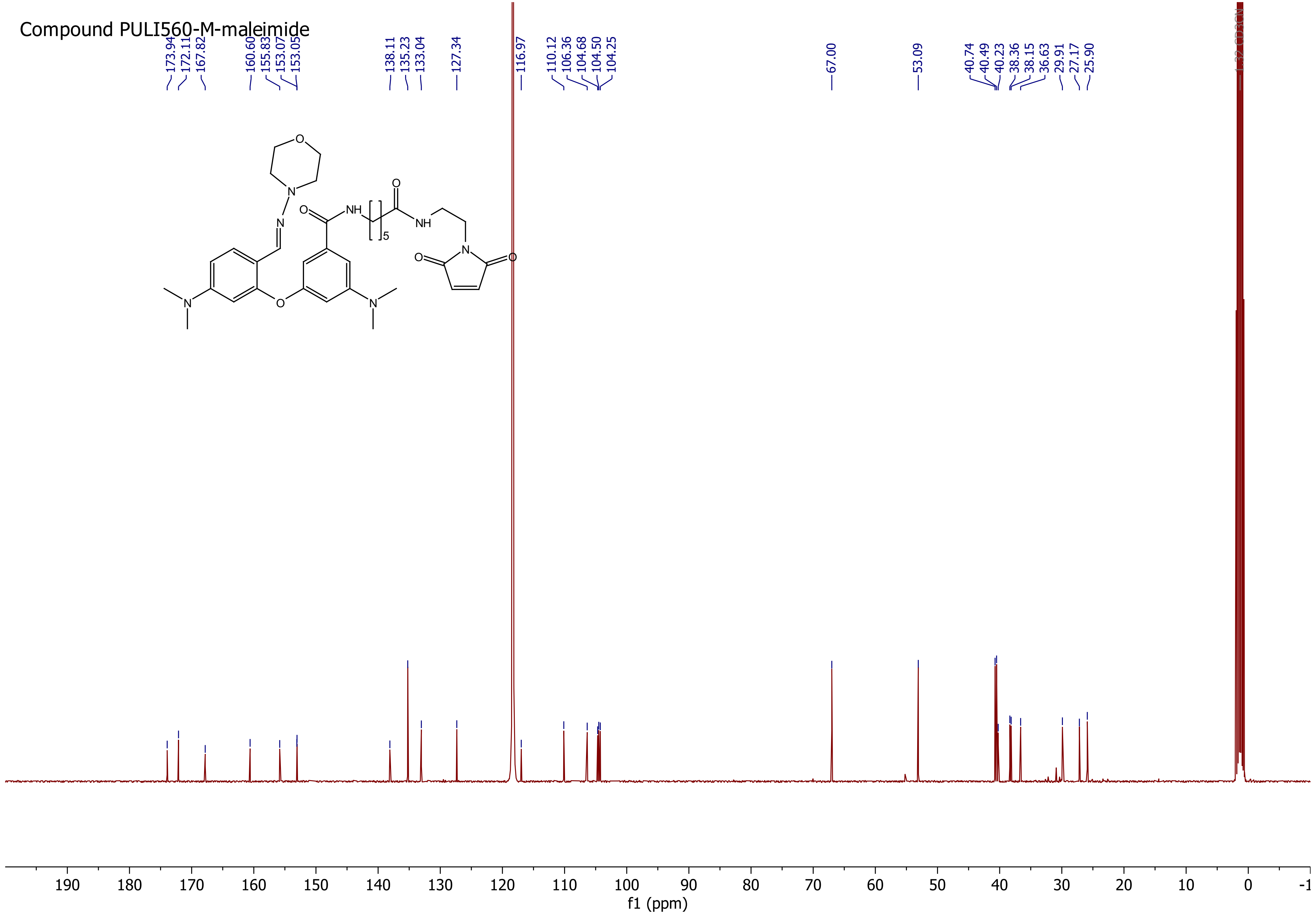

Compound PULI560-M-NHS

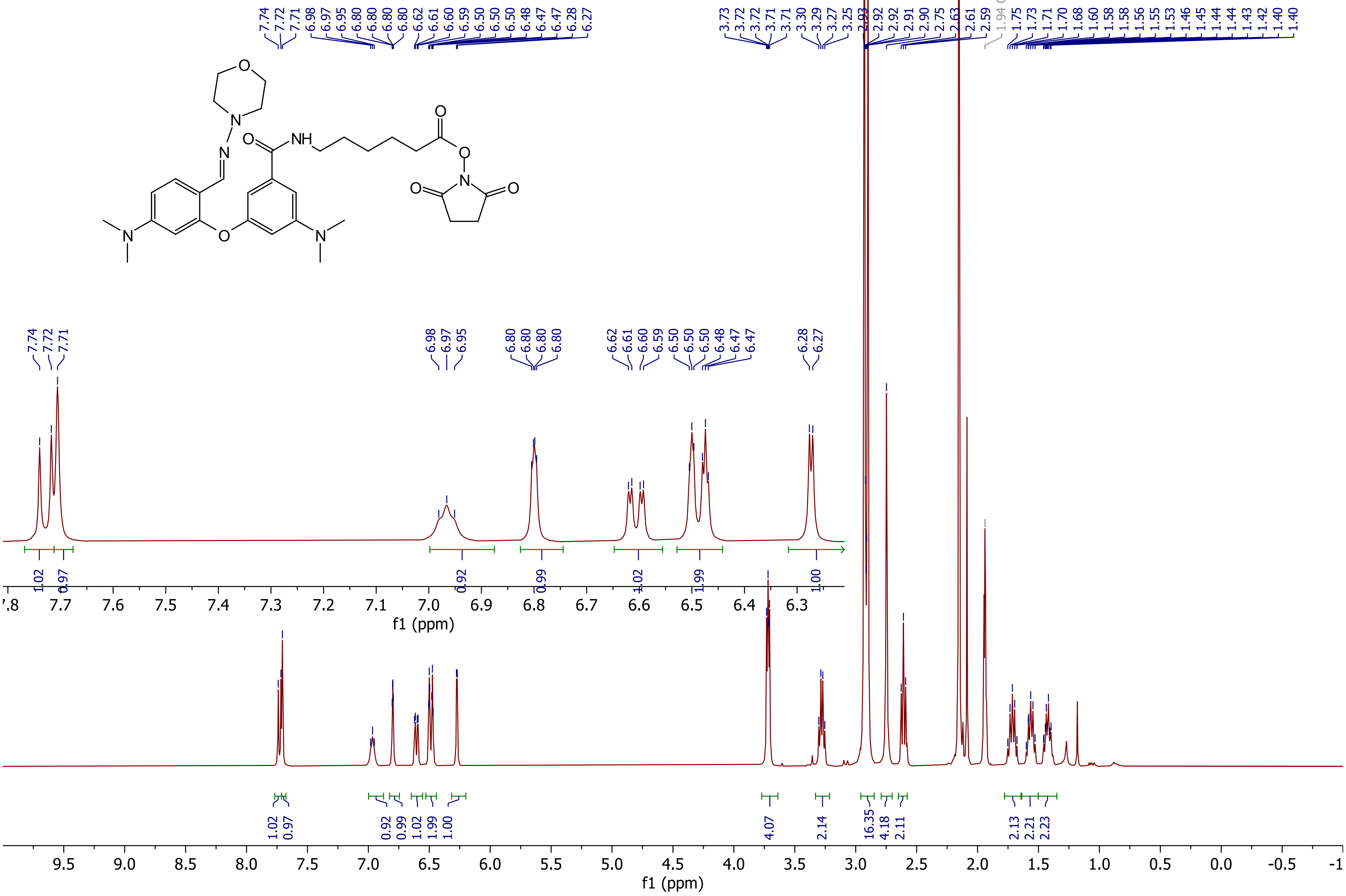

Compound PULI560-M-NHS

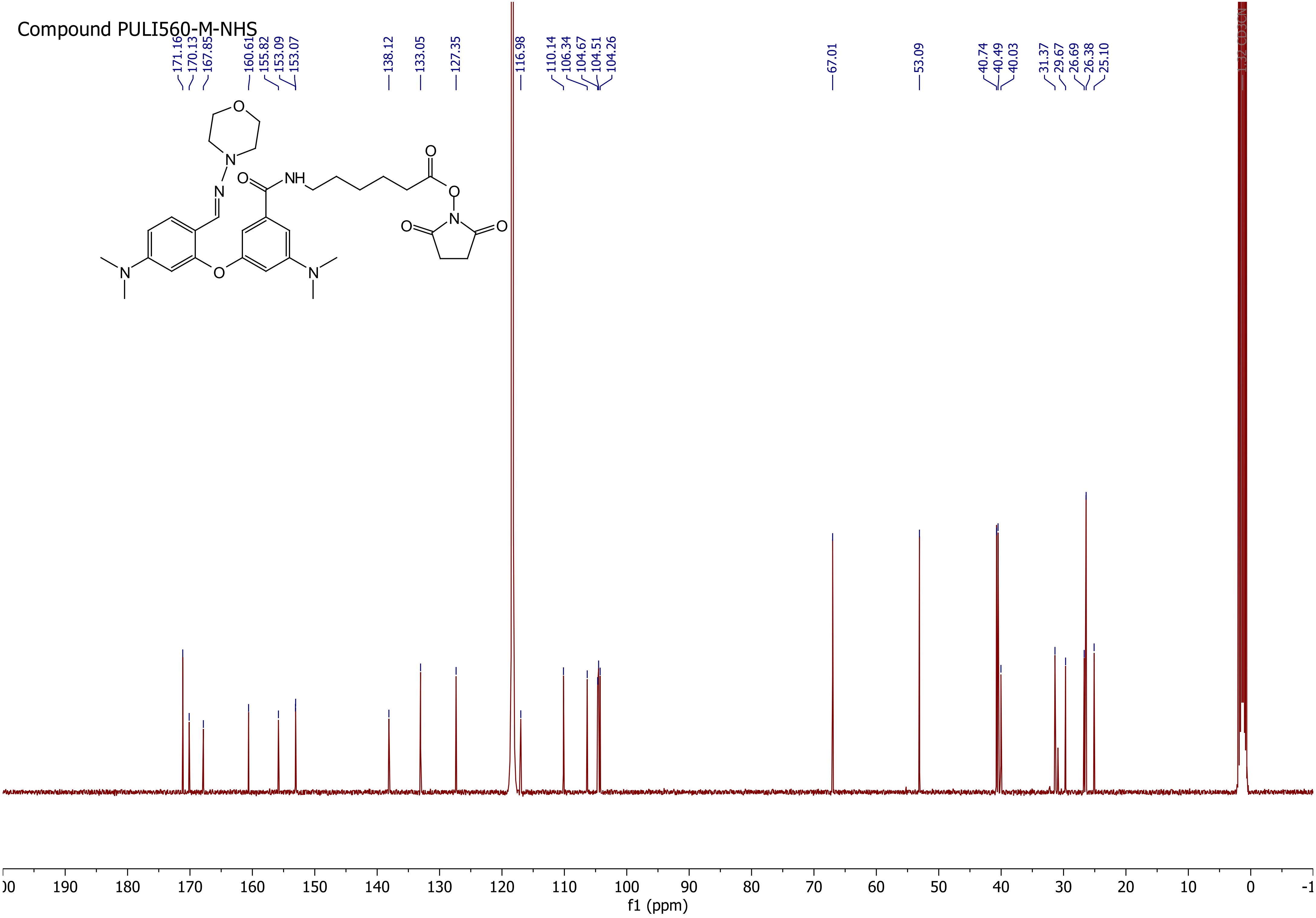

Compound PULI560-M-SNAP

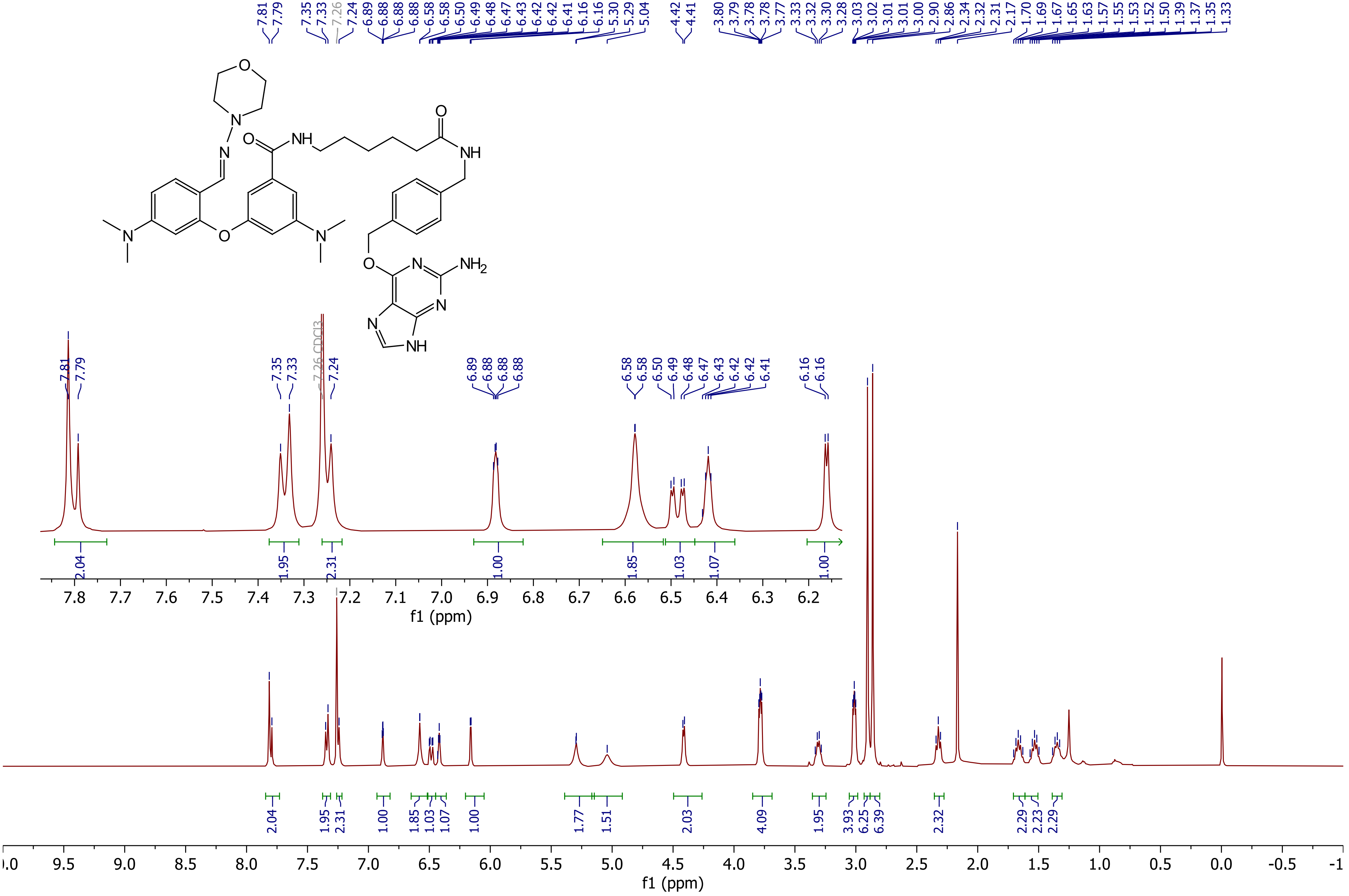

Compound PULI560-M-SNAP

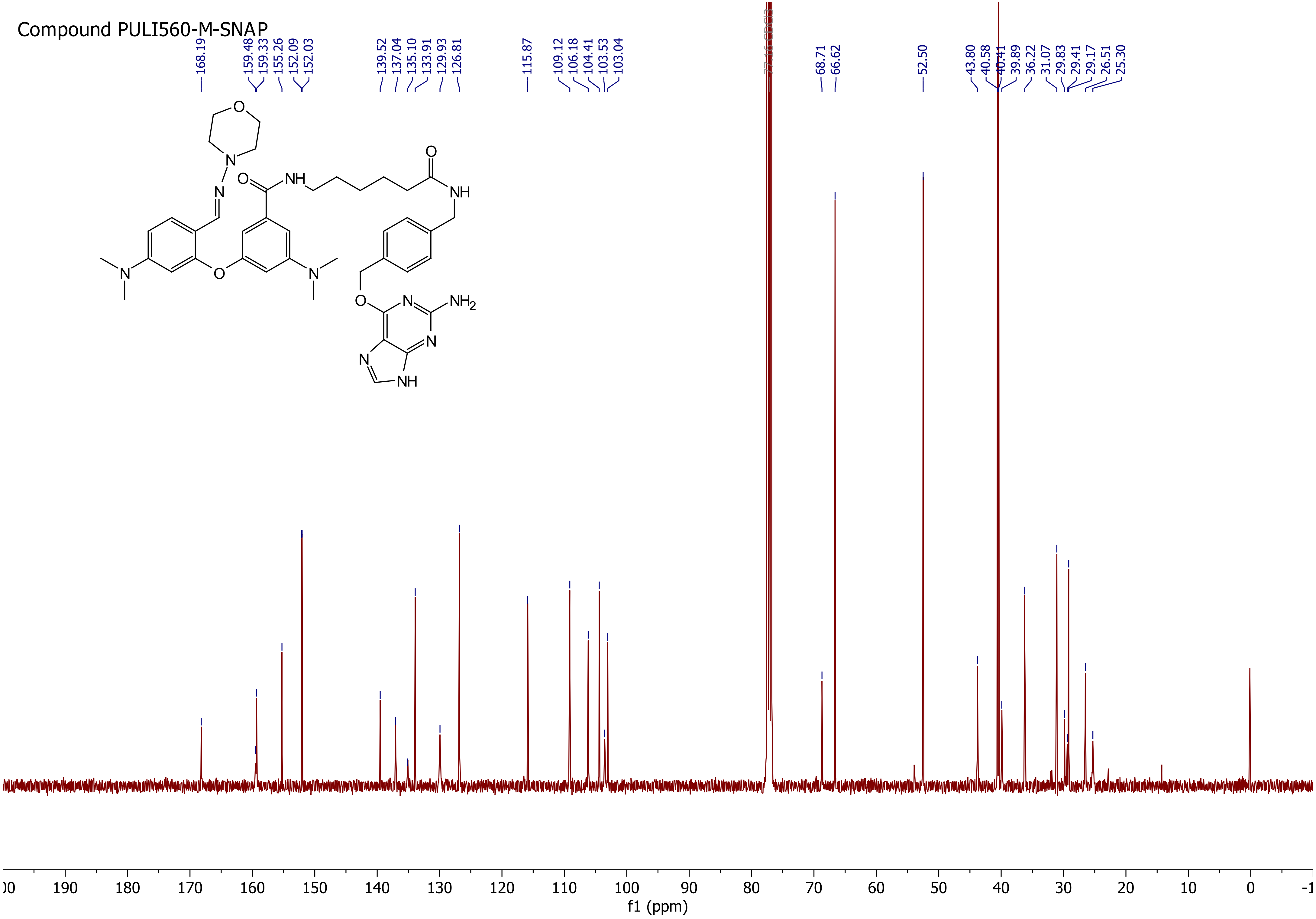

Compound PULI560-P

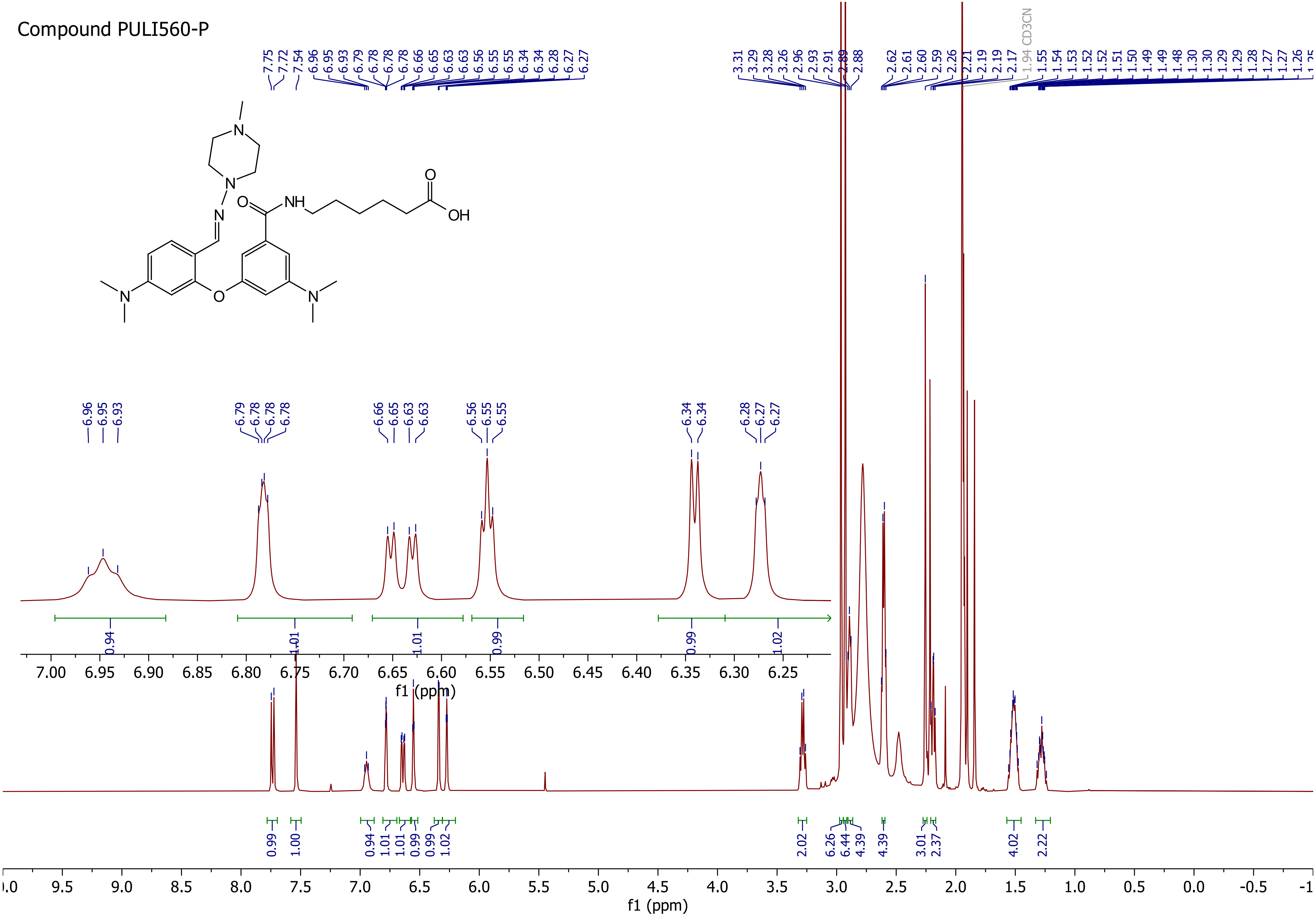

Compound PULI560-P

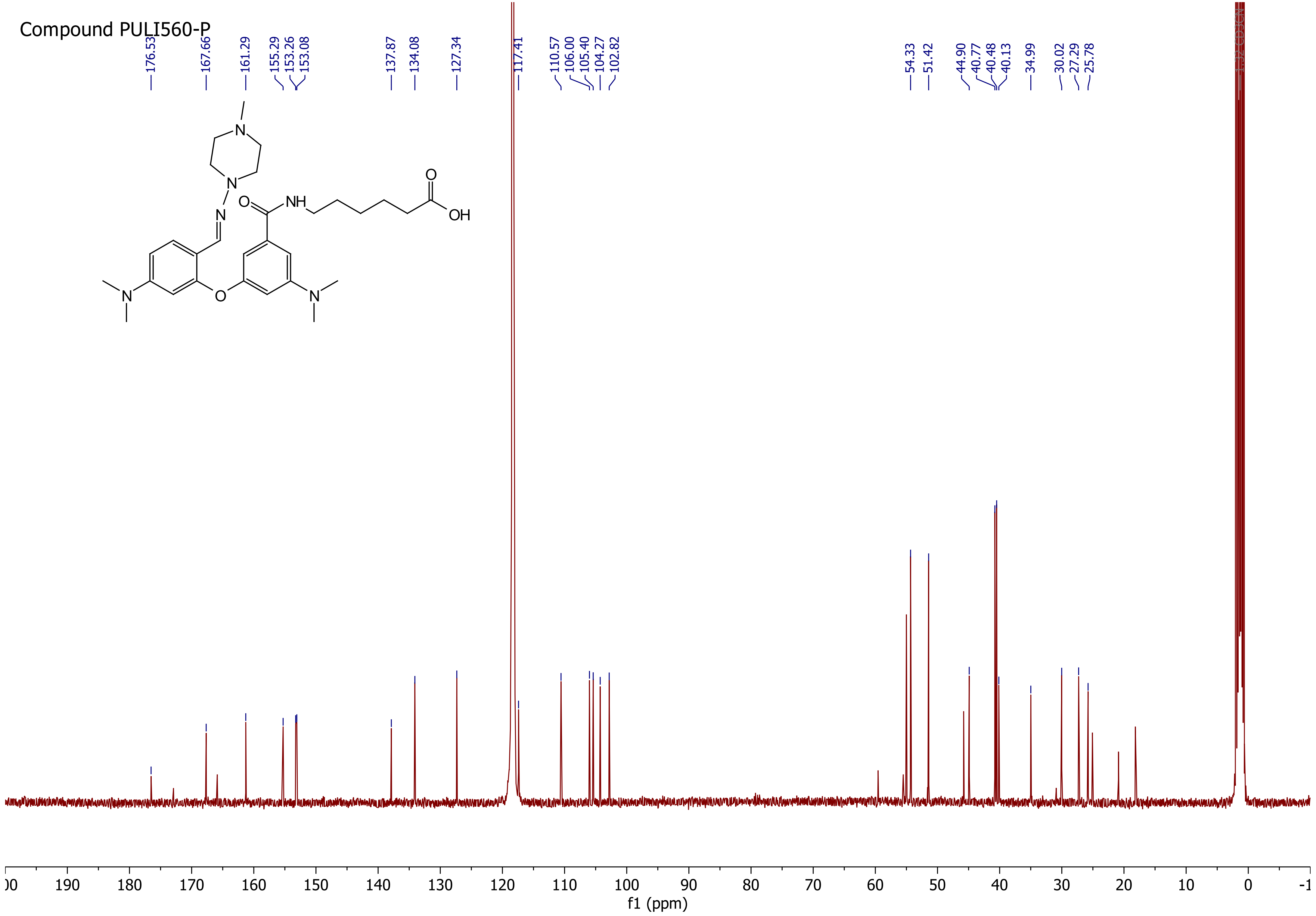

Compound PULI560-P-Halo

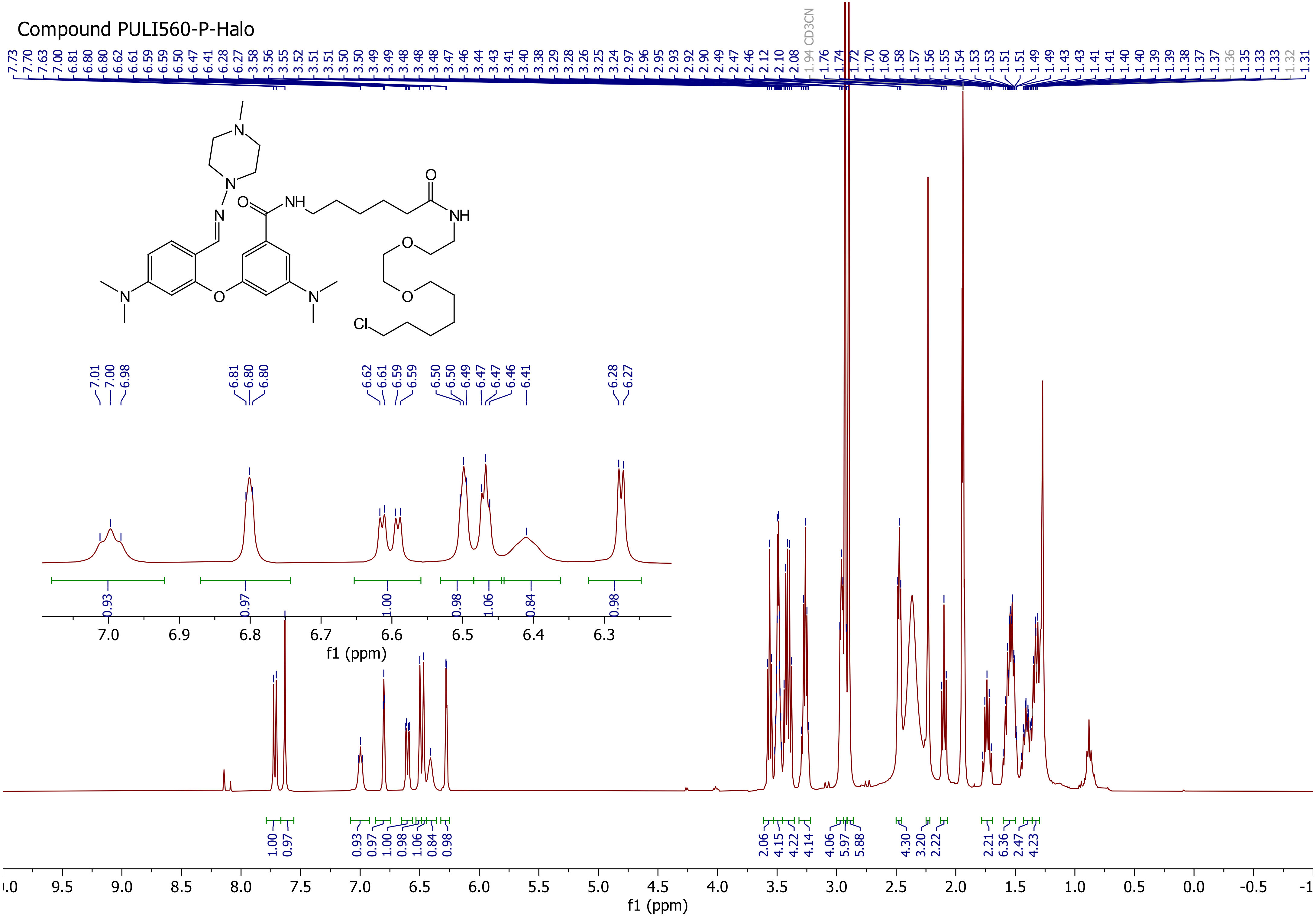

Compound PULI560-P-Halo

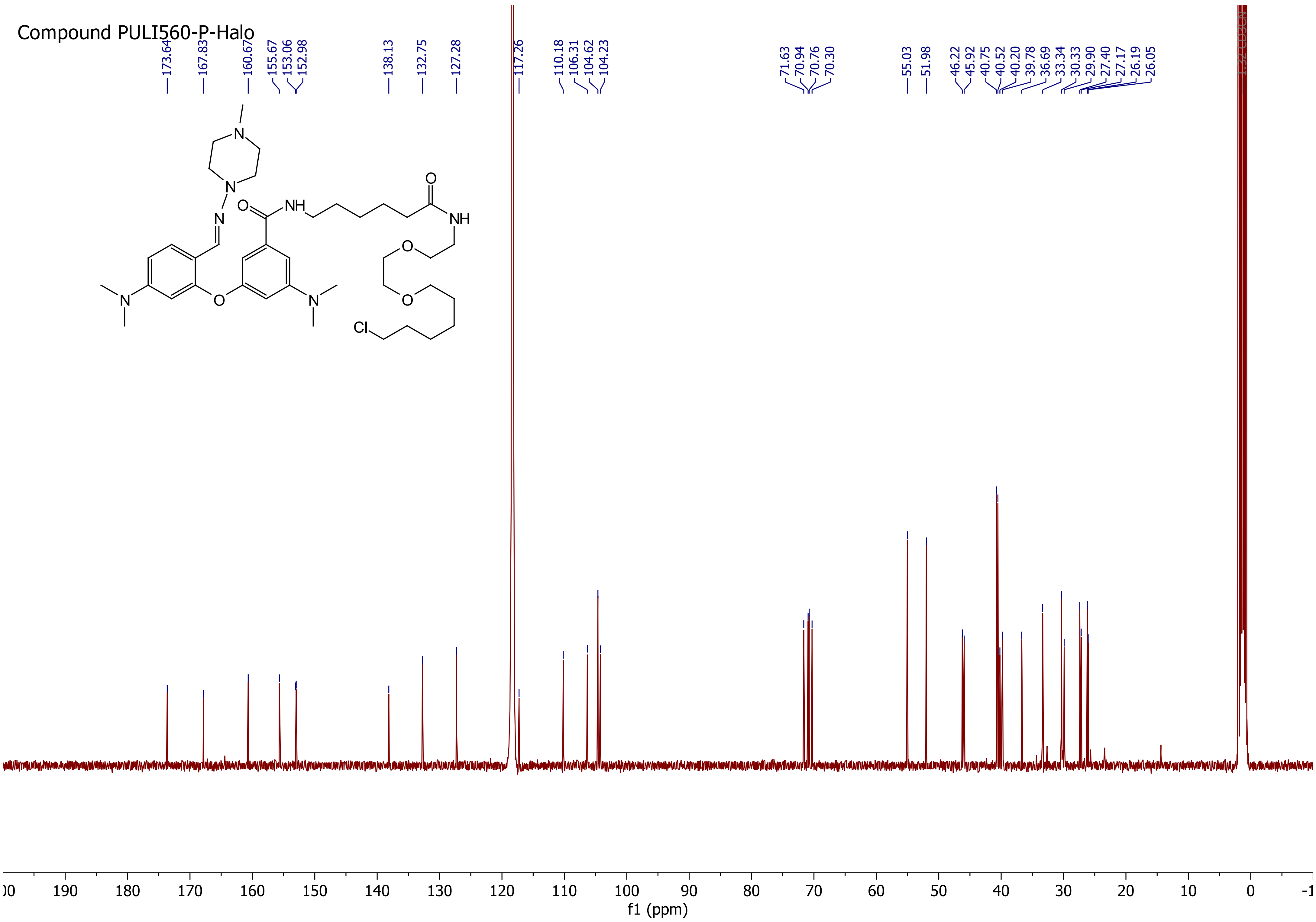

Compound PULI560-P-maleimide

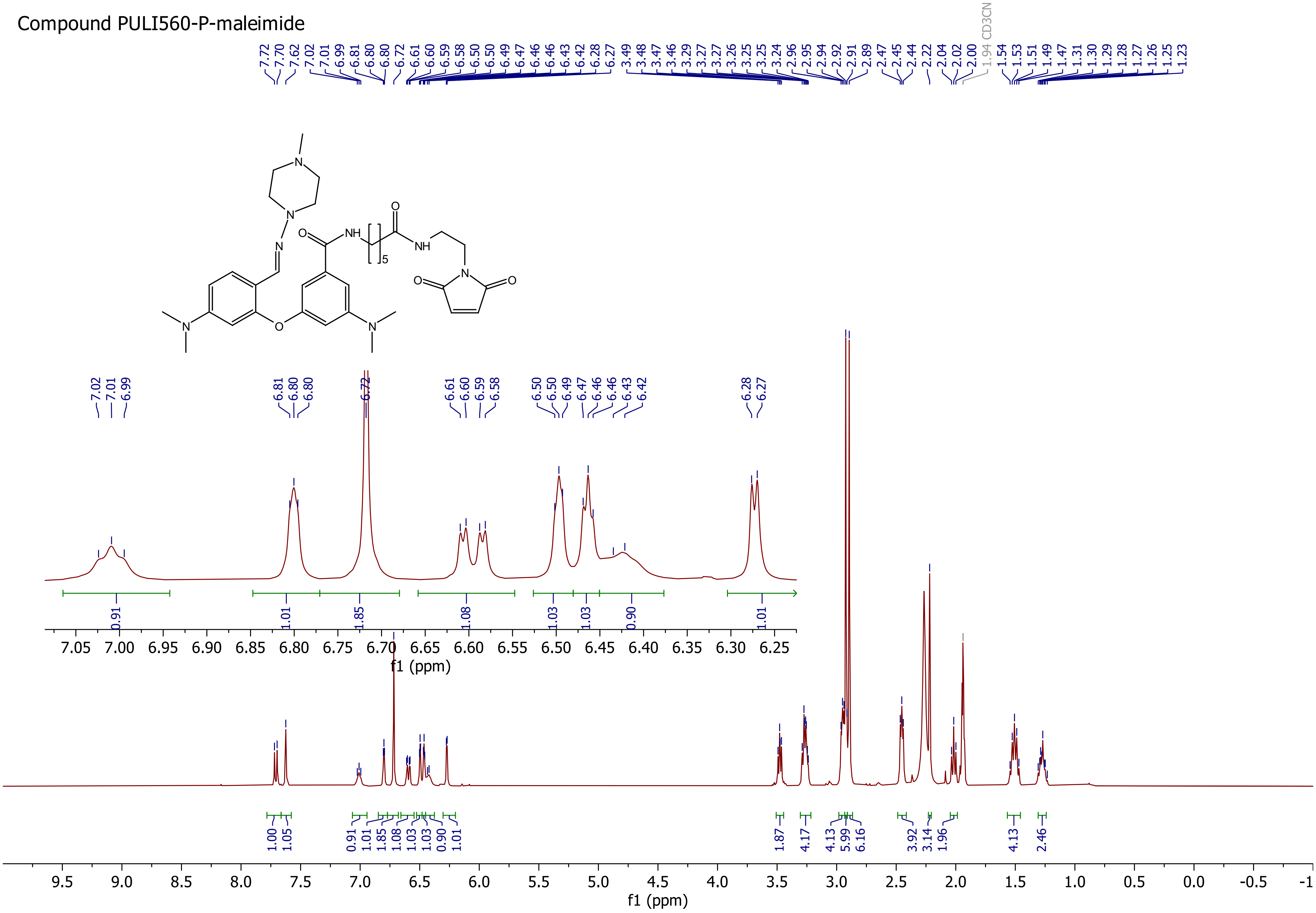

Compound PULI560-P-maleimide

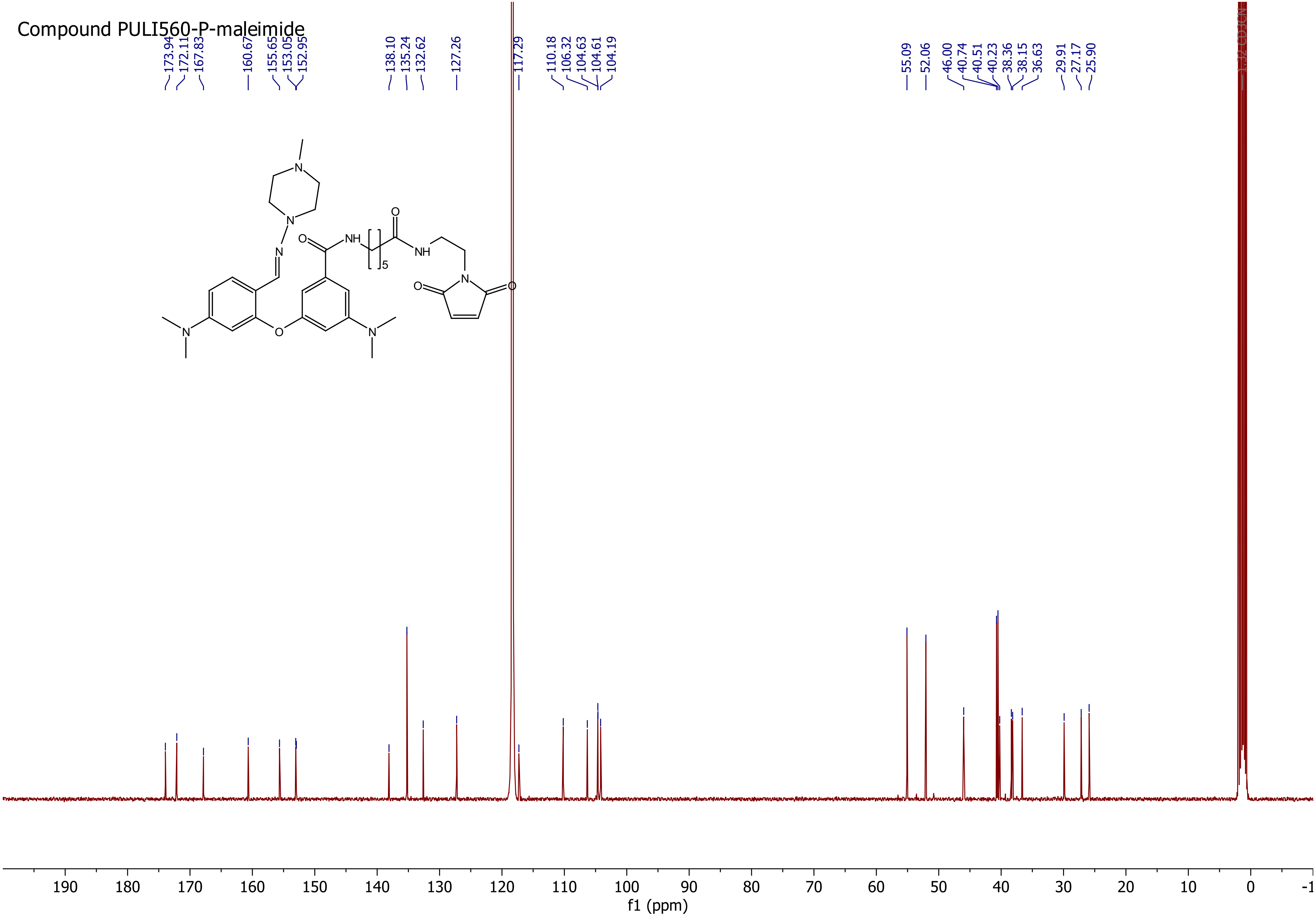

Compound PULI560-P-NHS

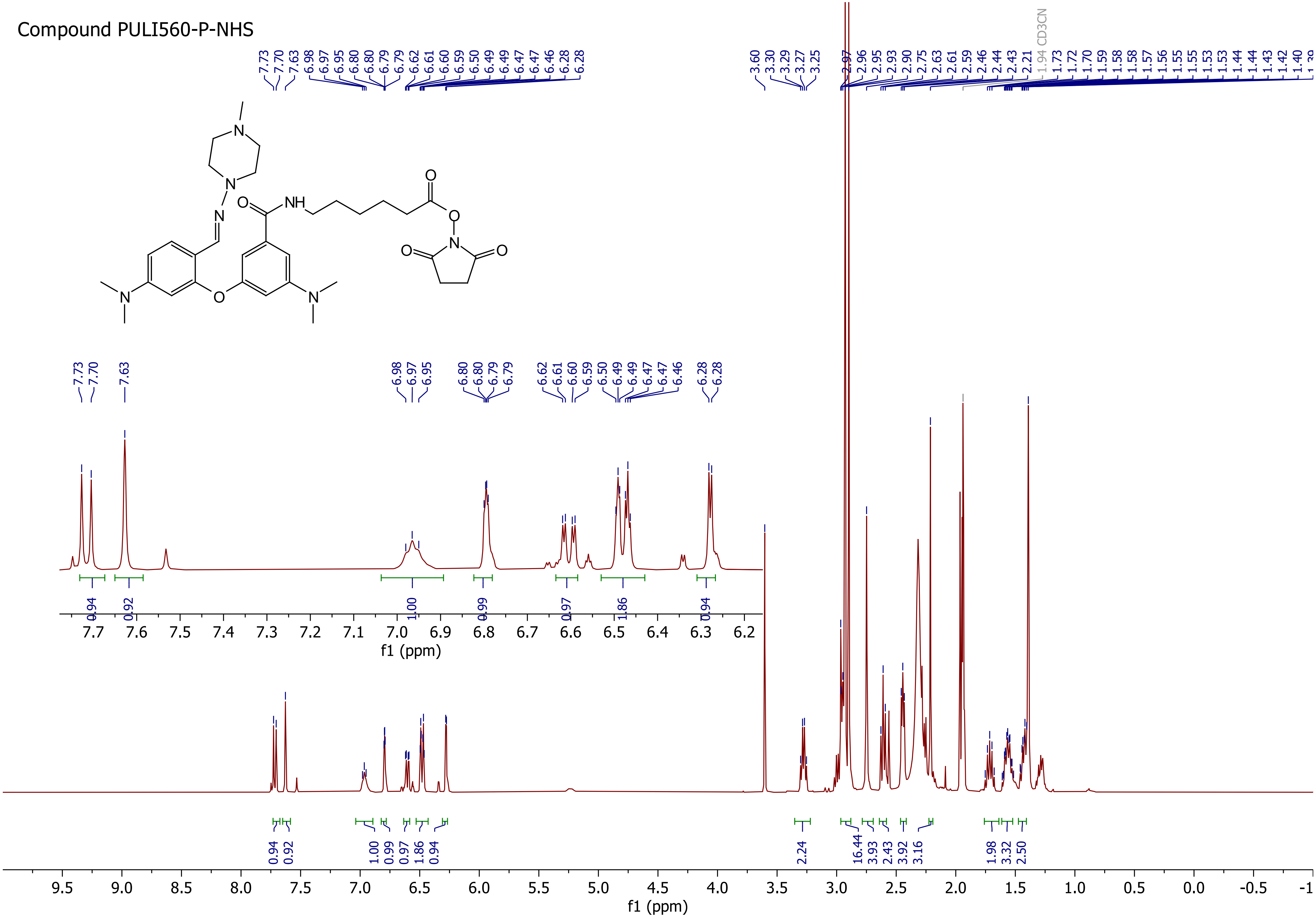

Compound PULI560-P-NHS

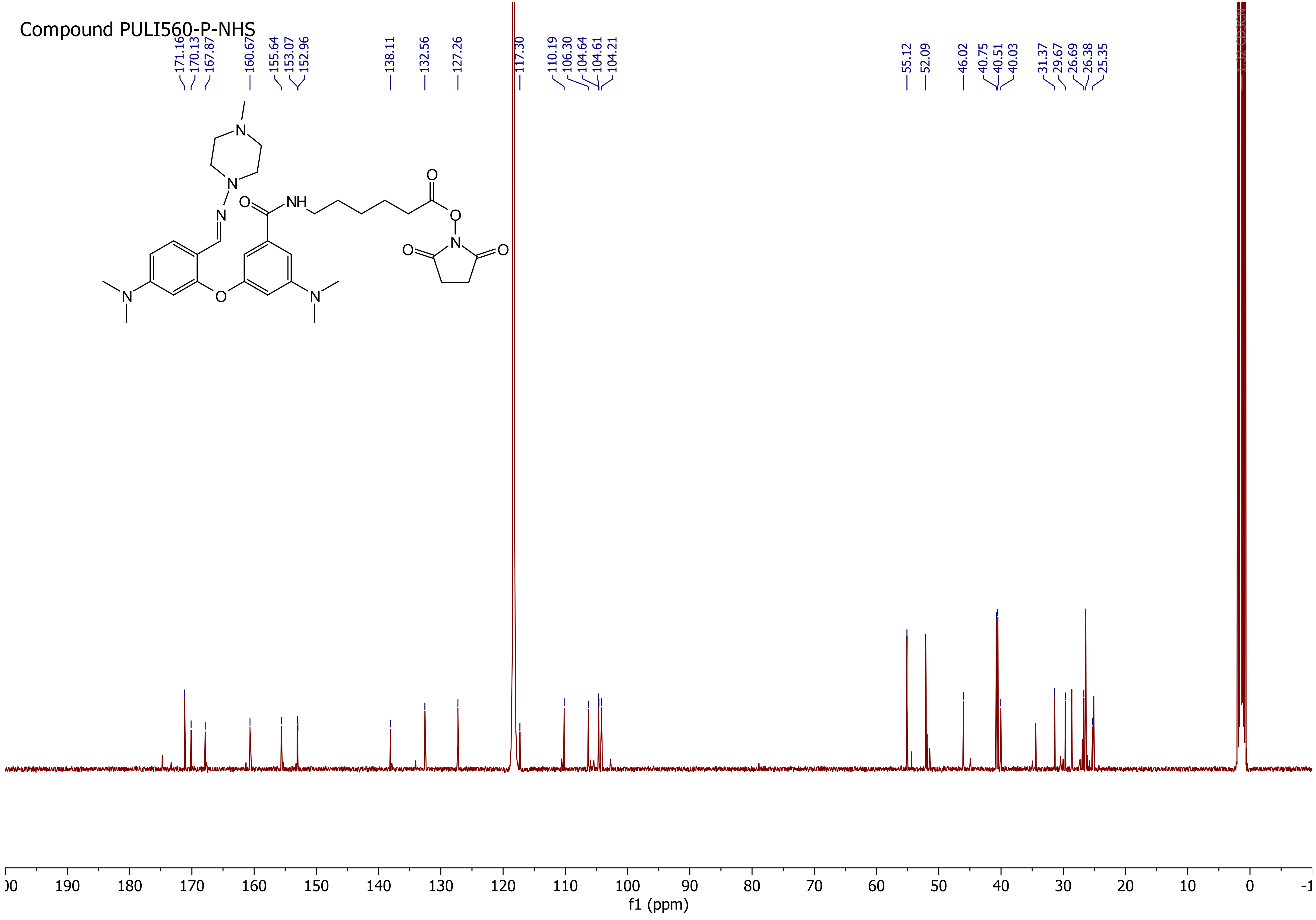

Compound PULI560-P-SNAP

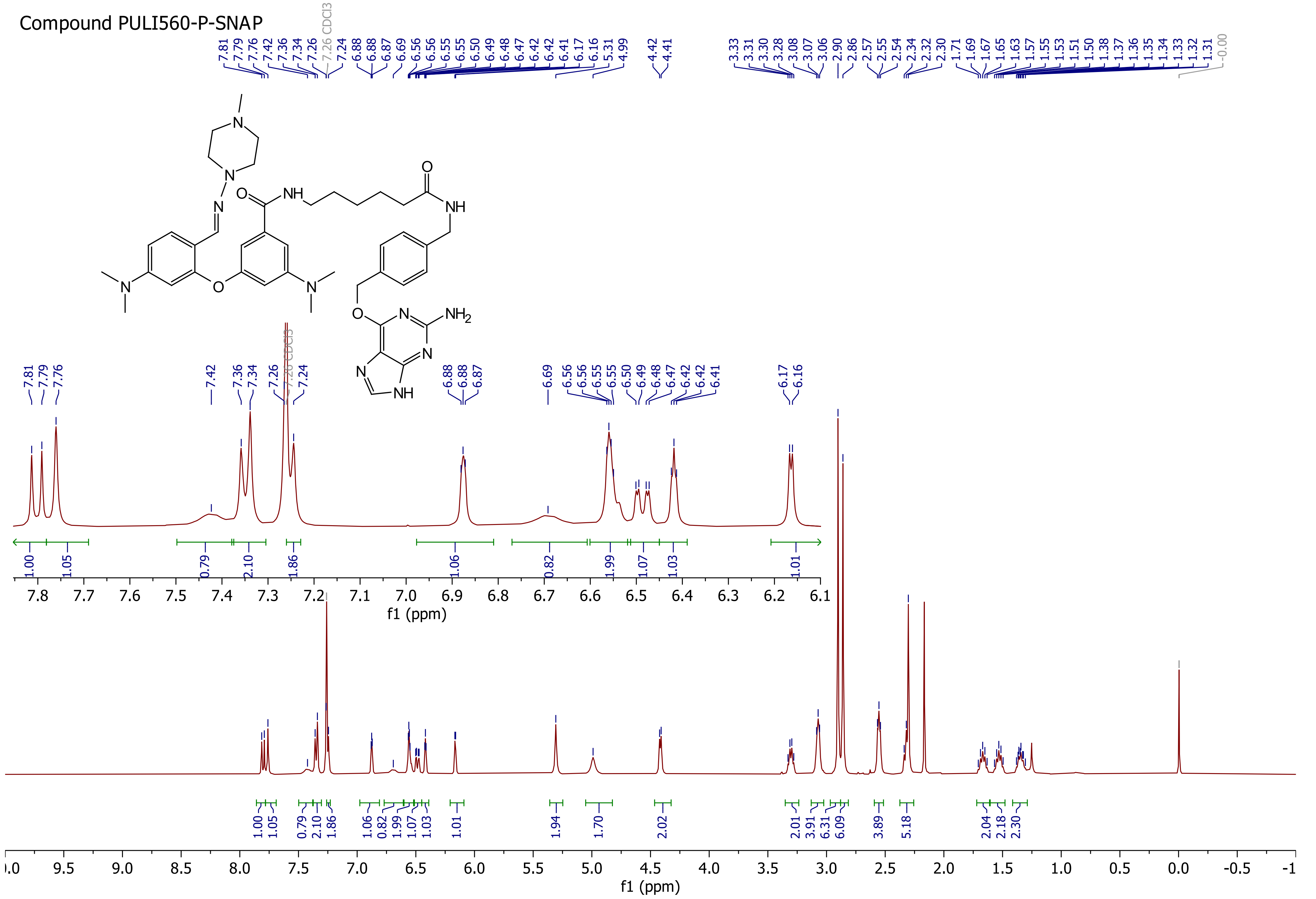

Compound PULI560-P-SNAP

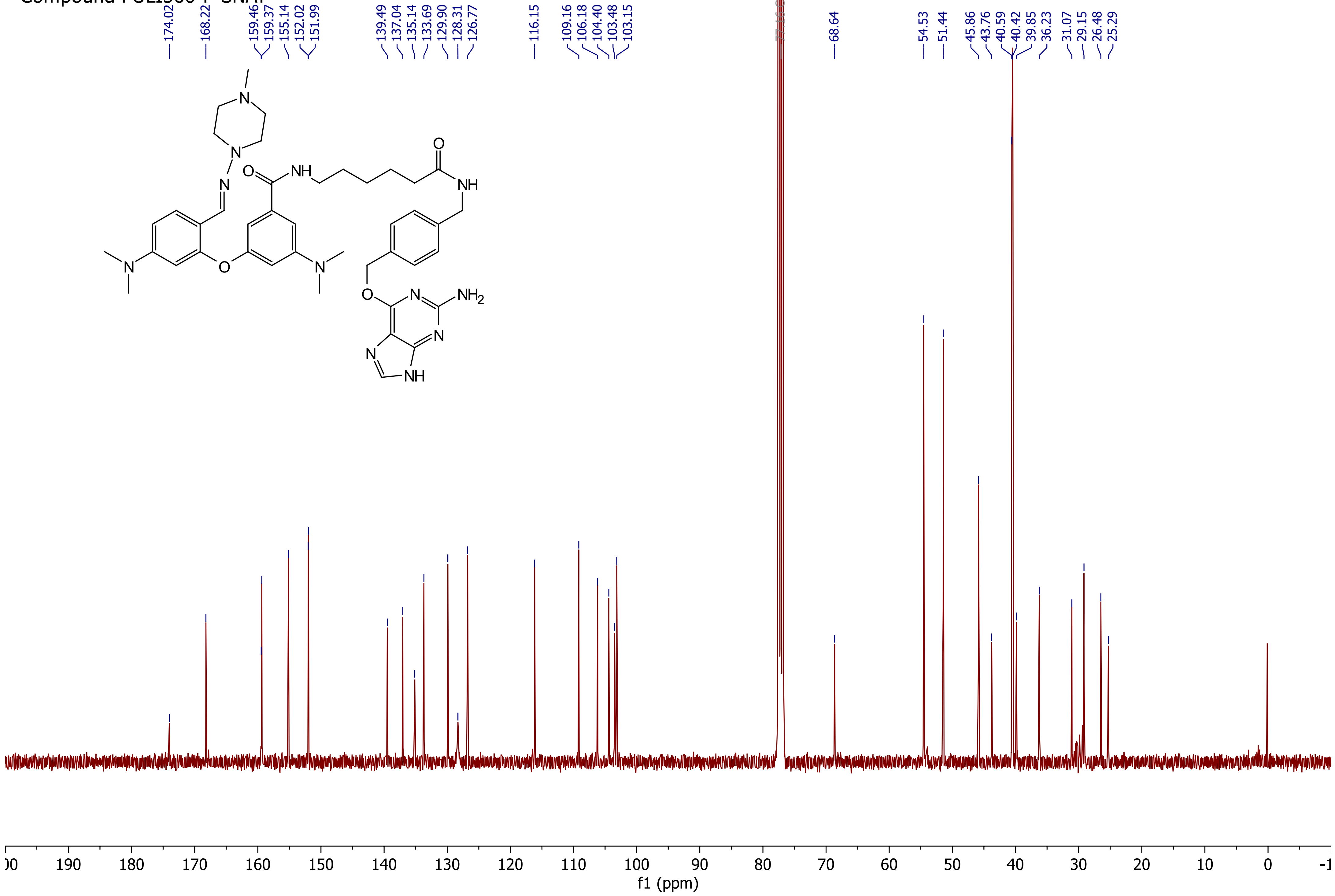

Compound PULI560-T

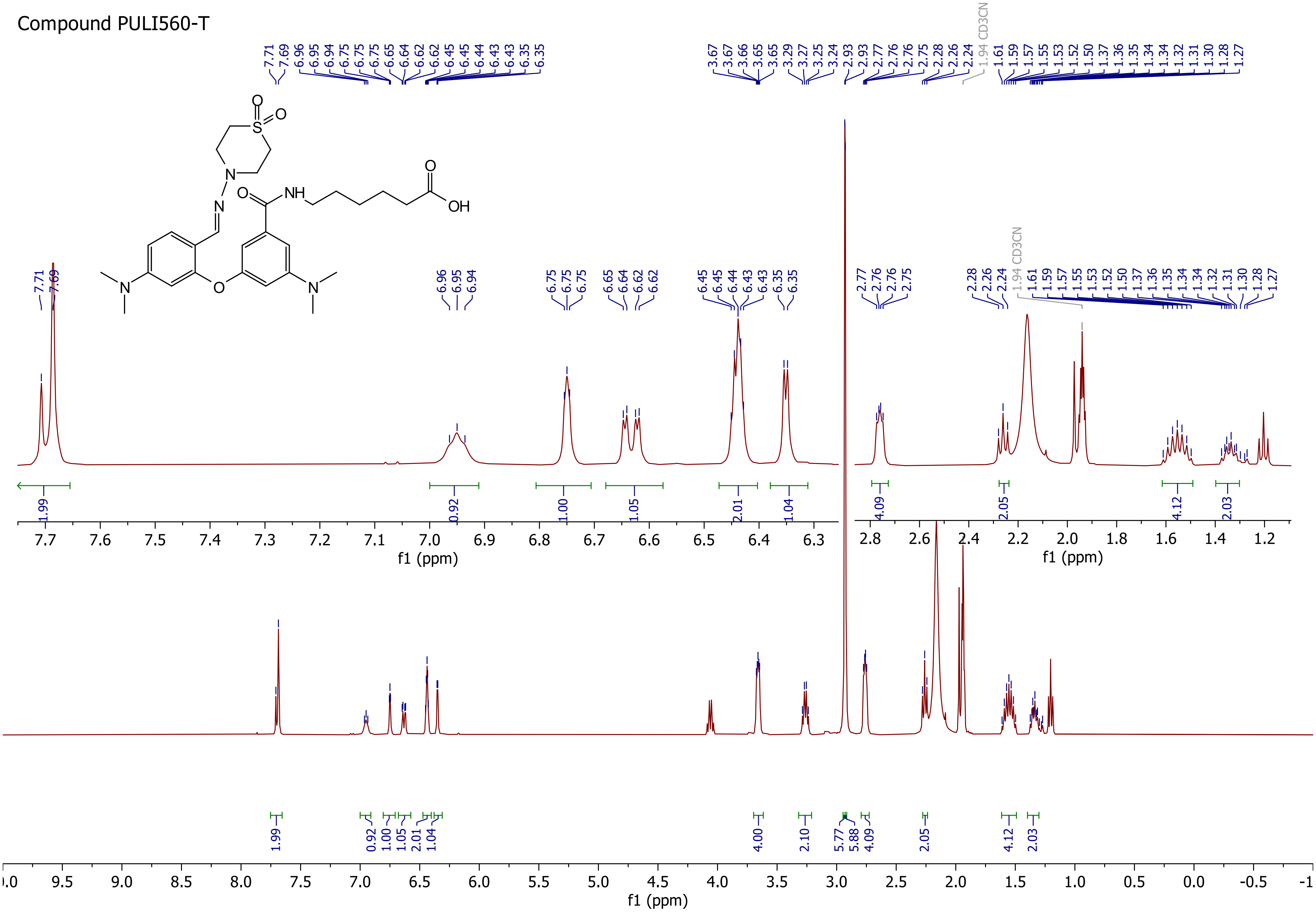

Compound PULI560-T

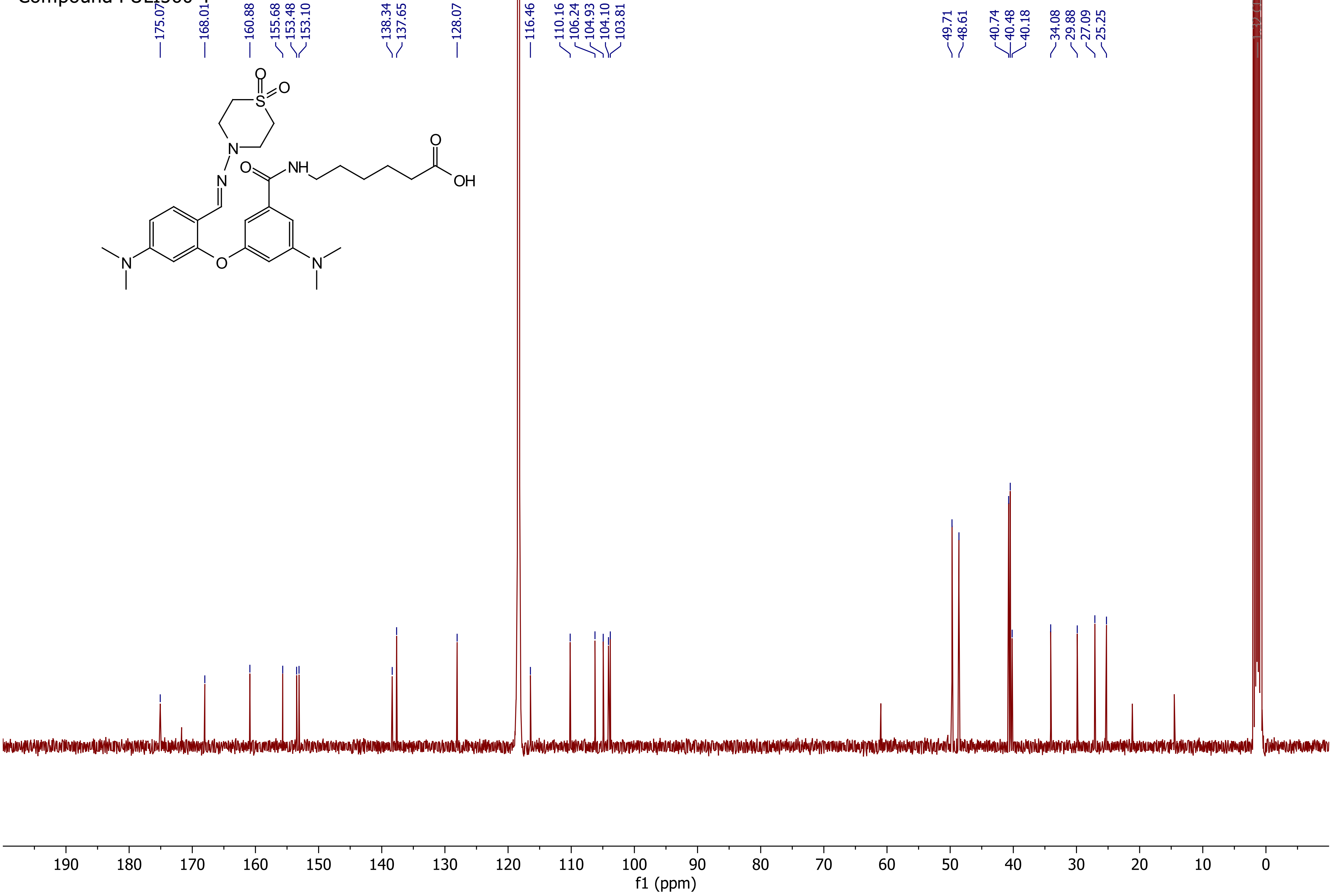

Compound PULI560-T-cabazitaxel

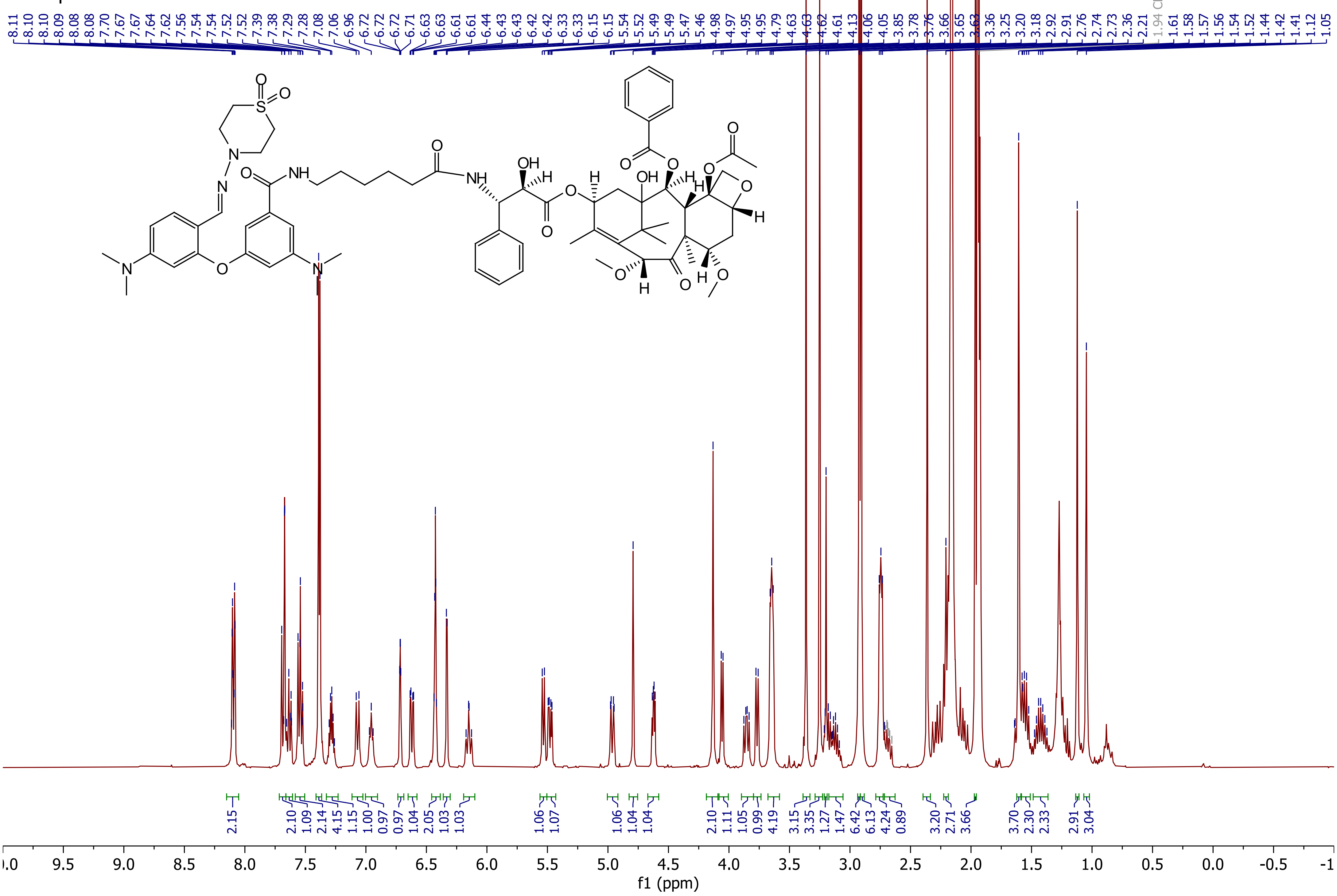

Compound PULI560-T-cabazitaxel

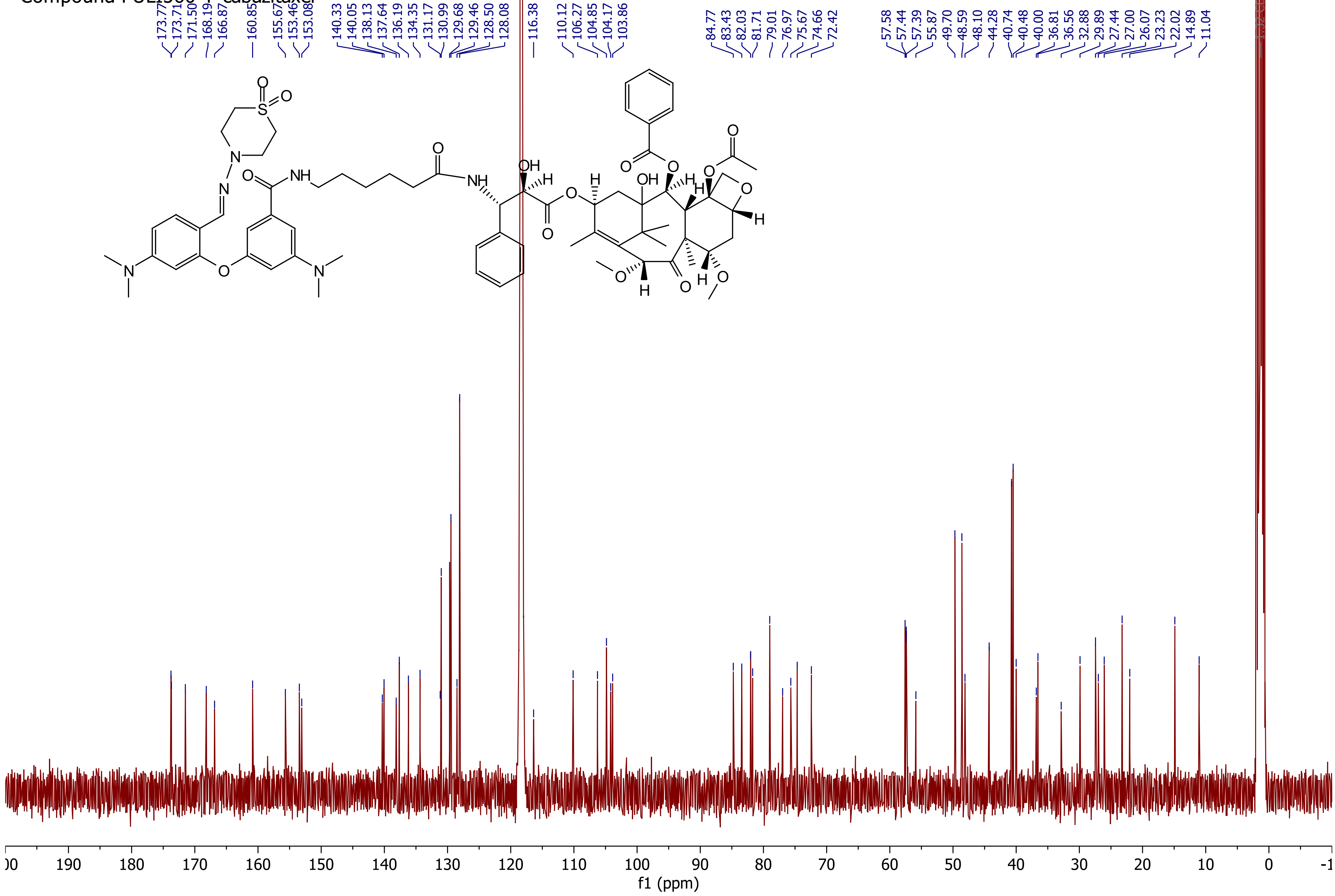

Compound PULI560-T-Halo

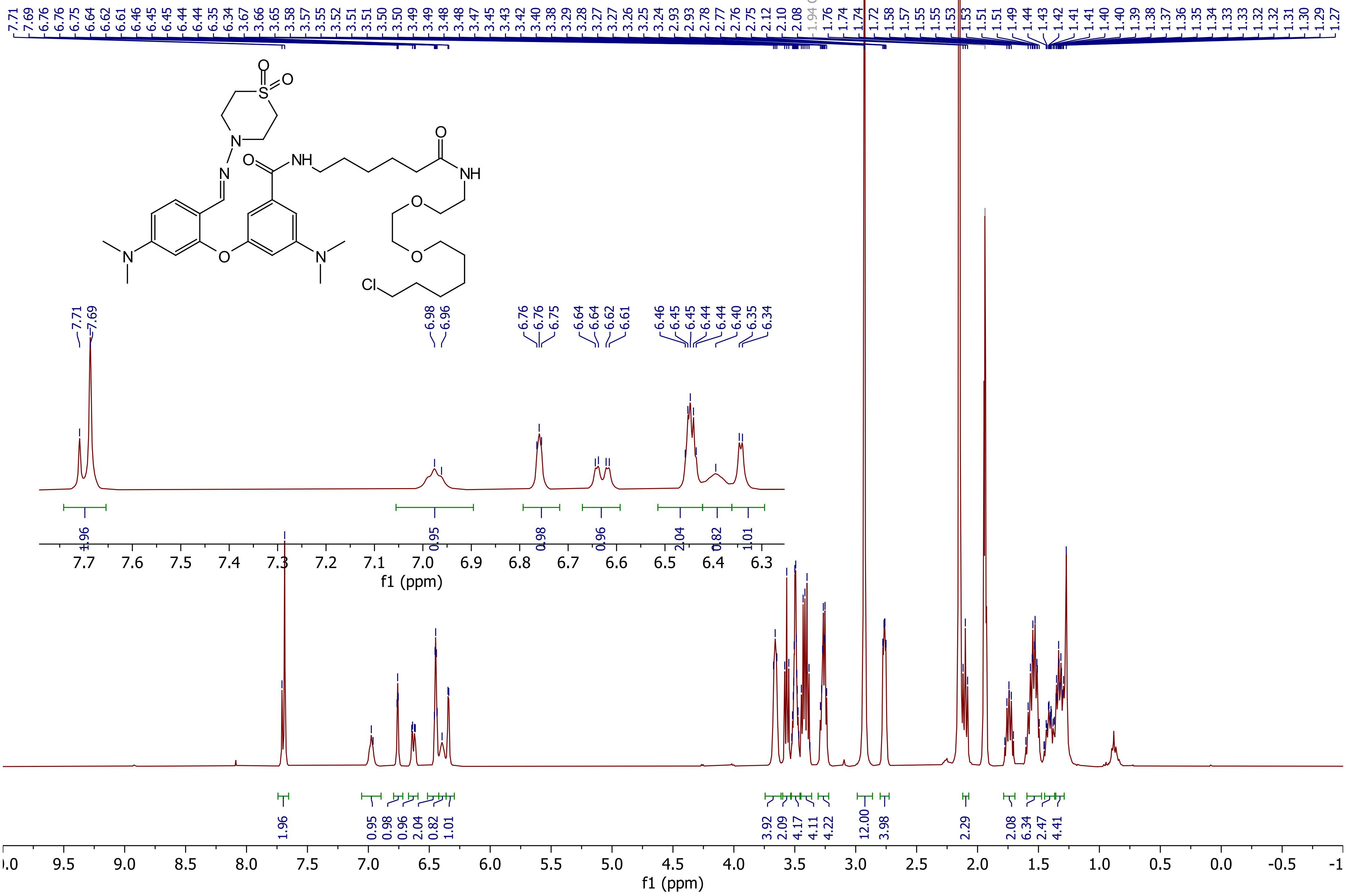

Compound PULI560-T-Halo

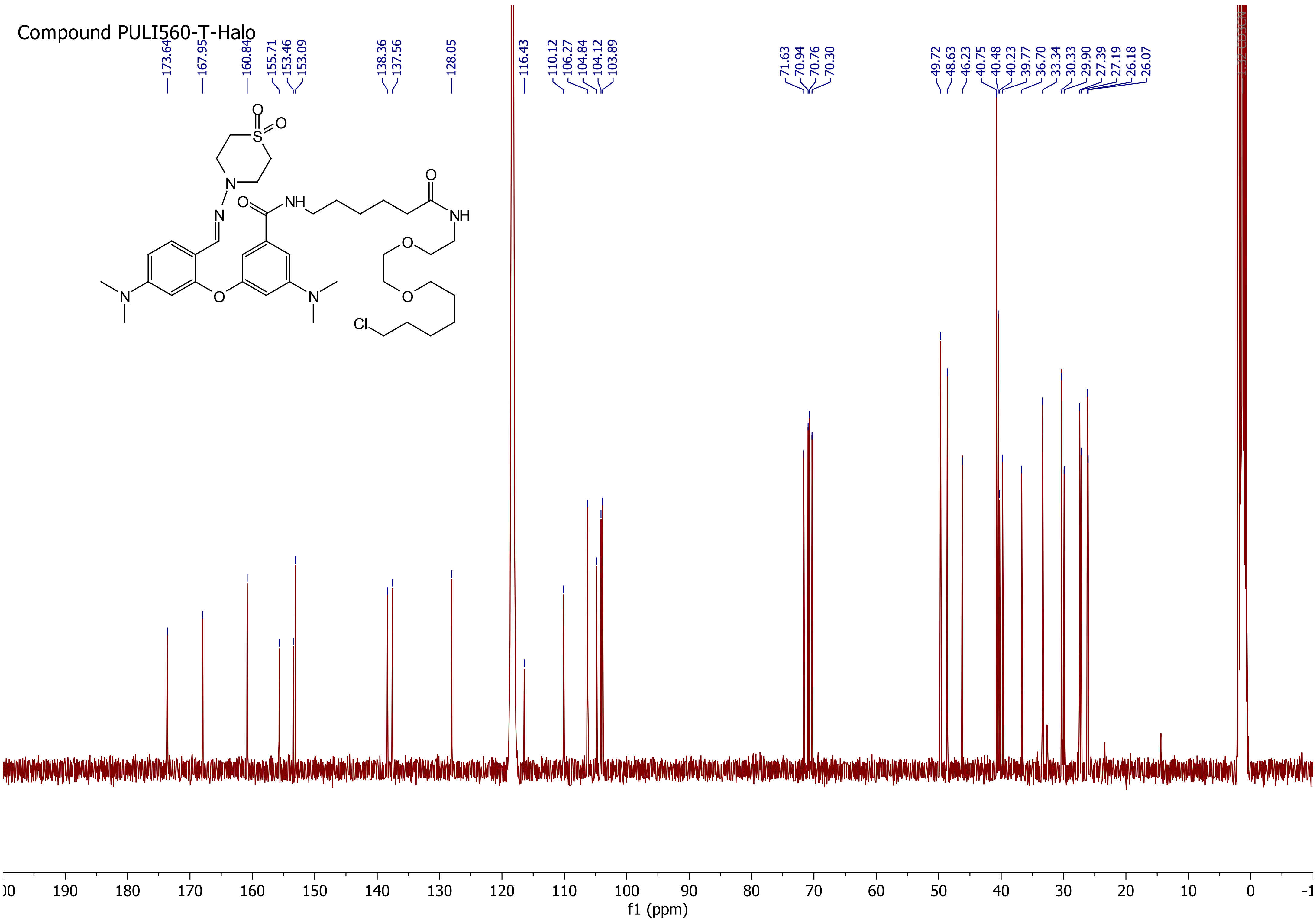

Compound PULI560-T-maleimide

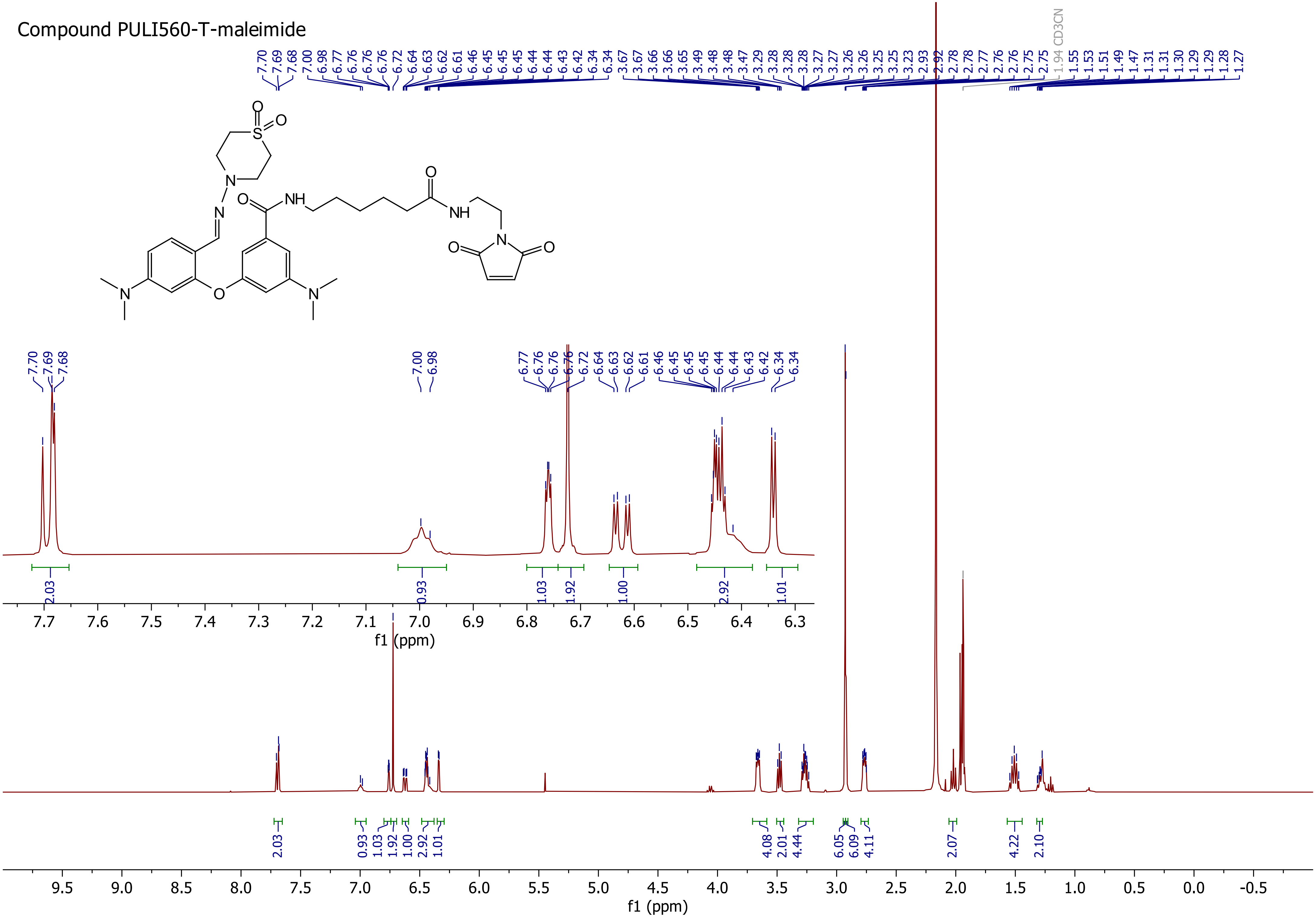

Compound PULI560-T-maleimide

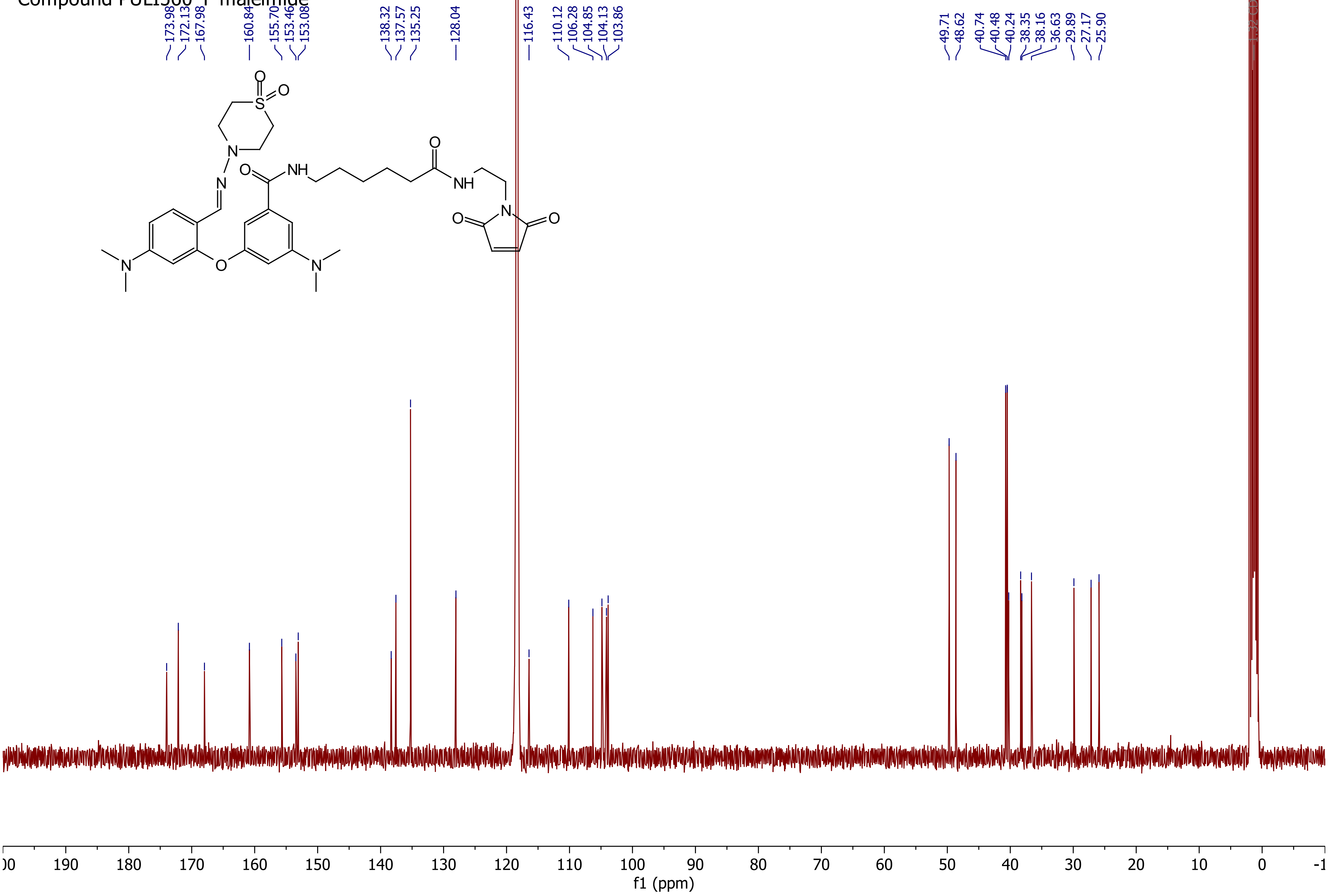

Compound PULI560-T-NHS

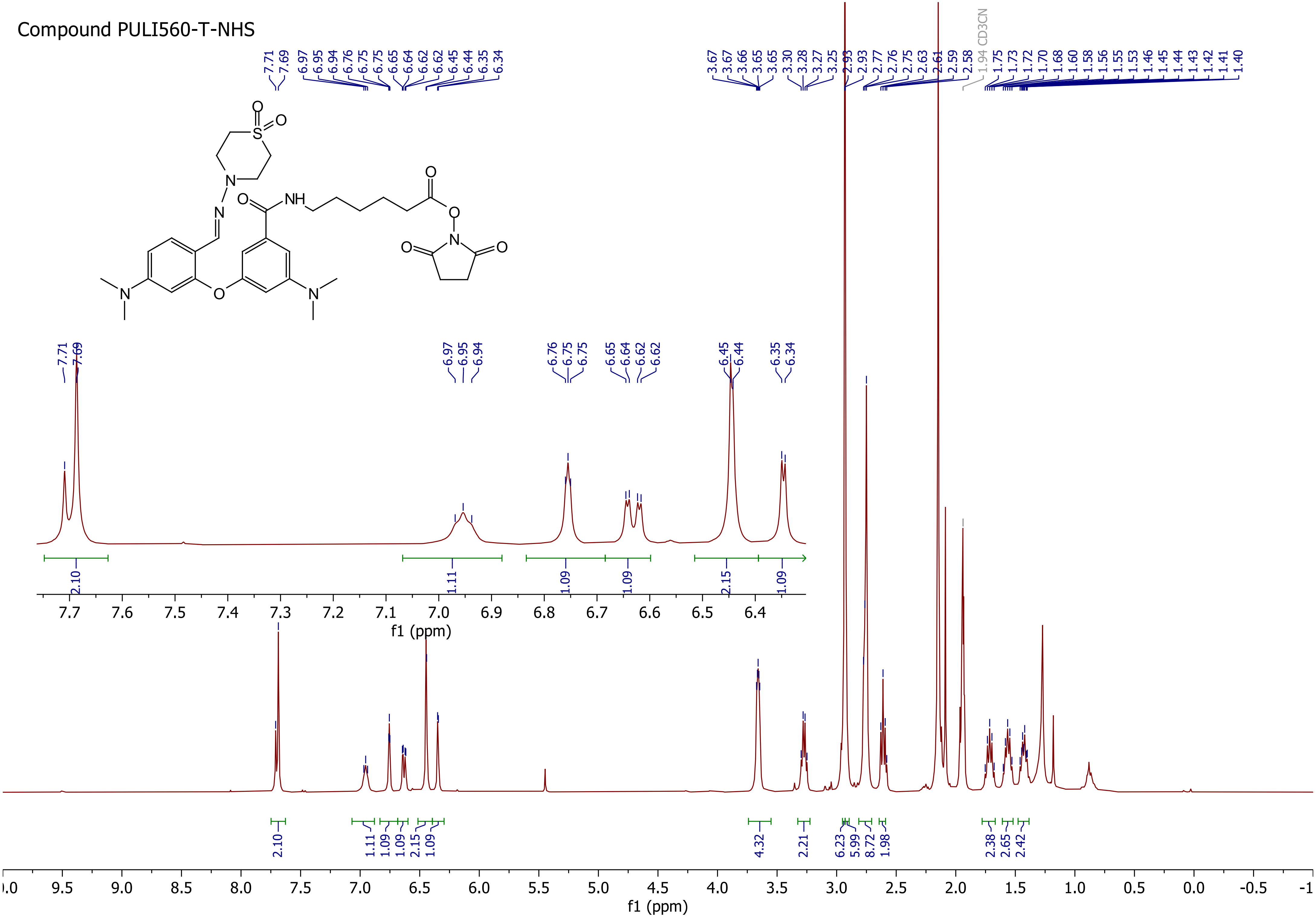

Compound PULI560-T-NHS

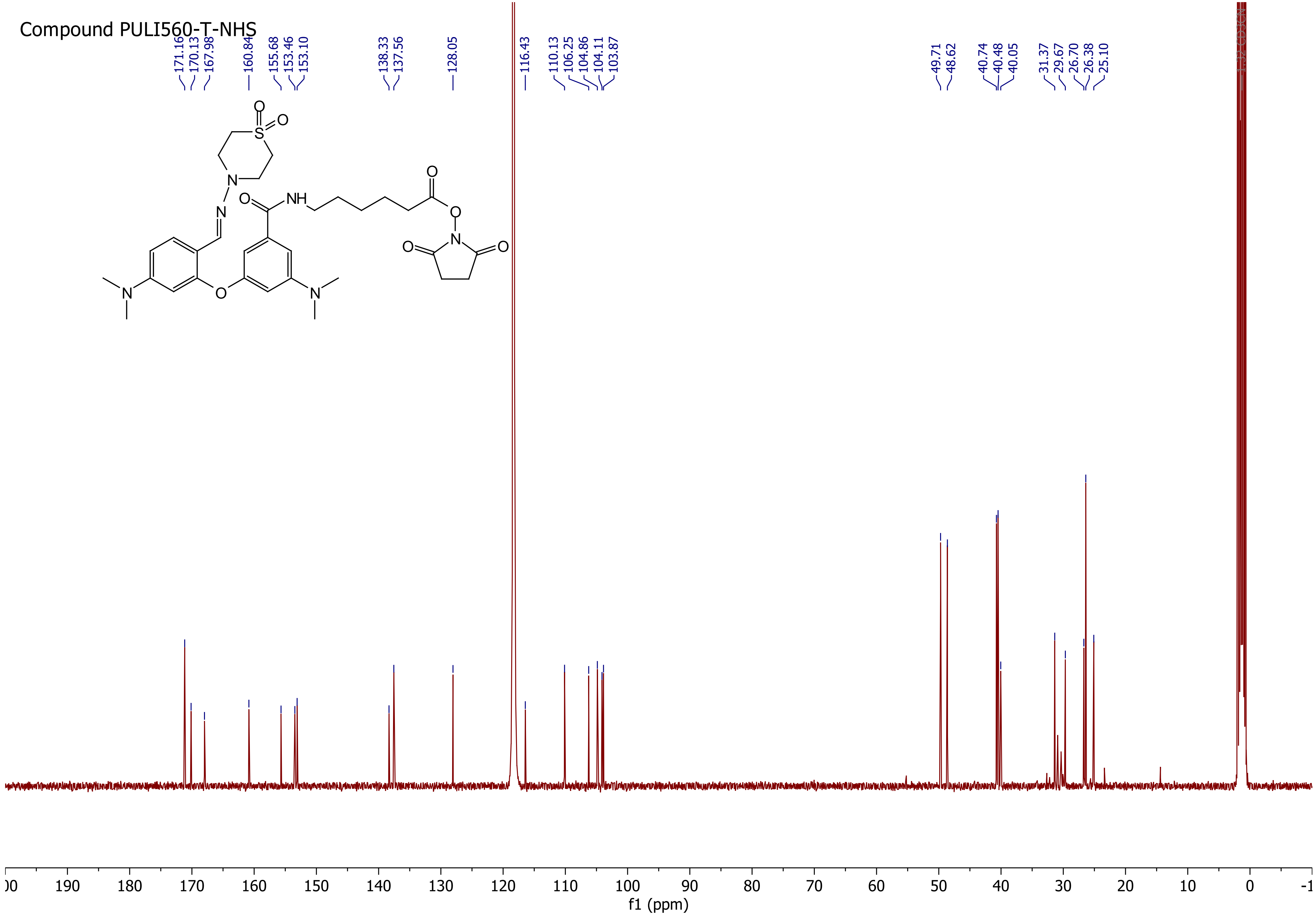

Compound PULI640

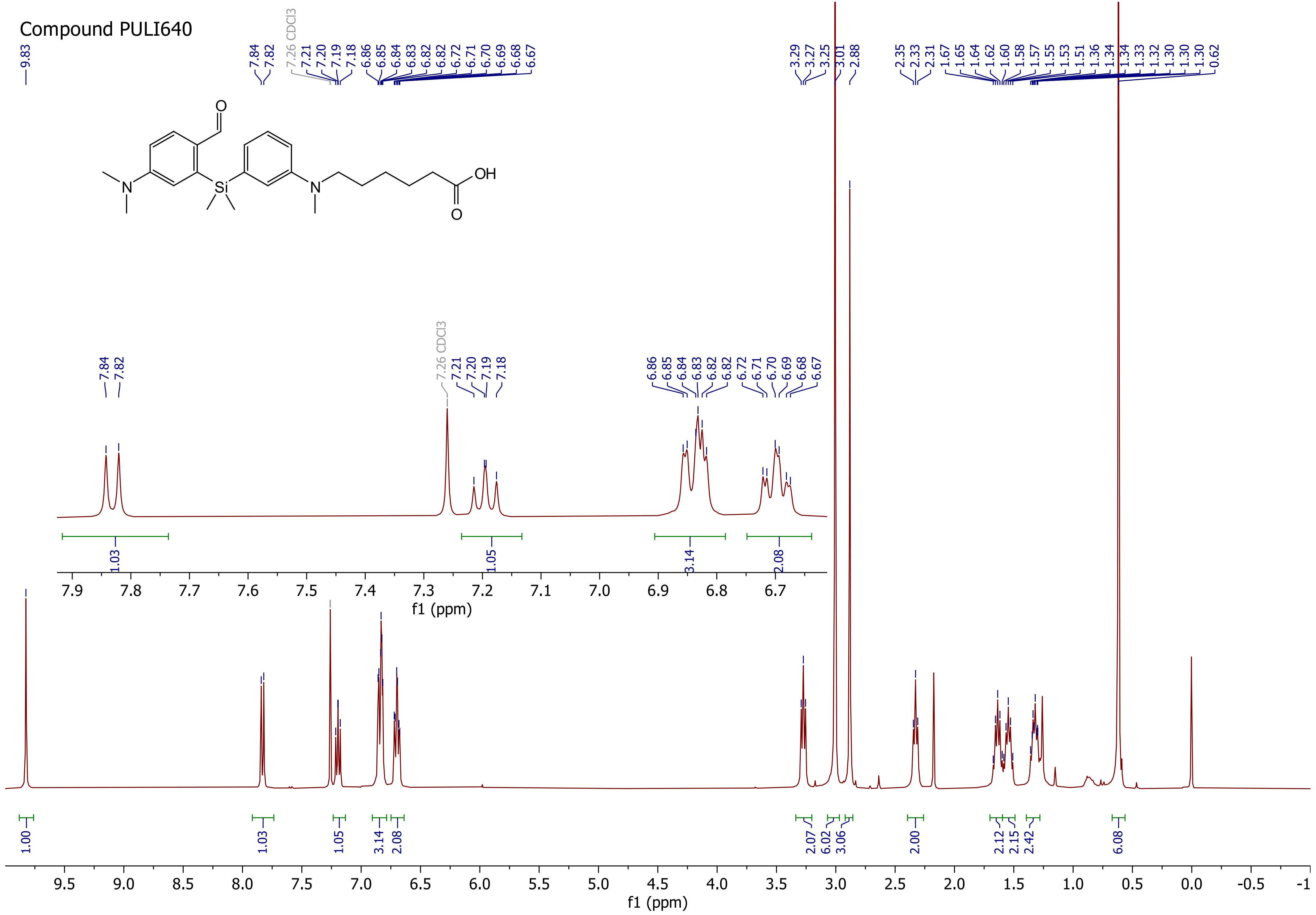

Compound PULI640

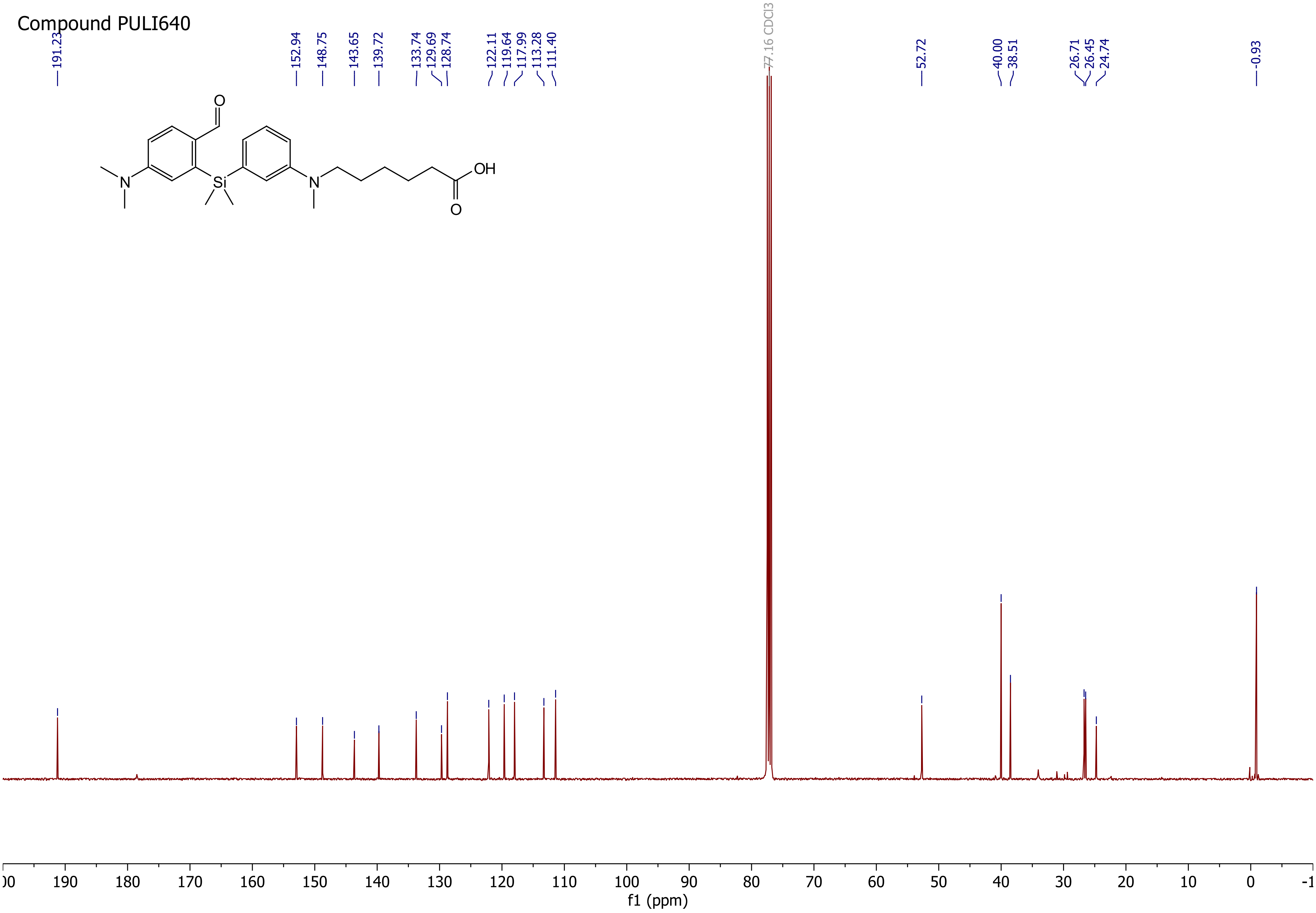

Compound PULI640-M

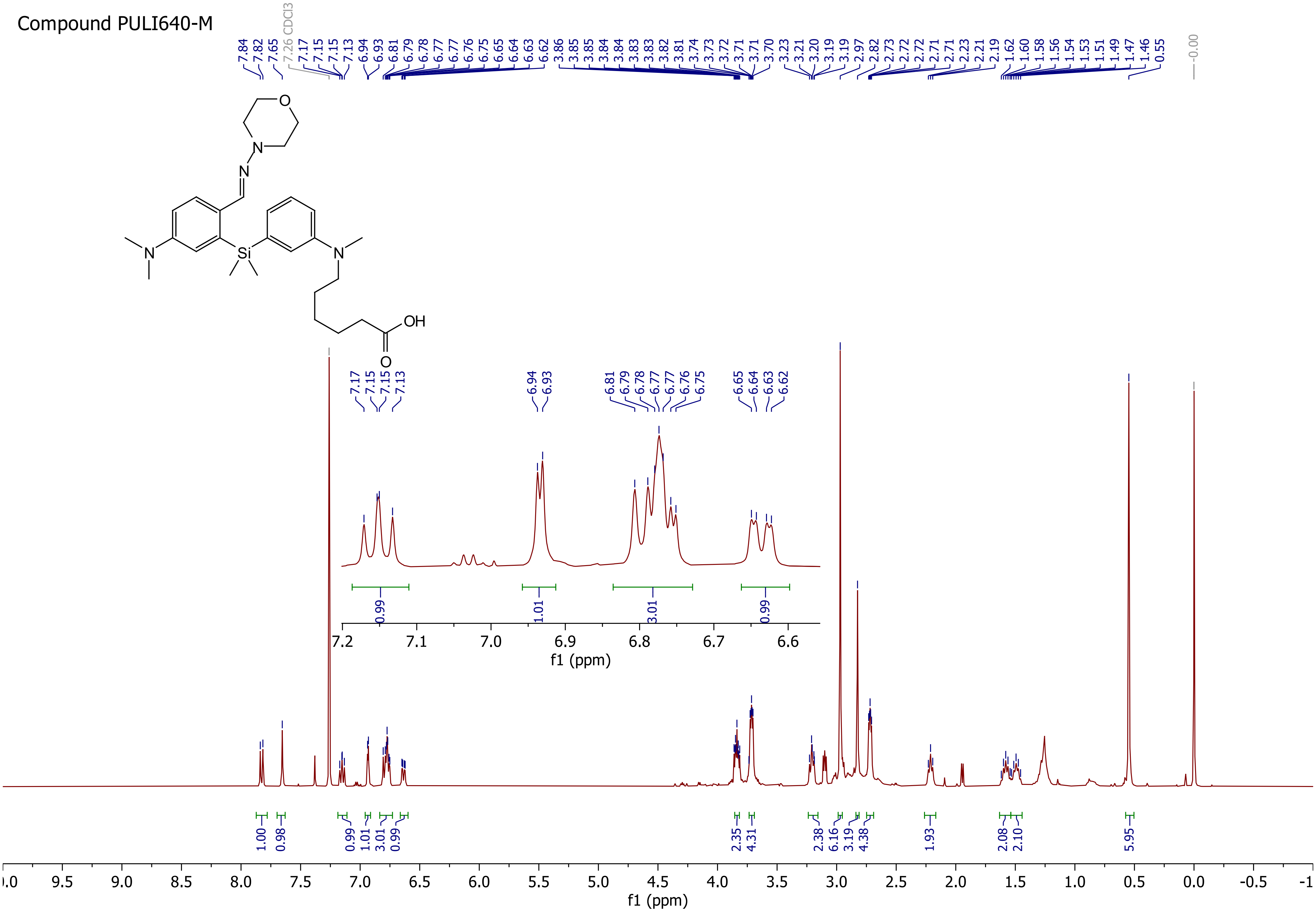

Compound PULI640-M

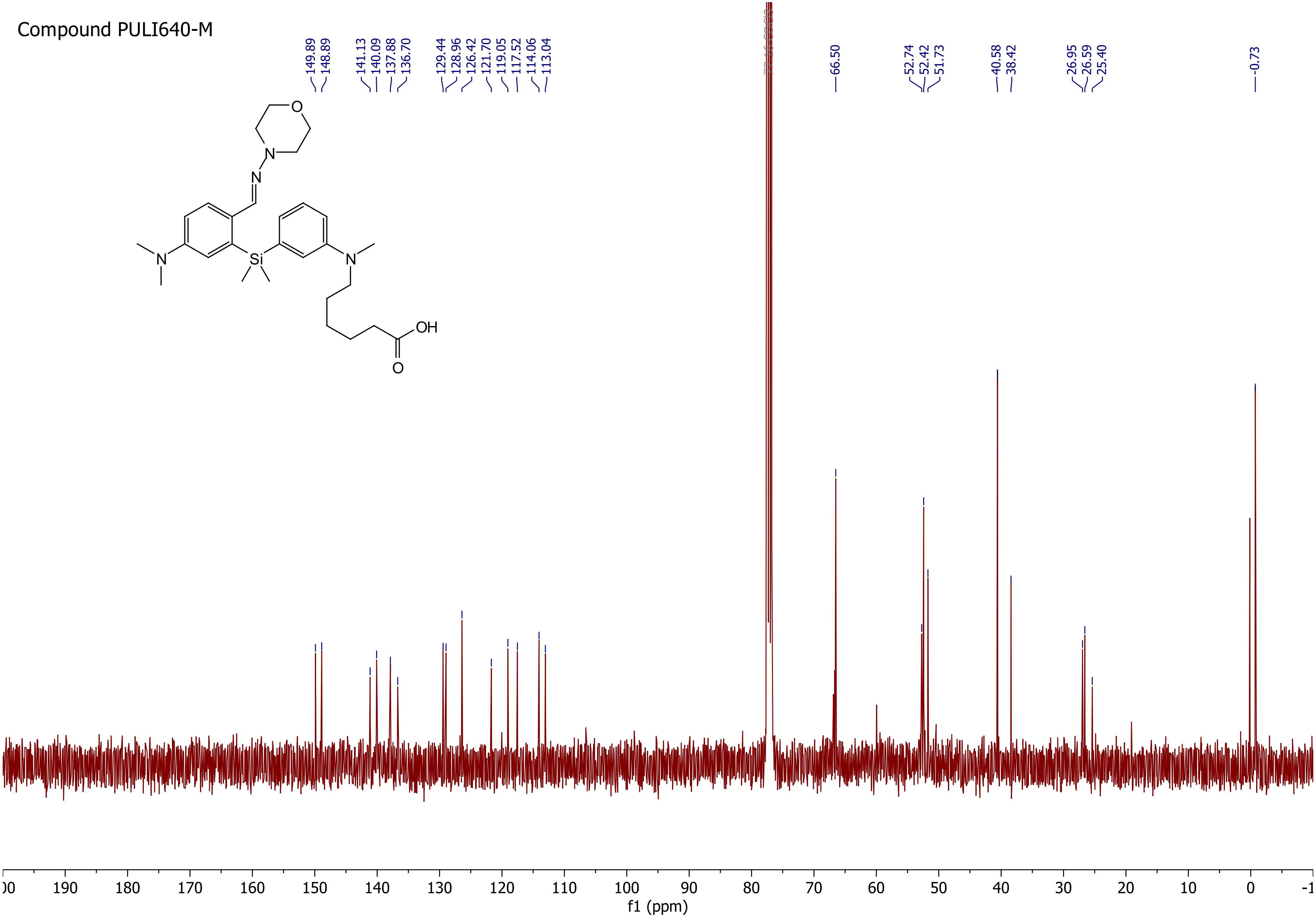

Compound PULI640-M-Halo

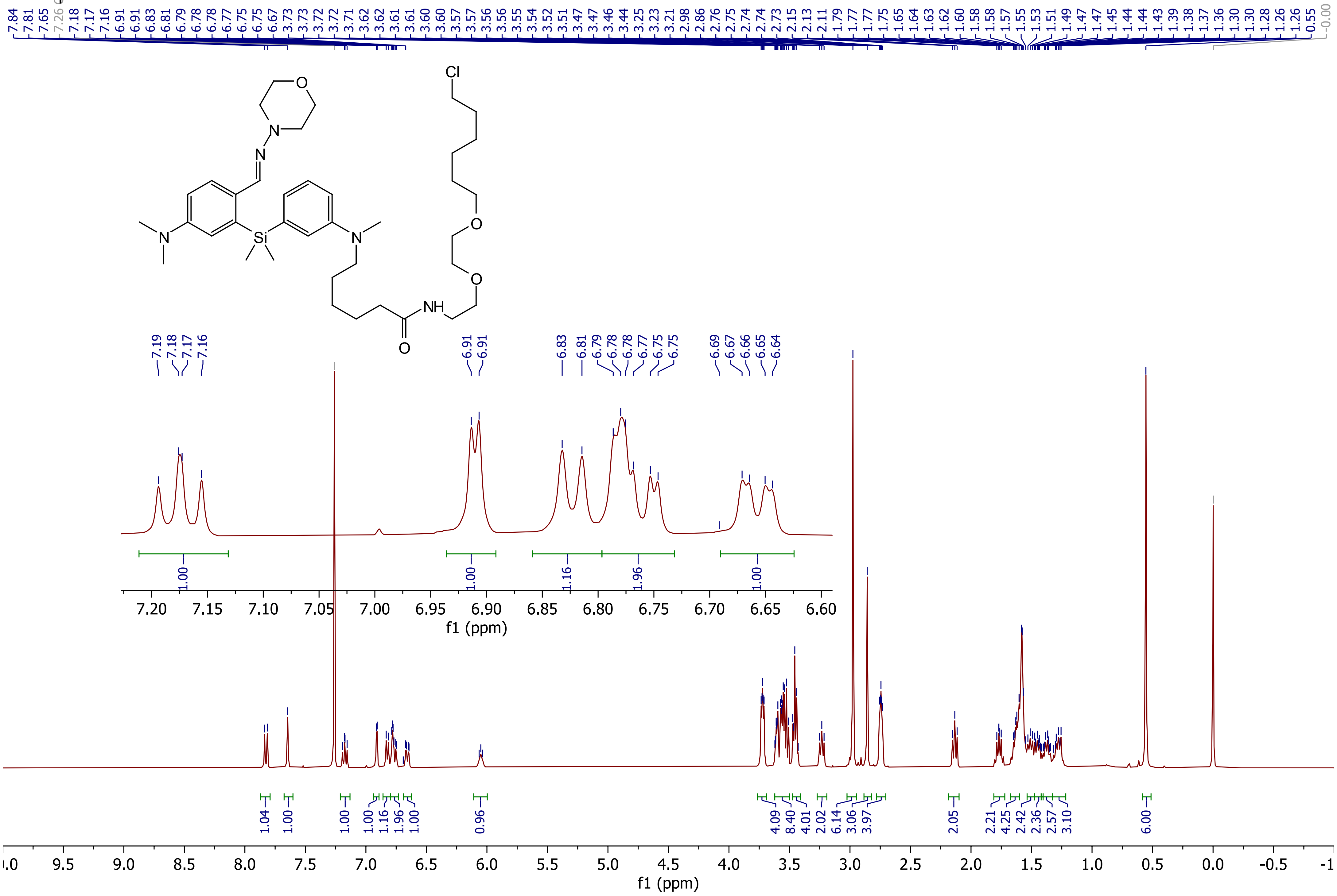

Compound PULI640-M-Halo

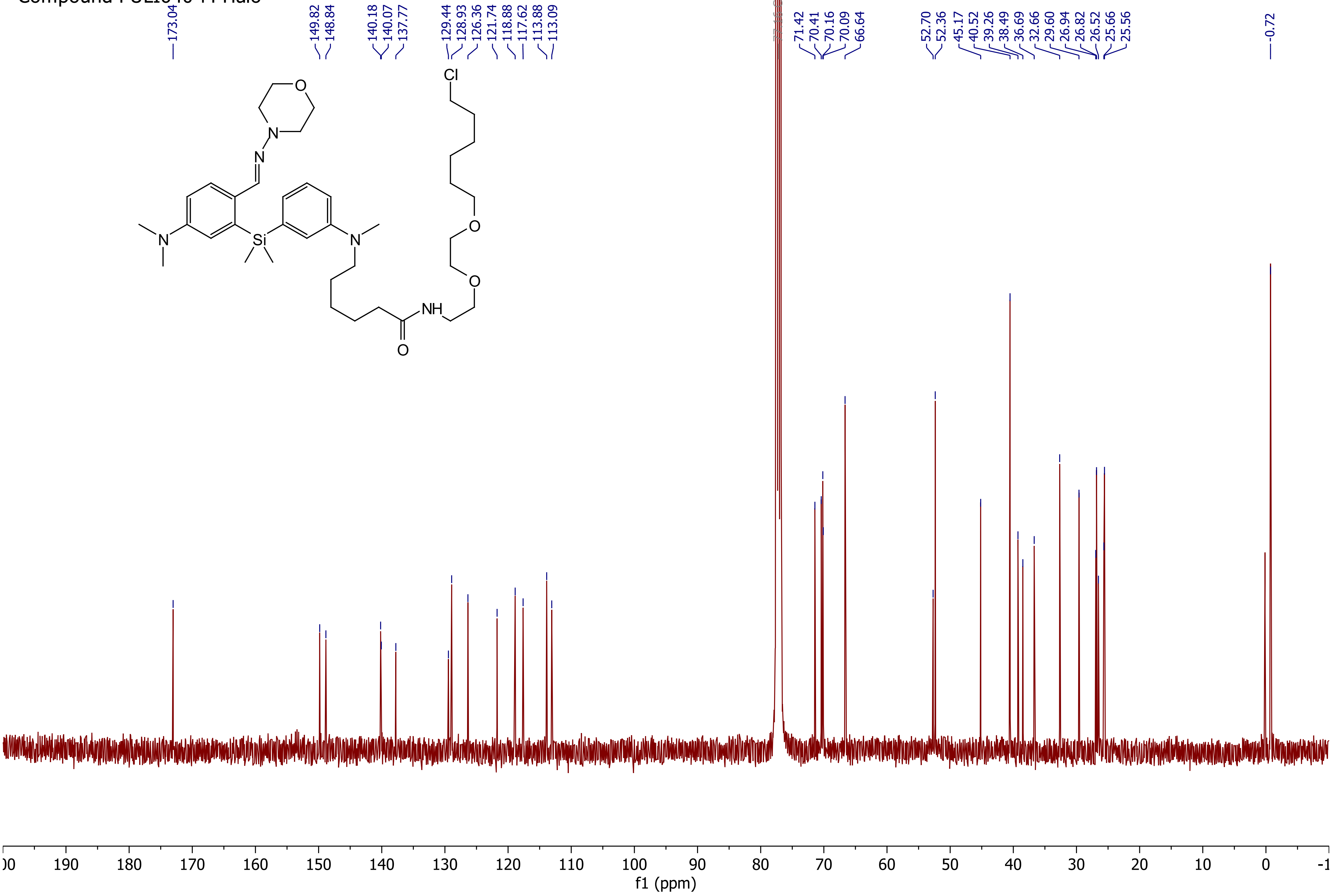

Compound PULI640-M-NHS

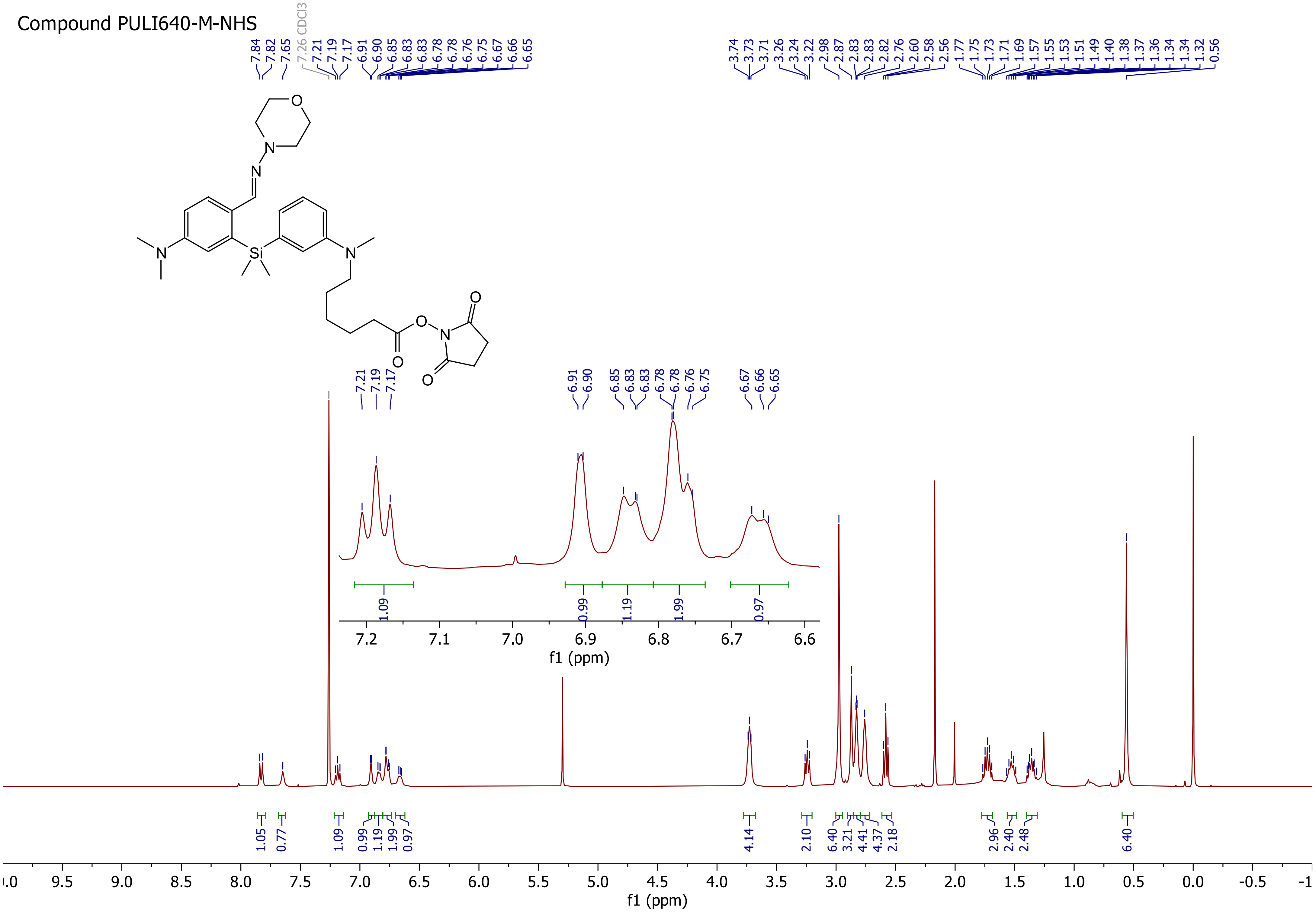

Compound PULI640-M-NHS

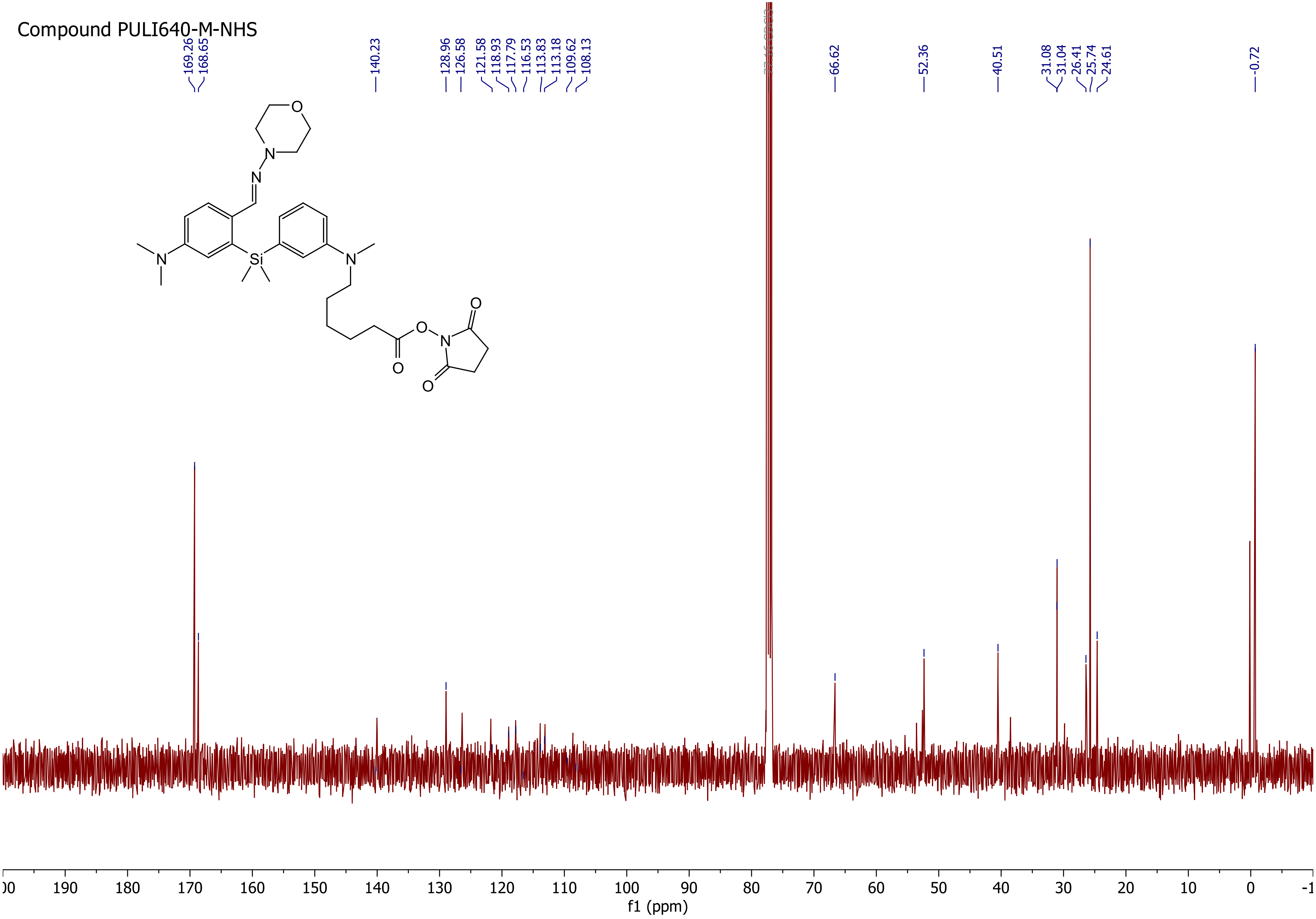

Compound PULI640-M-SNAP

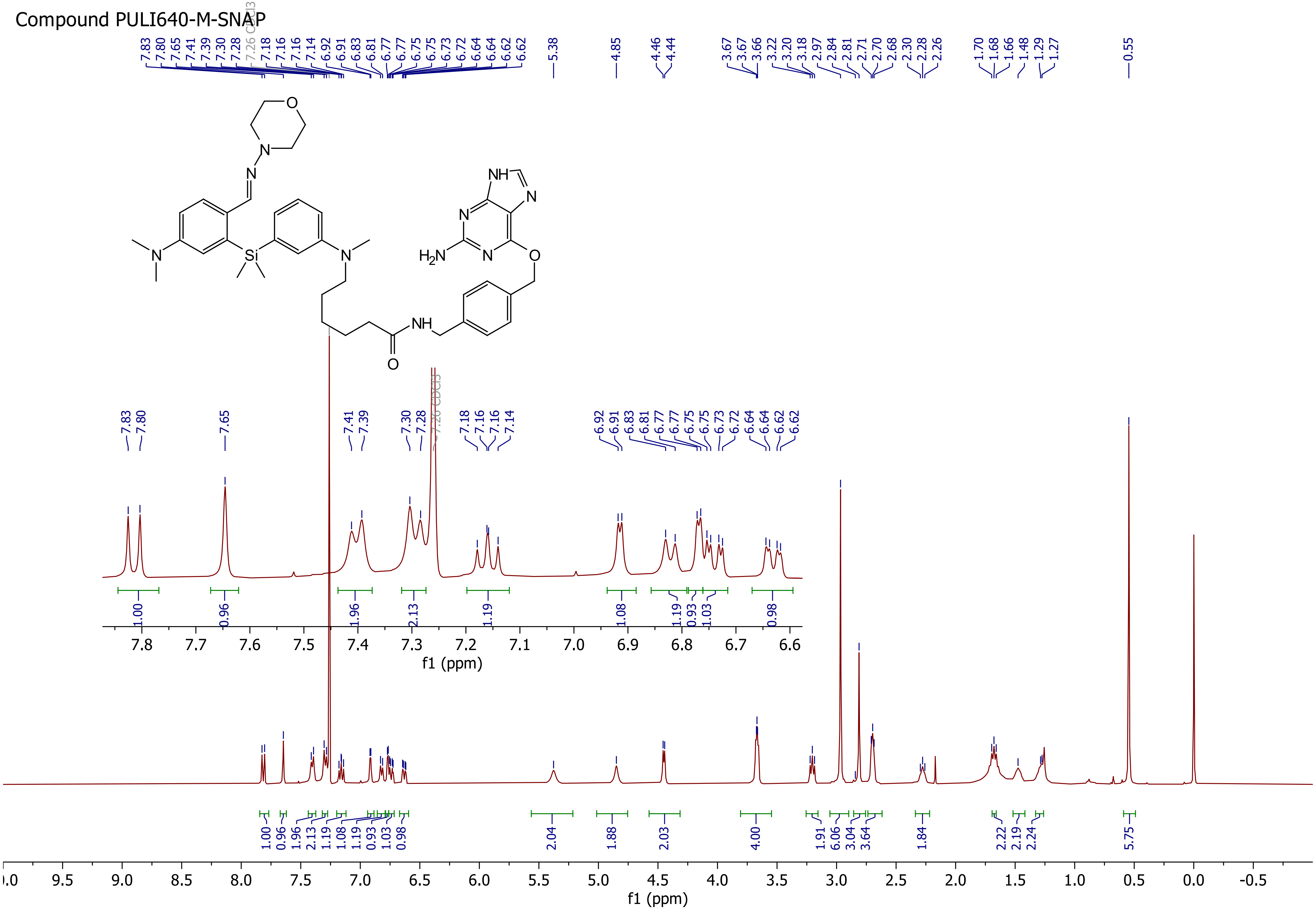

Compound PULI640-M-SNAP

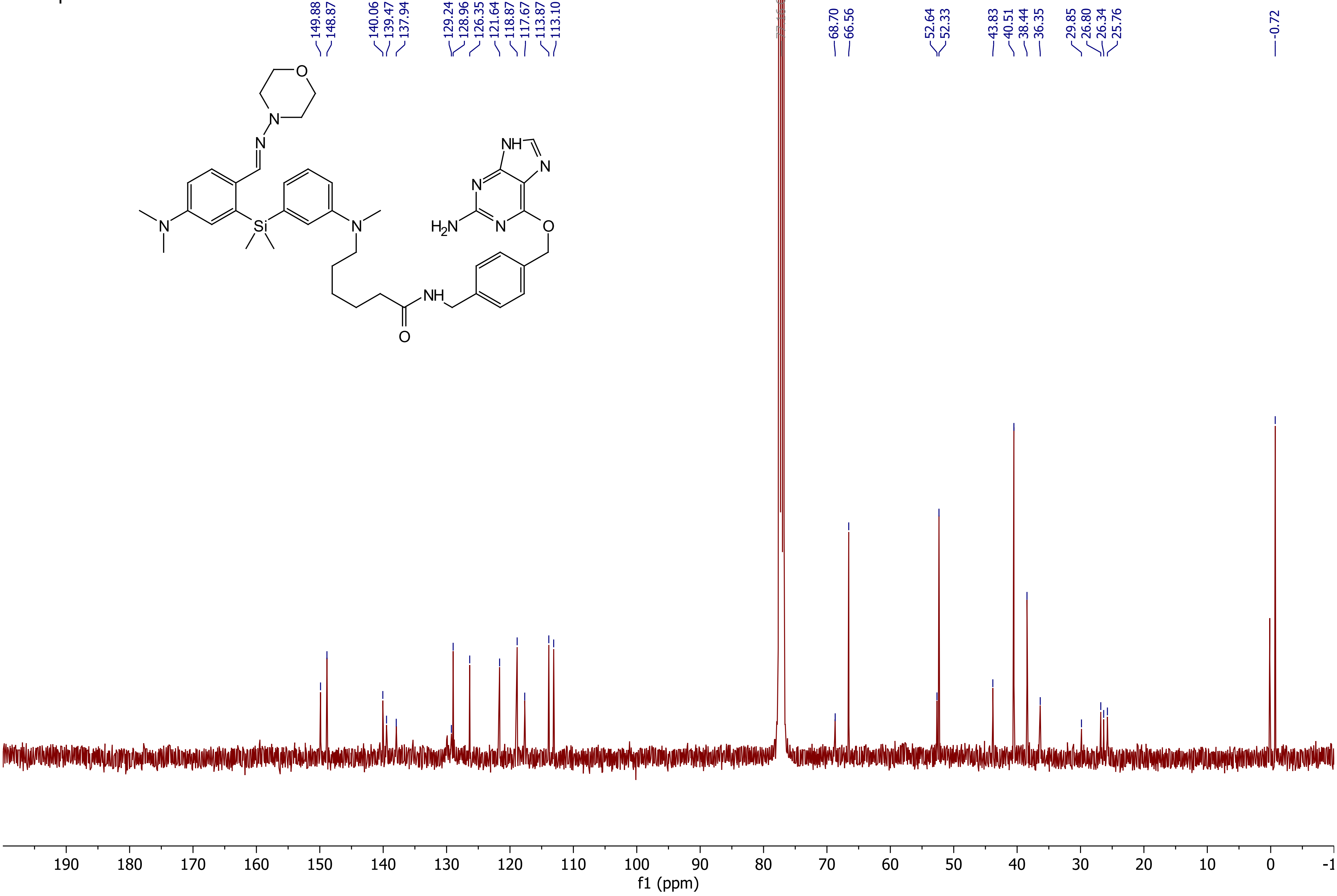

Compound PULI640-P

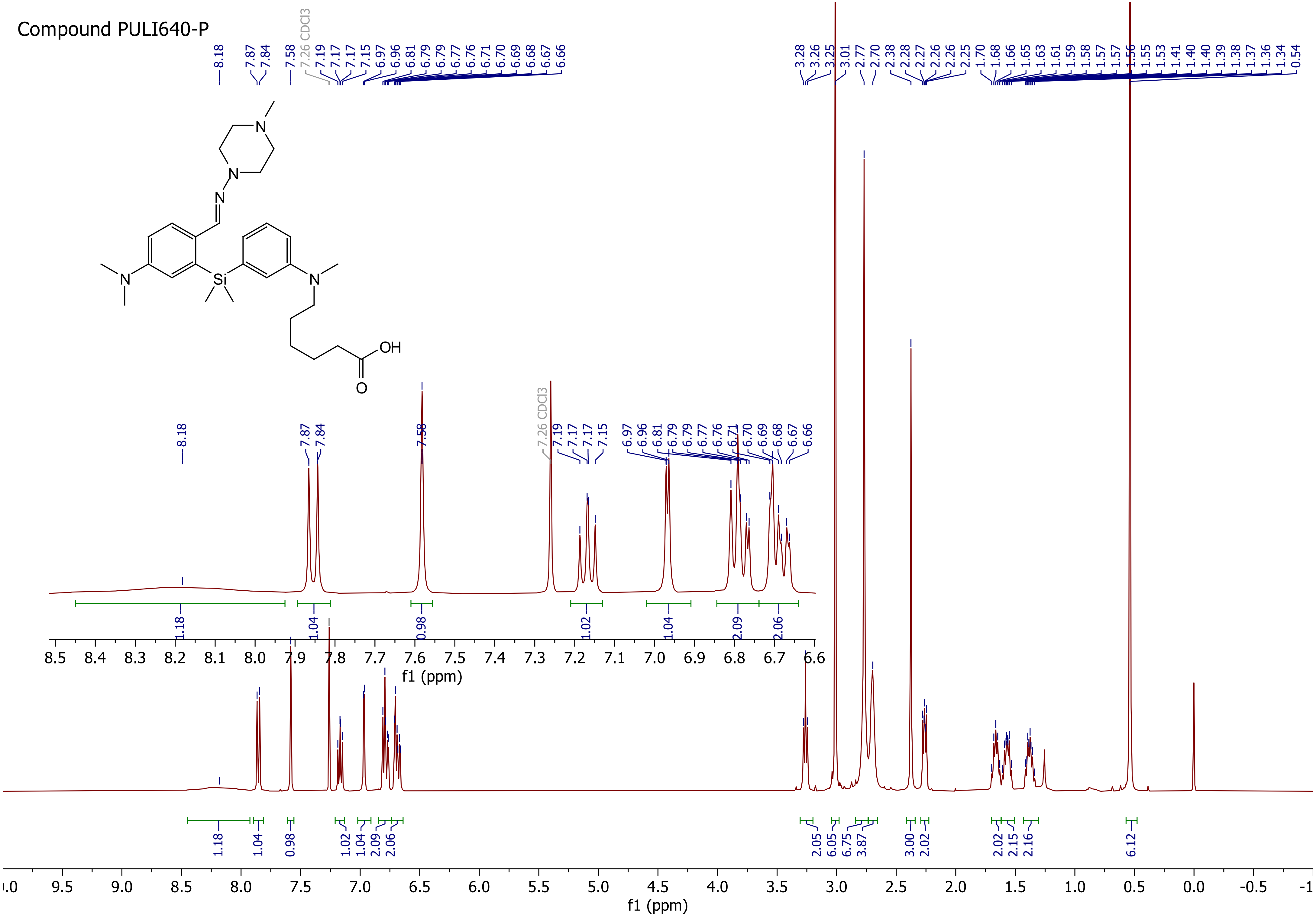

Compound PULI640-P

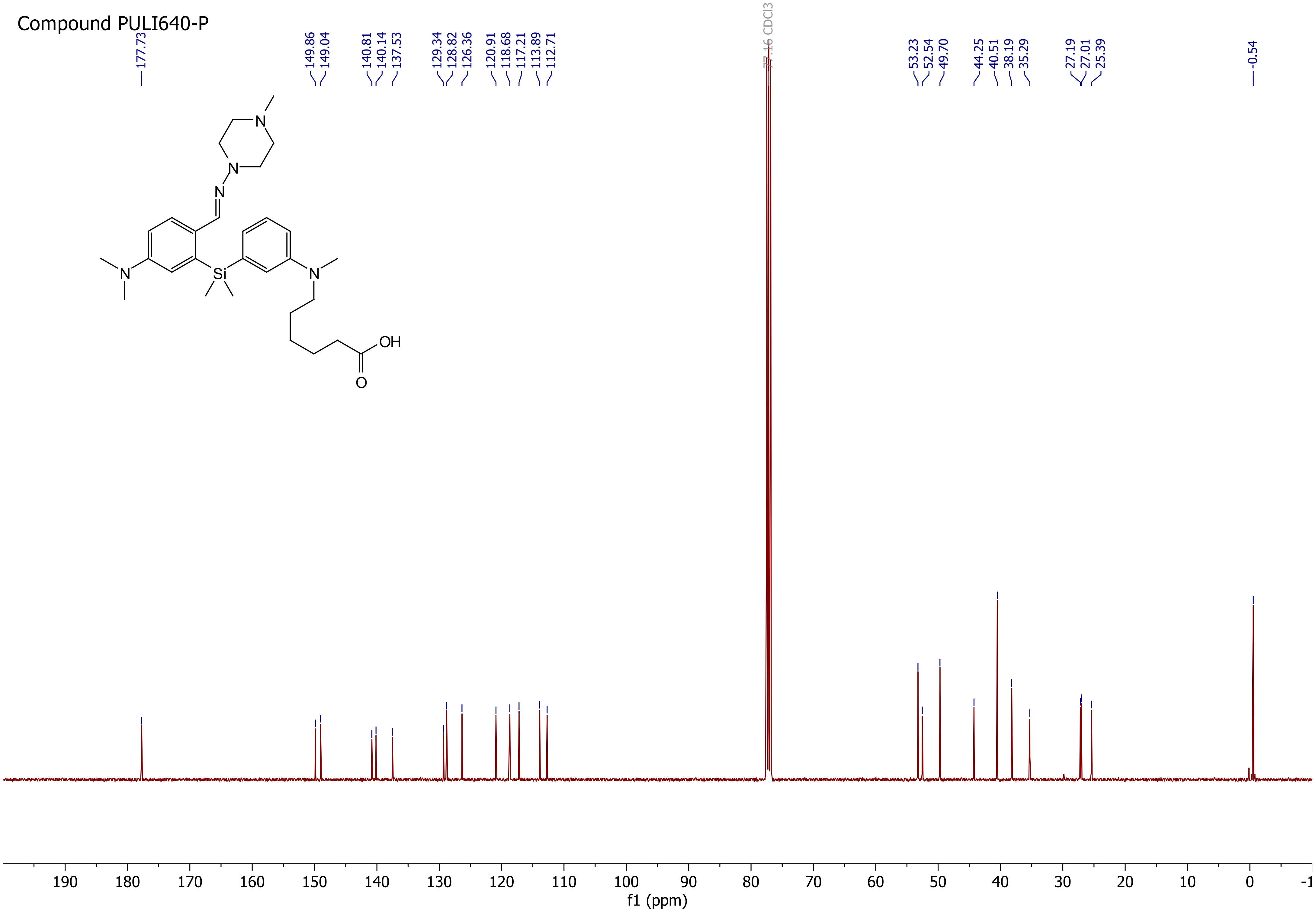

Compound PULI640-P-Halo

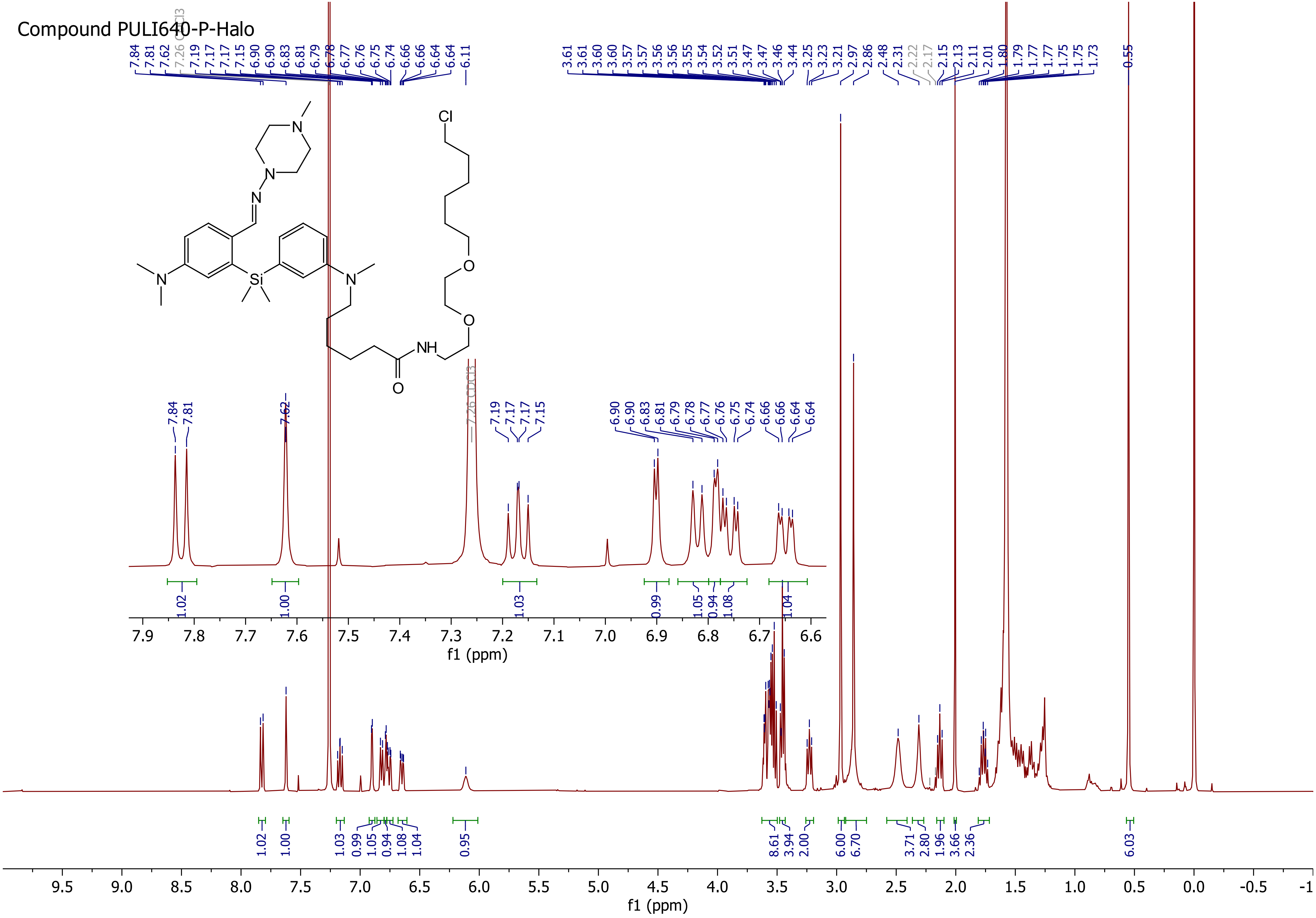

Compound PULI640-P-Halo

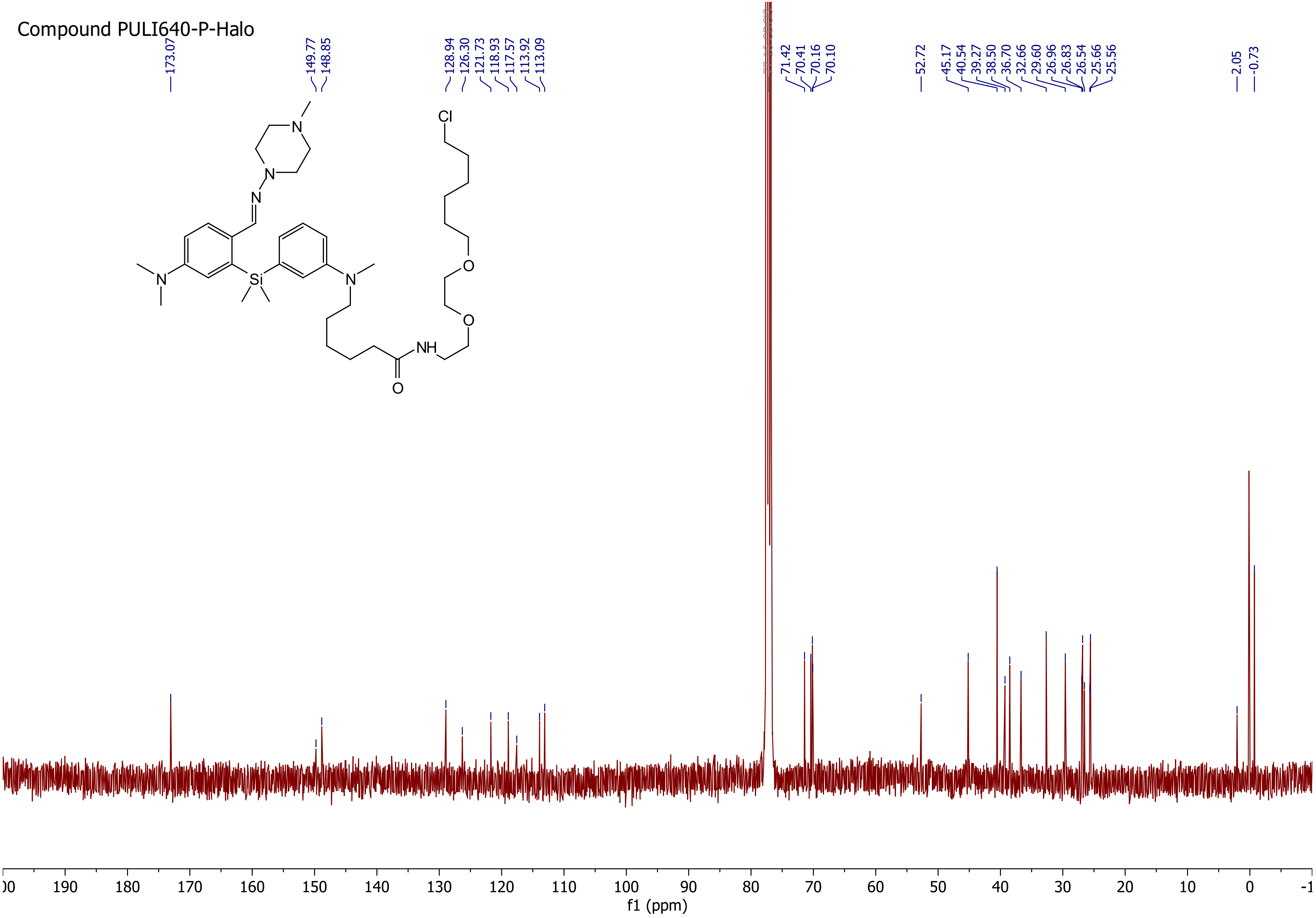

Compound PULI640-P-NHS

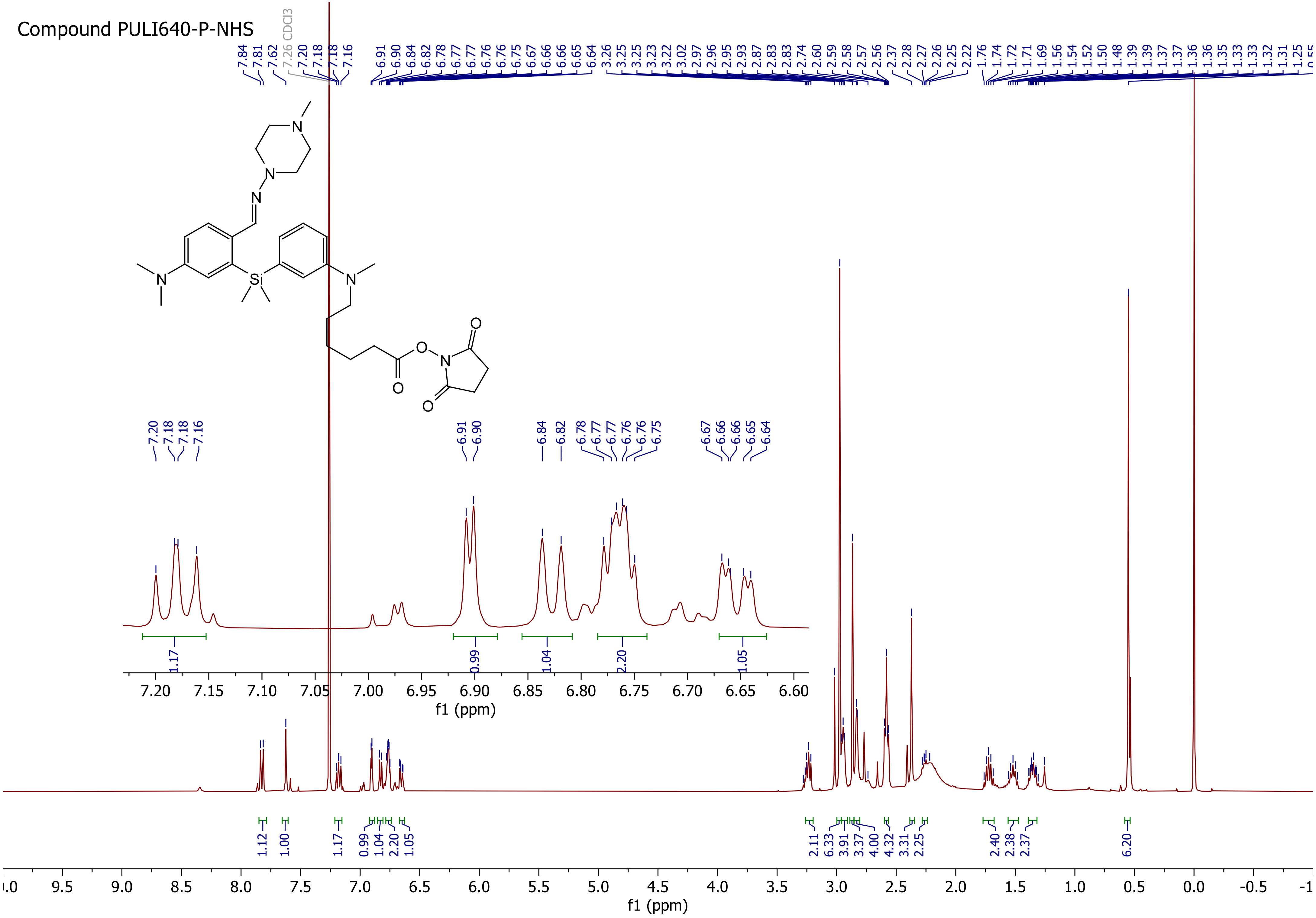

Compound PULI640-P-NHS

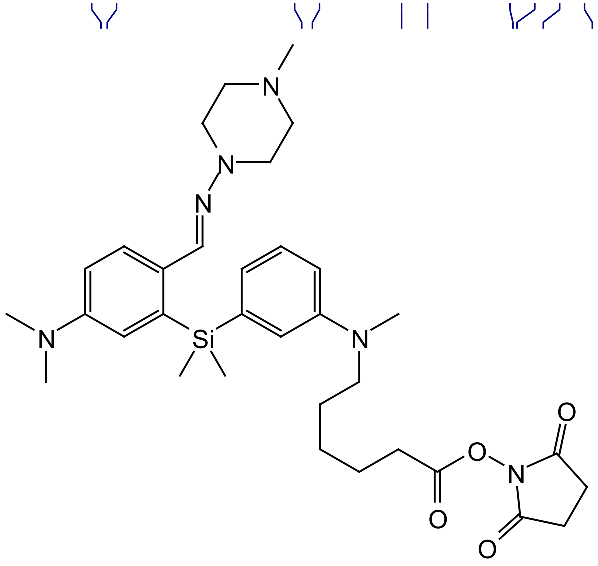

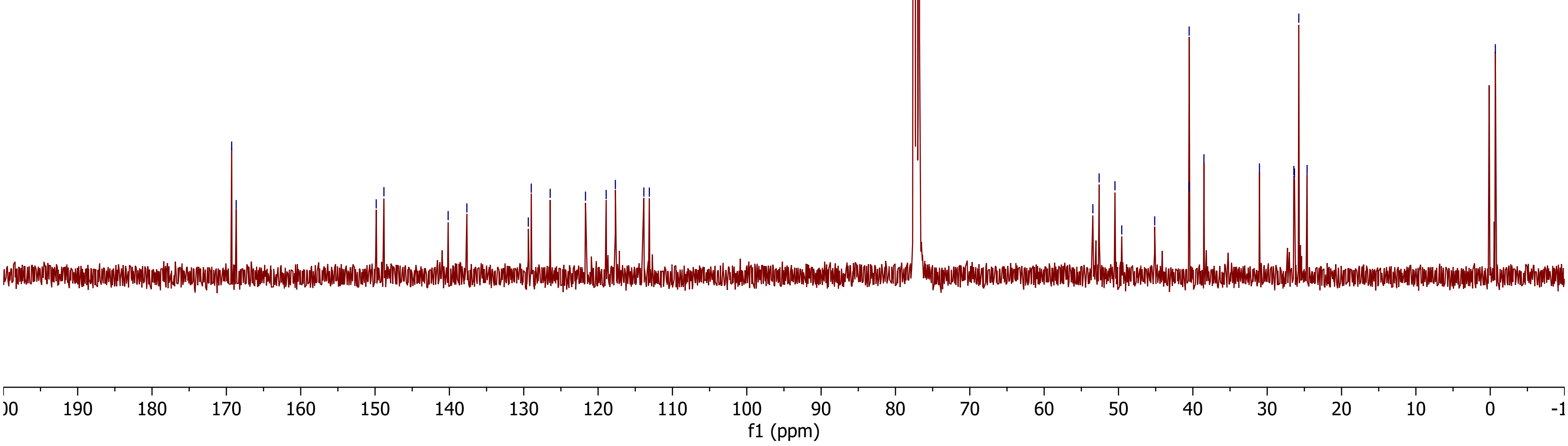

Compound PULI640-P-SNAP

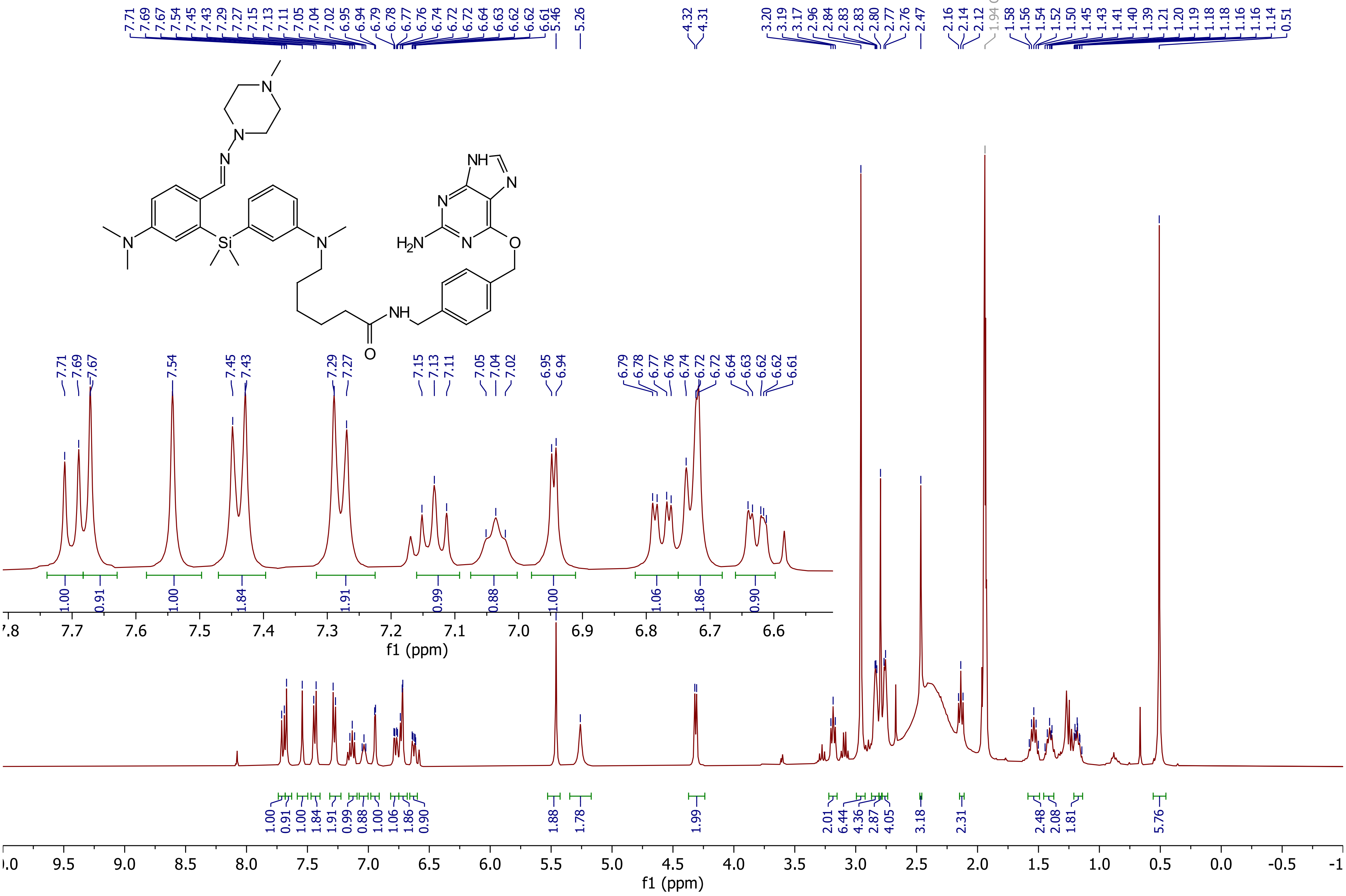

Compound PULI640-P-SNAP

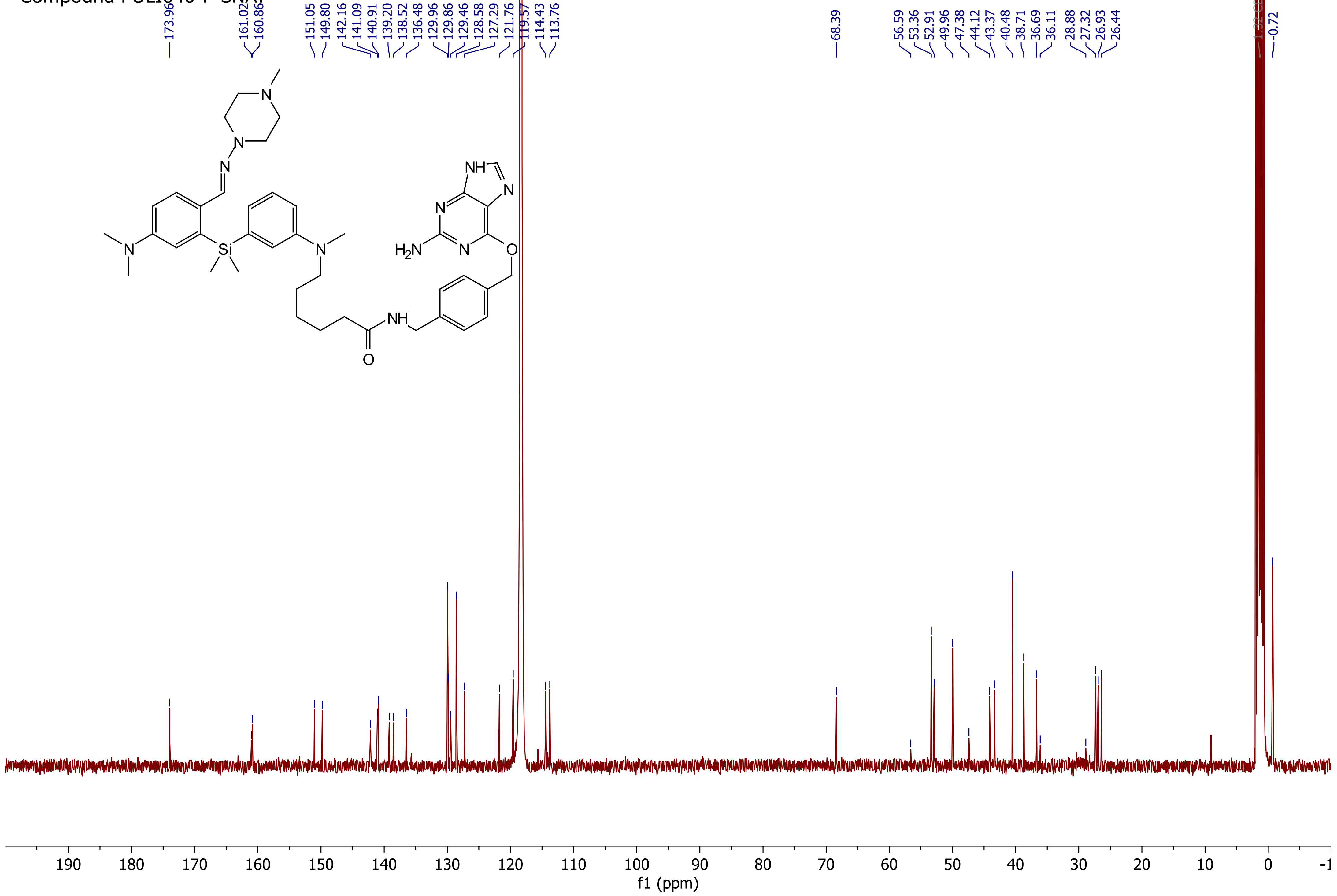

Compound PULI640-T

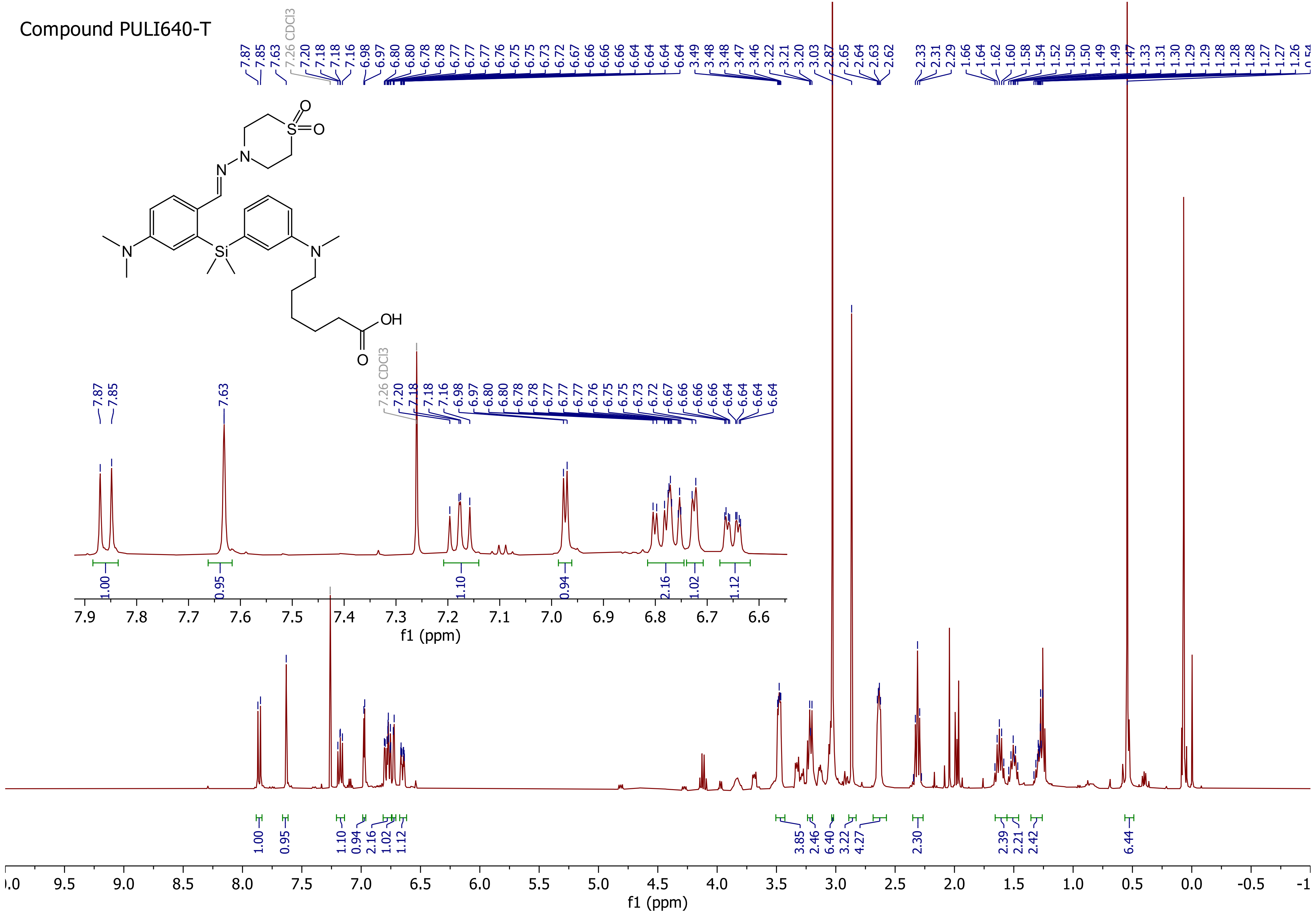

Compound PULI640-T

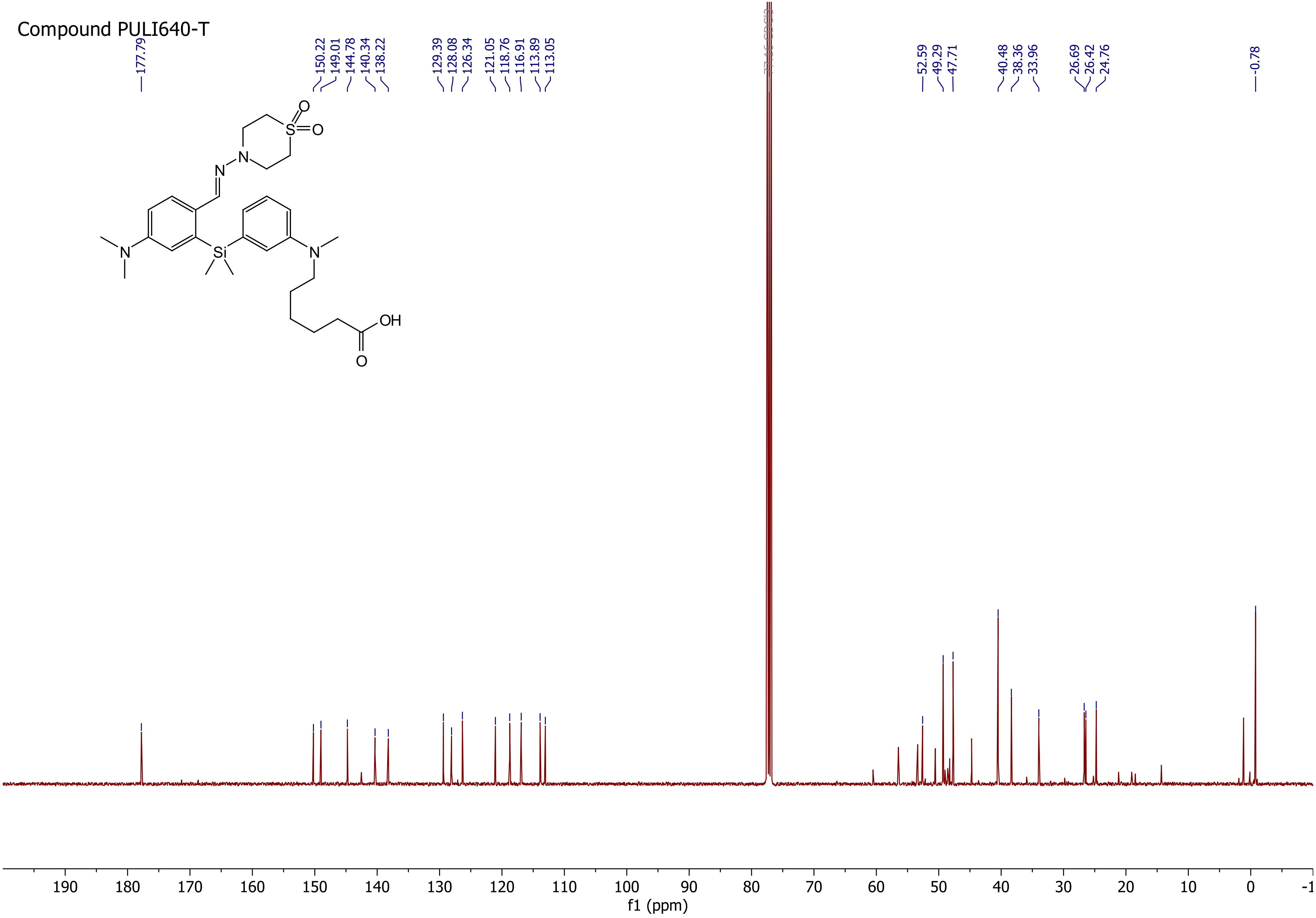

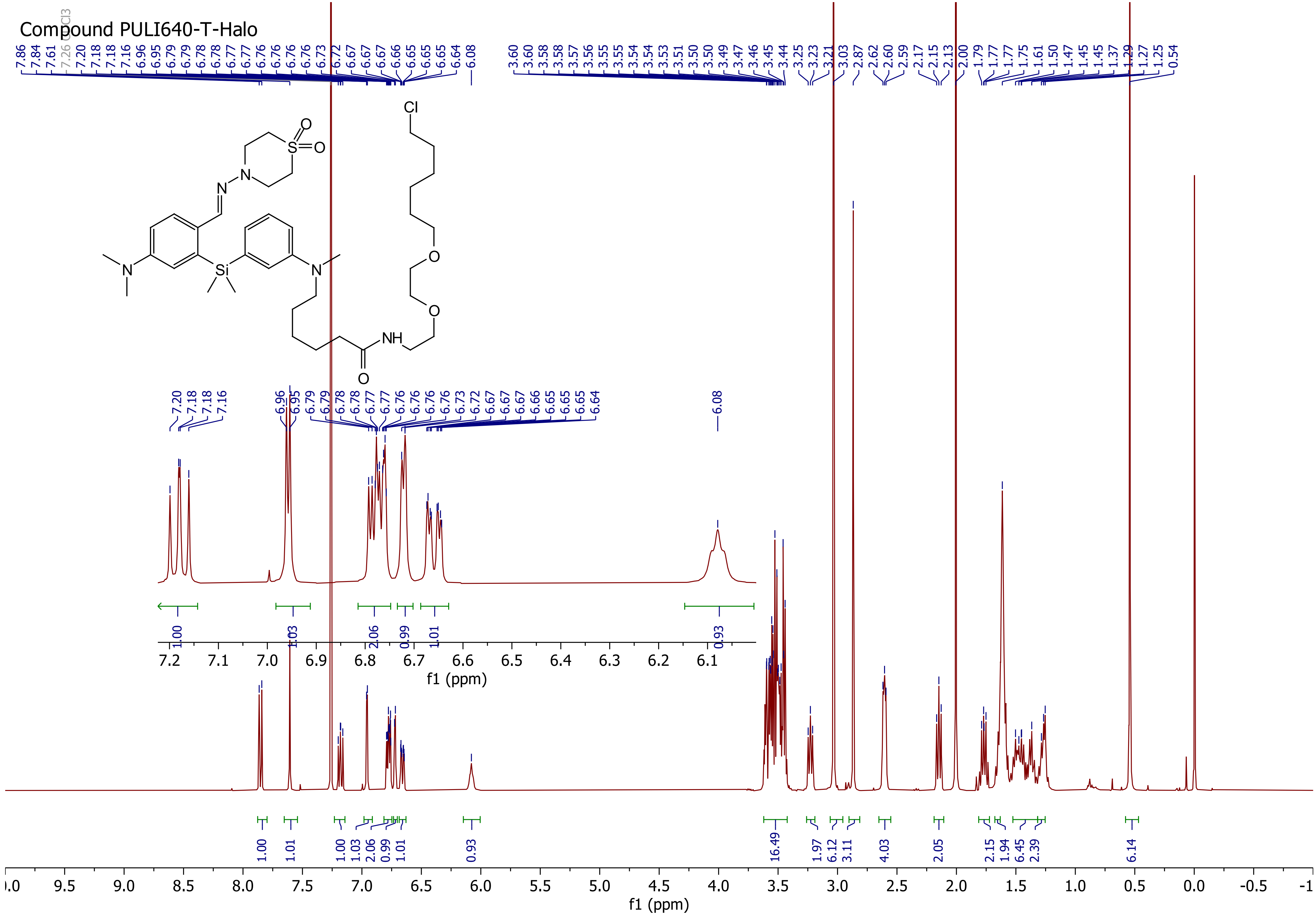
Compound PULI640-T-Halo
f1 (ppm)

Compound PULI640-T-Halo

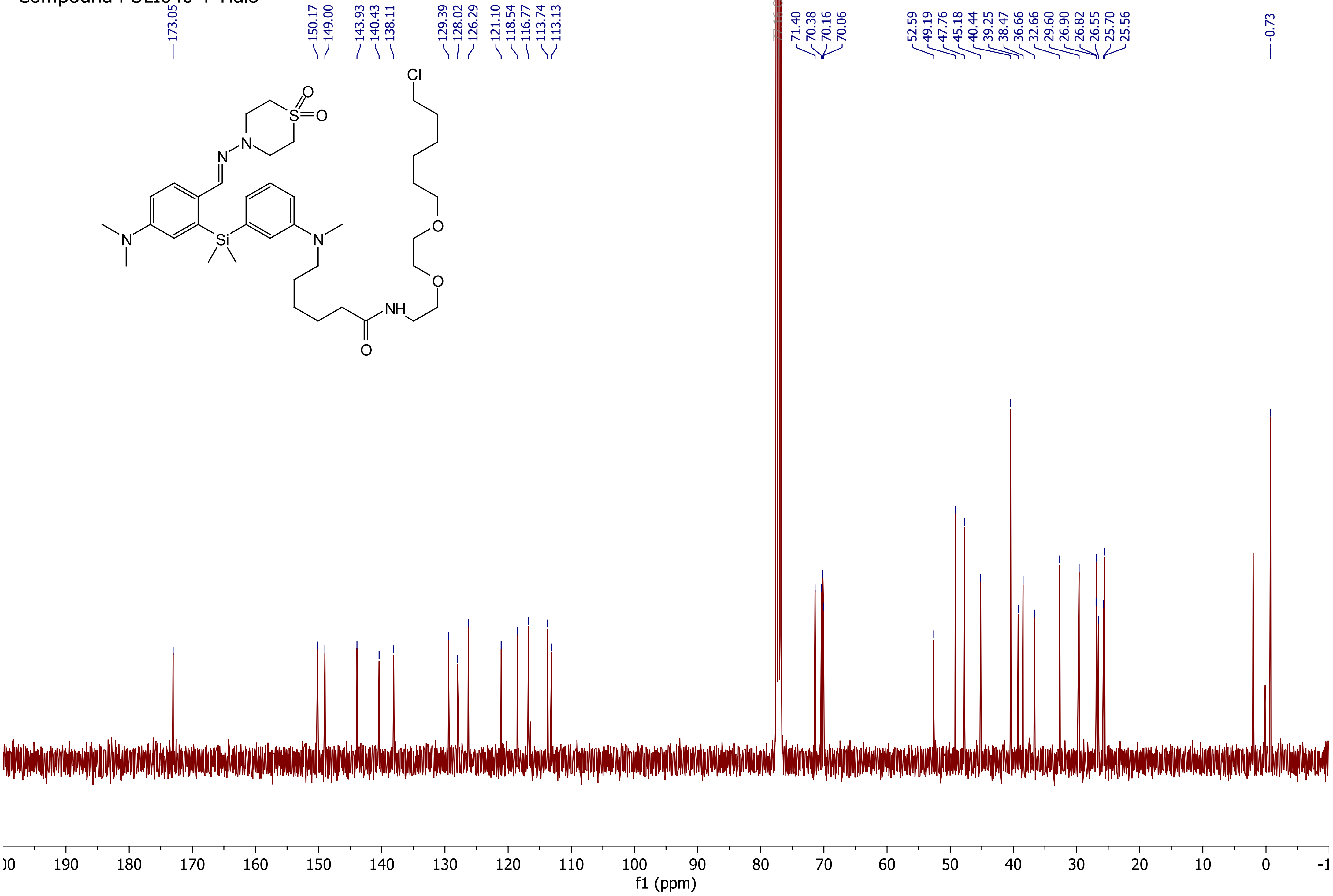

Compound PULI640-T-NHS

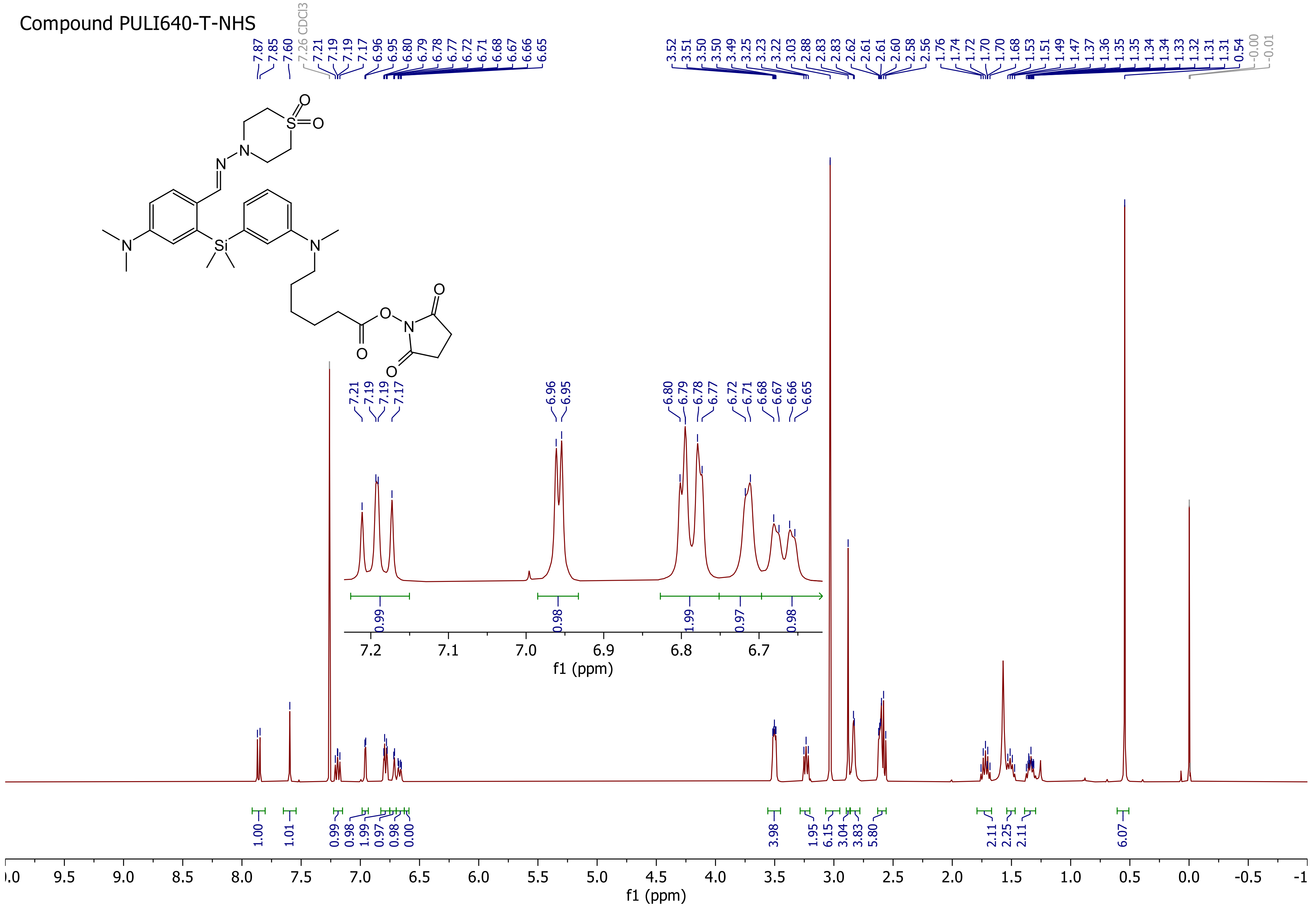

Compound PULI640-T-NHS

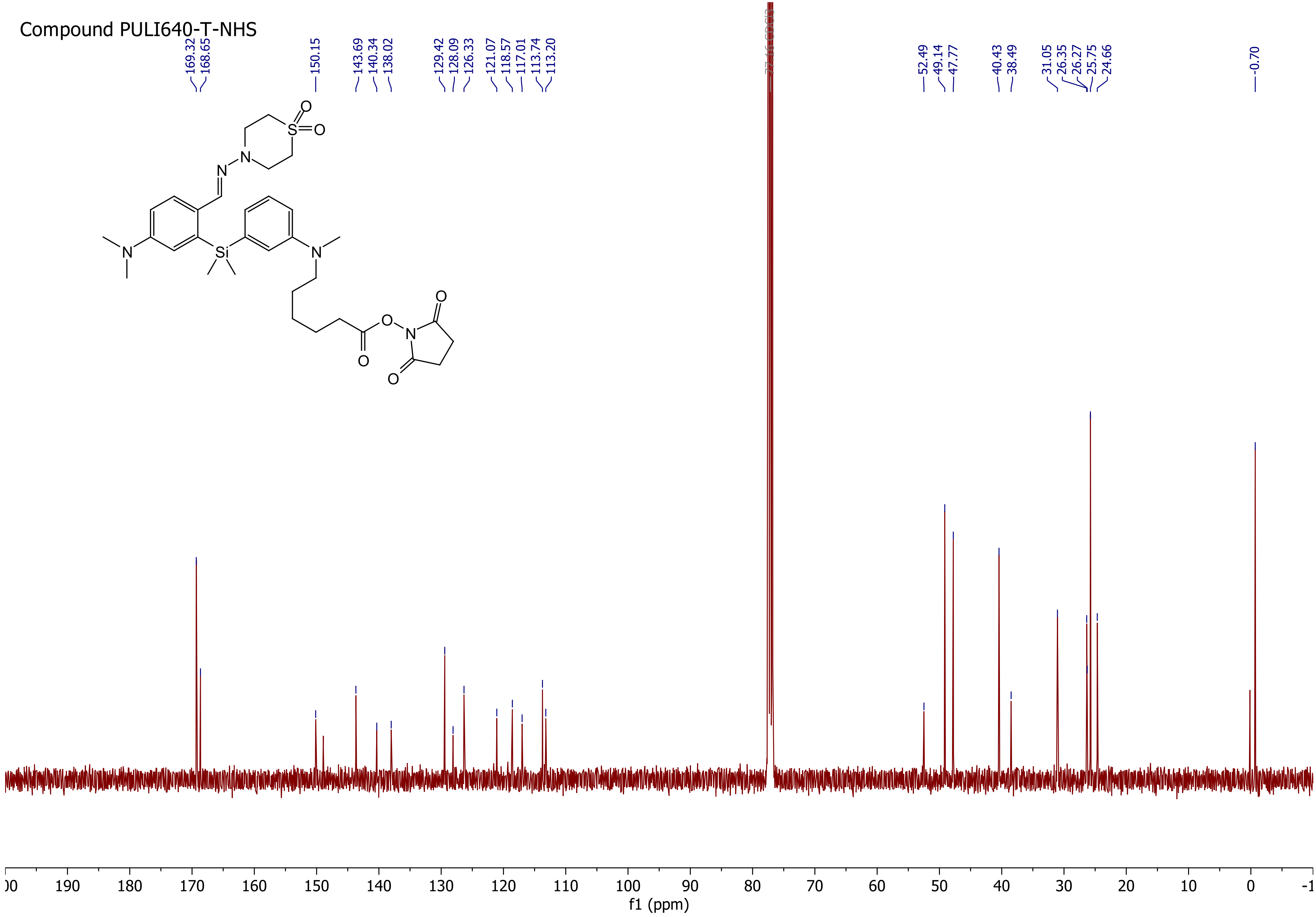

Supplement: Supplementary file 1 — Supporting Information [file ANIE-64-e202506894-s001.pdf]
